# Iron‐Catalyzed Cross‐Coupling of Propargyl Ethers with Grignard Reagents for the Synthesis of Functionalized Allenes and Allenols

**DOI:** 10.1002/anie.202106742

**Published:** 2021-09-02

**Authors:** Daniels Posevins, Aitor Bermejo‐López, Jan‐E. Bäckvall

**Affiliations:** ^1^ Department of Organic Chemistry Arrhenius Laboratory Stockholm University 10691 Stockholm Sweden; ^2^ Department of Natural Sciences Mid Sweden University 85170 Sundsvall Sweden

**Keywords:** cross-coupling, fluoroalkyl allenes, Grignard reagents, iron catalysis

## Abstract

Herein we disclose an iron‐catalyzed cross‐coupling reaction of propargyl ethers with Grignard reagents. The reaction was demonstrated to be stereospecific and allows for a facile preparation of optically active allenes via efficient chirality transfer. Various tri‐ and tetrasubstituted fluoroalkyl allenes can be obtained in good to excellent yields. In addition, an iron‐catalyzed cross‐coupling of Grignard reagents with α‐alkynyl oxetanes and tetrahydrofurans is disclosed herein, which constitutes a straightforward approach towards fully substituted β‐ or γ‐allenols, respectively.

## Graphical abstract

A method for facile synthesis of highly substituted allenols and fluoroalkyl allenes via iron‐catalyzed cross‐coupling reaction of propargyl ethers with Grignard reagents is disclosed. Various tri‐ and tetrasubstituted fluoroalkyl allenes and β‐/γ‐allenols were obtained in good to excellent yields. The reaction was demonstrated to be stereospecific occurring with *syn*‐S_N_2′ displacement of the methoxy group by the Grignard reagent.

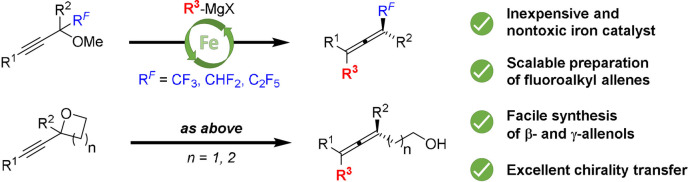

Allenes constitute an interesting class of compounds and have attracted considerable attention in synthetic organic chemistry in recent years.^[1]^ Functionalized allenes are frequently used as building blocks in organic synthesis and they occur in a range of natural products and pharmaceuticals.^[2]^ An interesting feature with functionalized allenes is that they can possess axial chirality.

We[^3^, ^4^, ^5^] and others^[6]^ have recently developed a large number of diverse synthetic methods that rely on the use of various allene‐based starting materials. The development of new procedures for the preparation of allenes is therefore highly desirable. A common route towards functionalized allenes involves the copper‐ or iron‐catalyzed S_N_2′ reaction between Grignard reagents and propargylic substrates.^[7]^ Copper‐catalyzed cross‐couplings between propargylic substrates and Grignard reagents are well known, but to date there are only few reports on the corresponding iron‐catalyzed cross‐couplings and they mainly rely on the use of sulfonates^[8]^ or halides^[9]^ as leaving groups. Fürstner has reported a related method for the preparation of α‐allenols that utilizes alkynyl epoxide with its high ring strain as the nucleofuge (Scheme 1 a).^[10]^ In addition, our group has recently disclosed a practical method for the preparation of highly substituted allenes^[11a]^ and α‐allenols^[11b]^ via iron‐catalyzed cross‐coupling of propargyl carboxylates and Grignard reagents (Scheme 1 b). In the present work we have studied iron‐catalyzed cross‐coupling of less reactive propargylic ethers with Grignard reagents. With methoxy as leaving group the stereospecificity (>99 % *syn*‐displacement) is the highest ever reported in an iron‐catalyzed S_N_2′ substitution of propargylic substrates. Importantly, the low reactivity of the alkoxy group compared to conventional leaving groups allows the presence of fluoroalkyl groups. The use of cyclic analogues, α‐alkynyl oxetanes and tetrahydrofurans as substrates led to synthetically useful allenols.

**Scheme 1:**
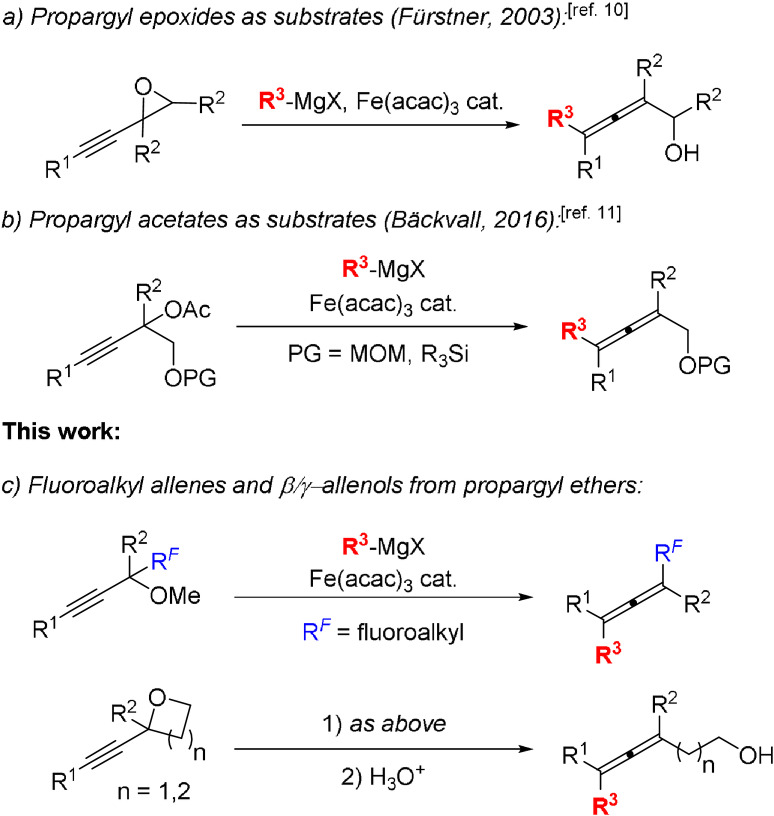
Syntheses of allenes and allenols via Fe‐catalyzed cross‐coupling of Grignard reagents with propargylic substrates.

Our investigations began with the screening of various leaving groups in the reaction of trifluoromethyl group‐containing propargylic substrates **1** in the presence of Fe(acac)_3_ as the catalyst (Table 1). Unexpected formation of *gem*‐difluoro 1,3‐enyne **3** was observed (60 % yield) in the case of the acetate as the leaving group (**1 aa**) and only 20 % yield of the desired trifluoromethyl allene **2 aa** was obtained (entry 1, Table 1). Formation of **3** is thought to proceed via a propargyl radical intermediate.[^12^, ^13^] Also, other oxygen‐based leaving groups tested, such as pivalate, carbonate, phosphonate, and mesylate provided the desired allene **2 aa** in poor yields together with the 1,3‐enyne **3** as the main product (entries 2–5). These results demonstrate the compatibility problems with fluoroalkyl group‐containing substrates in these attempted S_N_2′‐type cross‐coupling reactions. In a try to circumvent this problem we tested methoxy as the leaving group. Interestingly, this leaving group suppressed the undesired formation of the elimination product **3**, and the allene product **2 aa** was now obtained in 94 % yield with no detectable amounts of the side product **3** (entry 6).^[14]^ FeCl_3_ as the catalyst showed similar performance as that of Fe(acac)_3_ in this reaction, albeit providing the desired allene in a slightly decreased 92 % yield (entry 7). Fe(OAc)_2_ as the catalyst failed to give the desired product in this transformation (entry 8). Screening of some commonly used additives did not lead to any increase in yield of allene **2 aa** (entries 9 and 10). The use of CuBr as the catalyst in place of Fe(acac)_3_ did not lead to any detectable amounts of the desired product **2 aa** (entry 11). This observation not only shows that iron is superior to copper as the catalyst in this reaction but also rules out that the reaction is catalyzed by trace amounts of copper in the commercially available Fe(acac)_3_.^[14]^

**Table 1:** Optimization of the reaction conditions.^[a]^ 
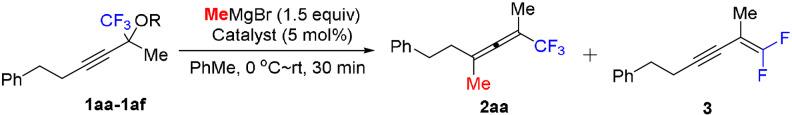

Entry	Substrate (**1**)	Catalyst	Yield of **2 aa** [%]^[b]^	Yield of **3** [%]^[b]^
1	**1 aa** (R=Ac)	Fe(acac)_3_^[c]^	20	60
2	**1 ab** (R=Piv)	Fe(acac)_3_	15	58
3	**1 ac** (R=CO_2_Me)	Fe(acac)_3_	19	69
4	**1 ad** (R=P(O)(OEt)_2_)	Fe(acac)_3_	25	67
5	**1 ae** (R=Ms)	Fe(acac)_3_	18	47
6	**1 af** (R=Me)	Fe(acac)_3_	94 (89)	n.d.
7	**1 af**	FeCl_3_	92	n.d.
8	**1 af**	Fe(OAc)_2_	–^[d]^	n.d.
9^[e]^	**1 af**	Fe(acac)_3_	92	n.d.
10^[f]^	**1 af**	Fe(acac)_3_	91	n.d.
11	**1 af**	CuBr	–^[g]^	n.d.

[a] Reaction conditions: 0.2 M solution of propargylic substrate **1** (0.3 mmol) in PhMe, catalyst (5 mol %) with dropwise addition of Grignard reagent. [b] Determined by NMR using anisole as the internal standard. Isolated yield in parentheses. [c]≥99.9 %. [d] **1 af** was recovered in 93 % yield. [e] Using 20 mol % of TMEDA as an additive. [f] Using 20 mol % of IMes⋅HCl as an additive. [g] **1 af** was recovered in 96 % yield. acac=acetylacetonate. TMEDA=*N*,*N*,*N*,*N*‐tetramethylethylenediamine.

With the optimized reaction conditions in hand, we further studied the reactivity of various fluorinated propargyl methyl ethers **1** (R^3^=fluoroalkyl) in this transformation (Table 2). The use of other Grignard reagents such as *n*‐BuMgCl and PhMgBr in the reaction with **1 af** afforded the corresponding trifluoromethyl allenes **2 ab** and **2 ac** in 43 % and 62 % yields, respectively (entries 2 and 3, Table 2). The observed lower yield of product **2 ab** is most likely due to competing β‐hydride elimination in the alkyliron intermediate initially formed from Fe(acac)_3_ and the Grignard reagent. Allenes **2 b** and **2 c**, containing masked alcohol and aldehyde functionalities, respectively, were prepared in good to high yields from the corresponding substrates **1 b** and **1 c** (entries 4 and 5). Pentafluoroethyl group‐containing substrate **1 d** afforded the corresponding allene **2 d** in 72 % yield (entry 6). Interestingly, TBS‐protected CF_3_ and CHF_2_ group‐containing α‐allenols **2 e** and **2 f** were prepared in 85 % and 77 % yields, respectively, using the newly developed methodology (entry 7). The β‐enallenes **2 g** and **2 h** were obtained from the corresponding **1 g** and **1 h** in 88 % and 75 % yields, respectively (entries 8 and 9). The cross‐coupling reaction with substrate **1 i** afforded allene product **2 i** without any formation of cyclopentyl moiety‐containing product(s) that would be expected from cyclization of an intermediate propargyl radical species (entry 9). Furthermore, cyclopropyl‐substituted propargyl methyl ether **1 j** as the substrate gave **2 j** in 56 % yield, and, interestingly, did not result in any side products arising from the radical ring‐opening of the cyclopropane ring (entry 10). The use of propargyl methyl ether **1 k** gave the desired allene **2 k** in a low 17 % yield, possibly due to an unfavorable coordination of the pyridine moiety to the metal center (entry 11).

**Table 2:** Preparation of fluoroalkyl allenes **2**.^[a]^ 
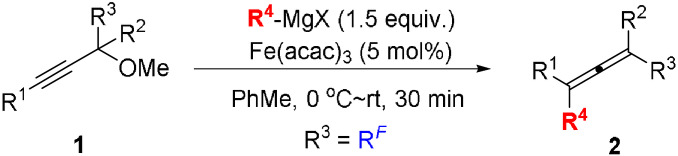

Entry	Substrate (**1**)	R^4^MgX	Product (**2**)	Yield of **2** [%]^[b]^
1	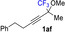	MeMgBr	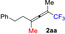	89
2	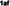	*n*‐BuMgCl	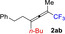	43^[c]^
3	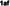	PhMgBr	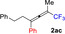	62^[d]^
4	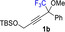	MeMgBr	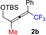	74
5	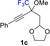	MeMgBr	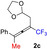	92
6	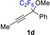	MeMgBr	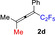	72
7	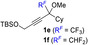	MeMgBr	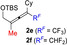	**2 e**: 85 **2 f**: 77
8	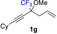	MeMgBr	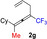	88
9	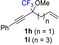	MeMgBr	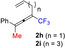	**2 h**: 75 **2 i**: 78
10	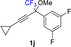	MeMgBr	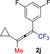	56
11	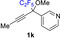	PhMgBr	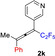	17^**[e]**^

[a] Reaction conditions: 0.2 M solution of propargyl methyl ether **1** (0.3 mmol) in PhMe, Fe(acac)_3_ (5 mol %) with dropwise addition of Grignard reagent (1.5 equiv). [b] Isolated yield. [c] Using 10 mol % of TMEDA as an additive. [d] 2.0 equiv of PhMgBr was used and the reaction time was 4 h. [e] The reaction time was 12 h and **1 k** was recovered in 43 % yield.

The use of 1 equiv of TEMPO as an additive in the reaction of **1 af** with PhMgBr did not completely shut down the reaction, but afforded the desired allene product **2 ac** in 34 % yield (see the Supporting Information). These results strongly suggest, that carbon‐centered radical intermediates are either extremely short‐lived or are not involved in the reaction process.

We were delighted to find that the use of cyclic ether **4 a** as the substrate in the reaction led to formation of the CF_3_ group‐containing γ‐allenol **5 a** in an excellent 88 % yield [Eq. 1]. γ‐Allenols are highly desired substrates for many transition metal‐catalyzed transformations as well as important building blocks in the total synthesis of natural products.^[15]^ The examples of preparation of γ‐allenols currently found in the literature typically involve multistep syntheses.

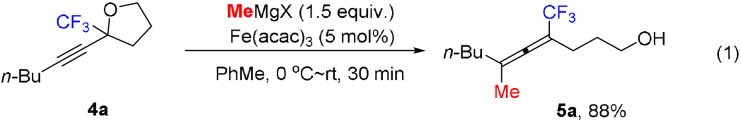




Because of the demand of new efficient methods for the preparation of functionalized allenols we decided to investigate additional α‐alkynyl tetrahydrofurans **4** as substrates in this reaction (Table 3). Tetrahydrofurans **4 b**–**4 e** containing alkyl substituents in the R^1^ and R^2^ positions afforded the corresponding γ‐allenols **5 b**–**5 e** in 56–85 % yields under the standard reaction conditions (entries 2 and 3, Table 3). Interestingly, the reaction tolerates the presence of a free hydroxyl group in substrate **4 e** and afforded the diol product **5 e** in a moderate 56 % yield. α‐Alkynyl tetrahydrofuran **4 f**, containing a hydrogen atom in the R^2^ position, gave the desired trisubstituted γ‐allenols **5 fa** and **5 fb** in good yields (entries 4 and 5). Substrates **4 g** and **4 h** bearing an aryl group in the R^2^ position furnished the corresponding products **5 g** and **5 h** in 65 % and 67 % yields, respectively (entries 6 and 7). Trisubstituted γ‐allenol **5 i** was prepared by cross‐coupling of **4 i** with MeMgBr under the standard reaction conditions in a moderate 55 % yield (entry 8).

**Table 3:** Preparation of γ‐allenols **5**.^[a]^ 
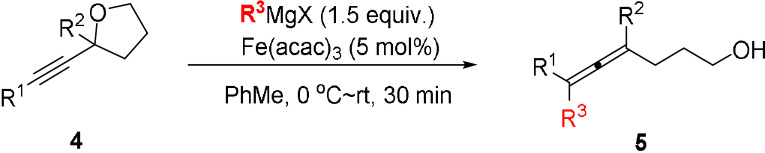

Entry	Substrate (**4**)	R^3^MgX	Product (**5**)	Yield of **5** [%]^[b]^
1	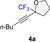	MeMgBr	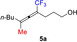	88
2	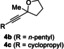	BnMgCl	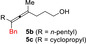	**5 b**: 73 **5 c**: 68
3	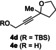	MeMgBr	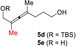	**5 d**: 85 **5 e**: 56^[c]^
4	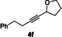	BnMgCl	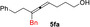	71
5	**4 f**	MeMgBr	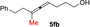	68
6	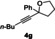	PhMgBr	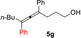	65^[d]^
7	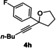	MeMgBr	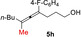	67
8	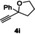	MeMgBr	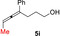	55

[a] Reaction conditions: 0.2 M solution of tetrahydrofuran **4** (0.3 mmol) in PhMe, Fe(acac)_3_ (5 mol %) with dropwise addition of Grignard reagent (1.5 equiv). [b] Isolated yield. [c] 2.5 equiv of MeMgBr was used. [d] 2.0 equiv of PhMgBr was used and the reaction time was 1 h.

To the best of our knowledge, the use of α‐alkynyl oxetanes as substrates has not previously been explored in the Fe‐catalyzed cross coupling with Grignard reagents. Herein we disclose a simple and practical method for accessing synthetically useful β‐allenols from the readily available α‐alkynyl oxetanes **6** as substrates (Scheme 2 and Table S1). β‐Allenols **7 a** and **7 b** were obtained in 78 % and 81 % yields, respectively under the standard reaction conditions via the cross‐coupling of **6 a** and **6 b** with the corresponding Grignard reagents. The use of β‐hydrogen‐containing Grignard reagents such as EtMgBr and CyMgBr in the transformations of oxetanes **6 c** and **6 d** allowed for the preparation of the corresponding products **7 c** and **7 d** in 56 % and 71 % yields, respectively. Trisubstituted β‐allenol **7 e** was obtained in 57 % yield under the standard conditions from the reaction of **6 e** with MeMgBr (entry 5).

**Scheme 2:**
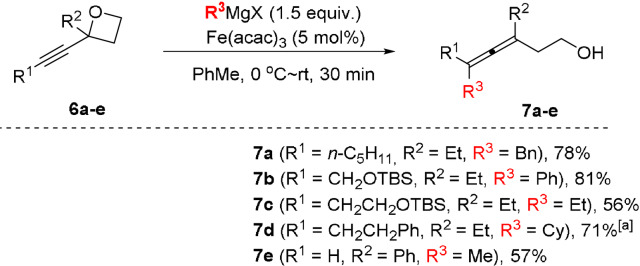
Preparation of β‐allenols **7**. Reaction conditions: 0.2 M solution of oxetane **6** (0.3 mmol) in PhMe, Fe(acac)_3_ (5 mol %) with dropwise addition of Grignard reagent (1.5 equiv).^[a]^ Using 10 mol % of TMEDA as an additive.

To demonstrate the scalability of the herein described iron‐catalyzed synthesis of fluoroalkyl allenes we performed the transformation of propargyl ether **1 e** on a gram‐scale (Scheme 3 a). The desired trifluoromethyl allene **2 e** was obtained in an excellent 88 % yield. To show‐case the potential synthetic utility of the obtained fluoroalkyl allenes, **2 h** was subjected to the regio‐ and stereoselective palladium‐catalyzed oxidative borylation reaction^[16]^ to give the trifluoromethyl group‐containing borylated triene product **8** in 67 % yield (Scheme 3 b).

**Scheme 3:**
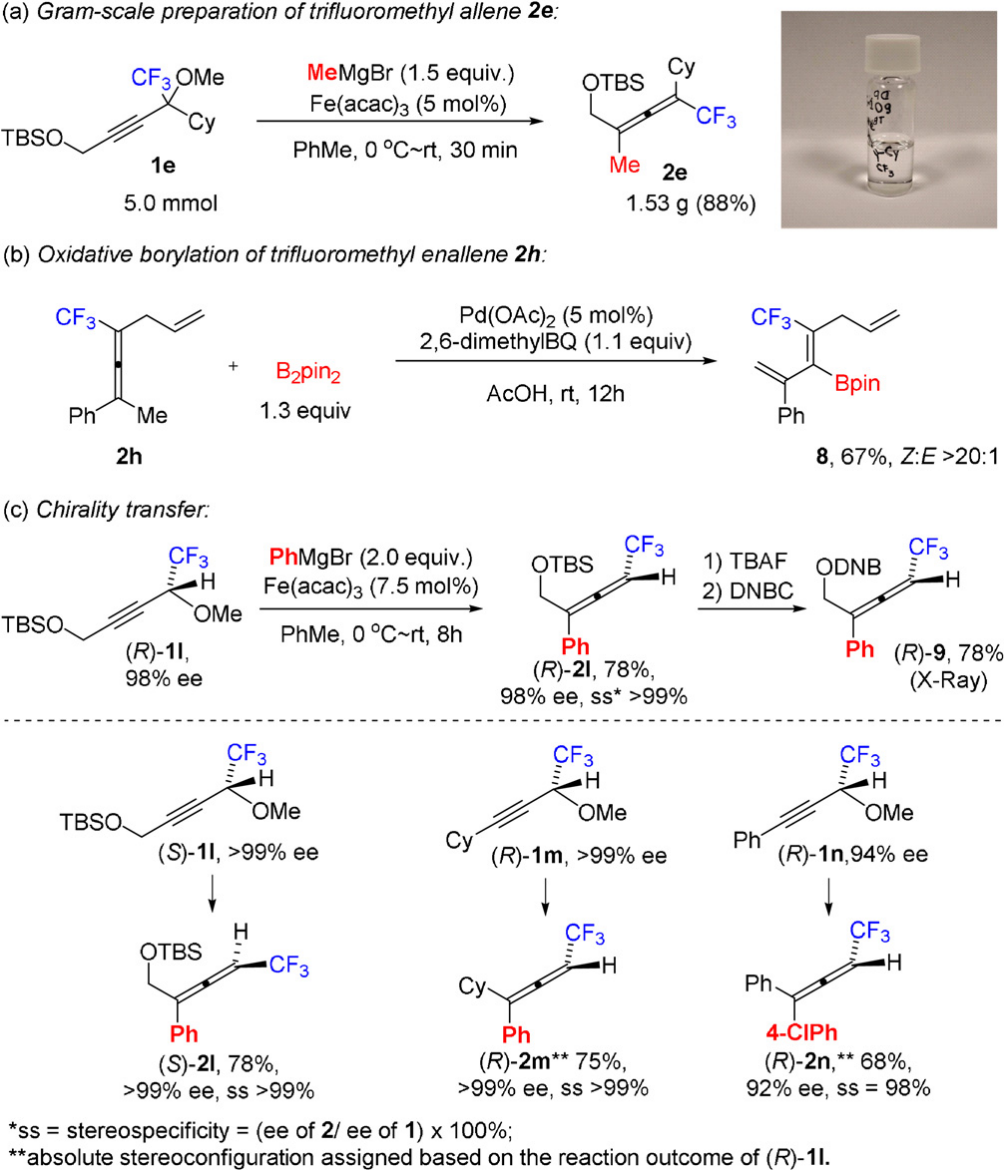
Additional experiments.

We also investigated the stereochemistry of the iron‐catalyzed Grignard reaction (Scheme 3 c). Enantiomerically enriched substrates **1 l**–**n** were conveniently prepared via an enzymatic kinetic resolution of the corresponding secondary propargyl alcohols, followed by methylation using methyl iodide (see the Supporting Information). Interestingly, products **2 l**–**n** were formed under the standard reaction conditions with excellent transfer of chirality from the corresponding propargyl ethers **1 l**–**n** in good yields. Propargylic substrate (*R*)‐**1 l** gave the allene product (*R*)‐**2 l**. The structure and absolute configuration of (*R*)‐**2 l** was established by X‐ray crystallography of its ester derivative (*R*)‐**9** (DNB=2,5‐dinitrobenzoyl). These results show that a *syn*‐S_N_2′ displacement of the methoxy group by the aryl group has occurred, which, in line with the radical probe experiments, rules out the involvement of carbon‐based radical species generated from the substrate (*R*)‐**1 l**.

We also investigated the preparation non‐fluorinated allenes **2 o**–**2 s** using propargyl methyl ethers **1 o**–**1 s** as substrates under the standard reaction conditions (Scheme 4). Substrates **1 o**–**1 p** bearing alkyl substituents in R^1^, R^2^ and R^3^ positions all afforded the corresponding tetrasubstituted allene products **2 o**–**2 p** in good yields with high selectivity. Trisubstituted allene **2 q** was prepared in 92 % yield by reacting TMS group‐containing Grignard reagent with propargyl methyl ether **1 q**. Cross‐coupling of ester group‐containing substrates **1 r** and **1 s** with MeMgBr afforded allenes **2 r** and **2 s** in 51 % and 73 % yields, respectively.

**Scheme 4:**
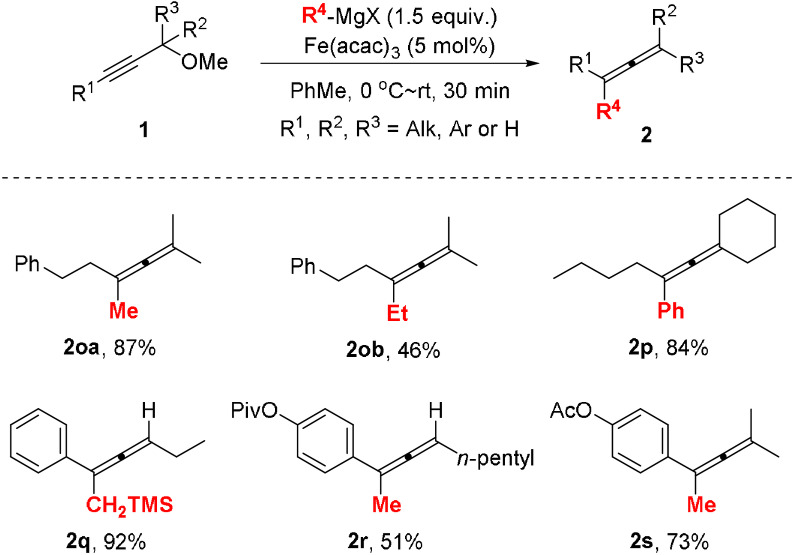
Preparation of non‐fluorinated allenes **2**. Reaction conditions: 0.2 M solution of methyl ether **1** (0.3 mmol) in PhMe, Fe(acac)_3_ (5 mol %) with dropwise addition of Grignard reagent (1.5 equiv).

In the iron‐catalyzed coupling reaction it is thought that the Grignard reagent initially reacts with the iron catalyst Fe(acac)_3_ to generate a reduced organoiron complex, probably an “ate” complex.[^17^, ^18^] Based on the observed transfer of chirality, the reaction is proposed to proceed via a *syn*‐S_N_2′ attack of the initially generated organoiron intermediate on substrate **1** (oxidative addition) to generate ***Int*** **‐B** via ***Int*** **‐A** (Scheme 5). Reductive elimination from ***Int*** **‐B** would give trisubstituted allene product **2** with the observed axial chirality.

**Scheme 5:**
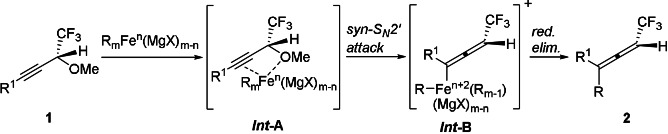
Proposed reaction mechanism.

In conclusion, we have developed a facile method for cross‐coupling of propargyl ethers with Grignard reagents that involves the use of a nontoxic and commercially available iron catalyst. Interestingly, the method allows for the preparation of highly substituted fluoroalkyl allenes as well as for the preparation of β‐ and γ‐allenols (from readily available α‐alkynyl cyclic ethers). The preparation of fluoroalkyl allenes was scalable up to gram‐scale and the use of enantiomerically enriched starting materials led to formation of the desired chiral allenes via a *syn*‐S_N_2′ process with excellent transfer of chirality. To the best of our knowledge this is the highest stereospecificity ever reported in an iron‐catalyzed S_N_2′ substitution reaction of propargylic substrates.^[19]^ Hence, the newly developed transformation constitutes a synthetically useful method for the preparation of chiral allenes.[^11a^, ^20^, ^21^] The results obtained by using radical probes together with the observed transfer of chirality from the substrate to product rules out a radical pathway in the oxidative addition (**1** → ***Int*** **‐B**, Scheme 5). More in‐depth investigations of the reaction mechanism and further applications of the obtained products are currently underway in our laboratory.

## Conflict of interest

The authors declare no conflict of interest.

## Supporting information

As a service to our authors and readers, this journal provides supporting information supplied by the authors. Such materials are peer reviewed and may be re‐organized for online delivery, but are not copy‐edited or typeset. Technical support issues arising from supporting information (other than missing files) should be addressed to the authors.

Supporting Information
