# Supplementary material for: Iron‐Catalyzed Cross‐Coupling of Propargyl Ethers with Grignard Reagents for the Synthesis of Functionalized Allenes and Allenols
Source: Angew Chem Int Ed Engl. 2021 Sep 2;60(41):22178–83. doi: 10.1002/anie.202106742 (PMC8518087; doi:10.1002/anie.202106742)

Angewandte Chemie
Eine Zeitschrift der Gesellschaft Deutscher Chemiker

Supporting Information

Iron-Catalyzed Cross-Coupling of Propargyl Ethers with Grignard Reagents for the Synthesis of Functionalized Allenes and Allenols

*Daniels Posevins, Aitor Bermejo-López, and Jan-E. Bäckvall**

anie_202106742_sm_miscellaneous_information.pdf

Supporting Information

Table of Contents

General information	S1
1. Preparation of starting materials	S2-S20
2. Iron-catalyzed synthesis of allenes	S20-S40
3. Additional experiments	S41-S55
4. References	S56
5. ^{1}H NMR and ^{13}C NMR spectra of compounds	S57-S255

General information

Unless otherwise noted, all reagents were used as received from commercial suppliers. Dry solvents were obtained from commercial sources, from a VAC™ drying system or dried over molecular sieves. $Fe(acac)_3$ was purchased from Sigma-Aldrich (Prod. Nr.: 517003, Lot Nr.: MKBS7930V with a Cu content of 0.5 ppm). Grignard reagents were titrated before use.[1] All reactions were conducted in dry flasks under argon atmosphere. Room temperature is ca. 22 °C. Reactions were monitored using Merck silica gel 60 F254 plates (TLC analysis). TLC plates were visualized with UV light (254 nm) or $KMnO_4$. Flash column chromatography was carried out with 60 Å (particle size 35 - 70 µm) silica gel. ^{1}H-/^{13}C-NMR experiments were performed on a Bruker NMR (400/101 MHz) or (500/125 MHz) at room temperature. ^{19}F-NMR experiments were performed on a 400 MHz Bruker NMR (377 MHz). Chemical shifts (δ) are reported in parts per million (ppm) relative to the $CDCl_3$ peak (δ(H) = 7.26 and δ(C) = 77.16 ppm). Coupling constants (*J*) are reported in Hertz (Hz). HRMS were recorded on a Bruker MicroTOF spectrometer equipped with an ESI or APCI as ion sources. The enantiomeric excess of compounds was determined by chiral GC using racemic compounds as references. GC analyses were performed using the following instruments: a) Varian GC 3900 using an IVADEX-1 chiral column from IVA Analysentechnik with FID detector and N_2 as a carrier gas with a flow of 1.8 mL/min; b) Agilent 8860 GC System using an Hydrodex β-DM chiral column with 5977B GC/MSD detector and N_2 as a carrier gas with a flow of 1.8 mL/min.

Preparation of starting materials

General procedure A for the preparation of substrates from Table 1 entries 1-5:

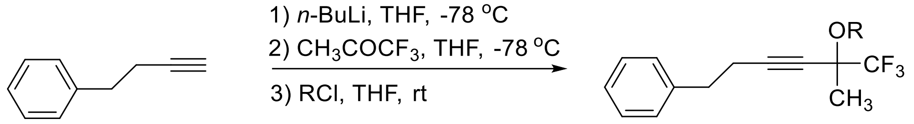

1aa (R=Ac)
1ab (R=Piv)
1ac (R=CO_2Me)
1ad (R=P(O)$(OEt)_2$)
1ae (R=Ms)

To anhydrous THF (10.0 mL) was added *n*-BuLi (2.5 M solution in THF, 2.5 mL, 6.25 mmol, 1.25 equiv.) under Ar atmosphere and the flask was cooled to –78 °C. Then, 4-phenyl-1-butyne (651 mg, 5.0 mmol, 1.00 equiv.) was added dropwise. After 30 minutes at –78 °C, 1,1,1-trifluoroacetone (670 µL, 7.5 mmol, 1.50 equiv.) in anhydrous THF (2.0 mL) was added dropwise. The reaction mixture was allowed to warm to room temperature and stirred for 2 h. The reaction mixture was cooled to 0 °C and the corresponding RCl reagent (10.0 mmol, 2.0 equiv.) was added dropwise. The mixture was allowed to reach room temperature and stirred overnight. After completion, sat. aq. NH_4Cl was added and the mixture was extracted with diethyl ether, washed with sat. aq. $NaHCO_3$, water and dried over Na_2SO_4. The crude product was purified over SiO_2 (eluent: ethyl acetate/pentane) to afford the corresponding alcohol esters **1aa-1ae** as products.

General procedure B for the preparation of α-trifluoromethyl tertiary propargyl methyl ethers <u>1af</u>, <u>1b</u>, <u>1d</u>, <u>1e</u>, <u>1f</u>, and <u>1j</u>:

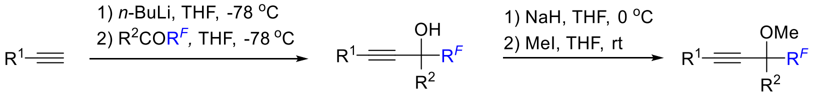

1af (R^1 = $PhCH_2CH_2$; R^2 = Me; R^F = CF_3)
1b (R^1 = $TBSOCH_2$; R^2 = Ph; R^F = CF_3)
1d (R^1 = Me; R^2 = Ph; R^F = CF_3)
1e (R^1 = $TBSOCH_2$; R^2 = Cy; R^F = CF_3)
1f (R^1 = $TBSOCH_2$; R^2 = Cy; R^F = CHF_2)
1j (R^1 = cyclopropyl; R^2 = 3,5-$F_2C_6H_3$; R^F = CF_3)

To a solution of alkyne (1.0 eq) in THF (0.5 M) was added dropwise *n*-BuLi (1.1 eq) at -78 °C under Ar atmosphere. The mixture was stirred for 1 h at -78 °C and the trifluoromethyl ketone (1.2 eq) was added. After the complete consumption of the alkyne (as monitored by TLC), the mixture was quenched with sat. aq. NH_4Cl solution and extracted with diethyl ether. Combined organic phases were washed with brine, dried over Na_2SO_4 and concentrated in vacuo. Purification over SiO_2 (eluent: ethyl acetate/pentane) afforded the corresponding α-trifluoromethyl tertiary propargyl alcohols.

To a solution of the propargyl alcohol (1.00 equiv) in anhydrous THF at 0 °C was slowly added sodium hydride (1.50 equiv). After stirring the mixture at 0 °C for 40 min, methyl iodide (3.00 equiv) was added. The solution was allowed to reach room temperature and stirred for additional 2 – 12 hours (reaction progress was monitored by TLC). The resulting mixture was carefully quenched with sat. aq. NH_4Cl solution and extracted with diethyl ether. The combined ether extracts were concentrated in vacuo and the crude products were purified over SiO_2 (eluent: ethyl acetate/pentane) to yield the desired propargyl methyl ethers as products.

***General procedure C* for the preparation of α-trifluoromethyl tertiary propargyl methyl ethers <u>1c</u>, <u>1g</u>, <u>1h</u> and <u>1i</u>:**

Alkynyl trifluoromethyl ketones **S1a**[2] and **S1b**[3] were prepared in accordance with the previously reported procedures.

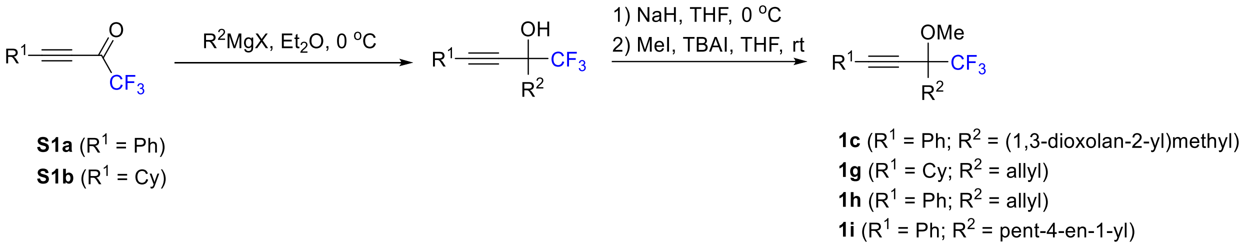

A solution of Grignard reagent (1.5 equiv.) was added dropwise to a stirred solution of trifluoromethyl ketone **S1a** or **S1b** (1.0 equiv.) in dry diethyl ether (0.5 M) at 0 °C under Ar atmosphere. The mixture was stirred for 3 h at 0 °C, and then carefully quenched with sat. aq. NH_4Cl solution. The organic layer was separated, and the aqueous layer was extracted diethyl ether. The combined organic layers were dried over Na_2SO_4, filtered, and concentrated in vacuo. Purification over SiO_2 (eluent: ethyl acetate/pentane) afforded the corresponding α-trifluoromethyl tertiary propargyl alcohols.

To a solution of the propargyl alcohol (1.00 equiv) in anhydrous THF at 0 °C was slowly added sodium hydride (1.50 equiv). After stirring at 0 °C for 40 min, methyl iodide (3.00 equiv) and tetrabutylammonium iodide (20 mol%) were added. The solution was allowed to reach room temperature and stirred for an additional 6 – 12 hours (reaction progress was monitored by TLC). The resulting mixture was carefully quenched with sat. aq. NH_4Cl solution and extracted with diethyl ether. The combined ether extracts were concentrated in vacuo and the crude products were

purified over SiO_2 (eluent: ethyl acetate/pentane) to yield the desired propargyl methyl ethers as products.

General procedure D **for the preparation of α-alkynyl tetrahydrofurans 4b-4d, 4f-4i and α-alkynyl oxetanes 6a-6e:**

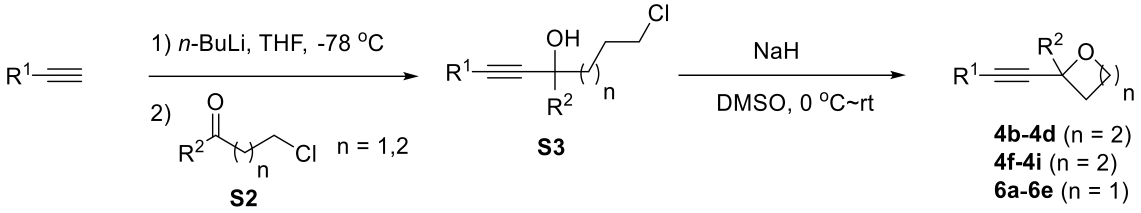

To a solution of alkyne (1.00 equiv) in THF (0.5 M) was added dropwise *n*-BuLi (1.10 equiv) at -78 °C under Ar atmosphere. The mixture was stirred for 1 h at -78 °C and the ketone **S2** (1.20 equiv) was added. After the complete consumption of the alkyne (as monitored by TLC), the mixture was quenched with sat. aq. NH_4Cl solution and extracted with diethyl ether. Combined organic phases were washed with brine, dried over Na_2SO_4 and concentrated in vacuo. Purification over SiO_2 (eluent: ethyl acetate/pentane) afforded the intermediate products **S3**.

To a solution of the propargyl alcohol **S3** (1.00 equiv) in anhydrous DMSO at 0 °C was slowly added sodium hydride (1.50 equiv). This reaction mixture was stirred at room temperature for 1-12 hours (reaction progress was monitored by TLC). After completion, the reaction mixture was carefully quenched with sat. aq. NH_4Cl solution and extracted with diethyl ether. The combined ether extracts were concentrated in vacuo and the crude products were purified over SiO_2 (eluent: ethyl acetate/pentane) to yield the desired α-alkynyl cyclic ether products.

Preparation of α-alkynyl tetrahydrofuran *3-(2-Methyltetrahydrofuran-2-yl)prop-2-yn-1-ol* (4e):

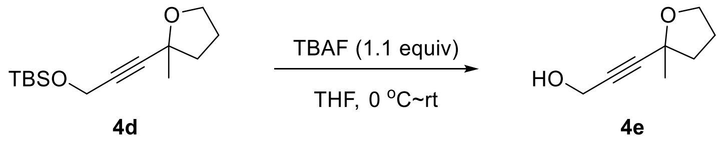

Silyl ether **4d** (254 mg, 1.0 mmol, 1.00 equiv) was dissolved in anhydrous THF (5mL) under Ar atmosphere and the reaction mixture was cooled to 0 °C. Then TBAF (1.0 M in THF, 1.1 mL, 1.1 mmol, 1.10 equiv) was added dropwise over 10 min and the reaction mixture was allowed to warm up to room temperature. After full consumption of starting material (2 h) the reaction was quenched

with water (5 mL), extracted with diethyl ether, and the combined organic phases were dried over Na_2SO_4. The evaporated crude mixture was purified over SiO_2 (eluent: ethyl acetate/pentane 20 – 50%) to yield the desired propargyl alcohol **4e** (107 mg, 76%) as a colorless oil. ^{1}H NMR (400 MHz, $CDCl_3$) δ 4.32 – 4.24 (m, 2H), 3.99 – 3.87 (m, 2H), 2.19 (ddd, J = 12.0, 8.1, 4.4 Hz, 1H), 2.13 – 2.03 (m, 1H), 2.01 – 1.89 (m, 1H), 1.84 – 1.73 (m, 2H), 1.53 (s, 3H); ^{13}C NMR (101 MHz, $CDCl_3$) δ 89.0, 81.0, 76.1, 67.8, 51.3, 40.1, 27.8, 25.8; HRMS (ESI): calc. for $C_8H_{12}NaO_2$ [M+Na]$^+$: 163.0730; found: 163.0735.

Characterization of compounds:

1,1,1-Trifluoro-2-methyl-6-phenylhex-3-yn-2-yl acetate (**1aa**)

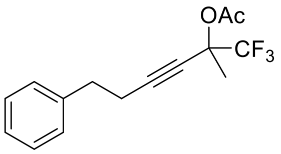

1aa

Following procedure A starting from 4-phenyl-1-butyne (5.0 mmol) and using acetyl chloride. Purification over SiO_2 (eluent: ethyl acetate/pentane 5%).

1.05 g (75% overall yield), colorless oil. ^{1}H NMR (400 MHz, $CDCl_3$) δ 7.32 – 7.27 (m, 2H), 7.22 (ddt, J = 7.0, 3.1, 1.3 Hz, 3H), 2.83 (t, J = 7.4 Hz, 2H), 2.53 (t, J = 7.4 Hz, 2H), 2.09 (s, 3H), 1.82 (q, J = 1.1 Hz, 3H); ^{13}C NMR (101 MHz, $CDCl_3$) δ 167.94, 140.30, 128.67 (2C), 128.49 (2C), 126.50, 123.12 (q, J = 282.9 Hz), 88.52, 73.88 (q, J = 32.7 Hz), 73.72, 34.49, 21.72, 21.09, 20.77; ^{19}F NMR (377 MHz, $CDCl_3$) δ -81.76; HRMS (ESI): calc. for $C_{15}H_{15}F_3NaO_2$ [M+Na]$^+$: 307.0916; found: 307.0925.

1,1,1-Trifluoro-2-methyl-6-phenylhex-3-yn-2-yl pivalate (**1ab**)

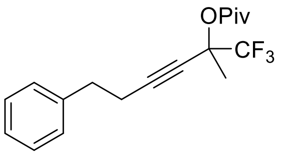

1ab

Following procedure A starting from 4-phenyl-1-butyne (5.0 mmol) and using pivaloyl chloride. Purification over SiO_2 (eluent: ethyl acetate/pentane 5%).

1.42 g (87% overall yield), colorless oil. ^{1}H NMR (400 MHz, $CDCl_3$) δ 7.32 – 7.25 (m, 2H), 7.24 – 7.18 (m, 3H), 2.83 (t, J = 7.4 Hz, 2H), 2.53 (t, J = 7.4 Hz, 2H), 1.80 (q, J = 1.1 Hz, 3H), 1.20 (s,

9H); ^{13}C NMR (101 MHz, $CDCl_3$) δ 175.20, 140.20, 128.51 (2C), 128.35 (2C), 126.34, 123.15 (q, *J* = 283.0 Hz), 88.02, 73.64, 73.48 (q, *J* = 32.5 Hz), 39.40, 34.40, 26.81 (3C), 26.52, 20.92; ^{19}F NMR (377 MHz, $CDCl_3$) δ -81.88; HRMS (ESI): calc. for $C_{18}H_{21}F_3NaO_2$ $[M+Na]^+$: 349.1386; found: 349.1395.

Methyl (1,1,1-trifluoro-2-methyl-6-phenylhex-3-yn-2-yl) carbonate (**1ac**)

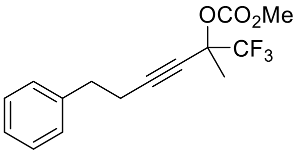

1ac

Following procedure A starting from 4-phenyl-1-butyne (5.0 mmol) and using methyl chloroformate.

Purification over SiO_2 (eluent: ethyl acetate/pentane 10%).

825 mg (55% overall yield), colorless oil. ^{1}H NMR (400 MHz, $CDCl_3$) δ 7.34 – 7.27 (m, 2H), 7.27 – 7.19 (m, 3H), 3.81 (s, 3H), 2.85 (t, *J* = 7.4 Hz, 2H), 2.56 (t, *J* = 7.4 Hz, 2H), 1.87 (q, *J* = 1.1 Hz, 3H); ^{13}C NMR (101 MHz, $CDCl_3$) δ 152.28, 140.18, 128.64 (2C), 128.50 (2C), 126.53, 122.78 (q, *J* = 283.2 Hz), 89.24, 75.52 (q, *J* = 33.0 Hz), 73.29, 55.05, 34.39, 21.07, 20.70; ^{19}F NMR (377 MHz, $CDCl_3$) δ -81.51; HRMS (ESI): calc. for $C_{15}H_{15}F_3NaO_3$ $[M+Na]^+$: 323.0866; found: 323.0870.

Diethyl (1,1,1-trifluoro-2-methyl-6-phenylhex-3-yn-2-yl) phosphate (**1ad**)

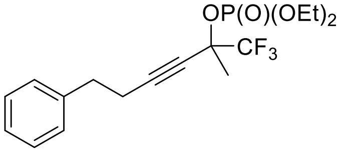

1ad

Following procedure A starting from 4-phenyl-1-butyne (5.0 mmol) and using diethyl chlorophosphate.

Purification over SiO_2 (eluent: ethyl acetate/pentane 10%).

905 mg (48% overall yield), colorless oil. ^{1}H NMR (400 MHz, $CDCl_3$) δ 7.32 – 7.25 (m, 2H), 7.23 – 7.17 (m, 3H), 4.18 – 4.00 (m, 4H), 2.84 (t, *J* = 7.5 Hz, 2H), 2.59 – 2.51 (m, 2H), 1.85 (q, *J* = 1.2 Hz, 3H), 1.31 (dtd, *J* = 9.7, 7.1, 1.1 Hz, 6H); ^{13}C NMR (101 MHz, $CDCl_3$) δ 140.06, 128.56 (2C), 128.50 (2C), 126.53, 127.30 – 118.27 (m), 89.91, 75.05 – 73.79 (m, 2C), 64.34 (d, *J* = 6.6 Hz),

64.10 (d, J = 6.1 Hz), 34.29, 23.06, 21.09, 16.05, 15.98; ^{19}F NMR (377 MHz, $CDCl_3$) δ -82.48; HRMS (ESI): calc. for $C_{17}H_{22}F_3NaO_4P$ $[M+Na]^+$: 401.1100; found: 401.1107.

1,1,1-Trifluoro-2-methyl-6-phenylhex-3-yn-2-yl methanesulfonate (**1ae**)

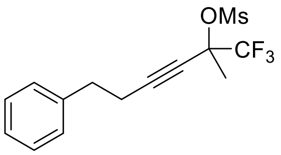

1ae

Following procedure A starting from 4-phenyl-1-butyne (5.0 mmol) and using mesyl chloride. Purification over SiO_2 (eluent: ethyl acetate/pentane 5%).

1.09 g (68% overall yield), colorless oil. ^{1}H NMR (400 MHz, $CDCl_3$) δ 7.35 – 7.26 (m, 2H), 7.27 – 7.18 (m, 3H), 2.96 – 2.80 (m, 5H), 2.65 (td, J = 7.1, 2.0 Hz, 2H), 1.90 (q, J = 1.0 Hz, 3H); ^{13}C NMR (101 MHz, $CDCl_3$) δ 139.72, 128.71 (2C), 128.68 (2C), 126.78, 122.16 (q, J = 283.7 Hz), 92.71, 78.80 (q, J = 34.2 Hz), 72.30, 40.10, 33.94, 23.51, 21.01; ^{19}F NMR (377 MHz, $CDCl_3$) δ -81.47; HRMS (ESI): calc. for $C_{14}H_{15}F_3NaO_3S$ $[M+Na]^+$: 343.0586; found: 343.0592.

Preparation of (6,6,6-Trifluoro-5-methoxy-5-methylhex-3-yn-1-yl)benzene (**1af**)

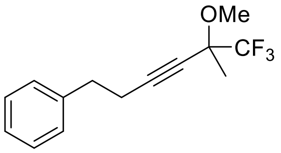

1af

Following procedure B using 4-phenyl-1-butyne (10.0 mmol) and 1,1,1-trifluoroacetone. Purification over SiO_2 (eluent: ethyl acetate/pentane 5%).

1.67 g (65% overall yield), colorless oil. ^{1}H NMR (400 MHz, $CDCl_3$) δ 7.34 – 7.27 (m, 2H), 7.25 – 7.19 (m, 3H), 3.38 (t, J = 1.0 Hz, 3H), 2.86 (t, J = 7.3 Hz, 2H), 2.58 (t, J = 7.3 Hz, 2H), 1.51 (t, J = 1.1 Hz, 3H); ^{13}C NMR (101 MHz, $CDCl_3$) δ 140.2, 128.5 (2C), 128.6 (2C), 126.6, 124.3 (q, J = 285.0 Hz), 89.2, 74.5, 74.2 (q, J = 30.8 Hz), 53.4, 34.7, 22.1, 20.9; ^{19}F NMR (377 MHz, $CDCl_3$) δ -79.85; HRMS (ESI): calc. for $C_{14}H_{15}F_3NaO$ $[M+Na]^+$: 279.0967; found: 279.0965.

tert-Butyldimethyl((5,5,5-trifluoro-4-methoxy-4-phenylpent-2-yn-1-yl)oxy)silane (**1b**)

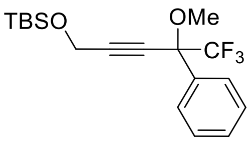

1b

Following procedure B using *tert*-butyldimethyl(prop-2-yn-1-yloxy)silane (5.0 mmol) and 2,2,2-trifluoroacetophenone.

Purification over SiO_2 (eluent: ethyl acetate/pentane 5%).

1.18 g (66% overall yield), colorless oil. ^{1}H NMR (400 MHz, $CDCl_3$) δ 7.74 – 7.66 (m, 2H), 7.48 – 7.37 (m, 3H), 4.52 (s, 2H), 3.39 (s, 3H), 0.94 (s, 9H), 0.16 (d, *J* = 1.2 Hz, 6H); ^{13}C NMR (101 MHz, $CDCl_3$) δ 133.3, 129.8 (2C), 128.5 (2C), 128.4 (2C), 123.1 (q, *J* = 285.1 Hz), 90.0, 80.0 (q, *J* = 31.2 Hz), 53.2, 51.7, 25.8 (3C), 18.4, -5.1, -5.1; ^{19}F NMR (377 MHz, $CDCl_3$) δ -78.52; HRMS (ESI): calc. for $C_{18}H_{25}F_3NaO_2Si$ $[M+Na]^+$: 381.1468; found: 381.1470.

2-(2-Methoxy-4-phenyl-2-(trifluoromethyl)but-3-yn-1-yl)-1,3-dioxolane (**1c**)

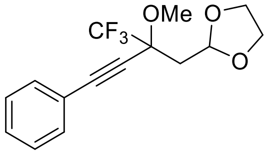

1c

Following procedure C using **S1a** (3.0 mmol) and (1,3-dioxolan-2-ylmethyl)magnesium bromide.

Purification over SiO_2 (eluent: ethyl acetate/pentane 10%).

765 mg (85% overall yield), colorless oil. ^{1}H NMR (400 MHz, $CDCl_3$) δ 7.56 – 7.47 (m, 2H), 7.44 – 7.31 (m, 3H), 5.27 (t, *J* = 4.4 Hz, 1H), 4.08 – 3.94 (m, 2H), 3.94 – 3.83 (m, 2H), 3.65 (q, *J* = 1.1 Hz, 3H), 2.38 – 2.26 (m, 2H); ^{13}C NMR (101 MHz, $CDCl_3$) δ 132.2 (2C), 129.6, 128.6 (2C), 124.2 (q, *J* = 288.5 Hz), 121.3, 101.5, 91.2, 80.1, 75.7 (q, *J* = 30.3 Hz), 65.1, 64.9, 55.2 (d, *J* = 1.4 Hz), 40.3; ^{19}F NMR (377 MHz, $CDCl_3$) δ -77.70; HRMS (ESI): calc. for $C_{15}H_{15}F_3NaO_3$ $[M+Na]^+$: 323.0866; found: 323.0871.

(1,1,1,2,2-Pentafluoro-3-methoxyhex-4-yn-3-yl)benzene (**1d**)

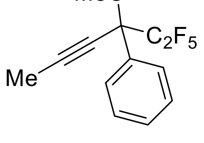

1d

Following procedure B using 1-propynylmagnesium bromide (5.0 mmol) and 2,2,3,3,3-pentafluoro-1-phenylpropan-1-one.

Purification over SiO_2 (eluent: ethyl acetate/pentane 5%).

905 mg (65% overall yield), colorless oil. ^{1}H NMR (400 MHz, $CDCl_3$) δ 7.71 – 7.60 (m, 2H), 7.47 – 7.37 (m, 3H), 3.35 (s, 3H), 2.03 (s, 3H); ^{13}C NMR (101 MHz, $CDCl_3$) δ 133.6, 129.6 (2C), 128.9, 128.2 (2C), 125.0 – 106.9 (m, 2C), 88.7, 80.5 – 79.5 (m), 72.0 (d, J = 5.0 Hz), 52.7 (d, J = 1.3 Hz), 3.8; ^{19}F NMR (377 MHz, $CDCl_3$) δ -77.66, -117.45, -118.17, -121.49, -122.21; HRMS (ESI): calc. for $C_{13}H_{11}F_5NaO$ $[M+Na]^+$: 301.0622; found: 301.0624.

tert-Butyl((4-cyclohexyl-5,5,5-trifluoro-4-methoxypent-2-yn-1-yl)oxy)dimethylsilane (**1e**)

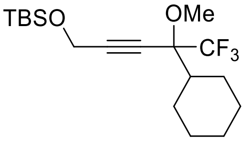

1e

Following procedure B using *tert*-butyldimethyl(prop-2-yn-1-yloxy)silane (12.0 mmol) and 1-cyclohexyl-2,2,2-trifluoroethan-1-one.

Purification over SiO_2 (eluent: ethyl acetate/pentane 10%).

3.15 g (72% overall yield), colorless oil. ^{1}H NMR (500 MHz, $CDCl_3$) δ 4.42 (s, 2H), 3.52 (d, J = 1.1 Hz, 3H), 1.90 (d, J = 10.0 Hz, 2H), 1.84 – 1.73 (m, 3H), 1.69 – 1.61 (m, 1H), 1.30 – 1.08 (m, 5H), 0.91 (s, 9H), 0.13 (s, 6H); ^{13}C NMR (126 MHz, $CDCl_3$) δ 124.8 (q, J = 289.9 Hz), 89.9, 80.5 (q, J = 28.0 Hz), 76.3, 55.5, 51.6, 44.4, 27.3 (q, J = 2.1 Hz), 26.8, 26.3 (2C), 26.2, 25.8 (3C), 18.4, -5.1 (2C); ^{19}F NMR (377 MHz, $CDCl_3$) δ -72.98; HRMS (ESI): calc. for $C_{18}H_{31}F_3NaO_2Si$ $[M+Na]^+$: 387.1938; found: 387.1948.

tert-Butyl((4-cyclohexyl-5,5-difluoro-4-methoxypent-2-yn-1-yl)oxy)dimethylsilane (**1f**)

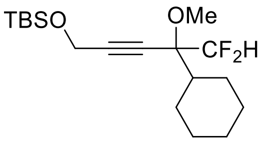

1f

Following procedure B using *tert*-butyldimethyl(prop-2-yn-1-yloxy)silane (4.0 mmol) and 1-cyclohexyl-2,2-difluoroethan-1-one.

Purification over SiO_2 (eluent: ethyl acetate/pentane 10%).

816 mg (59% overall yield), colorless oil. ^{1}H NMR (400 MHz, $CDCl_3$) δ 5.93 – 5.53 (m, 1H), 4.42 (s, 2H), 3.50 (d, *J* = 1.1 Hz, 3H), 1.94 – 1.59 (m, 6H), 1.34 – 1.08 (m, 5H), 0.91 (s, 9H), 0.13 (s, 6H); ^{13}C NMR (101 MHz, $CDCl_3$) δ 115.6 (t, *J* = 251.6 Hz), 89.7, 79.5 (t, *J* = 20.0 Hz), 77.8 (d, *J* = 4.7 Hz), 55.1 (t, *J* = 1.9 Hz), 51.7, 44.0, 27.3, 26.7, 26.7, 26.3, 26.3, 25.8 (3C), 18.4, -5.1 (2C); ^{19}F NMR (377 MHz, $CDCl_3$) δ -125.92, -126.66, -128.26, -129.00; HRMS (ESI): calc. for $C_{18}H_{32}F_2NaO_2Si$ $[M+Na]^+$: 369.2032; found: 369.2040.

(3-Methoxy-3-(trifluoromethyl)hex-5-en-1-yn-1-yl)cyclohexane (**1g**)

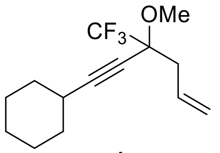

1g

Following procedure C using **S1b** (3.0 mmol) and allylmagnesium bromide.

Purification over SiO_2 (eluent: ethyl acetate/pentane 5%).

538 mg (69% overall yield), colorless oil.^{1}H NMR (500 MHz, $CDCl_3$) δ 5.88 (ddt, *J* = 16.9, 9.7, 7.1 Hz, 1H), 5.21 – 5.12 (m, 2H), 3.51 (s, 3H), 2.64 – 2.47 (m, 3H), 1.74 (dddt, *J* = 45.2, 12.9, 6.7, 3.6 Hz, 4H), 1.51 (ddt, *J* = 18.0, 9.3, 3.4 Hz, 3H), 1.44 – 1.29 (m, 3H); ^{13}C NMR (126 MHz, $CDCl_3$) δ 131.5, 124.5 (q, *J* = 288.1 Hz)z, 119.1, 96.2, 77.0 (q, *J* = 31.9 Hz), 72.0, 54.8, 40.4, 32.3, 32.3, 28.9, 26.0 (2C), 24.6; ^{19}F NMR (377 MHz, $CDCl_3$) δ -77.65; HRMS (ESI): calc. for $C_{14}H_{19}F_3NaO$ $[M+Na]^+$: 283.1280; found: 283.1287.

(3-Methoxy-3-(trifluoromethyl)hex-5-en-1-yn-1-yl)benzene (**1h**)

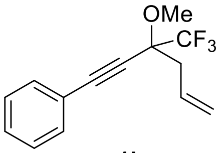

1h

Following procedure C using **S1a** (3.0 mmol) and allylmagnesium bromide.

Purification over SiO_2 (eluent: ethyl acetate/pentane 5%).

630 mg (83% overall yield), colorless oil. ^{1}H NMR (500 MHz, $CDCl_3$) δ 7.52 – 7.48 (m, 2H), 7.42 – 7.32 (m, 3H), 6.05 – 5.89 (m, 1H), 5.28 – 5.19 (m, 2H), 3.62 (q, *J* = 1.0 Hz, 3H), 2.75 – 2.64 (m, 2H); ^{13}C NMR (126 MHz, $CDCl_3$) δ 132.2 (2C), 131.2, 129.5, 128.6 (2C), 124.4 (q, *J* = 288.3 Hz),

121.4, 119.6, 90.9, 80.7, 77.3, 55.2, 40.3; ^{19}F NMR (377 MHz, $CDCl_3$) δ -77.10; HRMS (APCI): calc. for $C_{14}H_{13}F_3OH$ $[M+H]^+$: 255.0991; found: 255.0987.

(3-Methoxy-3-(trifluoromethyl)oct-7-en-1-yn-1-yl)benzene (**1i**)

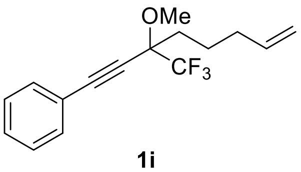

1i

Following procedure C using **S1a** (5.0 mmol) and pent-4-en-1-ylmagnesium bromide.
Purification over SiO_2 (eluent: ethyl acetate/pentane 5%).
917 mg (65% overall yield), colorless oil. ^{1}H NMR (400 MHz, $CDCl_3$) δ 7.54 – 7.45 (m, 2H), 7.41 – 7.30 (m, 3H), 5.82 (ddt, *J* = 16.9, 10.2, 6.7 Hz, 1H), 5.09 – 4.95 (m, 2H), 3.61 (q, *J* = 1.1 Hz, 3H), 2.14 (tdt, *J* = 8.0, 6.7, 1.4 Hz, 2H), 1.95 – 1.86 (m, 2H), 1.79 – 1.68 (m, 2H); ^{13}C NMR (101 MHz, $CDCl_3$) δ 138.3, 132.1 (2C), 129.4, 128.6 (2C), 124.7 (q, *J* = 288.4 Hz), 121.5, 115.3, 90.3, 81.0, 77.77 (q, *J* = 29.4 Hz), 55.2, 35.0, 33.6, 22.8; ^{19}F NMR (377 MHz, $CDCl_3$) δ -77.33; HRMS (ESI): calc. for $C_{16}H_{17}F_3NaO$ $[M+Na]^+$: 305.1124; found: 305.1131.

1-(4-Cyclopropyl-1,1,1-trifluoro-2-methoxybut-3-yn-2-yl)-3,5-difluorobenzene (**1j**)

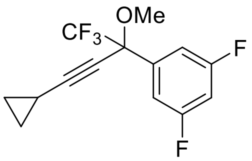

1j

Following procedure B using cyclopropylacetylene (5.0 mmol) and 1-(3,5-difluorophenyl)-2,2,2-trifluoroethan-1-one.
Purification over SiO_2 (eluent: ethyl acetate/pentane 5%).
1.06 g (73% overall yield), colorless oil. ^{1}H NMR (500 MHz, $CDCl_3$) δ 7.21 (h, *J* = 4.6 Hz, 2H), 6.85 (tt, *J* = 8.6, 2.4 Hz, 1H), 3.37 (s, 3H), 1.43 (tt, *J* = 8.3, 5.0 Hz, 1H), 0.93 (ddd, *J* = 8.3, 3.5, 1.5 Hz, 2H), 0.85 (ddd, *J* = 8.5, 4.3, 2.5 Hz, 2H); ^{13}C NMR (126 MHz, $CDCl_3$) δ 163.8 (d, *J* = 12.4 Hz), 161.9 (d, *J* = 12.4 Hz), 138.5 (t, *J* = 8.8 Hz), 122.8 (q, *J* = 285.3 Hz), 111.9 (d, *J* = 6.6 Hz), 111.7 (d, *J* = 6.5 Hz), 105.2 (t, *J* = 25.4 Hz), 96.3, 79.2 (q, *J* = 31.5 Hz), 66.6, 53.3, 8.9, 8.8, -0.5; ^{19}F NMR (377 MHz, $CDCl_3$) δ -80.57, -109.20; HRMS (APCI): calc. for $C_{14}H_{11}F_5OH$ $[M+H]^+$: 291.0803; found: 291.0805.

3-(1,1,1,2,2-Pentafluoro-3-methoxyhex-4-yn-3-yl)pyridine (**1k**)

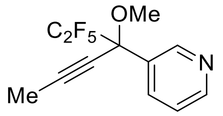

1k

Following procedure B using 1-propynylmagnesium bromide (5.0 mmol) and 2,2,3,3,3-pentafluoro-1-(pyridin-3-yl)propan-1-one.

Purification over SiO_2 (eluent: ethyl acetate/pentane 10%).

890 mg (64% overall yield), colorless oil. ^{1}H NMR (400 MHz, $CDCl_3$) δ 8.88 (dt, J = 1.8, 0.9 Hz, 1H), 8.65 (dd, J = 4.8, 1.6 Hz, 1H), 8.00 – 7.89 (m, 1H), 7.34 (ddd, J = 8.0, 4.8, 0.9 Hz, 1H), 3.35 (s, 3H), 2.04 (s, 3H); ^{13}C NMR (101 MHz, $CDCl_3$) δ 150.75 (2C), 150.4, 136.5, 129.8, 123.0, 123.4 – 109.0 (m), 90.1, 79.0 – 78.5 (m), 70.8 (d, J = 5.0 Hz), 52.9 (d, J = 1.3 Hz), 3.8; ^{19}F NMR (377 MHz, $CDCl_3$) δ -77.61, -117.39 (d, J = 273.3 Hz), -121.75 (d, J = 273.2 Hz); HRMS (ESI): calc. for $C_{12}H_{10}F_5NNaO$ $[M+Na]^+$: 302.0575; found: 302.0568.

(5-Methoxy-5-methylhex-3-yn-1-yl)benzene (**1o**)

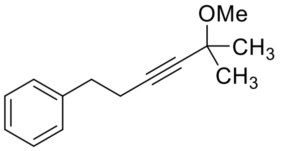

1o

Following procedure B using 4-phenyl-1-butyne (5.0 mmol) and acetone.

Purification over SiO_2 (eluent: ethyl acetate/pentane 5%).

550 mg (55% overall yield), colorless oil. ^{1}H NMR (400 MHz, $CDCl_3$) δ 7.33 – 7.27 (m, 2H), 7.25 – 7.18 (m, 3H), 3.28 (s, 3H), 2.83 (t, J = 7.5 Hz, 2H), 2.51 (t, J = 7.5 Hz, 2H), 1.40 (s, 6H); ^{13}C NMR (101 MHz, $CDCl_3$) δ 140.8, 128.7 (2C), 128.4 (2C), 126.4, 83.9, 82.9, 70.7, 51.5, 35.3, 28.7 (2C), 21.0; HRMS (ESI): calc. for $C_{14}H_{18}NaO$ $[M+Na]^+$: 225.1250; found: 225.1257.

1-(Hex-1-yn-1-yl)-1-methoxycyclohexane (**1p**)

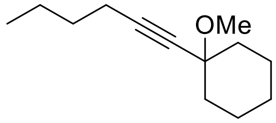

1p

Following procedure B using 1-hexyne (5.0 mmol) and acetone.
Purification over SiO_2 (eluent: ethyl acetate/pentane 5%).
457 mg (47% overall yield), colorless oil. ^{1}H NMR (400 MHz, $CDCl_3$) δ 3.34 (s, 3H), 2.24 (t, J = 6.9 Hz, 2H), 1.89 – 1.79 (m, 2H), 1.73 – 1.35 (m, 12H), 0.91 (t, J = 7.2 Hz, 3H); ^{13}C NMR (101 MHz, $CDCl_3$) δ 86.6, 81.1, 74.2, 50.6, 37.2 (2C), 31.1, 25.7, 23.1 (2C), 22.1, 18.5, 13.7; HRMS (ESI): calc. for $C_{13}H_{22}NaO$ $[M+Na]^+$: 217.1563; found: 217.1567.

(3-Methoxypent-1-yn-1-yl)benzene (**1q**)

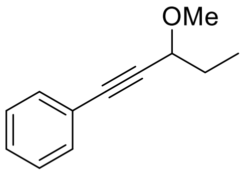

1q

Following procedure B using phenylacetylene (5.0 mmol) and *n*-propanal.
Purification over SiO_2 (eluent: ethyl acetate/pentane 5%).
620 mg (71% overall yield), colorless oil. ^{1}H NMR (500 MHz, $CDCl_3$) δ 7.45 (t, J = 4.1 Hz, 2H), 7.35 – 7.27 (m, 3H), 4.12 (t, J = 6.4 Hz, 1H), 3.48 (s, 3H), 1.90 – 1.75 (m, 2H), 1.07 (t, J = 7.4 Hz, 3H); ^{13}C NMR (126 MHz, $CDCl_3$) δ 131.9 (2C), 128.4 (3C), 123.0, 88.1, 86.0, 73.2, 56.6, 29.0, 9.8; HRMS (ESI): calc. for $C_{12}H_{14}NaO$ $[M+Na]^+$: 197.0937; found: 197.0938.

4-(3-Methoxyoct-1-yn-1-yl)phenyl pivalate (**1r**)

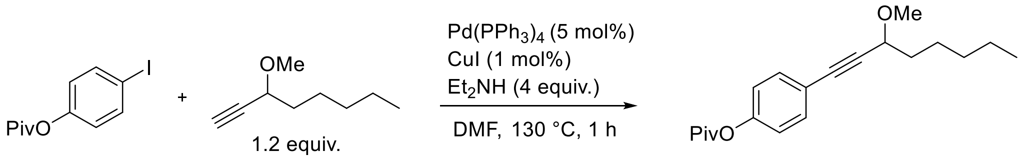

S10 **S11** **1r**, 84%

An oven-dried 35 mL Schlenk tube equipped with a magnetic stirring bar and a rubber septum was charged with $Pd(PPh_3)_4$ (5 mol%), CuI (1 mol%). After purging the vessel with alternating vacuum and nitrogen cycles, degassed DMF, aryl iodide **S10**[4] (608 mg, 2.0 mmol, 1 equiv.), Et_2NH (0.83 mL, 8 mmol, 4.0 equiv.) and 3-methoxyoct-1-yne (**S11**) (334 mg, 2.4 mmol, 1.2 equiv.) were added and the mixture was stirred at 130 °C for 1 h. After cooling to room temperature, the mixture was diluted with water and extracted with EtOAc. Combined organic extracts were washed with H_2O,

saturated aqueous NaCl, dried over $MgSO_4$ and concentrated in vacuum. The crude product was purified by silica gel chromatography affording product **1r** as a yellow oil (532 mg, 84 % isolated yield). ^{1}H NMR (400 MHz, $CDCl_3$) δ 7.45 (d, *J* = 8.7 Hz, 2H), 7.01 (d, *J* = 8.7 Hz, 2H), 4.15 (t, *J* = 6.5 Hz, 1H), 3.46 (s, 3H), 1.86 – 1.71 (m, 2H), 1.54-1.47 (m, 2H), 1.37-1.31 (m, 13 H), 0.99-0.88 (m, 3H); ^{13}C NMR (101 MHz, $CDCl_3$) δ 176.9, 151.1, 132.9 (2C), 121.7 (2C), 120.4, 88.3, 85.2, 71.9, 56.6, 39.2, 35.8, 31.7, 27.2 (2C), 25.1, 22.7, 14,2; HRMS (ESI): calc. for $C_{20}H_{28}NaO_3$ $[M+Na]^+$: 339.1931; found: 339.1943.

4-(3-Methoxy-3-methylbut-1-yn-1-yl)phenyl acetate (**1s**)

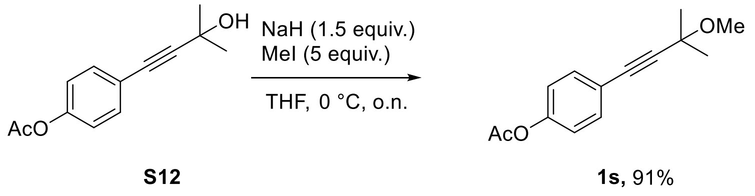

Compound **S12** was synthesized according to the reported procedure[5] from 4-iodophenol and 2-methylbut-3-yn-2-ol.

Following procedure B using alcohol **S12** (327 mg, 1.5 mmol), **1s** was obtained as a brown oil (318 mg, 91% isolated yield) after purification over SiO_2 (eluent: ethyl acetate/pentane 5%). ^{1}H NMR (400 MHz, $CDCl_3$) δ 7.97 (d, *J* = 8.1 Hz, 2H), 7.48 (d, *J* = 8.1 Hz, 2H), 3.91 (s, 3H), 3.43 (s, 3H), 1.54 (s, 6H); ^{13}C NMR (101 MHz, $CDCl_3$) δ 166.6, 131.7 (2C), 129.6, 129.6 (2C), 127.7, 94.2, 83.7, 71.1, 52.3, 51.9, 28.3 (2C); HRMS (ESI): calc. for $C_{14}H_{16}NaO_3$ $[M+Na]^+$: 255.0992; found: 255.0985.

Preparation of *2-(Hex-1-yn-1-yl)-2-(trifluoromethyl)tetrahydrofuran* (4a):

5-Bromo-1,1,1-trifluoropentan-2-one **S4** was prepared in accordance with the previously reported procedure.[6]

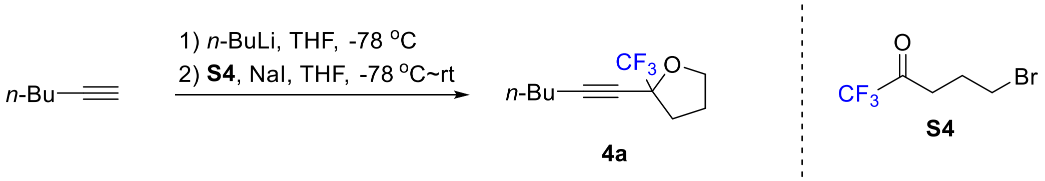

To a solution of 1-hexyne (1.49 mL, 13.0 mmol, 1.0 equiv) in THF (80 mL, 0.17 M) was added dropwise *n*-BuLi (5.72 mL, 2.5M in hexanes, 14.3 mmol, 1.1 equiv) at -78 °C under Ar atmosphere. The mixture was stirred for 1 h at -78 °C and the trifluoromethyl ketone **S4** (3.70 g, 16.9 mmol, 1.3 equiv) was added. The mixture was stirred for 1 h at -78 °C, and then NaI (780 mg, 5.2 mmol, 40 mol%) was added. The solution was warmed to room temperature and stirred overnight. The reaction mixture was quenched with sat. aq. NH_4Cl solution and extracted with diethyl ether. Combined organic phases were washed with brine, dried over Na_2SO_4 and concentrated in vacuo. Purification over SiO_2 (eluent: ethyl acetate/pentane 1 – 2%) afforded the product **4a** (1.60g, 56%) as a colorless oil.

^{1}H NMR (400 MHz, $CDCl_3$) δ 4.13 – 4.03 (m, 1H), 4.04 – 3.94 (m, 1H), 2.42 – 2.31 (m, 1H), 2.30 – 2.19 (m, 3H), 2.16 – 1.96 (m, 2H), 1.56 – 1.45 (m, 2H), 1.45 – 1.35 (m, 2H), 0.91 (t, *J* = 7.2 Hz, 3H); ^{13}C NMR (101 MHz, $CDCl_3$) δ 124.4 (q, *J* = 283.3 Hz), 87.7, 78.7 (q, *J* = 32.4 Hz), 75.9, 70.4, 35.6, 30.4, 26.0, 22.0, 18.5, 13.7;^{19}F NMR (377 MHz, $CDCl_3$) δ -80.10; HRMS (ESI): calc. for $C_{11}H_{15}F_3NaO$ $[M+Na]^+$: 243.0967; found: 243.0973.

2-(Hept-1-yn-1-yl)-2-methyltetrahydrofuran (**4b**)

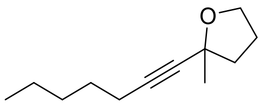

4b

Following procedure D using 1-heptyne (3.0 mmol) and 5-chloropentan-2-one.

Purification over SiO_2 (eluent: ethyl acetate/pentane 5%).

254 mg (47% overall yield), colorless oil. ^{1}H NMR (400 MHz, $CDCl_3$) δ 3.99 – 3.85 (m, 2H), 2.21 – 2.03 (m, 4H), 2.01 – 1.84 (m, 1H), 1.79 – 1.66 (m, 1H), 1.54 – 1.45 (m, 5H), 1.40 – 1.27 (m, 4H), 0.89 (t, *J* = 7.0 Hz, 3H); ^{13}C NMR (101 MHz, $CDCl_3$) δ 83.4, 83.4, 76.3, 67.5, 40.4, 31.2, 28.6, 28.1, 25.8, 22.3, 18.8, 14.1; HRMS (ESI): calc. for $C_{12}H_{20}NaO$ $[M+Na]^+$: 203.1406; found: 203.1398;.

2-(Cyclopropylethynyl)-2-methyltetrahydrofuran (**4c**)

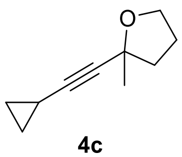

4c

Following procedure D using cyclopropylacetylene (3.0 mmol) and 5-chloropentan-2-one.

Purification over SiO_2 (eluent: ethyl acetate/pentane 5%).

203 mg (45% overall yield), colorless oil. ^{1}H NMR (500 MHz, $CDCl_3$) δ 3.95 – 3.84 (m, 2H), 2.16 – 1.99 (m, 2H), 1.96 – 1.87 (m, 1H), 1.78 – 1.69 (m, 1H), 1.49 (s, 3H), 1.26 – 1.19 (m, 1H), 0.76 – 0.71 (m, 2H), 0.67 – 0.61 (m, 2H); ^{13}C NMR (126 MHz, $CDCl_3$) δ 86.3, 78.6, 76.2, 67.5, 40.3, 28.1, 25.8, 8.4 (2C), -0.4; HRMS (ESI): calc. for $C_{10}H_{14}NaO$ $[M+Na]^+$: 173.0937; found: 173.0942.

tert-Butyldimethyl((3-(2-methyltetrahydrofuran-2-yl)prop-2-yn-1-yl)oxy)silane (**4d**)

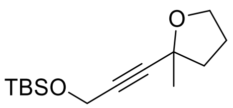

4d

Following procedure D using *tert*-butyldimethyl(prop-2-yn-1-yloxy)silane (3.0 mmol) and 5-chloropentan-2-one.

Purification over SiO_2 (eluent: ethyl acetate/pentane 5%).

395 mg (52% overall yield), colorless oil. ^{1}H NMR (400 MHz, $CDCl_3$) δ 4.33 (s, 2H), 3.98 – 3.85 (m, 2H), 2.18 (ddd, J = 11.9, 8.2, 4.2 Hz, 1H), 2.12 – 2.01 (m, 1H), 1.98 – 1.88 (m, 1H), 1.76 (ddd, J = 11.7, 8.9, 8.0 Hz, 1H), 1.53 (s, 3H), 0.91 (s, 9H), 0.12 (s, 6H); ^{13}C NMR (101 MHz, $CDCl_3$) δ 88.0, 81.5, 76.1, 67.7, 51.9, 40.0, 27.7, 25.9 (3C), 25.8, 18.4, -4.9 (2C); HRMS (ESI): calc. for $C_{14}H_{26}NaO_2Si$ $[M+Na]^+$: 277.1594; found: 277.1589.

2-(4-Phenylbut-1-yn-1-yl)tetrahydrofuran (**4f**)

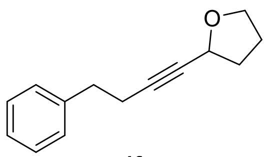

4f

Following procedure D using 4-phenyl-1-butyne (4.0 mmol) and 4-chlorobutanal.

Purification over SiO_2 (eluent: ethyl acetate/pentane 5%).

369 mg (46% overall yield), colorless oil. ^{1}H NMR (400 MHz, $CDCl_3$) δ 7.32 – 7.25 (m, 2H), 7.25 – 7.17 (m, 3H), 4.56 (ddt, J = 7.2, 5.4, 1.9 Hz, 1H), 3.97 – 3.87 (m, 1H), 3.84 – 3.73 (m, 1H), 2.82 (t, J = 7.6 Hz, 2H), 2.50 (td, J = 7.6, 1.9 Hz, 2H), 2.15 – 1.80 (m, 4H); ^{13}C NMR (126 MHz, $CDCl_3$) δ 140.8, 128.6 (2C), 128.5 (2C), 126.4, 84.5, 80.9, 68.5, 67.8, 35.2, 33.6, 25.5, 21.1; HRMS (ESI): calc. for $C_{14}H_{16}NaO$ $[M+Na]^+$: 223.1093; found: 223.1105.

2-(Hex-1-yn-1-yl)-2-phenyltetrahydrofuran (**4g**)

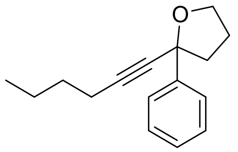

4g

Following procedure D using 1-hexyne (5.0 mmol) and 4-chloro-1-phenylbutan-1-one. Purification over SiO_2 (eluent: ethyl acetate/pentane 10%).

515 mg (45% overall yield), colorless oil. ^{1}H NMR (400 MHz, $CDCl_3$) δ 7.61 – 7.55 (m, 2H), 7.38 – 7.30 (m, 2H), 7.29 – 7.23 (m, 1H), 4.14 (dtd, *J* = 24.1, 8.0, 5.9 Hz, 2H), 2.45 (ddd, *J* = 11.6, 7.6, 4.4 Hz, 1H), 2.30 – 2.14 (m, 3H), 2.14 – 1.93 (m, 2H), 1.57 – 1.48 (m, 2H), 1.47 – 1.36 (m, 2H), 0.92 (t, *J* = 7.2 Hz, 3H); ^{13}C NMR (101 MHz, $CDCl_3$) δ 144.4, 128.2 (2C), 127.4, 125.4 (2C), 85.8, 82.3, 80.8, 68.3, 43.2, 30.9, 25.9, 22.1, 18.7, 13.7; HRMS (ESI): calc. for $C_{16}H_{20}NaO$ $[M+Na]^+$: 251.1406; found: 251.1414.

2-(4-Fluorophenyl)-2-(hex-1-yn-1-yl)tetrahydrofuran (**4h**)

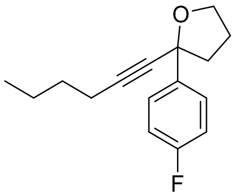

4h

Following procedure D using 1-hexyne (5.0 mmol) and 4-chloro-1-(4-fluorophenyl)butan-1-one. Purification over SiO_2 (eluent: ethyl acetate/pentane 10%).

628 mg (51% overall yield), colorless oil. ^{1}H NMR (400 MHz, $CDCl_3$) δ 7.58 – 7.49 (m, 2H), 7.06 – 6.95 (m, 2H), 4.20 – 4.04 (m, 2H), 2.48 – 2.34 (m, 1H), 2.30 – 2.13 (m, 3H), 2.10 – 1.93 (m, 2H), 1.61 – 1.32 (m, 4H), 0.91 (t, *J* = 7.2 Hz, 3H); ^{13}C NMR (101 MHz, $CDCl_3$) δ 162.2 (d, *J* = 245.3 Hz), 140.2 (d, *J* = 3.0 Hz), 127.2 (d, *J* = 8.1 Hz, 2C), 114.9 (d, *J* = 21.4 Hz, 2C), 86.1, 82.1, 80.4, 68.3, 43.3, 30.9, 25.9, 22.1, 18.6, 13.7; ^{19}F NMR (377 MHz, $CDCl_3$) δ -115.9; HRMS (ESI): calc. for $C_{16}H_{19}FNaO$ $[M+Na]^+$: 269.1312; found: 269.1324.

2-Ethynyl-2-phenyltetrahydrofuran (**4i**)

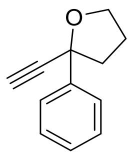

4i

Following procedure D using ethynylmagnesium bromide (4.5 mmol) and 4-chloro-1-phenylbutan-1-one.

Purification over SiO_2 (eluent: ethyl acetate/pentane 5%).

380 mg (49% overall yield), colorless oil. ^{1}H NMR (400 MHz, $CDCl_3$) δ 7.6 – 7.5 (m, 2H), 7.4 – 7.3 (m, 2H), 7.3 – 7.3 (m, 1H), 4.2 – 4.1 (m, 2H), 2.6 (s, 1H), 2.6 – 2.5 (m, 1H), 2.3 – 2.2 (m, 1H), 2.2 – 2.0 (m, 2H); ^{13}C NMR (101 MHz, $CDCl_3$) δ 143.3, 128.4 (2C), 127.7, 125.3 (2C), 86.1, 80.4, 73.2, 68.6, 42.9, 25.9; HRMS (ESI): calc. for $C_{12}H_{12}NaO$ $[M+Na]^+$: 195.0780; found: 195.0785.

2-Ethyl-2-(hept-1-yn-1-yl)oxetane (**6a**)

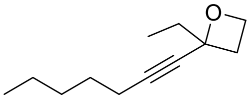

6a

Following procedure D using 1-heptyne (3.0 mmol) and 1-chloropentan-3-one.

Purification over SiO_2 (eluent: ethyl acetate/pentane 5%).

362 mg (67% overall yield), colorless oil. ^{1}H NMR (400 MHz, $CDCl_3$) δ 4.6 (ddd, J = 8.8, 7.0, 5.9 Hz, 1H), 4.4 (ddd, J = 8.8, 6.5, 5.9 Hz, 1H), 2.8 (ddd, J = 10.9, 8.8, 6.5 Hz, 1H), 2.6 (ddd, J = 10.9, 8.8, 7.0 Hz, 1H), 2.3 (t, J = 7.1 Hz, 2H), 1.9 (ttd, J = 13.3, 7.4, 5.9 Hz, 2H), 1.6 – 1.5 (m, 2H), 1.4 – 1.3 (m, 4H), 1.0 (t, J = 7.4 Hz, 3H), 0.9 (t, J = 7.2 Hz, 3H); ^{13}C NMR (101 MHz, $CDCl_3$) δ 88.4, 81.9, 81.6, 65.5, 35.5, 34.1, 31.2, 28.5, 22.3, 19.0, 14.1, 8.0; HRMS (ESI): calc. for $C_{12}H_{20}NaO$ $[M+Na]^+$: 203.1406; found: 203.1412.

tert-Butyl((3-(2-ethyloxetan-2-yl)prop-2-yn-1-yl)oxy)dimethylsilane (**6b**)

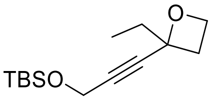

6b

Following procedure D using *tert*-butyldimethyl(prop-2-yn-1-yloxy)silane (3.0 mmol) and 1-chloropentan-3-one.

Purification over SiO_2 (eluent: ethyl acetate/pentane 5%).

465 mg (61% overall yield), colorless oil. ^{1}H NMR (500 MHz, CDCl$_3$) δ 4.63 (ddd, *J* = 8.8, 7.2, 5.9 Hz, 1H), 4.47 – 4.38 (m, 3H), 2.79 (ddd, *J* = 10.9, 8.8, 6.3 Hz, 1H), 2.62 (ddd, *J* = 10.9, 8.8, 7.2 Hz, 1H), 1.99 – 1.81 (m, 2H), 1.00 (t, *J* = 7.4 Hz, 3H), 0.91 (s, 9H), 0.13 – 0.12 (m, 6H); ^{13}C NMR (126 MHz, CDCl$_3$) δ 86.3, 86.1, 81.3, 65.7, 52.0, 35.1, 33.7, 25.9 (3C), 18.4, 7.9, -5.0 (2C); HRMS (ESI): calc. for $C_{14}H_{26}NaO_2Si$ [M+Na]$^+$: 277.1594; found: 277.1585.

tert-Butyl((4-(2-ethyloxetan-2-yl)but-3-yn-1-yl)oxy)dimethylsilane (**6c**)

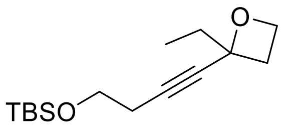

6c

Following procedure D using (but-3-yn-1-yloxy)(*tert*-butyl)dimethylsilane (3.0 mmol) and 1-chloropentan-3-one.

Purification over SiO_2 (eluent: ethyl acetate/pentane 5%).

440 mg (55% overall yield), colorless oil. ^{1}H NMR (400 MHz, CDCl$_3$) δ 4.6 (ddd, *J* = 8.9, 7.1, 5.9 Hz, 1H), 4.4 (ddd, *J* = 8.8, 6.5, 5.9 Hz, 1H), 3.7 (t, *J* = 7.2 Hz, 2H), 2.8 (ddd, *J* = 10.9, 8.8, 6.5 Hz, 1H), 2.6 (ddd, *J* = 10.9, 8.8, 7.1 Hz, 1H), 2.5 (t, *J* = 7.1 Hz, 2H), 2.0 – 1.8 (m, 2H), 1.0 (t, *J* = 7.4 Hz, 3H), 0.9 (s, 9H), 0.1 (s, 6H); ^{13}C NMR (101 MHz, CDCl$_3$) δ 85.1, 82.9, 81.5, 65.5, 62.0, 35.4, 34.0, 26.0 (3C), 23.4, 18.4, 8.0, -5.2 (2C); HRMS (ESI): calc. for $C_{15}H_{28}NaO_2Si$ [M+Na]$^+$: 291.1751; found: 291.1760

2-Ethyl-2-(4-phenylbut-1-yn-1-yl)oxetane (**6d**)

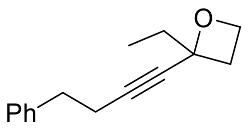

6d

Following procedure D using 4-phenyl-1-butyne (4.0 mmol) and 1-chloropentan-3-one.

Purification over SiO_2 (eluent: ethyl acetate/pentane 5%).

582 mg (68% overall yield), colorless oil. ^{1}H NMR (500 MHz, CDCl$_3$) δ 7.3 – 7.3 (m, 2H), 7.2 – 7.2 (m, 3H), 4.6 (ddd, *J* = 8.8, 7.1, 5.9 Hz, 1H), 4.4 (dt, *J* = 8.8, 6.2 Hz, 1H), 2.9 (t, *J* = 7.6 Hz, 2H), 2.7 (ddd, *J* = 10.9, 8.8, 6.5 Hz, 1H), 2.6 – 2.5 (m, 3H), 2.0 – 1.8 (m, 2H), 0.9 (t, *J* = 7.4 Hz, 3H); ^{13}C NMR (126 MHz, CDCl$_3$) δ 140.8, 128.6 (2C), 128.5 (2C), 126.4, 87.4, 82.7, 81.6, 65.6,

35.3, 35.2, 34.0, 21.3, 8.0; HRMS (ESI): calc. for $C_{15}H_{18}NaO$ $[M+Na]^+$: 237.1250; found: 237.1242.

2-Ethynyl-2-phenyloxetane (**6e**)

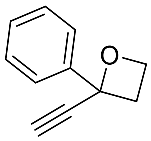

6e

Following procedure D using ethynylmagnesium bromide (5.0 mmol) and 1-chloropentan-3-one. Purification over SiO_2 (eluent: ethyl acetate/pentane 5%).

301 mg (38% overall yield), colorless oil. ^1H NMR (400 MHz, $CDCl_3$) δ 7.7 – 7.6 (m, 2H), 7.5 – 7.4 (m, 2H), 7.4 – 7.3 (m, 1H), 4.9 (ddd, J = 8.5, 7.4, 5.9 Hz, 1H), 4.6 (dt, J = 8.9, 6.0 Hz, 1H), 3.2 (ddd, J = 11.0, 8.5, 6.2 Hz, 1H), 3.0 – 2.9 (m, 2H); ^{13}C NMR (101 MHz, $CDCl_3$) δ 143.4, 128.6 (2C), 128.2, 124.7 (2C), 85.4, 80.5, 76.8, 66.2, 38.4; HRMS (ESI): calc. for $C_{11}H_{10}NaO$ $[M+Na]^+$: 181.0624; found: 181.0627

1. Iron-catalyzed synthesis of allenes

General procedure for the preparation of allenes 2, 5 and 7:

In a dry 5 mL microwave flask under argon were added catalyst $Fe(acac)_3$ (5 – 7.5 mol%) and anhydrous toluene (0.2 M) to obtain an orange solution. The reaction solution was cooled to 0 °C and propargyl ether (1.00 equiv) was added followed by the dropwise addition of the corresponding Grignard reagent (1.5 – 2.5 equiv.). The reaction mixture was allowed to reach the room temperature and was stirred for another 30 min, quenched with aq. 5% citric acid solution, extracted with pentane or diethyl ether (3 x 5 mL) and the combined organic phase was carefully evaporated in vacuo. The crude products were purified by flash column chromatography over SiO_2 (eluent: ethyl acetate/pentane) to obtain the desired products.

Preparation of allenes 2:

(6,6,6-Trifluoro-3,5-dimethylhexa-3,4-dien-1-yl)benzene (**2aa**)

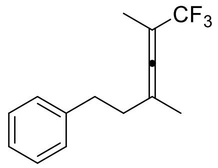

2aa

According to the general procedure: $Fe(acac)_3$ (5.3 mg, 0.015 mmol, 5 mol%), substrate **1af** (77 mg, 0.3 mmol, 1.0 equiv.) and methylmagnesium bromide (3.0 M in diethyl ether, 150 µL, 0.45 mmol, 1.5 equiv.) in anhydrous toluene (1.5 mL) were reacted. The reaction mixture was extracted and purified over SiO_2 (ethyl acetate/pentane 1 – 2%) to yield product **2aa** (64 mg, 89%) as a colorless oil.

^{1}H NMR (500 MHz, $CDCl_3$) δ 7.33 – 7.27 (m, 2H), 7.23 – 7.17 (m, 3H), 2.74 (h, J = 6.4 Hz, 2H), 2.36 (t, J = 7.8 Hz, 2H), 1.79 (s, 3H), 1.70 (s, 3H); ^{13}C NMR (126 MHz, $CDCl_3$) δ 200.2 (q, J = 4.2 Hz), 141.4, 128.5 (2C), 128.5 (2C), 126.1, 124.2 (q, J = 273.0 Hz), 106.0, 93.3 (q, J = 34.6 Hz), 35.2, 33.6, 18.7, 13.2; ^{19}F NMR (377 MHz, $CDCl_3$) δ -65.74; HRMS (ESI): calc. for $C_{14}H_{15}F_3Na$ $[M+Na]^+$: 263.1018; found: despite repeated attempts, we were not able to obtain high-resolution mass data for **2aa**.

(6,6-Difluoro-5-methylhex-5-en-3-yn-1-yl)benzene (**3**)

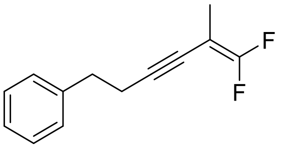

3

According to the general procedure: $Fe(acac)_3$ (5.3 mg, 0.015 mmol, 5 mol%), substrate **1aa** (85 mg, 0.3 mmol, 1.0 equiv.) and methylmagnesium bromide (3.0 M in diethyl ether, 150 µL, 0.45 mmol, 1.5 equiv.) in anhydrous toluene (1.5 mL) were reacted. The reaction mixture was extracted and purified over SiO_2 (ethyl acetate/pentane 1 – 2%) to yield product **3** (37 mg, 60%) as a pale yellow oil.

^{1}H NMR (400 MHz, $CDCl_3$) δ 7.37 – 7.30 (m, 2H), 7.28 – 7.21 (m, 3H), 2.88 (t, J = 7.5 Hz, 2H), 2.63 (tt, J = 7.5, 1.4 Hz, 2H), 1.71 (t, J = 3.4 Hz, 3H); ^{13}C NMR (101 MHz, $CDCl_3$) δ 158.9 (dd, J = 292.2, 290.8 Hz), 140.7, 128.6 (2C), 128.5 (2C), 126.5, 93.0 (dd, J = 6.4, 5.2 Hz), 74.1 (dd, J = 8.1, 4.0 Hz), 73.6 (dd, J = 35.3, 18.0 Hz), 35.2, 21.8, 13.4. ^{19}F NMR (377 MHz, $CDCl_3$) δ -65.72, -78.56, -82.81 – -82.95 (m), -87.54 – -87.66 (m); HRMS (ESI): calc. for $C_{13}H_{12}F_2Na$ $[M+Na]^+$:

229.0799; found: despite repeated attempts, we were not able to obtain high-resolution mass data for **3**.

(3-(3,3,3-Trifluoro-2-methylprop-1-en-1-ylidene)heptyl)benzene (**2ab**)

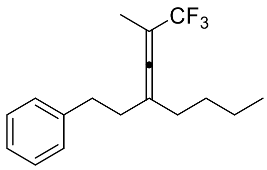

2ab

According to the general procedure: $Fe(acac)_3$ (5.3 mg, 0.015 mmol, 5 mol%), TMEDA (4.5 µL, 0.03 mmol, 10 mol%) substrate **1af** (77 mg, 0.3 mmol, 1.0 equiv.) and butylmagnesium chloride (2.0 M in diethyl ether, 225 µL, 0.45 mmol, 1.5 equiv.) in anhydrous toluene (1.5 mL) were reacted. The reaction mixture was extracted and purified over SiO_2 (ethyl acetate/pentane 1 – 2%) to yield product **2ab** (36 mg, 43%) as a colorless oil.

^{1}H NMR (400 MHz, $CDCl_3$) δ 7.33 – 7.25 (m, 2H), 7.19 (ddq, J = 5.6, 3.6, 2.5, 1.8 Hz, 3H), 2.80 – 2.66 (m, 2H), 2.34 (dd, J = 8.2, 7.2 Hz, 2H), 2.04 (t, J = 7.3 Hz, 2H), 1.71 (s, 3H), 1.47 – 1.25 (m, 4H), 0.90 (t, J = 7.1 Hz, 3H); ^{13}C NMR (101 MHz, $CDCl_3$) δ 199.9 (d, J = 4.3 Hz), 141.6, 128.5 (2C), 128.5 (2C), 126.1, 125.7 (q, J = 273.1 Hz), 111.0, 94.8 (q, J = 34.7 Hz), 33.9, 33.7, 32.3, 29.5, 22.3, 14.00, 13.3; ^{19}F NMR (377 MHz, $CDCl_3$) δ -65.61; HRMS (ESI): calc. for $C_{17}H_{21}F_3Na$ $[M+Na]^+$: 305.1488; found: despite repeated attempts, we were not able to obtain high-resolution mass data for **2ab**.

(6,6,6-Trifluoro-5-methylhexa-3,4-diene-1,3-diyl)dibenzene (**2ac**)

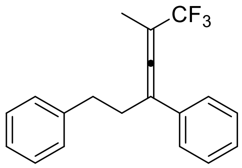

2ac

According to the general procedure: $Fe(acac)_3$ (5.3 mg, 0.015 mmol, 5 mol%), substrate **1af** (77 mg, 0.3 mmol, 1.0 equiv.) and phenylmagnesium bromide (3.0 M in diethyl ether, 200 µL, 0.6 mmol, 2.0 equiv.) in anhydrous toluene (1.5 mL) were reacted. The reaction mixture was extracted and purified over SiO_2 (ethyl acetate/pentane 1 – 2%) to yield product **2ac** (56 mg, 62%) as a colorless oil.

^{1}H NMR (400 MHz, $CDCl_3$) δ 7.43 – 7.28 (m, 7H), 7.27 – 7.19 (m, 3H), 2.95 – 2.78 (m, 4H), 1.83 (s, 3H); ^{13}C NMR (101 MHz, $CDCl_3$) δ 203.0 (d, J = 4.1 Hz), 141.3, 134.8, 128.8 (2C), 128.6 (2C), 128.5 (2C), 128.1, 126.5 (2C), 126.2, 123.9 (q, J = 273.9 Hz), 111.7, 97.4 (q, J = 34.9 Hz), 33.9, 31.7, 13.1; ^{19}F NMR (377 MHz, $CDCl_3$) δ -65.30; HRMS (APCI): calc. for $C_{19}H_{17}F_3H$ $[M+H]^+$: 303.1355; found: 303.1365.

tert-Butyldimethyl((5,5,5-trifluoro-2-methyl-4-phenylpenta-2,3-dien-1-yl)oxy)silane (**2b**)

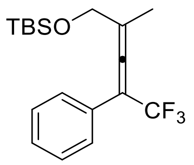

2b

According to the general procedure: $Fe(acac)_3$ (5.3 mg, 0.015 mmol, 5 mol%), substrate **1b** (108 mg, 0.3 mmol, 1.0 equiv.) and methylmagnesium bromide (3.0 M in diethyl ether, 150 µL, 0.45 mmol, 1.5 equiv.) in anhydrous toluene (1.5 mL) were reacted. The reaction mixture was extracted and purified over SiO_2 (ethyl acetate/pentane 1 – 5%) to yield product **2b** (76 mg, 74%) as a colorless oil.

^{1}H NMR (500 MHz, $CDCl_3$) δ 7.47 – 7.41 (m, 2H), 7.38 – 7.32 (m, 2H), 7.31 – 7.27 (m, 1H), 4.25 (d, J = 1.6 Hz, 2H), 1.88 (s, 3H), 0.88 (s, 9H), 0.05 (s, 6H); ^{13}C NMR (126 MHz, $CDCl_3$) δ 201.7 – 201.5 (m), 131.0, 128.7 (2C), 128.0, 127.4 (2C), 123.7 (q, J = 274.0 Hz), 109.9, 102.5 (q, J = 34.3 Hz), 63.8, 25.9 (3C), 18.4, 14.8, -5.4 (2C); ^{19}F NMR (377 MHz, $CDCl_3$) δ -60.31; HRMS (ESI): calc. for $C_{18}H_{25}F_3NaOSi$ $[M+Na]^+$: 365.1519; found: 365.1520.

2-(4-Phenyl-2-(trifluoromethyl)penta-2,3-dien-1-yl)-1,3-dioxolane (**2c**)

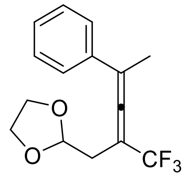

2c

According to the general procedure: $Fe(acac)_3$ (5.3 mg, 0.015 mmol, 5 mol%), substrate **1c** (90 mg, 0.3 mmol, 1.0 equiv.) and methylmagnesium bromide (3.0 M in diethyl ether, 150 µL, 0.45 mmol, 1.5 equiv.) in anhydrous toluene (1.5 mL) were reacted. The reaction mixture was extracted and purified over SiO_2 (ethyl acetate/pentane 1 – 5%) to yield product **2c** (79 mg, 92%) as a colorless

oil.

^{1}H NMR (400 MHz, $CDCl_3$) δ 7.47 – 7.41 (m, 2H), 7.36 (ddd, *J* = 7.8, 6.8, 1.2 Hz, 2H), 7.32 – 7.25 (m, 1H), 5.07 (t, *J* = 4.8 Hz, 1H), 4.00 – 3.81 (m, 4H), 2.66 – 2.53 (m, 2H), 2.19 (s, 3H); ^{13}C NMR (101 MHz, $CDCl_3$) δ 204.7 (d, *J* = 4.1 Hz), 134.8, 128.7 (2C), 128.1, 126.4 (2C), 123.6 (q, *J* = 273.9 Hz), 108.4, 102.7, 95.3 (q, *J* = 35.0 Hz), 65.1, 65.1, 32.2, 16.6; ^{19}F NMR (377 MHz, $CDCl_3$) δ -63.76; HRMS (ESI): calc. for $C_{15}H_{15}F_3NaO_2$ $[M+Na]^+$: 307.0916; found: 307.0915.

(1,1,1,2,2-Pentafluoro-5-methylhexa-3,4-dien-3-yl)benzene (**2d**)

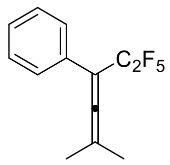

2d

According to the general procedure: $Fe(acac)_3$ (5.3 mg, 0.015 mmol, 5 mol%), substrate **1d** (84 mg, 0.3 mmol, 1.0 equiv.) and methylmagnesium bromide (3.0 M in diethyl ether, 150 µL, 0.45 mmol, 1.5 equiv.) in anhydrous toluene (1.5 mL) were reacted. The reaction mixture was extracted and purified over SiO_2 (ethyl acetate/pentane 1 – 5%) to yield product **2d** (57 mg, 72%) as a colorless oil.

^{1}H NMR (400 MHz, $CDCl_3$) δ 7.45 – 7.27 (m, 5H), 1.88 (s, 6H); ^{13}C NMR (101 MHz, $CDCl_3$) δ 204.6 (t, *J* = 7.6 Hz), 131.8, 128.6 (2C), 128.3, 128.0 (2C), 124.1 – 110.6 (m, 2C), 104.3, 97.0 (t, *J* = 26.7 Hz), 19.6 (t, *J* = 1.9 Hz, 2C); ^{19}F NMR (377 MHz, $CDCl_3$) δ -83.04, -108.04; HRMS (APCI): calc. for $C_{13}H_{11}F_5H$ $[M+H]^+$: 263.0854; found: 263.0853.

tert-Butyl((4-cyclohexyl-5,5,5-trifluoro-2-methylpenta-2,3-dien-1-yl)oxy)dimethylsilane (**2e**)

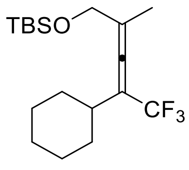

2e

According to the general procedure: $Fe(acac)_3$ (5.3 mg, 0.015 mmol, 5 mol%), substrate **1e** (109 mg, 0.3 mmol, 1.0 equiv.) and methylmagnesium bromide (3.0 M in diethyl ether, 150 µL, 0.45 mmol, 1.5 equiv.) in anhydrous toluene (1.5 mL) were reacted. The reaction mixture was extracted and purified over SiO_2 (ethyl acetate/pentane 1 – 5%) to yield product **2e** (89 mg, 85%) as a

colorless oil.

^{1}H NMR (400 MHz, $CDCl_3$) δ 4.12 (s, 2H), 2.05 (tt, *J* = 11.4, 3.5 Hz, 1H), 1.94 – 1.80 (m, 2H), 1.79 – 1.60 (m, 6H), 1.37 – 1.04 (m, 5H), 0.89 (s, 9H), 0.06 (s, 6H); ^{13}C NMR (101 MHz, $CDCl_3$) δ 199.2 (d, *J* = 4.7 Hz), 124.4 (q, *J* = 274.0 Hz), 108.8, 105.0 (q, *J* = 32.1 Hz), 64.1 (d, *J* = 1.7 Hz), 36.2, 33.2, 33.0, 26.4, 26.0, 25.9 (3C), 18.4, 15.2, 15.2, -5.3, -5.4; ^{19}F NMR (377 MHz, $CDCl_3$) δ -62.33; HRMS (ESI): calc. for $C_{18}H_{31}F_3NaOSi$ $[M+Na]^+$: 371.1988; found: 371.1986.

tert-Butyl((4-cyclohexyl-5,5-difluoro-2-methylpenta-2,3-dien-1-yl)oxy)dimethylsilane (**2f**)

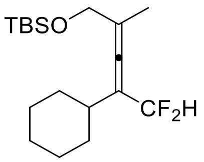

2f

According to the general procedure: $Fe(acac)_3$ (5.3 mg, 0.015 mmol, 5 mol%), substrate **1f** (104 mg, 0.3 mmol, 1.0 equiv.) and methylmagnesium bromide (3.0 M in diethyl ether, 150 μL, 0.45 mmol, 1.5 equiv.) in anhydrous toluene (1.5 mL) were reacted. The reaction mixture was extracted and purified over SiO_2 (ethyl acetate/pentane 1 – 5%) to yield product **2f** (76 mg, 77%) as a colorless oil.

^{1}H NMR (400 MHz, $CDCl_3$) δ 6.08 (t, *J* = 56.8 Hz, 1H), 4.11 (s, 2H), 2.11 (tt, *J* = 11.5, 3.5 Hz, 1H), 1.91 – 1.79 (m, 2H), 1.77 – 1.60 (m, 6H), 1.37 – 1.23 (m, 2H), 1.21 – 1.08 (m, 3H), 0.90 (s, 9H), 0.06 (s, 6H); ^{13}C NMR (101 MHz, $CDCl_3$) δ 200.5 (t, *J* = 10.3 Hz), 116.3 (t, *J* = 241.0 Hz), 107.3 (t, *J* = 24.1 Hz), 106.4, 64.5 (t, *J* = 3.1 Hz), 35.1, 33.5, 33.3, 26.5 (2C), 26.2, 26.0 (3C), 18.5, 15.5 (t, *J* = 2.6 Hz), -5.2 (2C); ^{19}F NMR (377 MHz, $CDCl_3$) δ -110.96, -110.97; HRMS (ESI): calc. for $C_{18}H_{32}F_2NaOSi$ $[M+Na]^+$: 353.2083; found: 353.2092.

(4-(Trifluoromethyl)hepta-2,3,6-trien-2-yl)cyclohexane (**2g**)

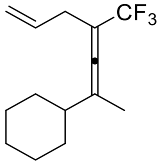

2g

According to the general procedure: $Fe(acac)_3$ (5.3 mg, 0.015 mmol, 5 mol%), substrate **1g** (78 mg, 0.3 mmol, 1.0 equiv.) and methylmagnesium bromide (3.0 M in diethyl ether, 150 μL, 0.45 mmol,

1.5 equiv.) in anhydrous toluene (1.5 mL) were reacted. The reaction mixture was extracted and purified over SiO_2 (ethyl acetate/pentane 1 – 5%) to yield product **2g** (65 mg, 88%) as a colorless oil.

^{1}H NMR (400 MHz, $CDCl_3$) δ 5.76 (ddt, *J* = 16.9, 10.0, 6.8 Hz, 1H), 5.15 – 5.03 (m, 2H), 2.86 (d, *J* = 6.8 Hz, 2H), 1.88 – 1.71 (m, 8H), 1.66 (dddd, *J* = 11.9, 4.0, 2.8, 1.5 Hz, 1H), 1.35 – 0.99 (m, 5H); ^{13}C NMR (101 MHz, $CDCl_3$) δ 200.1 (q, *J* = 4.4 Hz), 134.5, 124.2 (q, *J* = 273.5 Hz), 116.8, 113.5, 97.2 (q, *J* = 33.6 Hz), 41.9, 31.9, 31.7, 31.6, 26.4 (2C), 26.3, 17.0 (d, *J* = 1.7 Hz); ^{19}F NMR (377 MHz, $CDCl_3$) δ -64.11; HRMS (APCI): calc. for $C_{14}H_{19}F_3H$ $[M+H]^+$: 245.1512; found: 245.1513.

(4-(Trifluoromethyl)hepta-2,3,6-trien-2-yl)benzene (**2h**)

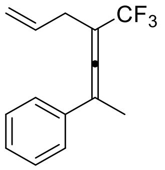

2h

According to the general procedure: $Fe(acac)_3$ (5.3 mg, 0.015 mmol, 5 mol%), substrate **1h** (76 mg, 0.3 mmol, 1.0 equiv.) and methylmagnesium bromide (3.0 M in diethyl ether, 150 µL, 0.45 mmol, 1.5 equiv.) in anhydrous toluene (1.5 mL) were reacted. The reaction mixture was extracted and purified over SiO_2 (ethyl acetate/pentane 1 – 5%) to yield product **2h** (54 mg, 75%) as a colorless oil.

^{1}H NMR (400 MHz, $CDCl_3$) δ 7.41 – 7.33 (m, 4H), 7.31 – 7.25 (m, 1H), 5.82 (ddt, *J* = 16.9, 10.1, 6.8 Hz, 1H), 5.17 (dq, *J* = 17.1, 1.6 Hz, 1H), 5.09 (dq, *J* = 10.1, 1.4 Hz, 1H), 3.03 (dq, *J* = 6.8, 1.6 Hz, 2H), 2.18 (s, 3H); ^{13}C NMR (126 MHz, $CDCl_3$) δ 203.5 (q, *J* = 4.2 Hz), 135.1, 133.7, 128.7 (2C), 128.1, 126.2 (2C), 123.7 (q, *J* = 274.3 Hz), 117.5, 108.8, 99.4 (q, *J* = 33.7 Hz), 31.7, 16.8; ^{19}F NMR (377 MHz, $CDCl_3$) δ -63.76; HRMS (ESI): calc. for $C_{14}H_{13}F_3Na$ $[M+Na]^+$: 261.0862; found: despite repeated attempts, we were not able to obtain high-resolution mass data for **2h**.

(4-(Trifluoromethyl)nona-2,3,8-trien-2-yl)benzene (**2i**)

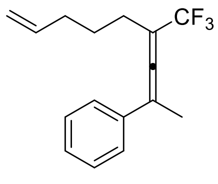

2i

According to the general procedure: $Fe(acac)_3$ (5.3 mg, 0.015 mmol, 5 mol%), substrate **1i** (85 mg, 0.3 mmol, 1.0 equiv.) and methylmagnesium bromide (3.0 M in diethyl ether, 150 µL, 0.45 mmol, 1.5 equiv.) in anhydrous toluene (1.5 mL) were reacted. The reaction mixture was extracted and purified over SiO_2 (ethyl acetate/pentane 1 – 2%) to yield product **2i** (62 mg, 78%) as a colorless oil.

^{1}H NMR (400 MHz, $CDCl_3$) δ 7.42 – 7.32 (m, 4H), 7.32 – 7.24 (m, 1H), 5.76 (ddt, J = 17.0, 10.2, 6.7 Hz, 1H), 5.04 – 4.92 (m, 2H), 2.26 (td, J = 7.4, 3.0 Hz, 2H), 2.18 (s, 3H), 2.10 (dt, J = 8.1, 6.6 Hz, 2H), 1.59 (p, J = 7.5 Hz, 2H); ^{13}C NMR (101 MHz, $CDCl_3$) δ 202.9 (q, J = 4.2 Hz), 138.1, 135.2, 128.7 (2C), 128.0, 126.2 (2C), 123.9 (q, J = 274.2 Hz), 115.3, 108.52, 100.6 (q, J = 33.5 Hz), 33.3, 26.8, 26.3, 16.8; ^{19}F NMR (377 MHz, $CDCl_3$) δ -63.86; HRMS (APCI): calc. for $C_{16}H_{17}F_3H$ $[M+H]^+$: 267.1355; found: 267.1356.

1-(4-Cyclopropyl-1,1,1-trifluoropenta-2,3-dien-2-yl)-3,5-difluorobenzene (**2j**)

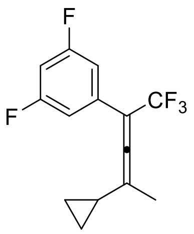

2j

According to the general procedure: $Fe(acac)_3$ (5.3 mg, 0.015 mmol, 5 mol%), substrate **1j** (87 mg, 0.3 mmol, 1.0 equiv.) and methylmagnesium bromide (3.0 M in diethyl ether, 150 µL, 0.45 mmol, 1.5 equiv.) in anhydrous toluene (1.5 mL) were reacted. The reaction mixture was extracted and purified over SiO_2 (ethyl acetate/pentane 1 – 5%) to yield product **2j** (63 mg, 76%) as a colorless oil.

^{1}H NMR (400 MHz, $CDCl_3$) δ 6.95 – 6.88 (m, 2H), 6.73 (tt, J = 8.8, 2.3 Hz, 1H), 1.94 (s, 3H), 1.40 – 1.32 (m, 1H), 0.87 – 0.77 (m, 2H), 0.57 – 0.47 (m, 2H); ^{13}C NMR (101 MHz, $CDCl_3$) δ 202.5 (q, J = 4.0 Hz), 164.4 (d, J = 13.0 Hz), 162.0 (d, J = 13.1 Hz), 134.6 (t, J = 9.8 Hz), 123.0 (q, J = 274.1 Hz), 114.4, 110.2 – 109.6 (m, 3C), 103.3 (t, J = 25.5 Hz), 17.4, 13.3, 7.3, 6.4; ^{19}F NMR (377 MHz,

$CDCl_3$) δ -60.60, -109.44, -109.46, -109.48; HRMS: calc. for $C_{14}H_{11}F_5Na$ $[M+Na]^+$: 274.2340; found: despite repeated attempts, we were not able to obtain high-resolution mass data for **2j**.

3-(1,1,1,2,2-Pentafluoro-5-phenylhexa-3,4-dien-3-yl)pyridine (**2k**)

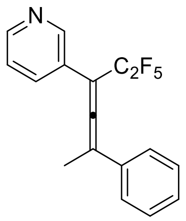

2k

According to the general procedure: $Fe(acac)_3$ (5.3 mg, 0.015 mmol, 5 mol%), substrate **1k** (84 mg, 0.3 mmol, 1.0 equiv.) and phenylmagnesium bromide (3.0 M in diethyl ether, 150 µL, 0.45 mmol, 1.5 equiv.) in anhydrous toluene (1.5 mL) were reacted. The reaction mixture was extracted and purified over SiO_2 (ethyl acetate/pentane 10 – 20%) to yield product **2k** (17 mg, 17%) as a colorless oil and recovered **1k** (36 mg, 43%).

^{1}H NMR (400 MHz, $CDCl_3$) δ 8.69 (d, J = 2.3 Hz, 1H), 8.56 (dd, J = 4.8, 1.6 Hz, 1H), 7.78 (ddt, J = 8.0, 2.3, 1.2 Hz, 1H), 7.46 – 7.27 (m, 6H), 2.31 (s, 3H); ^{13}C NMR (101 MHz, $CDCl_3$) δ 208.4 (t, J = 7.4 Hz), 149.5, 149.4, 135.3, 133.3, 129.0 (2C), 128.9, 127.4, 126.3 (2C), 123.5, 121.4 – 112.2 (m, 2C), 110.0, 98.5 (t, J = 27.5 Hz), 16.5; ^{19}F NMR (377 MHz, $CDCl_3$) δ -82.91 (t, J = 2.4 Hz), -107.52 (dd, J = 274.0, 2.4 Hz), -108.93 (dd, J = 274.4, 2.4 Hz); HRMS (ESI): calc. for $C_{17}H_{12}F_5NNa$ $[M+Na]^+$: 348.0782; found: 348.0793.

(3,5-Dimethylhexa-3,4-dien-1-yl)benzene (**2oa**)

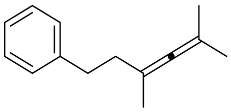

2oa

According to the general procedure: $Fe(acac)_3$ (5.3 mg, 0.015 mmol, 5 mol%), substrate **1o** (61 mg, 0.3 mmol, 1.0 equiv.) and methylmagnesium bromide (3.0 M in diethyl ether, 150 µL, 0.45 mmol, 1.5 equiv.) in anhydrous toluene (1.5 mL) were reacted. The reaction mixture was extracted and purified over SiO_2 (ethyl acetate/pentane 1 – 2%) to yield product **2oa** (49 mg, 87%) as a colorless oil.

^{1}H NMR (500 MHz, $CDCl_3$) δ 7.28 (t, J = 7.5 Hz, 2H), 7.24 – 7.12 (m, 3H), 2.71 (t, J = 7.8 Hz,

2H), 2.23 (t, J = 7.8 Hz, 2H), 1.68 (s, 3H), 1.60 (s, 6H); ^{13}C NMR (126 MHz, $CDCl_3$) δ 199.3, 142.7, 128.5 (2C), 128.3 (2C), 125.7, 96.6, 94.7, 36.1, 34.2, 21.0 (2C), 19.7; HRMS (ESI): calc. for $C_{14}H_{18}Na$ $[M+Na]^+$: 209.1301; found: 209.1307.

(3-Ethyl-5-methylhexa-3,4-dien-1-yl)benzene (**2ob**)

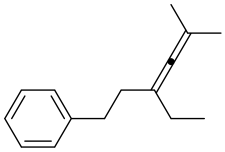

2ob

According to the general procedure: $Fe(acac)_3$ (5.3 mg, 0.015 mmol, 5 mol%), substrate **1o** (61 mg, 0.3 mmol, 1.0 equiv.) and ethylmagnesium bromide (3.0 M in diethyl ether, 150 µL, 0.45 mmol, 1.5 equiv.) in anhydrous toluene (1.5 mL) were reacted. The reaction mixture was extracted and purified over SiO_2 (ethyl acetate/pentane 1 – 2%) to yield product **2ob** (28 mg, 46%) as a colorless oil.

^{1}H NMR (400 MHz, $CDCl_3$) δ 7.31 – 7.24 (m, 2H), 7.22 – 7.12 (m, 3H), 2.76 – 2.66 (m, 2H), 2.28 – 2.20 (m, 2H), 1.99 – 1.87 (m, 2H), 1.63 (s, 6H), 0.97 (t, J = 7.4 Hz, 3H); ^{13}C NMR (101 MHz, $CDCl_3$) δ 198.5, 142.8, 128.5 (2C), 128.3 (2C), 125.7, 103.5, 96.9, 34.8, 34.3, 26.3, 21.1 (2C), 12.6; HRMS (ESI): calc. for $C_{15}H_{20}Na$ $[M+Na]^+$: 223.1457; found: 223.1455.

(1-Cyclohexylidenehex-1-en-2-yl)benzene (**2p**)

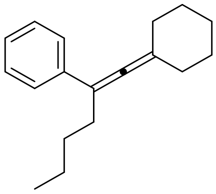

2p

According to the general procedure: $Fe(acac)_3$ (5.3 mg, 0.015 mmol, 5 mol%), substrate **1p** (58 mg, 0.3 mmol, 1.0 equiv.) and phenylmagnesium bromide (3.0 M in diethyl ether, 150 µL, 0.45 mmol, 1.5 equiv.) in anhydrous toluene (1.5 mL) were reacted. The reaction mixture was extracted and purified over SiO_2 (ethyl acetate/pentane 1 – 2%) to yield product **2p** (61 mg, 84%) as a colorless oil.

^{1}H NMR (400 MHz, $CDCl_3$) δ 7.43 – 7.38 (m, 2H), 7.33 – 7.27 (m, 2H), 7.19 – 7.13 (m, 1H), 2.43 – 2.37 (m, 2H), 2.22 (ddd, J = 6.9, 5.3, 2.1 Hz, 4H), 1.74 – 1.48 (m, 8H), 1.42 (dddd, J = 13.9, 9.4,

7.5, 6.1 Hz, 2H), 0.94 (t, *J* = 7.3 Hz, 3H); ^{13}C NMR (101 MHz, $CDCl_3$) δ 198.3, 138.8, 128.3 (2C), 126.1, 126.0 (2C), 105.8, 103.2, 31.7 (2C), 30.3, 29.9, 28.0 (2C), 26.4, 22.5, 14.2; HRMS (ESI): calc. for $C_{18}H_{24}Na$ [M+Na]$^+$: 263.1770; found: 263.1772.

Trimethyl(2-phenylhexa-2,3-dien-1-yl)silane (**2q**)

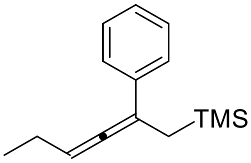

2q

According to the general procedure: $Fe(acac)_3$ (5.3 mg, 0.015 mmol, 5 mol%), substrate **1q** (52 mg, 0.3 mmol, 1.0 equiv.) and (trimethylsilyl)methylmagnesium chloride (1.0 M in diethyl ether, 450 μL, 0.45 mmol, 1.5 equiv.) in anhydrous toluene (1.5 mL) were reacted. The reaction mixture was extracted and purified over SiO_2 (ethyl acetate/pentane 1 – 2%) to yield product **2q** (64 mg, 92%) as a colorless oil.

^{1}H NMR (500 MHz, $CDCl_3$) δ 7.41 (d, *J* = 7.8 Hz, 2H), 7.34 – 7.23 (m, 2H), 7.17 (t, *J* = 7.3 Hz, 1H), 5.47 (tt, *J* = 6.3, 2.5 Hz, 1H), 2.13 (p, *J* = 7.2 Hz, 2H), 1.87 – 1.74 (m, 2H), 1.09 (t, *J* = 7.4 Hz, 3H), 0.02 (s, 9H); ^{13}C NMR (126 MHz, $CDCl_3$) δ 204.0, 138.8, 131.6, 128.2 (2C), 126.3 (2C), 103.4, 95.1, 22.9, 19.3, 13.9, -0.9 (3C); HRMS (ESI): calc. for $C_{15}H_{22}NaSi$ [M+Na]$^+$: 253.1383; found: 253.1380.

4-(Nona-2,3-dien-2-yl)phenyl pivalate (**2r**)

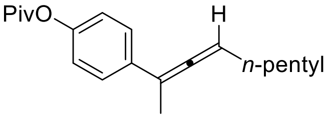

2r

According to the general procedure: $Fe(acac)_3$ (5.3 mg, 0.015 mmol, 5 mol%), substrate **1r** (95 mg, 0.3 mmol, 1.0 equiv.) and methylmagnesium bromide (3.0 M in diethyl ether, 150 μL, 0.45 mmol, 1.5 equiv.) in anhydrous toluene (1.5 mL) were reacted. The reaction mixture was extracted and purified over SiO_2 (ethyl acetate/pentane 1 – 2%) to yield product **2r** (46 mg, 51% isolated yield) as a colorless oil.

^{1}H NMR (400 MHz, $CDCl_3$) δ 7.38 (d, J = 8.5 Hz, 2H), 6.99 (d, J = 8.5 Hz, 2H), 5.46 – 5.42 (m, 1H), 2.12 – 2.07 (m, 5H), 1.50 – 1.42 (m, 2H), 1.44 – 1.26 (m, 13H), 0.90 – 0.87 (m, 3H); ^{13}C NMR (101 MHz, $CDCl_3$) δ 204.2, 177.3, 149.7, 135.4, 126.6, 121.3, 99.8, 93.4, 39.2, 31.5, 29.1, 29.1, 27.3, 22.6, 17.5, 14.2; HRMS (ESI): calc. for $C_{20}H_{28}NaO_2$ $[M+Na]^+$: 323.1982; found: 323.1997.

4-(4-Methylpenta-2,3-dien-2-yl)phenyl acetate (**2s**)

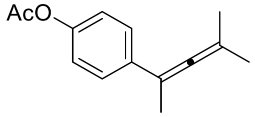

2s

According to the general procedure: $Fe(acac)_3$ (5.3 mg, 0.015 mmol, 5 mol%), substrate **1s** (70 mg, 0.3 mmol, 1.0 equiv.) and methylmagnesium bromide (3.0 M in diethyl ether, 150 µL, 0.45 mmol, 1.5 equiv.) in anhydrous toluene (1.5 mL) were reacted. The reaction mixture was extracted and purified over SiO_2 (ethyl acetate/pentane 1 – 2%) to yield product **2s** (48 mg, 73% isolated yield) as a colorless oil.

^{1}H NMR (400 MHz, $CDCl_3$) δ 7.95 (d, J = 8.2 Hz, 2H), 7.40 (d, J = 8.2 Hz, 2H), 3.90 (s, 3H), 2.06 (s, 3H), 1.81 (s, 6H); ^{13}C NMR (101 MHz, $CDCl_3$) δ 203.3, 167.3, 143.9, 129.6 (2C), 127.6, 125.6 (2C), 98.0, 97.6, 52.1, 20.3 (2C), 17.3; HRMS (ESI): calc. for $C_{14}H_{16}NaO_2$ $[M+Na]^+$: 239.1043; found: 239.1051.

Preparation of γ-allenols 5:

6-Methyl-4-(trifluoromethyl)deca-4,5-dien-1-ol (**5a**)

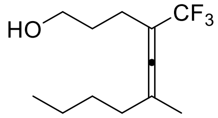

5a

According to the general procedure: $Fe(acac)_3$ (5.3 mg, 0.015 mmol, 5 mol%), substrate **4a** (66 mg, 0.3 mmol, 1.0 equiv.) and methylmagnesium bromide (3.0 M in diethyl ether, 150 µL, 0.45 mmol, 1.5 equiv.) in anhydrous toluene (1.5 mL) were reacted. The reaction mixture was extracted and

purified over SiO_2 (ethyl acetate/pentane 10 – 20%) to yield product **5a** (62 mg, 88%) as a colorless oil.

^{1}H NMR (400 MHz, $CDCl_3$) δ 3.68 (t, *J* = 6.4 Hz, 2H), 2.24 – 2.14 (m, 2H), 2.09 – 1.93 (m, 2H), 1.73 (d, *J* = 19.6 Hz, 6H), 1.48 – 1.27 (m, 4H), 0.90 (t, *J* = 7.2 Hz, 3H); ^{13}C NMR (101 MHz, $CDCl_3$) δ 199.7 (d, *J* = 4.2 Hz), 124.3 (q, *J* = 273.5 Hz), 108.5, 97.2 (q, *J* = 33.4 Hz), 62.2, 33.5, 30.8, 29.4, 23.0, 22.3, 18.6 (d, *J* = 1.4 Hz), 14.0; ^{19}F NMR (377 MHz, $CDCl_3$) δ -64.24; HRMS (ESI): calc. for $C_{12}H_{19}F_3ONa$ $[M+Na]^+$: 259.1280; found: 259.1285.

6-Benzyl-4-methylundeca-4,5-dien-1-ol (**5b**)

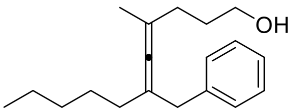

5b

According to the general procedure: $Fe(acac)_3$ (5.3 mg, 0.015 mmol, 5 mol%) substrate **4b** (54 mg, 0.3 mmol, 1.0 equiv.) and benzylmagnesium chloride (2.0 M in diethyl ether, 225 µL, 0.45 mmol, 1.5 equiv.) in anhydrous toluene (1.5 mL) were reacted. The reaction mixture was extracted and purified over SiO_2 (ethyl acetate/pentane 10 – 20%) to yield product **5b** (64 mg, 73%) as a colorless oil.

^{1}H NMR (400 MHz, $CDCl_3$) δ 7.32 – 7.23 (m, 2H), 7.19 (dt, *J* = 6.3, 1.8 Hz, 3H), 3.58 (t, *J* = 6.5 Hz, 2H), 3.26 (s, 2H), 2.03 – 1.84 (m, 4H), 1.67 – 1.56 (m, 5H), 1.43 – 1.19 (m, 7H), 0.95 – 0.83 (m, 3H); ^{13}C NMR (101 MHz, $CDCl_3$) δ 199.5, 140.6, 129.1 (2C), 128.2 (2C), 126.0, 103.6, 99.5, 62.8, 40.5, 32.4, 31.7, 30.8, 30.7, 27.6, 22.7, 19.5, 14.2; HRMS (ESI): calc. for $C_{19}H_{28}ONa$ $[M+Na]^+$: 295.2032; found: 295.2034.

6-Cyclopropyl-4-methyl-7-phenylhepta-4,5-dien-1-ol (**5c**)

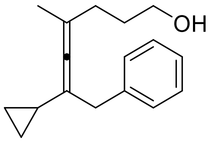

5c

According to the general procedure: $Fe(acac)_3$ (5.3 mg, 0.015 mmol, 5 mol%), substrate **4c** (45 mg, 0.3 mmol, 1.0 equiv.) and benzylmagnesium chloride (2.0 M in diethyl ether, 225 µL, 0.45 mmol, 1.5 equiv.) in anhydrous toluene (1.5 mL) were reacted. The reaction mixture was extracted and

purified over SiO_2 (ethyl acetate/pentane 10 – 20%) to yield product **5c** (49 mg, 68%) as a colorless oil.

^{1}H NMR (400 MHz, $CDCl_3$) δ 7.32 – 7.15 (m, 5H), 3.56 (t, *J* = 6.5 Hz, 2H), 3.37 (s, 2H), 2.00 – 1.84 (m, 2H), 1.62 (s, 3H), 1.61 – 1.50 (m, 2H), 1.25 (s, 1H), 1.08 (tt, *J* = 8.1, 5.0 Hz, 1H), 0.64 – 0.53 (m, 2H), 0.35 – 0.27 (m, 2H); ^{13}C NMR (101 MHz, $CDCl_3$) δ 197.8, 140.5, 129.2 (2C), 128.2 (2C), 126.0, 107.1, 101.4, 62.7, 40.5, 30.7, 30.6, 19.6, 12.7, 7.0 (2C); HRMS (ESI): calc. for $C_{17}H_{22}NaO$ $[M+Na]^+$: 265.1563; found: 265.1571.

7-((tert-Butyldimethylsilyl)oxy)-4,6-dimethylhepta-4,5-dien-1-ol (**5d**)

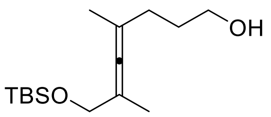

5d

According to the general procedure: $Fe(acac)_3$ (5.3 mg, 0.015 mmol, 5 mol%), substrate **4d** (76 mg, 0.3 mmol, 1.0 equiv.) and methylmagnesium bromide (3.0 M in diethyl ether, 150 µL, 0.45 mmol, 1.5 equiv.) in anhydrous toluene (1.5 mL) were reacted. The reaction mixture was extracted and purified over SiO_2 (ethyl acetate/pentane 10 – 20%) to yield product **5d** (69 mg, 85%) as a colorless oil.

^{1}H NMR (400 MHz, $CDCl_3$) δ 4.05 (s, 2H), 3.66 (t, *J* = 6.4 Hz, 2H), 2.02 (td, *J* = 7.2, 2.0 Hz, 2H), 1.78 – 1.62 (m, 8H), 1.56 (s, 1H), 0.90 (s, 9H), 0.07 (s, 6H); ^{13}C NMR (101 MHz, $CDCl_3$) δ 198.2, 99.3, 99.2, 65.8, 62.7, 30.7, 30.5, 26.0 (3C), 19.3, 18.5, 16.1, -5.1 (2C); HRMS (ESI): calc. for $C_{15}H_{30}NaO_2Si$ $[M+Na]^+$: 293.1907; found: 293.1912.

2,4-Dimethylhepta-2,3-diene-1,7-diol (**5e**)

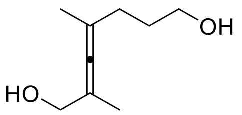

5e

According to the general procedure: $Fe(acac)_3$ (5.3 mg, 0.015 mmol, 5 mol%), substrate **4e** (42 mg, 0.3 mmol, 1.0 equiv.) and methylmagnesium bromide (3.0 M in diethyl ether, 250 µL, 0.75 mmol, 2.5 equiv.) in anhydrous toluene (1.5 mL) were reacted. The reaction mixture was extracted and purified over SiO_2 (ethyl acetate/pentane 20 – 50%) to yield product **5e** (26 mg, 56%) as a colorless oil.

^{1}H NMR (400 MHz, $CDCl_3$) δ 4.03 – 3.91 (m, 2H), 3.67 (t, *J* = 6.3 Hz, 2H), 2.06 (qt, *J* = 14.9, 7.2 Hz, 2H), 1.93 – 1.73 (m, 2H), 1.74 – 1.63 (m, 8H); ^{13}C NMR (101 MHz, $CDCl_3$) δ 196.4, 102.7, 100.4, 64.1, 62.4, 30.8, 30.2, 19.5, 16.1; HRMS (ESI): calc. for $C_9H_{16}NaO_2$ $[M+Na]^+$: 179.1043; found: 179.1045.

6-Benzyl-8-phenylocta-4,5-dien-1-ol (**5fa**)

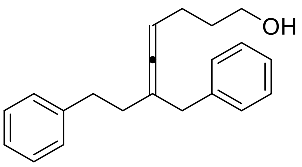

5fa

According to the general procedure: $Fe(acac)_3$ (5.3 mg, 0.015 mmol, 5 mol%), substrate **4f** (60 mg, 0.3 mmol, 1.0 equiv.) and benzylmagnesium bromide (2.0 M in diethyl ether, 225 µL, 0.45 mmol, 1.5 equiv.) in anhydrous toluene (1.5 mL) were reacted. The reaction mixture was extracted and purified over SiO_2 (ethyl acetate/pentane 10 – 20%) to yield product **5fa** (62 mg, 71%) as a colorless oil.

^{1}H NMR (500 MHz, $CDCl_3$) δ 7.32 – 7.24 (m, 4H), 7.23 – 7.14 (m, 6H), 5.12 (tt, *J* = 6.4, 3.0 Hz, 1H), 3.58 (t, *J* = 6.5 Hz, 2H), 3.32 (d, *J* = 2.5 Hz, 2H), 2.73 (td, *J* = 8.2, 3.5 Hz, 2H), 2.31 – 2.18 (m, 2H), 1.98 (qd, *J* = 7.5, 7.1, 1.3 Hz, 2H), 1.60 – 1.49 (m, 2H), 1.20 (s, 1H); ^{13}C NMR (126 MHz, $CDCl_3$) δ 202.2, 142.2, 139.9, 129.1 (2C), 128.5 (2C), 128.4 (2C), 128.3 (2C), 126.3, 125.8, 104.0, 91.9, 62.5, 40.4, 34.1, 33.6, 32.2, 25.6; HRMS (ESI): calc. for $C_{21}H_{24}NaO$ $[M+Na]^+$: 315.1719; found: 315.1712.

6-Methyl-8-phenylocta-4,5-dien-1-ol (**5fb**)

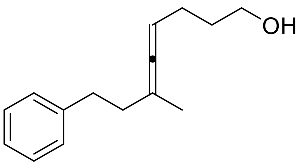

5fb

According to the general procedure: $Fe(acac)_3$ (5.3 mg, 0.015 mmol, 5 mol%), substrate **4f** (60 mg, 0.3 mmol, 1.0 equiv.) and methylmagnesium bromide (3.0 M in diethyl ether, 150 µL, 0.45 mmol, 1.5 equiv.) in anhydrous toluene (1.5 mL) were reacted. The reaction mixture was extracted and purified over SiO_2 (ethyl acetate/pentane 10 – 20%) to yield product **5fb** (44 mg, 68%) as a

colorless oil.

^{1}H NMR (400 MHz, $CDCl_3$) δ 7.31 – 7.24 (m, 2H), 7.23 – 7.10 (m, 3H), 5.06 (tq, J = 6.2, 3.0 Hz, 1H), 3.65 (t, J = 6.5 Hz, 2H), 2.73 (t, J = 7.9 Hz, 2H), 2.31 – 2.18 (m, 2H), 1.99 (td, J = 7.3, 6.4 Hz, 2H), 1.72 (d, J = 2.9 Hz, 3H), 1.66 – 1.53 (m, 2H), 1.29 (s, 1H); ^{13}C NMR (101 MHz, $CDCl_3$) δ 201.5, 142.3, 128.5 (2C), 128.4 (2C), 125.8, 99.5, 90.3, 62.6, 35.8, 34.1, 32.1, 25.6, 19.5; HRMS (ESI): calc. for $C_{15}H_{20}NaO$ [M+Na]$^+$: 239.1406; found: 239.1415.

4,6-Diphenyldeca-4,5-dien-1-ol (**5g**)

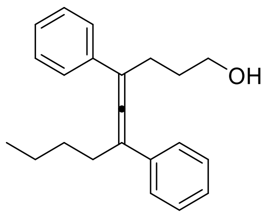

5g

According to the general procedure: Fe(acac)$_3$ (5.3 mg, 0.015 mmol, 5 mol%), substrate **4g** (69 mg, 0.3 mmol, 1.0 equiv.) and phenylmagnesium bromide (3.0 M in diethyl ether, 200 µL, 0.6 mmol, 2.0 equiv.) in anhydrous toluene (1.5 mL) were reacted. The reaction mixture was extracted and purified over SiO_2 (ethyl acetate/pentane 5 – 20%) to yield product **5g** (60 mg, 65%) as a colorless oil.

^{1}H NMR (500 MHz, $CDCl_3$) δ 7.48 – 7.42 (m, 4H), 7.32 (td, J = 7.7, 4.3 Hz, 4H), 7.21 (td, J = 7.5, 3.2 Hz, 2H), 3.74 (q, J = 6.1 Hz, 2H), 2.66 (t, J = 7.6 Hz, 2H), 2.61 – 2.51 (m, 2H), 1.97 – 1.77 (m, 2H), 1.63 – 1.51 (m, 2H), 1.43 (h, J = 7.3 Hz, 2H), 1.31 – 1.24 (m, 1H), 0.92 (t, J = 7.3 Hz, 3H); ^{13}C NMR (126 MHz, $CDCl_3$) δ 205.2, 137.0, 136.9, 128.6 (4C), 126.9 (2C), 126.1 (2C), 126.0 (2C), 109.9, 108.7, 62.9, 31.3, 30.5, 30.3, 26.7, 22.9, 14.1; HRMS (ESI): calc. for $C_{22}H_{26}ONa$ [M+Na]$^+$: 329.1876; found: 329.1868.

4-(4-Fluorophenyl)-6-methyldeca-4,5-dien-1-ol (**5h**)

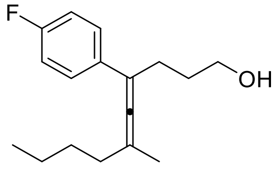

5h

According to the general procedure: Fe(acac)$_3$ (5.3 mg, 0.015 mmol, 5 mol%), substrate **4h** (74 mg,

0.3 mmol, 1.0 equiv.) and methylmagnesium bromide (3.0 M in diethyl ether, 150 μL, 0.45 mmol, 1.5 equiv.) in anhydrous toluene (1.5 mL) were reacted. The reaction mixture was extracted and purified over SiO_2 (ethyl acetate/pentane 5 – 20%) to yield product **5h** (53 mg, 67%) as a colorless oil.

^1H NMR (400 MHz, $CDCl_3$) δ 7.32 (dd, *J* = 8.8, 5.4 Hz, 2H), 6.98 (t, *J* = 8.8 Hz, 2H), 3.74 (t, *J* = 6.5 Hz, 2H), 2.49 – 2.42 (m, 2H), 2.09 – 2.03 (m, 2H), 1.84 – 1.74 (m, 5H), 1.48 – 1.20 (m, 4H), 0.89 (t, *J* = 7.2 Hz, 3H); ^{13}C NMR (101 MHz, $CDCl_3$) δ 201.0 (d, *J* = 1.9 Hz), 161.6 (d, *J* = 245.1 Hz), 134.3 (d, *J* = 3.3 Hz), 127.4 (d, *J* = 7.8 Hz, 2C), 115.1 (d, *J* = 21.4 Hz, 2C), 103.9, 103.4, 62.7, 34.2, 31.2, 30.0, 26.8, 22.7, 19.1, 14.1; ^{19}F NMR (377 MHz, $CDCl_3$) δ -117.03; HRMS (ESI): calc. for $C_{17}H_{23}FONa$ $[M+Na]^+$: 285.1625; found: 285.1624.

4-Phenylhepta-4,5-dien-1-ol (**5i**)

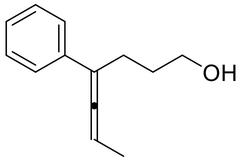

5i

According to the general procedure: $Fe(acac)_3$ (5.3 mg, 0.015 mmol, 5 mol%), substrate **4i** (52 mg, 0.3 mmol, 1.0 equiv.) and methylmagnesium bromide (3.0 M in diethyl ether, 150 μL, 0.45 mmol, 1.5 equiv.) in anhydrous toluene (1.5 mL) were reacted. The reaction mixture was extracted and purified over SiO_2 (ethyl acetate/pentane 5 – 20%) to yield product **5i** (31 mg, 55%) as a colorless oil.

^1H NMR (400 MHz, $CDCl_3$) δ 7.41 (d, *J* = 7.4 Hz, 2H), 7.32 (t, *J* = 7.8 Hz, 2H), 7.20 (t, *J* = 7.3 Hz, 1H), 5.51 (dt, *J* = 6.6, 3.2 Hz, 1H), 3.74 (t, *J* = 6.4 Hz, 2H), 2.59 – 2.43 (m, 2H), 1.90 – 1.73 (m, 5H), 1.45 (br. s, 1H); ^{13}C NMR (101 MHz, $CDCl_3$) δ 204.6, 137.4, 128.5 (2C), 126.6, 126.1 (2C), 104.6, 89.5, 62.6, 31.1, 26.2, 14.5; HRMS (ESI): calc. for $C_{13}H_{16}ONa$ $[M+Na]^+$: 211.1093; found: 211.1082.

Preparation of β-allenols 7:

Table S1. Preparation of β-allenols **7**. [a]

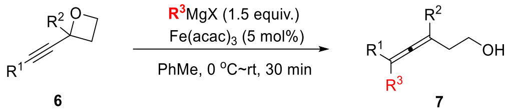

Entry	Substrate (**6**)	R^3MgX	Product (**7**)	Yield of **7** [%][b]
1	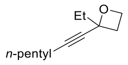 **6a**	BnMgCl	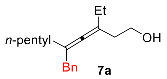 **7a**	78
2	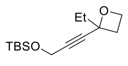 **6b**	PhMgBr	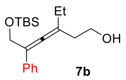 **7b**	81
3	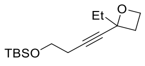 **6c**	EtMgBr	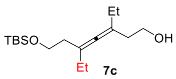 **7c**	56
4	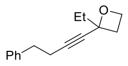 **6d**	CyMgBr	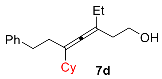 **7d**	71[c]
5	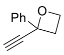 **6e**	MeMgBr	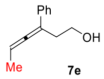 **7e**	57

[a]Reaction conditions: 0.2 M solution of oxetane **6** (0.3 mmol) in PhMe, $Fe(acac)_3$ (5 mol%) with dropwise addition of Grignard reagent (1.5 equiv). [b]Isolated yield. [c]Using 10 mol% of TMEDA as an additive.

5-Benzyl-3-ethyldeca-3,4-dien-1-ol (**7a**)

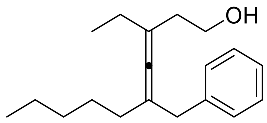

7a

According to the general procedure: $Fe(acac)_3$ (5.3 mg, 0.015 mmol, 5 mol%), substrate **6a** (54 mg, 0.3 mmol, 1.0 equiv.) and benzylmagnesium chloride (2.0 M in diethyl ether, 225 µL, 0.45 mmol, 1.5 equiv.) in anhydrous toluene (1.5 mL) were reacted. The reaction mixture was extracted and purified over SiO_2 (ethyl acetate/pentane 10 – 20%) to yield product **7a** (64 mg, 78%) as a colorless oil.

^{1}H NMR (400 MHz, $CDCl_3$) δ 7.31 – 7.25 (m, 2H), 7.22 – 7.16 (m, 3H), 3.56 (qd, J = 5.5, 2.8 Hz, 2H), 3.35 – 3.21 (m, 2H), 2.19 – 2.01 (m, 2H), 2.00 – 1.93 (m, 2H), 1.89 (q, J = 7.4 Hz, 2H), 1.47

– 1.38 (m, 2H), 1.34 – 1.21 (m, 5H), 0.94 (t, *J* = 7.4 Hz, 3H), 0.91 – 0.84 (m, 3H); ^{13}C NMR (101 MHz, $CDCl_3$) δ 198.5, 140.0, 129.2 (2C), 128.3 (2C), 126.3, 107.1, 104.0, 60.9, 40.4, 36.2, 33.0, 31.7, 27.7, 26.3, 22.7, 14.2, 12.5; HRMS (ESI): calc. for $C_{19}H_{28}ONa$ $[M+Na]^+$: 295.2032; found: 295.2034.

6-((tert-Butyldimethylsilyl)oxy)-3-ethyl-5-phenylhexa-3,4-dien-1-ol (**7b**)

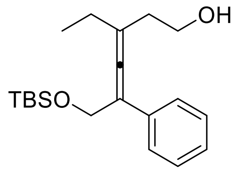

7b

According to the general procedure: $Fe(acac)_3$ (5.3 mg, 0.015 mmol, 5 mol%), substrate **6b** (76 mg, 0.3 mmol, 1.0 equiv.) and phenylmagnesium bromide (3.0 M in diethyl ether, 150 µL, 0.45 mmol, 1.5 equiv.) in anhydrous toluene (1.5 mL) were reacted. The reaction mixture was extracted and purified over SiO_2 (ethyl acetate/pentane 10 – 20%) to yield product **7b** (81 mg, 81%) as a colorless oil.

^{1}H NMR (500 MHz, $CDCl_3$) δ 7.35 – 7.28 (m, 4H), 7.23 – 7.18 (m, 1H), 4.64 (d, *J* = 12.6 Hz, 1H), 4.56 (d, *J* = 12.6 Hz, 1H), 3.75 (dddd, *J* = 11.5, 8.6, 5.5, 3.2 Hz, 1H), 3.66 (dddd, *J* = 11.4, 8.8, 5.8, 3.6 Hz, 1H), 3.24 (dd, *J* = 8.2, 5.6 Hz, 1H), 2.44 (ddd, *J* = 15.6, 6.0, 3.1 Hz, 1H), 2.24 (ddd, *J* = 15.6, 8.6, 3.6 Hz, 1H), 2.14 (q, *J* = 7.5 Hz, 2H), 1.09 (t, *J* = 7.3 Hz, 3H), 0.93 (s, 9H), 0.13 (s, 6H); ^{13}C NMR (126 MHz, $CDCl_3$) δ 199.1, 135.9, 128.6 (2C), 127.0, 125.8 (2C), 109.6, 109.0, 62.0, 60.1, 36.3, 26.6, 26.0 (3C), 18.6, 12.5, -5.2, -5.4; HRMS (ESI): calc. for $C_{20}H_{32}O_2SiNa$ $[M+Na]^+$: 355.2064; found: 355.2049.

7-((tert-Butyldimethylsilyl)oxy)-3,5-diethylhepta-3,4-dien-1-ol (**7c**)

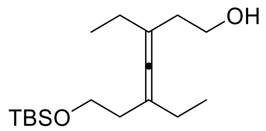

7c

According to the general procedure: $Fe(acac)_3$ (5.3 mg, 0.015 mmol, 5 mol%), substrate **6c** (81 mg, 0.3 mmol, 1.0 equiv.) and ethylmagnesium chloride (3.0 M in diethyl ether, 150 µL, 0.45 mmol, 1.5 equiv.) in anhydrous toluene (1.5 mL) were reacted. The reaction mixture was extracted and purified over SiO_2 (ethyl acetate/pentane 10 – 20%) to yield product **7c** (50 mg, 56%) as a colorless

oil.

^{1}H NMR (400 MHz, $CDCl_3$) δ 3.72 (t, J = 6.7 Hz, 4H), 2.27 – 2.14 (m, 4H), 2.03 – 1.90 (m, 4H), 0.98 (td, J = 7.4, 1.4 Hz, 6H), 0.89 (s, 9H), 0.06 (s, 6H); ^{13}C NMR (101 MHz, $CDCl_3$) δ 196.8, 105.8, 105.1, 62.3, 60.9, 36.6, 36.4, 26.5, 26.4, 26.2 (3C), 18.6, 12.6, 12.5, -5.1, -5.1; HRMS (ESI): calc. for $C_{17}H_{34}O_2SiNa$ [M+Na]$^+$: 321.2220; found: 321.2211.

5-Cyclohexyl-3-ethyl-7-phenylhepta-3,4-dien-1-ol (**7d**)

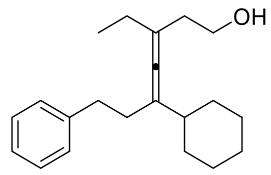

7d

According to the general procedure: $Fe(acac)_3$ (5.3 mg, 0.015 mmol, 5 mol%), substrate **6d** (64 mg, 0.3 mmol, 1.0 equiv.) and cyclohexylmagnesium chloride (2.0 M in diethyl ether, 225 µL, 0.45 mmol, 1.5 equiv.) in anhydrous toluene (1.5 mL) were reacted. The reaction mixture was extracted and purified over SiO_2 (ethyl acetate/pentane 10 – 20%) to yield product **7d** (64 mg, 71%) as a colorless oil.

^{1}H NMR (400 MHz, $CDCl_3$) δ 7.31 – 7.24 (m, 2H), 7.21 – 7.14 (m, 3H), 3.66 (t, J = 6.4 Hz, 2H), 2.69 (t, J = 7.7 Hz, 2H), 2.30 (ddd, J = 9.8, 6.7, 1.8 Hz, 2H), 2.18 (t, J = 6.3 Hz, 2H), 1.94 (qd, J = 7.3, 0.9 Hz, 2H), 1.88 – 1.70 (m, 5H), 1.70 – 1.62 (m, 1H), 1.54 (d, J = 9.2 Hz, 1H), 1.34 – 1.00 (m, 5H), 0.95 (t, J = 7.4 Hz, 3H); ^{13}C NMR (101 MHz, $CDCl_3$) δ 196.6, 142.5, 128.5 (2C), 128.4 (2C), 125.8, 112.4, 105.4, 61.4, 41.7, 36.5, 34.6, 33.3, 32.9 (2C), 26.8 (2C), 26.6, 26.5, 12.5; HRMS (ESI): calc. for $C_{21}H_{30}ONa$ [M+Na]$^+$: 321.2189; found: 321.2178.

3-Phenylhexa-3,4-dien-1-ol (**7e**)

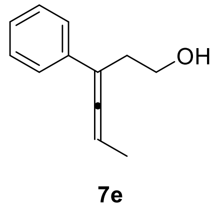

7e

According to the general procedure: $Fe(acac)_3$ (5.3 mg, 0.015 mmol, 5 mol%), substrate **6e** (48 mg, 0.3 mmol, 1.0 equiv.) and methylmagnesium chloride (3.0 M in diethyl ether, 150 µL, 0.45 mmol, 1.5 equiv.) in anhydrous toluene (1.5 mL) were reacted. The reaction mixture was extracted and

purified over SiO_2 (ethyl acetate/pentane 10 – 20%) to yield product **7e** (30 mg, 57%) as a colorless oil.

^{1}H NMR (500 MHz, $CDCl_3$) δ 7.40 (d, *J* = 8.4 Hz, 2H), 7.32 (t, *J* = 7.6 Hz, 2H), 7.24 – 7.19 (m, 1H), 5.57 (qt, *J* = 6.9, 3.1 Hz, 1H), 3.86 (t, *J* = 6.2 Hz, 2H), 2.70 (tt, *J* = 6.5, 3.4 Hz, 2H), 1.80 (d, *J* = 7.1 Hz, 3H), 1.57 (s, 1H); ^{13}C NMR (126 MHz, $CDCl_3$) δ 204.5, 136.9, 128.6 (2C), 126.9, 126.1 (2C), 102.1, 90.0, 61.4, 33.3, 14.5; HRMS (ESI): calc. for $C_{12}H_{14}ONa$ $[M+Na]^+$: 197.0937; found: 197.0923.

2. Additional experiments

Experiments for evaluating the transfer of chirality

Propargyl methyl ethers (*R*)-**1l**, (*S*)-**1l**, (*R*)-**1m**, and (*R*)-**1n** were prepared *via* kinetic resolution of propargyl alcohols **S5**:

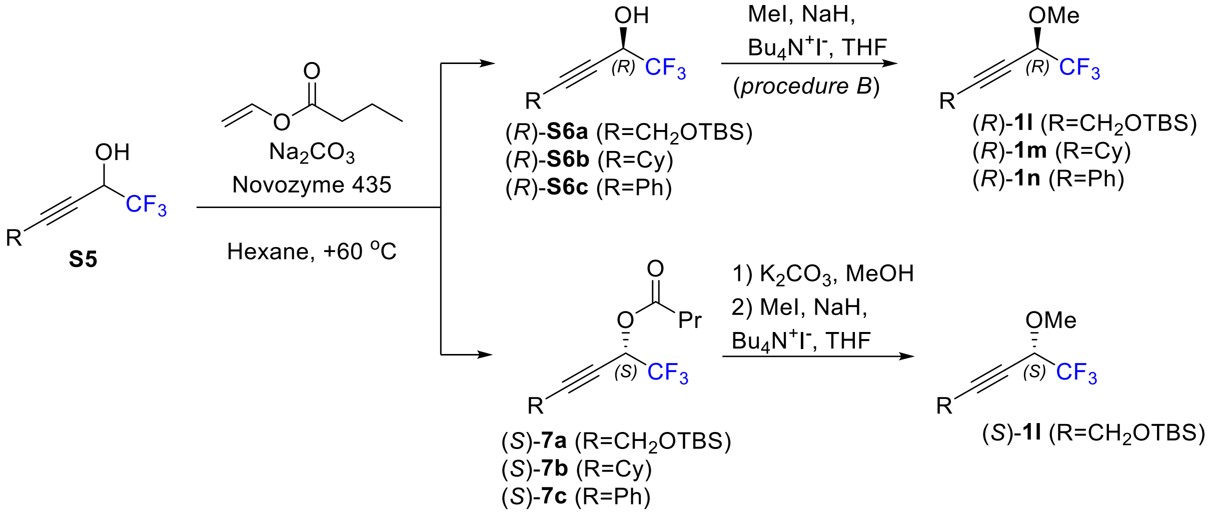

Preparation of alcohol (*R*)-**S6b** (representative procedure):

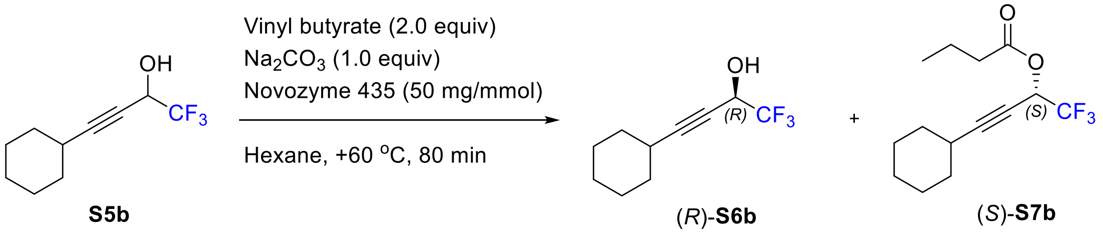

Reaction procedure: Vinyl butyrate (1.27 mL, 10.0 mmol, 2.0 equiv), Na_2CO_3 (530 mg, 5.0 mmol, 1.0 equiv) and *Candida antarctica* lipase B (CALB) as Novozyme 435 (25 mg) were mixed in anhydrous hexane (3.0 mL) in a 10 mL microwave vial. To this mixture was added propargyl alcohol **S5b**[7] (1.03 g, 5.0 mmol, 1.0 equiv). The reaction mixture was stirred at 60 °C for 80 minutes. After that, the mixture was filtered and the solvent evaporated. The crude product was purified *via* column chromatography on silica gel (eluent: petroleum ether/ethyl ether = 20/1) to afford product (*R*)-**S6b** (485 mg, 47% yield, >99% ee) and butyrate (*S*)-**S7b** (622 mg, 45%, ee not determined). GC: IVADEX-I, 80-2.0 °C/min-150-5.0 °C/min-200, t_R: 24.5 min for major isomer and 24.1 min for minor isomer.

Racemate (**S6b**):

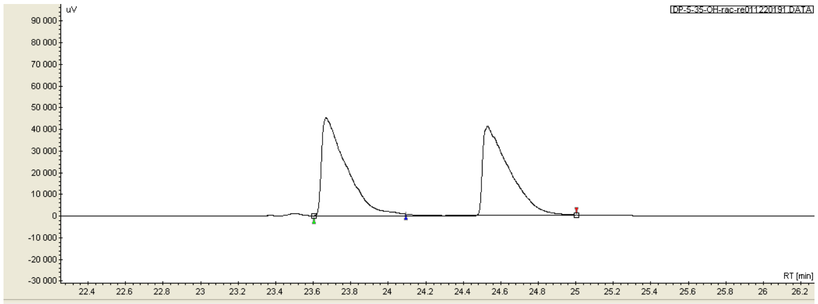

#	Name	Time [Min]	Quantity [% Area]	Height [uV]	Area [uV.Min]	Area % [%]
1	UNKNOWN	23.67	50.84	45327.6	6885.2	50.842
2	UNKNOWN	24.53	49.16	41069.1	6657.3	49.158
Total			100.00	86396.7	13542.5	100.000

Enantiomer ((*R*)-**S6b**):

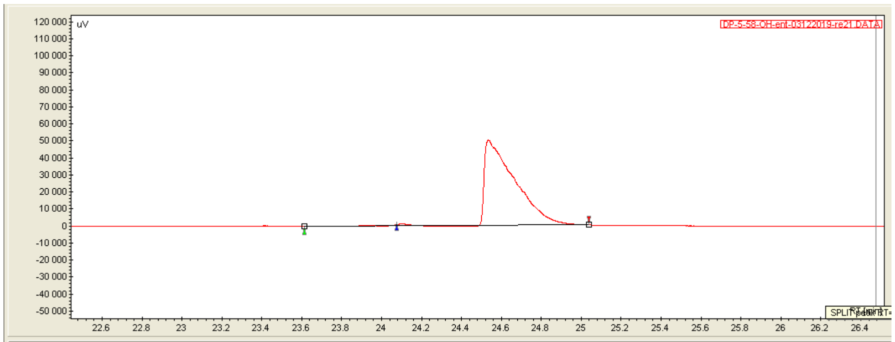

#	Name	Time [Min]	Quantity [% Area]	Height [uV]	Area [uV.Min]	Area % [%]
1	UNKNOWN	24.08	0.15	596.0	13.2	0.149
2	UNKNOWN	24.53	99.85	50246.8	8834.8	99.851
Total			100.00	50842.9	8848.0	100.000

Propargyl methyl ether (*R*)-**1m** was prepared by methylation of (*R*)-**S6b** (*procedure B*):

(R)-(4,4,4-Trifluoro-3-methoxybut-1-yn-1-yl)cyclohexane ((*R*)-**1m**)

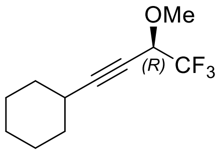

(*R*)-**1m**

73% isolated yield, colorless oil, >99% ee. ^{1}H NMR (500 MHz, $CDCl_3$) δ 4.40 (qd, *J* = 5.8, 1.9 Hz, 1H), 3.51 (s, 3H), 2.48 (tt, *J* = 10.6, 8.6, 4.1 Hz, 1H), 1.88 – 1.76 (m, 2H), 1.75 – 1.64 (m, 2H), 1.54 – 1.45 (m, 3H), 1.38 – 1.28 (m, 3H); ^{13}C NMR (126 MHz, $CDCl_3$) δ 122.74 (q, *J* = 281.6 Hz), 94.53, 70.31 (q, *J* = 34.7 Hz), 70.15 – 70.13 (m), 57.06, 32.21 (2C), 28.98, 25.91 (2C), 24.70. ^{19}F NMR (377 MHz, $CDCl_3$) δ -77.23, -77.24; HRMS (APCI): calc. for $C_{11}H_{15}F_3OH$ $[M+H]^+$: 221.1148; found: 221.1150.

GC: IVADEX-I, 80-2.0 °C/min-150-5.0 °C/min-200, t_R: 11.9 min for major isomer and 12.2 min for minor isomer.

Racemate:

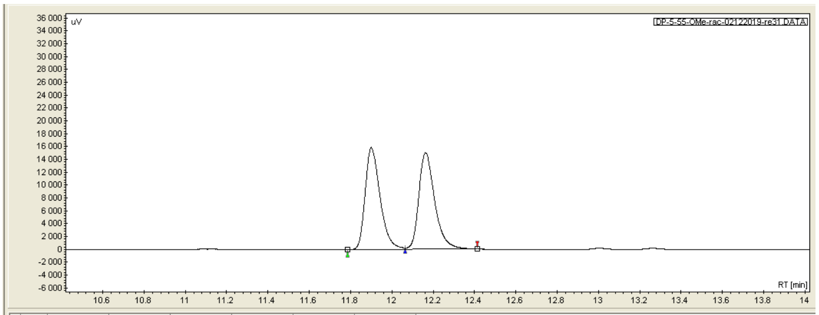

#	Name	Time [Min]	Quantity [% Area]	Height [uV]	Area [uV.Min]	Area % [%]
1	UNKNOWN	11.90	49.72	15920.6	1331.0	49.721
2	UNKNOWN	12.16	50.28	15042.8	1345.9	50.279
Total			100.00	30963.4	2676.9	100.000

Enantiomer:

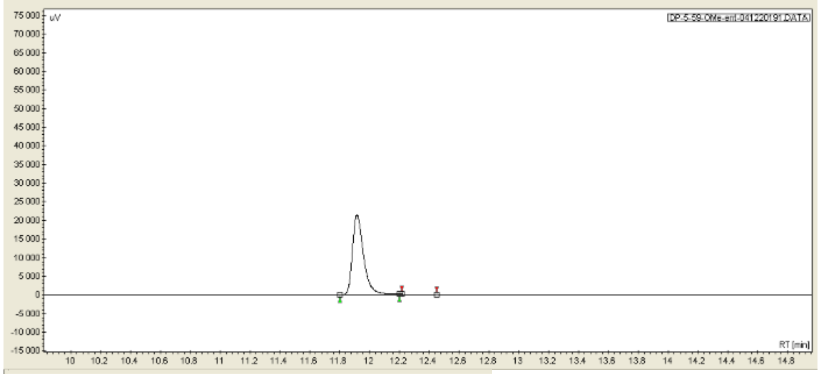

#	Name	Time [Min]	Quantity [% Area]	Height [uV]	Area [uV.Min]	Area % [%]
1	UNKNOWN	11.92	100.00	21451.5	1866.4	100.000
2	UNKNOWN	12.22	0.00	0.0	0.0	0.000
Total			100.00	21451.5	1866.4	100.000

(R)-tert-Butyldimethyl((5,5,5-trifluoro-4-methoxypent-2-yn-1-yl)oxy)silane ((*R*)-**1l**)

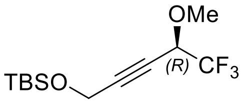

(*R*)-**1l**

73% isolated yield starting from (*R*)-**S6a**, colorless oil, 98% ee. ^{1}H NMR (500 MHz, $CDCl_3$) δ 4.43 (dddd, *J* = 7.5, 5.8, 3.8, 1.7 Hz, 1H), 4.39 (d, *J* = 1.7 Hz, 2H), 3.53 (s, 3H), 0.91 (s, 9H), 0.13 (s, 6H); ^{13}C NMR (126 MHz, $CDCl_3$) δ 122.5 (q, *J* = 281.8 Hz), 88.2, 74.7 (d, *J* = 2.2 Hz), 70.3 (q, *J* = 35.1 Hz), 57.5, 51.6, 25.8 (3C), 18.4, -5.1 (2C); ^{19}F NMR (377 MHz, $CDCl_3$) δ -76.8; HRMS (ESI): calc. for $C_{12}H_{21}F_3NaO_2Si$ $[M+Na]^+$: 305.1155; found: 305.1162.

GC: Hydrodex β-DM, 50-1.0 °C/min-170, t_R: 38.2 min for major isomer and 38.9 min for minor isomer.

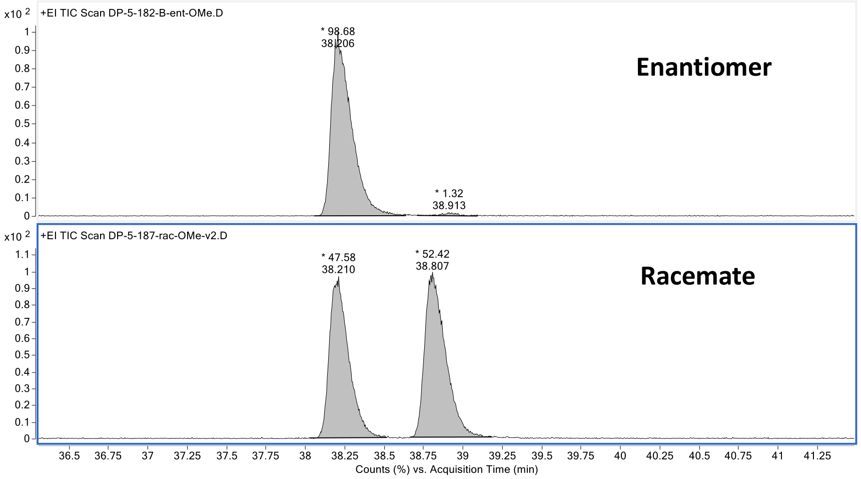

(S)-tert-Butyldimethyl((5,5,5-trifluoro-4-methoxypent-2-yn-1-yl)oxy)silane ((*S*)-**1l**)

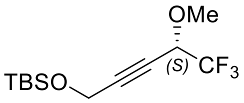

(*S*)-**1l**

65% isolated yield over two steps starting from (*S*)-**S7a**, colorless oil, >99% ee.

GC: Hydrodex β-DM, 50-1.0 ºC/min-170, t_R: 38.8 min for major isomer and 38.2 min for minor isomer.

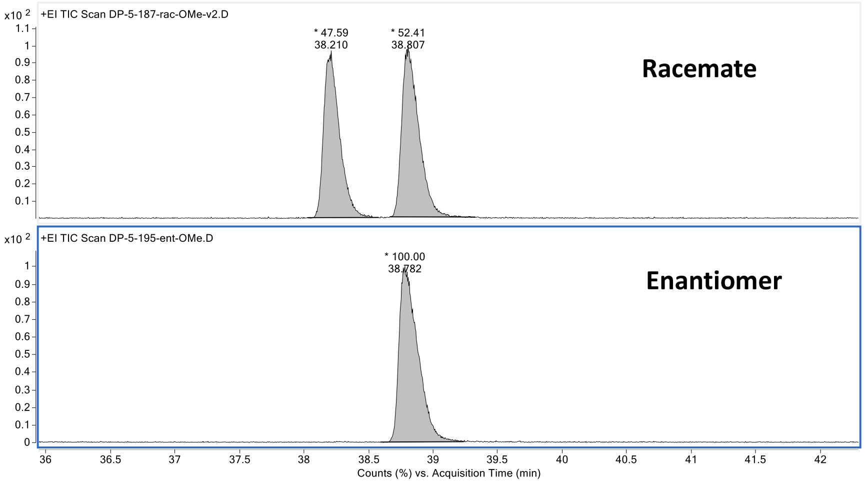

(R)-(4,4,4-Trifluoro-3-methoxybut-1-yn-1-yl)benzene ((*R*)-**1n**)

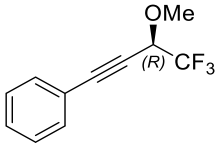

(*R*)-**1n**

67% isolated yield from (*R*)-**S6c**, colorless oil, 94% ee. ^{1}H NMR (400 MHz, $CDCl_3$) δ 7.53 – 7.48 (m, 2H), 7.42 – 7.31 (m, 3H), 4.64 (q, *J* = 5.8 Hz, 1H), 3.61 (s, 3H); ^{13}C NMR (101 MHz, $CDCl_3$) δ 132.2 (2C), 129.6, 128.6 (2C), 122.6 (q, *J* = 281.8 Hz), 121.3, 89.1, 78.9 – 78.6 (m), 70.8 (q, *J* = 35.0 Hz), 57.6; ^{19}F NMR (377 MHz, $CDCl_3$) δ -76.7; HRMS (ESI): calc. for $C_{11}H_9F_3NaO$ $[M+Na]^+$: 237.0498; found: 237.0491.

GC: Hydrodex β-DM, 60°C for 100 min (isocratic), t_R: 80.1 min for major isomer and 82.8 min for minor isomer.

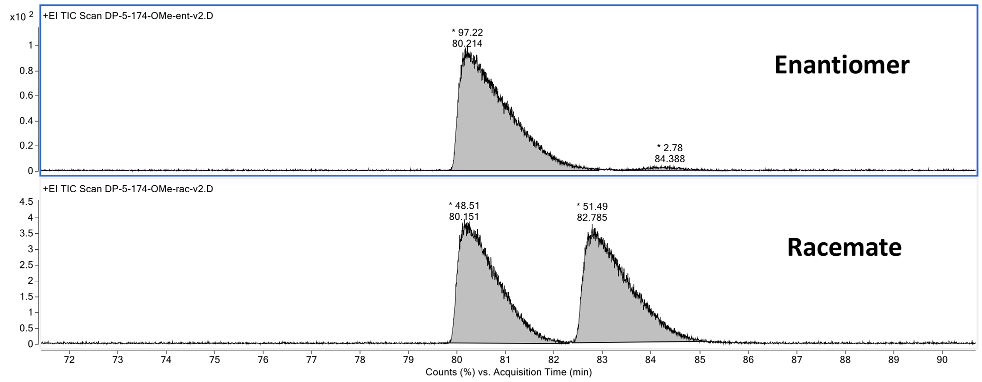

Iron-catalyzed synthesis of optically enriched allenes __2l__-__n__:

(1-Cyclohexyl-4,4,4-trifluorobuta-1,2-dien-1-yl)benzene ((*R*)-**2m**):

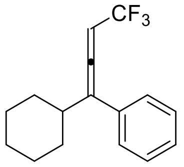

2m

Product (*R*)-**2m** (75% yield, >99% ee, absolute stereoconfiguration was not determined) was obtained as a colorless oil under standard reaction conditions (following the general procedure) using 7.5 mol% of $Fe(acac)_3$ as the catalyst, PhMgBr (2.0 equiv) and (*R*)-**1m** (0.3 mmol, >99% ee) as the substrate. ^{1}H NMR (400 MHz, $CDCl_3$) δ 7.40 – 7.33 (m, 4H), 7.33 – 7.27 (m, 1H), 5.77 (qd, *J* = 5.7, 2.5 Hz, 1H), 2.52 (tq, *J* = 11.4, 3.1 Hz, 1H), 1.91 (dqd, *J* = 12.9, 3.2, 2.8, 1.6 Hz, 2H), 1.87 – 1.68 (m, 3H), 1.46 – 1.12 (m, 5H); ^{13}C NMR (101 MHz, $CDCl_3$) δ 205.4 (q, *J* = 5.8 Hz), 134.0 (d, *J* = 1.6 Hz), 128.9 (2C), 128.2, 127.1 (2C), 123.0 (q, *J* = 270.8 Hz), 119.9, 89.3 (q, *J* = 38.6 Hz), 38.5, 32.6, 32.4, 26.6, 26.5, 26.3; ^{19}F NMR (377 MHz, $CDCl_3$) δ -60.13, -60.14; HRMS (ESI): calc.

for $C_{16}H_{17}F_3Na$ [M+Na]$^+$: 289.1175; found: despite repeated attempts, we were not able to obtain high-resolution mass data for **2m**.

GC: Hydrodex β-DM, 50-1.0 ºC/min-170, t_R: 73.0 min for major isomer and 71.4 min for minor isomer.

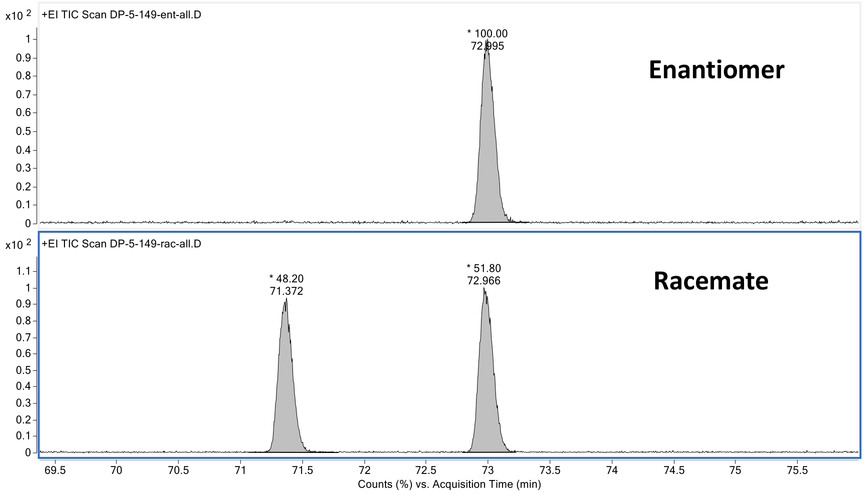

(R)-tert-Butyldimethyl((5,5,5-trifluoro-2-phenylpenta-2,3-dien-1-yl)oxy)silane ((*R*)-**2l**):

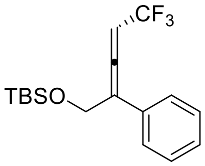

(*R*)-**2l**

Product (*R*)-**2l** (78% yield, 98% ee) was obtained as a colorless oil under standard reaction conditions (following the general procedure) using 7.5 mol% of $Fe(acac)_3$ as the catalyst, PhMgBr (2.0 equiv) and (*R*)-**1l** (0.3 mmol, 98% ee) as the substrate. ^{1}H NMR (400 MHz, $CDCl_3$) δ 7.42 – 7.28 (m, 5H), 5.88 (qt, *J* = 5.8, 2.9 Hz, 1H), 4.66 (dd, *J* = 2.9, 0.8 Hz, 2H), 0.90 (s, 9H), 0.11 – 0.09 (m, 6H); ^{13}C NMR (101 MHz, $CDCl_3$) δ 205.4 (q, *J* = 5.8 Hz), 132.0, 128.9 (2C), 128.6, 126.9 (2C), 124.0 (q, *J* = 271.0 Hz), 114.4, 90.0 (q, *J* = 39.0 Hz), 61.7 – 61.6 (m), 25.9 (3C), 18.4, -5.3, -5.4; ^{19}F NMR (377 MHz, $CDCl_3$) δ -60.0; HRMS (APCI): calc. for $C_{17}H_{23}F_3OSiH$ [M+H]$^+$: 329.1543; found: 329.1554.

Absolute stereoconfiguration was determined by using X-ray diffraction analysis (see below).

GC: Hydrodex β-DM, 50-1.0 ºC/min-170, t_R: 71.9 min for major isomer and 72.5 min for minor isomer.

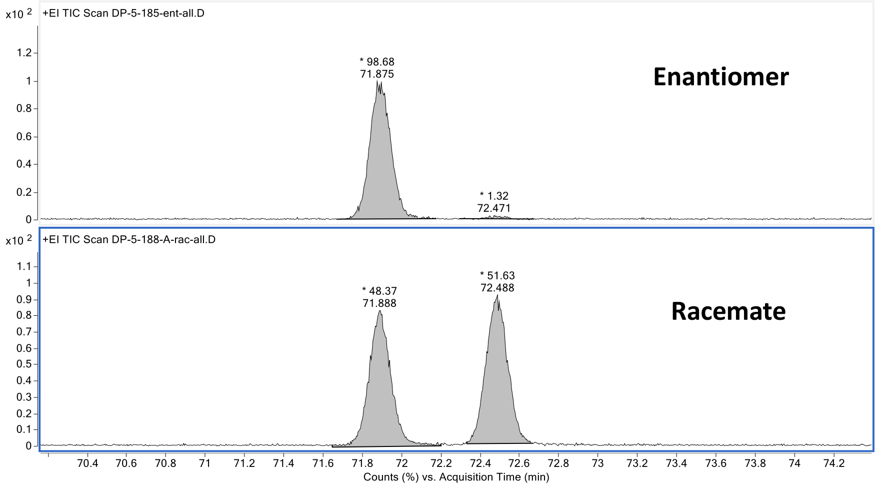

(S)-tert-Butyldimethyl((5,5,5-trifluoro-2-phenylpenta-2,3-dien-1-yl)oxy)silane ((*S*)-**2l**):

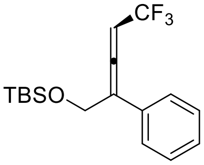

(*S*)-**2l**

Product (*S*)-**2l** (78% yield, >99% ee) was obtained as a colorless oil under standard reaction conditions (following the general procedure) using 7.5 mol% of $Fe(acac)_3$ as the catalyst, PhMgBr (2.0 equiv) and (*S*)-**1l** (0.3 mmol, >99% ee) as the substrate.

GC: Hydrodex β-DM, 50-1.0 ºC/min-170, t_R: 72.5 min for major isomer and 71.9 min for minor isomer.

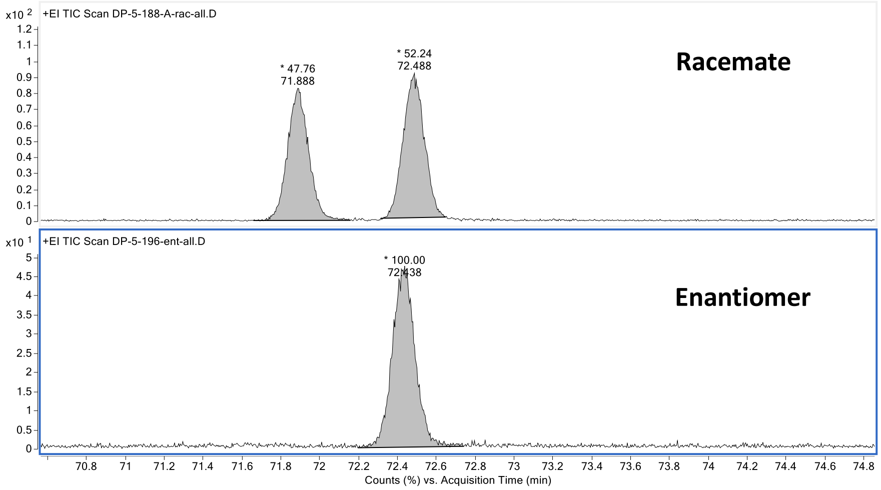

1-Chloro-4-(4,4,4-trifluoro-1-phenylbuta-1,2-dien-1-yl)benzene (**2n**):

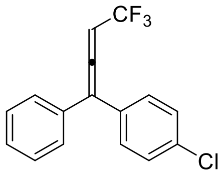

2n

Product **2n** (68% yield, 92% ee, absolute stereoconfiguration was not determined) was obtained as a colorless oil under standard reaction conditions (following the general procedure) using 7.5 mol% of $Fe(acac)_3$ as the catalyst, PhMgBr (2.0 equiv) and (*R*)-**1n** (0.3 mmol, 94% ee) as the substrate. ^{1}H NMR (400 MHz, $CDCl_3$) δ 7.41 – 7.34 (m, 5H), 7.33 – 7.30 (m, 2H), 7.29 – 7.25 (m, 2H), 5.97 (q, *J* = 5.6 Hz, 1H); ^{13}C NMR (101 MHz, $CDCl_3$) δ 206.5 (q, *J* = 5.8 Hz), 134.8, 133.6, 132.6, 130.1 (2C), 129.1 (2C), 129.0 (3C), 128.8 (2C), 122.6 (q, *J* = 271.5 Hz), 116.2, 89.0 (q, *J* = 39.1 Hz); ^{19}F NMR (377 MHz, $CDCl_3$) δ -60.0; HRMS (ESI): calc. for $C_{16}H_{10}ClF_3Na$ $[M+Na]^+$: 317.0315; found: 317.0312.

GC: Hydrodex β-DM, 110 ºC for 200min (isocratic), t_R: 72.5 min for major isomer and 71.9 min for minor isomer.

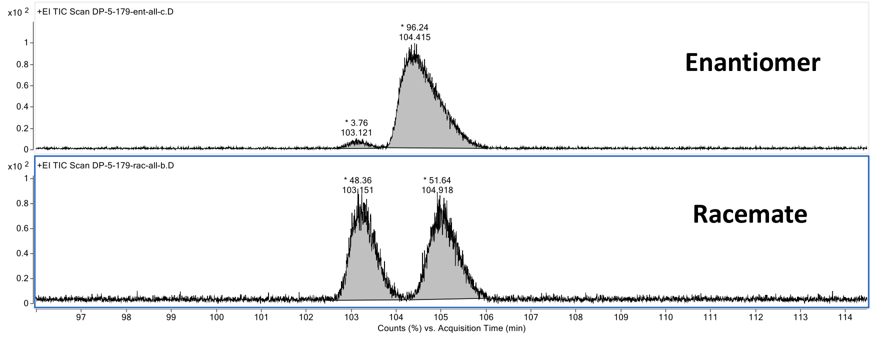

Preparation of (R)-5,5,5-trifluoro-2-phenylpenta-2,3-dien-1-yl 3,5-dinitrobenzoate ((*R*)-**9**):

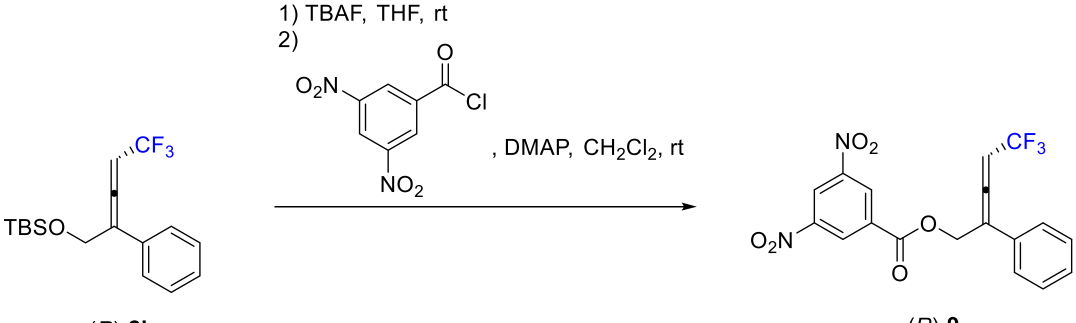

Reaction procedure: A stirred solution of trifluoromethyl allene (*R*)-**2l** (obtained from Fe-catalyzed cross-coupling of (*R*)-**1l** with PhMgBr, 101 mg, 0.31 mmol, 1.0 equiv, 98% ee) in anhydrous THF (0.5 mL) was cooled to 0 ºC under argon atmosphere and TBAF (1.0 M solution

in THF, 370 µL, 0.37 mmol, 1.2 equiv) was added dropwise. After full consumption of the starting material (1h) the reaction was quenched with water (2 mL), extracted with diethyl ether, and the combined organic phases were dried over Na_2SO_4. The evaporated crude mixture was purified over SiO_2 (eluent: ethyl acetate/pentane 20 – 50%) to yield the allenic alcohol product (64 mg, 97%) as a colorless oil, that was used in the next step without characterization.

To a stirred solution of allenic alcohol obtained in the previous step (64 mg, 0.3 mmol, 1.0 equiv) in CH_2Cl_2 (2.0 mL) at 0 °C were added DMAP (40.3 mg, 0.33 mmol, 1.1 equiv) and 3,5-dinitrobenzoyl chloride (DNBC, 103.8 mg, 0.45 mmol, 1.5 equiv). The resulting reaction mixture was stirred at room temperature for 3 hours and the solvent was evaporated under vacuum. The crude mixture was purified over SiO_2 (eluent: ethyl acetate/pentane 5 – 20%) to yield the ester product (*R*)-**9** (88 mg, 72%) as a white crystalline solid. ^{1}H NMR (400 MHz, $CDCl_3$) δ 9.24 (t, *J* = 2.1 Hz, 1H), 9.15 (d, *J* = 2.2 Hz, 2H), 7.49 – 7.34 (m, 5H), 6.03 (qt, *J* = 5.6, 2.7 Hz, 1H), 5.45 (t, *J* = 2.8 Hz, 2H); ^{13}C NMR (101 MHz, $CDCl_3$) δ 205.7 (q, *J* = 5.6 Hz), 162.1, 148.9 (2C), 133.3, 130.4, 129.6 (2C), 129.4, 129.4 (2C), 126.5 (2C), 124.9 (q, *J* = 274.0 Hz), 122.9, 109.7, 91.4 (q, *J* = 39.4 Hz), 63.2 (q, *J* = 1.4 Hz); ^{19}F NMR (377 MHz, $CDCl_3$) δ -60.1; HRMS (ESI): calc. for $C_{18}H_{11}F_3N_2NaO_6$ $[M+Na]^+$: 431.0461; found: 431.0462.

Crystal structure determination of (*R*)-9:

Crystals of compound (*R*)-**9** were analyzed by single crystal X-ray diffraction using Cu Kα radiation on a Bruker D8 VENTURE diffractometer equipped with a PHOTON II detector. The data sets were reduced and absorption corrections applied using the Bruker APEX3 suite. The crystal structure was solved and refined by SHELXT and SHELXL respectively.[8] The crystal structure was refined using full-matrix least-squares based on F^2 with all non-hydrogen atoms anisotropically defined. A summary of the crystallographic data and refinement parameters are provided in Table S2 below. CCDC deposition number 2042627 (for (*R*)-**9**), contain the supplementary crystallographic data for this paper. These data can be obtained free of charge from The Cambridge Crystallographic Data Center via http://www.ccdc.cam.ac.uk/structures.

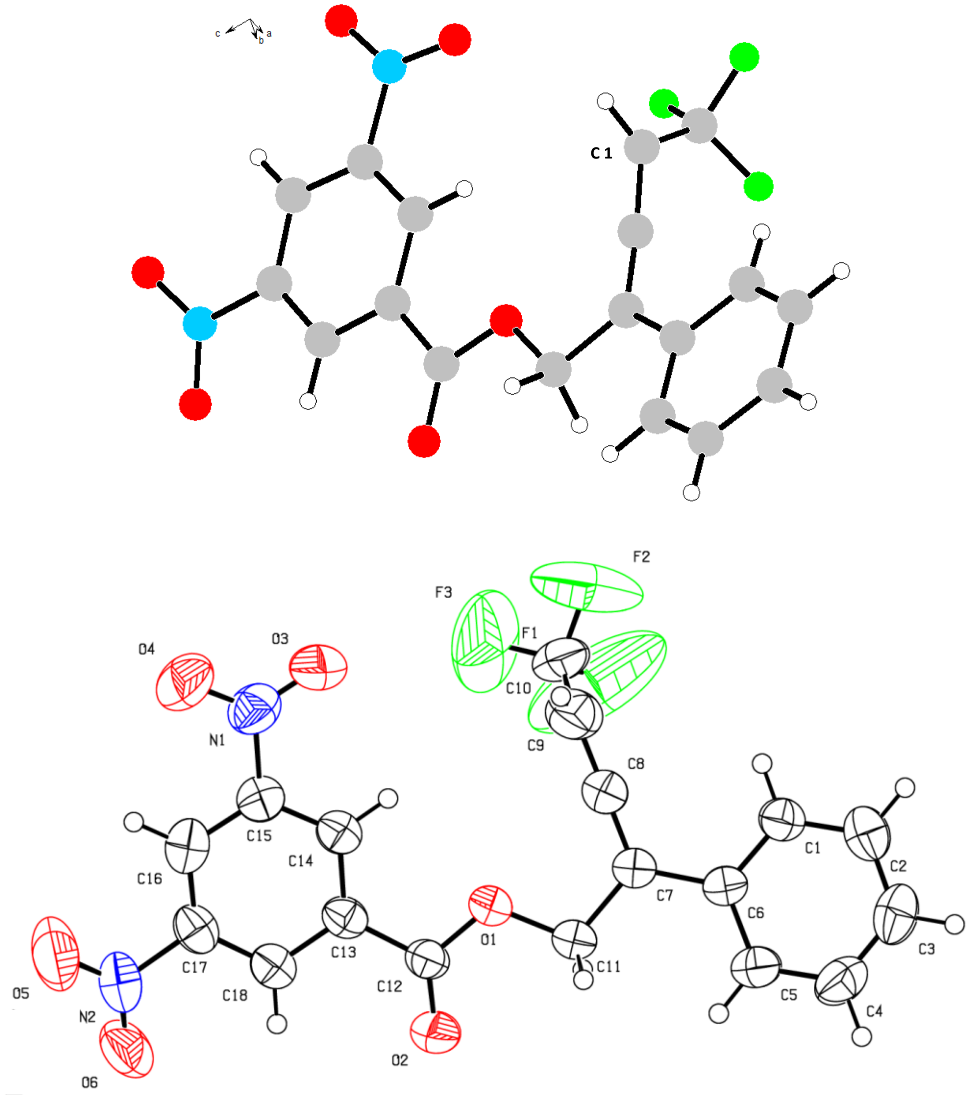

Figure S1. X-ray structure of compound (*R*)-**9**.

Table S2. Crystallographic data and refinement parameters for compound (*R*)-**9**.

Empirical formula	$C_{18}H_{11}F_3N_2O_6$
Formula weight	408.29 g mol^{-1}
Temperature	293 K
Wavelength	1.54178 Å
Crystal system	Monoclinic
Space group	$P2_1/n$ (No. 14)
Unit cell dimensions	a = 7.3147(2) Å<br>b = 12.7413(4) Å<br>c = 19.2524(6) Å<br>β = 90.027(2)°
Volume	1794.30(9) Å^3
Z	4
Density (calc.)	1.511 g cm^{-3}
Absorption coefficient	1.179 mm^{-1}
F(000)	832
Crystal size	0.21 $\times$ 0.29 $\times$ 0.53 mm^3
θ range for data collection	4.121 to 74.673°
Index ranges	$-9 \le h \le 8$<br>$-15 \le k \le 15$<br>$-23 \le l \le 24$
Reflections collected	17591
Independent reflections	3650<br>[R(int) = 0.0591]
Data / restr. / param.	3650/0/262
Goodness-pf-fit on F^2	1.079
Final R indices [I > 2σ(I)]	R1 = 0.1211,<br>wR2 = 0.3328
Largest diff. peak and hole	1.077 and -0.469 e Å^{-3}

Gram-scale preparation of trifluoromethyl allene <u>2e</u>:

Reaction procedure: In a dry 50 mL Schlenk flask under argon was added substrate **1e** (1.82 g, 5.0 mmol, 1.0 equiv), catalyst $Fe(acac)_3$ (88 mg, 0.25 mmol, 5 mol%) and anhydrous toluene (25 mL) to obtain an orange solution. The stirred solution was cooled to 0 °C and MeMgBr (2.50 mL, 3.0 M in Et_2O, 7.5 mmol, 1.5 equiv) was added dropwise (Figure S2). The reaction mixture was allowed to reach room temperature and stirred for an additional 30 minutes. The reaction was quenched with aq. 5% citric acid solution, extracted and purified over SiO_2 (eluent: ethyl acetate/pentane 1 - 10%) to yield 1.53 g (88%) of product **2e** as a pale-yellow oil.

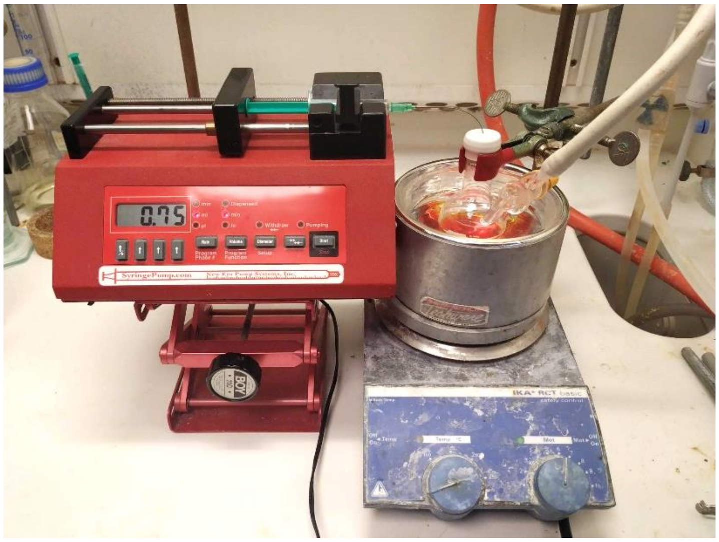

Figure S2. Reaction setup for gram-scale preparation of trifluoromethyl allene **2e.**

Palladium-catalyzed oxidative borylation of trifluoromethyl allene <u>2h</u>:[9]

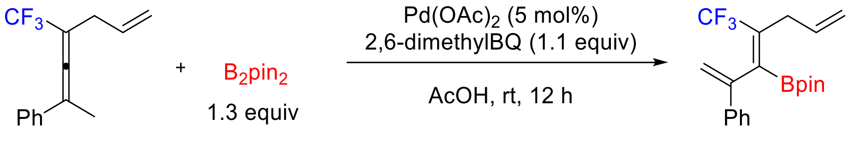

2h **8**

Reaction procedure: To a mixture of $Pd(OAc)_2$ (2.2 mg, 0.01 mmol), B_2pin_2 (66.0 mg, 0.26 mmol), and 2,6-dimethyl-BQ (30.0 mg, 0.22 mmol), was added a solution of allene **2h** (48 mg, 0.2 mmol) in AcOH (1 mL). The reaction was stirred at room temperature for 12 h. After full consumption of starting material **2h** (as monitored by TLC), the reaction mixture was concentrated in vacuo and

purified by column chromatography on SiO_2 (eluent: ethyl acetate/pentane 1 - 10%) to yield product **8** (49 mg, 67%) as a pale yellow oil.

(Z)-4,4,5,5-Tetramethyl-2-(2-phenyl-4-(trifluoromethyl)hepta-1,3,6-trien-3-yl)-1,3,2-dioxaborolane (**8**)

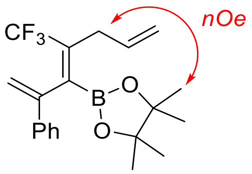

8

^1H NMR (500 MHz, $CDCl_3$) δ 7.41 – 7.36 (m, 2H), 7.33 – 7.28 (m, 2H), 7.28 – 7.23 (m, 1H), 5.92 (ddt, *J* = 16.7, 10.1, 6.5 Hz, 1H), 5.58 (d, *J* = 1.0 Hz, 1H), 5.20 (dq, *J* = 17.1, 1.6 Hz, 1H), 5.15 – 5.09 (m, 2H), 3.30 (dt, *J* = 6.3, 1.6 Hz, 2H), 1.10 (s, 12H); ^{13}C NMR (126 MHz, $CDCl_3$) δ 146.2, 139.0, 137.8 (q, *J* = 26.5 Hz), 135.5, 128.3 (2C), 127.9, 126.8 (2C), 123.8 (q, *J* = 278.9 Hz), 116.9, 112.8 (q, *J* = 2.9 Hz), 84.4 (2C), 35.7 (q, *J* = 2.2 Hz), 24.5 (4C), the signal corresponding to carbon attached to the boron atom was not detected; ^{19}F NMR (377 MHz, $CDCl_3$) δ -58.01; HRMS (ESI): calc. for $C_{20}H_{24}BF_3O_2Na$ $[M+Na]^+$: 387.1717; found: 387.1722.

Mechanistic experiments

Reaction in the presence of TEMPO:

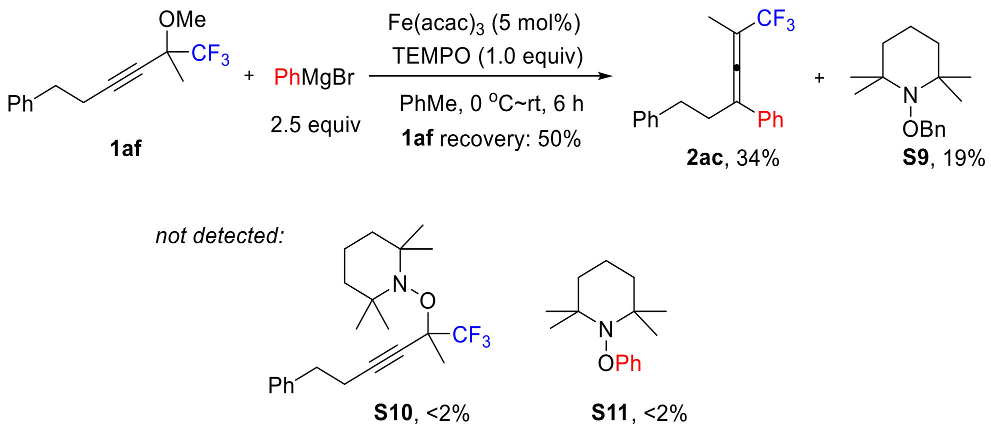

Reaction procedure: In a dry 5 mL microwave vial under argon was added substrate **1af** (77 mg,

0.3 mmol, 1.0 equiv), catalyst Fe(acac)$_3$ (5.3 mg, 0.015 mmol, 5 mol%), TEMPO (47 mg, 0.3 mmol, 1.0 equiv) and anhydrous toluene (1.5 mL, 0.2 M). The stirred solution was cooled to 0 °C and PhMgBr (250 µL, 3.0 M in Et_2O, 0.75 mmol, 2.5 equiv) was added dropwise. The reaction mixture was allowed to reach the room temperature and stirred for additional 6 hours. Reaction mixture was filtered through a plug of Celite over silica (1:5) with Et_2O (5 x 4 mL) and concentrated under reduced pressure. The crude residue was analyzed by NMR using anisole (0.3 mmol, 1.0 equiv) as the internal standard:

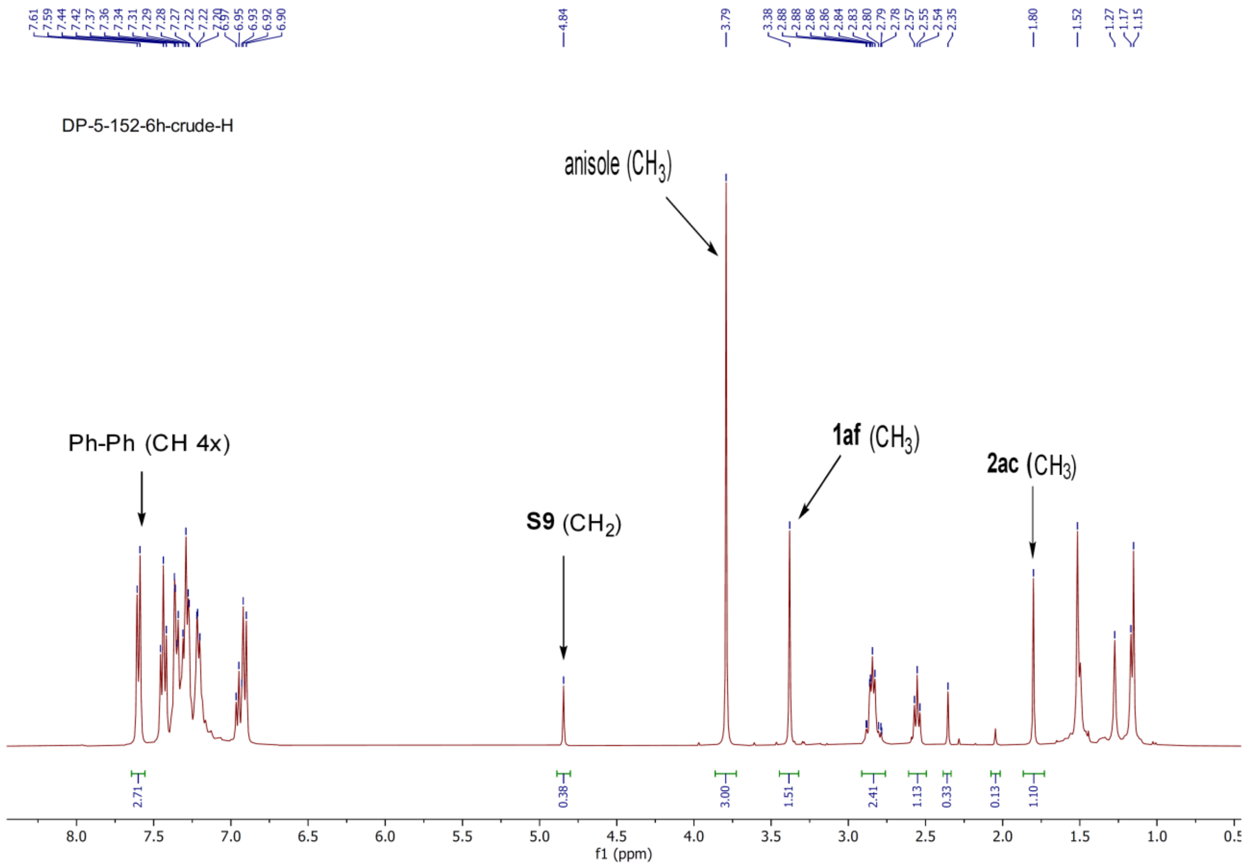

1-(Benzyloxy)-2,2,6,6-tetramethylpiperidine (**S9**)

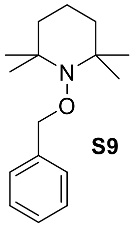

S9

^{1}H NMR (400 MHz, $CDCl_3$) δ 7.38 – 7.27 (m, 5H), 4.84 (s, 2H), 1.70 – 1.49 (m, 6H), 1.27 (s, 6H), 1.17 (s, 6H). NMR data is in accordance with the literature[10]; HRMS (ESI): calc. for $C_{16}H_{25}NNaO$ $[M+Na]^+$: 270.1828; found: 270.1830.

Analysis of the crude reaction mixture in the preparation of ***2ob****:*

Based on the suggestion by one reviewer, reaction of the substrate **1o** with EtMgBr was investigated in closer detail. The reviewer was wondering if any side products could be observed in this reaction arising from hydromagnesiation of the alkyne moiety in **1o**, since the reaction conditions applied in this work bear similarity to those described previously by others in hydromagnesiation reactions of alkenes and alkynes.[11] As can be seen from the ^{1}H-NMR of the crude reaction mixture (see below, using anisole as the internal standard), under the standard reaction conditions the desired allene product **2ob** was obtained in 46% NMR yield as the main product (46% isolated yield) with formation of multiple unidentified side products (each in <5% yield).

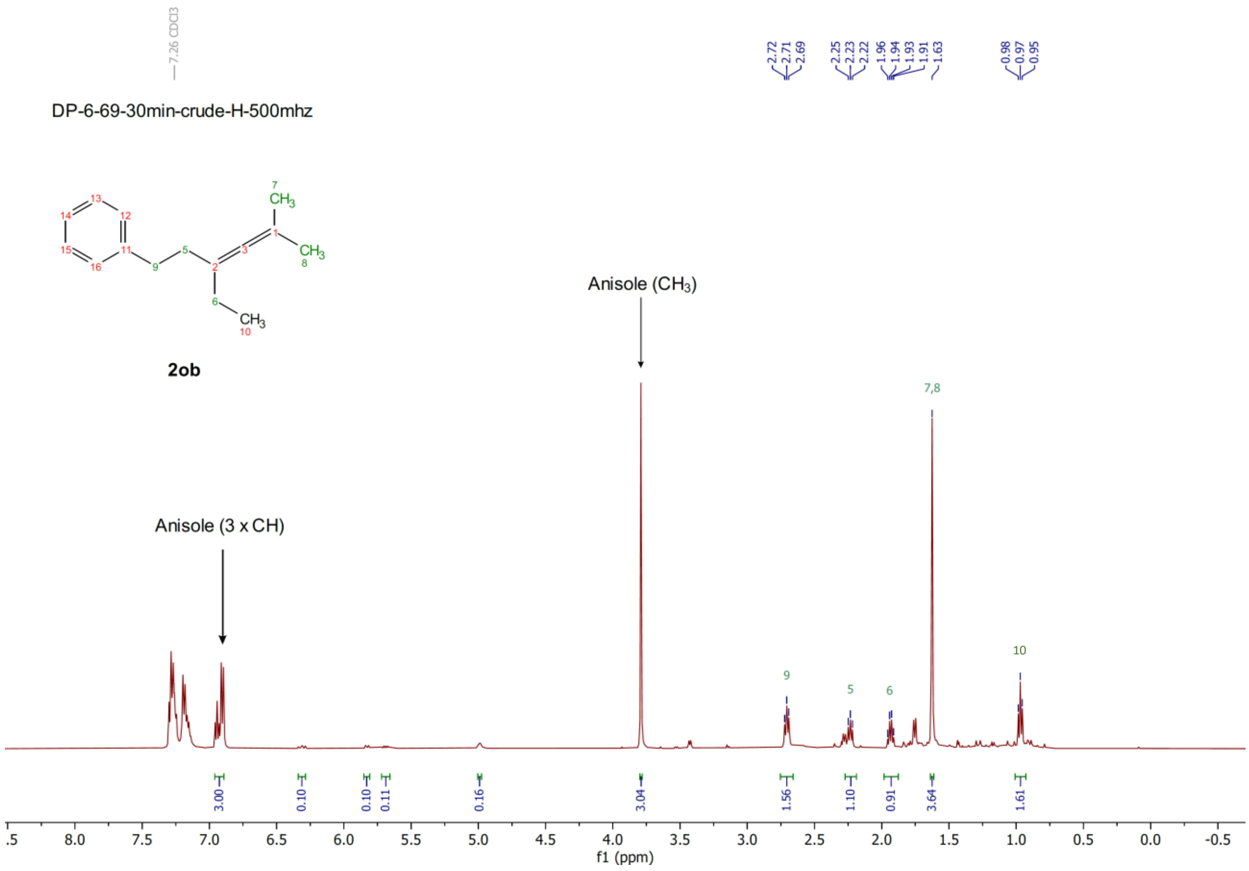

3. References:

[1] Krasovskiy, A.; Knochel, P. *Synthesis* **2006**, 890.

[2] Zhang, Y. -M.; Yuan, M. -L.; Liu, W. -P.; Xie, J. -H.; Zhou, Q. -L. *Org. Lett.* **2018**, *20*, 4486.

[3] Trost, B. M.; Hung, C. -I.; Scharf, M. J. *Angew.Chem., Int.Ed.* **2018**, *57*, 11408.

[4] Gerleve, C.; Studer, A.; *Angew. Chem. Int. Ed*. **2020**, *59*, 15468.

[5] Ying, J.; Le, Z.; Wu, X.-F. *Org. Lett.* **2020**, *22*, 194.

[6] Doucet-Personeni, C.; Bentley, P. D.; Fletcher, R. J.; Kinkaid, A.; Kryger, G.; Pirard, B.; Taylor, A.; Taylor, R.; Taylor, J.; Viner, R.; Silman, I.; Sussman, J. L.; Greenblatt, H. M.; Terence Lewis, T. *J. Med. Chem.* **2001**, *44*, 3203.

[7] Ko, S.-J.; Lim, J. Y.; Jeon, N. Y.; Won, K.; Ha, D.-C.; Kim, B. T.; Lee, H. *Tetrahedron: Asymmetry*, **2009**, *20*, 1109.

[8] Sheldrick, G. M. A Short History of SHELX. *Acta Crystallogr., Sect. A: Found. Crystallogr*., **2008**, *64*, 112.

[9] Qiu, Y.; Yang, B.; Zhu, C.; Bäckvall, J.-E. *Angew. Chem., Int. Ed.* **2016**, *55*, 6520.

[10] Yasu, Y.; Koike, T.; Akita, M. *Adv. Synth. Catal.* **2012**, *354*, 3414.

[11] (a) Shirakawa, E.; Ikeda, D.; Masui, S.; Yoshida, M.; Hayashi, T. *J. Am. Chem. Soc*., **2012**, *134*, 272. (b) Iles, L.; Yoshida, T.; Nakamura, E. *J. Am. Chem. Soc*., **2012**, *134*, 16951. (c) Greenhalgh M. D.; Thomas, S. P. *J. Am. Chem. Soc.*, **2012**, *134*, 11903.

4. ^{1}H and ^{13}C NMR spectra of compounds 1aa – 1s:

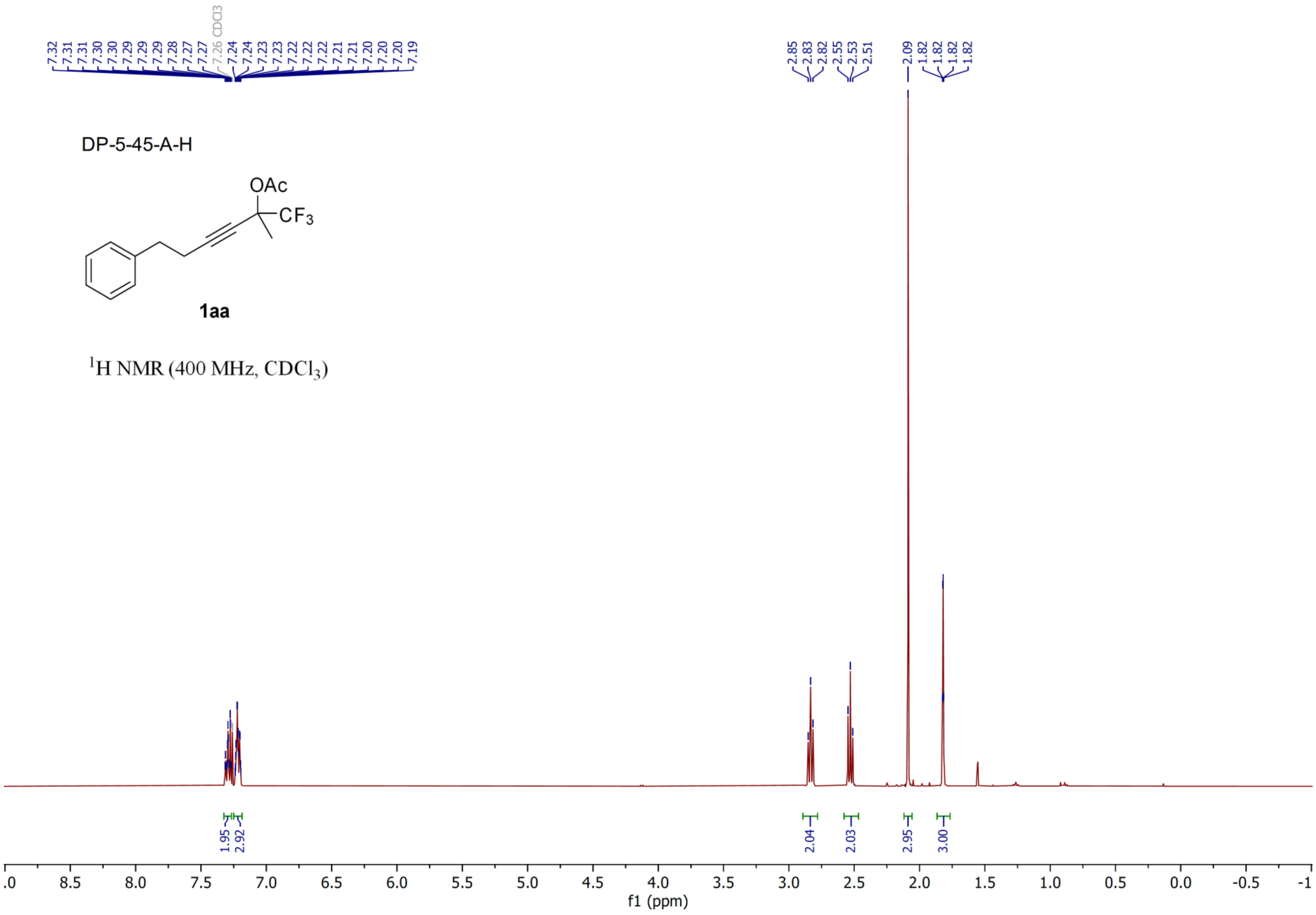

DP-5-45-A-C

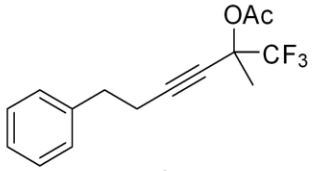

1aa

^{13}C NMR (101 MHz, $CDCl_3$)

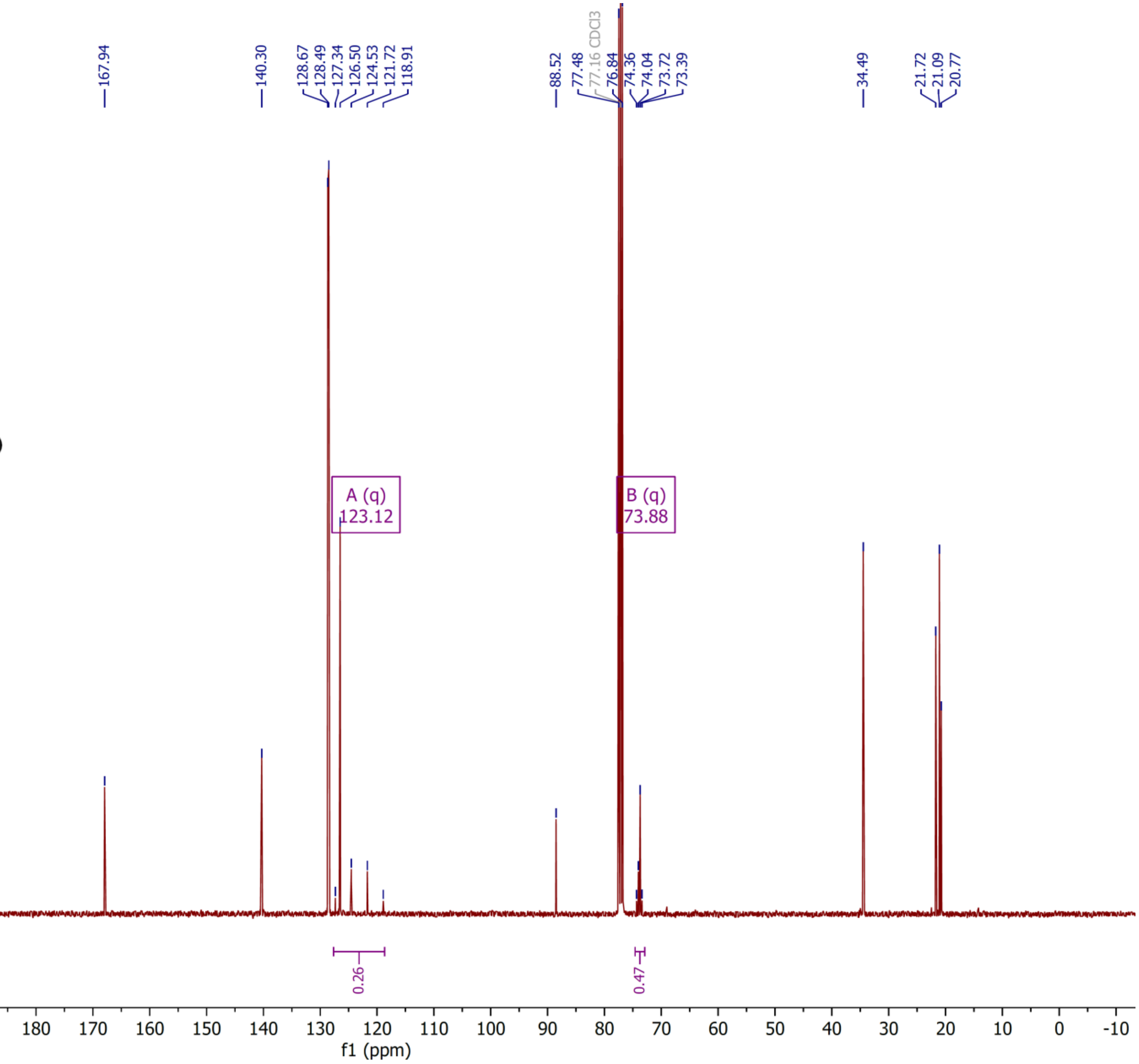

DP-5-45-A-F

1aa

^{19}F NMR (377 MHz, $CDCl_3$)

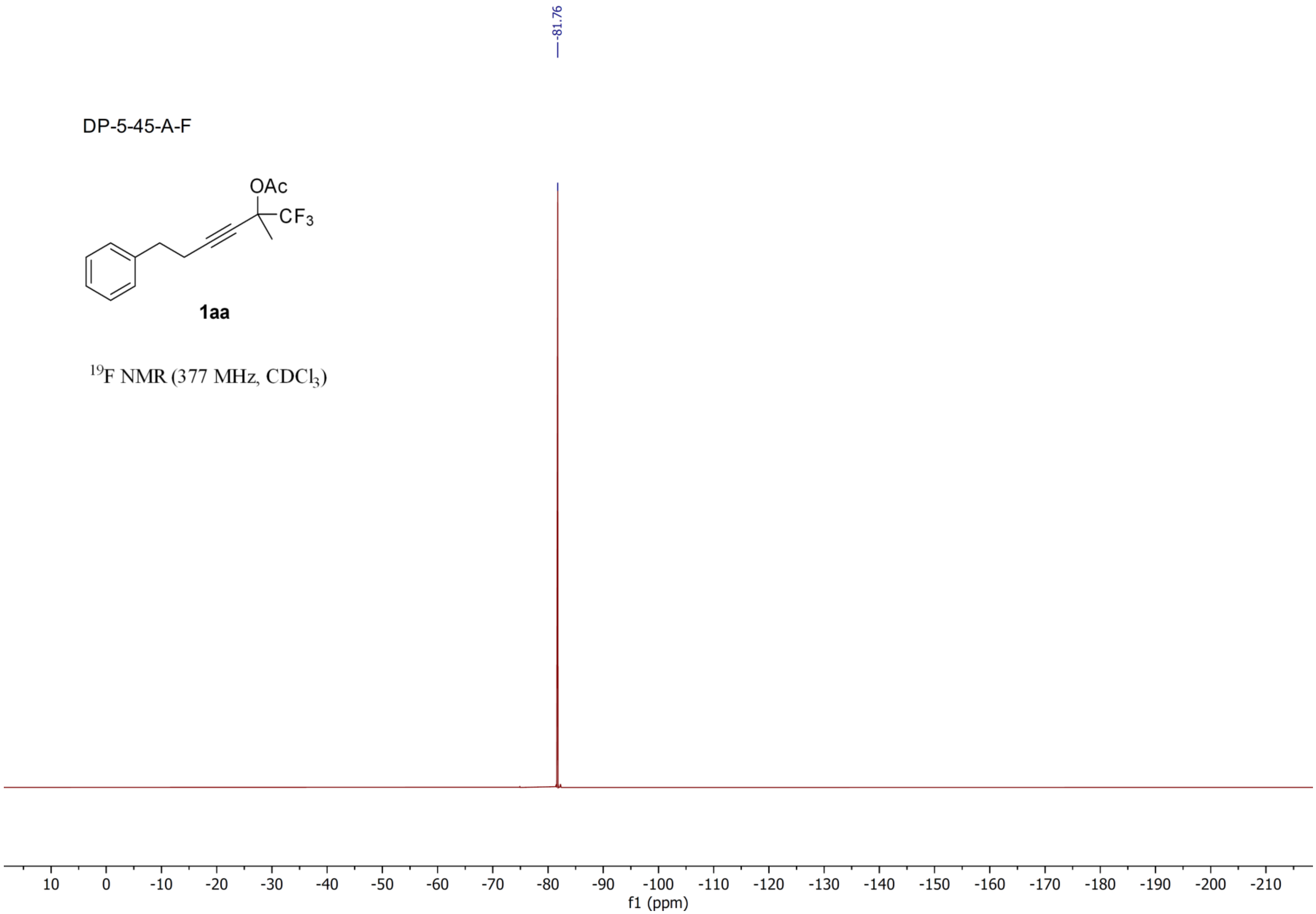

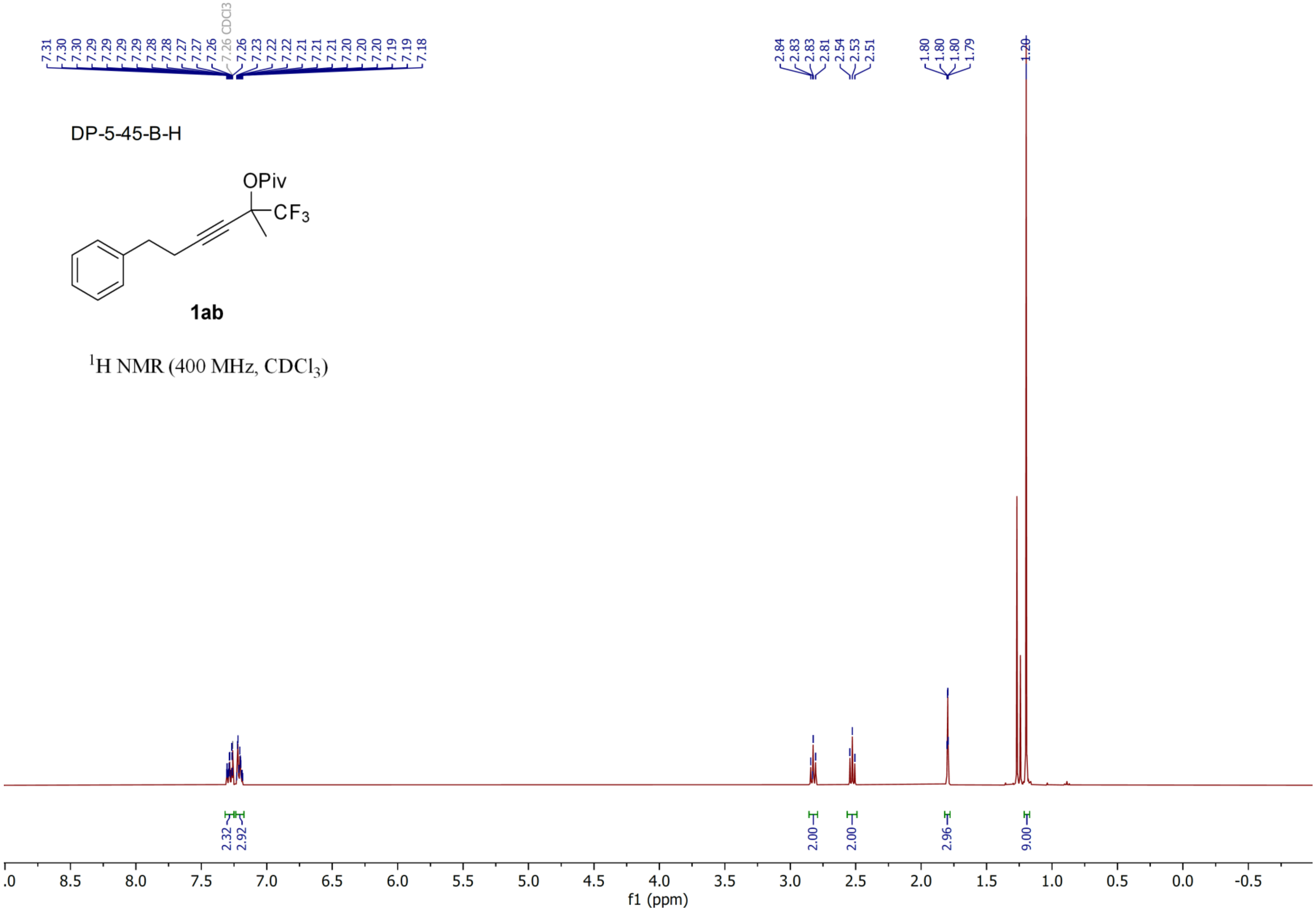
7.31 7.30 7.30 7.29 7.29 7.29 7.29 7.28 7.28 7.27 7.27 7.26 7.26 CDCl3 7.26 7.23 7.22 7.22 7.21 7.21 7.21 7.20 7.20 7.20 7.19 7.19 7.18
2.84 2.83 2.83 2.81 2.54 2.53 2.51
1.80 1.80 1.80 1.79
1.20
DP-5-45-B-H
OPiv
CF3
1ab
1H NMR (400 MHz, CDCl3)
2.32 2.92 2.00 2.00 2.96 9.00
.0 8.5 8.0 7.5 7.0 6.5 6.0 5.5 5.0 4.5 4.0 3.5 3.0 2.5 2.0 1.5 1.0 0.5 0.0 -0.5
f1 (ppm)

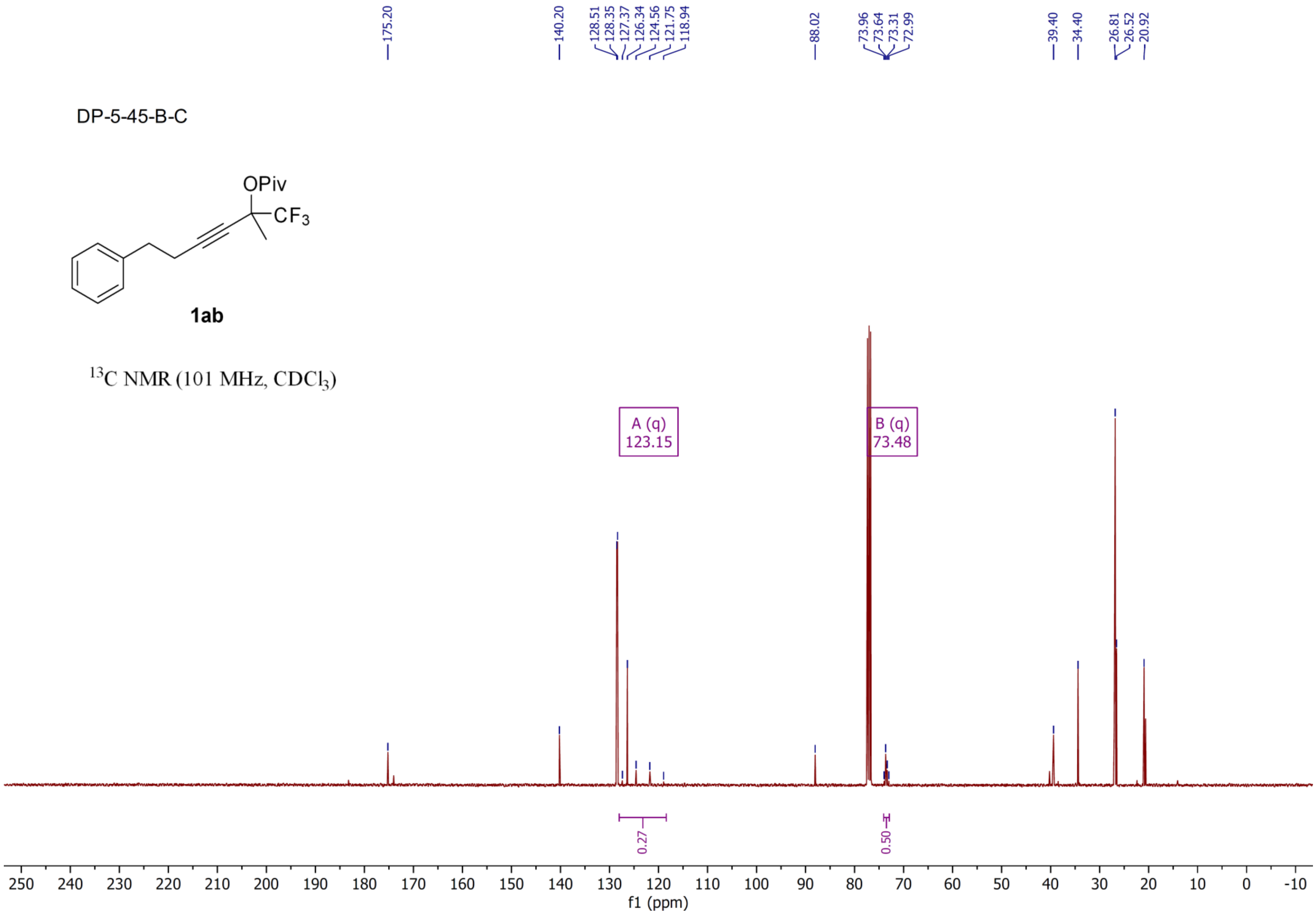
175.20
140.20
128.51
128.35
127.37
126.34
124.56
121.75
118.94
88.02
73.96
73.64
73.31
72.99
39.40
34.40
26.81
26.52
20.92
DP-5-45-B-C
OPiv
CF3
1ab
13C NMR (101 MHz, CDCl3)
A (q)
123.15
B (q)
73.48
0.27
0.50
f1 (ppm)

DP-5-45-B-F

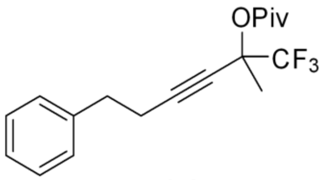

1ab

^{19}F NMR (377 MHz, $CDCl_3$)

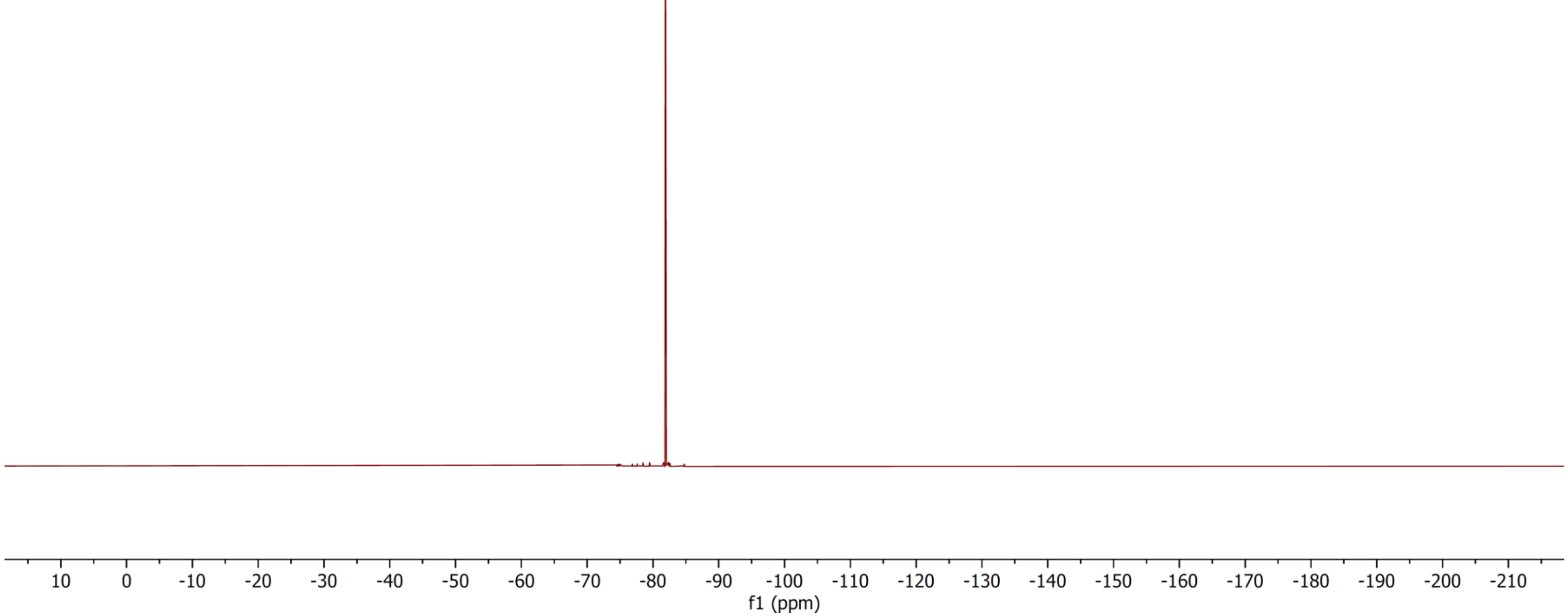

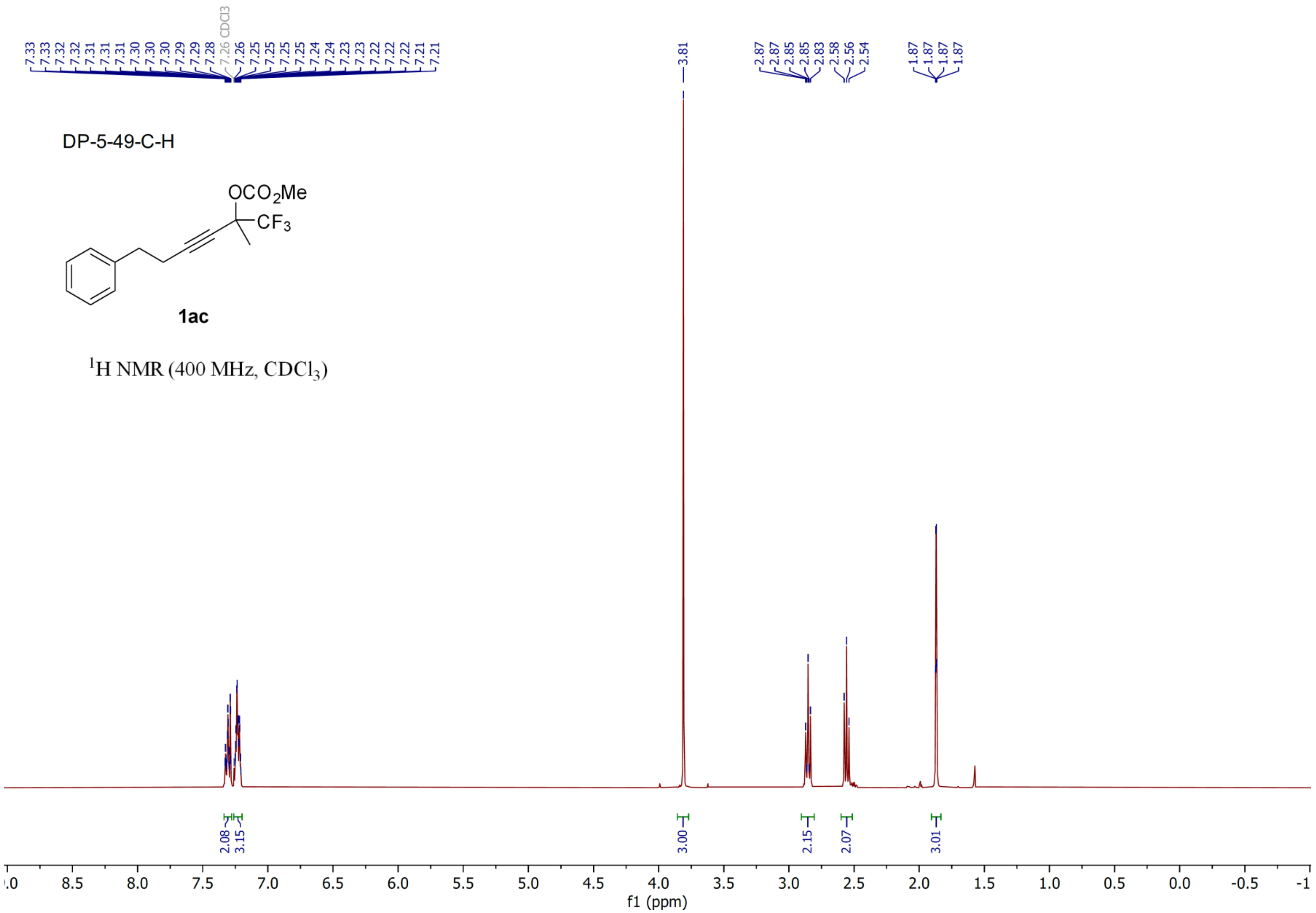

DP-5-49-C-H
OCO2Me
CF3
1ac
1H NMR (400 MHz, CDCl3)
7.33 7.33 7.32 7.32 7.31 7.31 7.31 7.30 7.30 7.30 7.29 7.29 7.28 7.26 CDCl3 7.26 7.25 7.25 7.25 7.25 7.25 7.24 7.24 7.23 7.23 7.22 7.22 7.22 7.21 7.21
3.81
2.87 2.87 2.85 2.85 2.83 2.58 2.56 2.54
1.87 1.87 1.87 1.87
2.08 3.15 3.00 2.15 2.07 3.01
f1 (ppm)

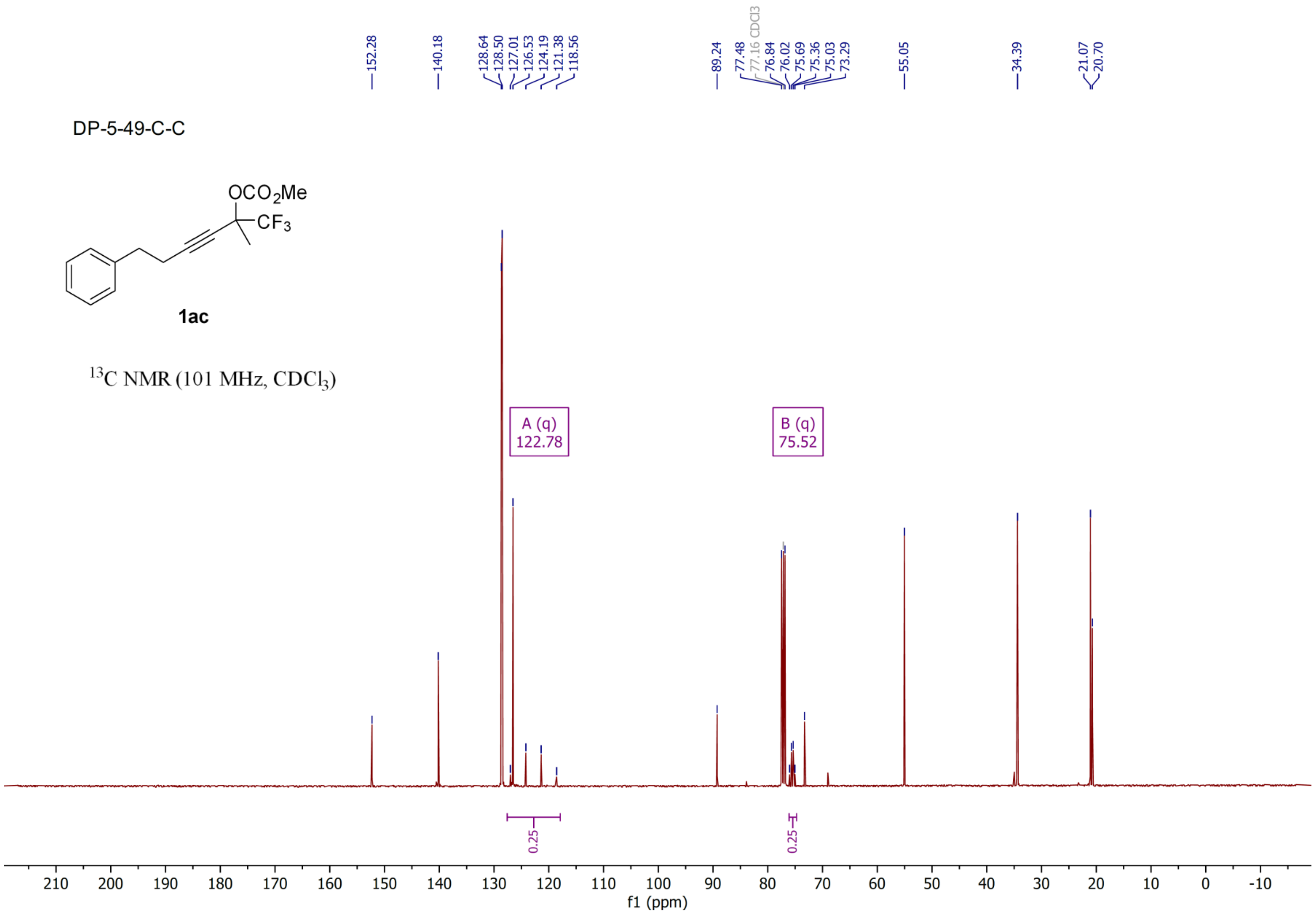
152.28
140.18
128.64
128.50
127.01
126.53
124.19
121.38
118.56
89.24
77.48
77.16 CDCl3
76.84
76.02
75.69
75.36
75.03
73.29
55.05
34.39
21.07
20.70
DP-5-49-C-C
OCO2Me
CF3
1ac
13C NMR (101 MHz, CDCl3)
A (q)
122.78
B (q)
75.52
0.25
0.25
210
200
190
180
170
160
150
140
130
120
110
100
90
80
70
60
50
40
30
20
10
0
-10
f1 (ppm)

DP-5-49-C-F

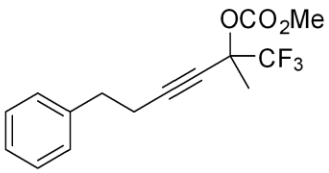

1ac

^{19}F NMR (377 MHz, $CDCl_3$)

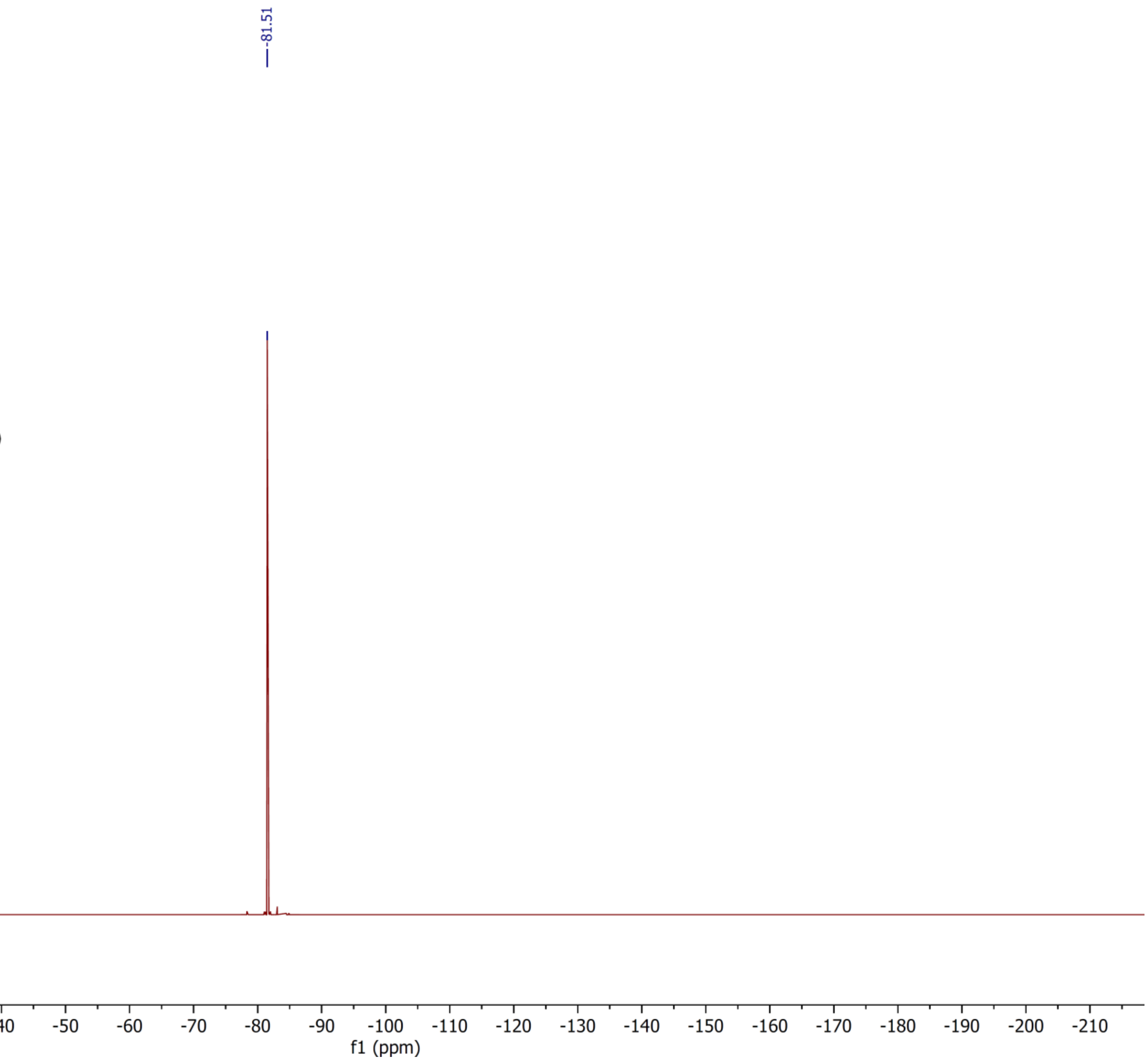

DP-5-49-A-H

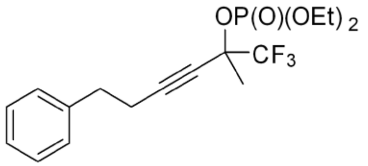

1ad

^{1}H NMR (400 MHz, $CDCl_3$)

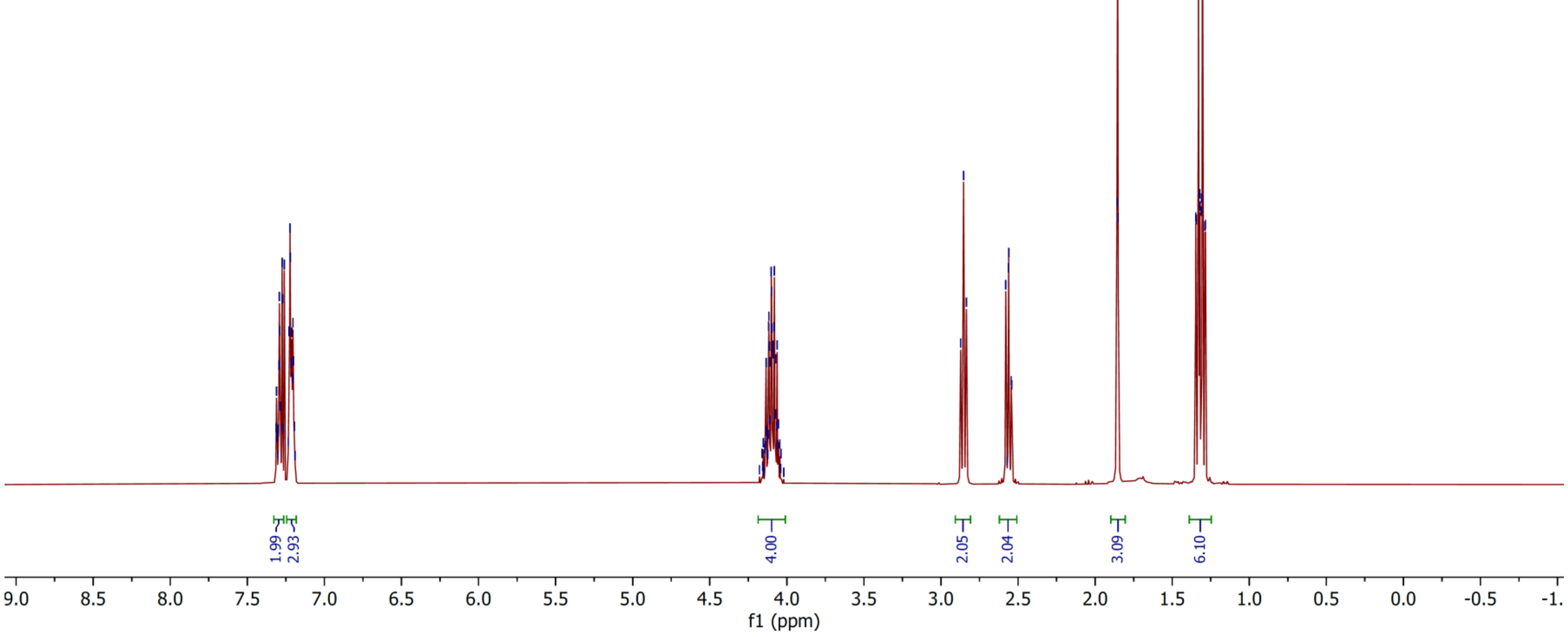

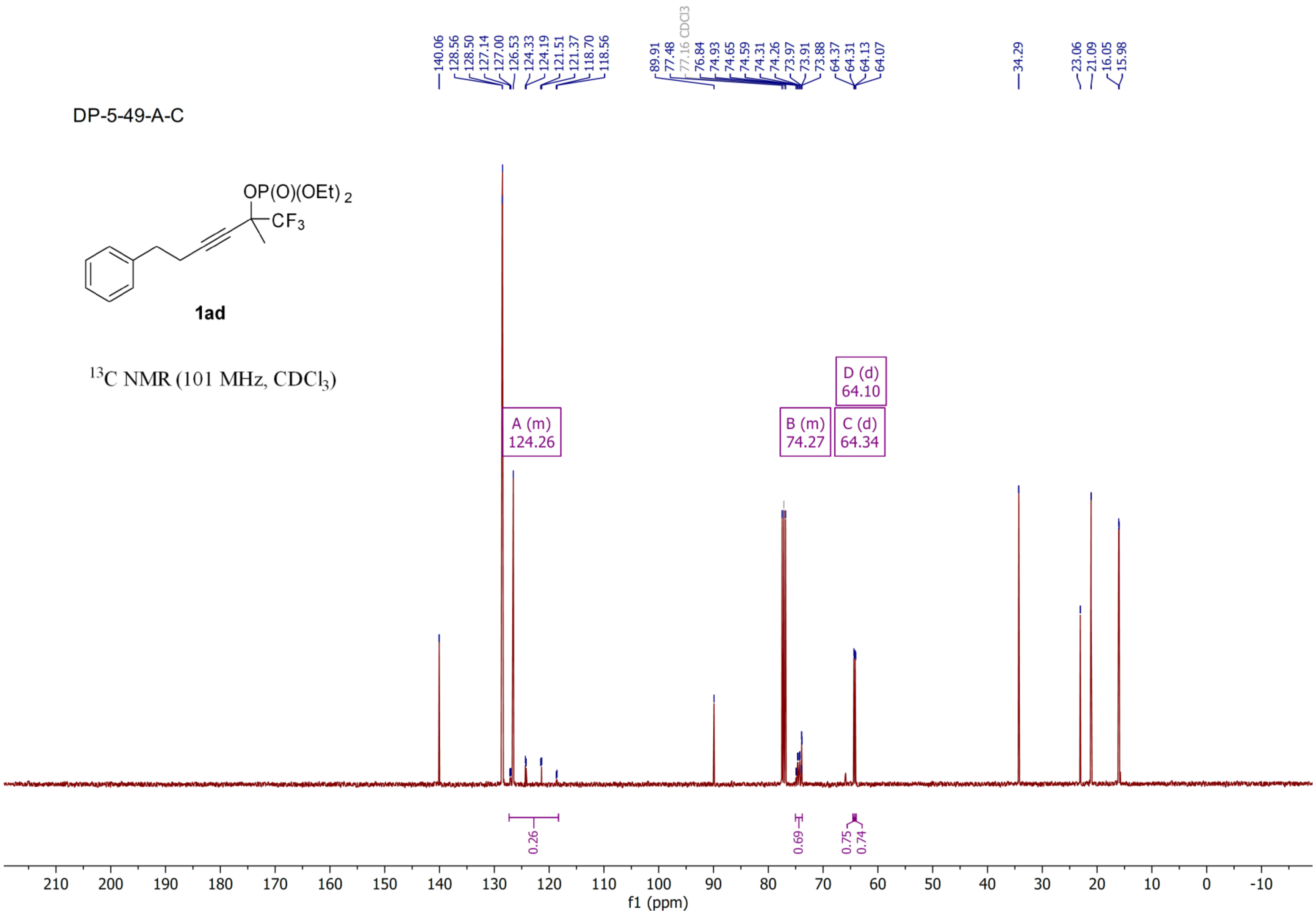
DP-5-49-A-C
OP(O)(OEt)2
CF3
1ad
13C NMR (101 MHz, CDCl3)
140.06
128.56
128.50
127.14
127.00
126.53
124.33
124.19
121.51
121.37
118.70
118.56
89.91
77.48
77.16 CDCl3
76.84
74.93
74.65
74.59
74.31
74.26
73.97
73.91
73.88
64.37
64.31
64.13
64.07
34.29
23.06
21.09
16.05
15.98
A (m)
124.26
B (m)
74.27
C (d)
64.34
D (d)
64.10
0.26
0.69
0.75
0.74
f1 (ppm)

DP-5-49-A-F

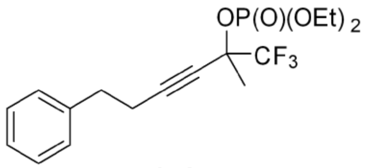

1ad

^{19}F NMR (377 MHz, $CDCl_3$)

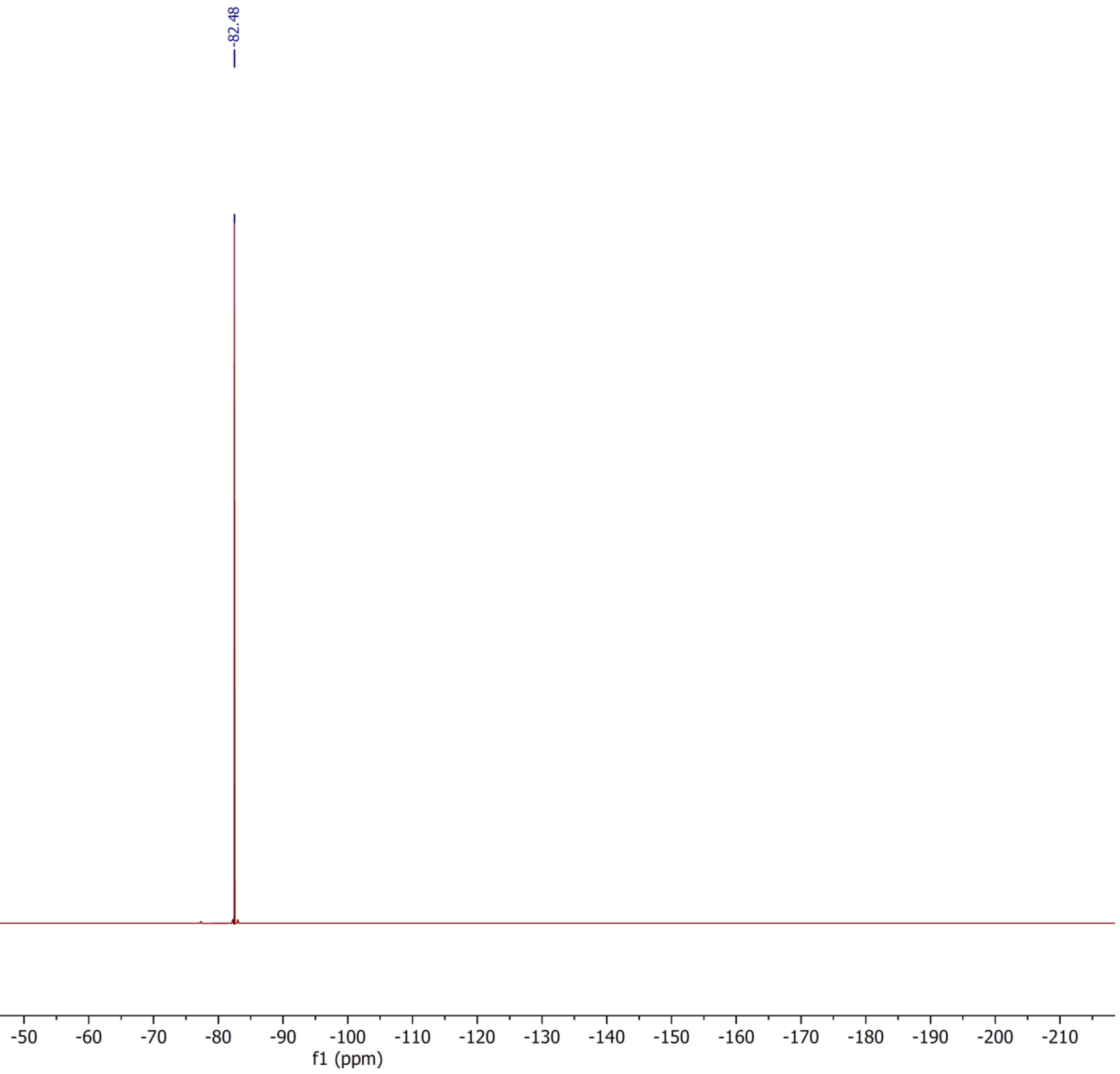

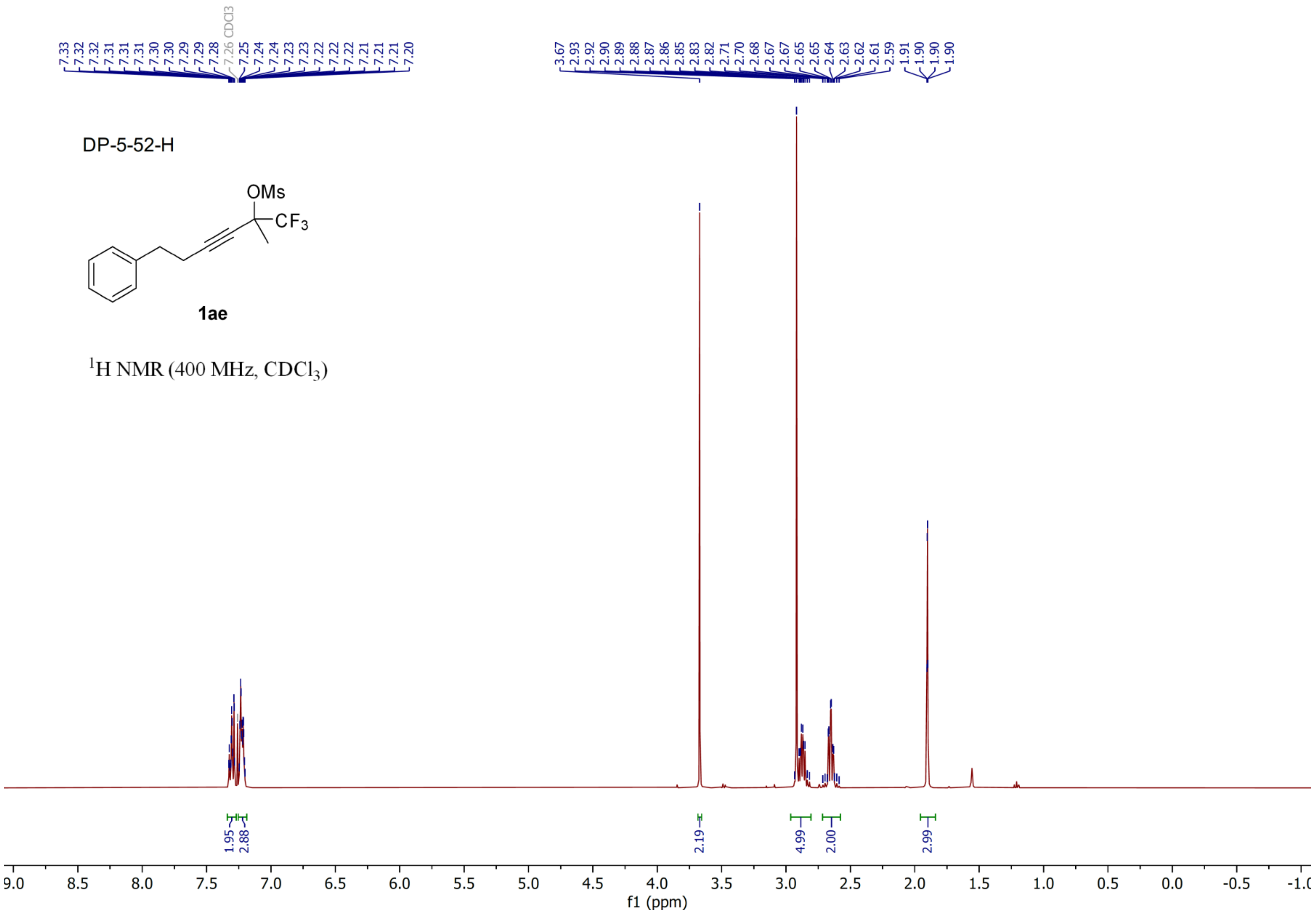

7.33
7.32
7.32
7.31
7.31
7.31
7.30
7.30
7.29
7.29
7.28
7.26 CDCl3
7.25
7.24
7.24
7.23
7.23
7.22
7.22
7.22
7.21
7.21
7.21
7.20
3.67
2.93
2.92
2.90
2.89
2.88
2.87
2.86
2.85
2.83
2.82
2.71
2.70
2.68
2.67
2.67
2.65
2.65
2.64
2.63
2.62
2.61
2.59
1.91
1.90
1.90
1.90
DP-5-52-H
OMs
CF3
1ae
1H NMR (400 MHz, CDCl3)
1.95
2.88
2.19
4.99
2.00
2.99
9.0
8.5
8.0
7.5
7.0
6.5
6.0
5.5
5.0
4.5
4.0
3.5
3.0
2.5
2.0
1.5
1.0
0.5
0.0
-0.5
f1 (ppm)

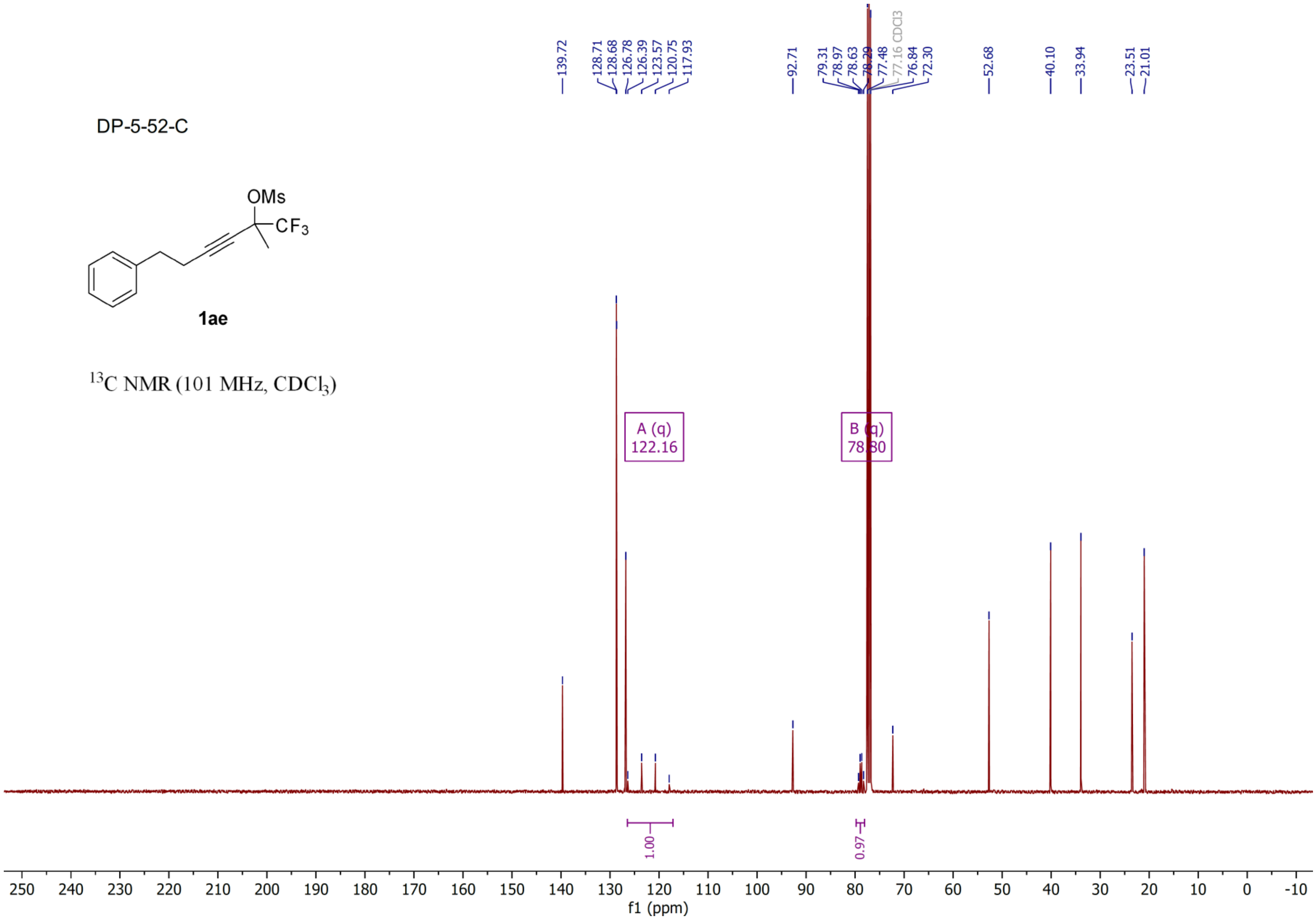
DP-5-52-C
OMs
CF3
1ae
13C NMR (101 MHz, CDCl3)
139.72
128.71
128.68
126.78
126.39
123.57
120.75
117.93
92.71
79.31
78.97
78.63
78.29
77.48
77.16 CDCl3
76.84
72.30
52.68
40.10
33.94
23.51
21.01
A (q)
122.16
B (q)
78.80
1.00
0.97
f1 (ppm)

DP-5-52-F

1ae

^{19}F NMR (377 MHz, CDCl$_3$)

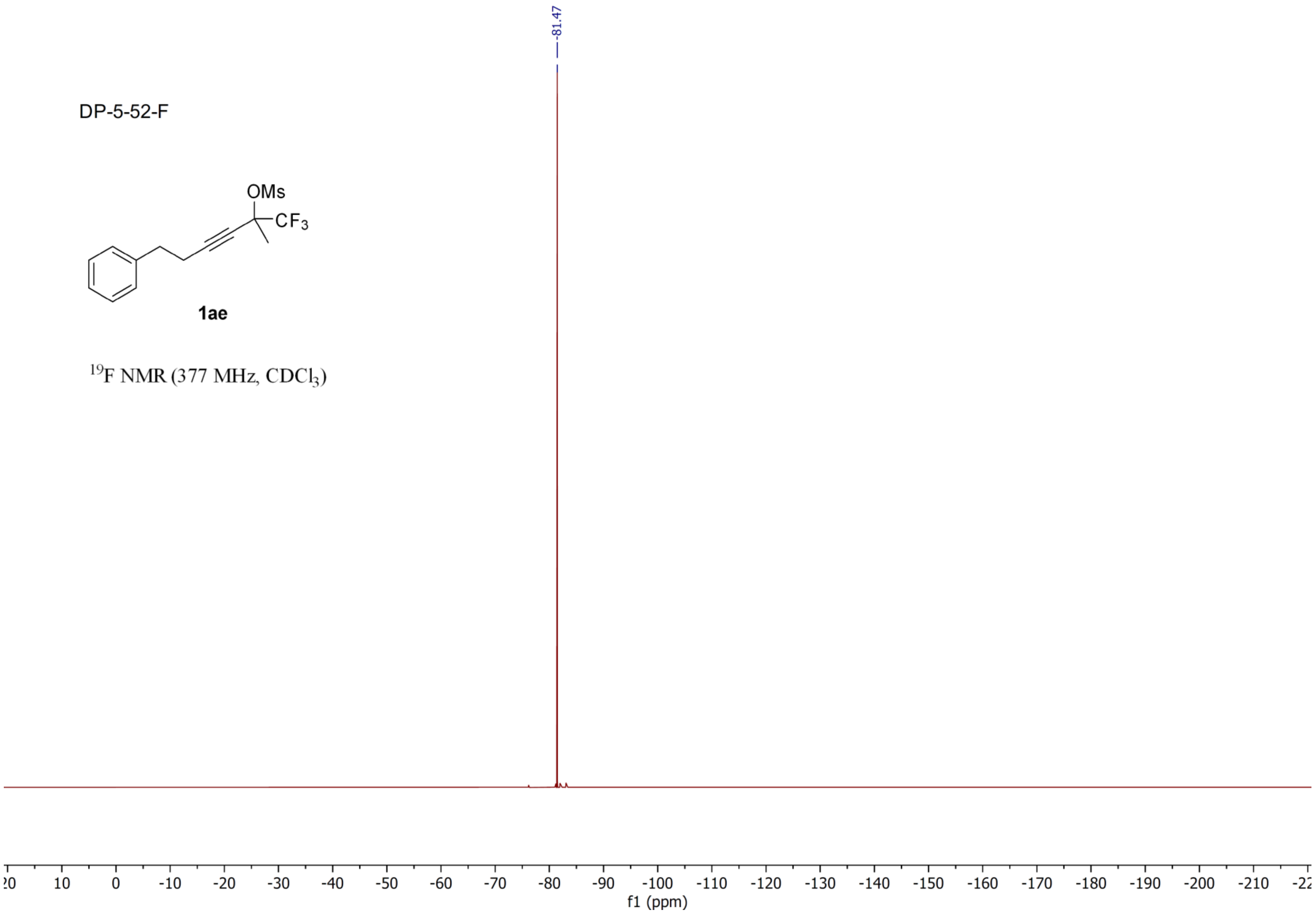

DP-5-4-H

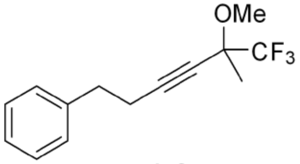

1af

^{1}H NMR (400 MHz, $CDCl_3$)

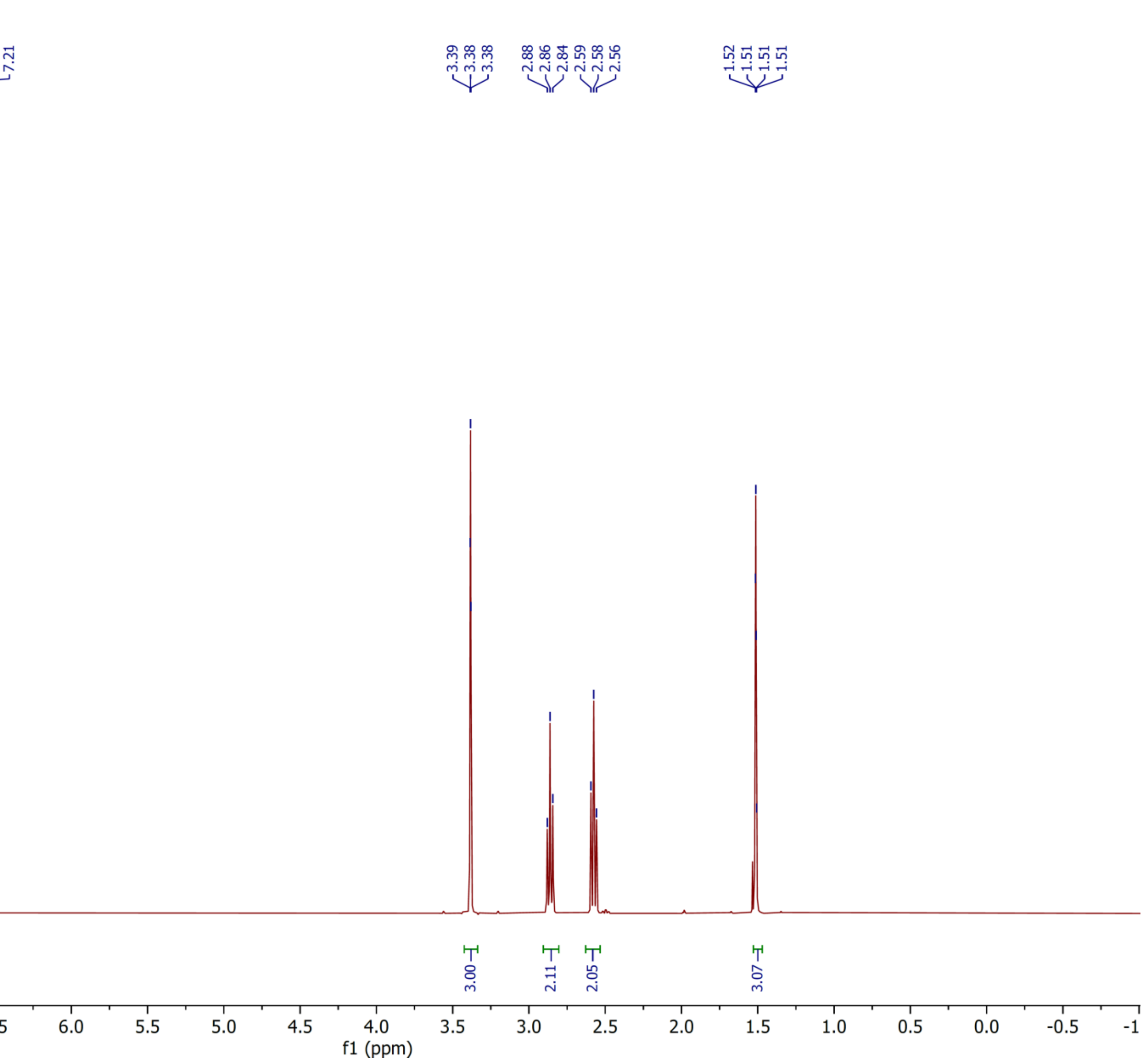

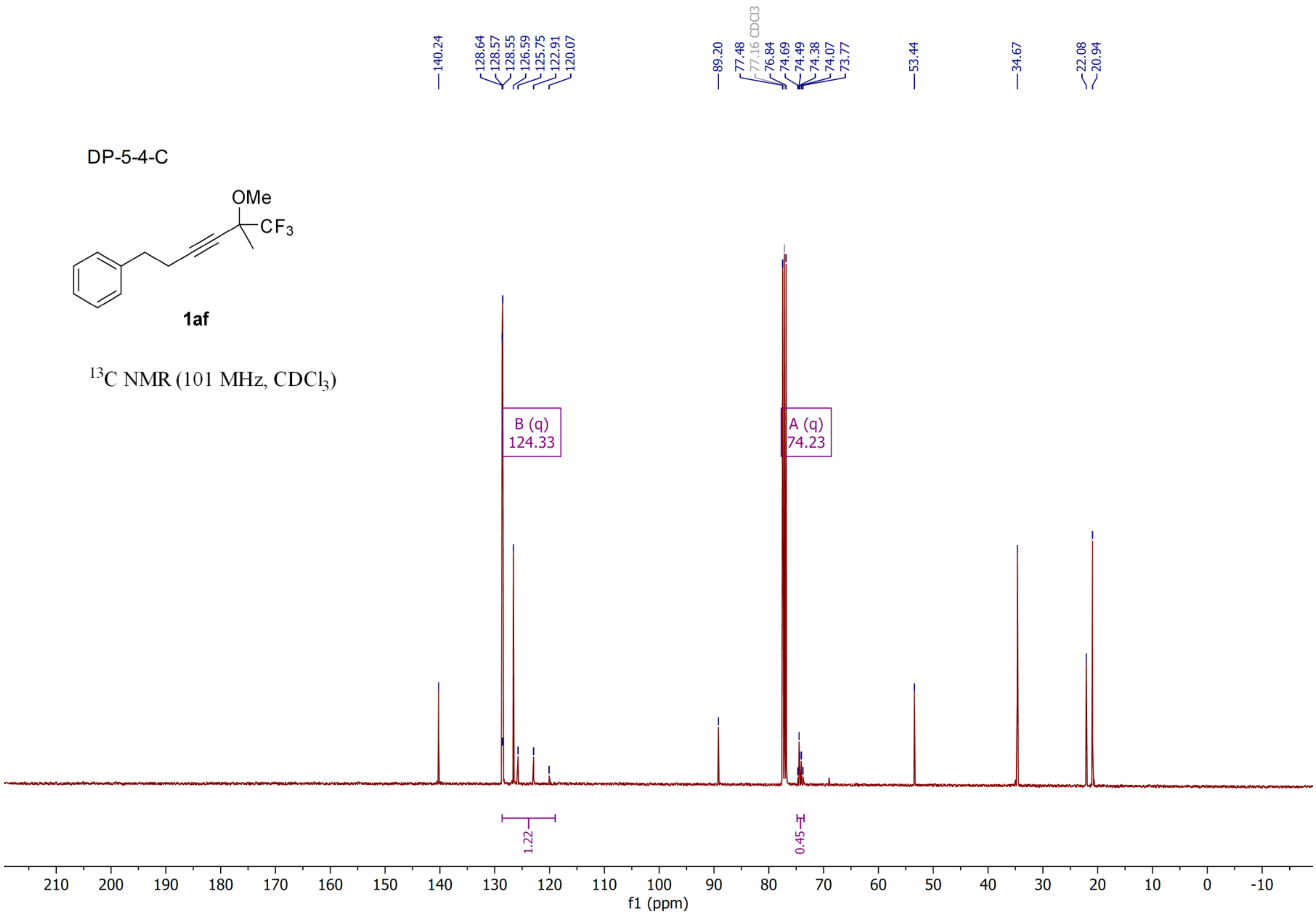
140.24
128.64
128.57
128.55
126.59
125.75
122.91
120.07
89.20
77.48
77.16 CDCl3
76.84
74.69
74.49
74.38
74.07
73.77
53.44
34.67
22.08
20.94
DP-5-4-C
OMe
CF3
1af
13C NMR (101 MHz, CDCl3)
B (q)
124.33
A (q)
74.23
1.22
0.45
210
200
190
180
170
160
150
140
130
120
110
100
90
80
70
60
50
40
30
20
10
0
-10
f1 (ppm)

DP-5-4-F

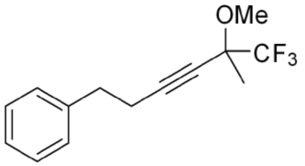

1af

^{19}F NMR (377 MHz, $CDCl_3$)

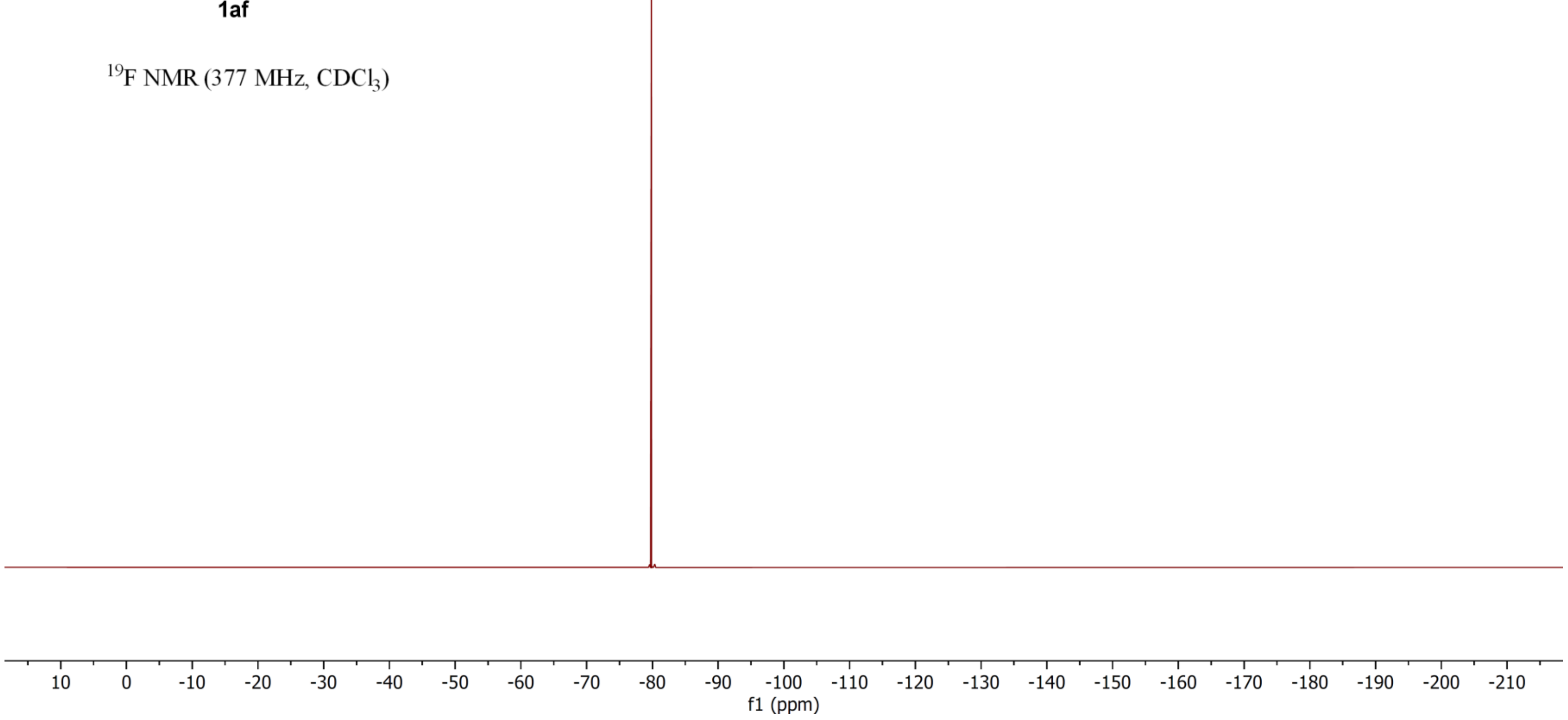

DP-5-7-H

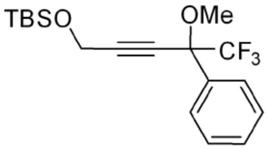

1b

^{1}H NMR (400 MHz, $CDCl_3$)

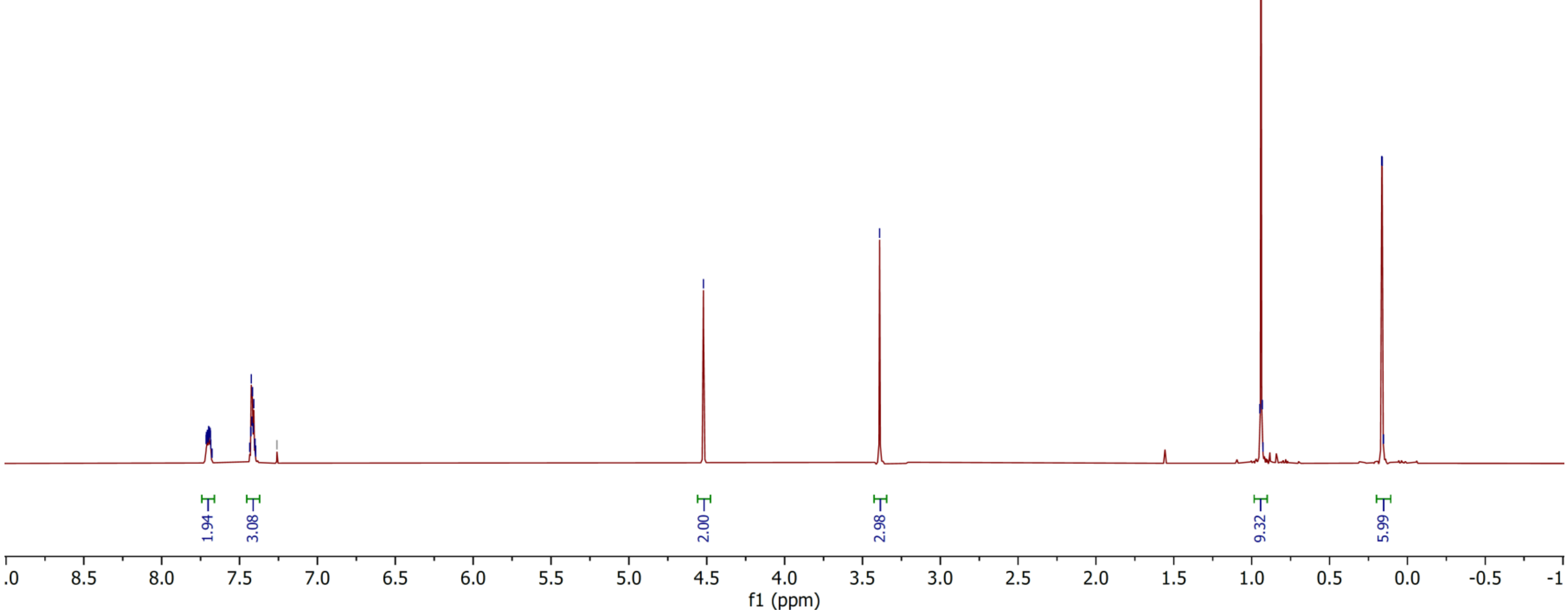

DP-5-7-C

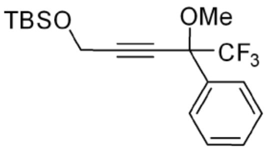

1b

^{13}C NMR (101 MHz, $CDCl_3$)

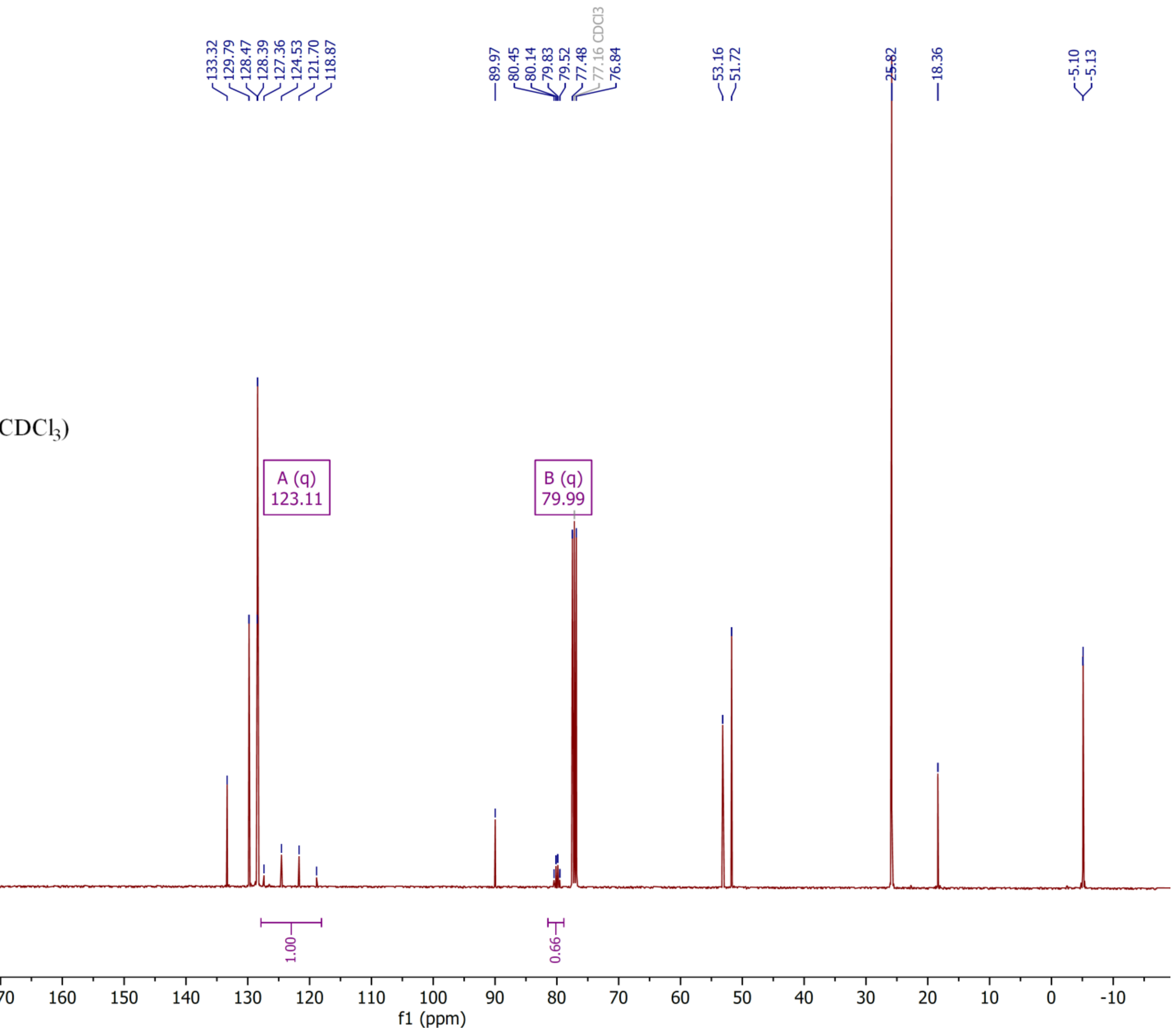

DP-5-7-F

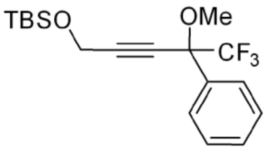

1b

^{19}F NMR (377 MHz, $CDCl_3$)

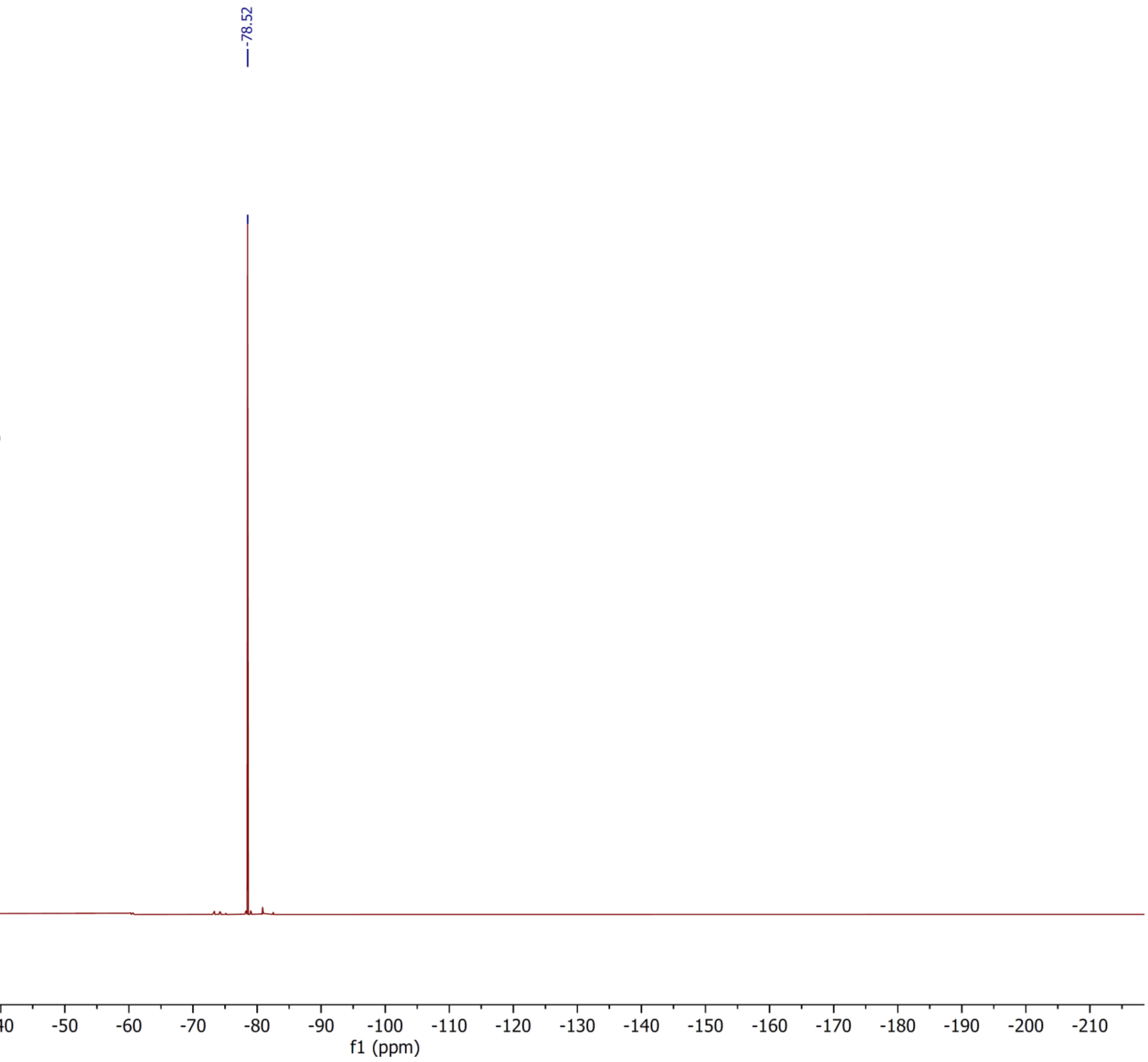

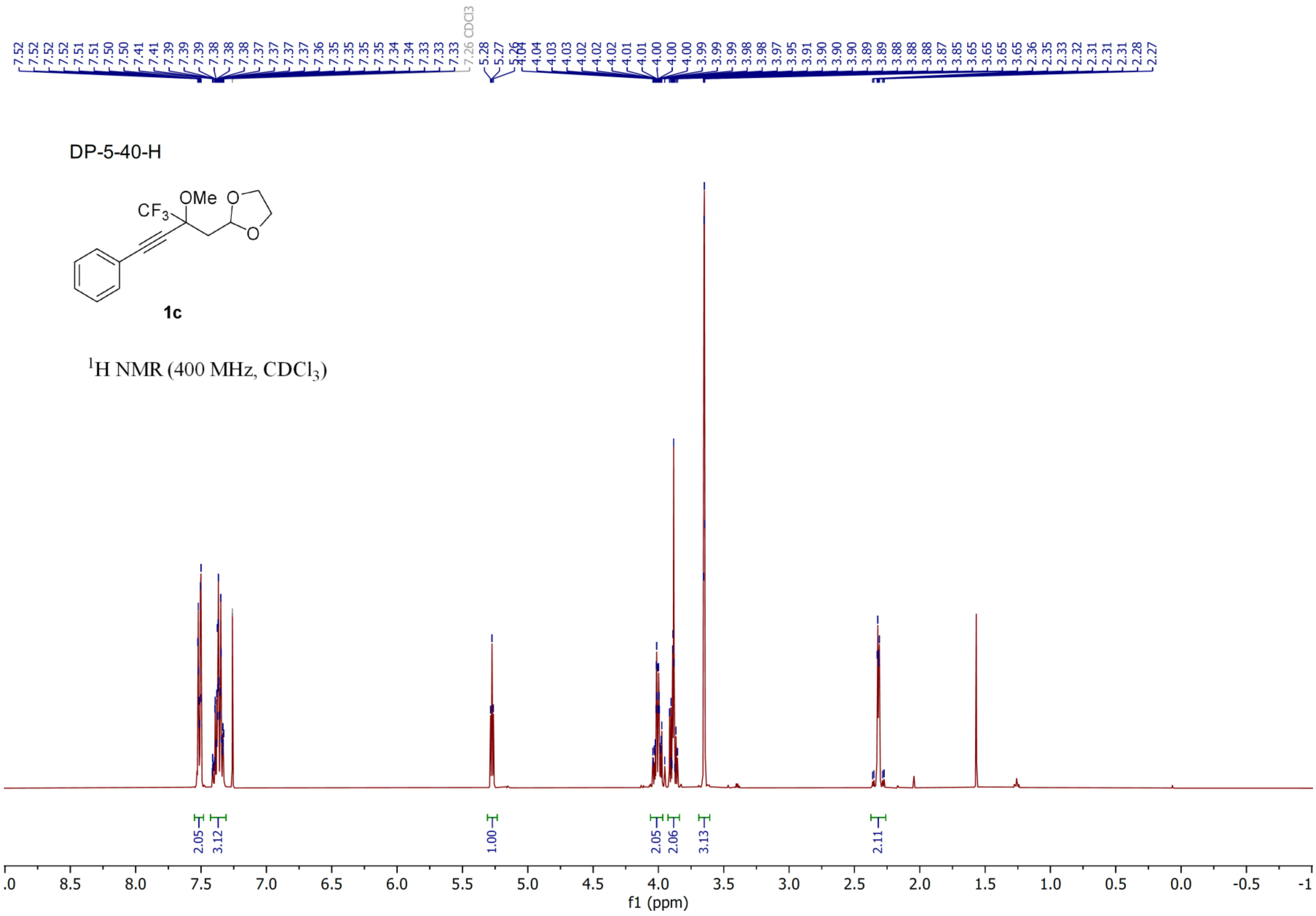
DP-5-40-H
CF3
OMe
O
O
1c
1H NMR (400 MHz, CDCl3)
7.26 CDCl3
2.05
3.12
1.00
2.05
2.06
3.13
2.11
f1 (ppm)

DP-5-40-C

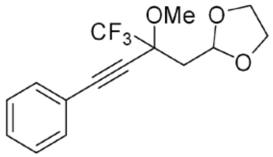

1c

^{13}C NMR (101 MHz, $CDCl_3$)

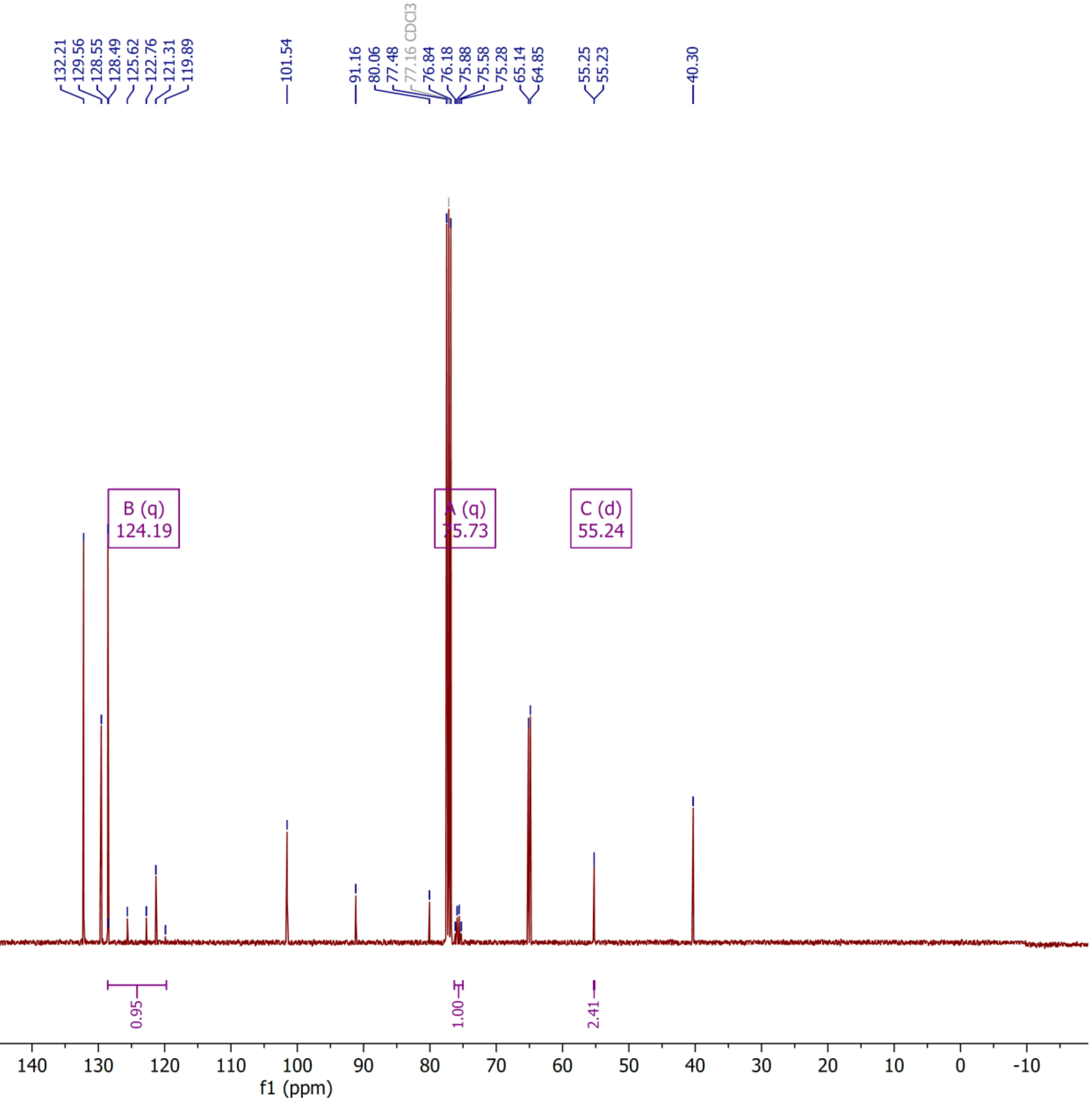

DP-5-40-F

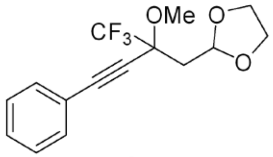

1c

^{19}F NMR (377 MHz, $CDCl_3$)

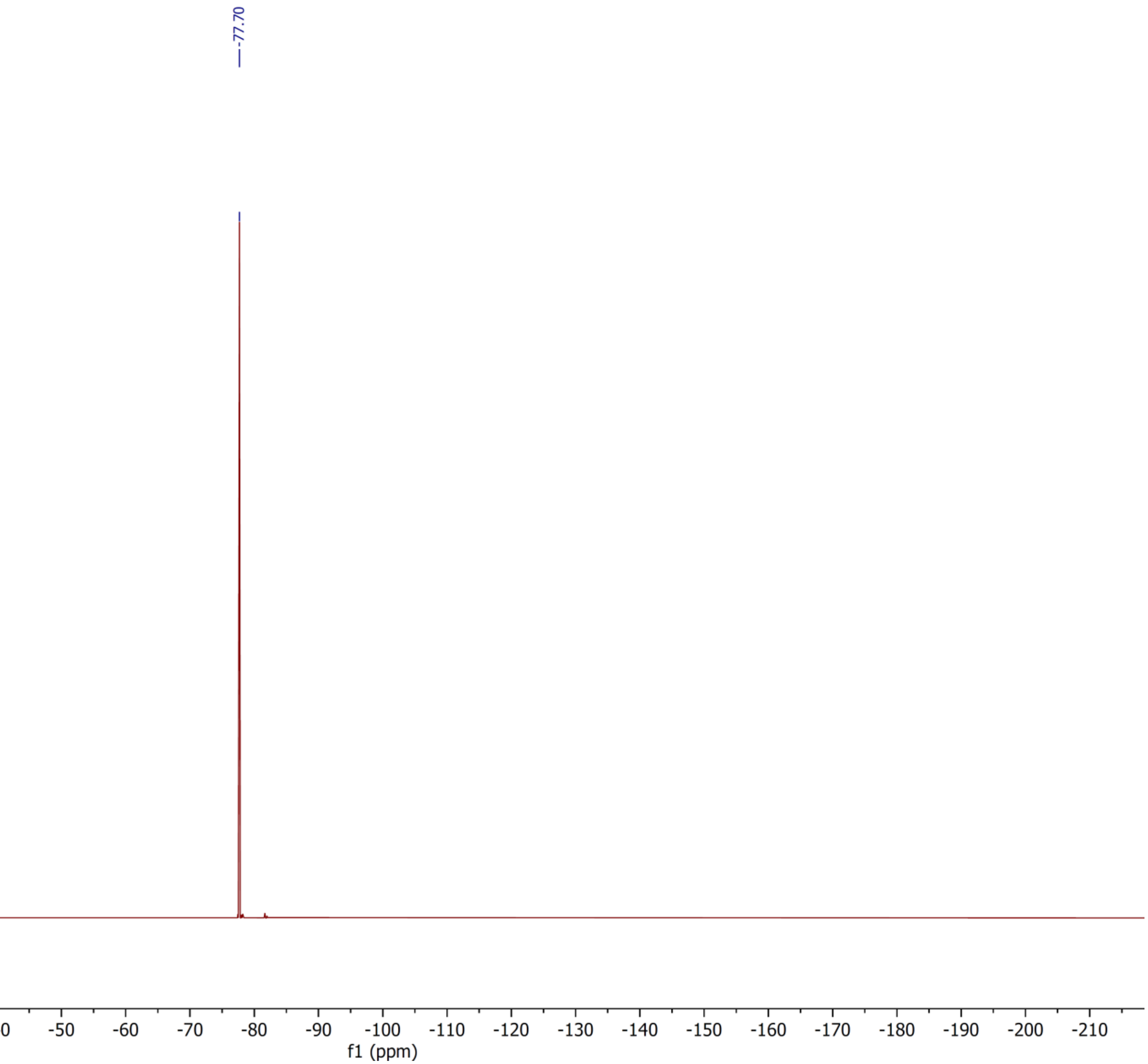

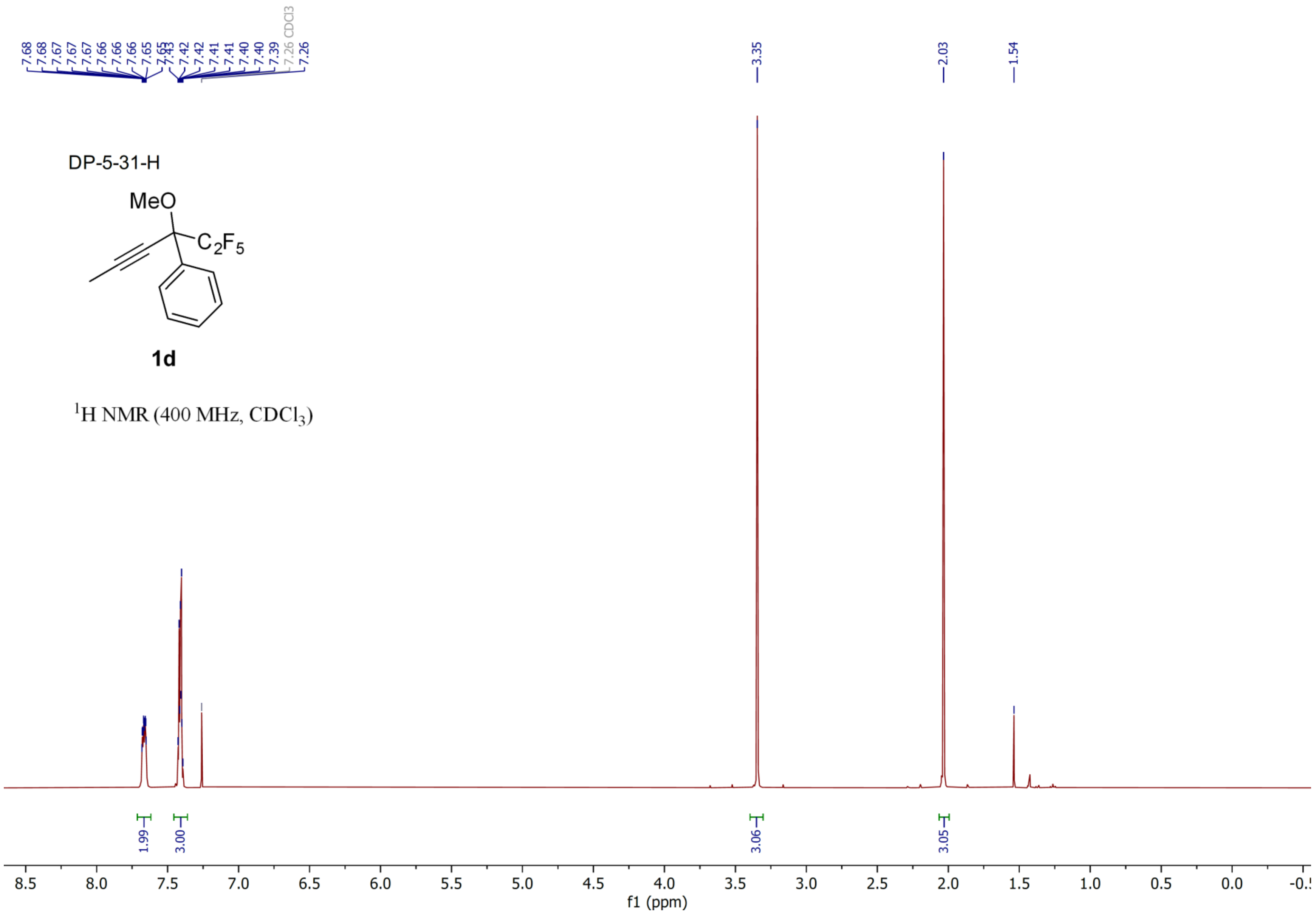
7.68
7.68
7.67
7.67
7.67
7.66
7.66
7.66
7.65
7.65
7.43
7.42
7.42
7.41
7.41
7.40
7.40
7.39
7.26 CDCl3
7.26
3.35
2.03
1.54
DP-5-31-H
MeO
C_2F_5
1d
^{1}H NMR (400 MHz, $CDCl_3$)
1.99
3.00
3.06
3.05
f1 (ppm)

DP-5-31-C

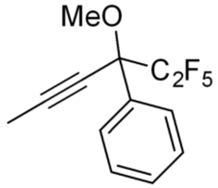

1d

^{13}C NMR (101 MHz, $CDCl_3$)

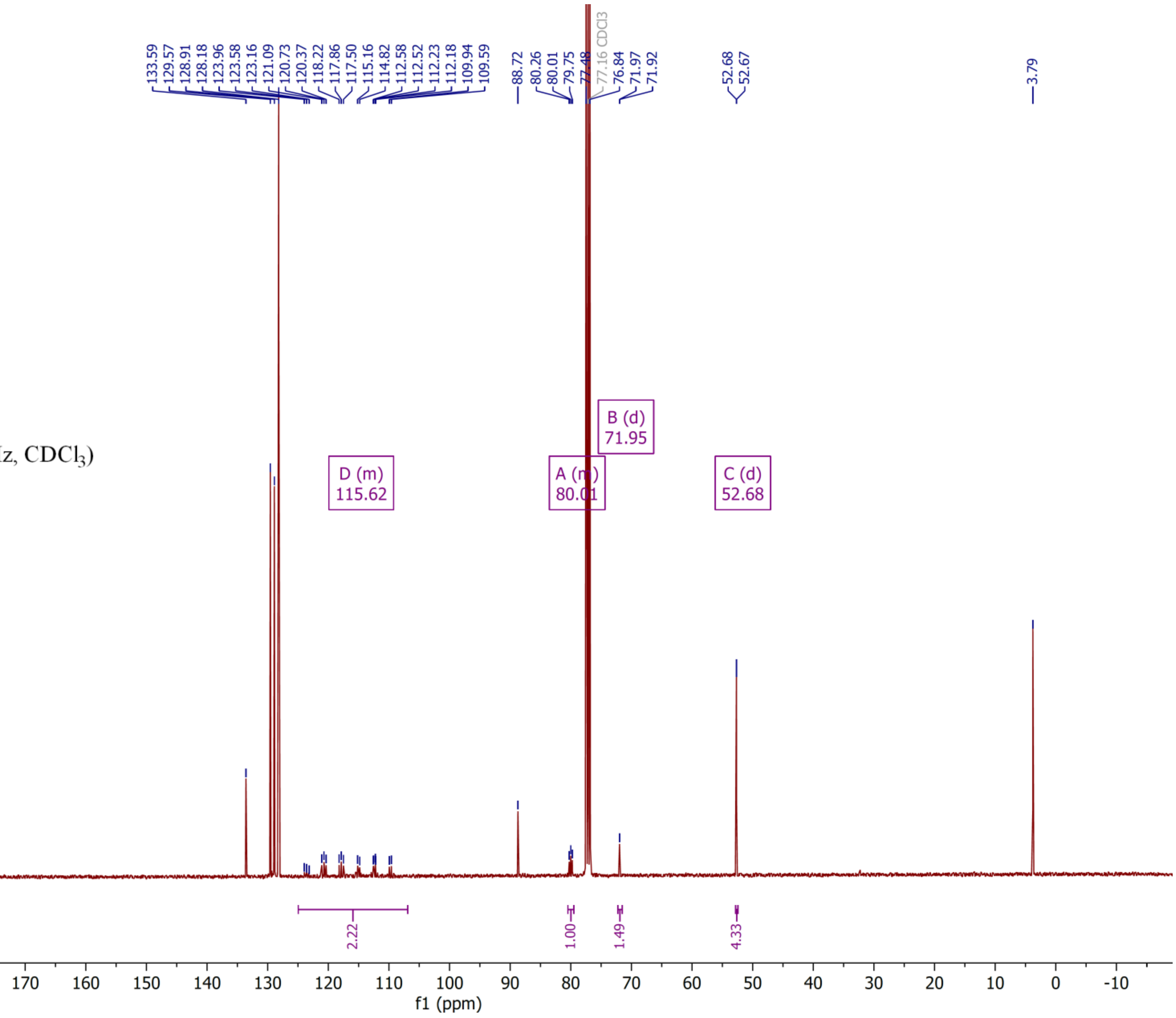

DP-5-31-F

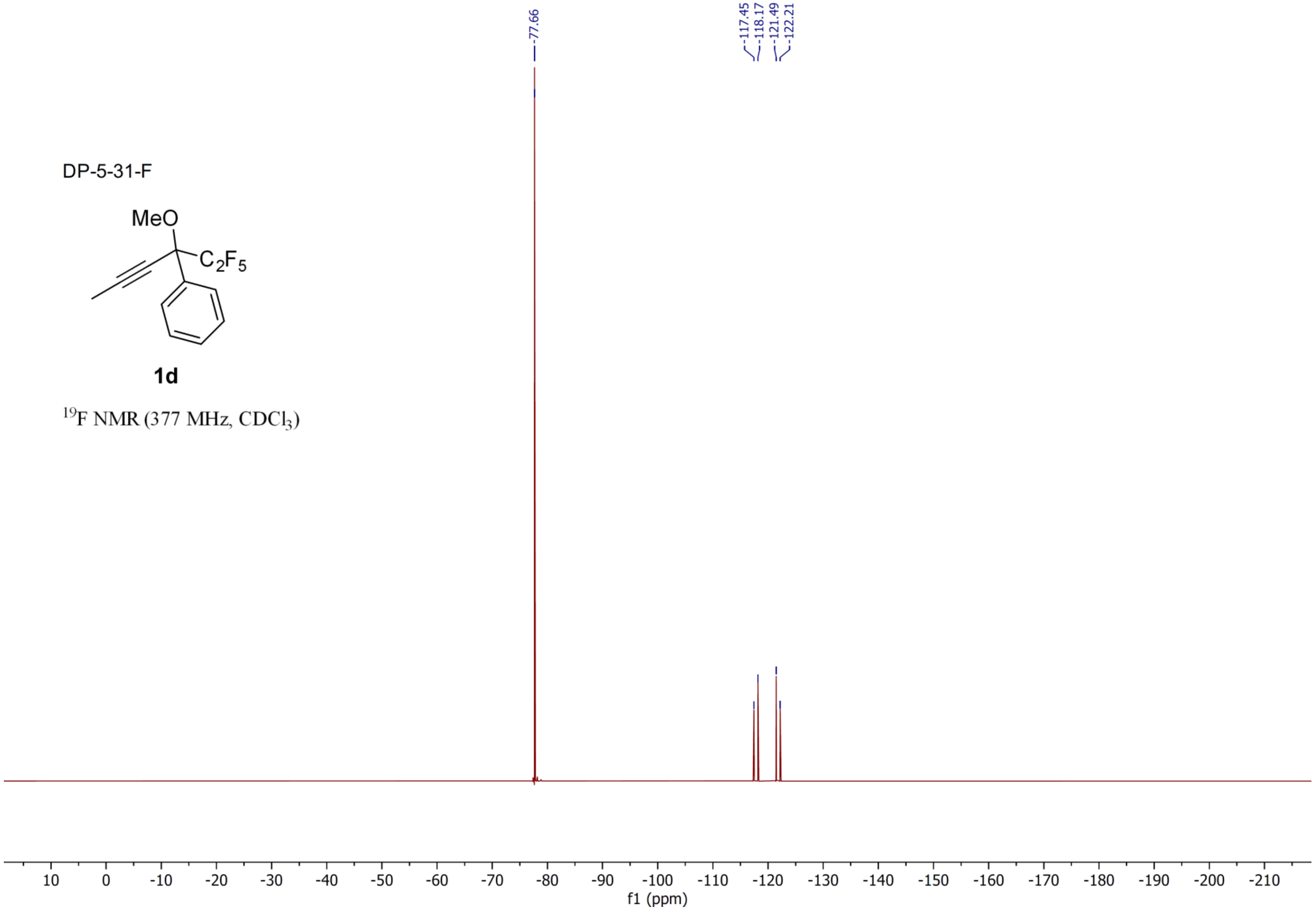

1d

^{19}F NMR (377 MHz, $CDCl_3$)

DP-5-24-H-500mhz

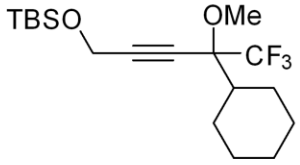

1e

^{1}H NMR (500 MHz, $CDCl_3$)

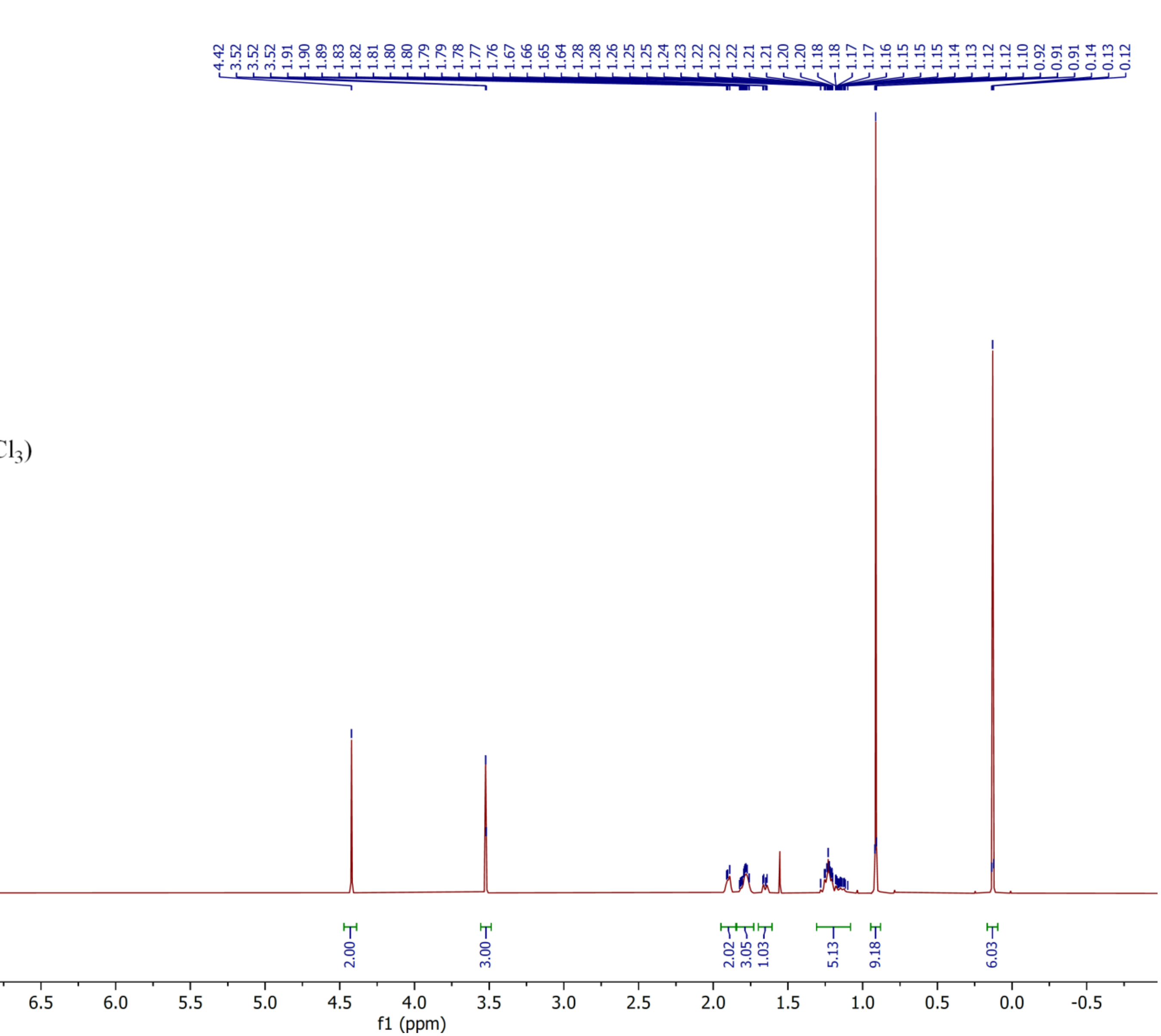

DP-5-24-C-500mhz

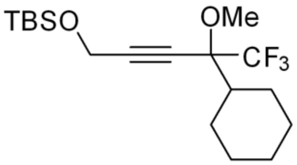

1e

^{13}C NMR (126 MHz, $CDCl_3$)

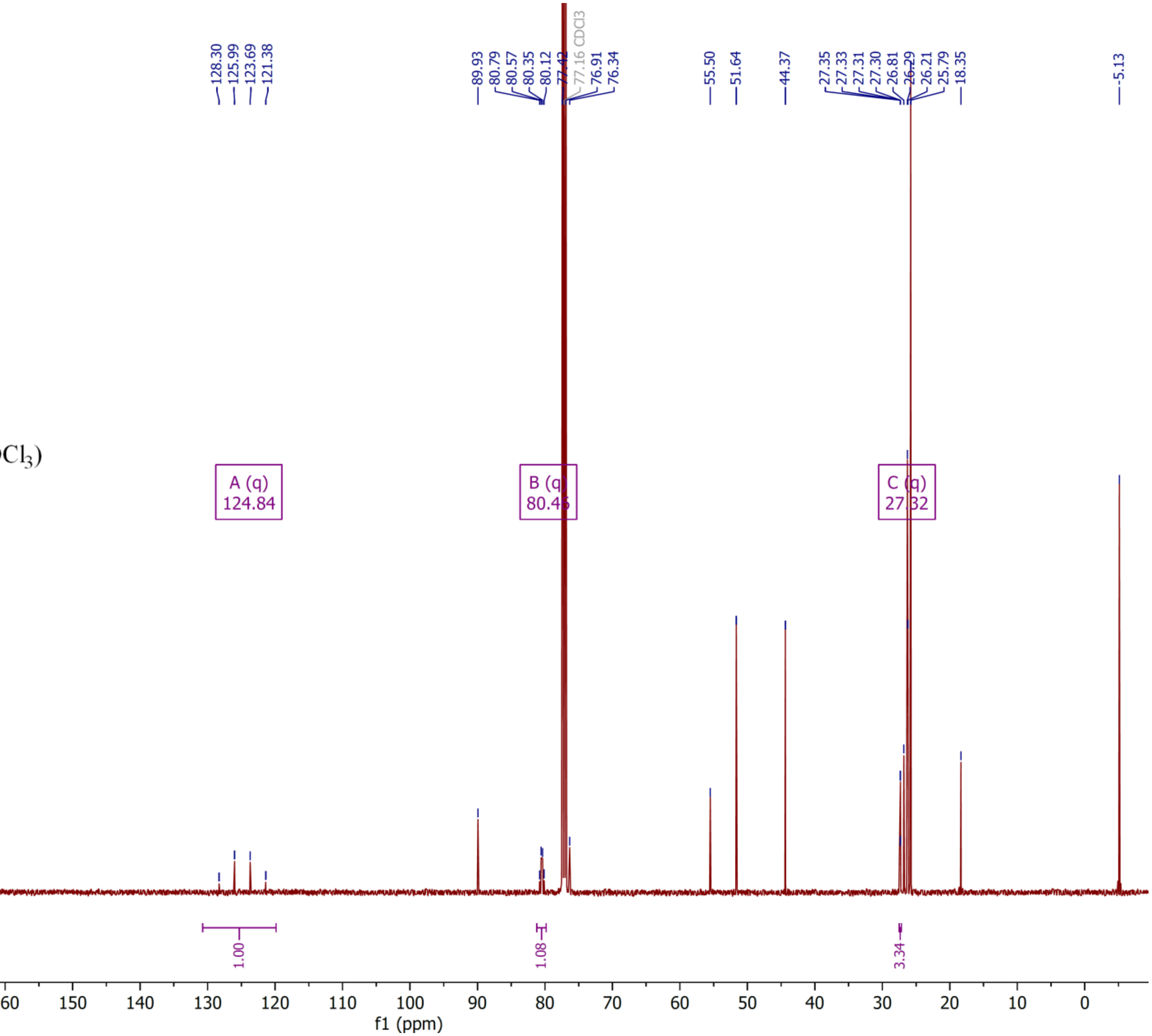

DP-5-24-F

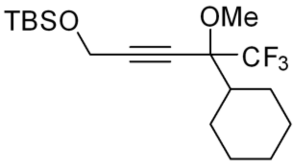

1e

^{19}F NMR (377 MHz, $CDCl_3$)

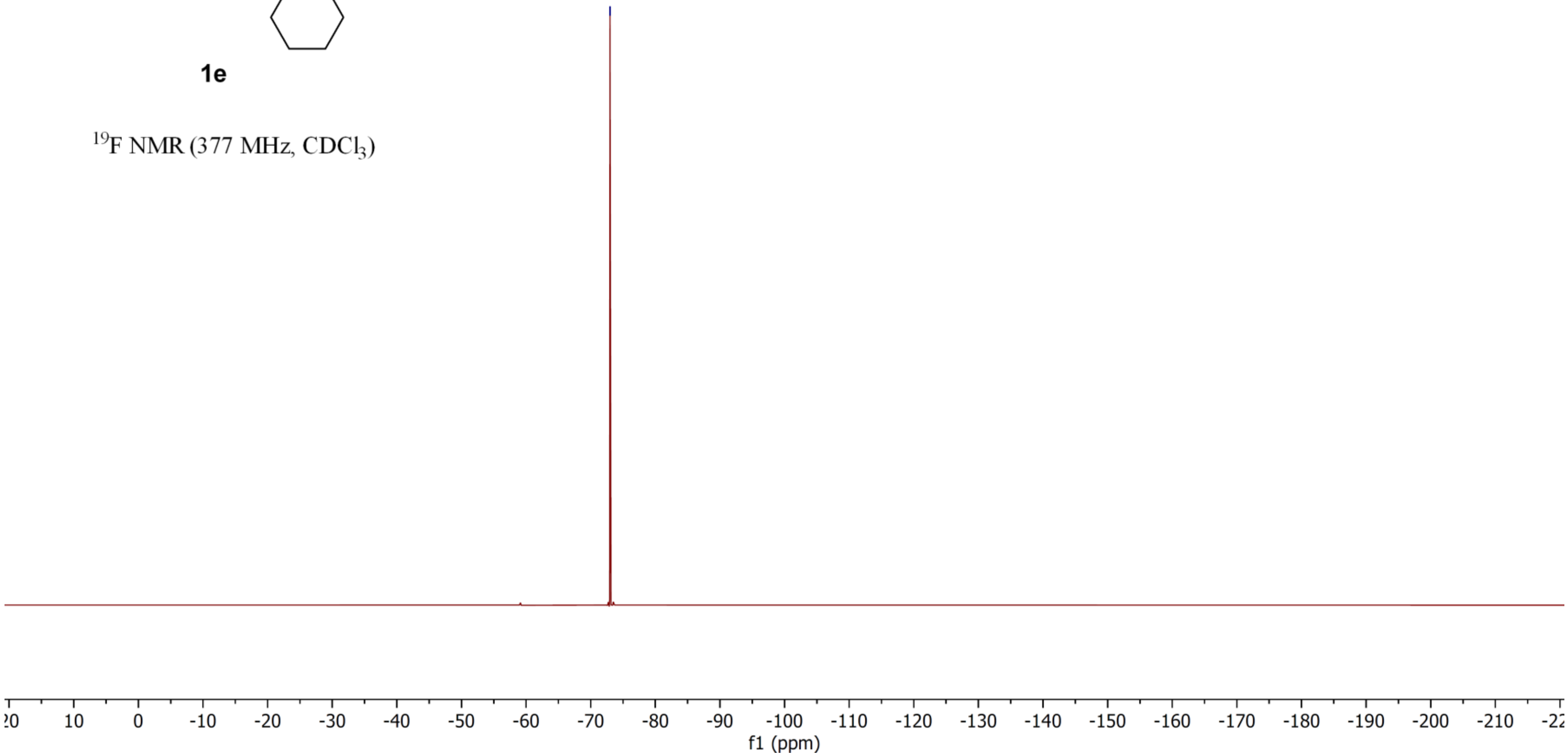

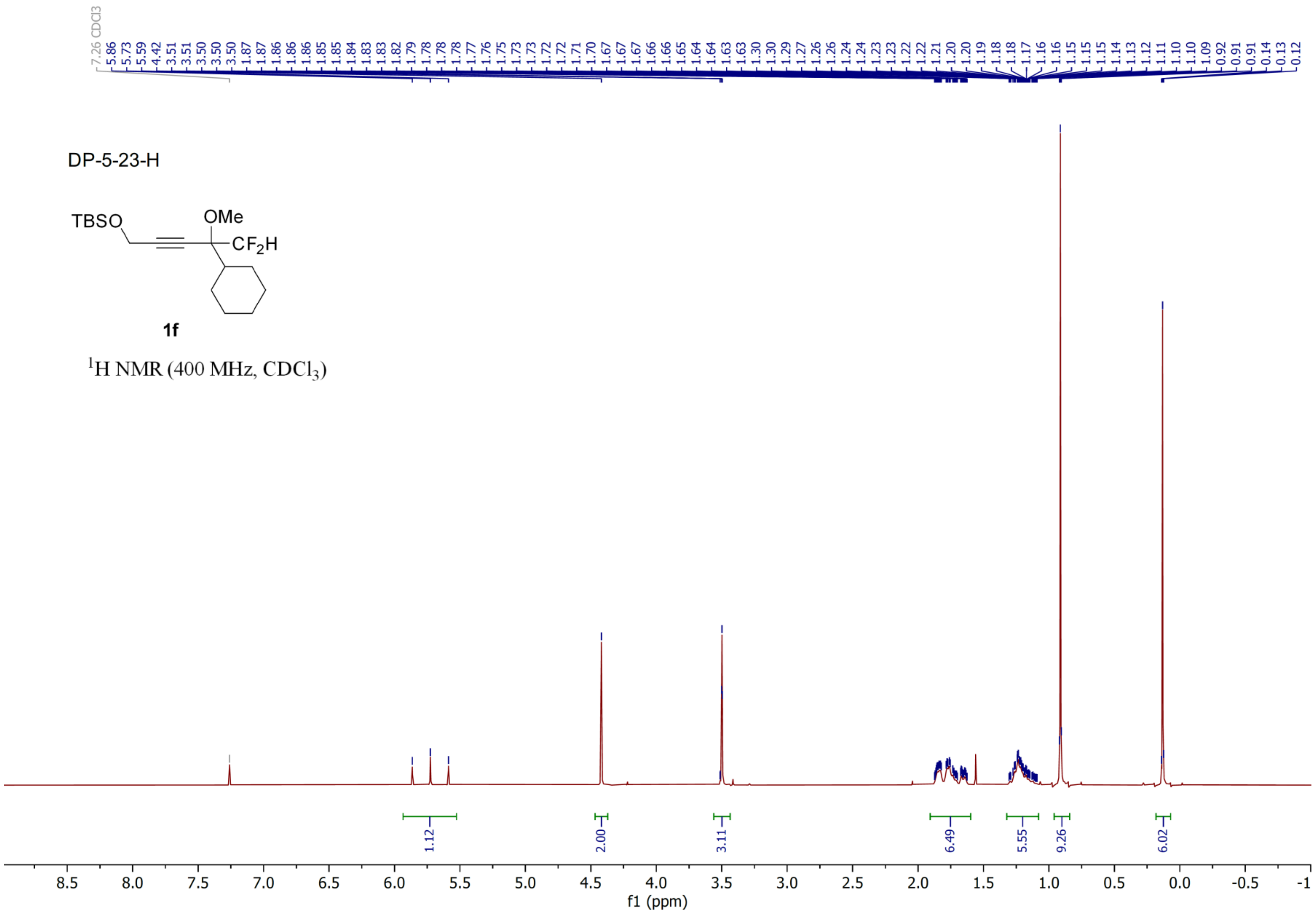
7.26 CDCl3
5.86
5.73
5.59
4.42
3.51
3.51
3.50
3.50
3.50
1.87
1.87
1.86
1.86
1.86
1.85
1.85
1.84
1.83
1.83
1.82
1.79
1.78
1.78
1.78
1.77
1.76
1.75
1.73
1.73
1.72
1.72
1.71
1.70
1.67
1.67
1.66
1.66
1.65
1.64
1.64
1.63
1.63
1.30
1.30
1.29
1.27
1.26
1.26
1.24
1.24
1.23
1.23
1.23
1.22
1.22
1.22
1.21
1.20
1.20
1.19
1.18
1.18
1.17
1.16
1.16
1.15
1.15
1.15
1.14
1.13
1.12
1.11
1.11
1.10
1.09
0.92
0.91
0.91
0.14
0.13
0.12
DP-5-23-H
TBSO
OMe
CF2H
1f
1H NMR (400 MHz, CDCl3)
1.12
2.00
3.11
6.49
5.55
9.26
6.02
8.5
8.0
7.5
7.0
6.5
6.0
5.5
5.0
4.5
4.0
3.5
3.0
2.5
2.0
1.5
1.0
0.5
0.0
-0.5
-1
f1 (ppm)

DP-5-23-C

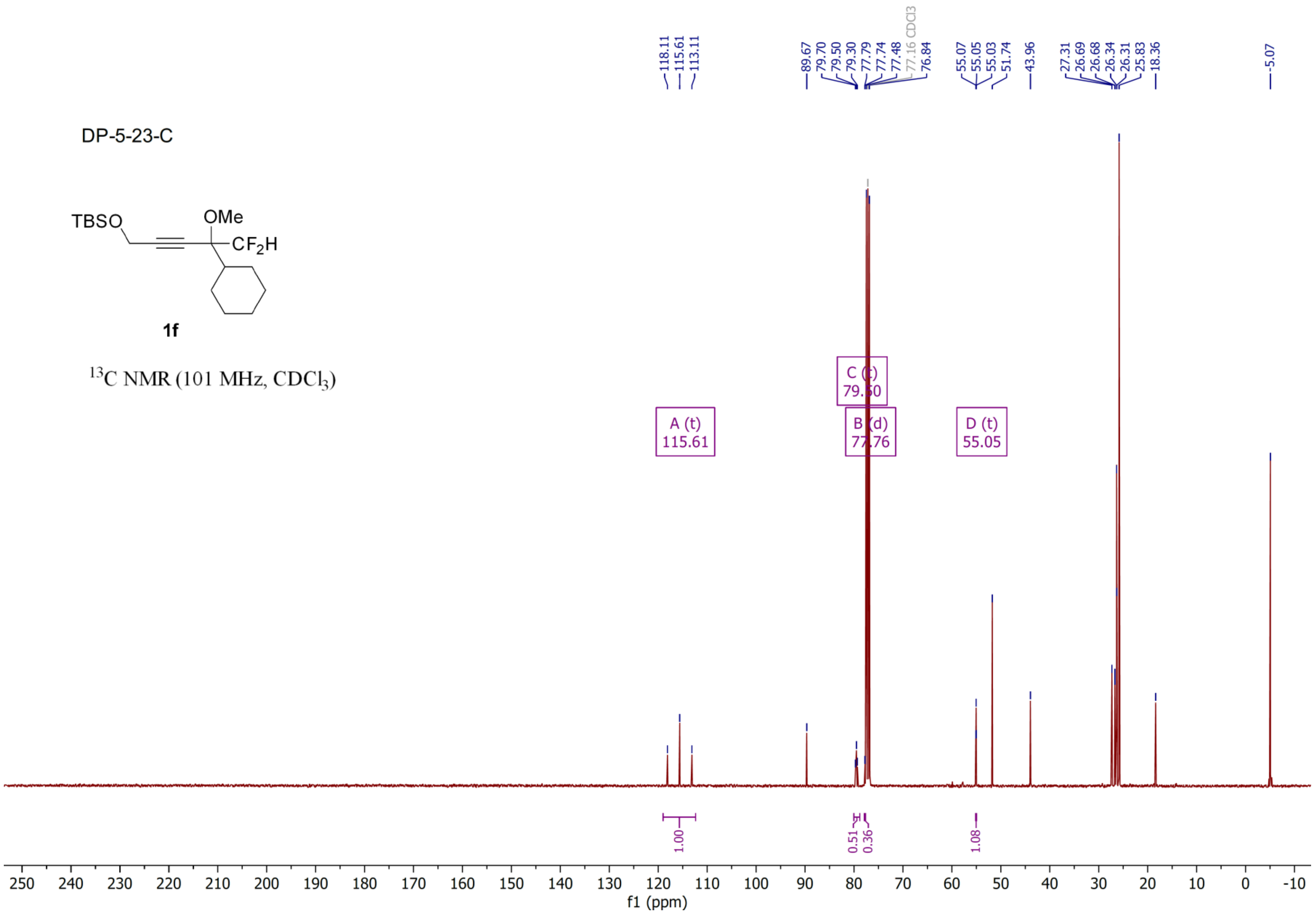

1f

^{13}C NMR (101 MHz, CDCl$_3$)

DP-5-23-F

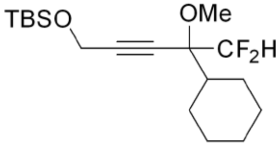

1f

^{19}F NMR (377 MHz, $CDCl_3$)

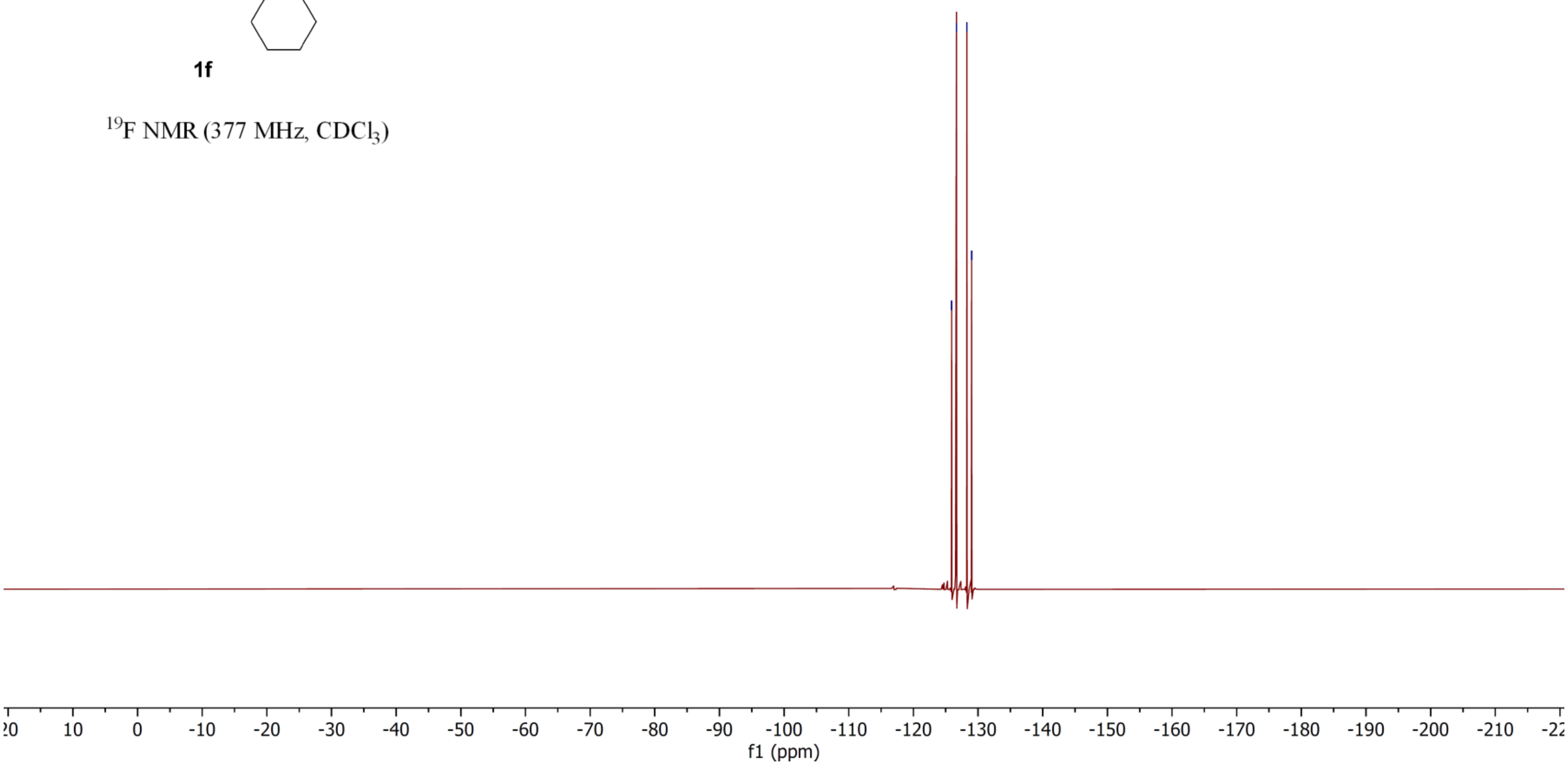

DP-5-37-H-500mhz

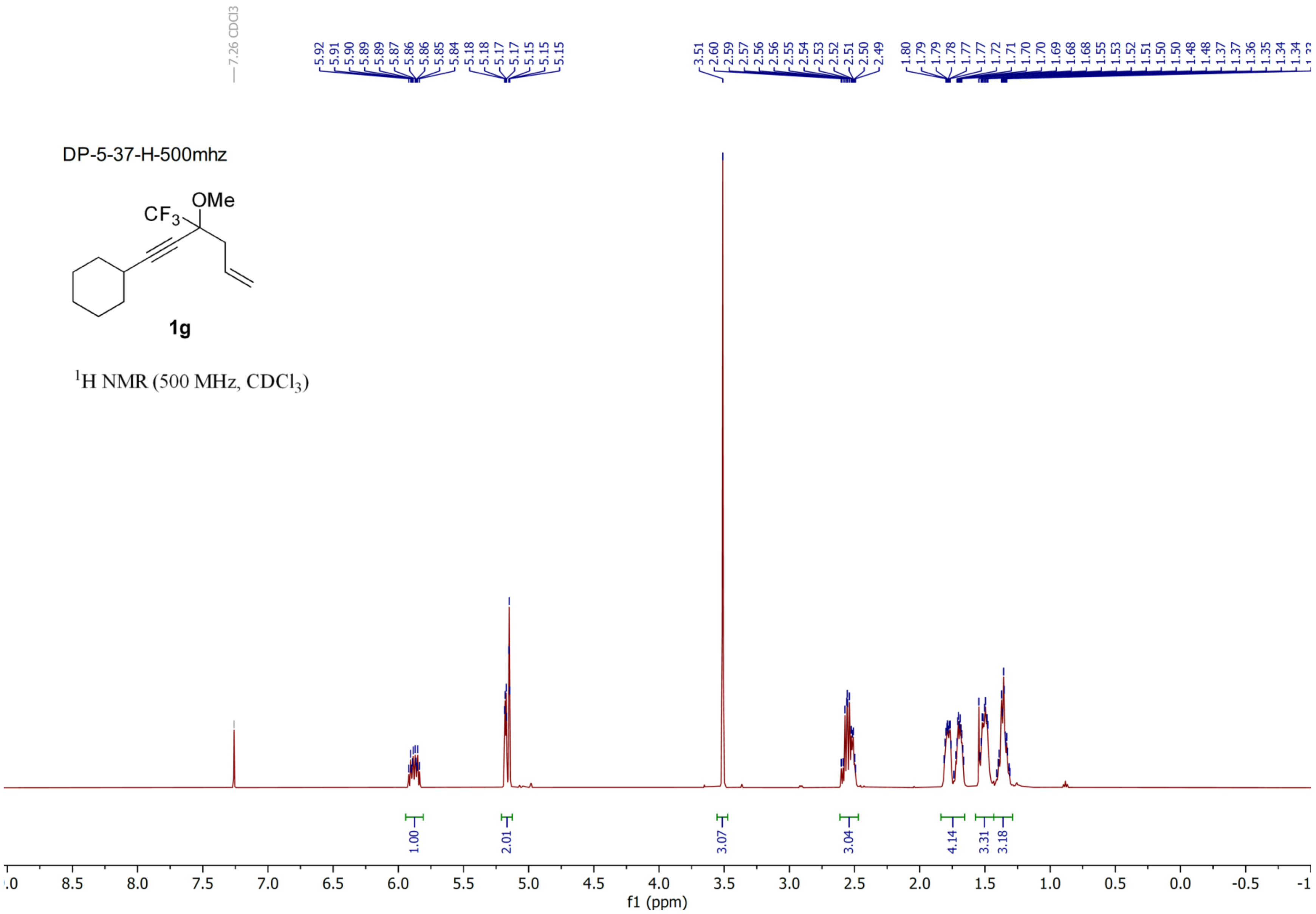

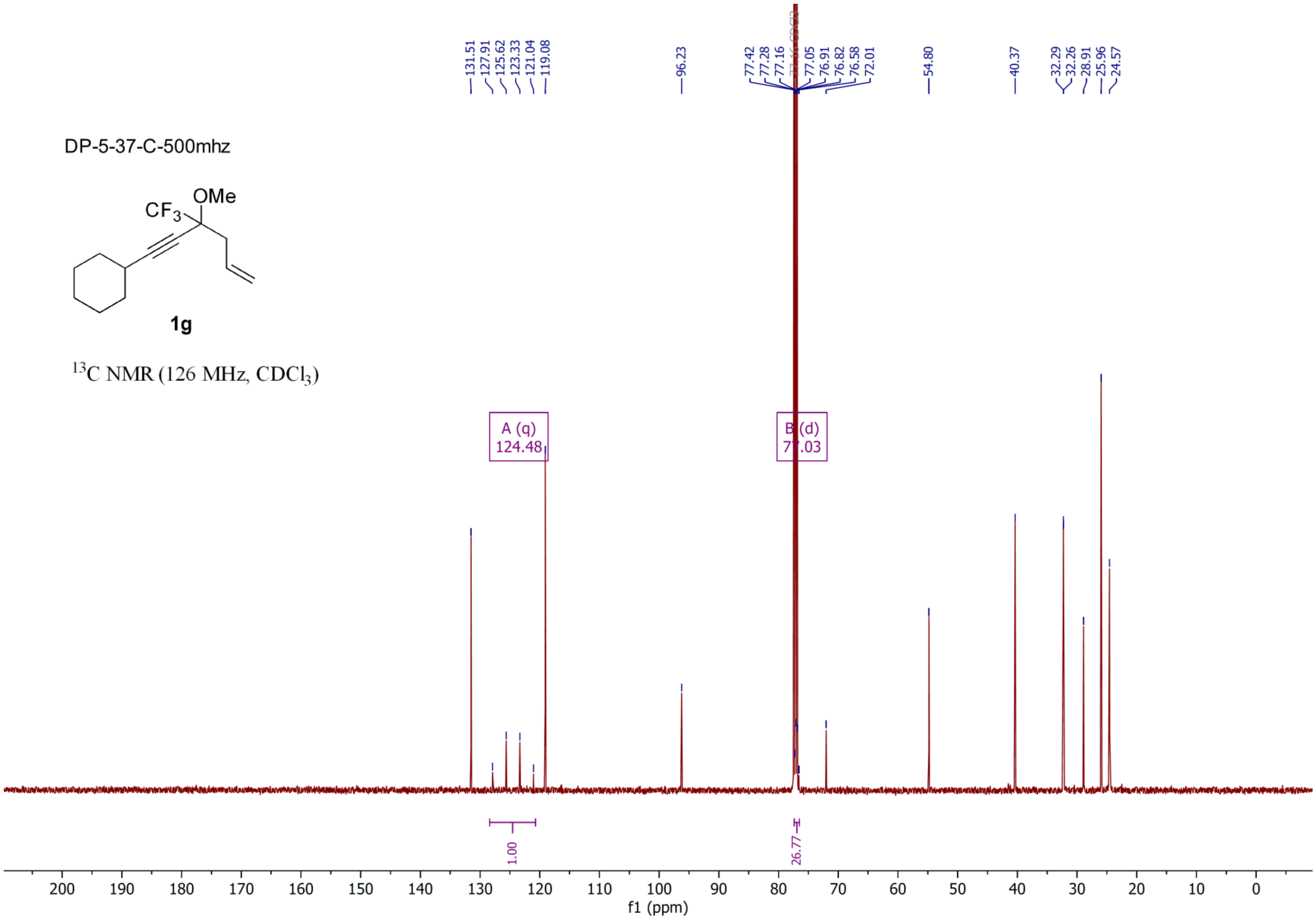
DP-5-37-C-500mhz
CF3
OMe
1g
^{13}C NMR (126 MHz, CDCl$_3$)
131.51
127.91
125.62
123.33
121.04
119.08
96.23
77.42
77.28
77.16
77.05
76.91
76.82
76.58
72.01
54.80
40.37
32.29
32.26
28.91
25.96
24.57
A (q)
124.48
B (d)
77.03
1.00
26.77
200
190
180
170
160
150
140
130
120
110
100
90
80
70
60
50
40
30
20
10
0
f1 (ppm)

DP-5-37-F

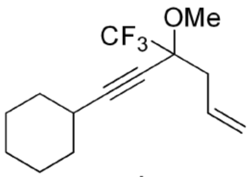

1g

^{19}F NMR (377 MHz, $CDCl_3$)

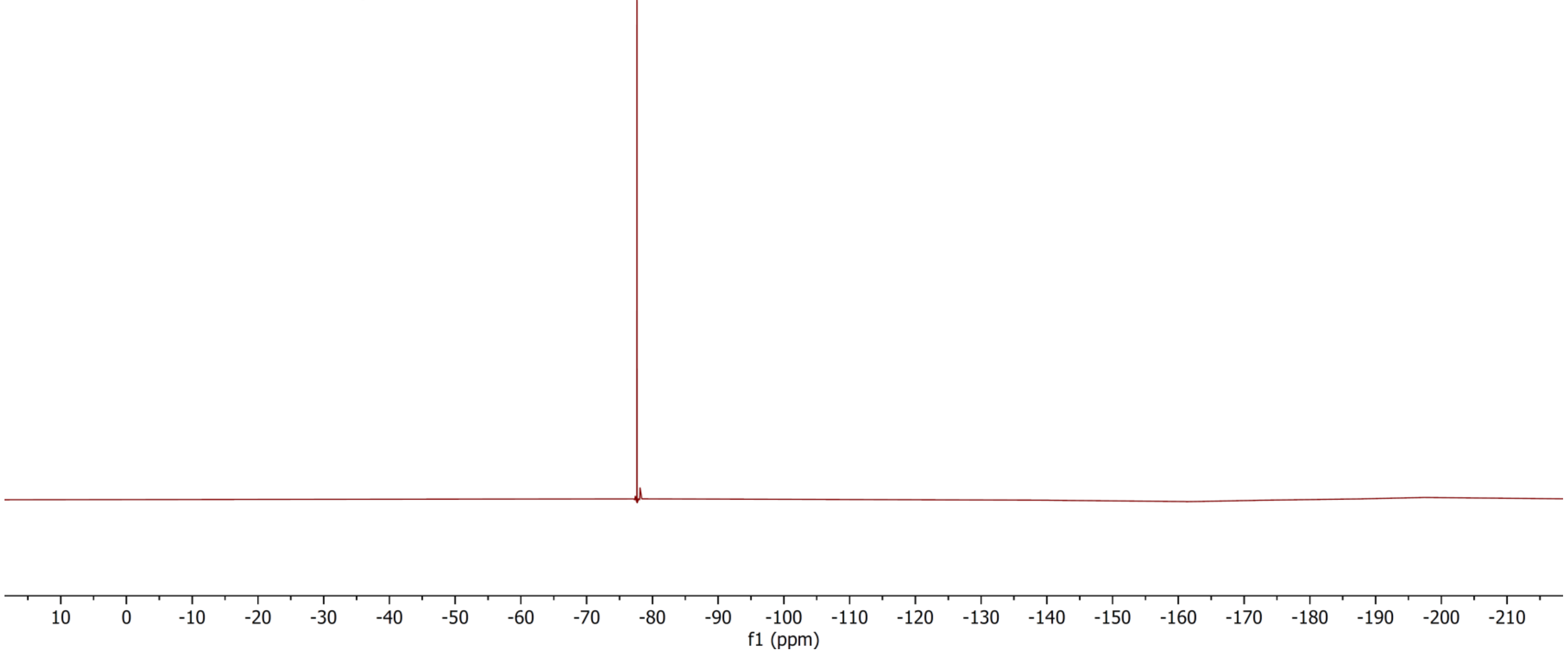

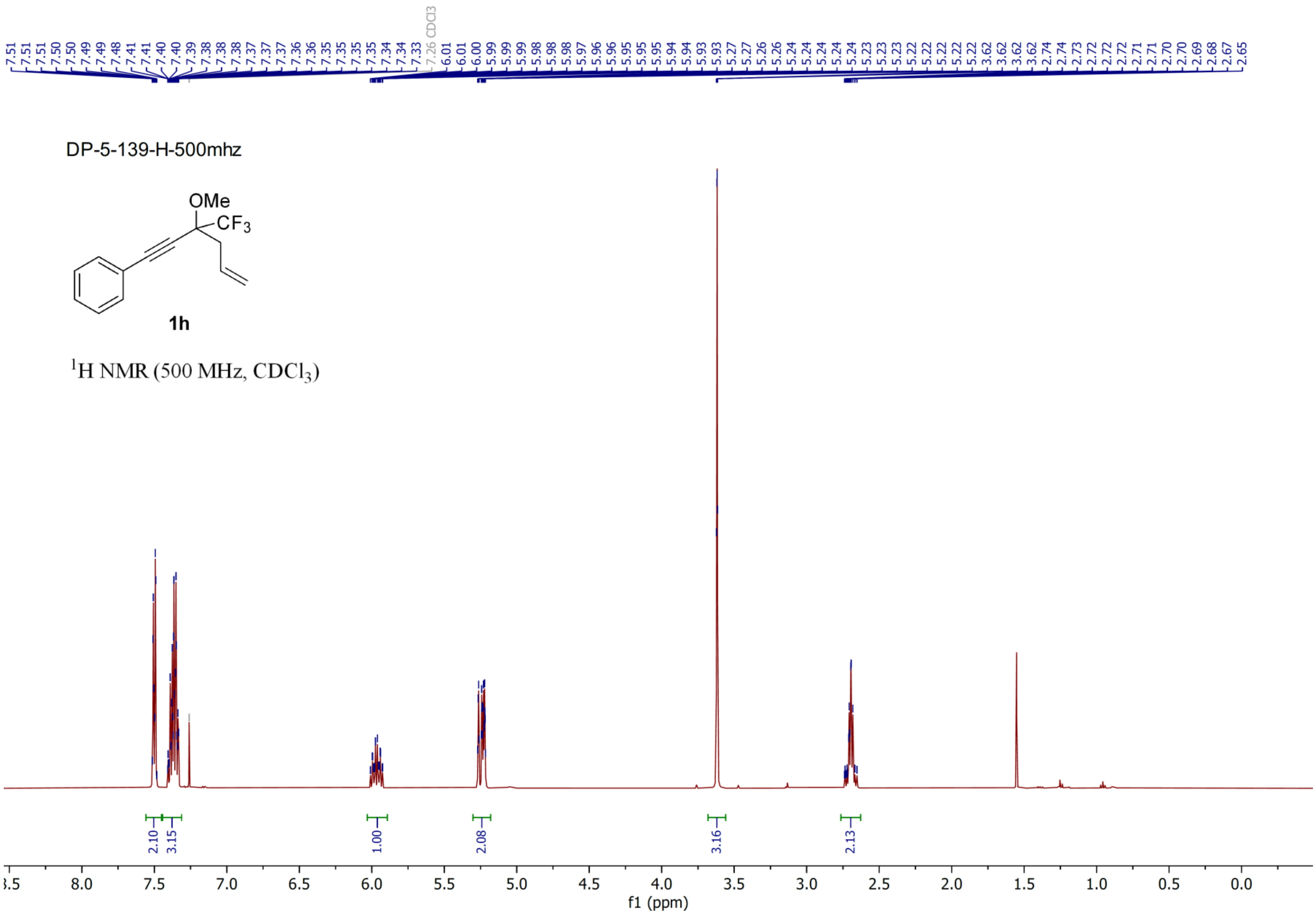

DP-5-139-H-500mhz
OMe
CF3
1h
1H NMR (500 MHz, CDCl3)
CDCl3
f1 (ppm)

DP-5-139-C-500mhz

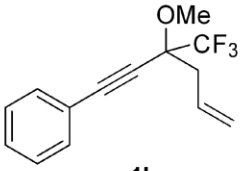

1h

^{13}C NMR (126 MHz, $CDCl_3$)

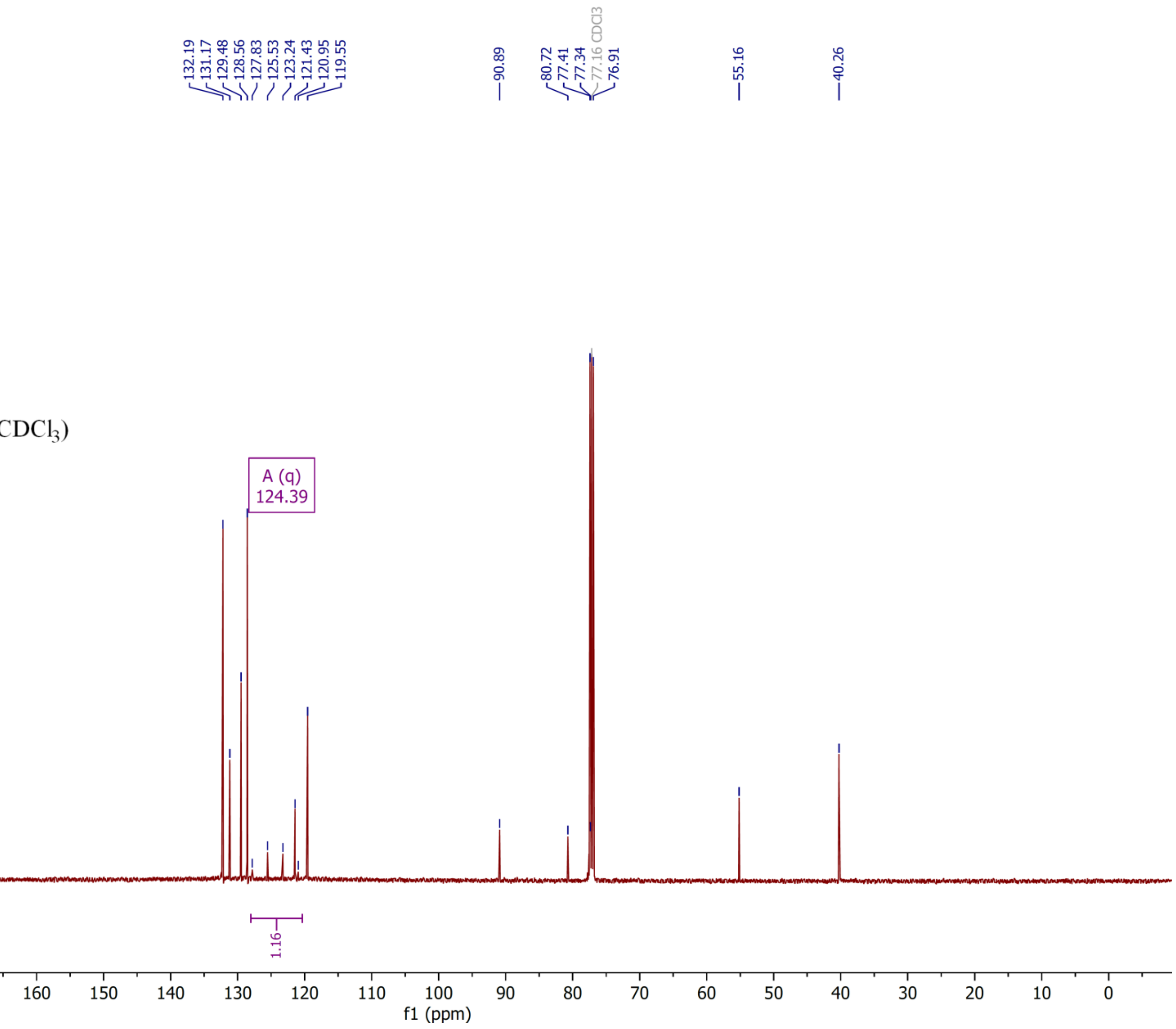

DP-5-139-F

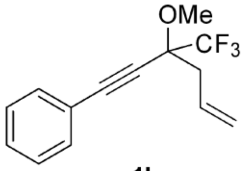

1h

^{19}F NMR (377 MHz, $CDCl_3$)

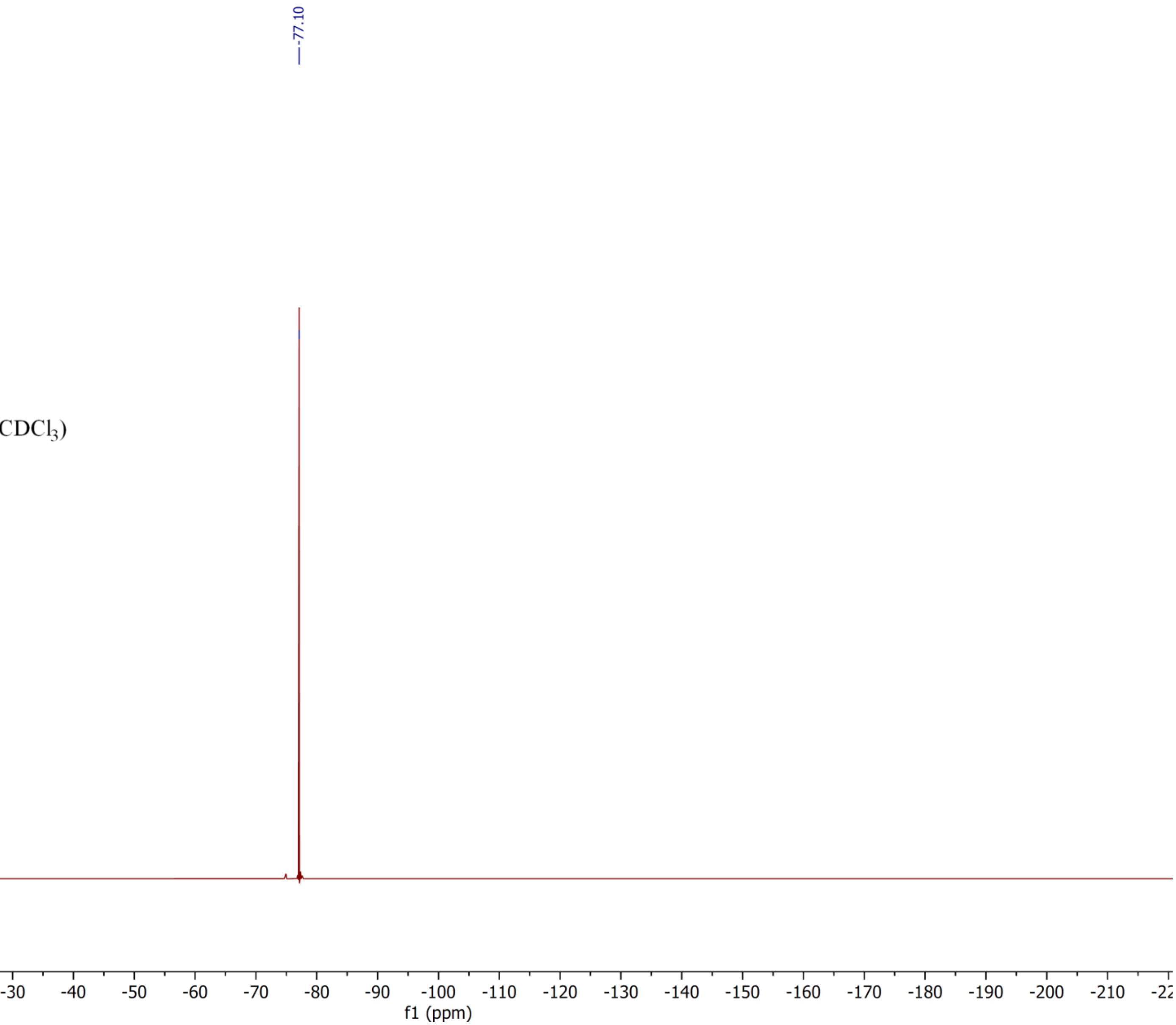

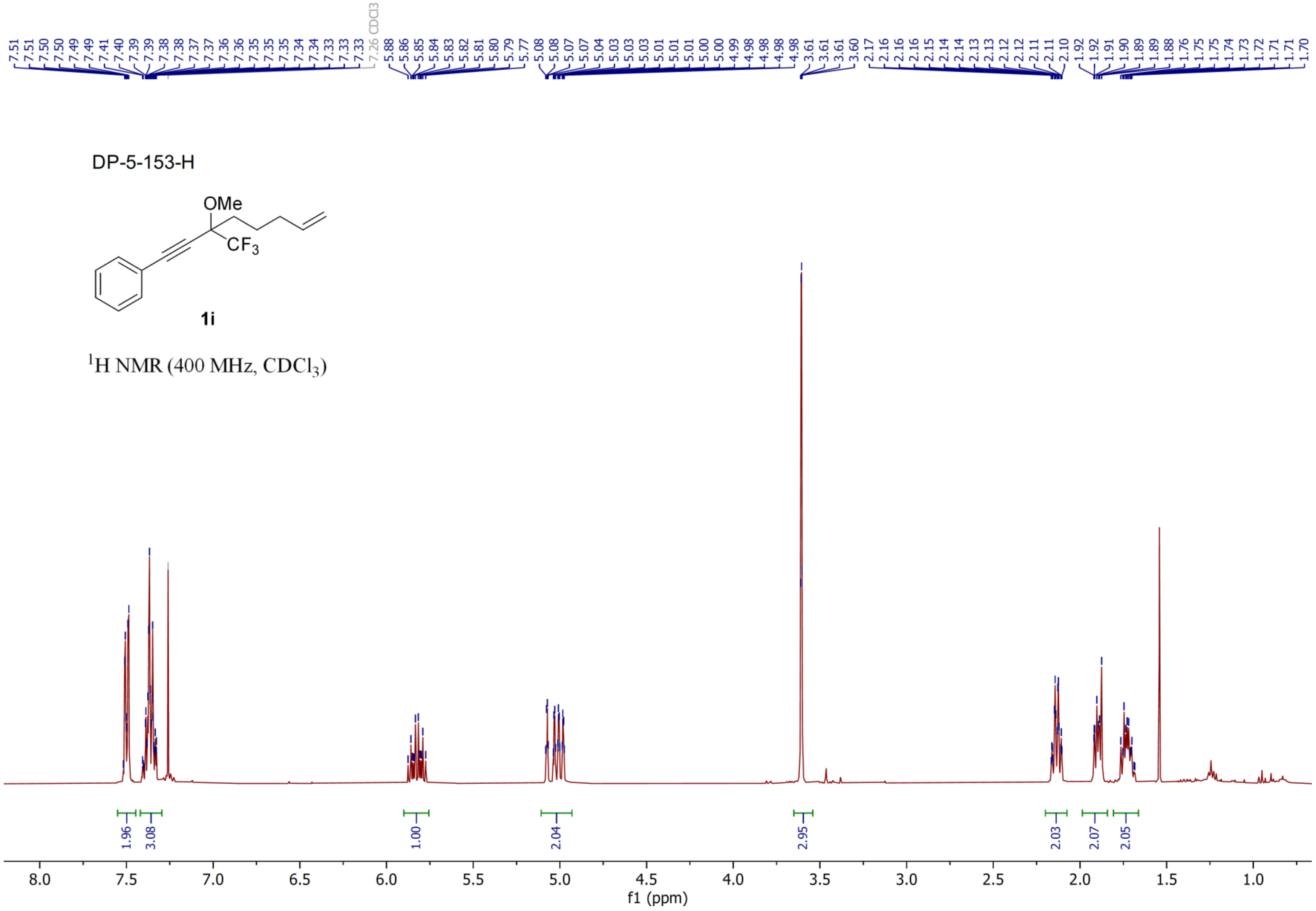

7.51 7.51 7.50 7.49 7.49 7.41 7.40 7.39 7.39 7.38 7.38 7.37 7.37 7.36 7.36 7.35 7.35 7.35 7.34 7.34 7.33 7.33 7.33
7.26 CDCl3
5.88 5.86 5.85 5.84 5.83 5.82 5.81 5.80 5.79 5.77
5.08 5.08 5.07 5.07 5.04 5.03 5.03 5.03 5.01 5.01 5.01 5.00 5.00 4.99 4.98 4.98 4.98 4.98
3.61 3.61 3.61 3.60
2.17 2.16 2.16 2.15 2.14 2.14 2.13 2.13 2.12 2.12 2.11 2.11 2.10
1.92 1.92 1.91 1.90 1.89 1.89 1.88 1.76 1.75 1.75 1.74 1.73 1.72 1.71 1.71 1.70
DP-5-153-H
OMe
CF3
1i
1H NMR (400 MHz, CDCl3)
1.96
3.08
1.00
2.04
2.95
2.03
2.07
2.05
8.0
7.5
7.0
6.5
6.0
5.5
5.0
4.5
4.0
3.5
3.0
2.5
2.0
1.5
1.0
f1 (ppm)

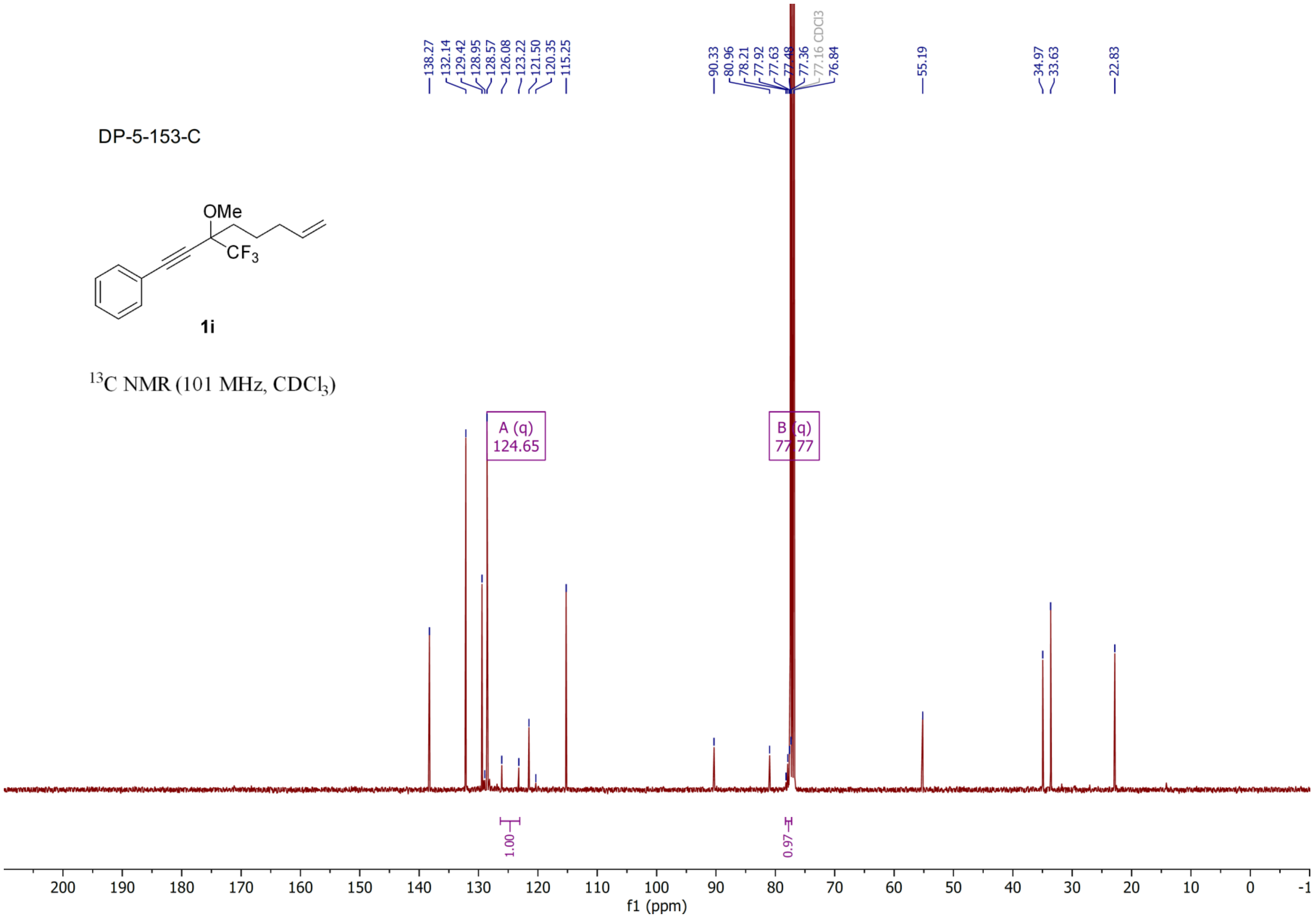
DP-5-153-C
OMe
CF3
1i
13C NMR (101 MHz, CDCl3)
138.27
132.14
129.42
128.95
128.57
126.08
123.22
121.50
120.35
115.25
90.33
80.96
78.21
77.92
77.63
77.48
77.36
77.16 CDCl3
76.84
55.19
34.97
33.63
22.83
A (q)
124.65
B (q)
77.77
1.00
0.97
f1 (ppm)

DP-5-153-F

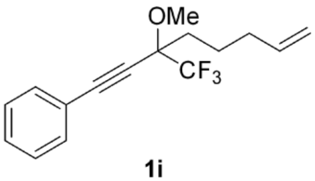

1i

^{19}F NMR (377 MHz, $CDCl_3$)

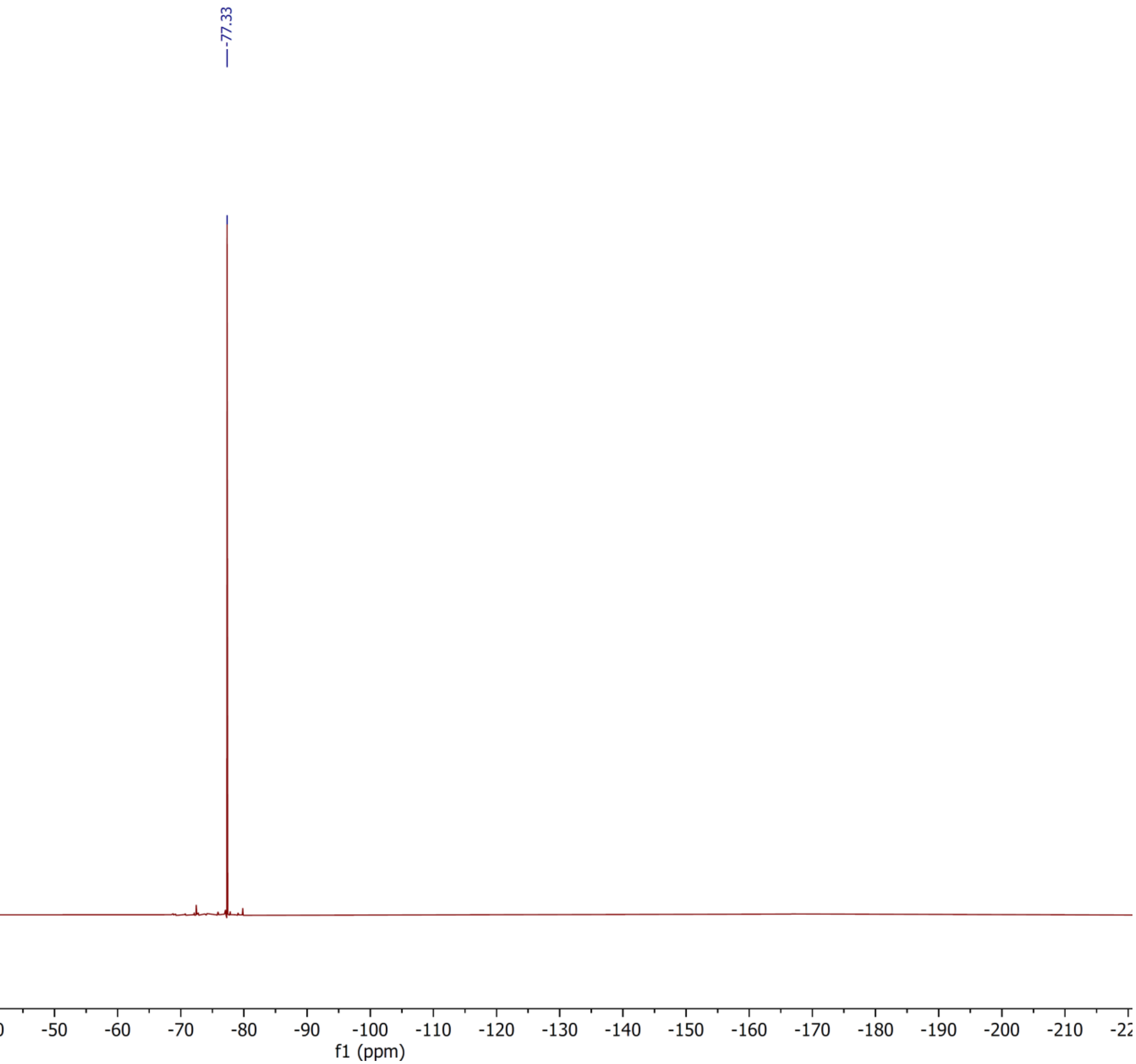

DP-5-34-H-500mhz

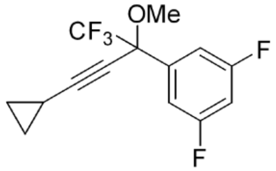

1j

^1H NMR (500 MHz, $CDCl_3$)

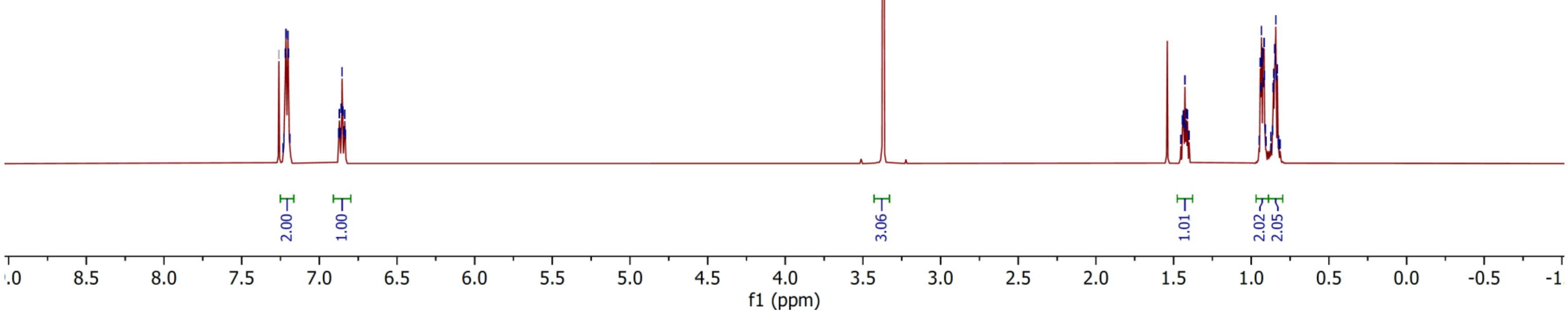

DP-5-34-C-500mhz

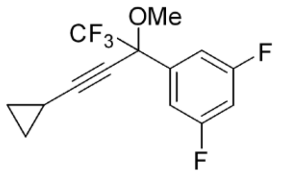

1j

^{13}C NMR (126 MHz, $CDCl_3$)

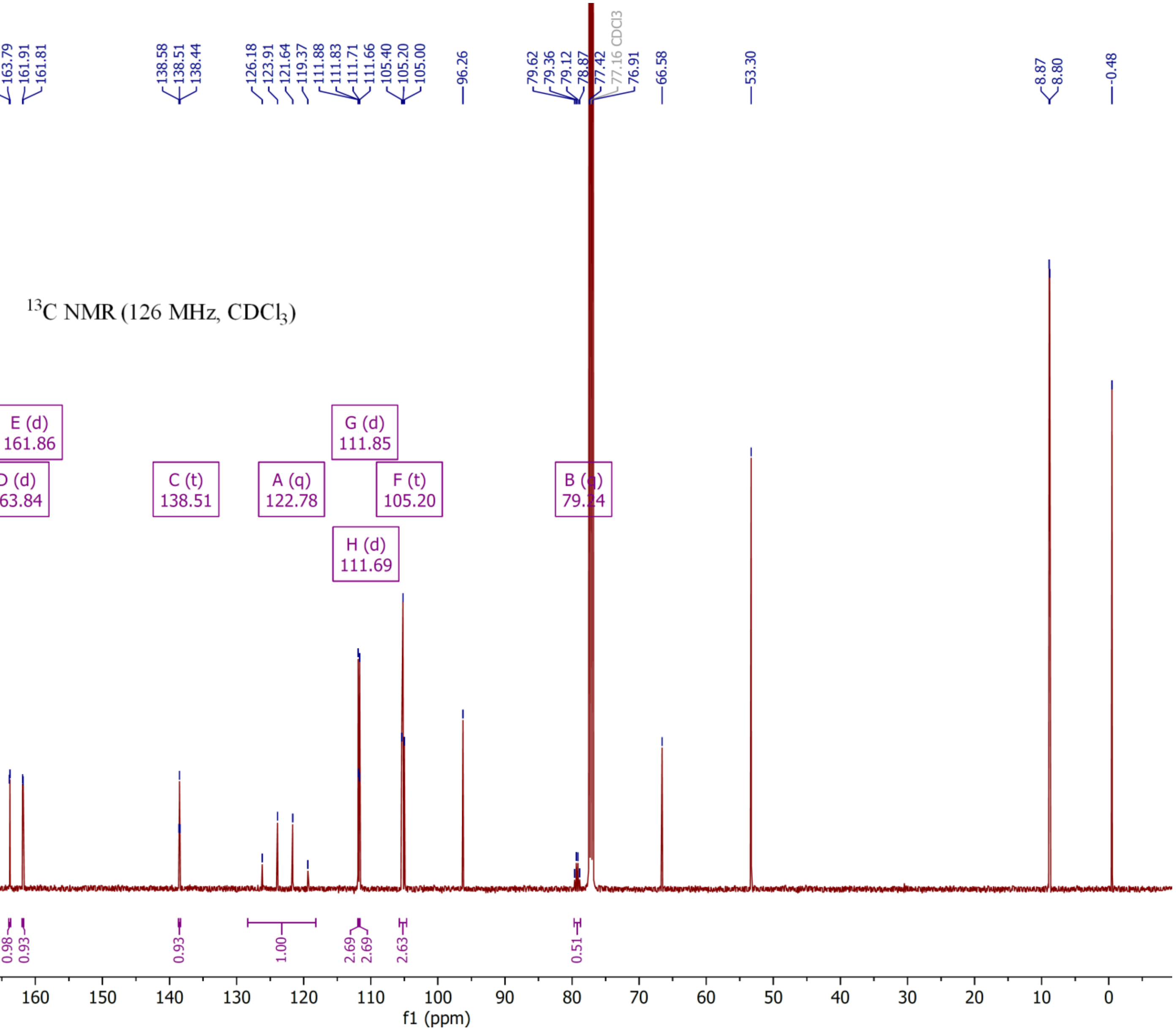

DP-5-34-F

1j

^{19}F NMR (377 MHz, $CDCl_3$)

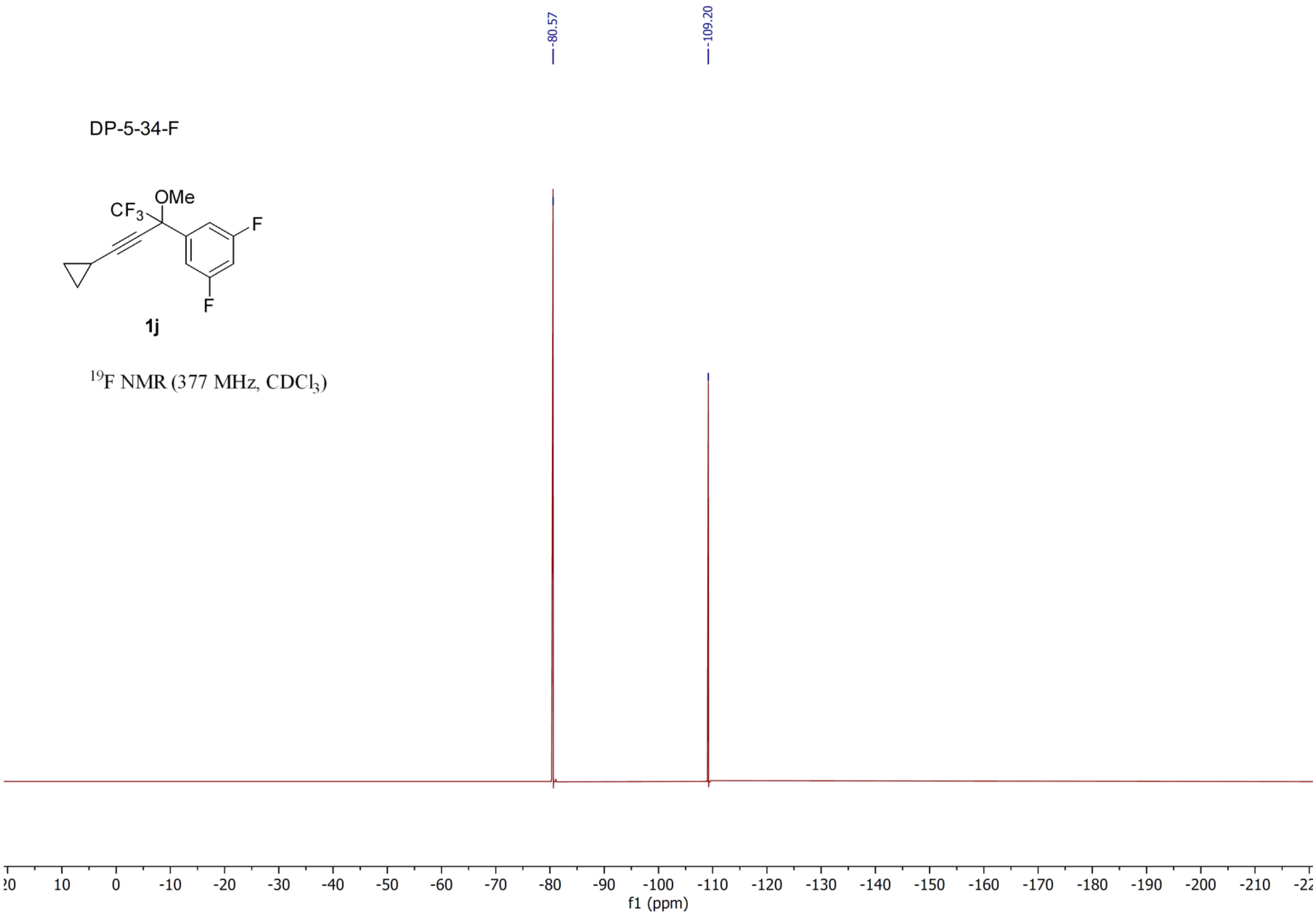

DP-5-46-H

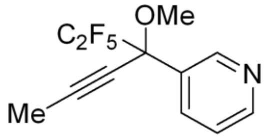

1k

^{1}H NMR (400 MHz, $CDCl_3$)

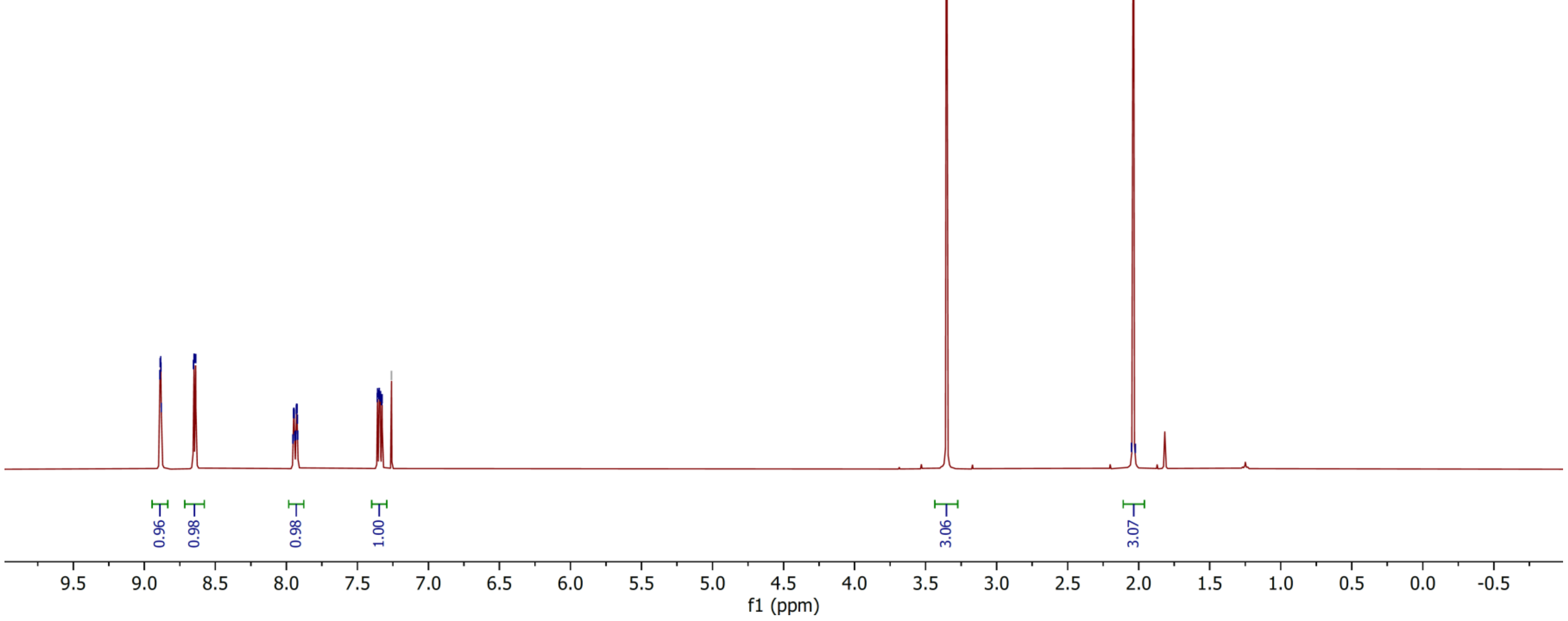

DP-5-46-C

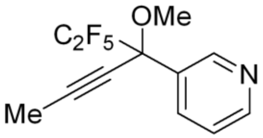

1k

^{19}F NMR (377 MHz, $CDCl_3$)

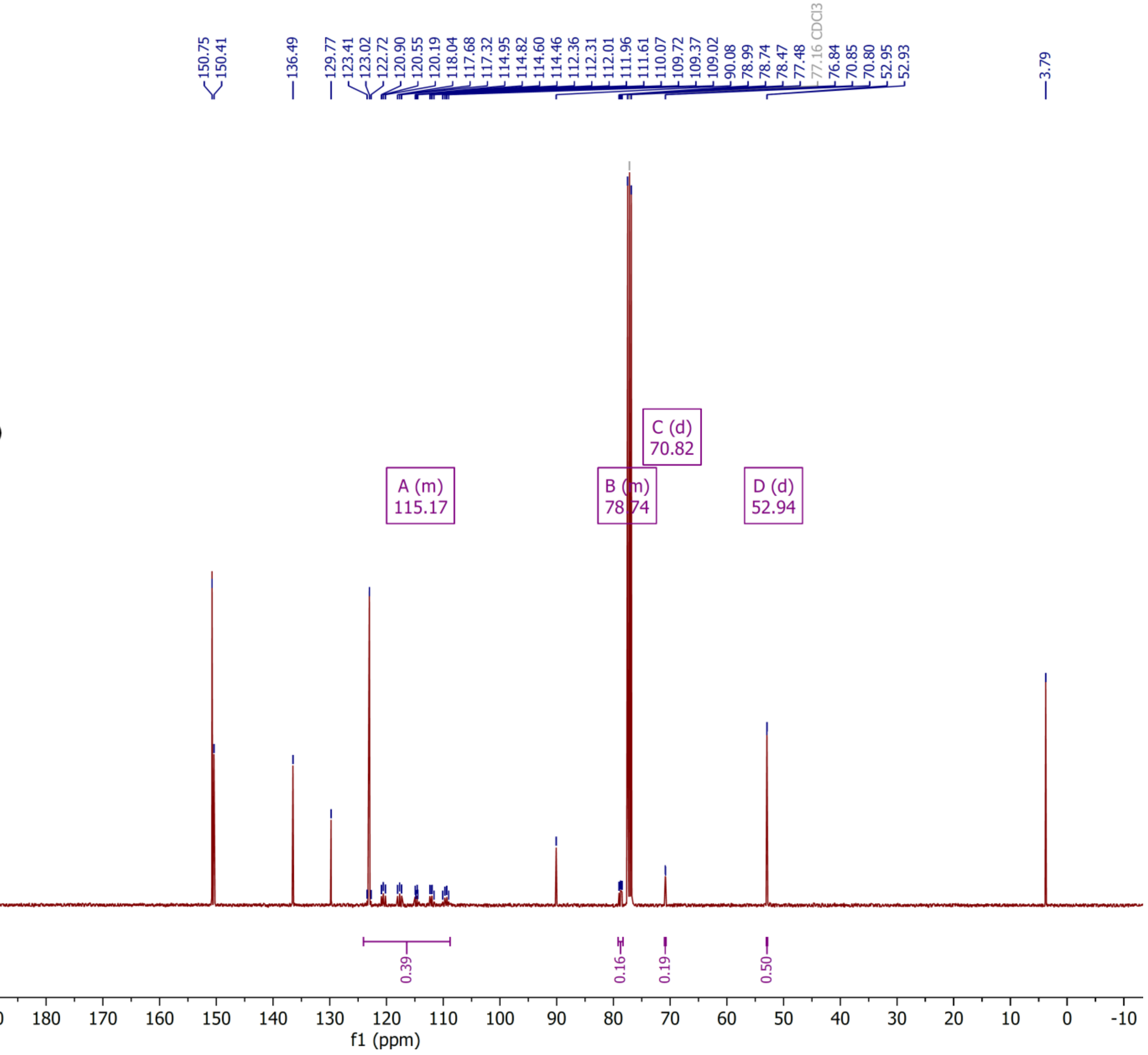

DP-5-46-F

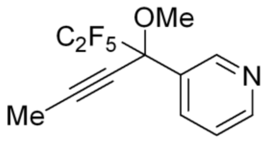

1k

^{19}F NMR (377 MHz, $CDCl_3$)

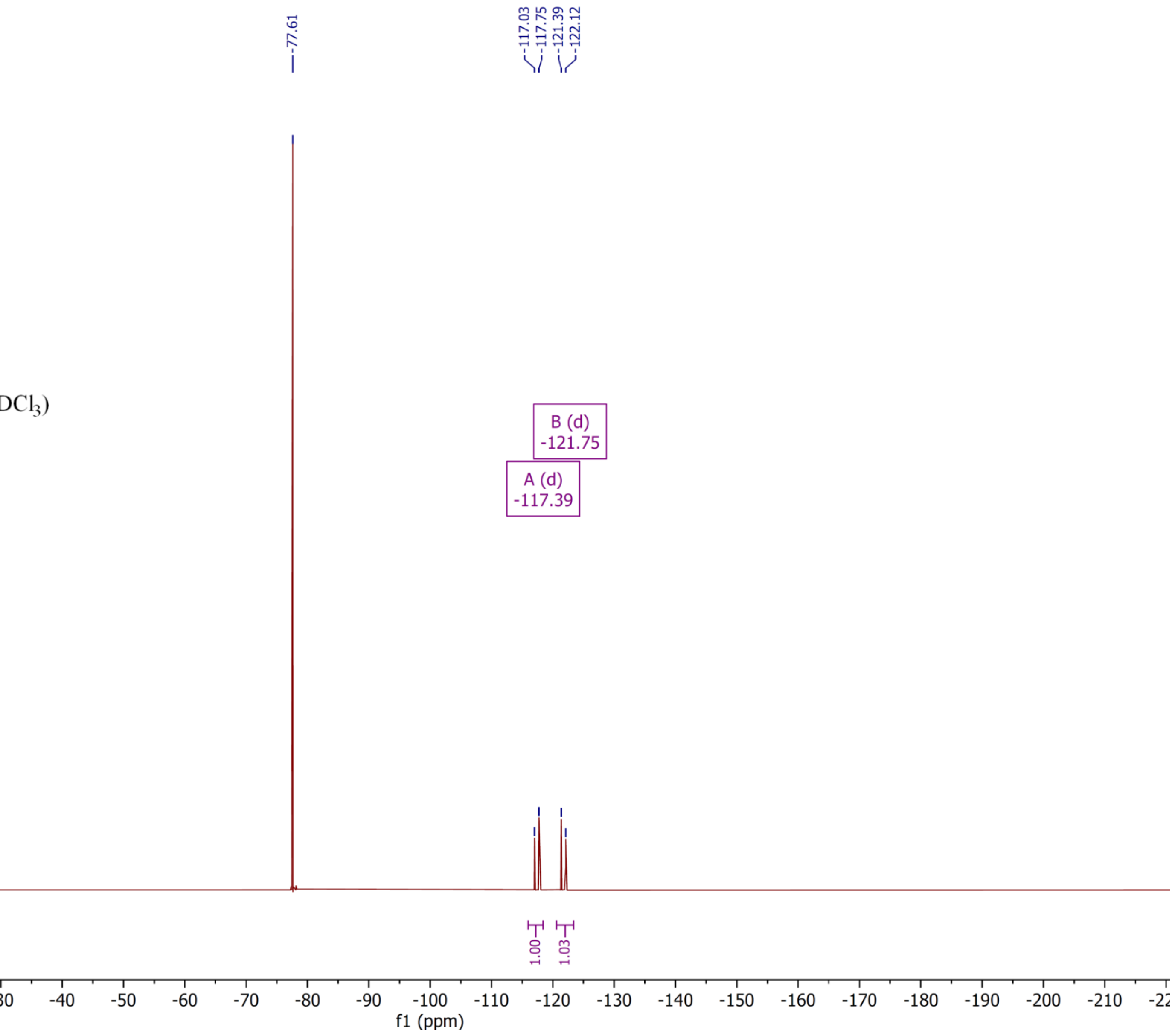

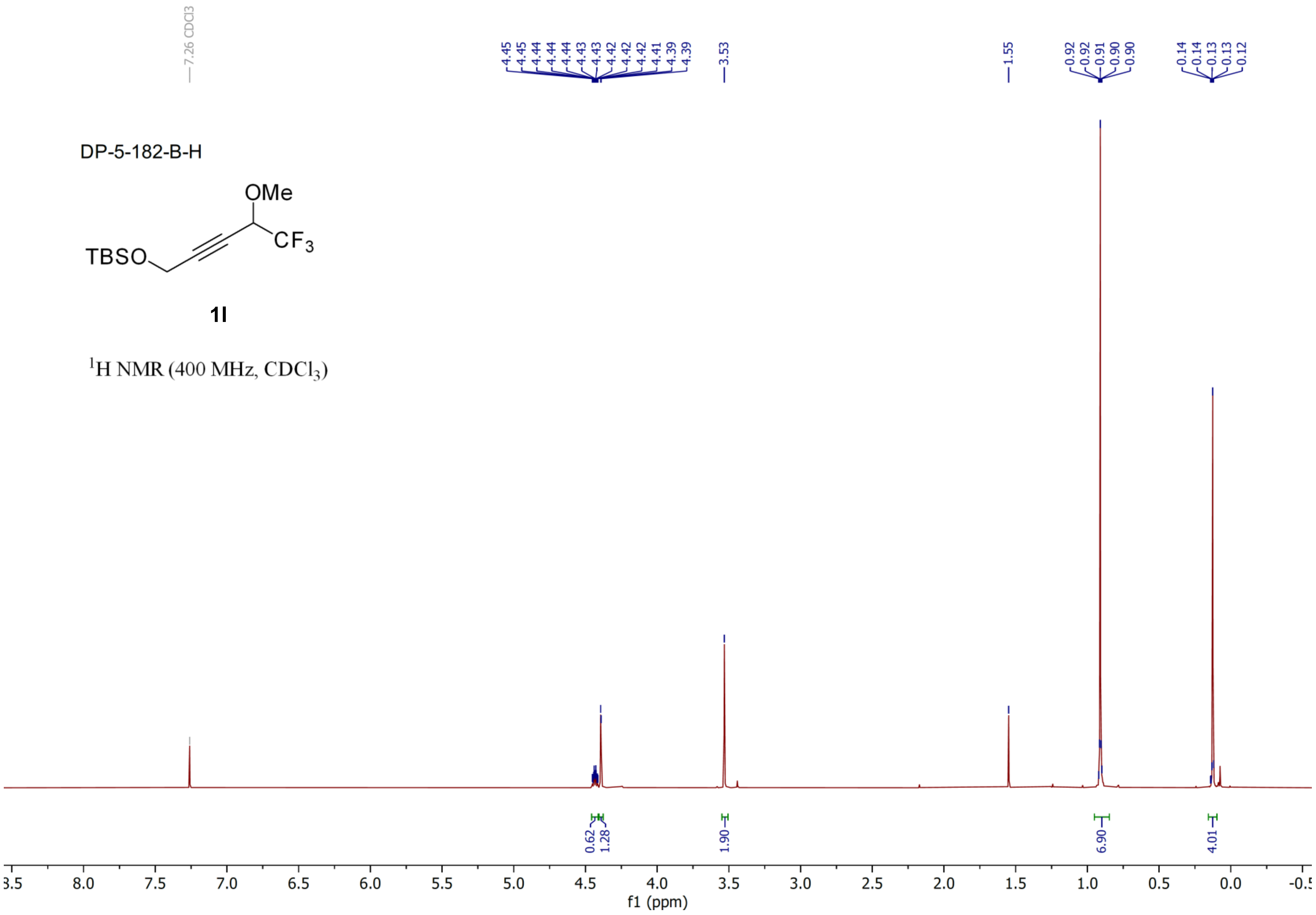

7.26 CDCl3
4.45
4.45
4.44
4.44
4.44
4.43
4.43
4.42
4.42
4.42
4.41
4.39
4.39
3.53
1.55
0.92
0.92
0.91
0.90
0.90
0.14
0.14
0.13
0.13
0.12
DP-5-182-B-H
OMe
TBSO
CF3
1l
1H NMR (400 MHz, CDCl3)
0.62
1.28
1.90
6.90
4.01
8.0
7.5
7.0
6.5
6.0
5.5
5.0
4.5
4.0
3.5
3.0
2.5
2.0
1.5
1.0
0.5
0.0
f1 (ppm)

DP-5-182-B-C

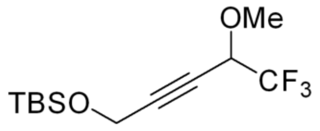

1l

^{13}C NMR (101 MHz, $CDCl_3$)

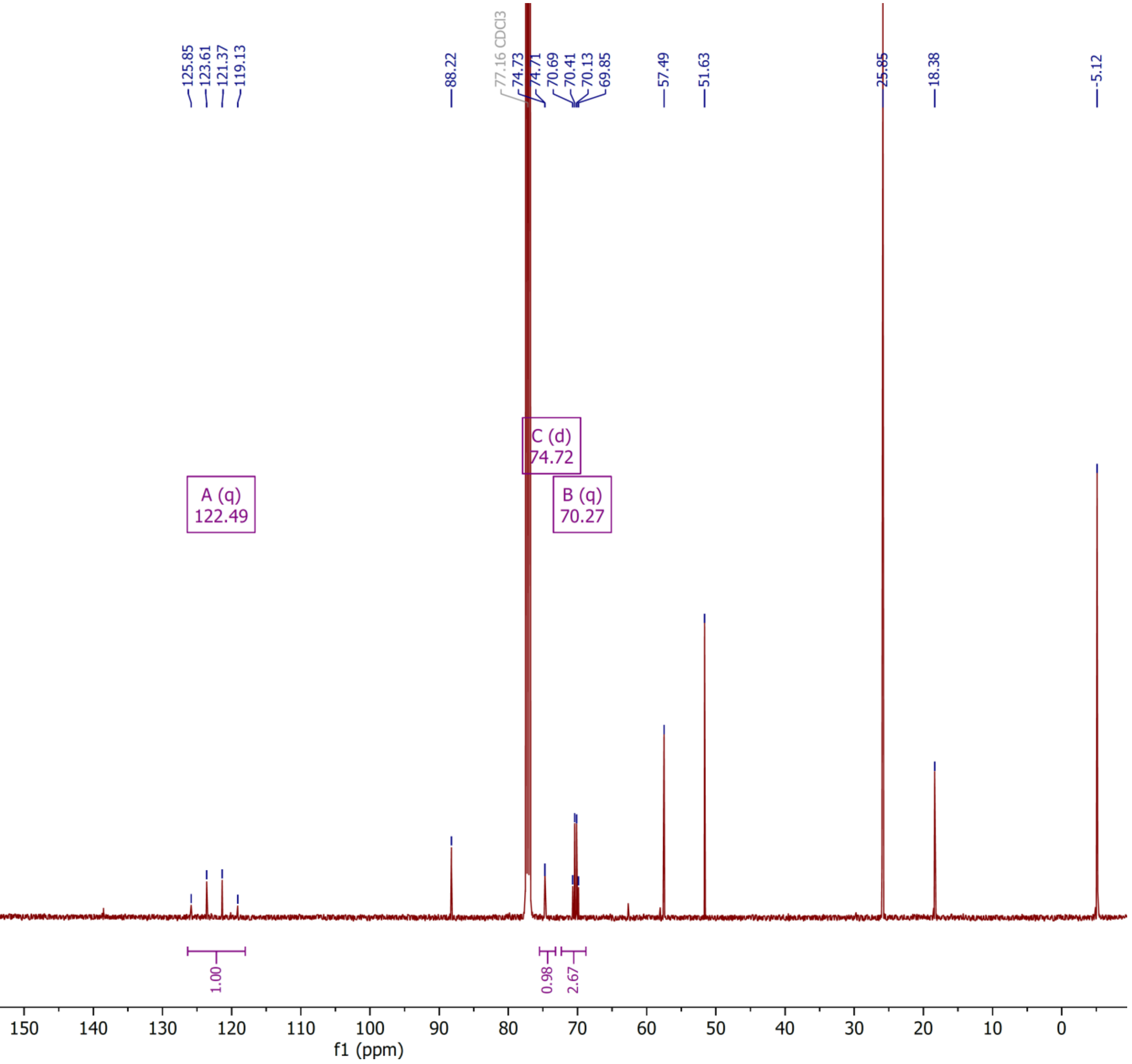

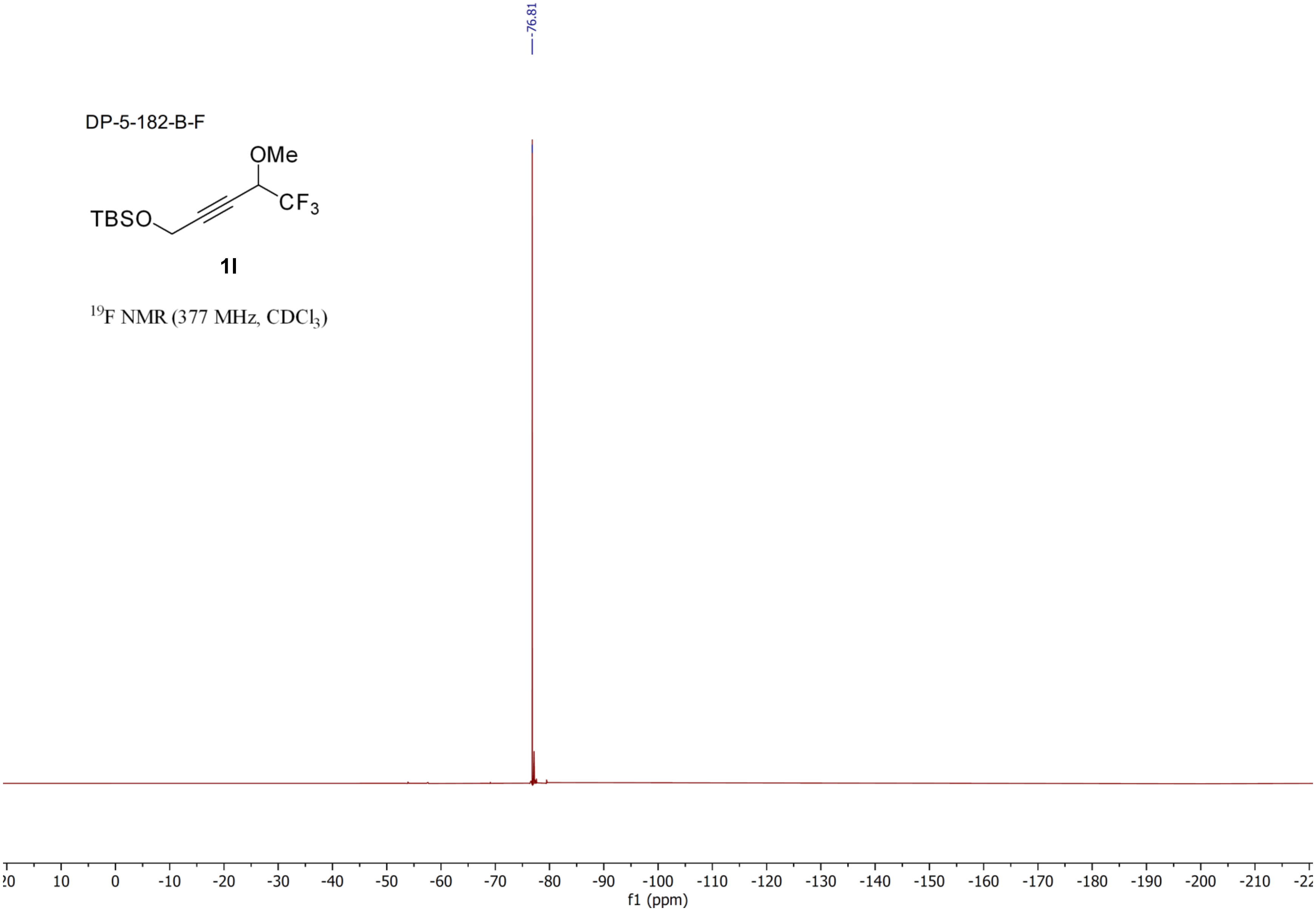
-76.81
DP-5-182-B-F
OMe
TBSO
CF3
1l
19F NMR (377 MHz, CDCl3)
20
10
0
-10
-20
-30
-40
-50
-60
-70
-80
-90
-100
-110
-120
-130
-140
-150
-160
-170
-180
-190
-200
-210
f1 (ppm)

DP-5-55-H-500mhz

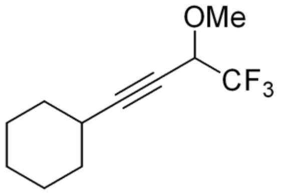

1m

^{1}H NMR (500 MHz, $CDCl_3$)

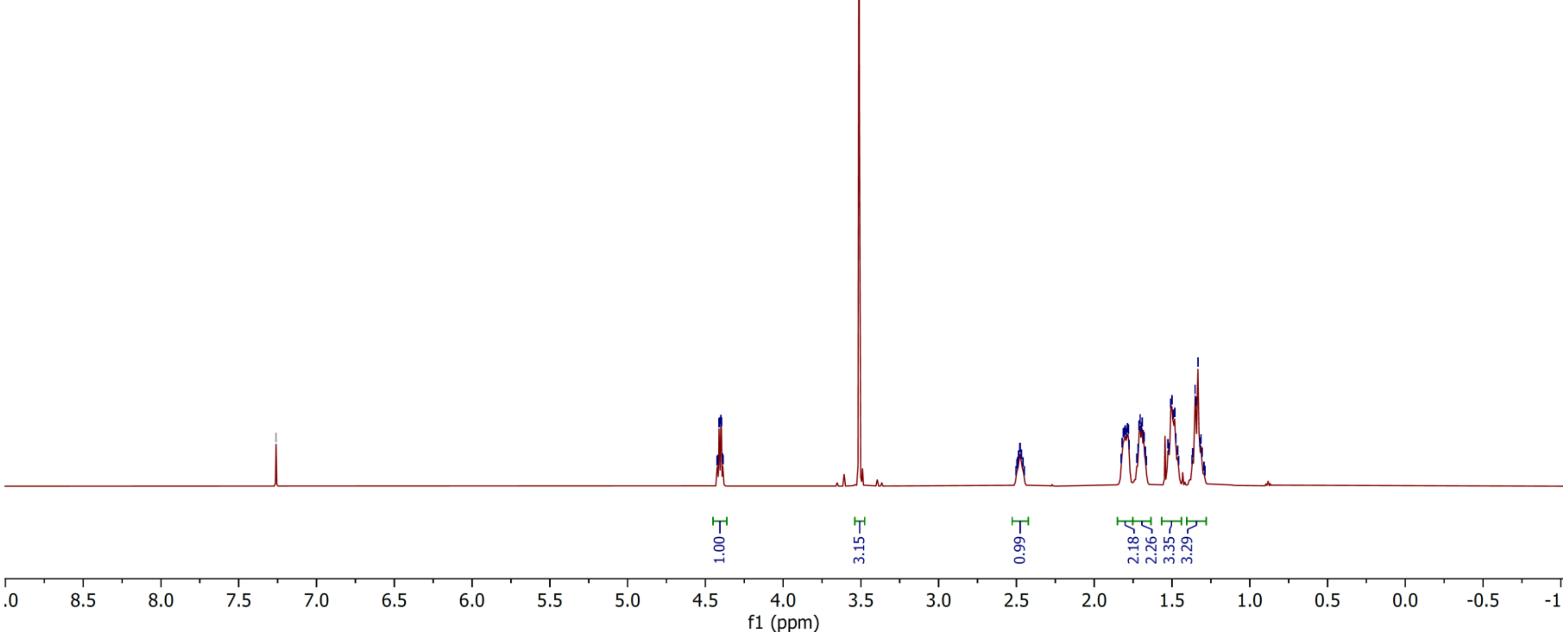

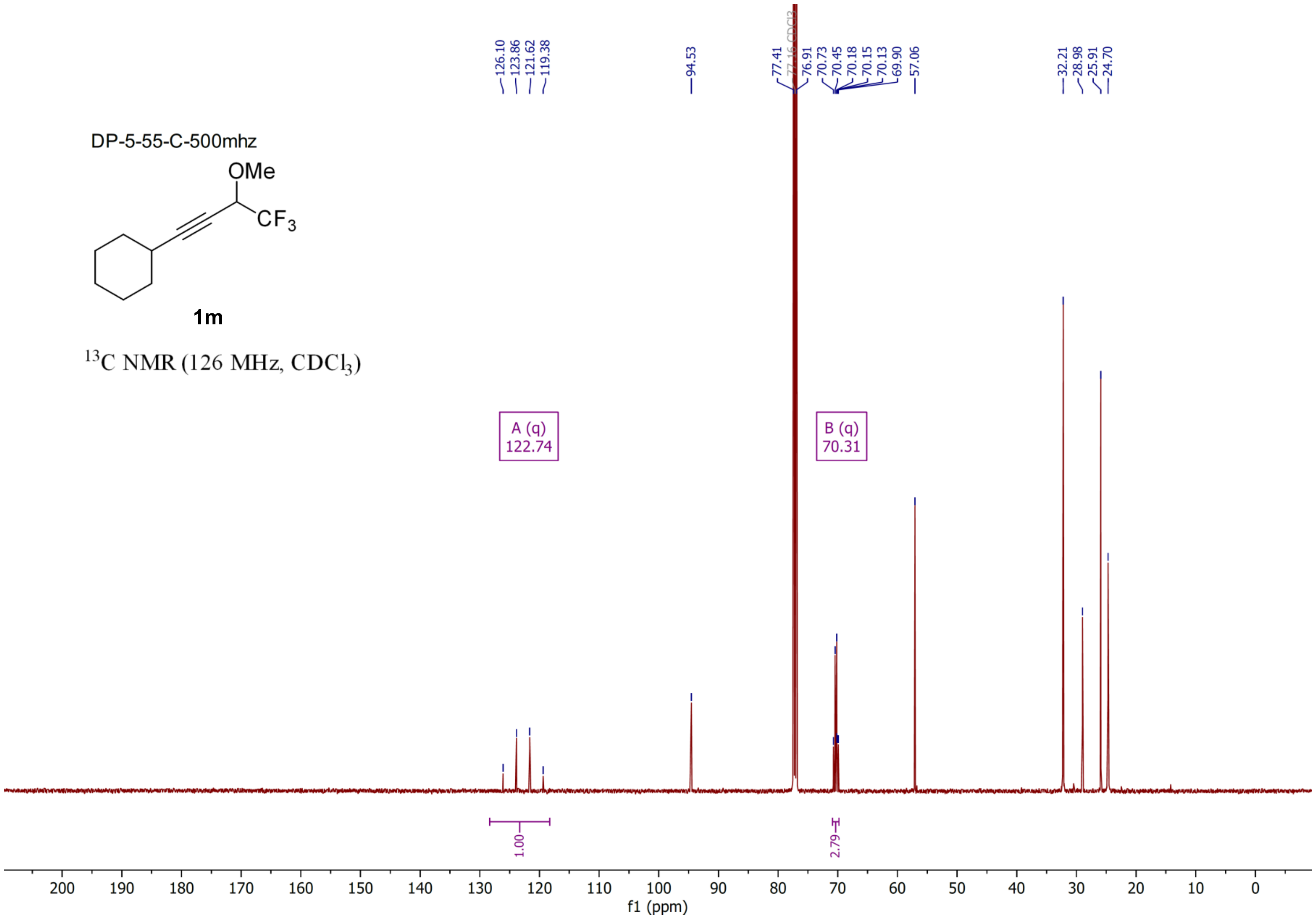
DP-5-55-C-500mhz
OMe
CF3
1m
13C NMR (126 MHz, CDCl3)
126.10
123.86
121.62
119.38
94.53
77.41
77.16 CDCl3
76.91
70.73
70.45
70.18
70.15
70.13
69.90
57.06
32.21
28.98
25.91
24.70
A (q)
122.74
B (q)
70.31
1.00
2.79
f1 (ppm)

DP-5-55-F

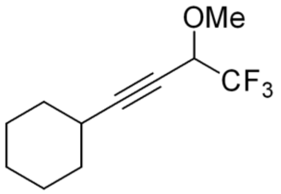

1m

^{19}F NMR (377 MHz, $CDCl_3$)

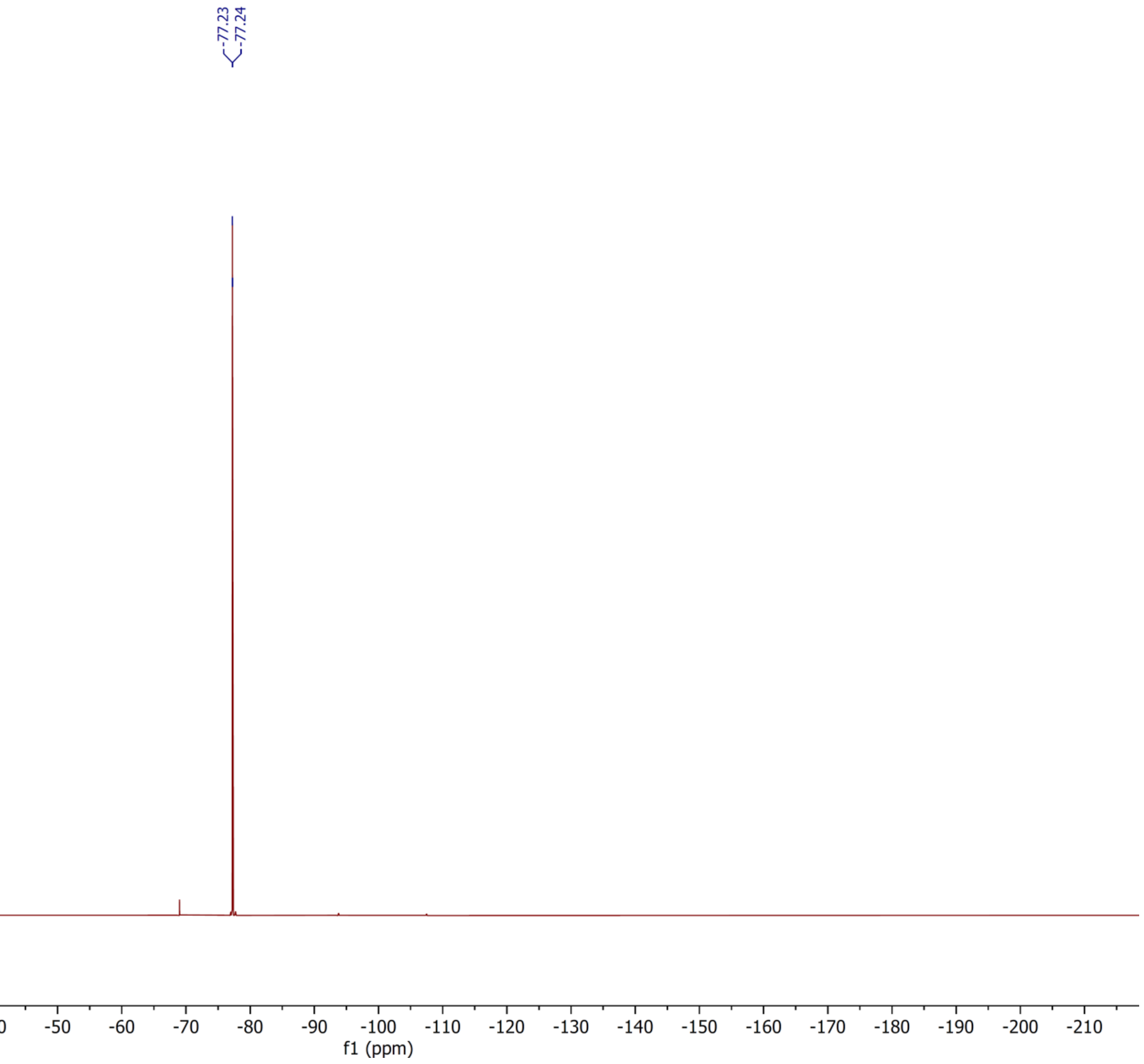

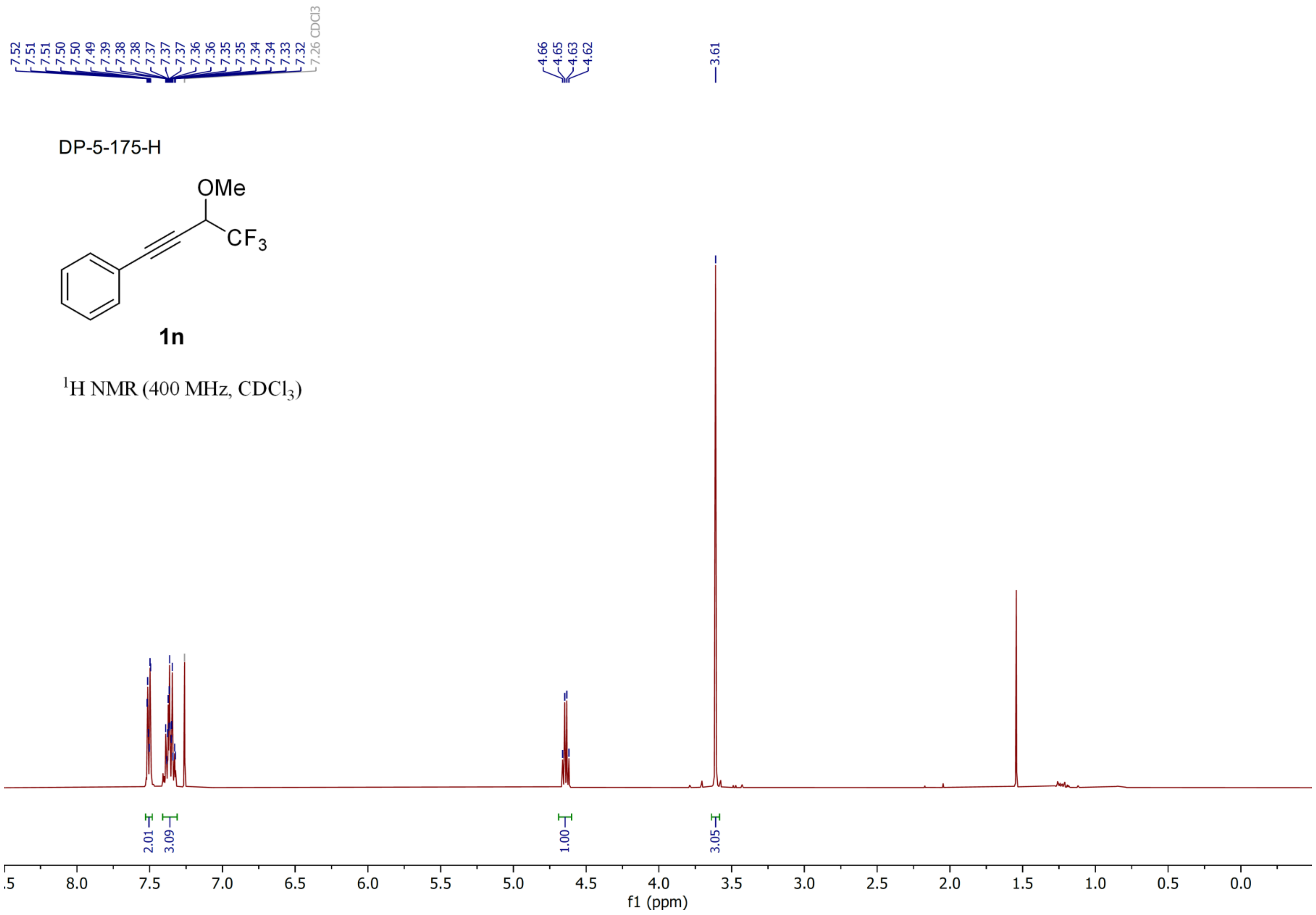

7.52
7.51
7.51
7.50
7.50
7.49
7.39
7.38
7.38
7.37
7.37
7.37
7.36
7.36
7.35
7.35
7.34
7.34
7.33
7.32
7.26 CDCl3
4.66
4.65
4.63
4.62
3.61
DP-5-175-H
OMe
CF3
1n
¹H NMR (400 MHz, CDCl3)
2.01
3.09
1.00
3.05
.5
8.0
7.5
7.0
6.5
6.0
5.5
5.0
4.5
4.0
3.5
3.0
2.5
2.0
1.5
1.0
0.5
0.0
f1 (ppm)

DP-5-175-C

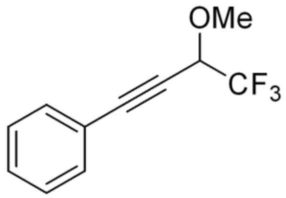

1n

^{13}C NMR (101 MHz, $CDCl_3$)

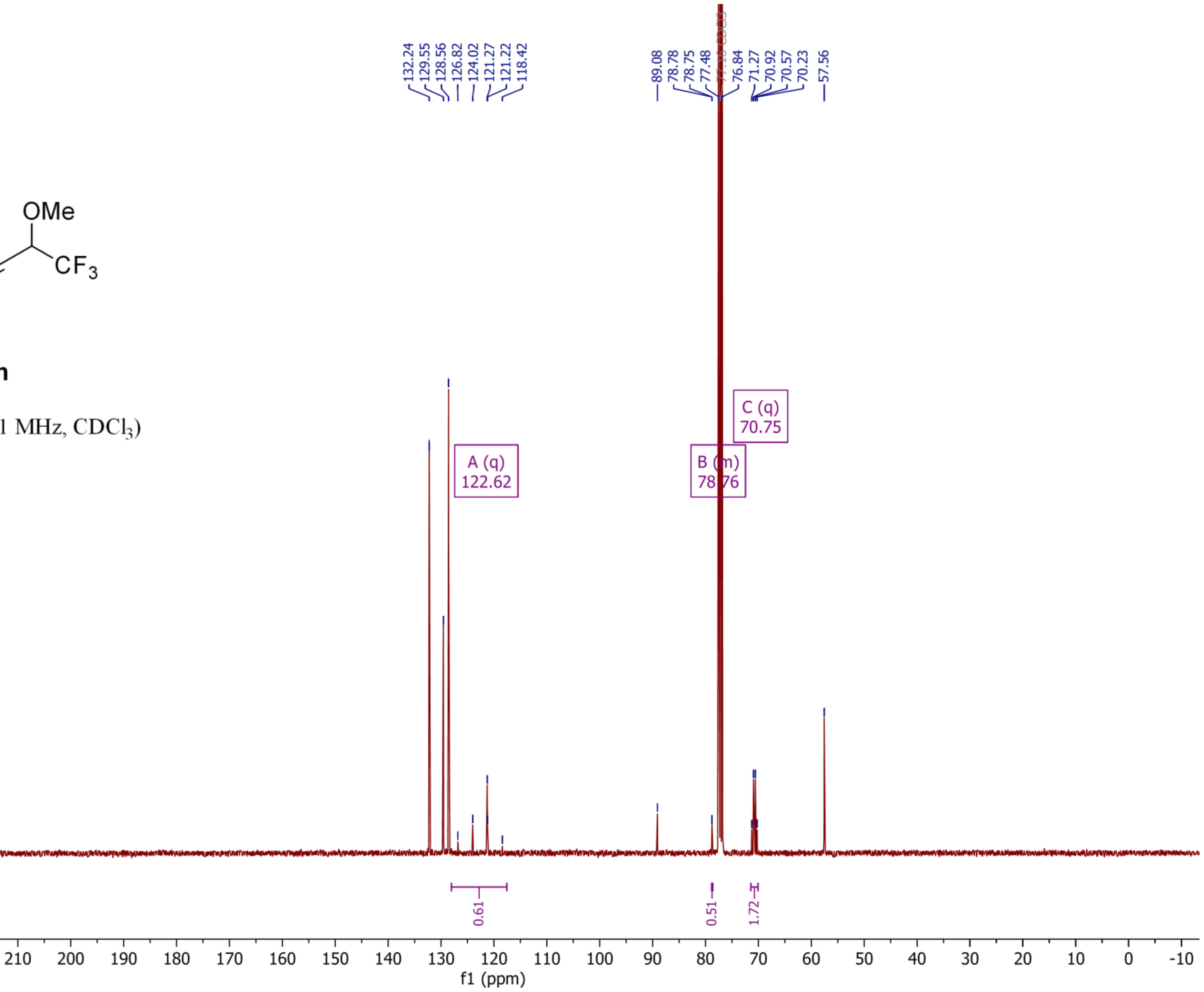

DP-5-175-F

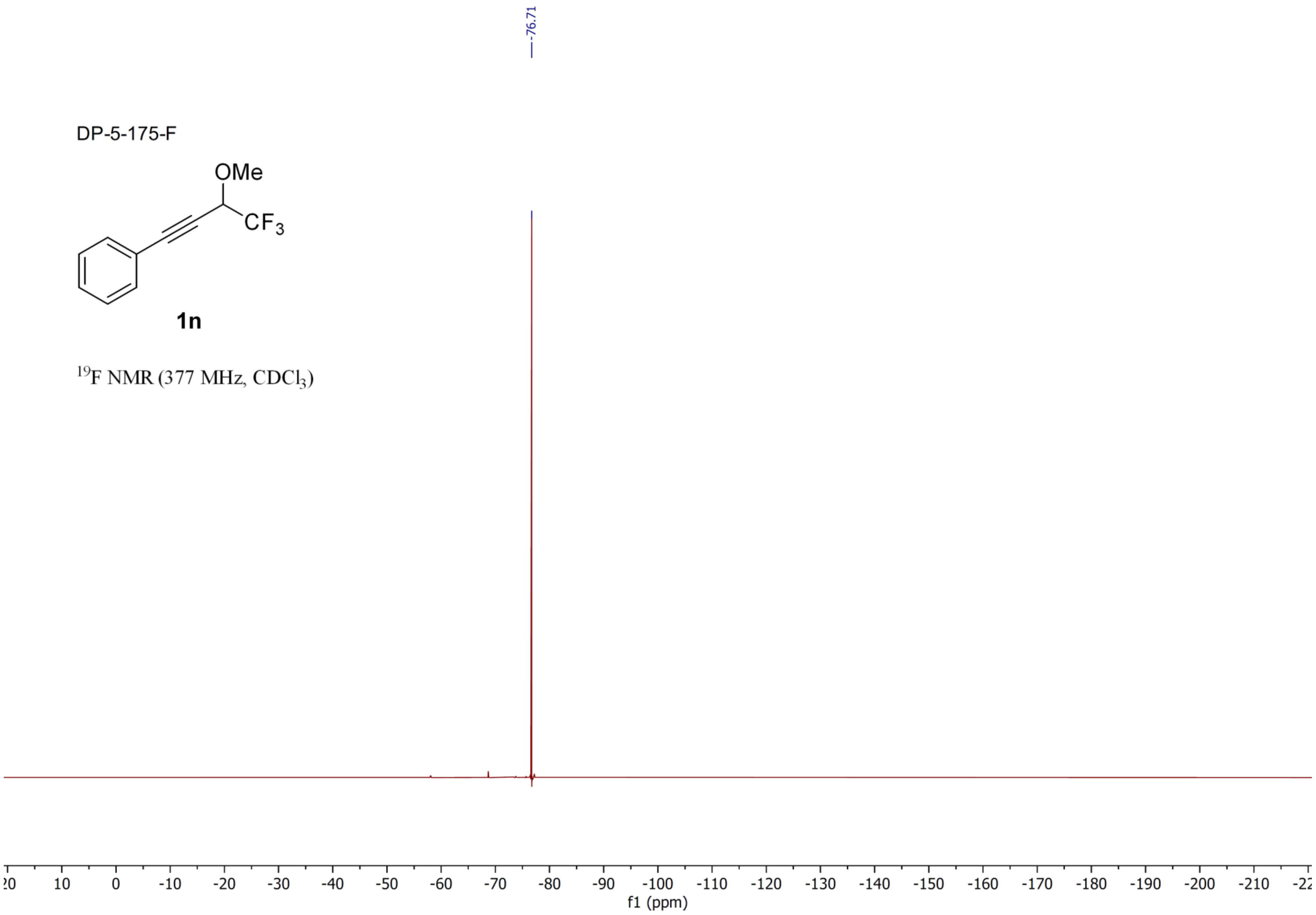

1n

^{19}F NMR (377 MHz, $CDCl_3$)

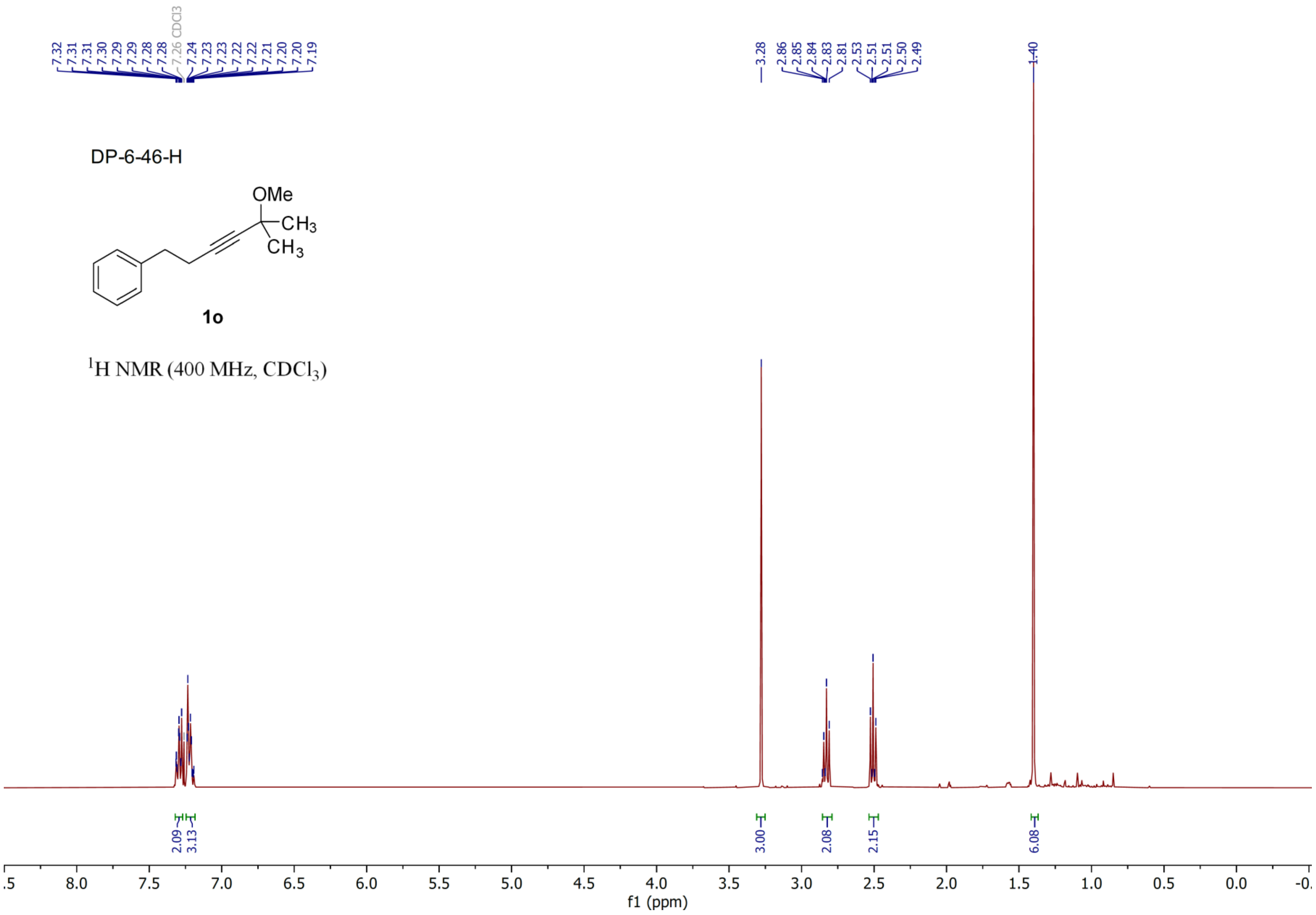
7.32
7.31
7.31
7.30
7.29
7.29
7.28
7.28
7.26 CDCl3
7.24
7.23
7.23
7.22
7.22
7.21
7.20
7.20
7.19
3.28
2.86
2.85
2.84
2.83
2.81
2.53
2.51
2.51
2.50
2.49
1.40
DP-6-46-H
OMe
CH3
CH3
1o
1H NMR (400 MHz, CDCl3)
2.09
3.13
3.00
2.08
2.15
6.08
.5
8.0
7.5
7.0
6.5
6.0
5.5
5.0
4.5
4.0
3.5
3.0
2.5
2.0
1.5
1.0
0.5
0.0
-0.
f1 (ppm)

DP-6-46-C

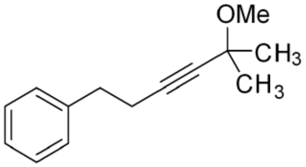

1o

^{13}C NMR (101 MHz, $CDCl_3$)

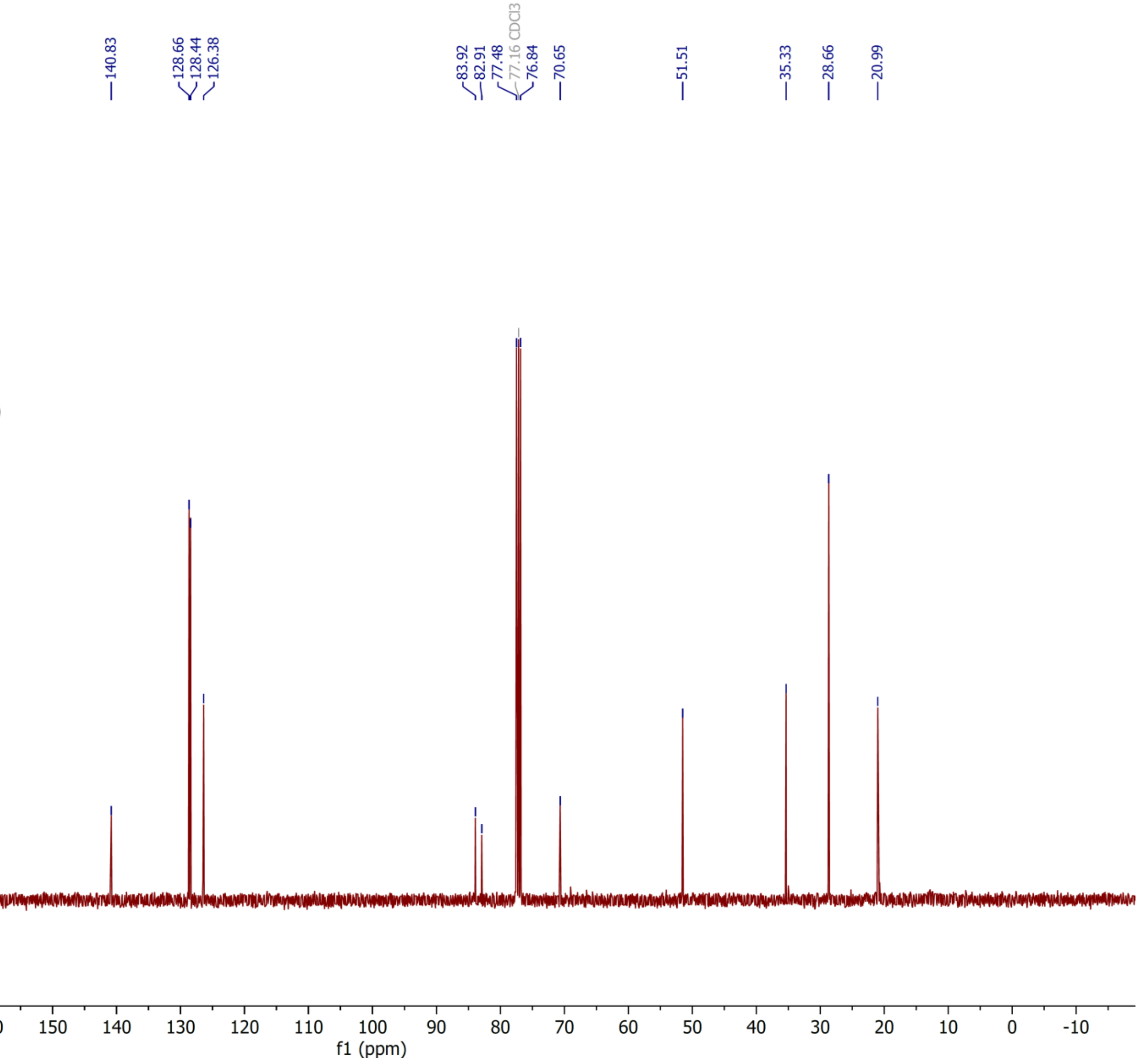

DP-5-43-H

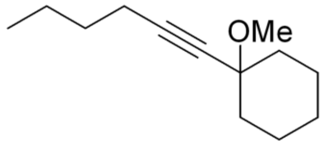

1p

^{1}H NMR (400 MHz, $CDCl_3$)

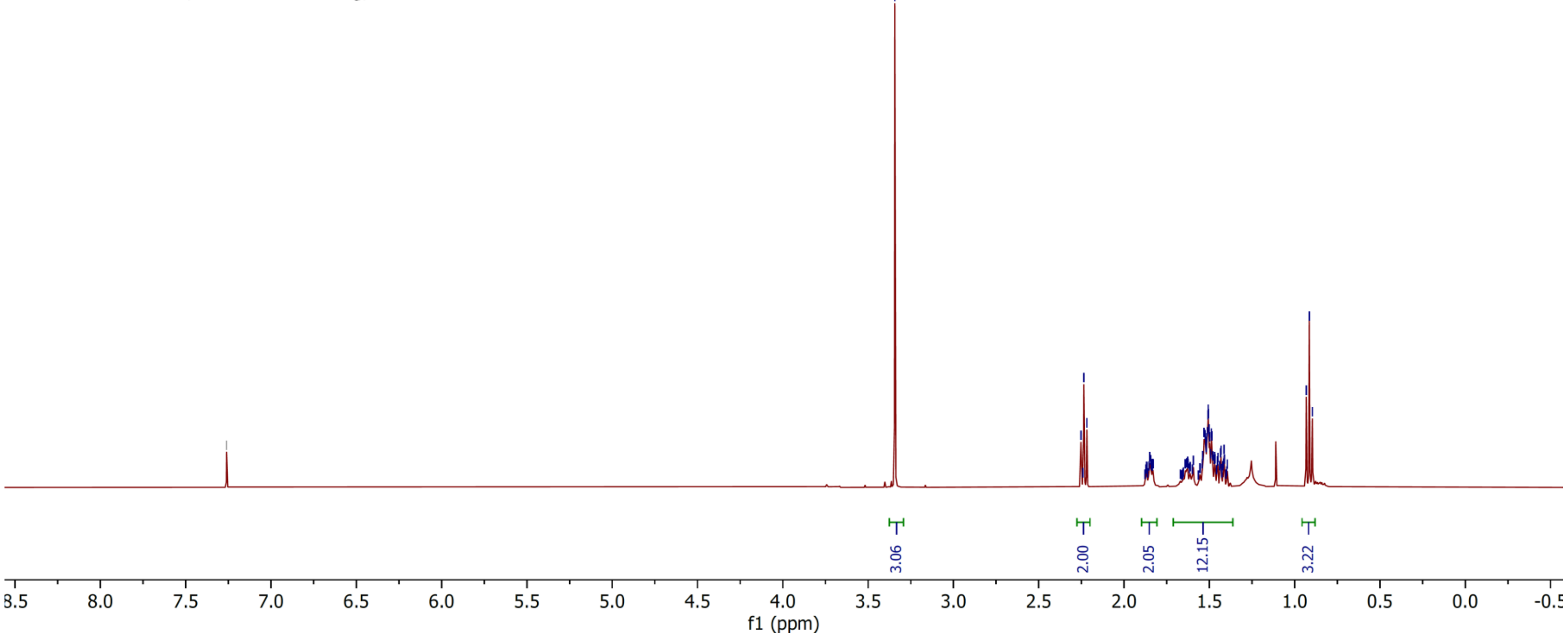

DP-5-43-C

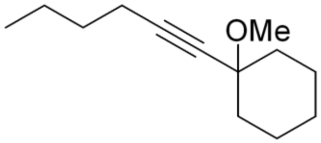

1p

^{13}C NMR (101 MHz, $CDCl_3$)

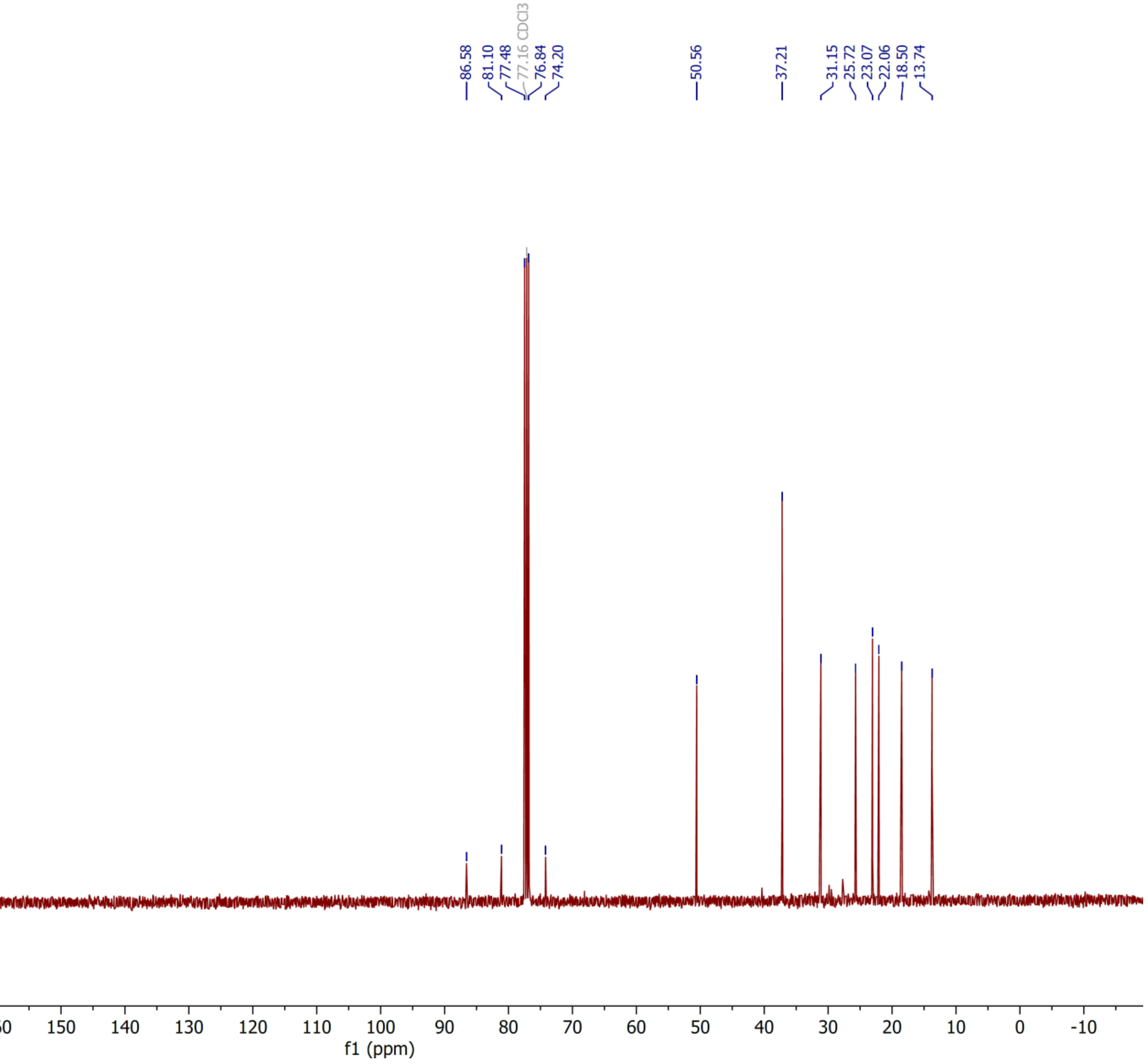

DP-6-48-H-500mhz

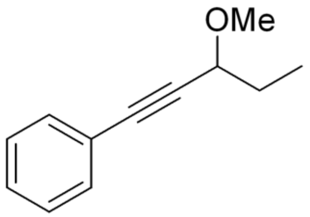

1q

^{1}H NMR (500 MHz, $CDCl_3$)

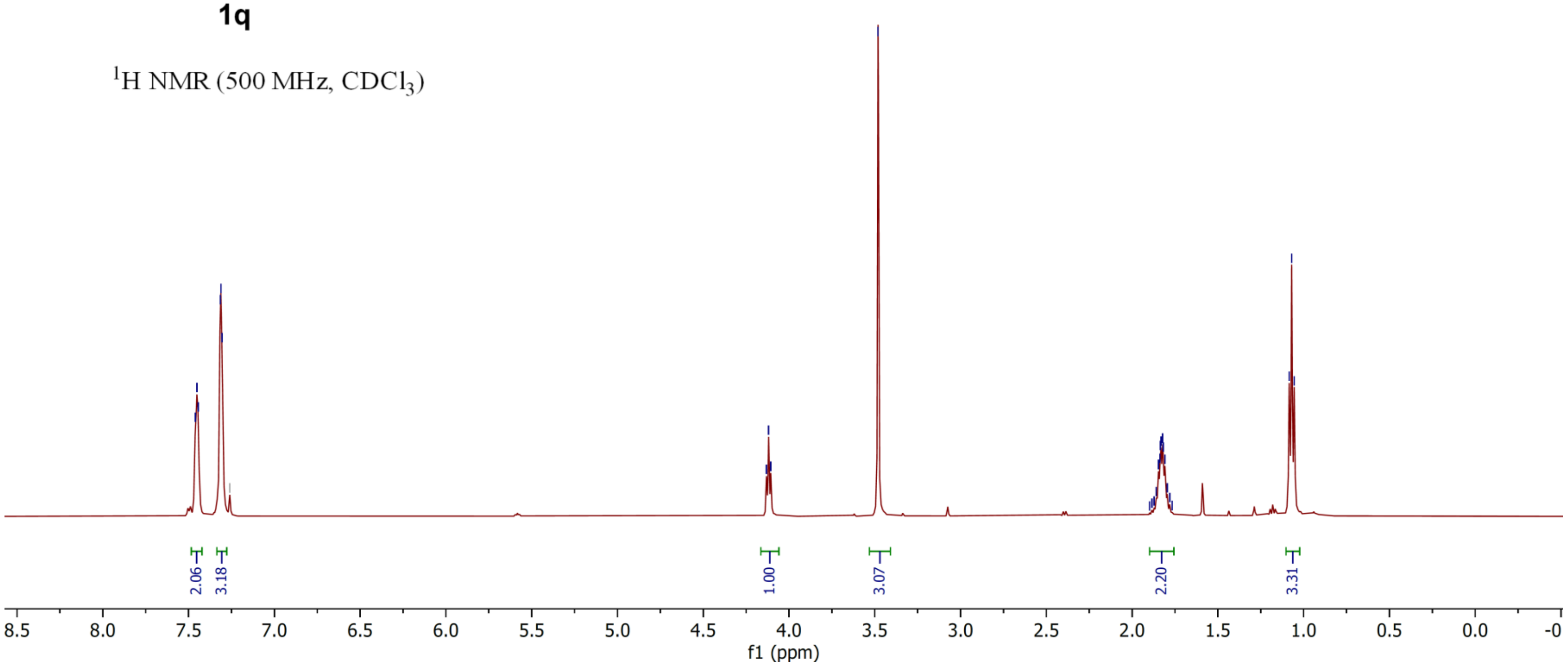

DP-6-48-C-500mhz

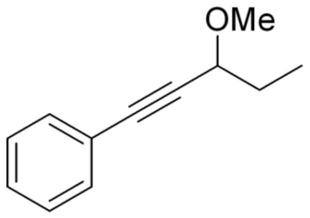

1q

^{13}C NMR (126 MHz, $CDCl_3$)

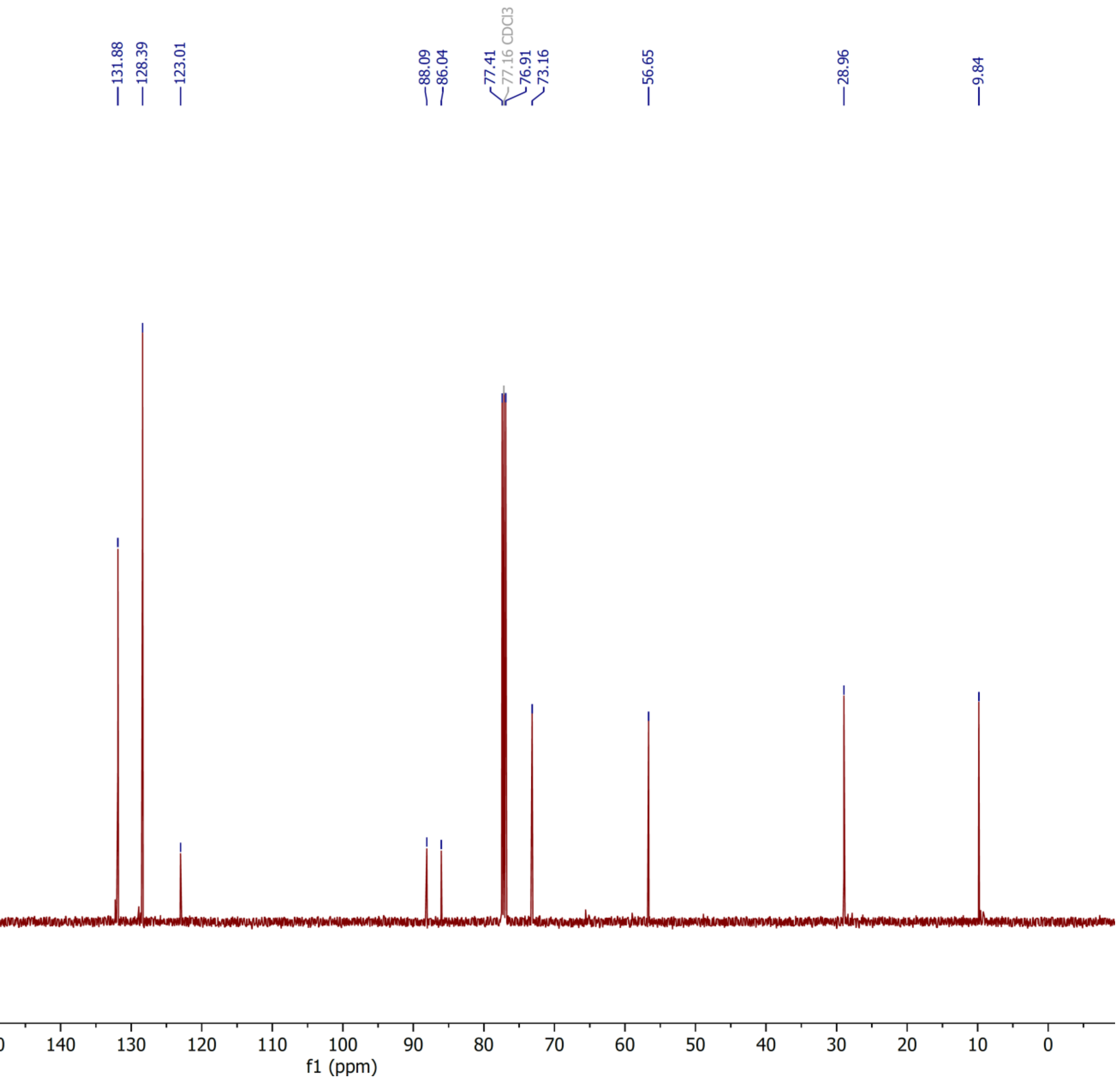

ABL-D-19-H

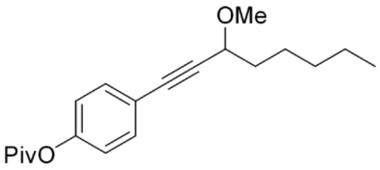

1r

^{1}H NMR (400 MHz, $CDCl_3$)

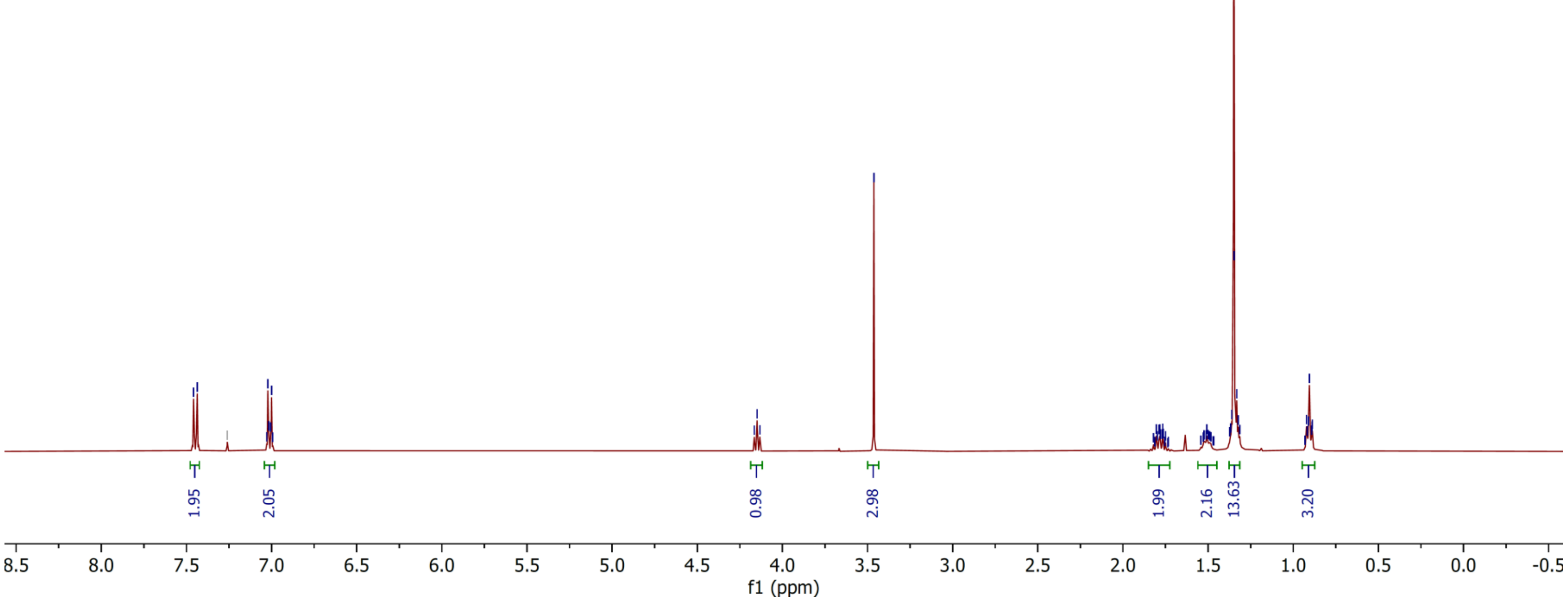

ABL-D-19-C

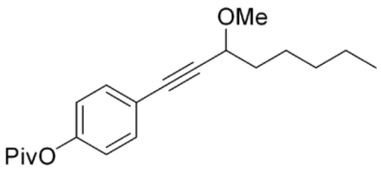

1r

^{13}C NMR (101 MHz, $CDCl_3$)

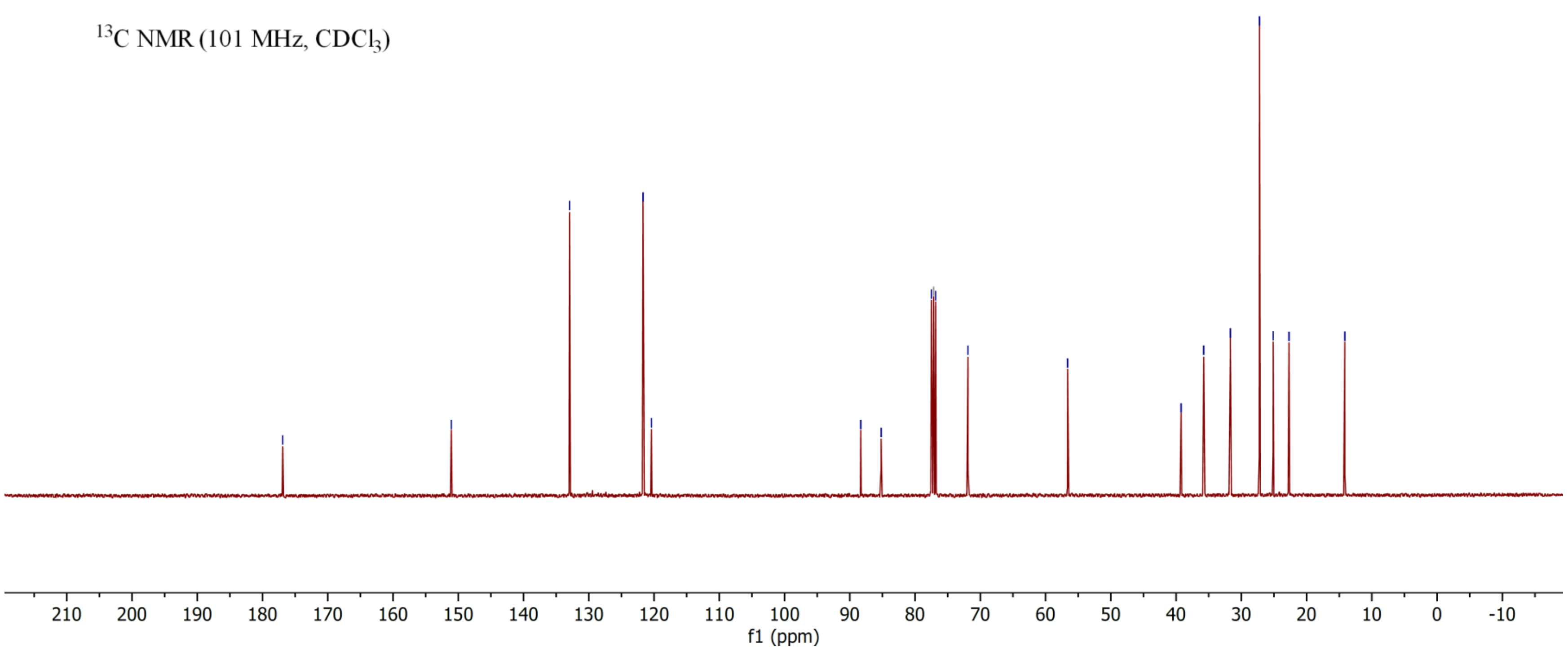

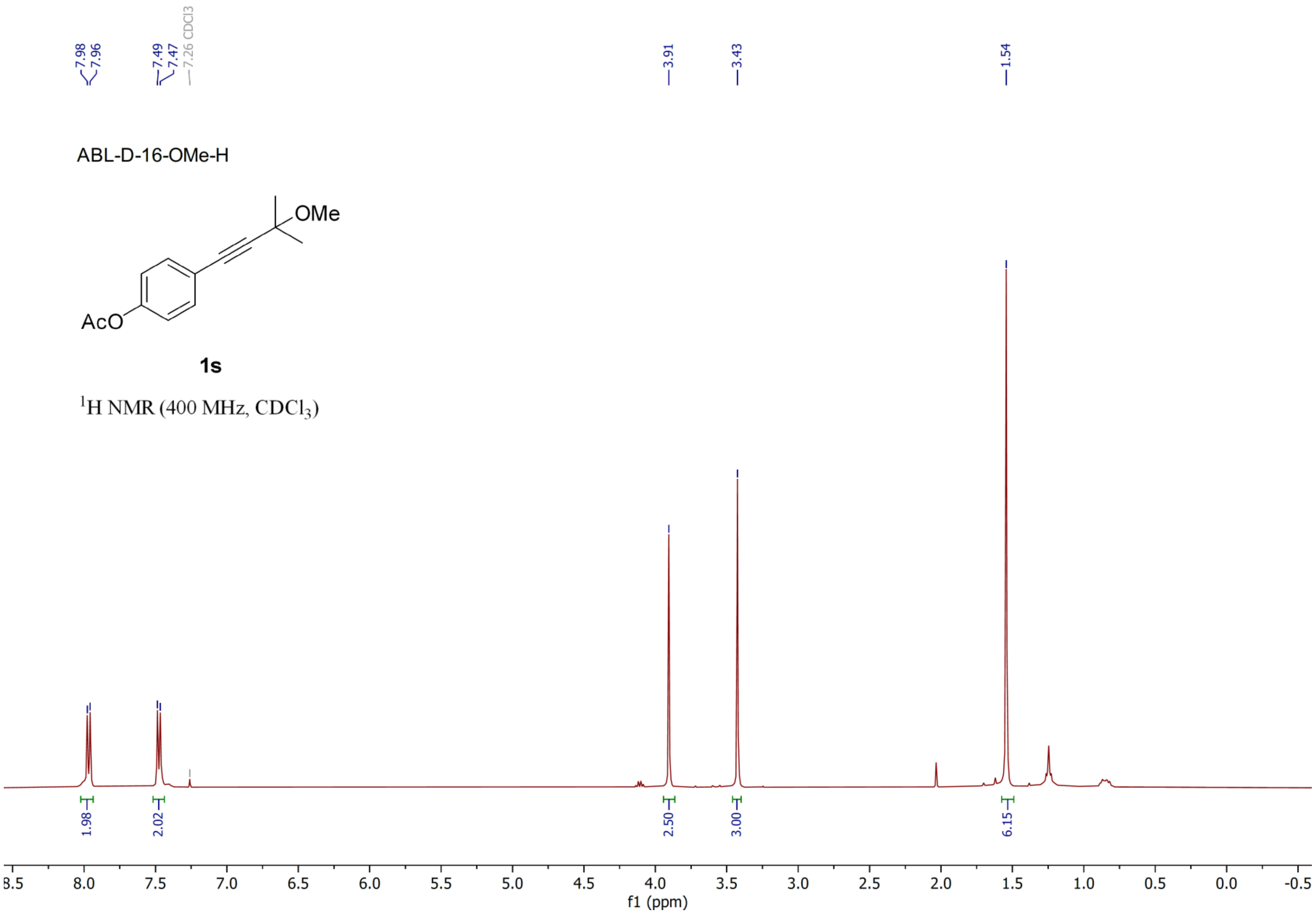
7.98
7.96
7.49
7.47
7.26 CDCl3
3.91
3.43
1.54
ABL-D-16-OMe-H
OMe
AcO
1s
^{1}H NMR (400 MHz, CDCl$_3$)
1.98
2.02
2.50
3.00
6.15
8.5
8.0
7.5
7.0
6.5
6.0
5.5
5.0
4.5
4.0
3.5
3.0
2.5
2.0
1.5
1.0
0.5
0.0
-0.5
f1 (ppm)

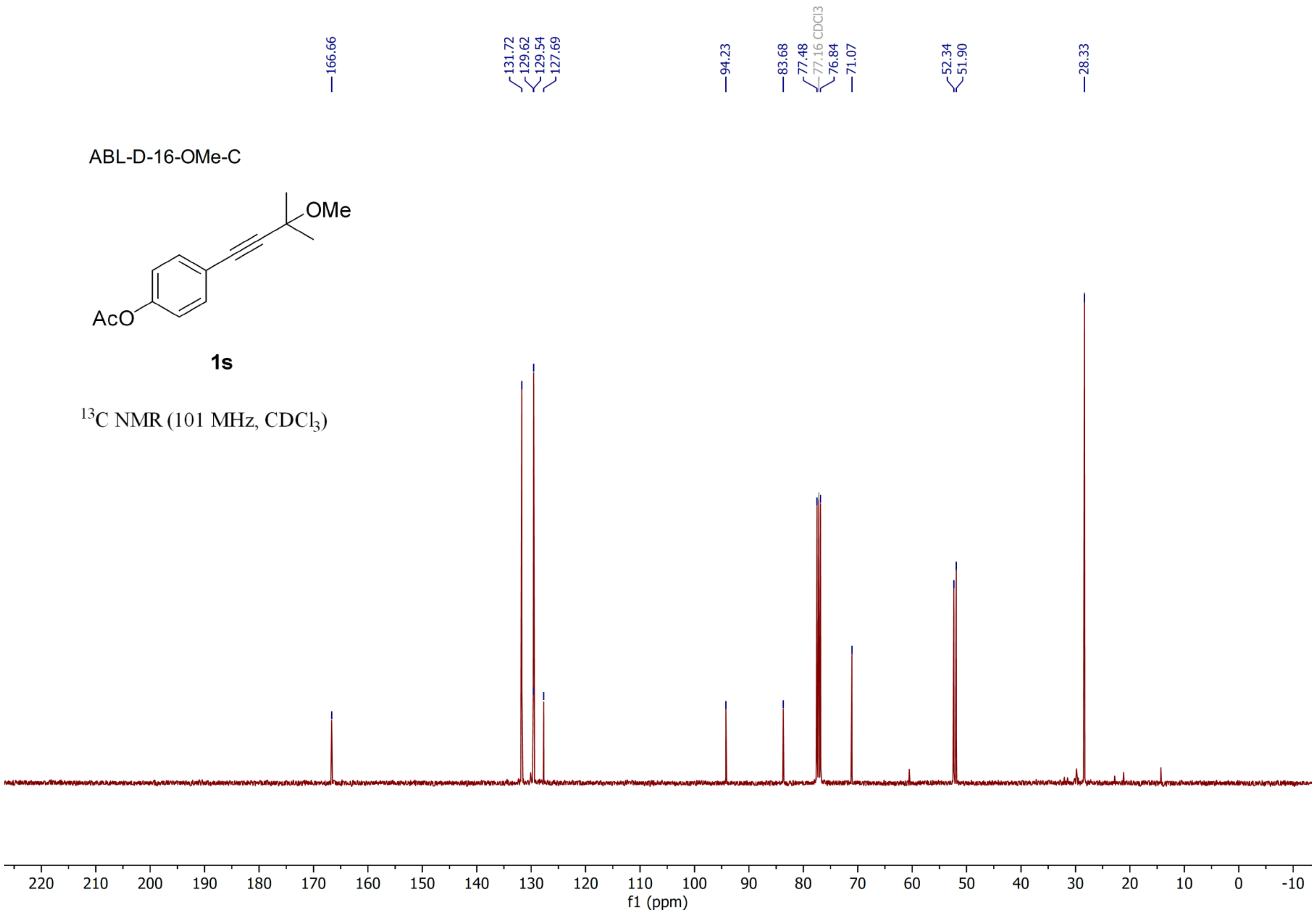

ABL-D-16-OMe-C

1s

^{13}C NMR (101 MHz, $CDCl_3$)

^{1}H and ^{13}C NMR spectra of compounds <u>4a</u> – <u>4i</u>:

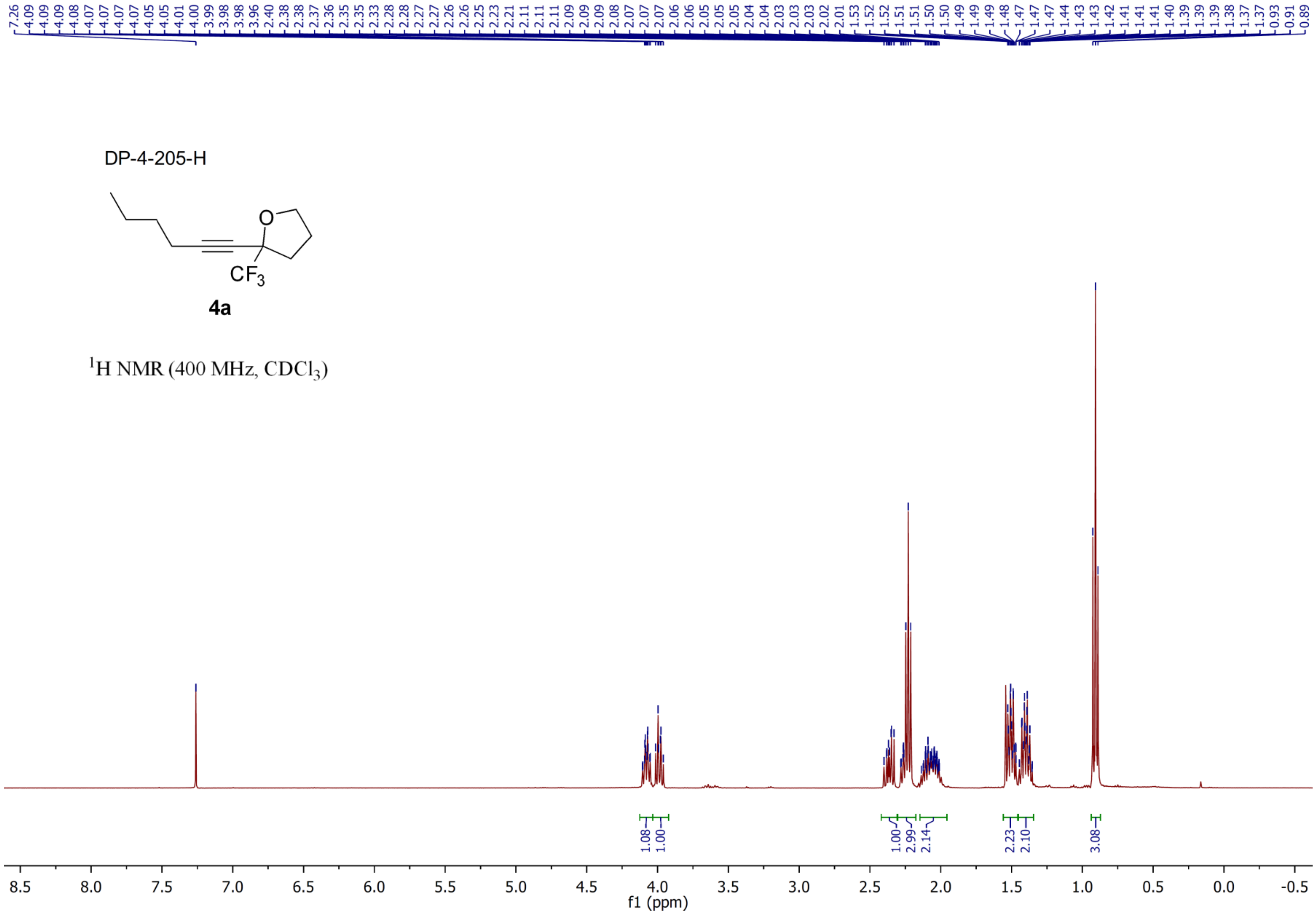

DP-4-205-C

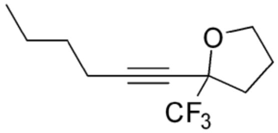

4a

^{13}C NMR (101 MHz, $CDCl_3$)

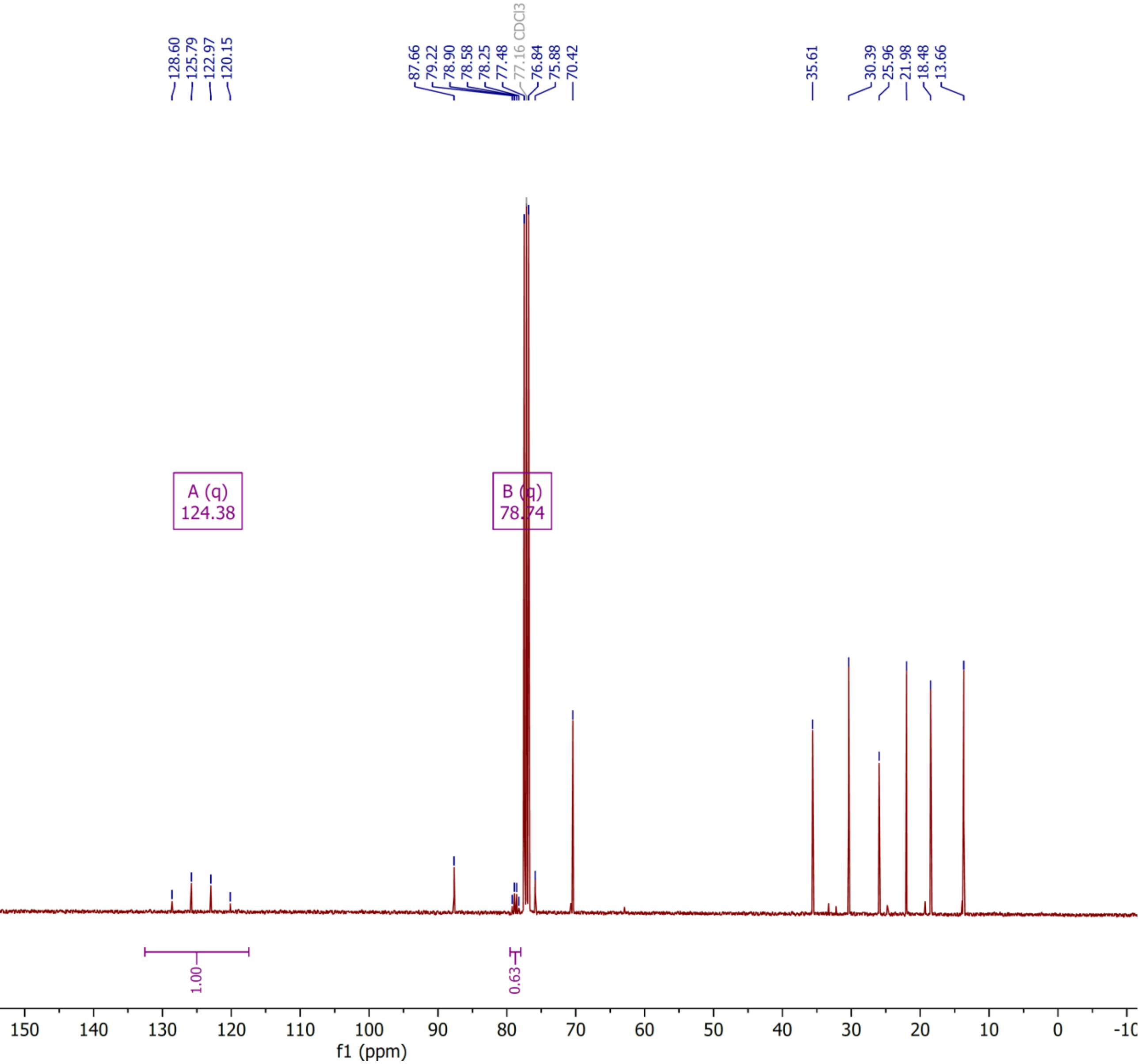

DP-4-205-F

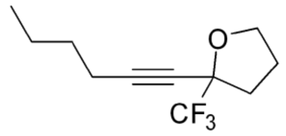

4a

^{19}F NMR (377 MHz, $CDCl_3$)

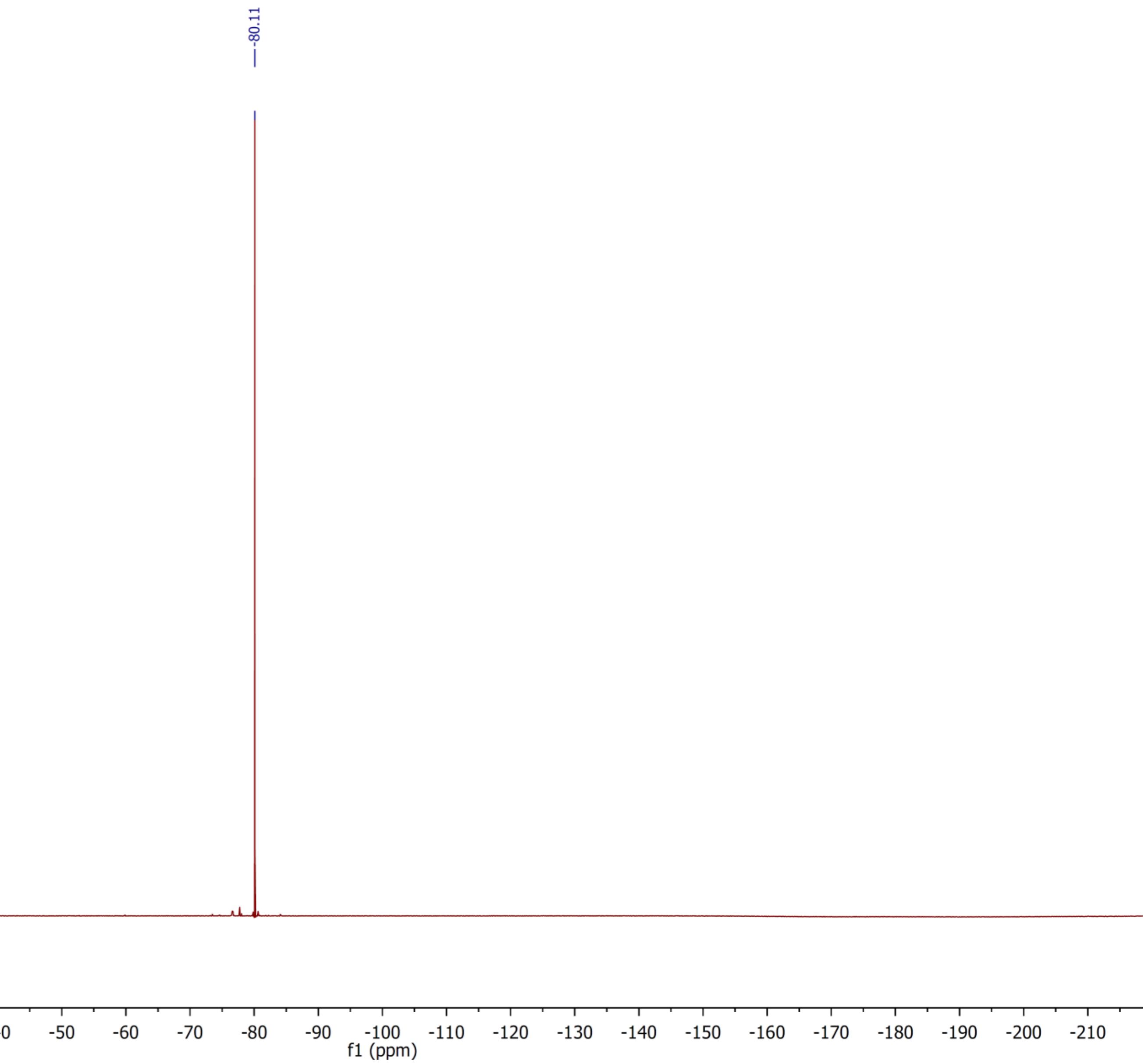

DP-2-153-H

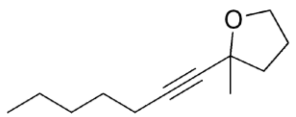

4b

^{1}H NMR (400 MHz, $CDCl_3$)

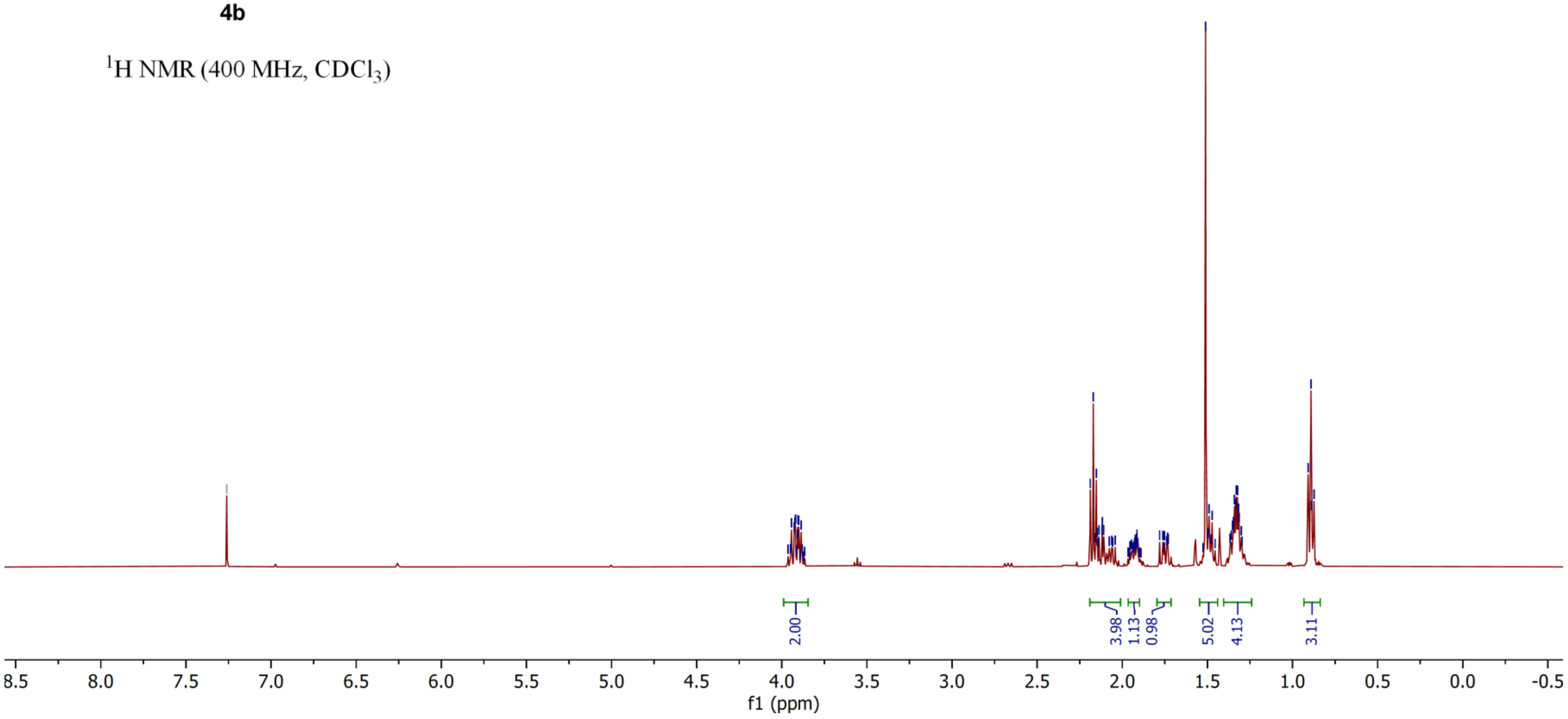

DP-2-153-C

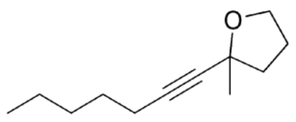

4b

^{13}C NMR (101 MHz, $CDCl_3$)

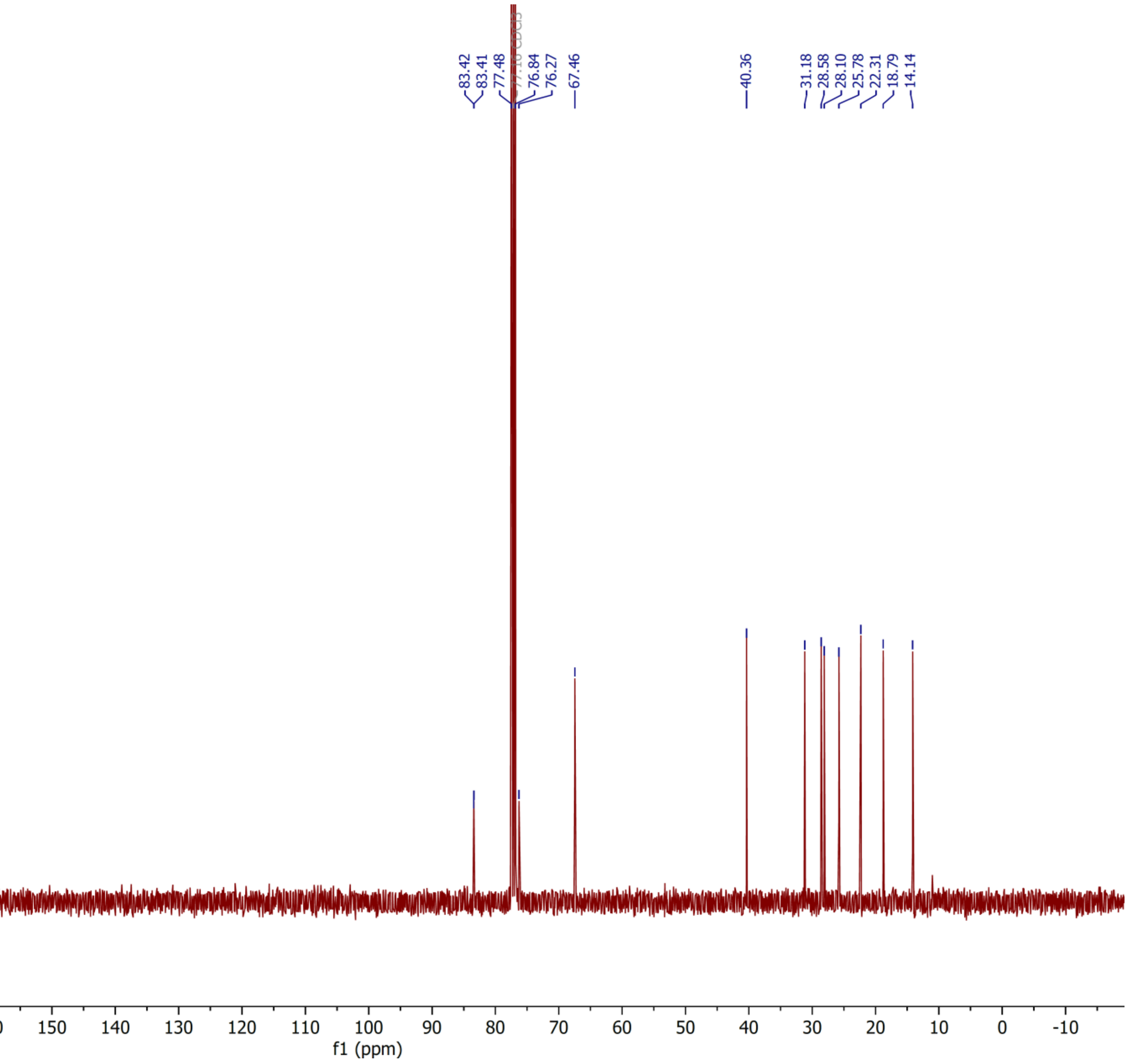

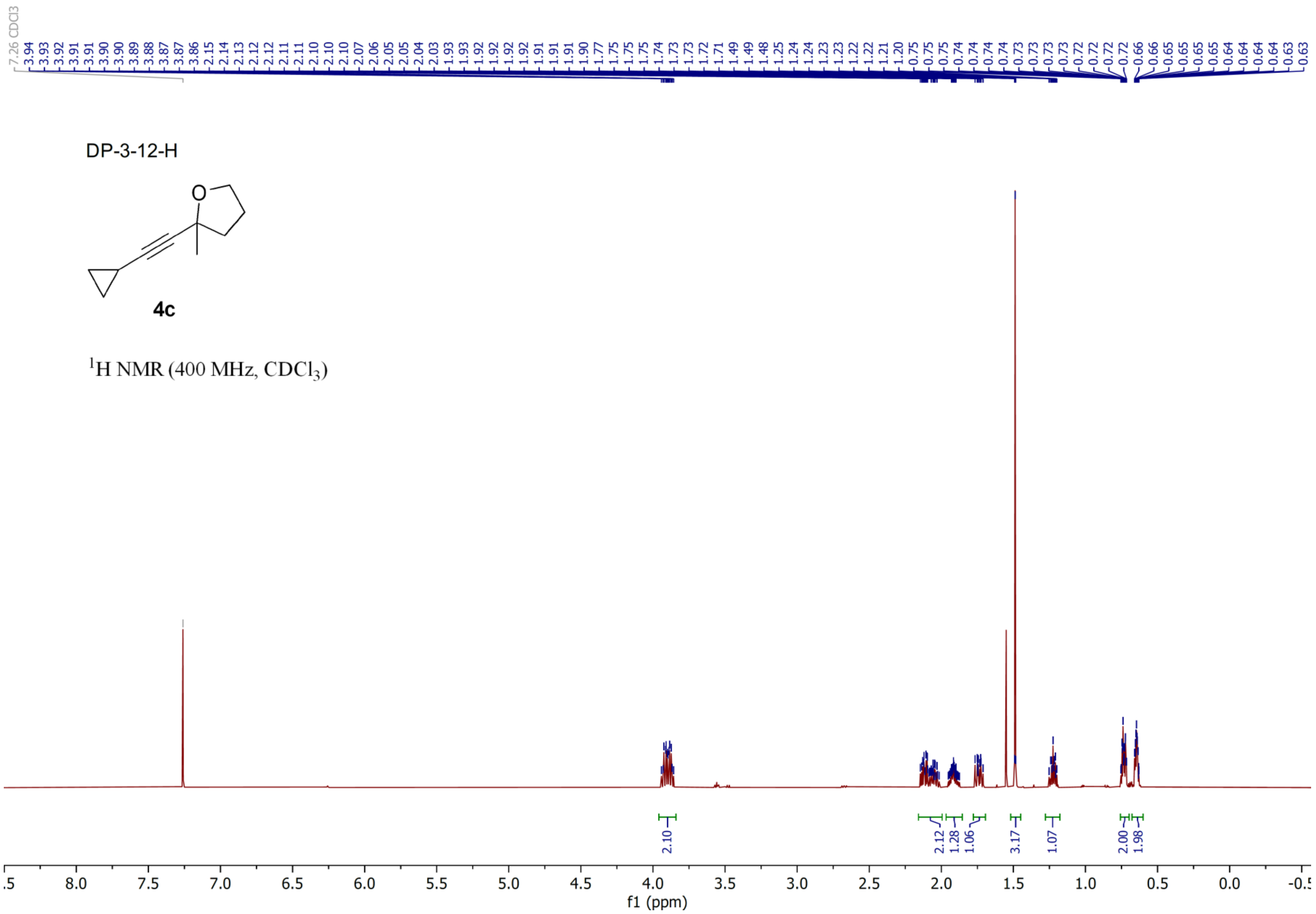
DP-3-12-H
4c
^{1}H NMR (400 MHz, CDCl$_3$)
f1 (ppm)

DP-3-12-C

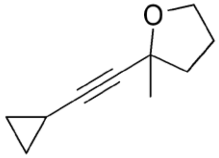

4c

^{13}C NMR (101 MHz, $CDCl_3$)

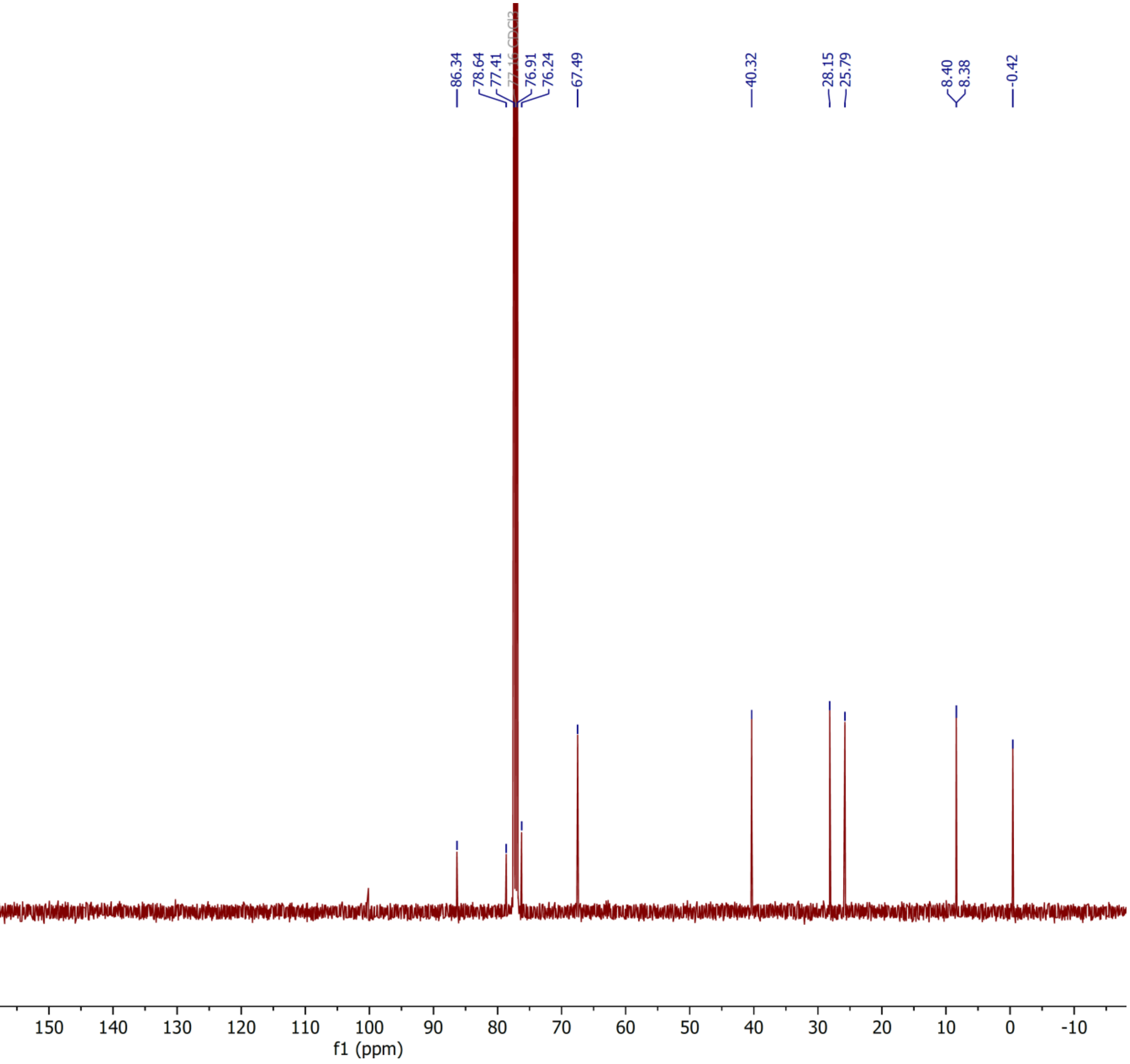

DP-4-185-H

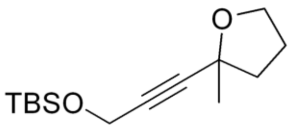

4d

^{1}H NMR (400 MHz, $CDCl_3$)

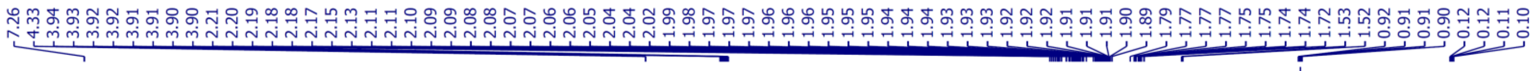

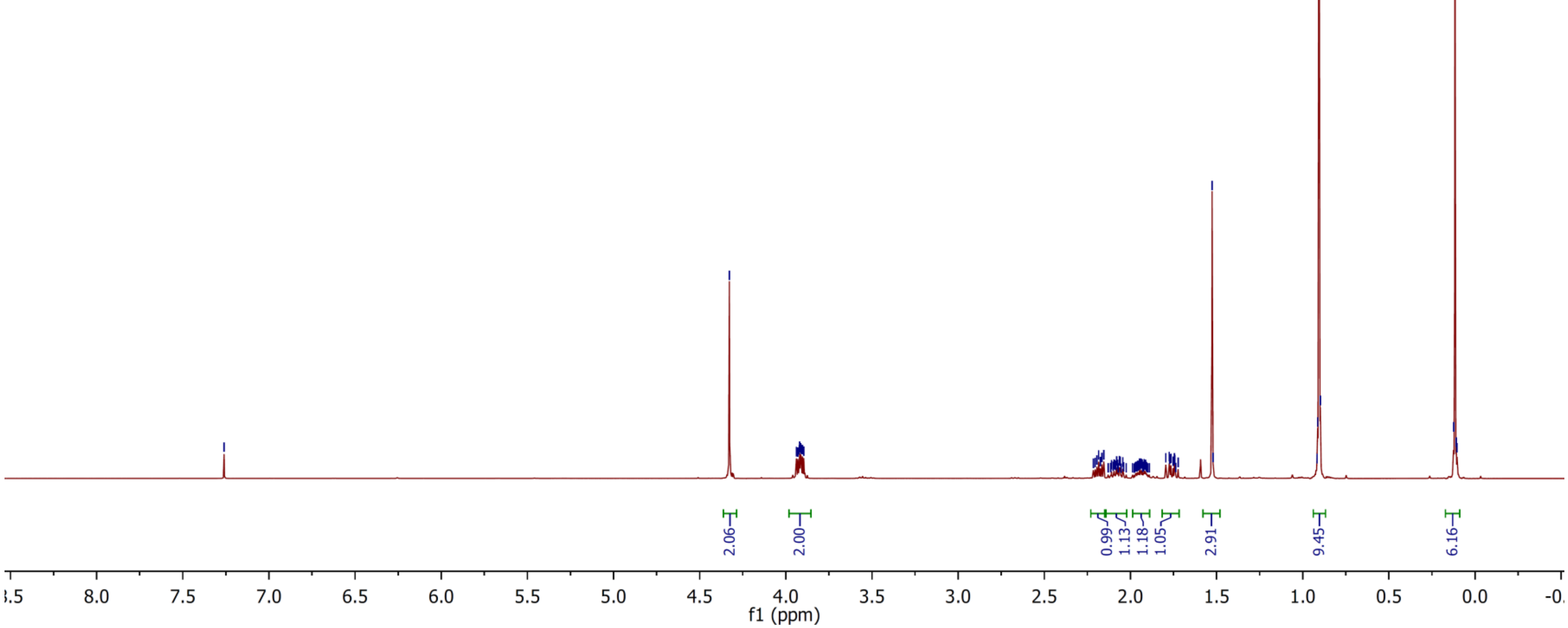

DP-4-185-C

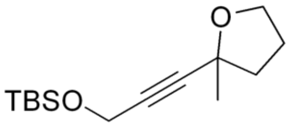

4d

^{13}C NMR (101 MHz, $CDCl_3$)

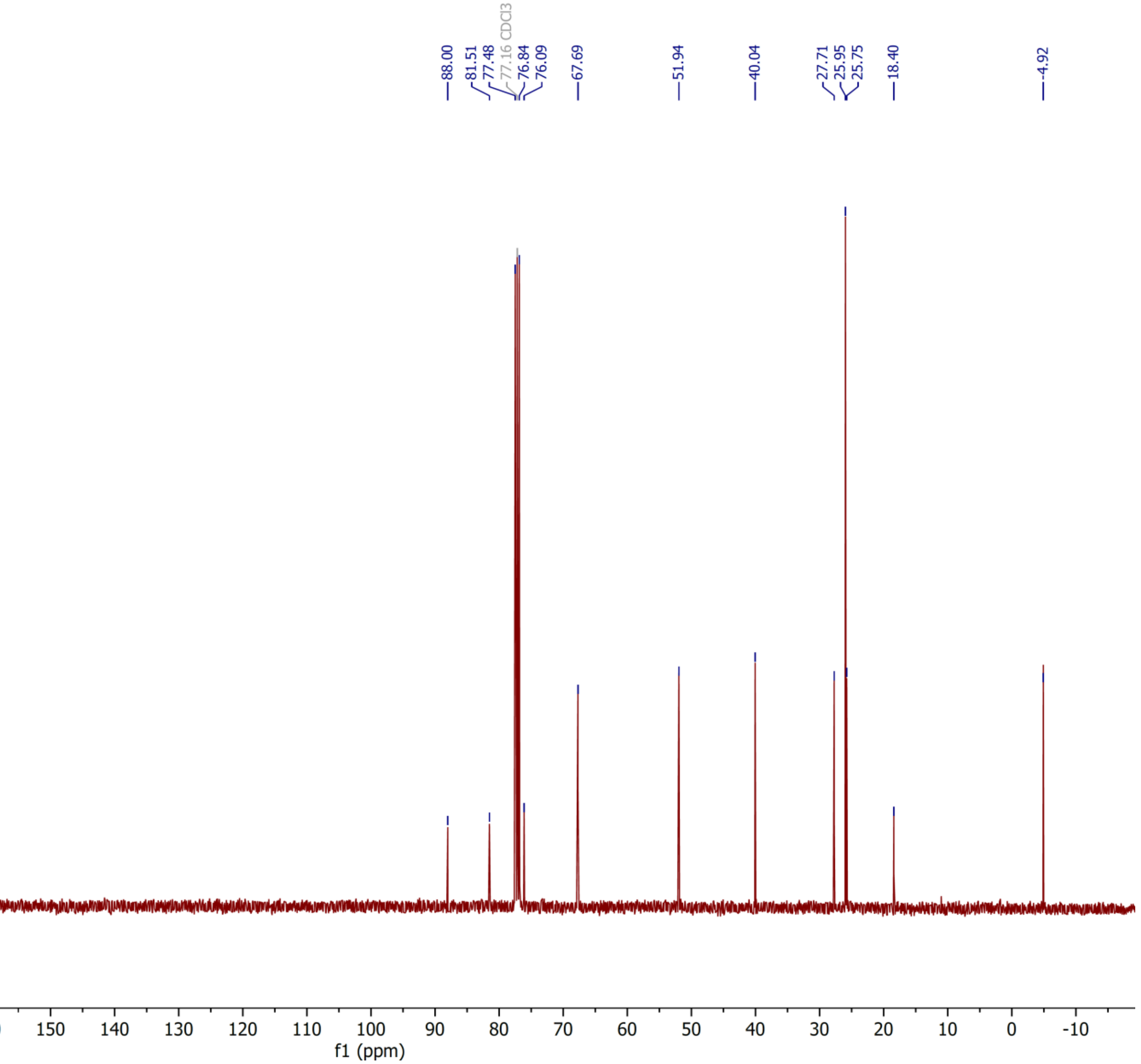

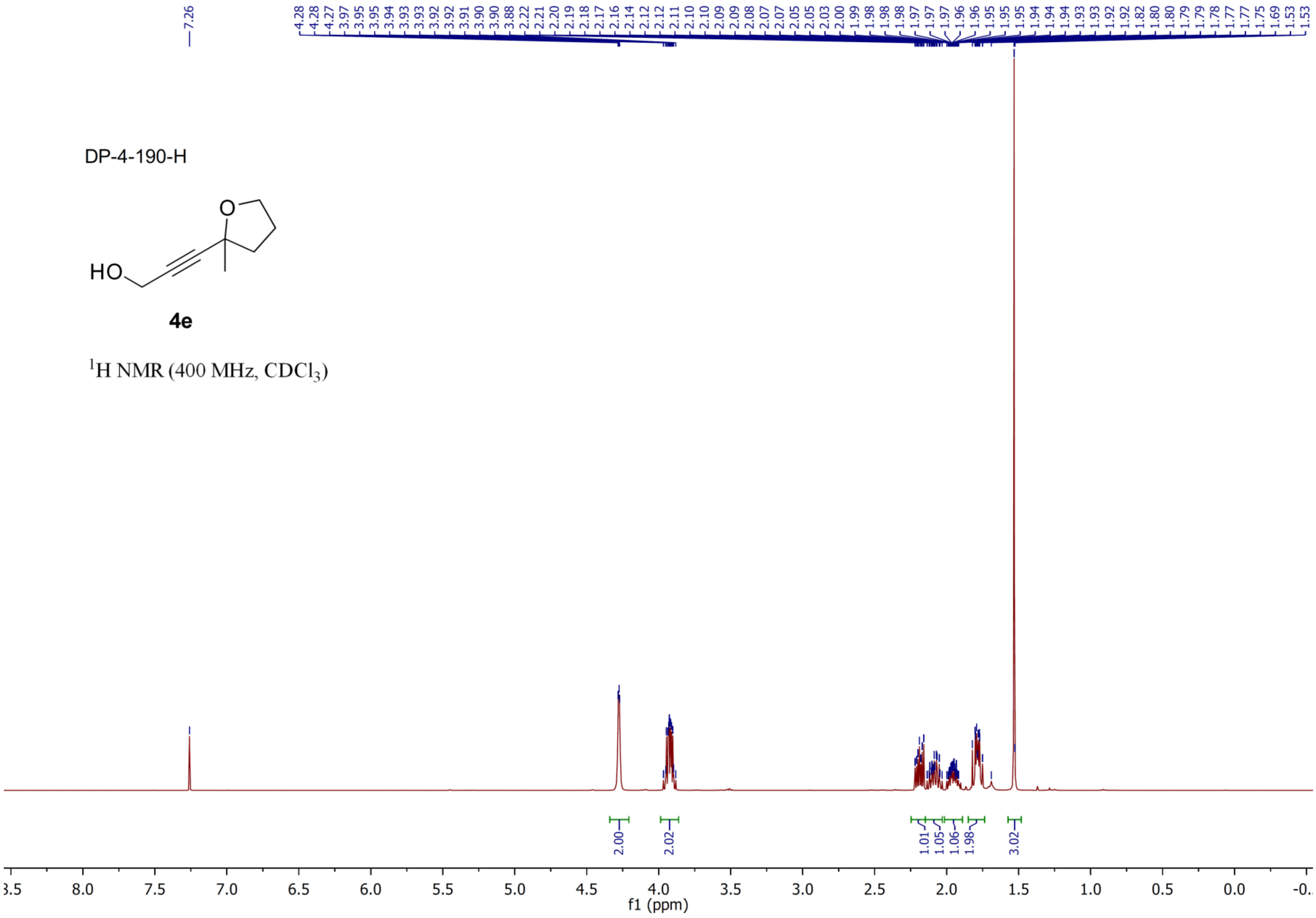
DP-4-190-H
HO
O
4e
^{1}H NMR (400 MHz, CDCl$_3$)
f1 (ppm)

DP-4-190-C

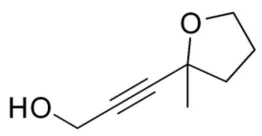

4e

^{13}C NMR (101 MHz, $CDCl_3$)

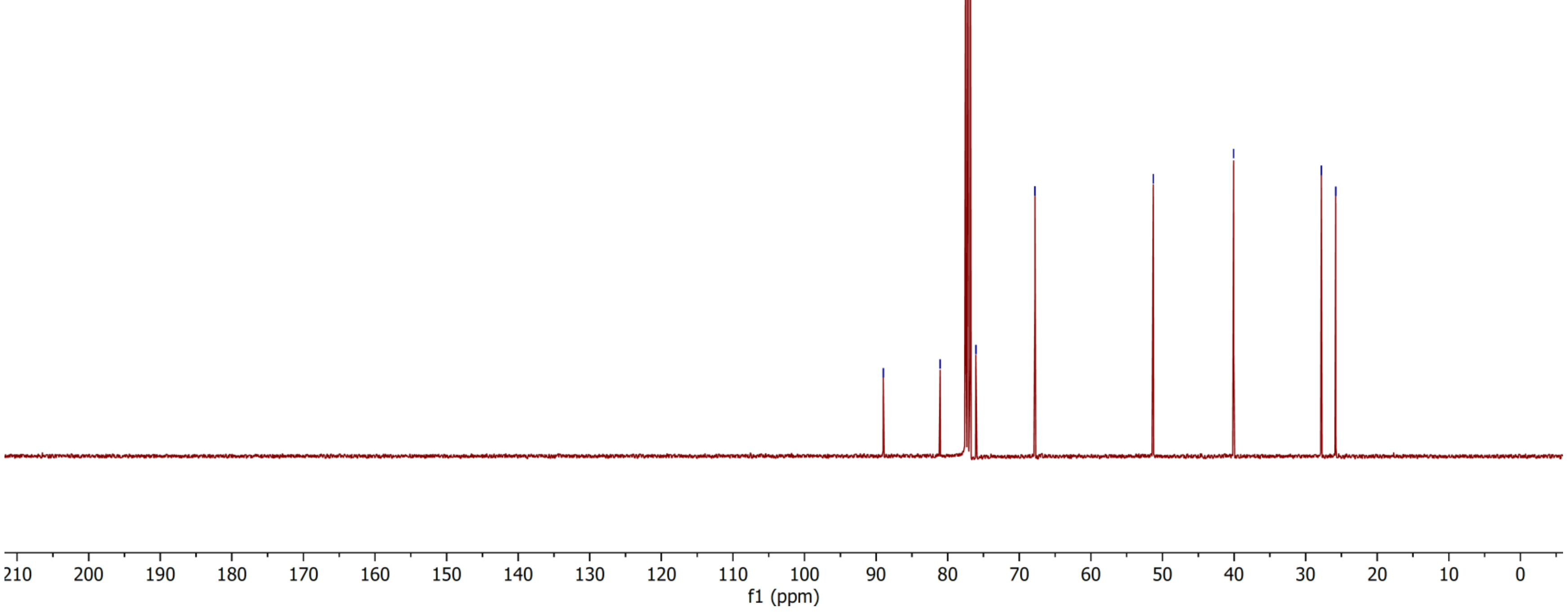

DP-2-198-H

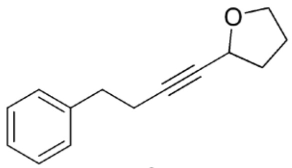

4f

^{1}H NMR (400 MHz, $CDCl_3$)

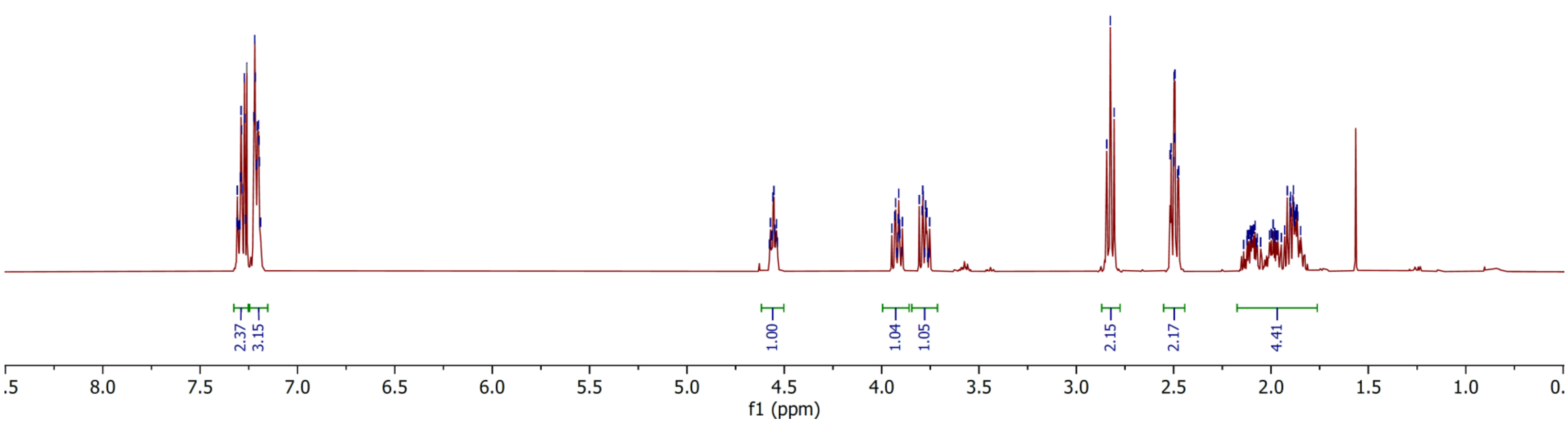

DP-2-198-C

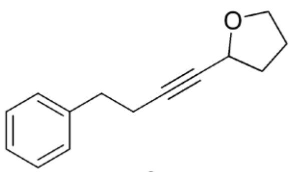

4f

^{13}C NMR (101 MHz, $CDCl_3$)

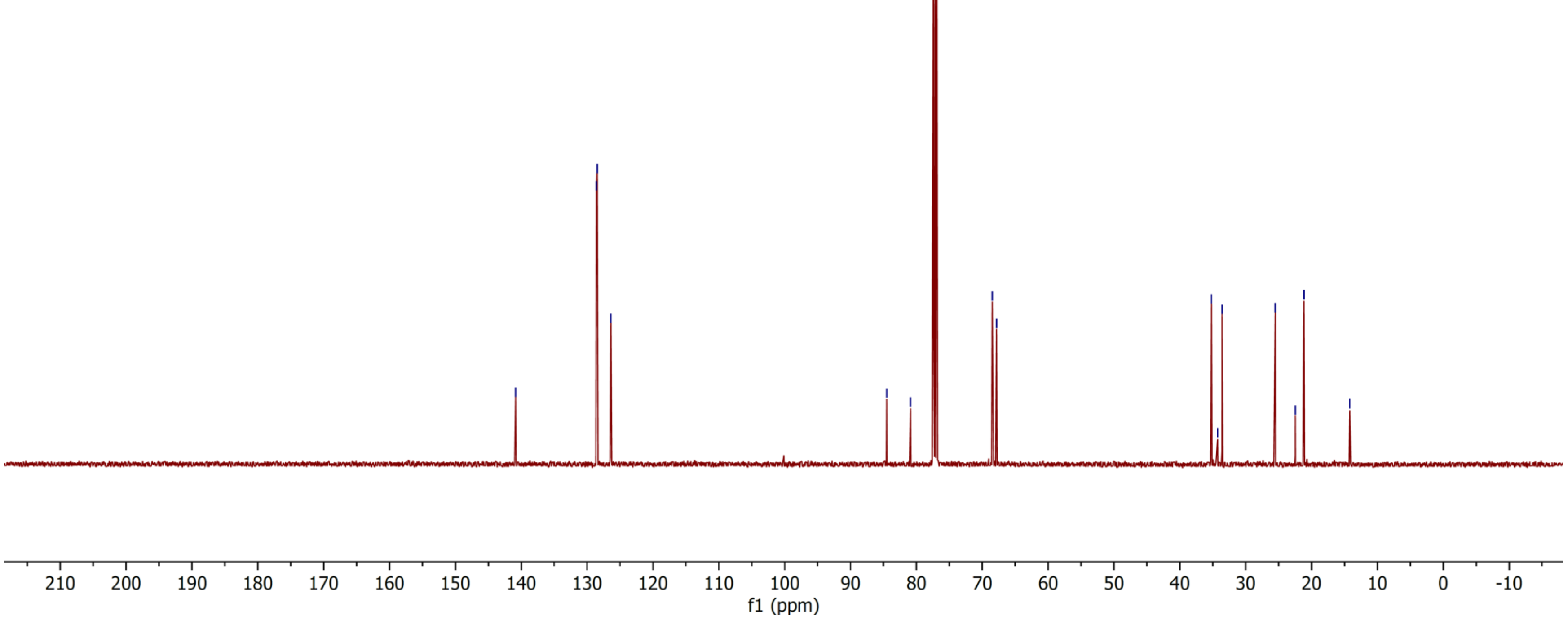

DP-4-195-A-H

4g

^{1}H NMR (400 MHz, $CDCl_3$)

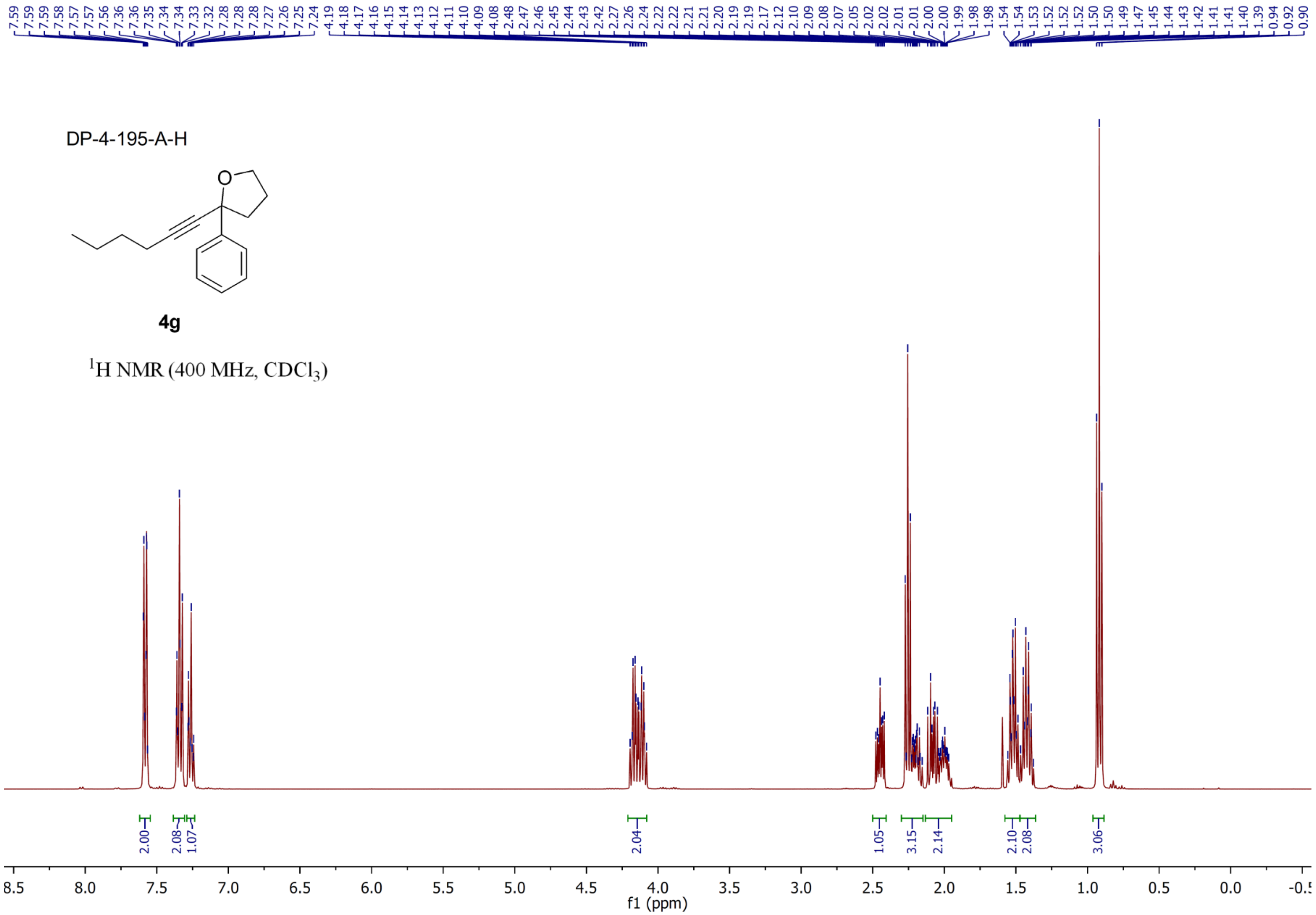

DP-4-195-A-C

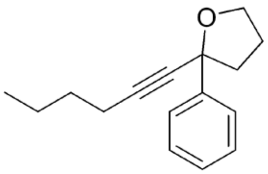

4g

^{13}C NMR (101 MHz, $CDCl_3$)

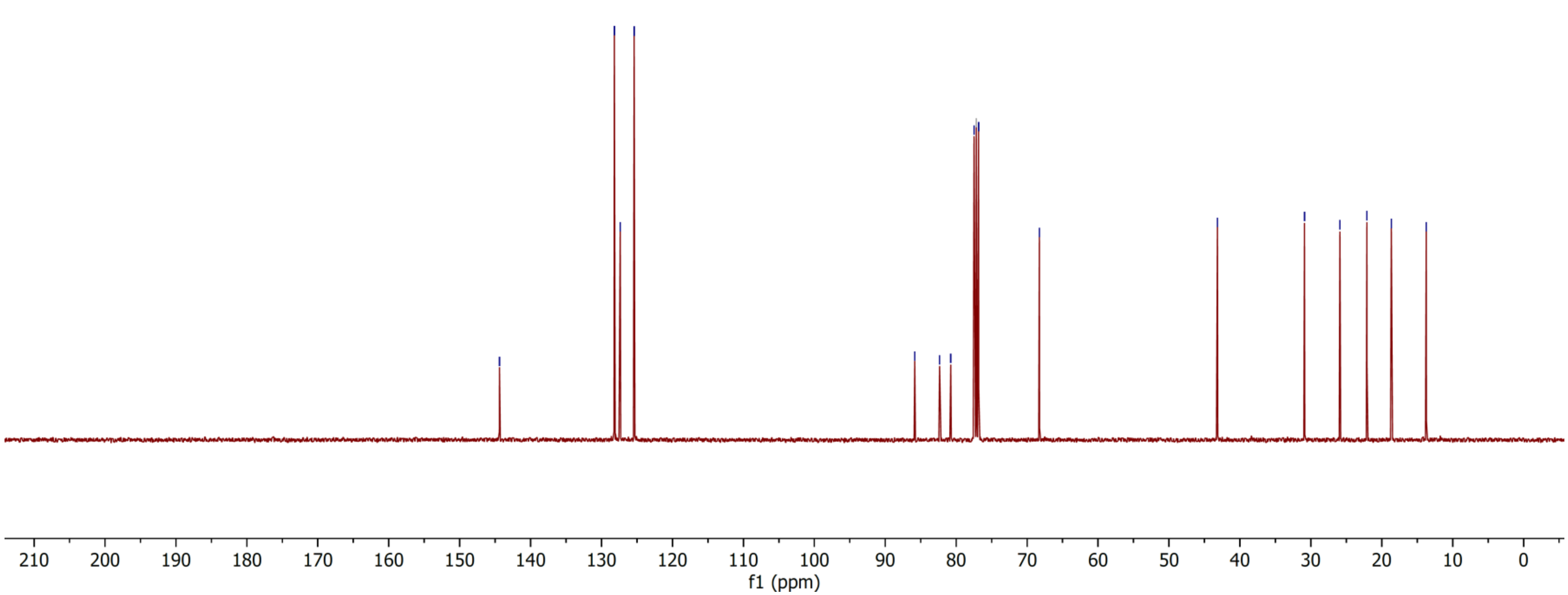

DP-4-195-C-H

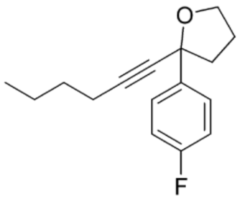

4h

^{1}H NMR (400 MHz, $CDCl_3$)

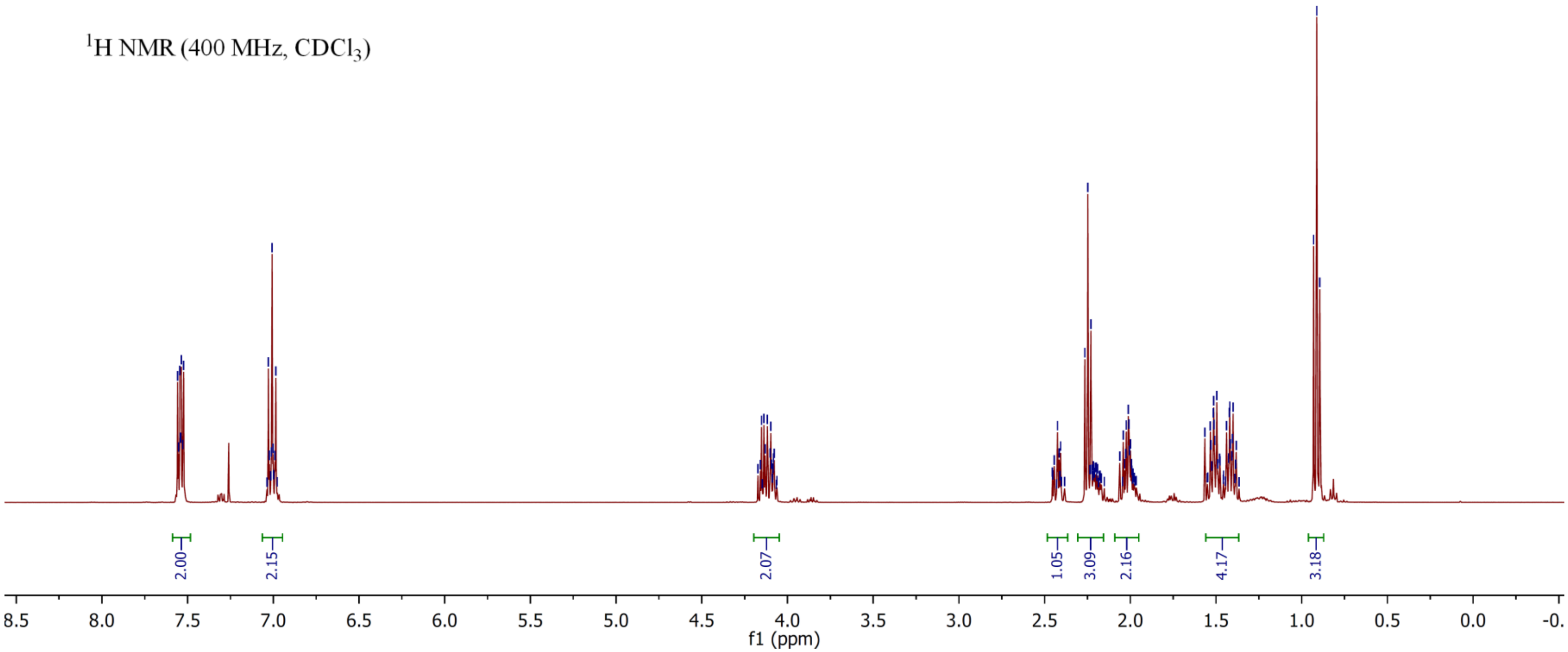

DP-4-195-C-C

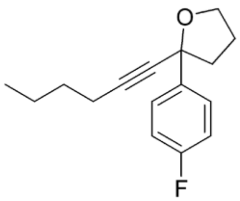

4h

^{13}C NMR (101 MHz, $CDCl_3$)

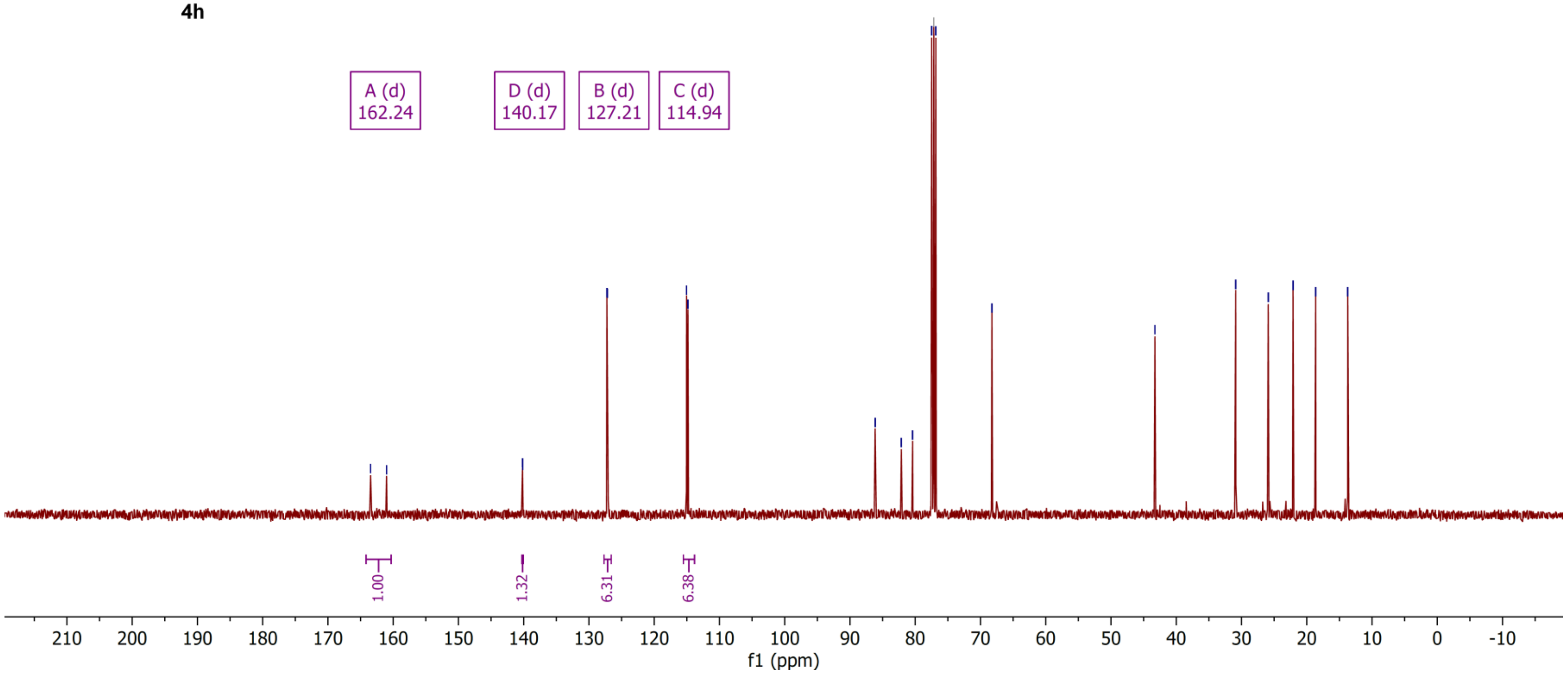

DP-4-195-C-F

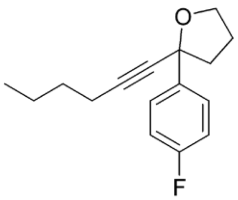

4h

^{19}F NMR (377 MHz, $CDCl_3$)

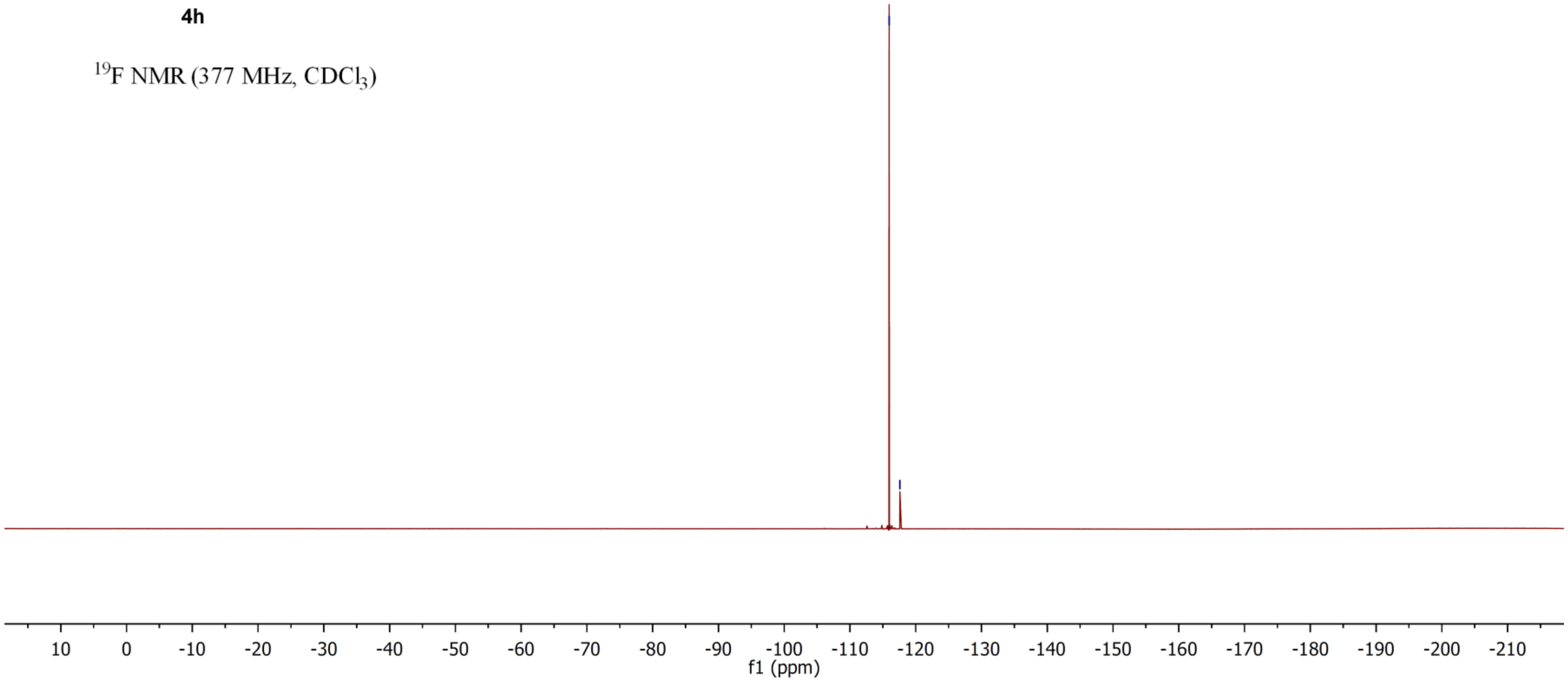

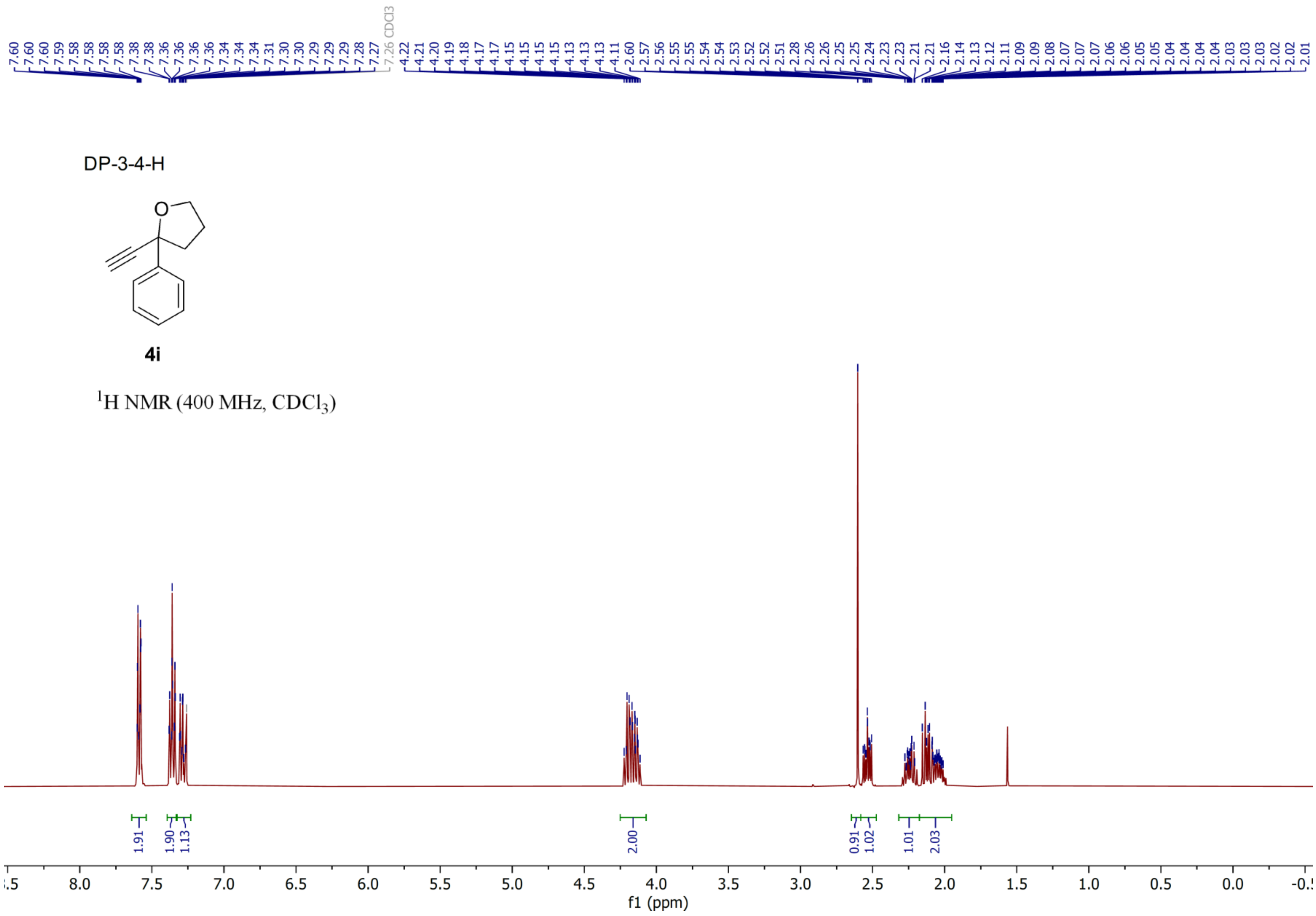
DP-3-4-H
4i
1H NMR (400 MHz, CDCl3)
f1 (ppm)

DP-3-4-C

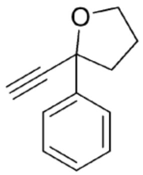

4i

^{13}C NMR (101 MHz, $CDCl_3$)

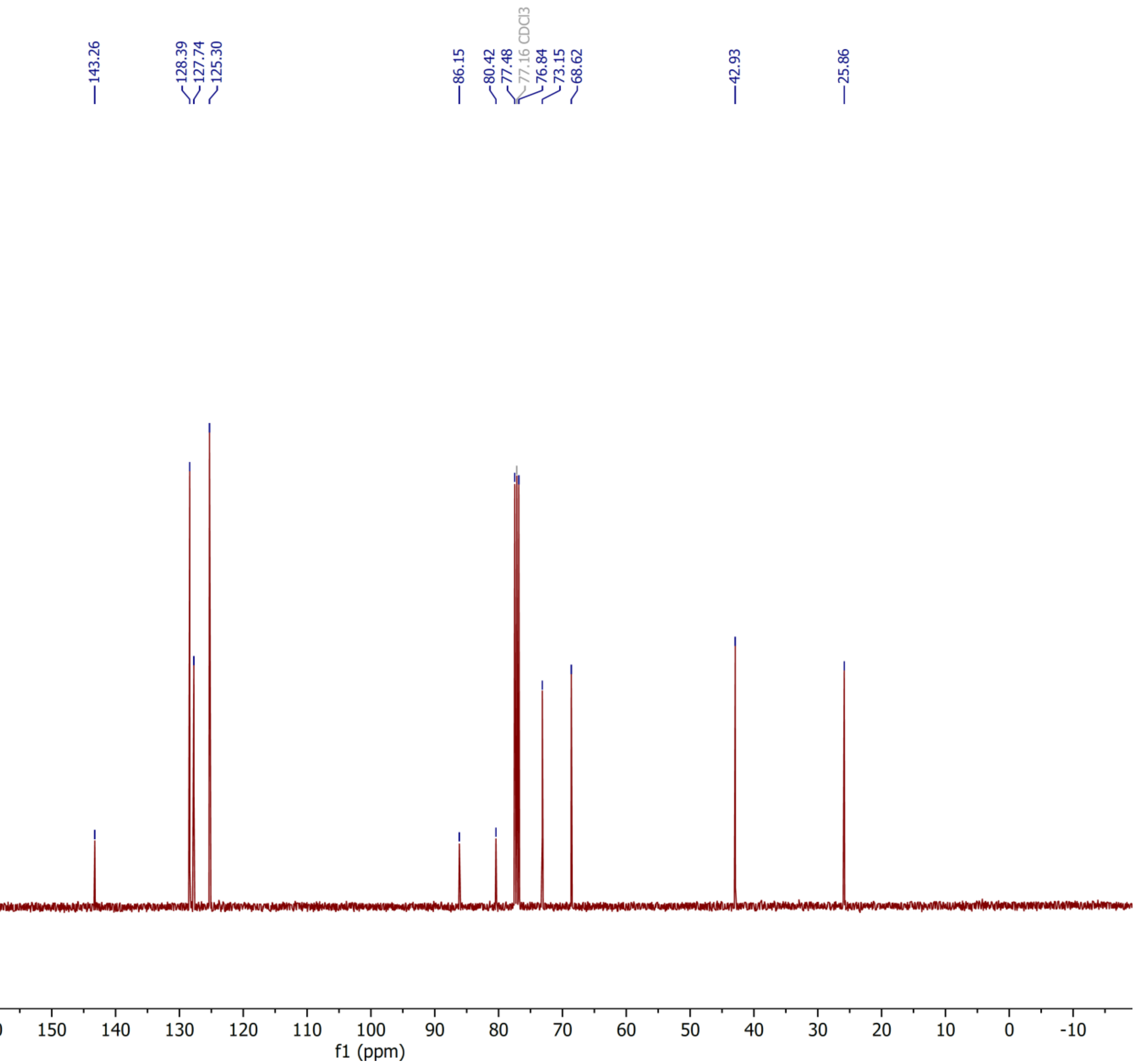

^{1}H and ^{13}C NMR spectra of compounds 6a – 6e:

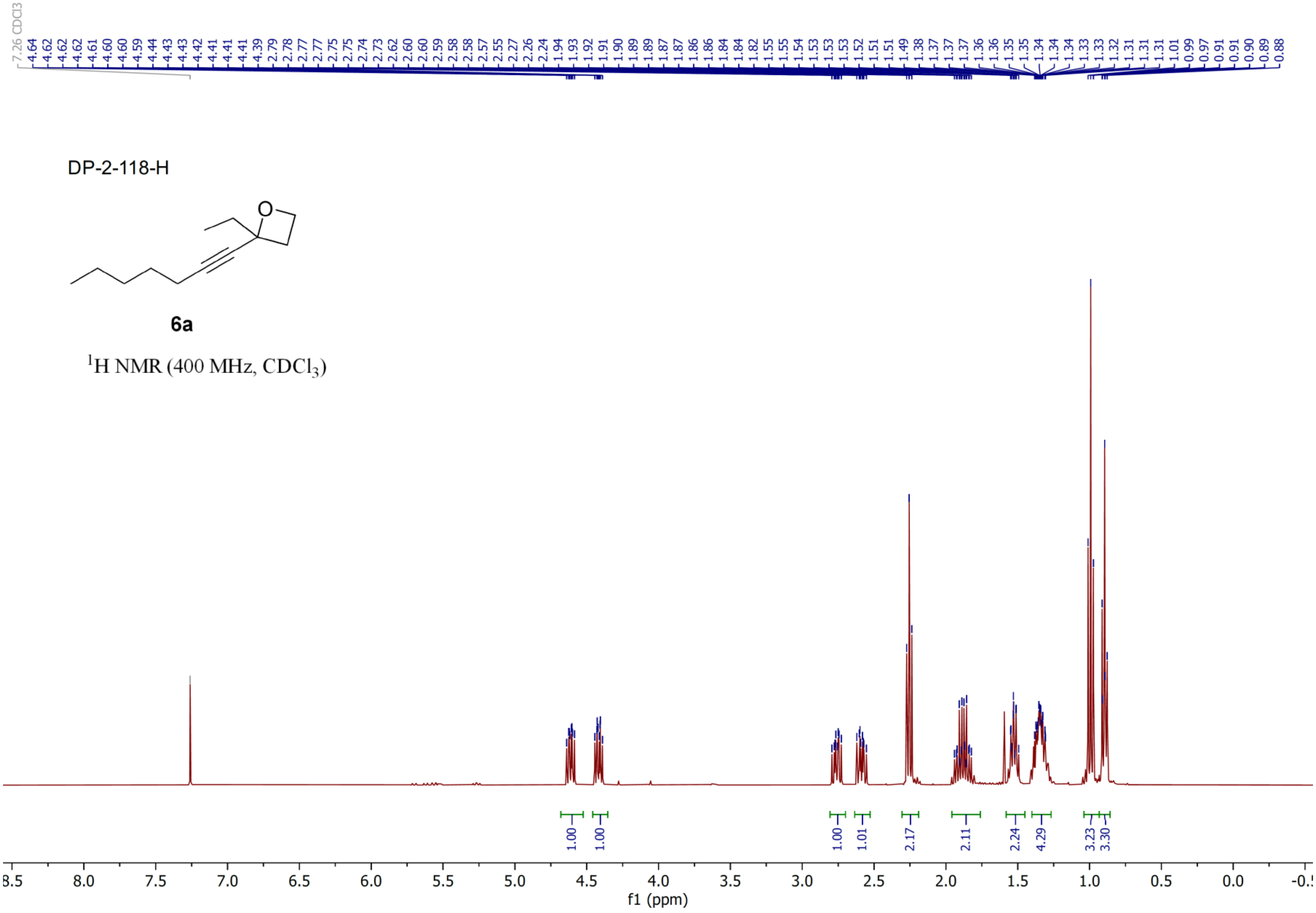

DP-2-118-C

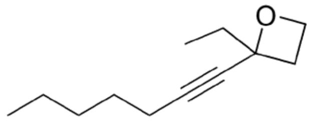

6a

^{13}C NMR (101 MHz, $CDCl_3$)

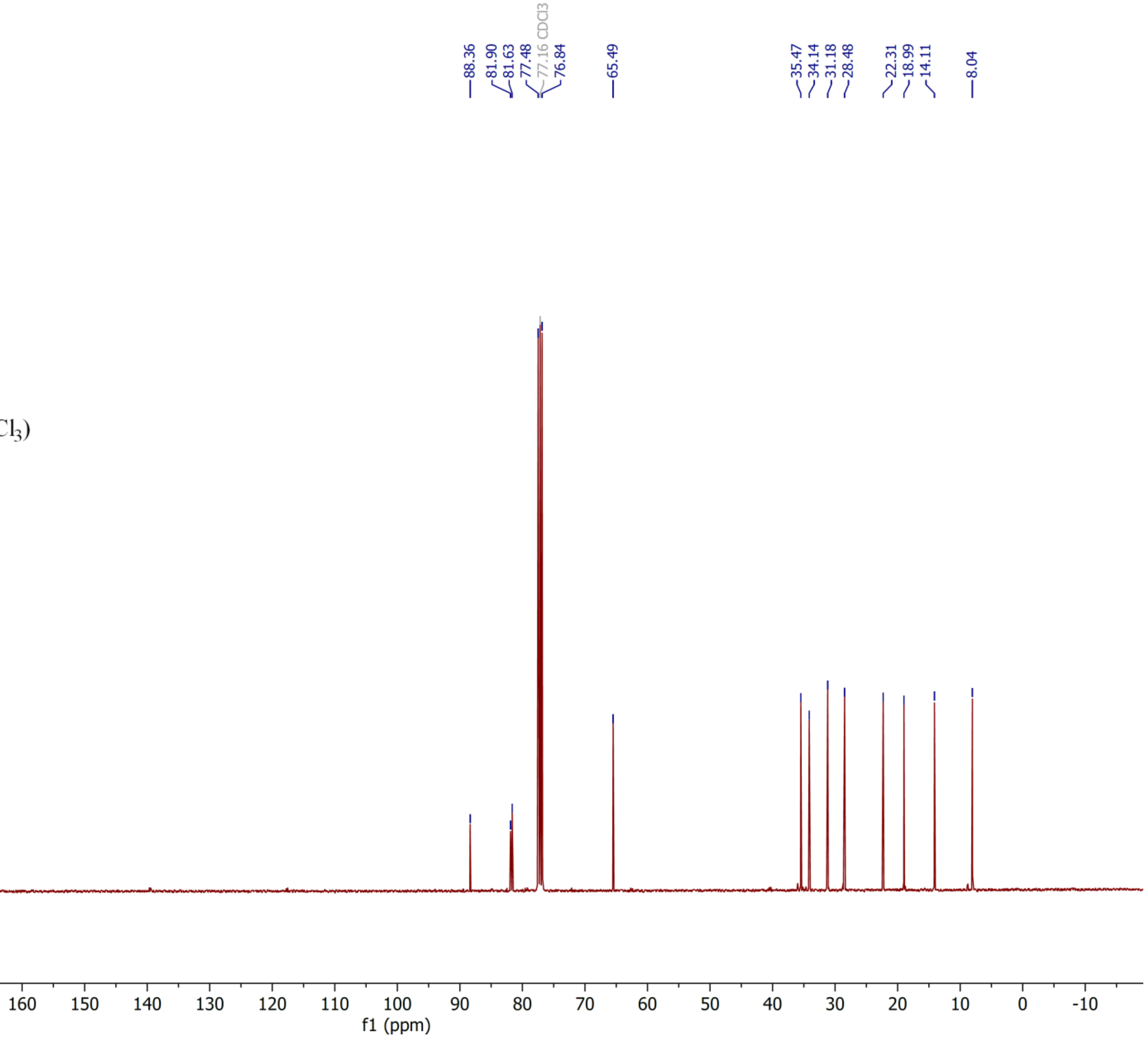

DP-3-30-H-500mhz

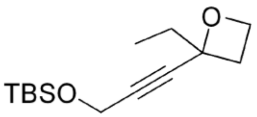

6b

^{1}H NMR (500 MHz, $CDCl_3$)

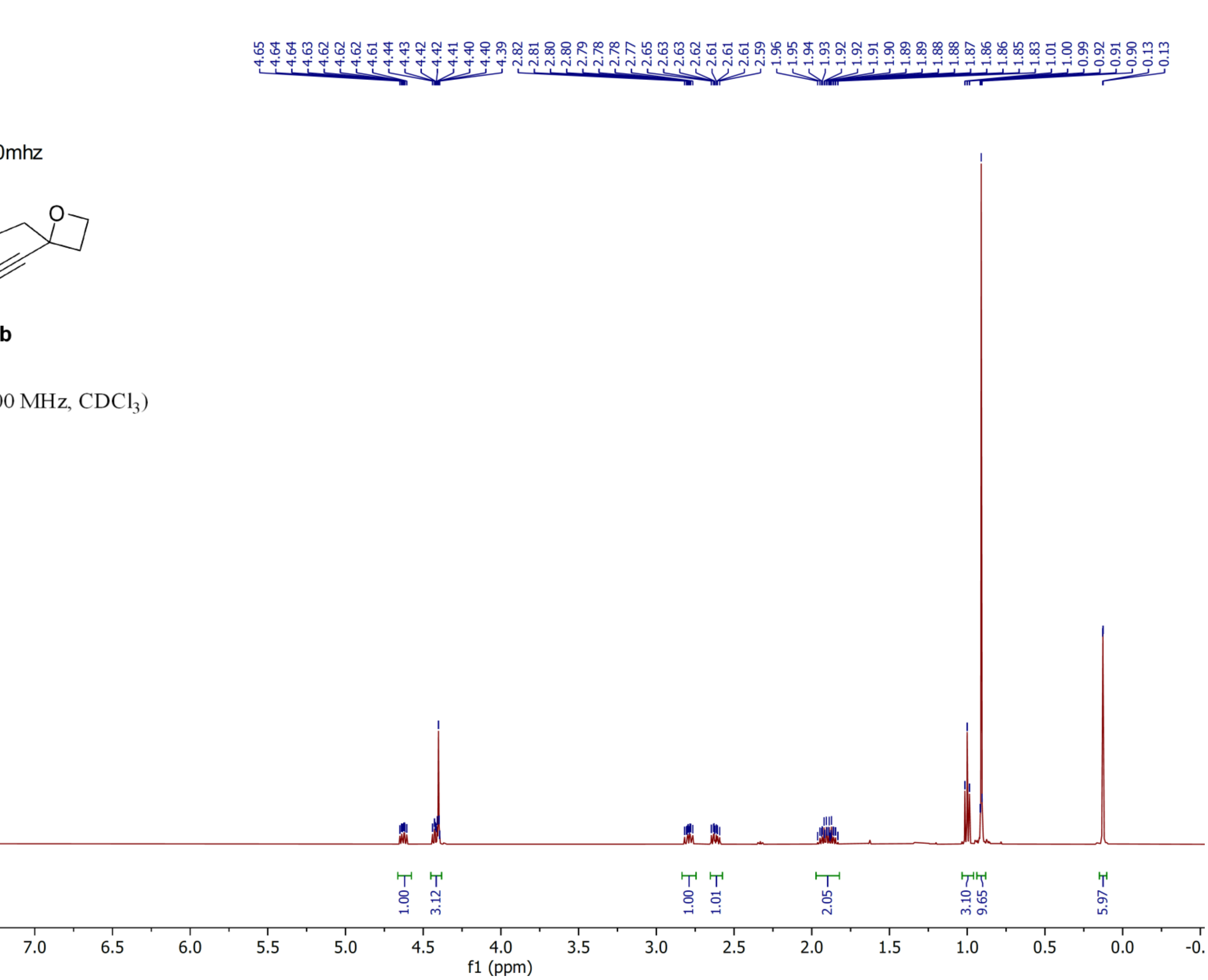

DP-3-30-C-500mhz

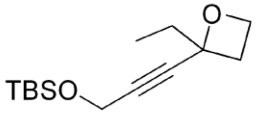

6b

^{13}C NMR (126 MHz, $CDCl_3$)

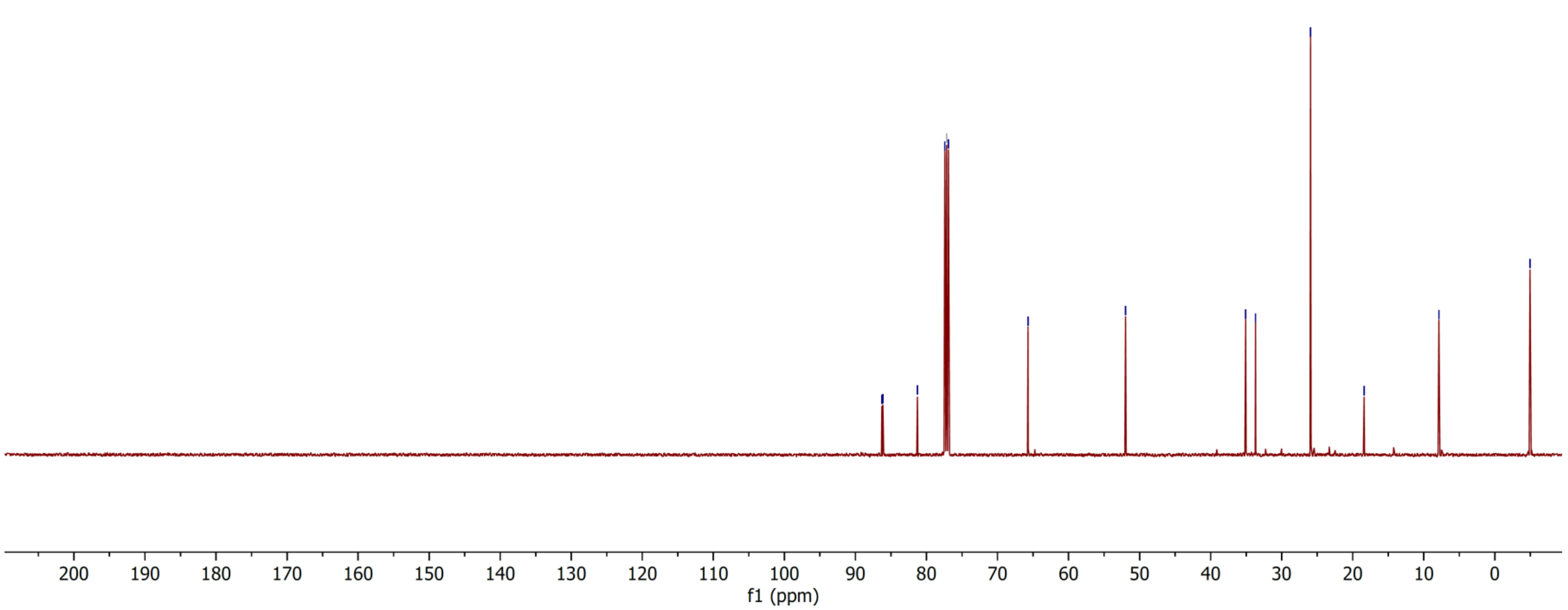

DP-3-31-H

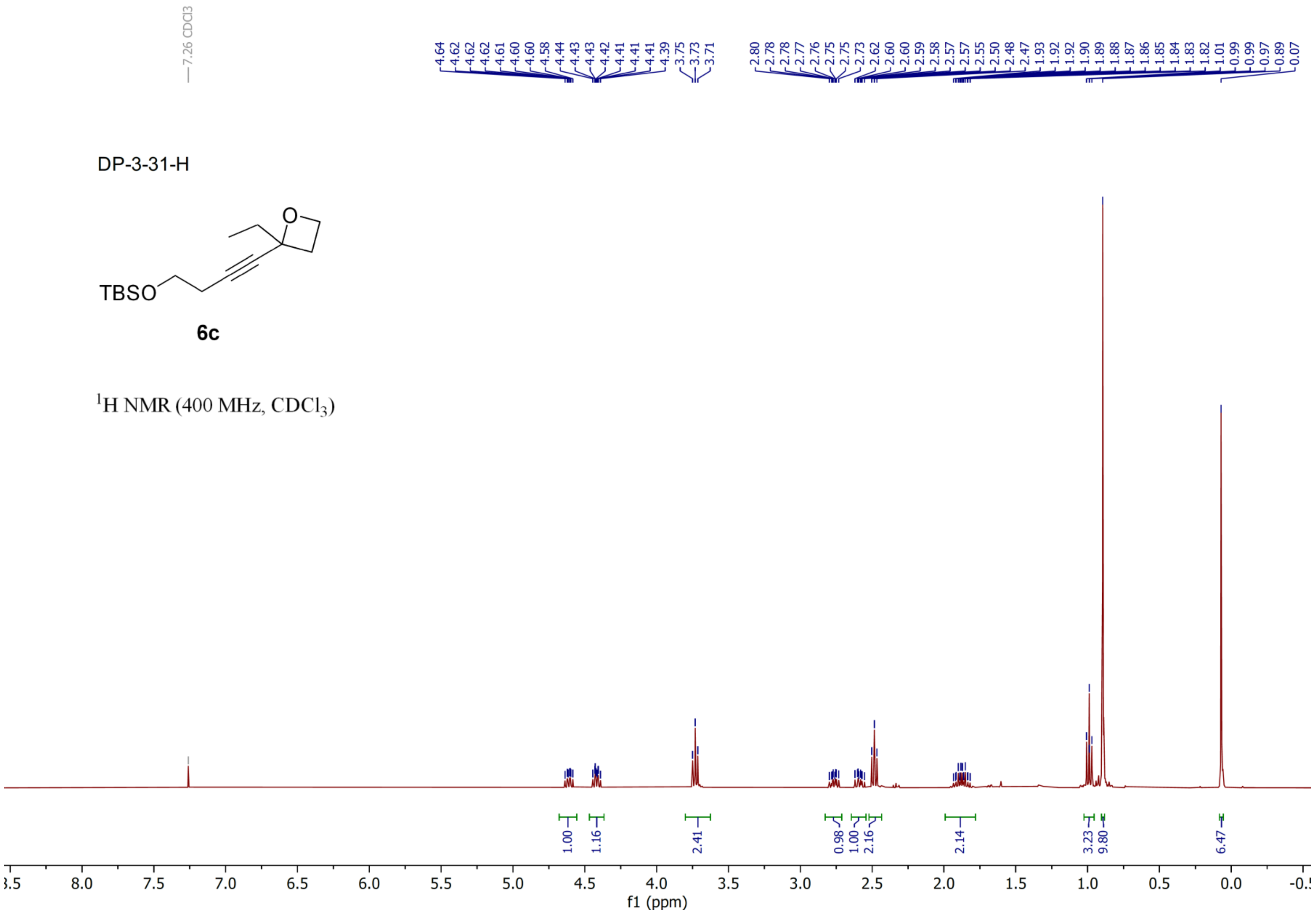

6c

^{1}H NMR (400 MHz, $CDCl_3$)

DP-3-31-C

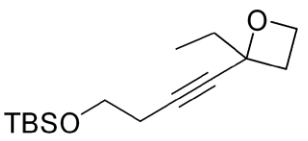

6c

^{13}C NMR (101 MHz, $CDCl_3$)

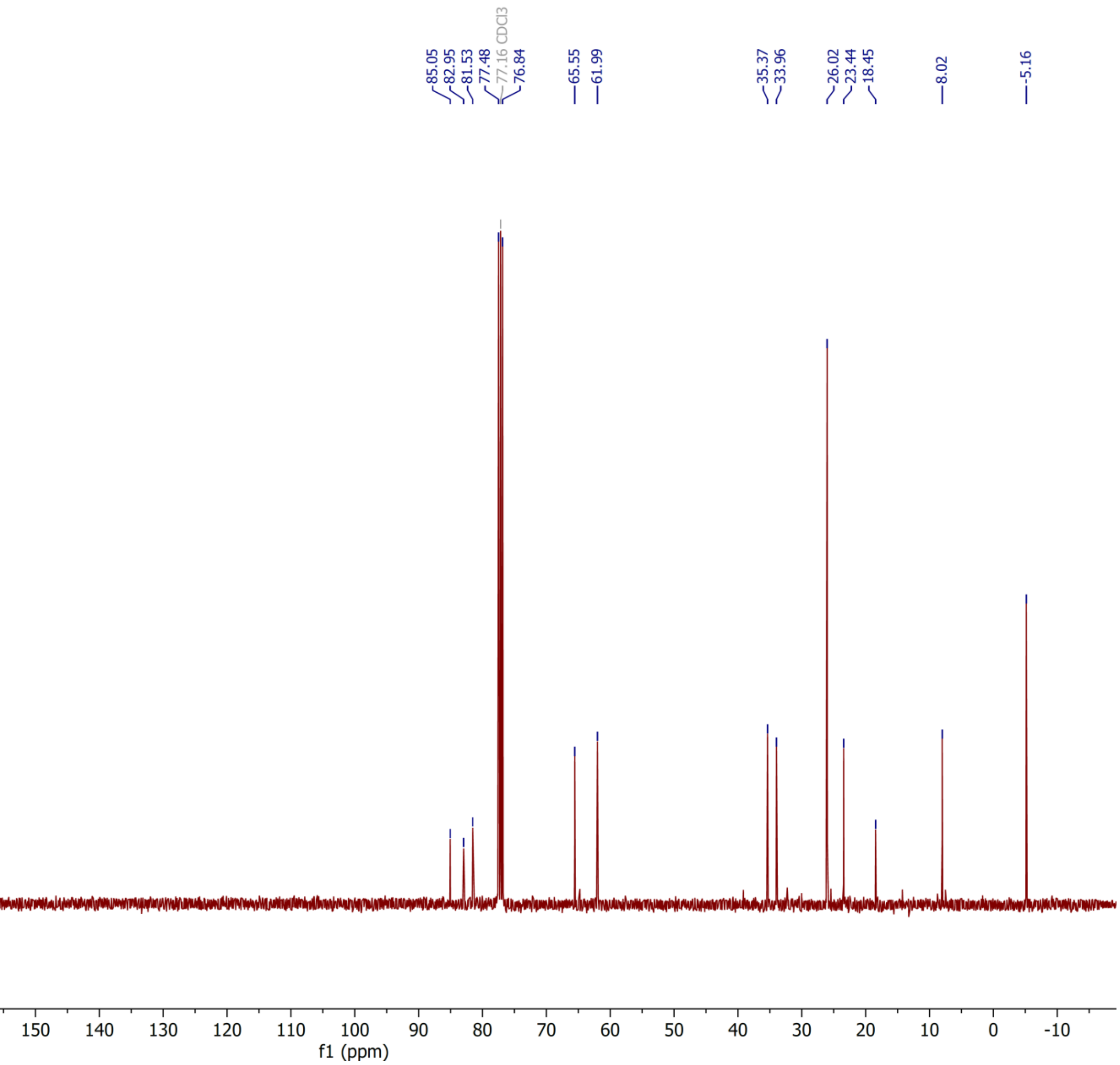

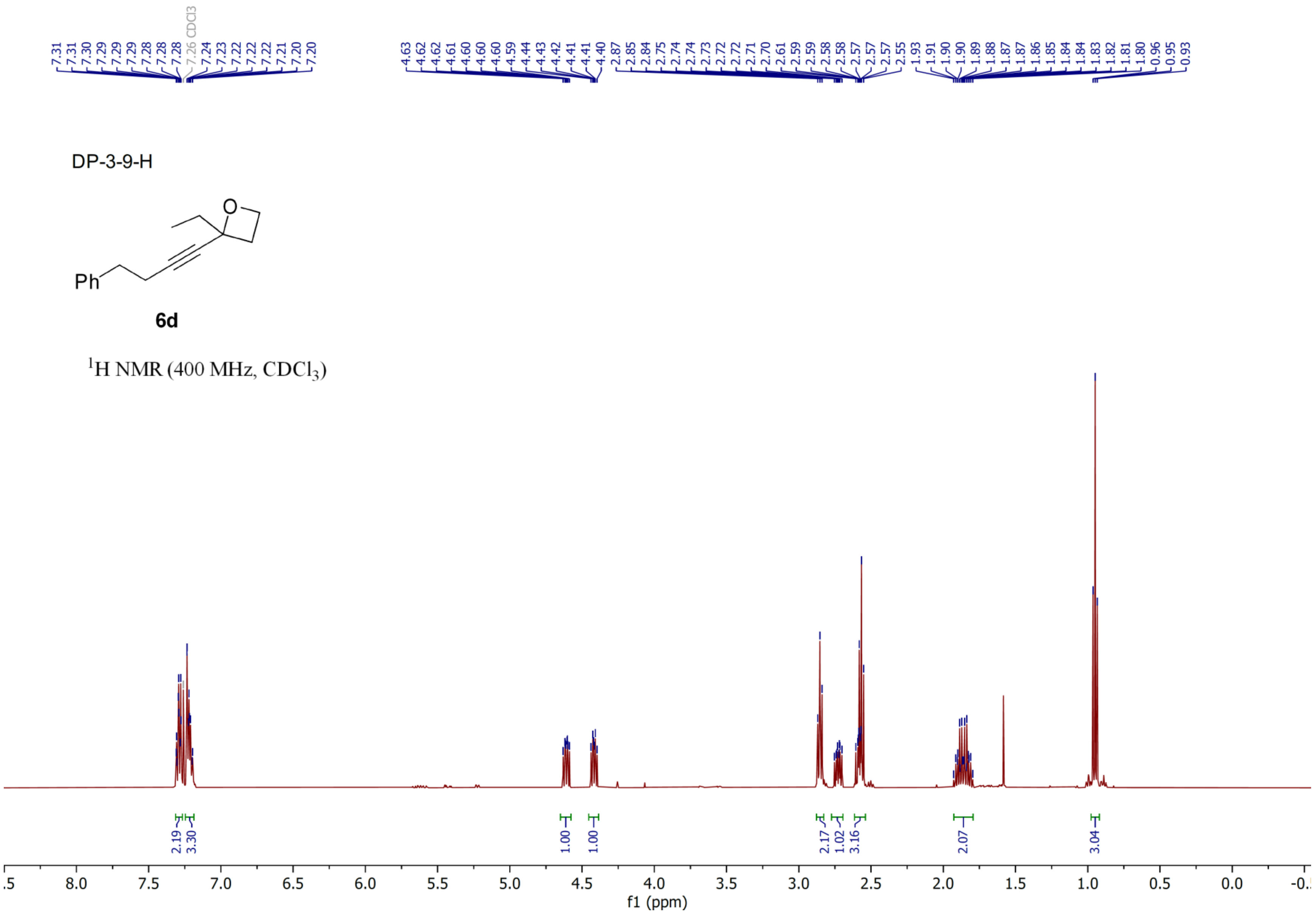

DP-3-9-H
Ph
O
6d
^{1}H NMR (400 MHz, $CDCl_3$)
7.31 7.31 7.30 7.29 7.29 7.29 7.28 7.28 7.28 7.26 CDCl3 7.24 7.23 7.22 7.22 7.22 7.21 7.20 7.20
4.63 4.62 4.62 4.61 4.60 4.60 4.60 4.59 4.44 4.43 4.42 4.41 4.41 4.40 2.87 2.85 2.84 2.75 2.74 2.74 2.73 2.72 2.72 2.71 2.70 2.61 2.59 2.59 2.58 2.58 2.57 2.57 2.57 2.55 1.93 1.91 1.90 1.90 1.89 1.88 1.87 1.87 1.86 1.85 1.84 1.84 1.83 1.83 1.82 1.81 1.80 0.96 0.95 0.93
2.19 3.30 1.00 1.00 2.17 1.02 3.16 2.07 3.04
f1 (ppm)

DP-3-9-C

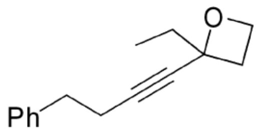

6d

^{13}C NMR (101 MHz, $CDCl_3$)

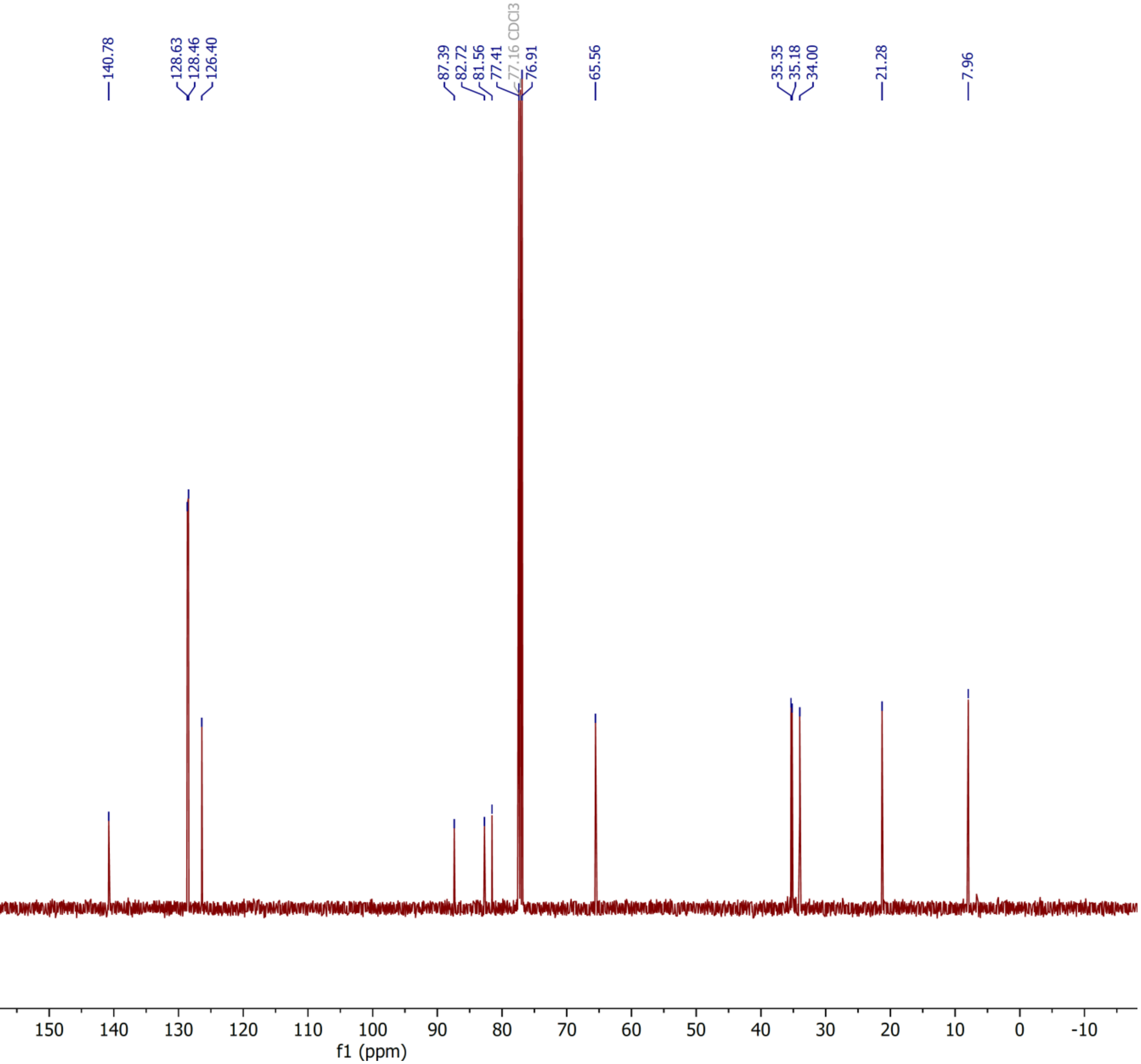

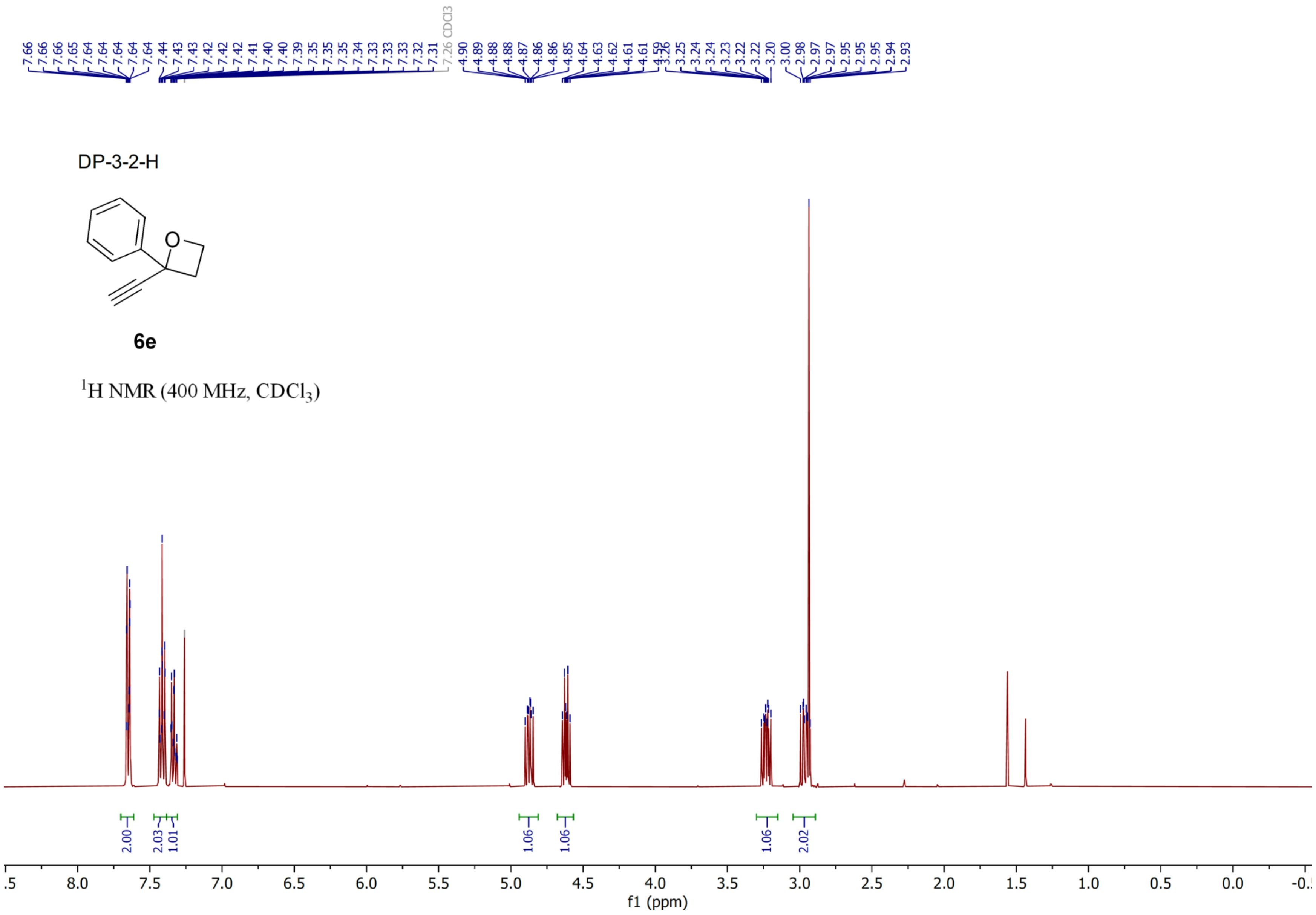
DP-3-2-H
6e
1H NMR (400 MHz, CDCl3)
CDCl3
f1 (ppm)
2.00
2.03
1.01
1.06
1.06
1.06
2.02

DP-3-2-C

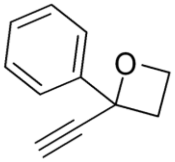

6e

^{13}C NMR (101 MHz, $CDCl_3$)

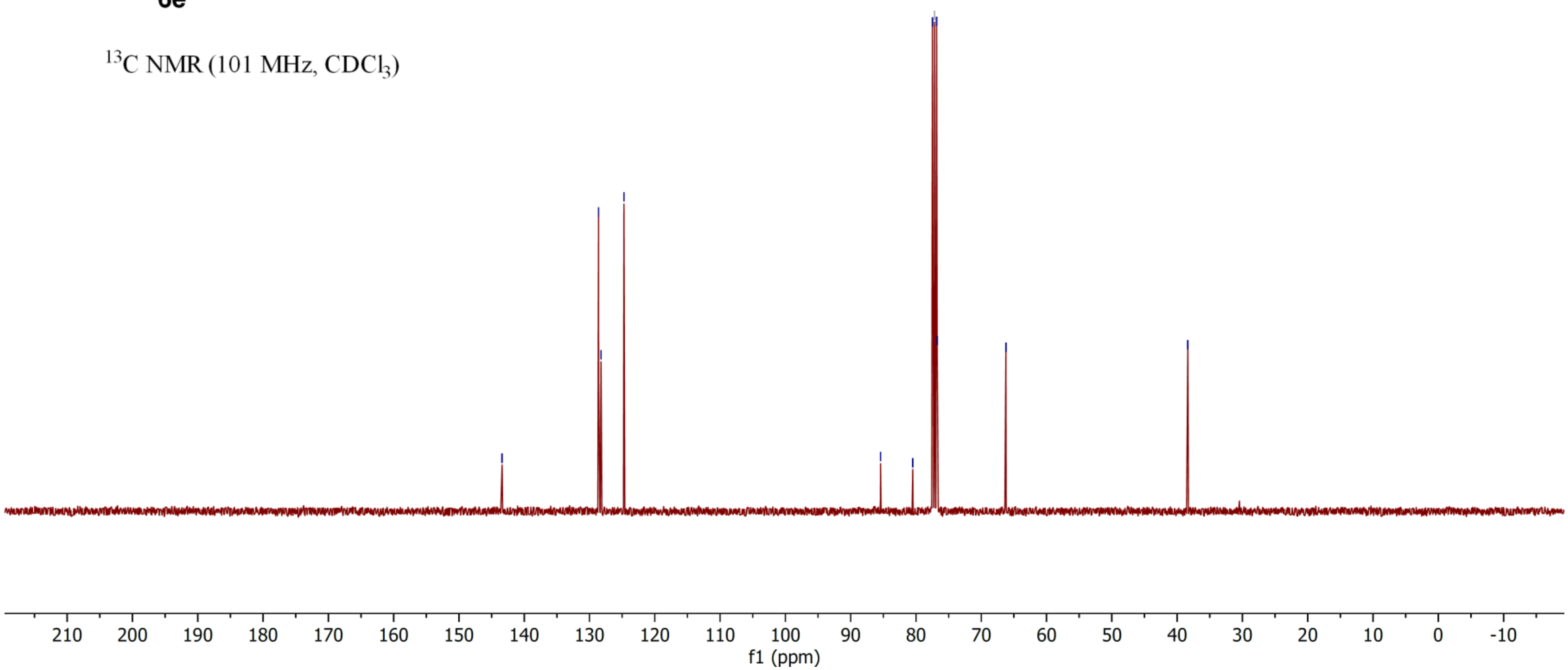

^{1}H and ^{13}C NMR spectra of compounds 2aa – 2s and 3:

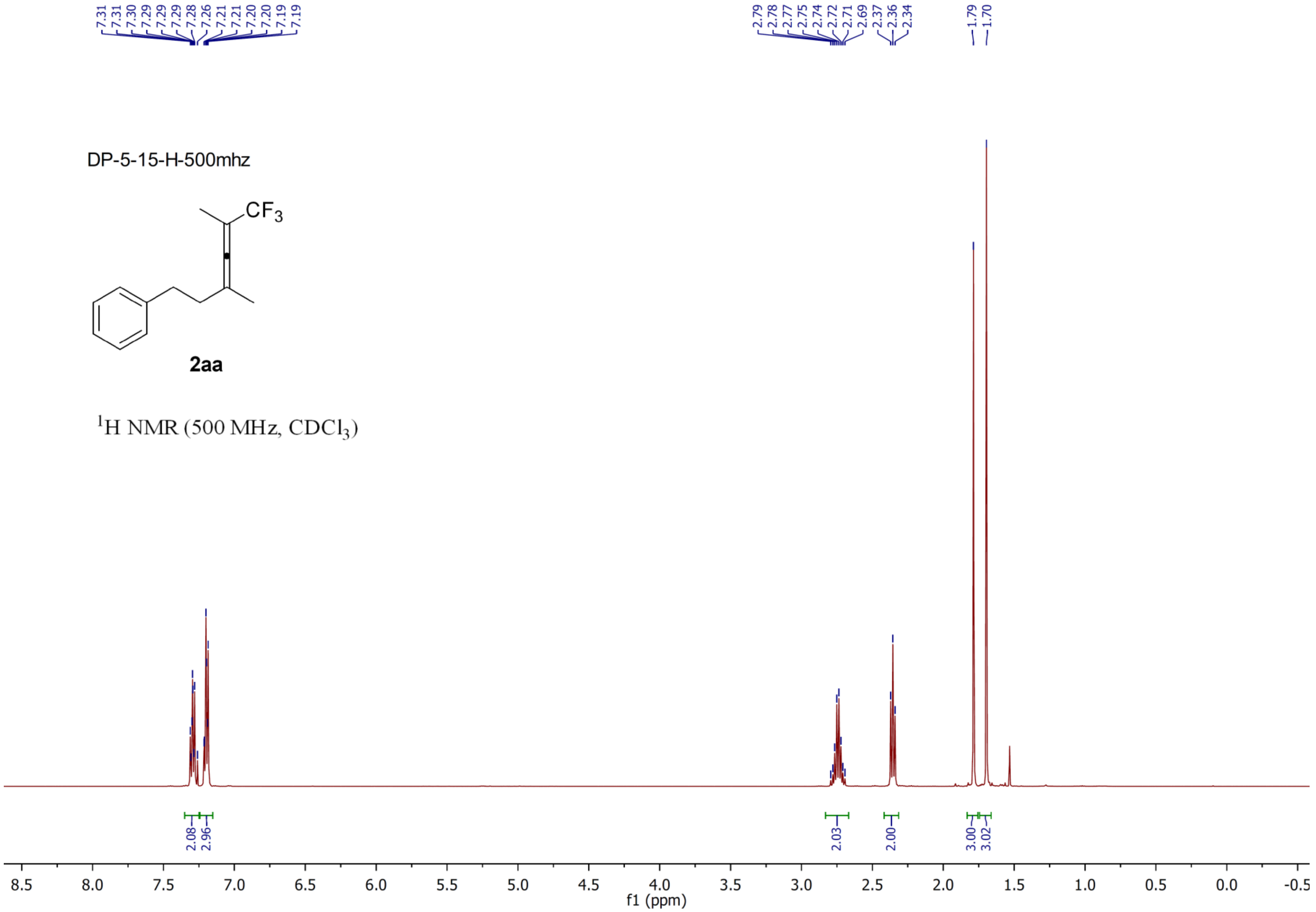

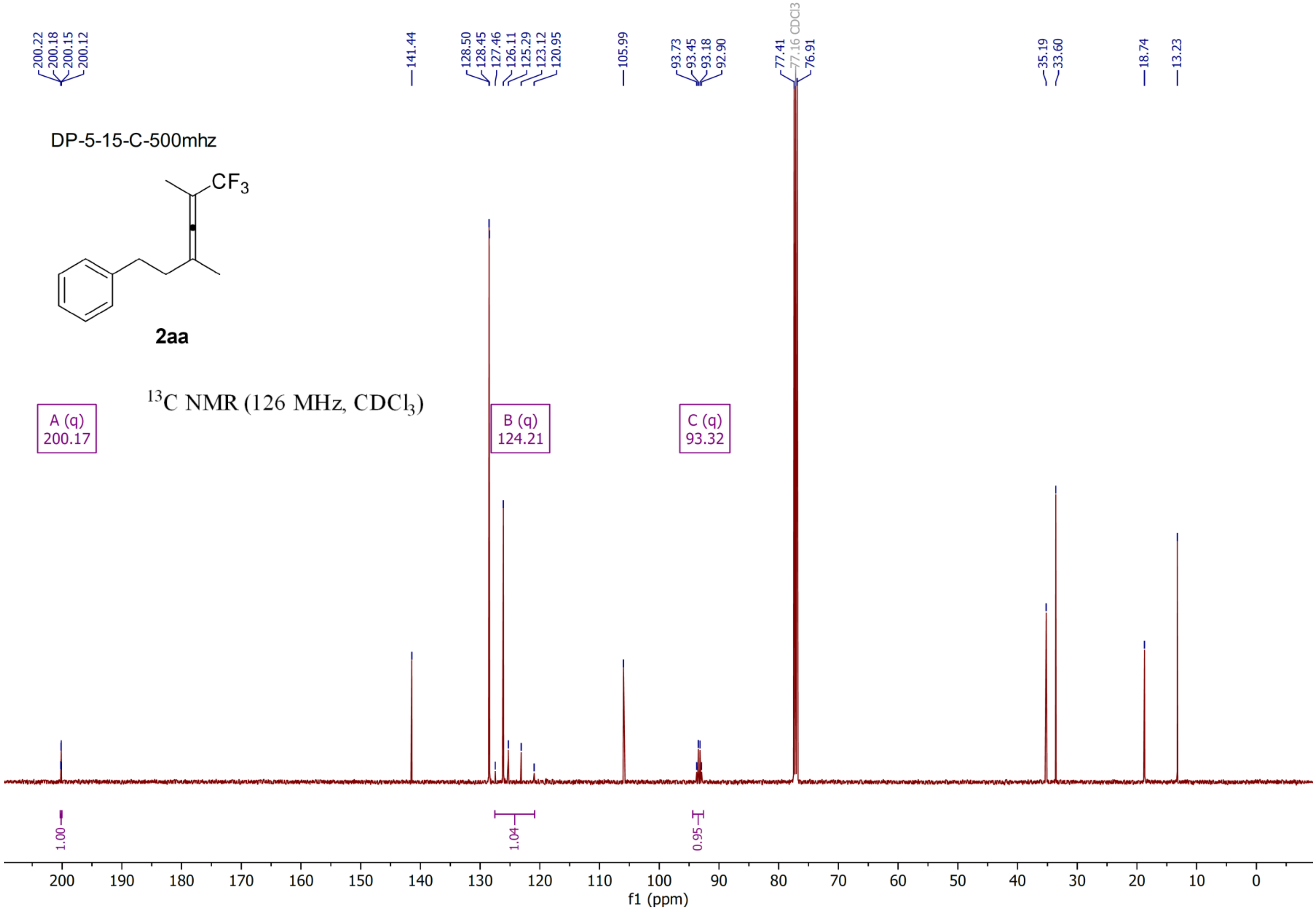
200.22
200.18
200.15
200.12
141.44
128.50
128.45
127.46
126.11
125.29
123.12
120.95
105.99
93.73
93.45
93.18
92.90
77.41
77.16 CDCl3
76.91
35.19
33.60
18.74
13.23
DP-5-15-C-500mhz
CF3
2aa
13C NMR (126 MHz, CDCl3)
A (q)
200.17
B (q)
124.21
C (q)
93.32
1.00
1.04
0.95
f1 (ppm)

DP-5-15-F

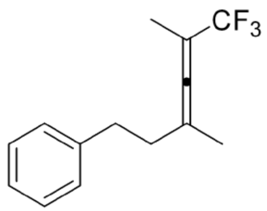

2aa

^{19}F NMR (377 MHz, $CDCl_3$)

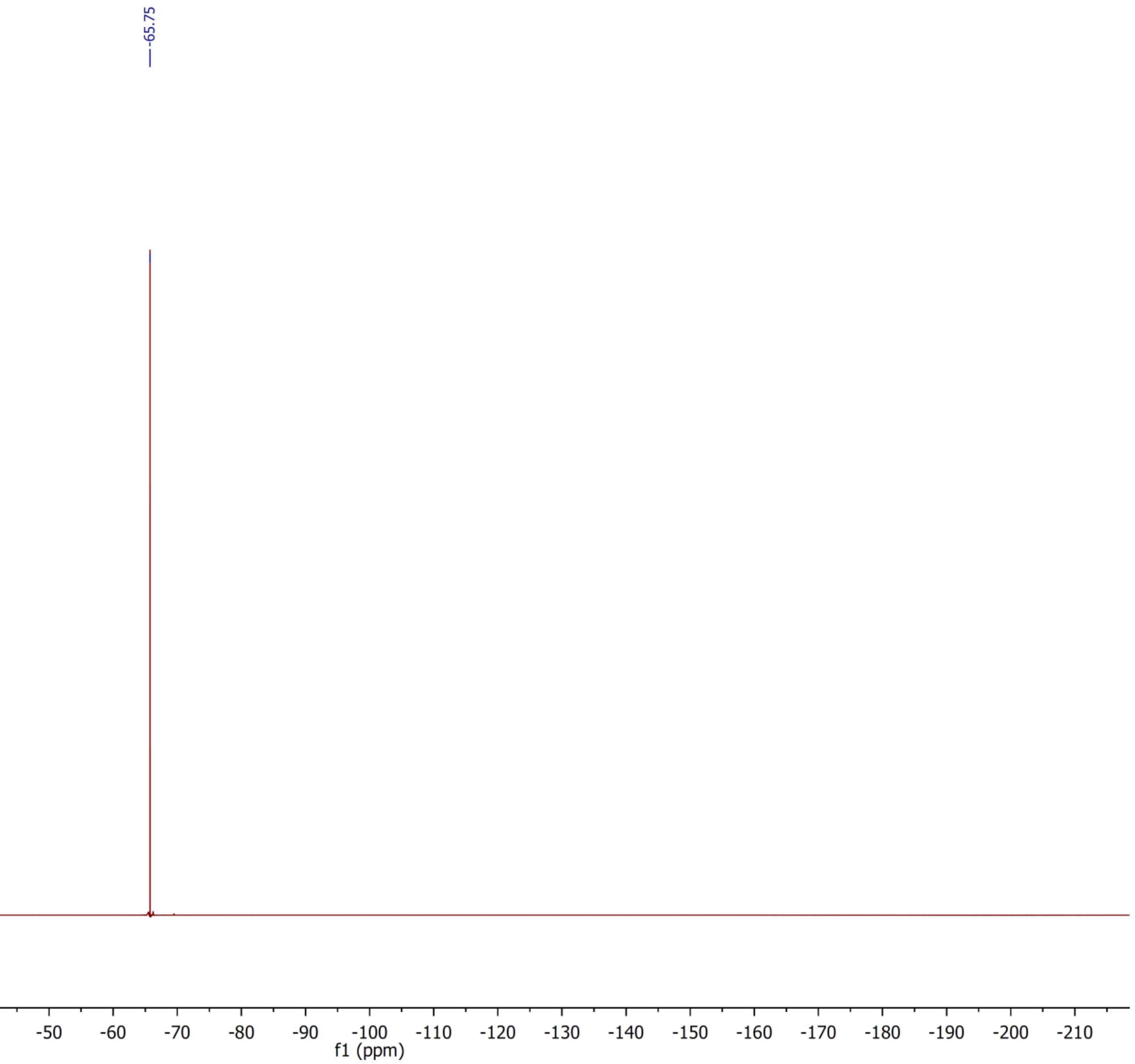

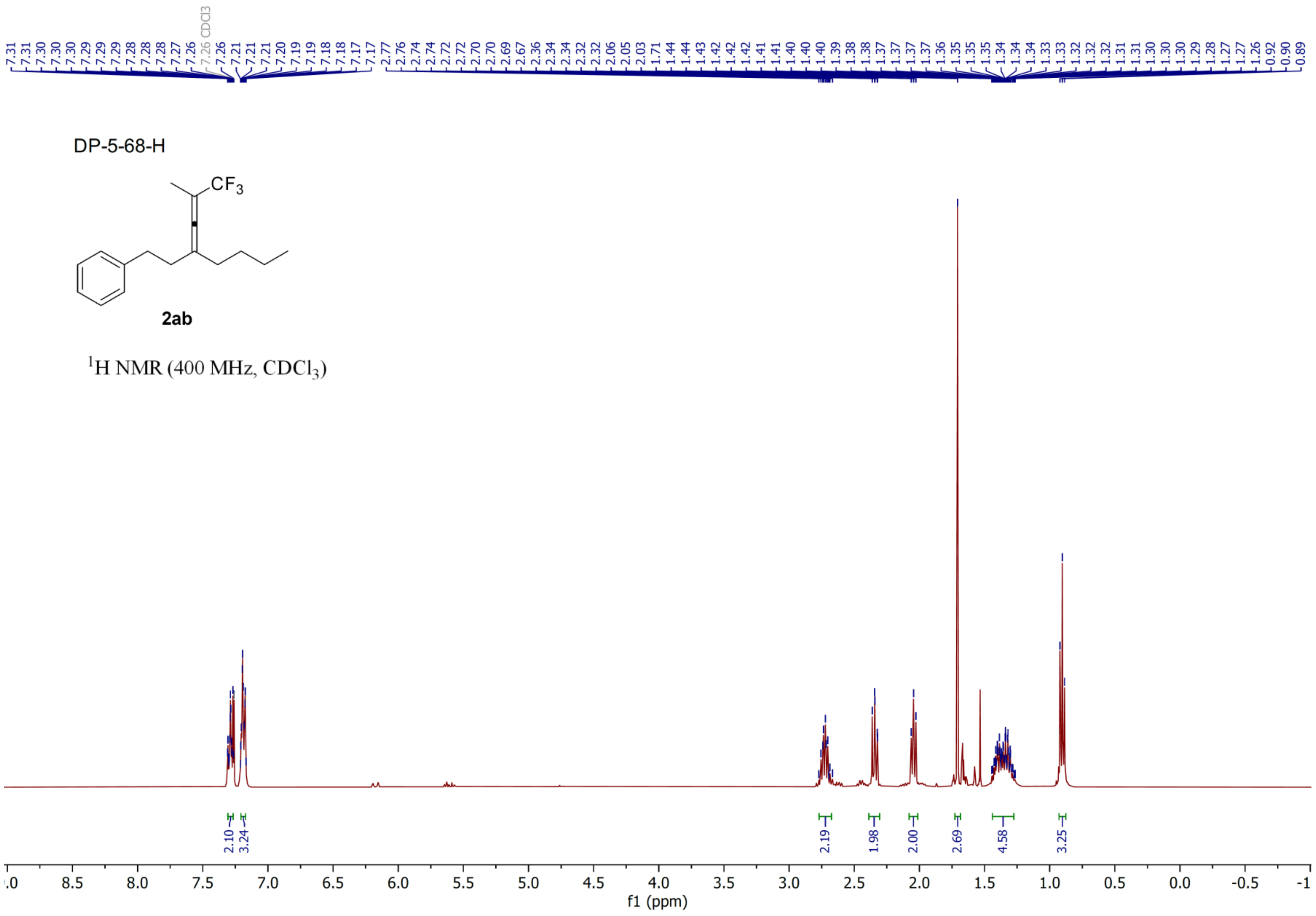
DP-5-68-H
CF3
2ab
1H NMR (400 MHz, CDCl3)
f1 (ppm)

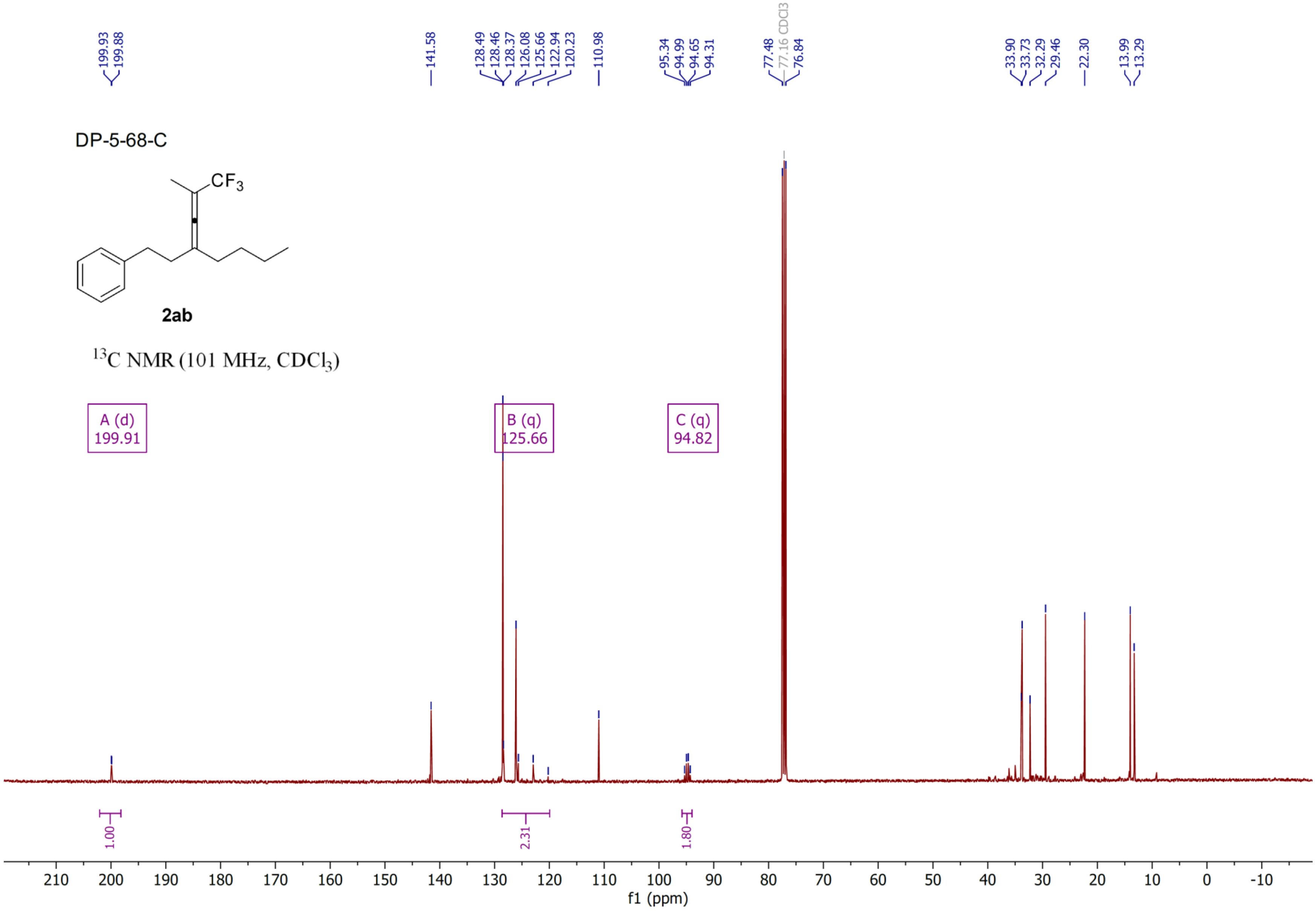

199.93
199.88
141.58
128.49
128.46
128.37
126.08
125.66
122.94
120.23
110.98
95.34
94.99
94.65
94.31
77.48
77.16 CDCl3
76.84
33.90
33.73
32.29
29.46
22.30
13.99
13.29
DP-5-68-C
CF3
2ab
13C NMR (101 MHz, CDCl3)
A (d)
199.91
B (q)
125.66
C (q)
94.82
1.00
2.31
1.80
210
200
190
180
170
160
150
140
130
120
110
100
90
80
70
60
50
40
30
20
10
0
-10
f1 (ppm)

DP-5-68-F

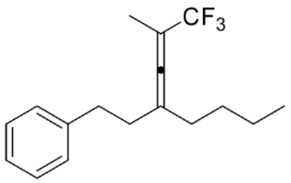

2ab

^{19}F NMR (377 MHz, $CDCl_3$)

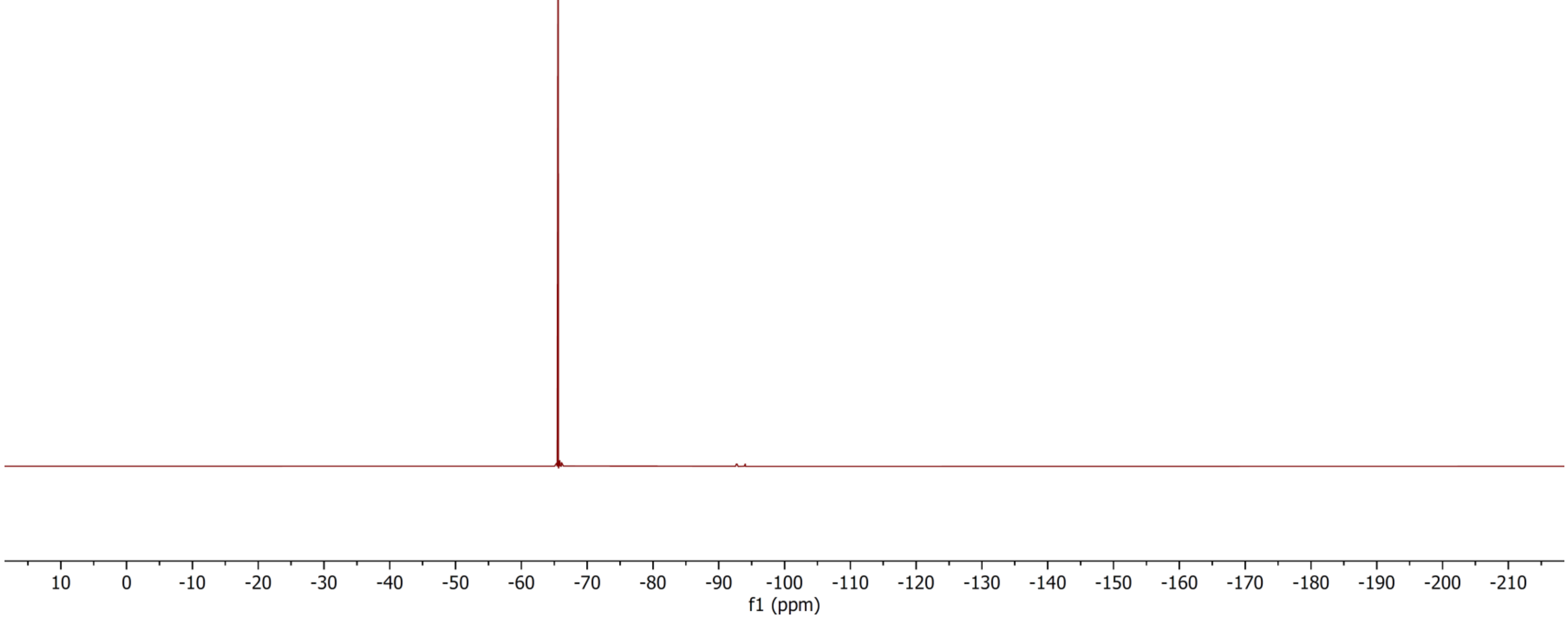

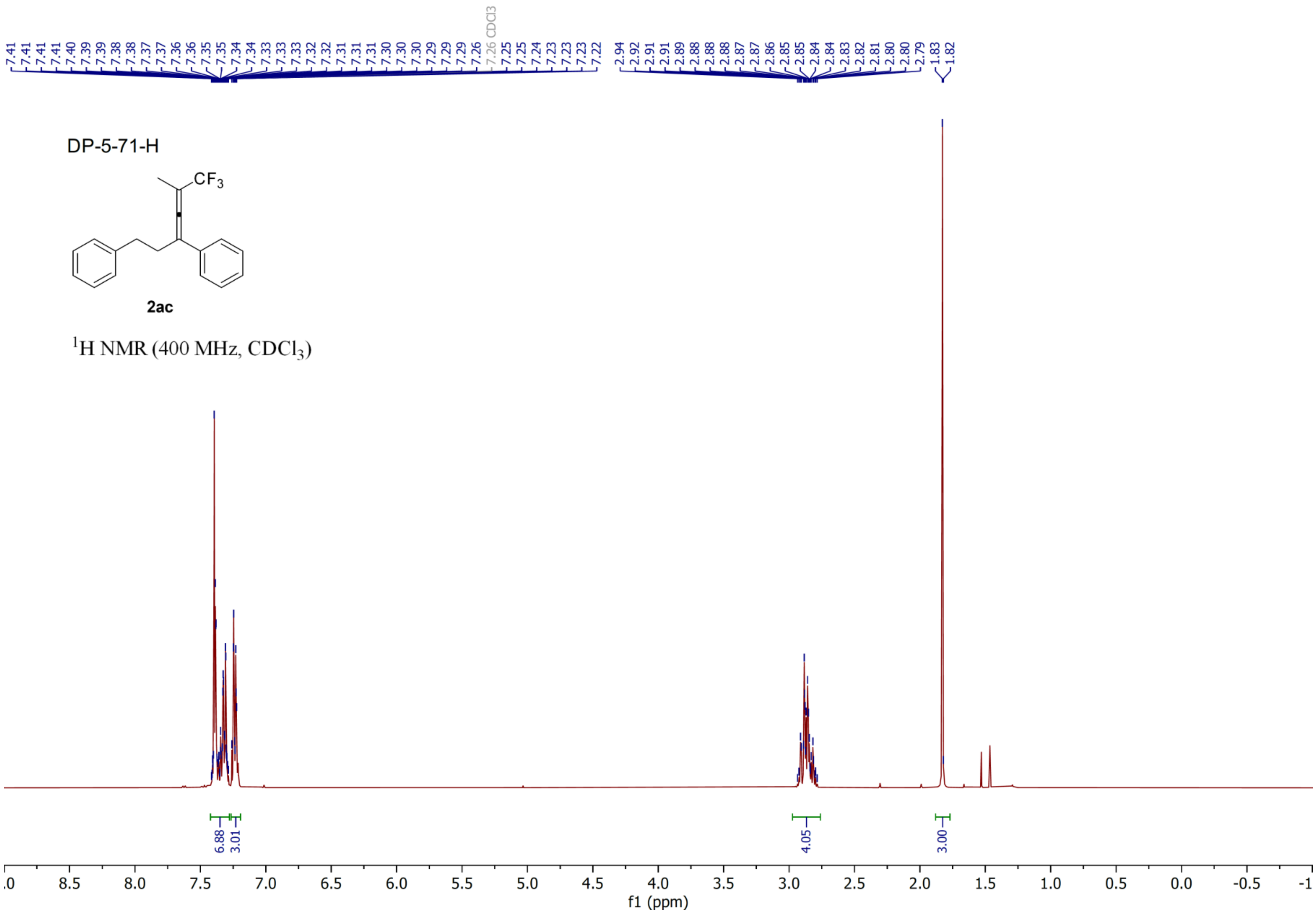
DP-5-71-H
2ac
^{1}H NMR (400 MHz, $CDCl_3$)
f1 (ppm)

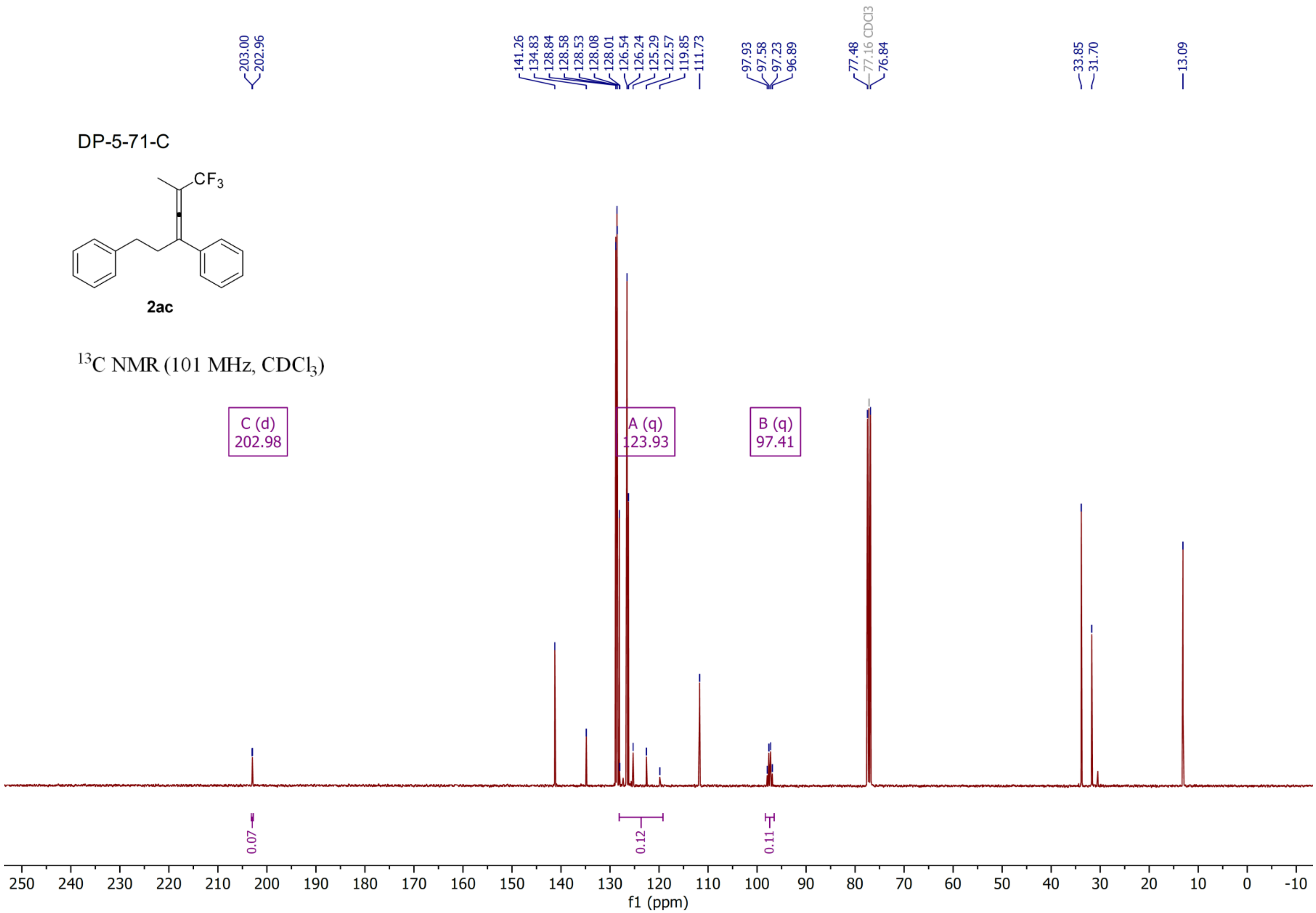

203.00
202.96
141.26
134.83
128.84
128.58
128.53
128.08
128.01
126.54
126.24
125.29
122.57
119.85
111.73
97.93
97.58
97.23
96.89
77.48
77.16 CDCl3
76.84
33.85
31.70
13.09
DP-5-71-C
CF3
2ac
13C NMR (101 MHz, CDCl3)
C (d)
202.98
A (q)
123.93
B (q)
97.41
0.07
0.12
0.11
f1 (ppm)

DP-5-71-F

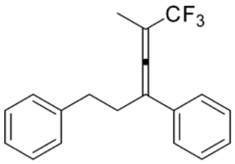

2ac

^{19}F NMR (377 MHz, $CDCl_3$)

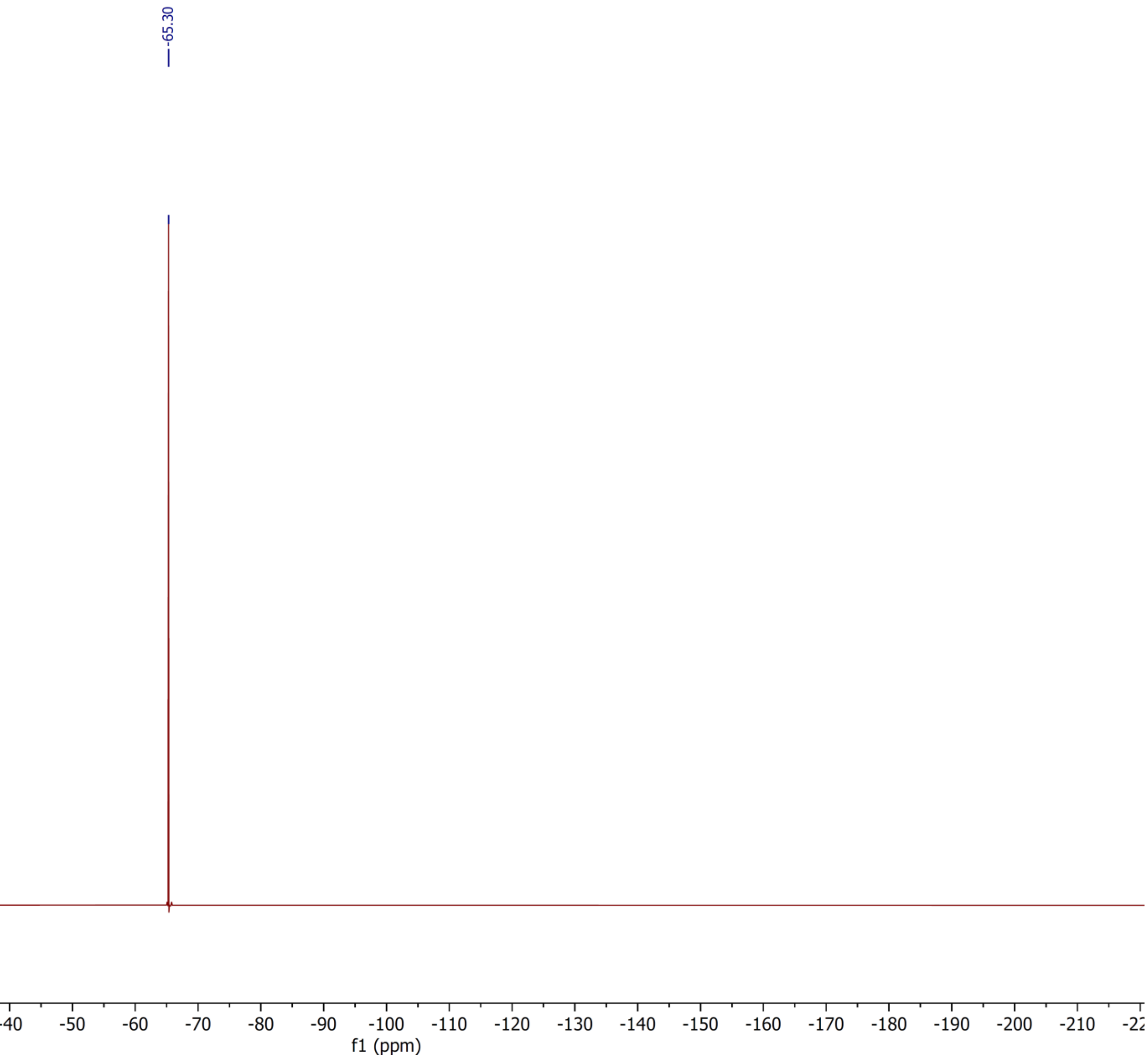

DP-5-20-H-500mhz

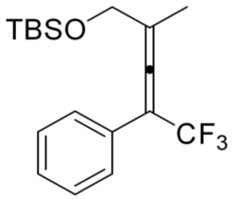

2b

^{1}H NMR (500 MHz, $CDCl_3$)

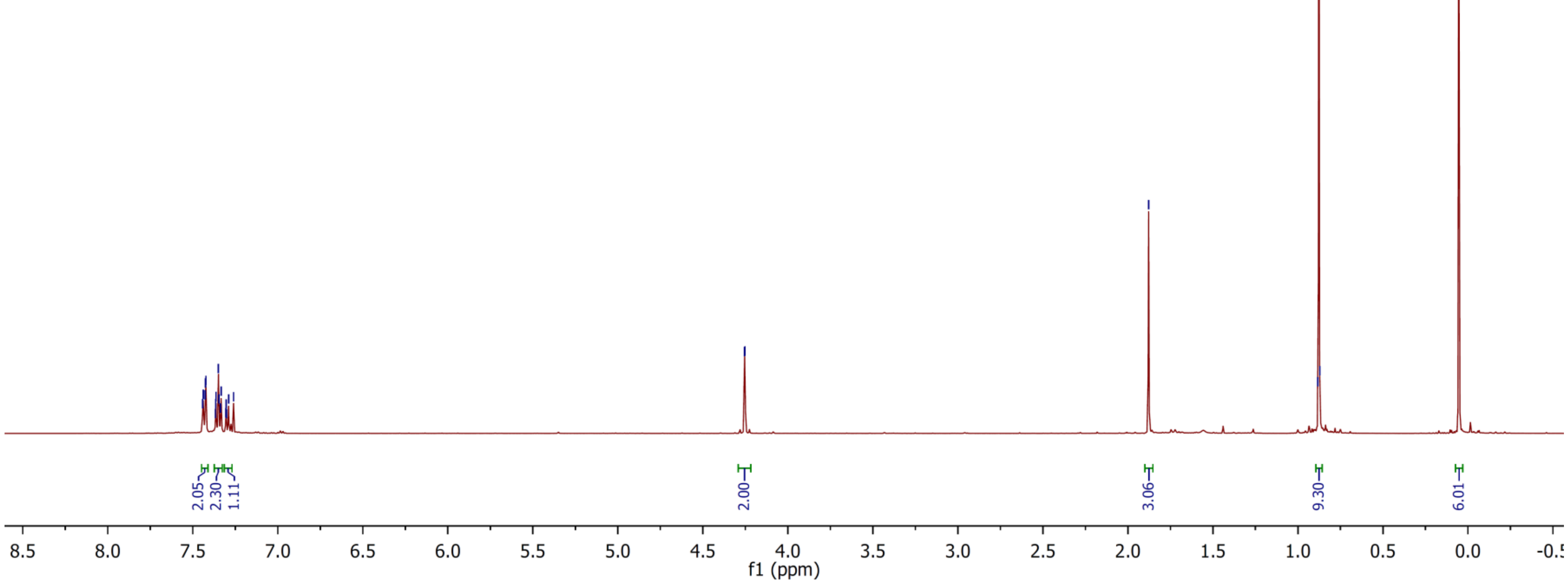

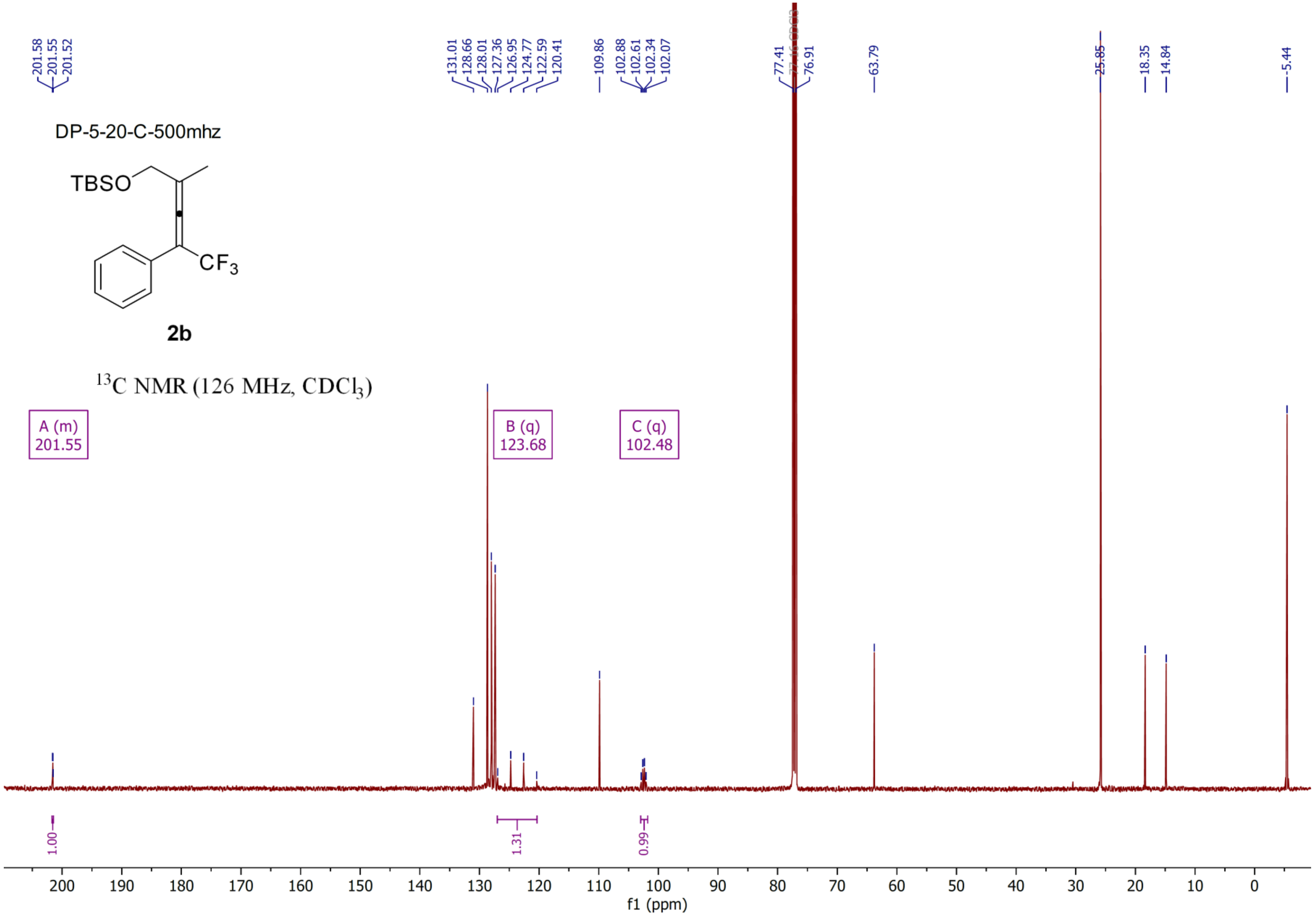

DP-5-20-C-500mhz
TBSO
CF3
2b
13C NMR (126 MHz, CDCl3)
201.58
201.55
201.52
131.01
128.66
128.01
127.36
126.95
124.77
122.59
120.41
109.86
102.88
102.61
102.34
102.07
77.41
77.16 CDCl3
76.91
63.79
25.85
18.35
14.84
-5.44
A (m)
201.55
B (q)
123.68
C (q)
102.48
1.00
1.31
0.99
200
190
180
170
160
150
140
130
120
110
100
90
80
70
60
50
40
30
20
10
0
f1 (ppm)

DP-5-20-F

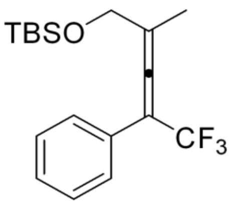

2b

^{19}F NMR (377 MHz, $CDCl_3$)

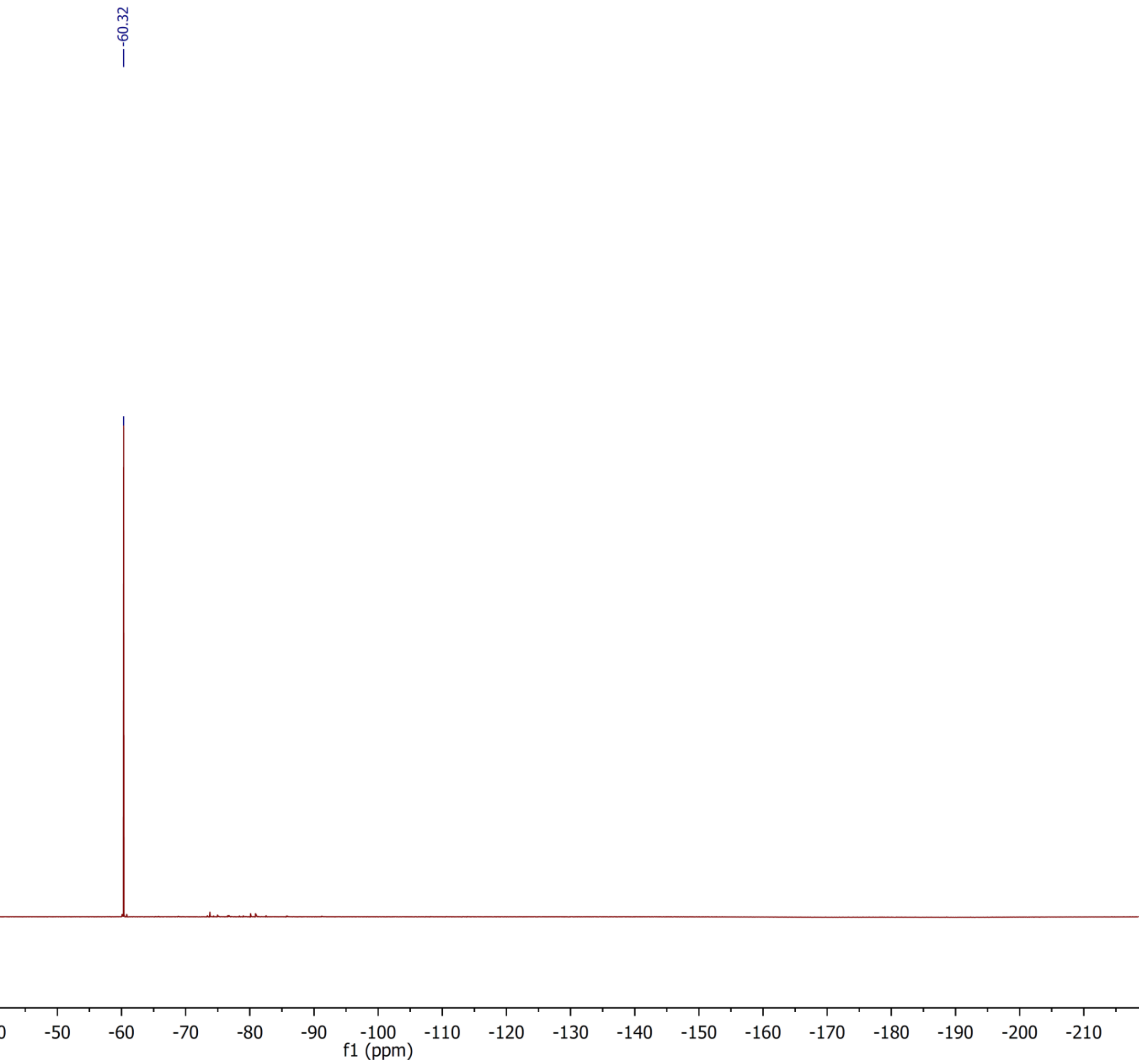

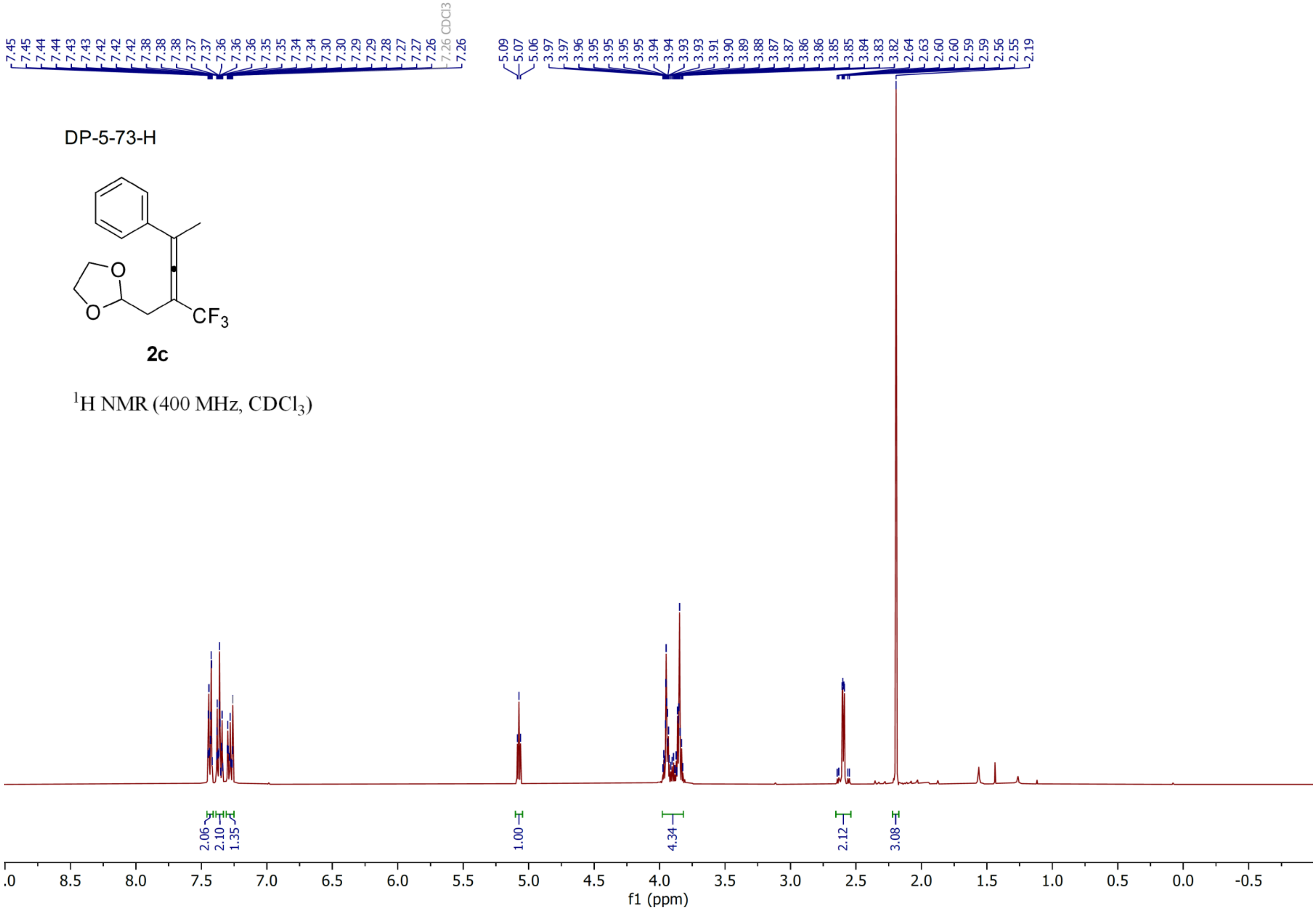

DP-5-73-H

2c

^{1}H NMR (400 MHz, $CDCl_3$)

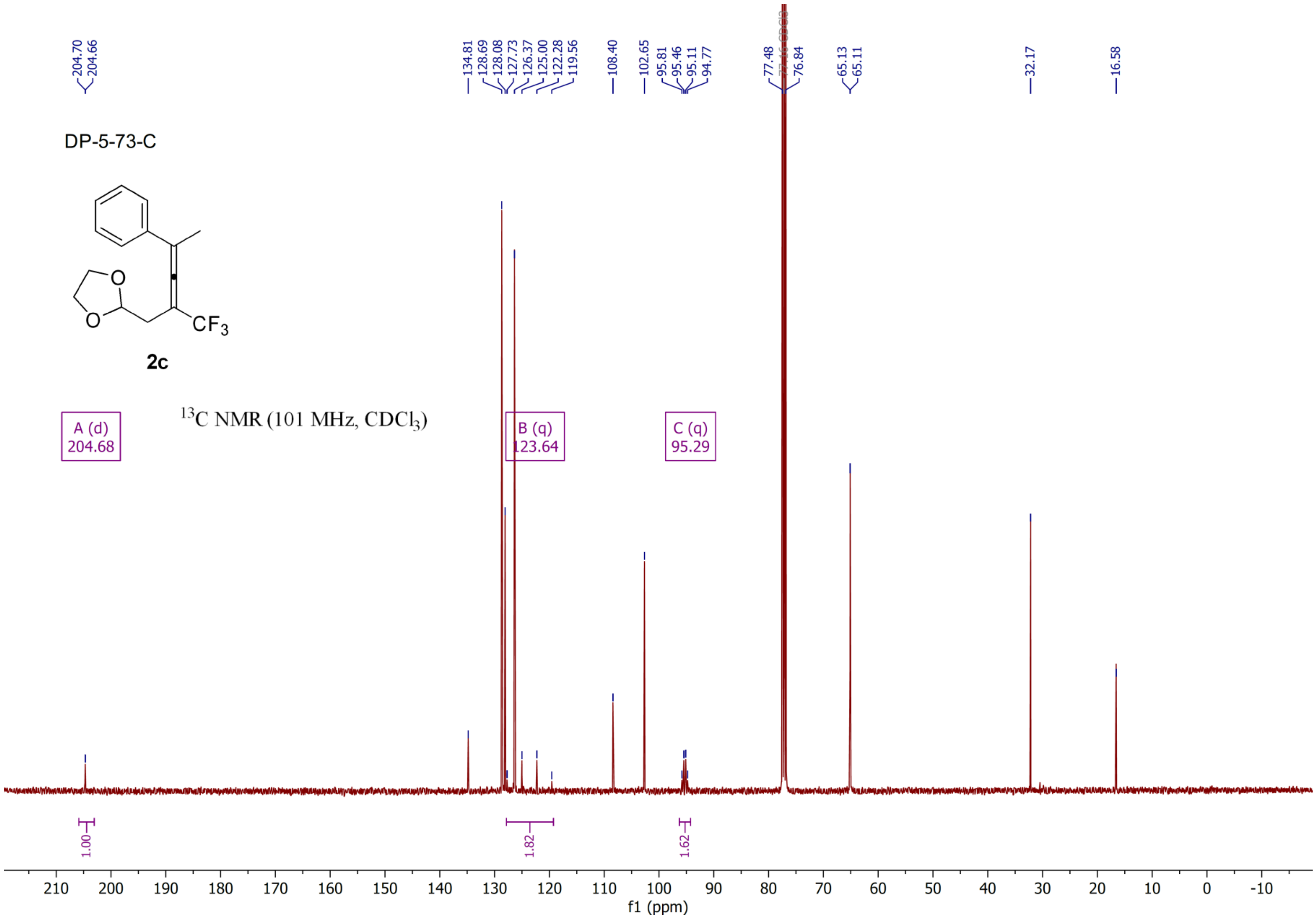

204.70
204.66
134.81
128.69
128.08
127.73
126.37
125.00
122.28
119.56
108.40
102.65
95.81
95.46
95.11
94.77
77.48
76.84
65.13
65.11
32.17
16.58
DP-5-73-C
2c
^{13}C NMR (101 MHz, $CDCl_3$)
A (d) 204.68
B (q) 123.64
C (q) 95.29
1.00
1.82
1.62
f1 (ppm)

DP-5-73-F

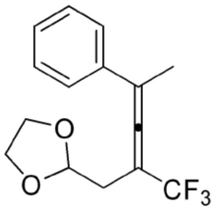

2c

^{19}F NMR (377 MHz, $CDCl_3$)

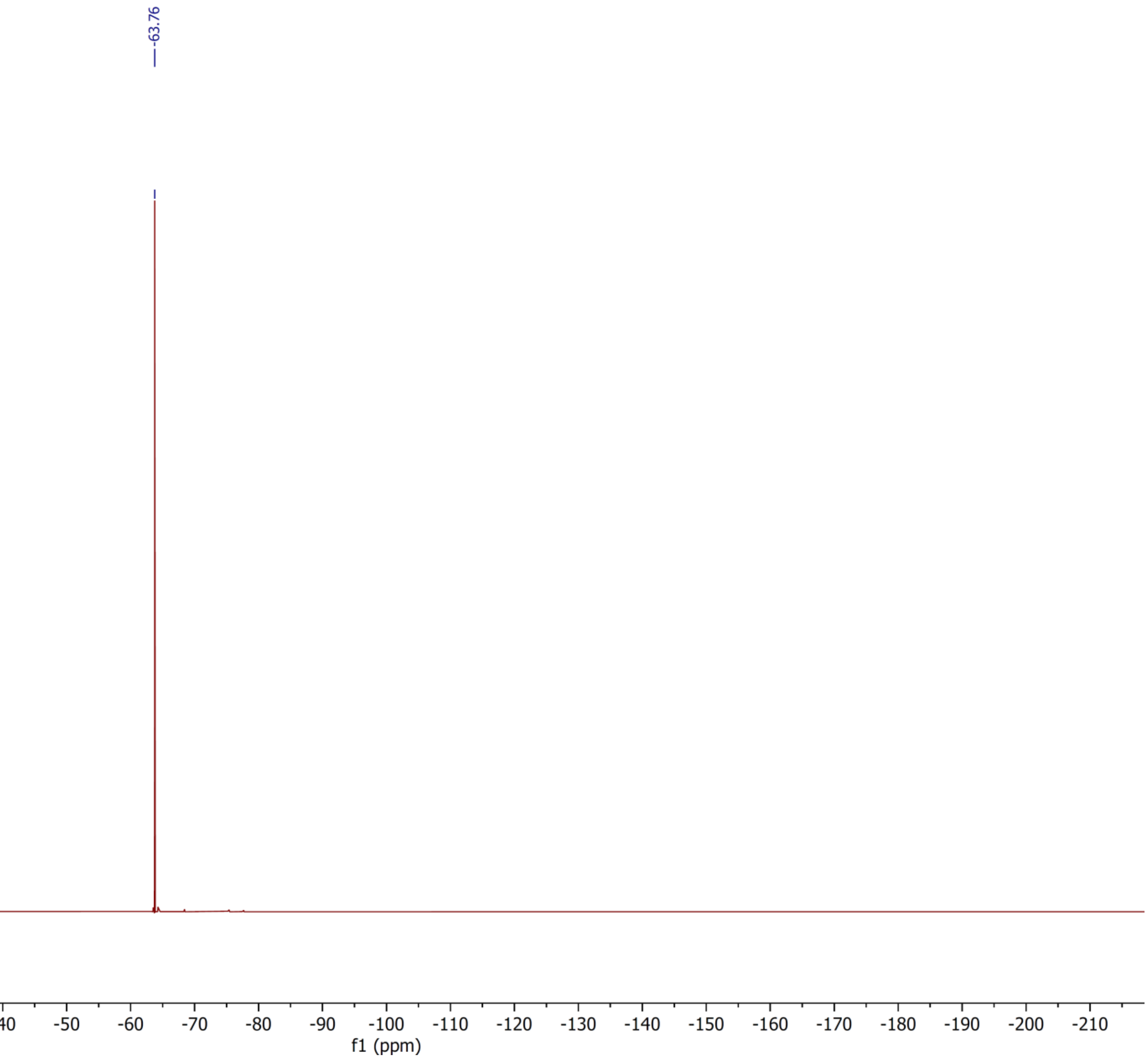

DP-5-86-H

2d

^{1}H NMR (400 MHz, $CDCl_3$)

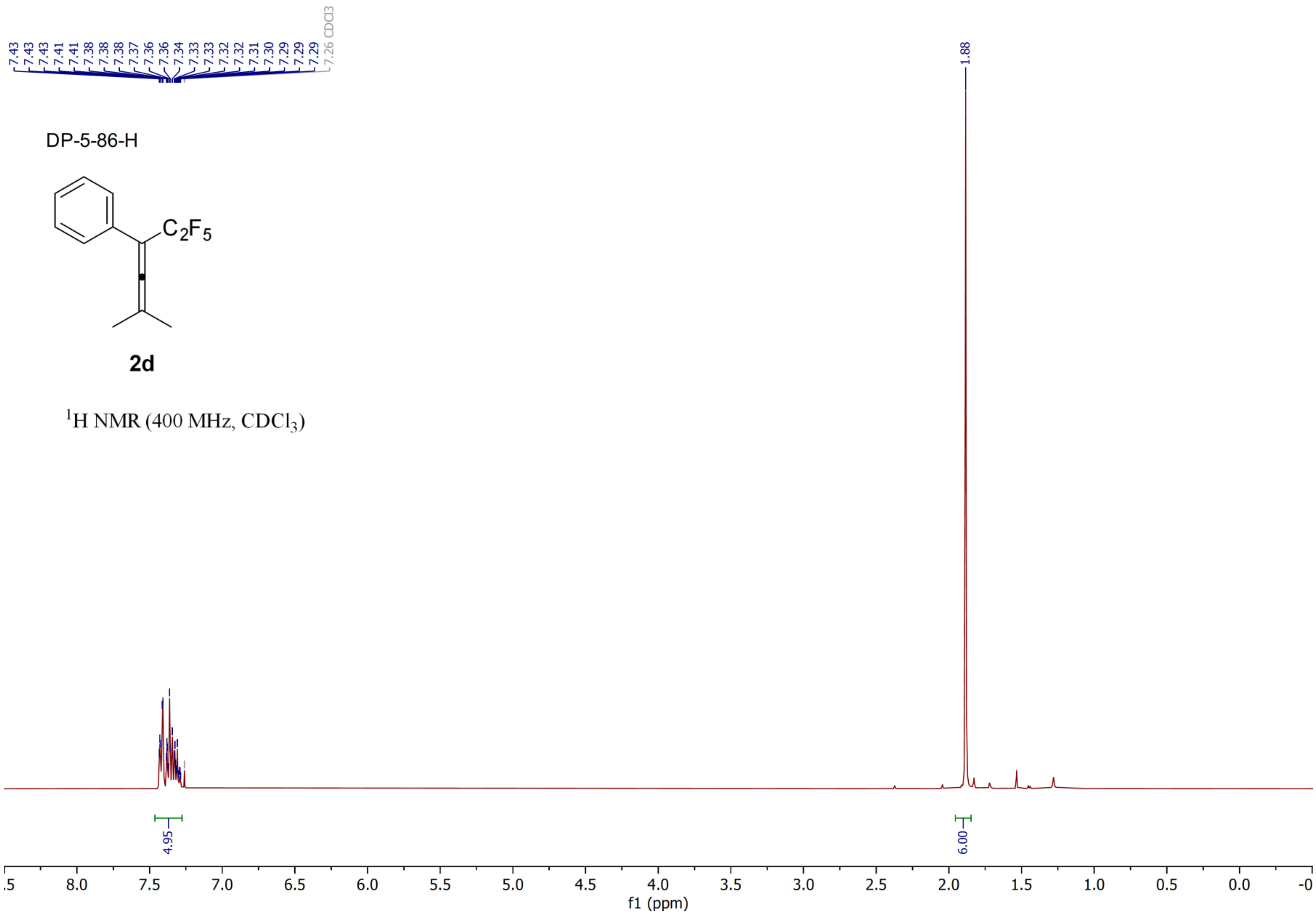

DP-5-86-C

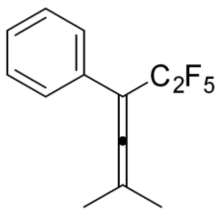

2d

^{13}C NMR (101 MHz, $CDCl_3$)

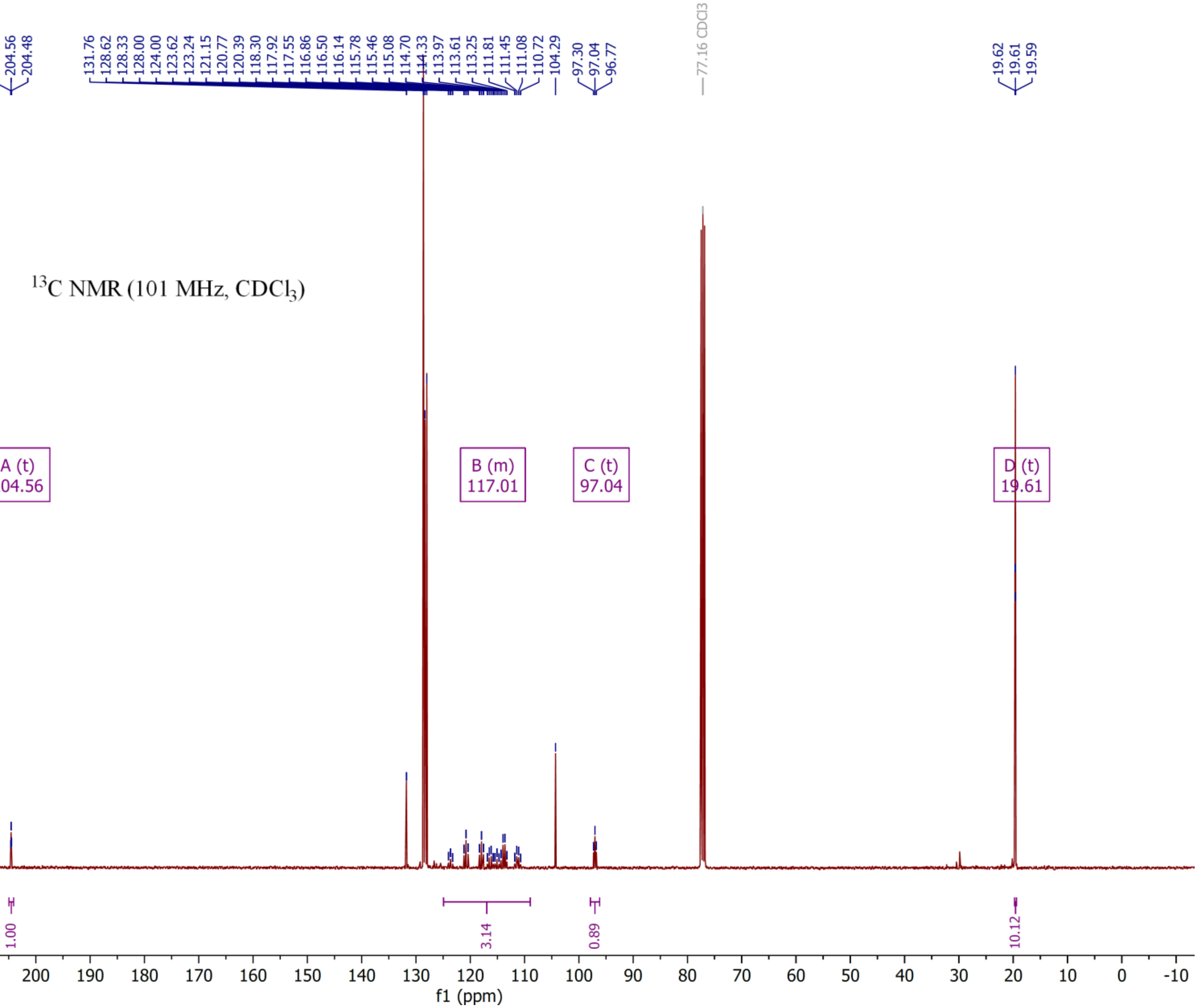

DP-5-86-F

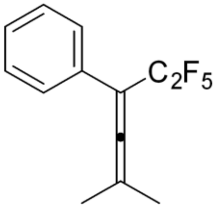

2d

^{19}F NMR (377 MHz, $CDCl_3$)

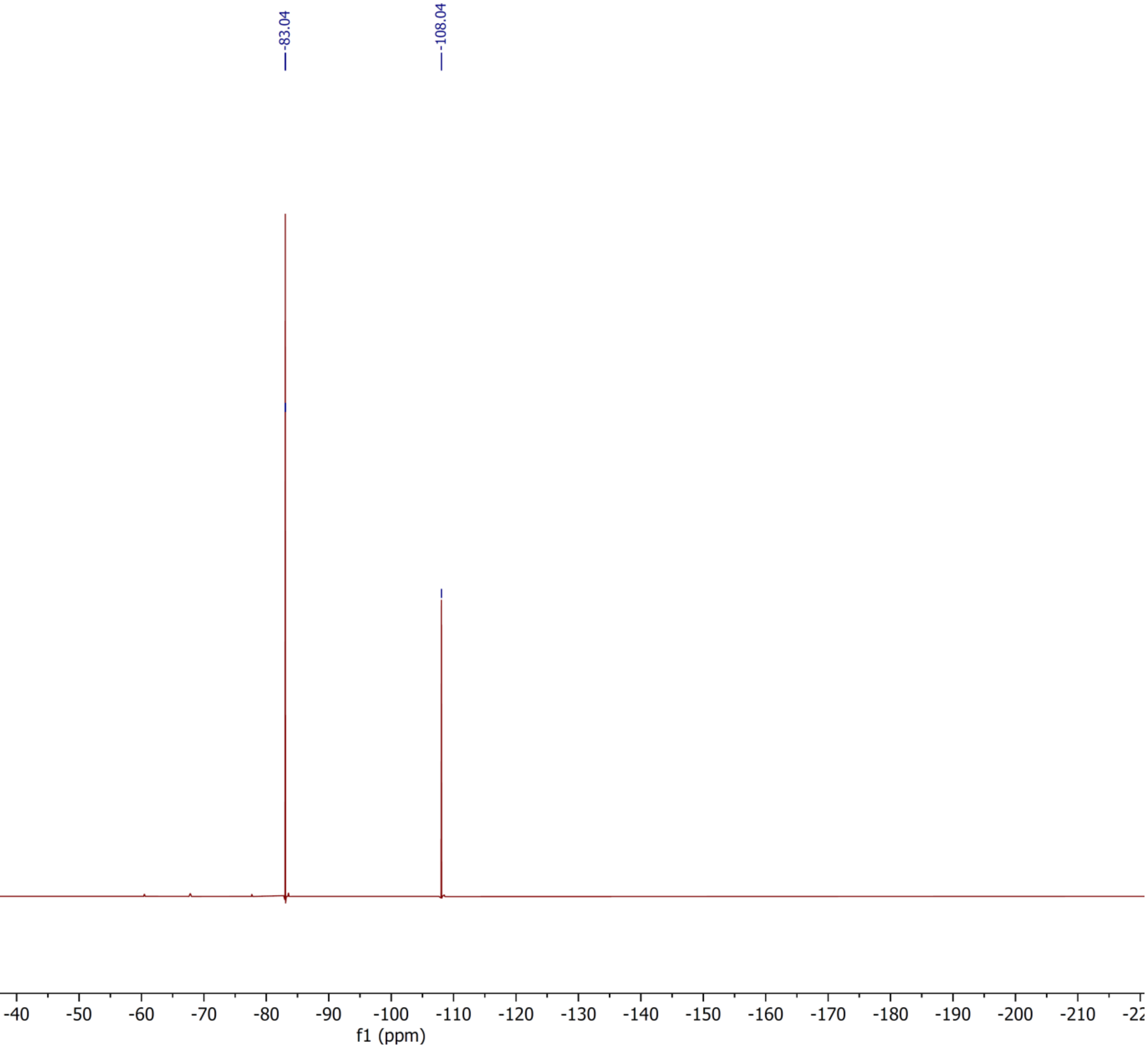

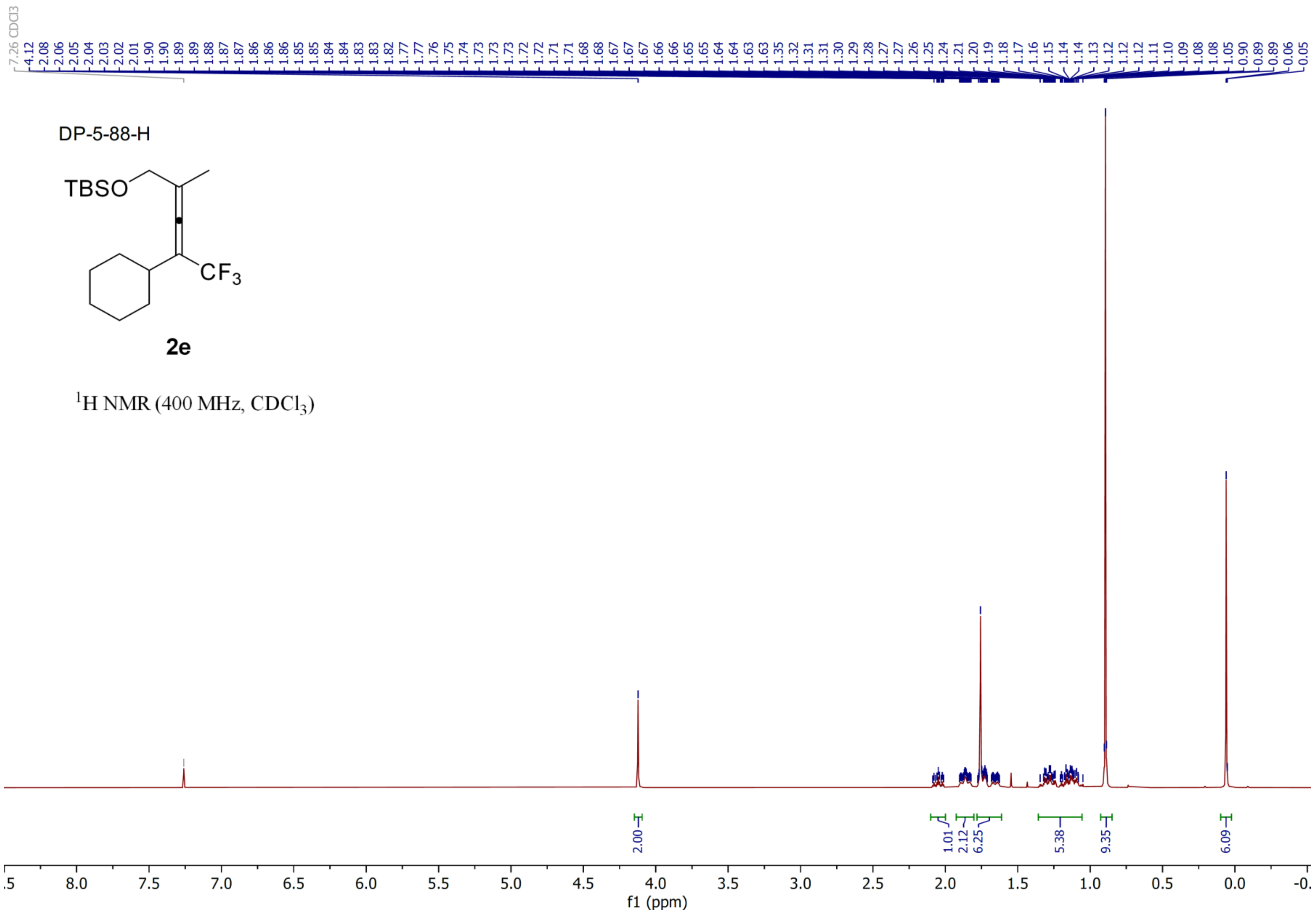
DP-5-88-H
TBSO
CF3
2e
1H NMR (400 MHz, CDCl3)
f1 (ppm)
2.00
1.01
2.12
6.25
5.38
9.35
6.09

DP-5-88-C

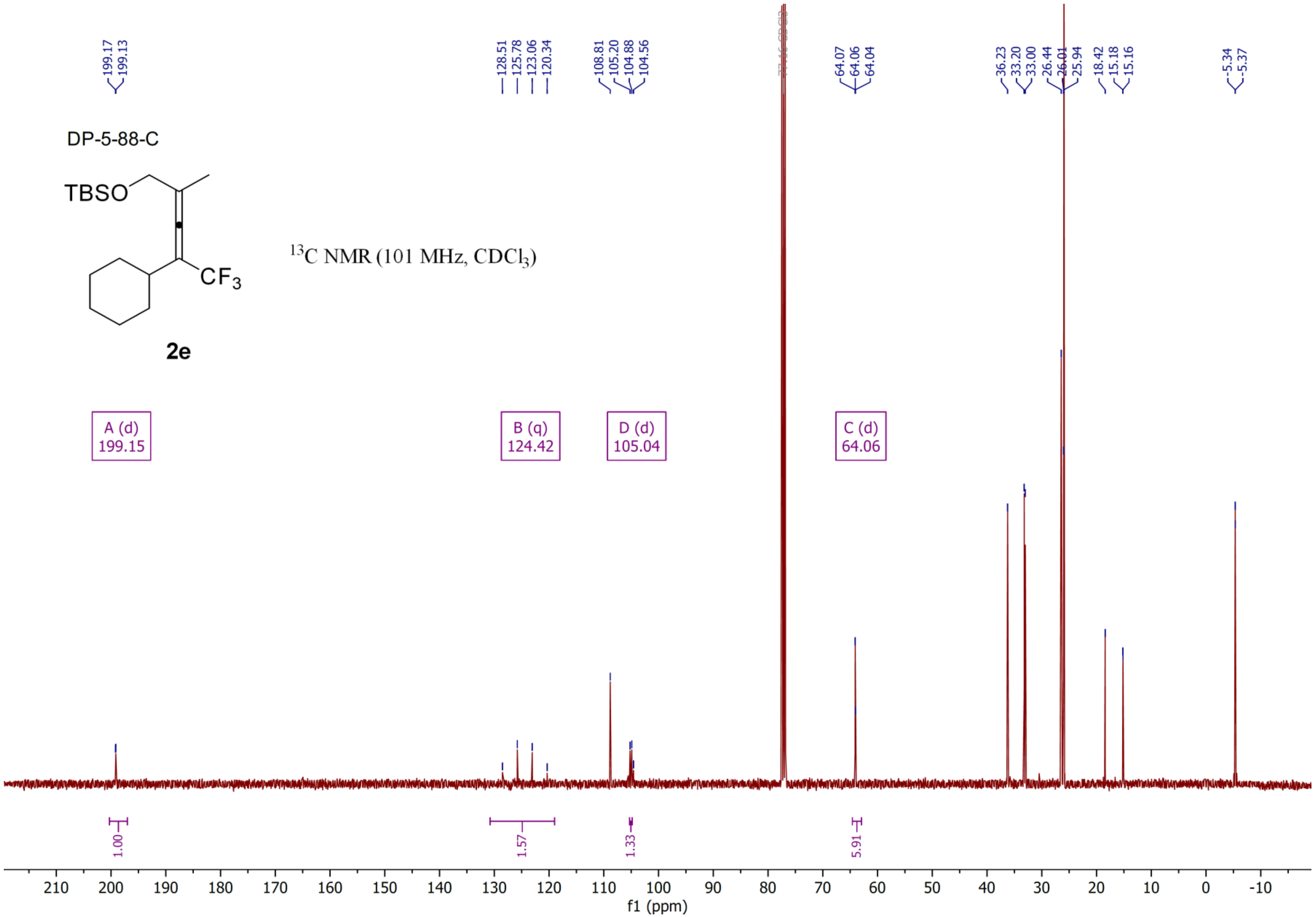

TBSO
CF3
2e
13C NMR (101 MHz, CDCl3)
199.17
199.13
128.51
125.78
123.06
120.34
108.81
105.20
104.88
104.56
64.07
64.06
64.04
36.23
33.20
33.00
26.44
26.01
25.94
18.42
15.18
15.16
-5.34
-5.37
A (d)
199.15
B (q)
124.42
D (d)
105.04
C (d)
64.06
1.00
1.57
1.33
5.91
210
200
190
180
170
160
150
140
130
120
110
100
90
80
70
60
50
40
30
20
10
0
-10
f1 (ppm)

DP-5-88-F

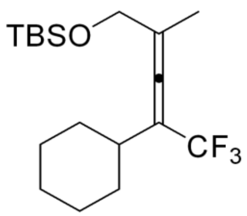

2e

^{19}F NMR (377 MHz, $CDCl_3$)

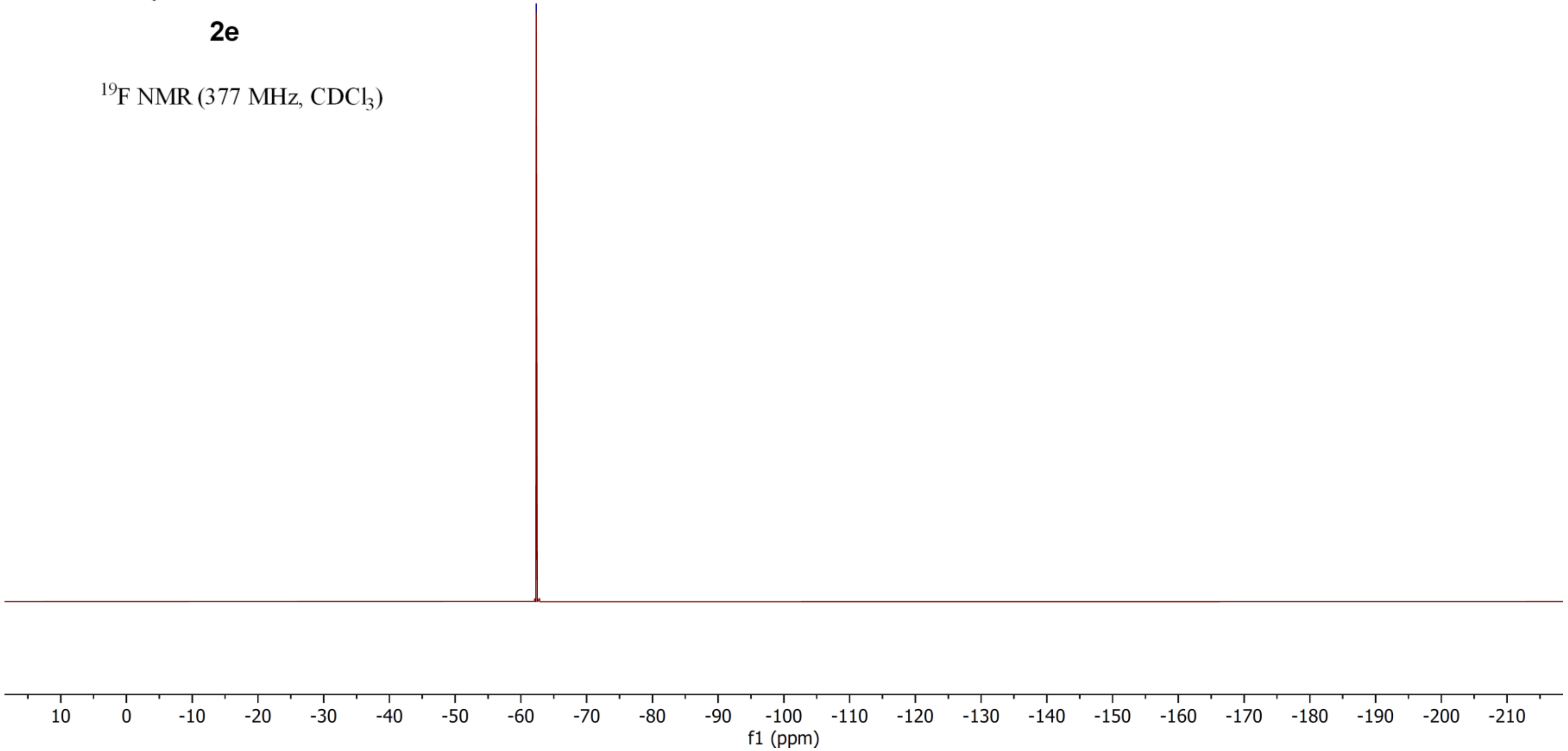

DP-5-99-H

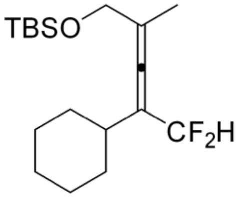

2f

^{1}H NMR (400 MHz, $CDCl_3$)

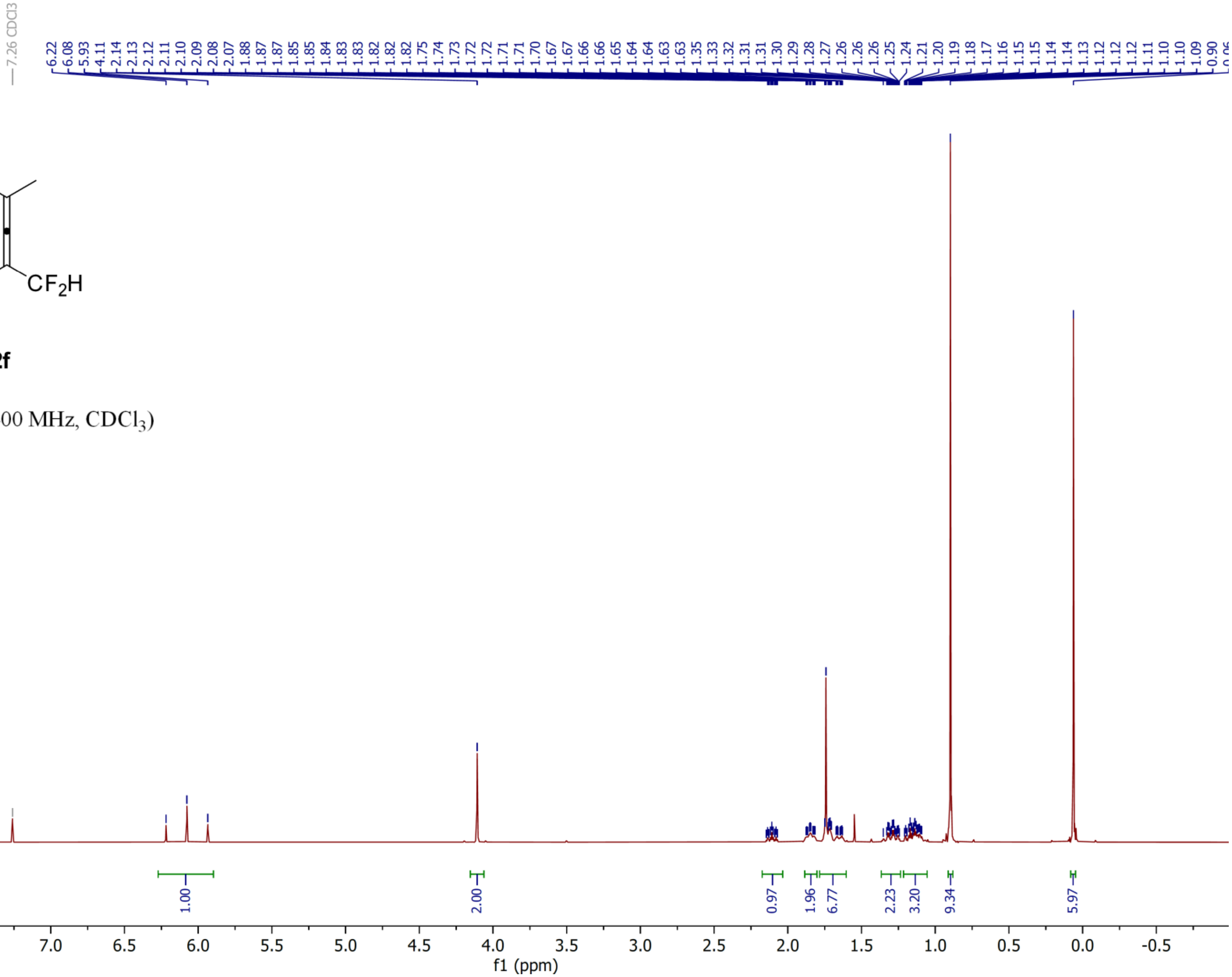

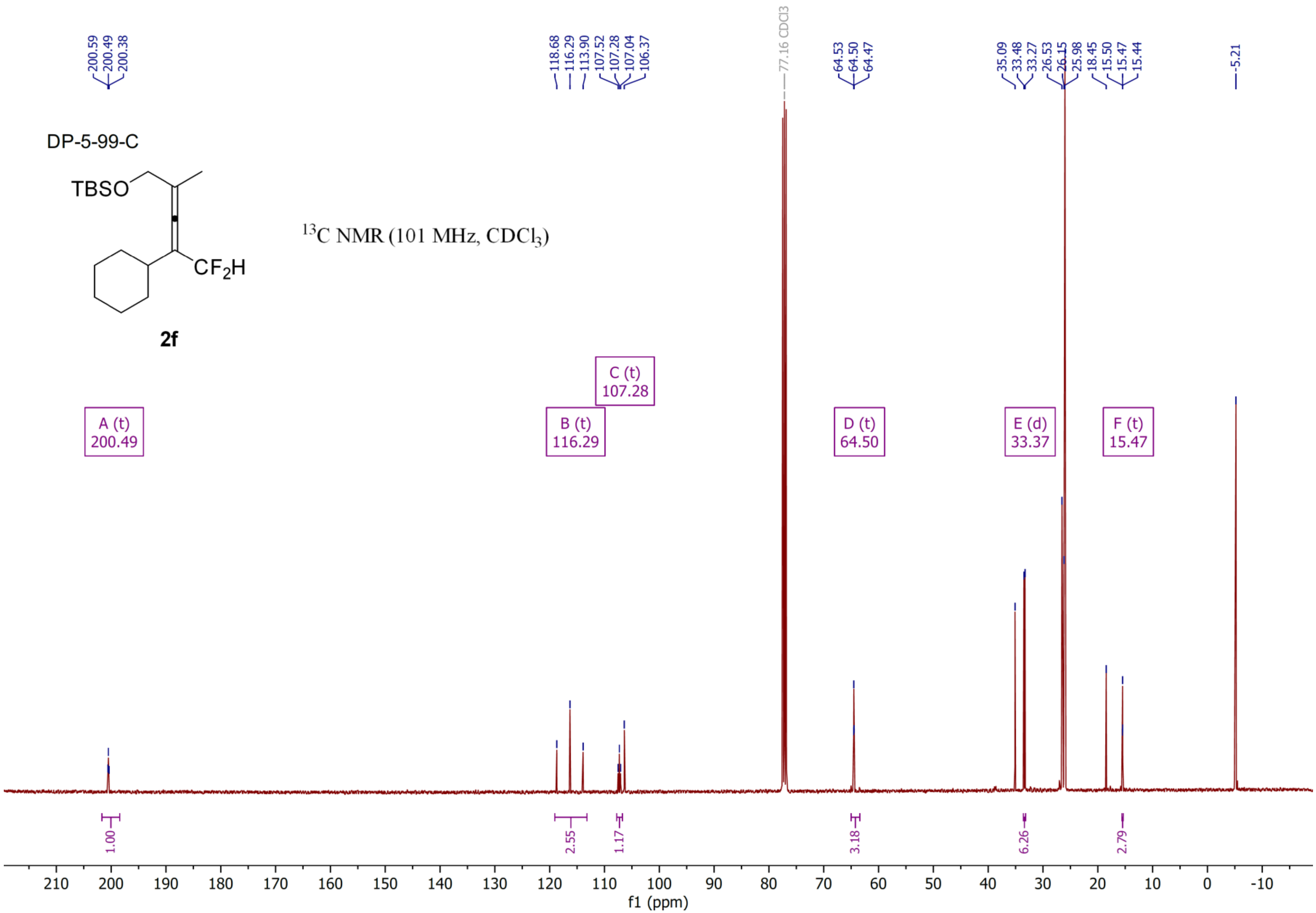
DP-5-99-C
TBSO
CF_2H
2f
^{13}C NMR (101 MHz, $CDCl_3$)
200.59
200.49
200.38
118.68
116.29
113.90
107.52
107.28
107.04
106.37
77.16 CDCl3
64.53
64.50
64.47
35.09
33.48
33.27
26.53
26.15
25.98
18.45
15.50
15.47
15.44
-5.21
A (t) 200.49
B (t) 116.29
C (t) 107.28
D (t) 64.50
E (d) 33.37
F (t) 15.47
1.00
2.55
1.17
3.18
6.26
2.79
210 200 190 180 170 160 150 140 130 120 110 100 90 80 70 60 50 40 30 20 10 0 -10
f1 (ppm)

DP-5-99-F

2f

^{19}F NMR (377 MHz, $CDCl_3$)

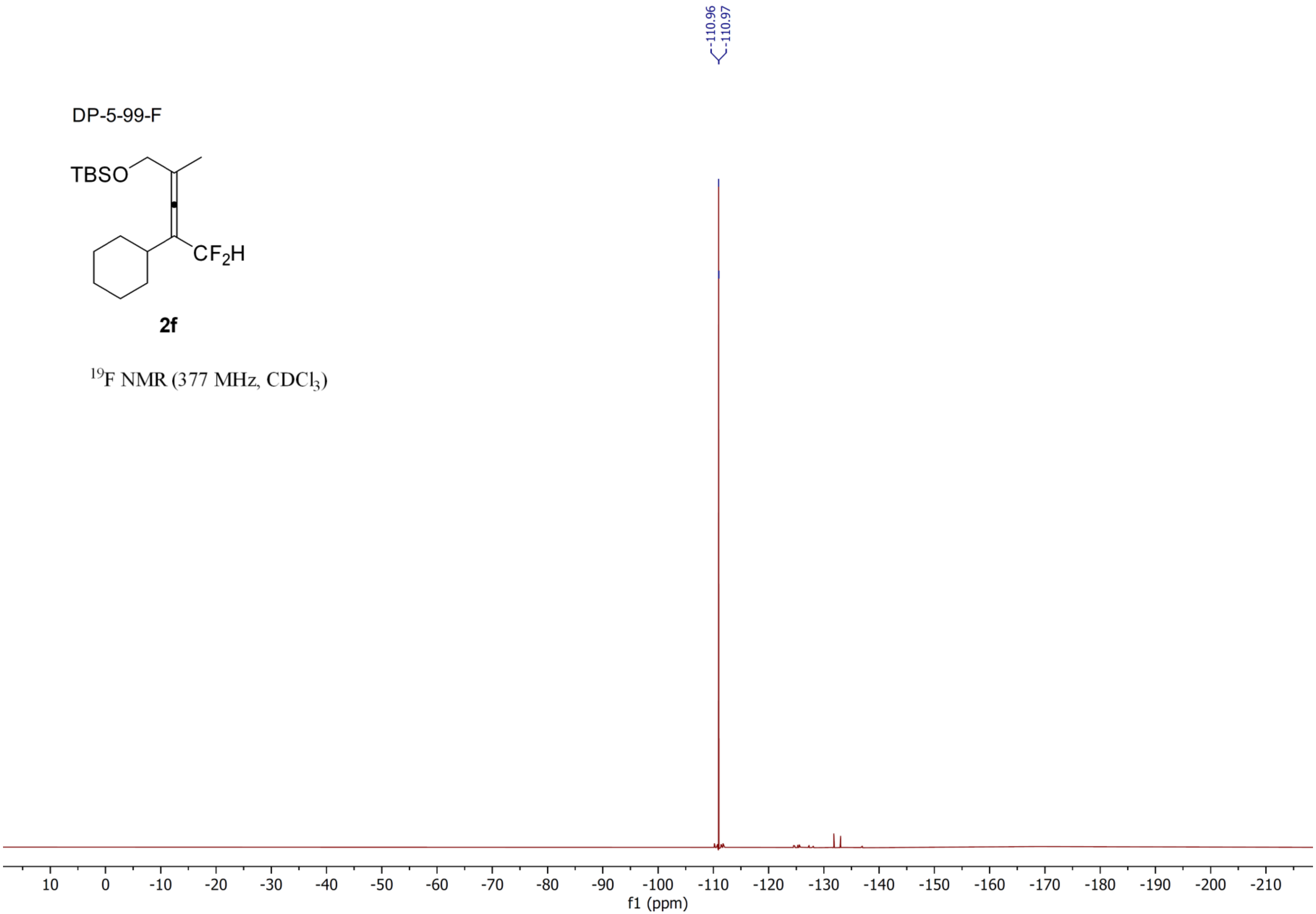

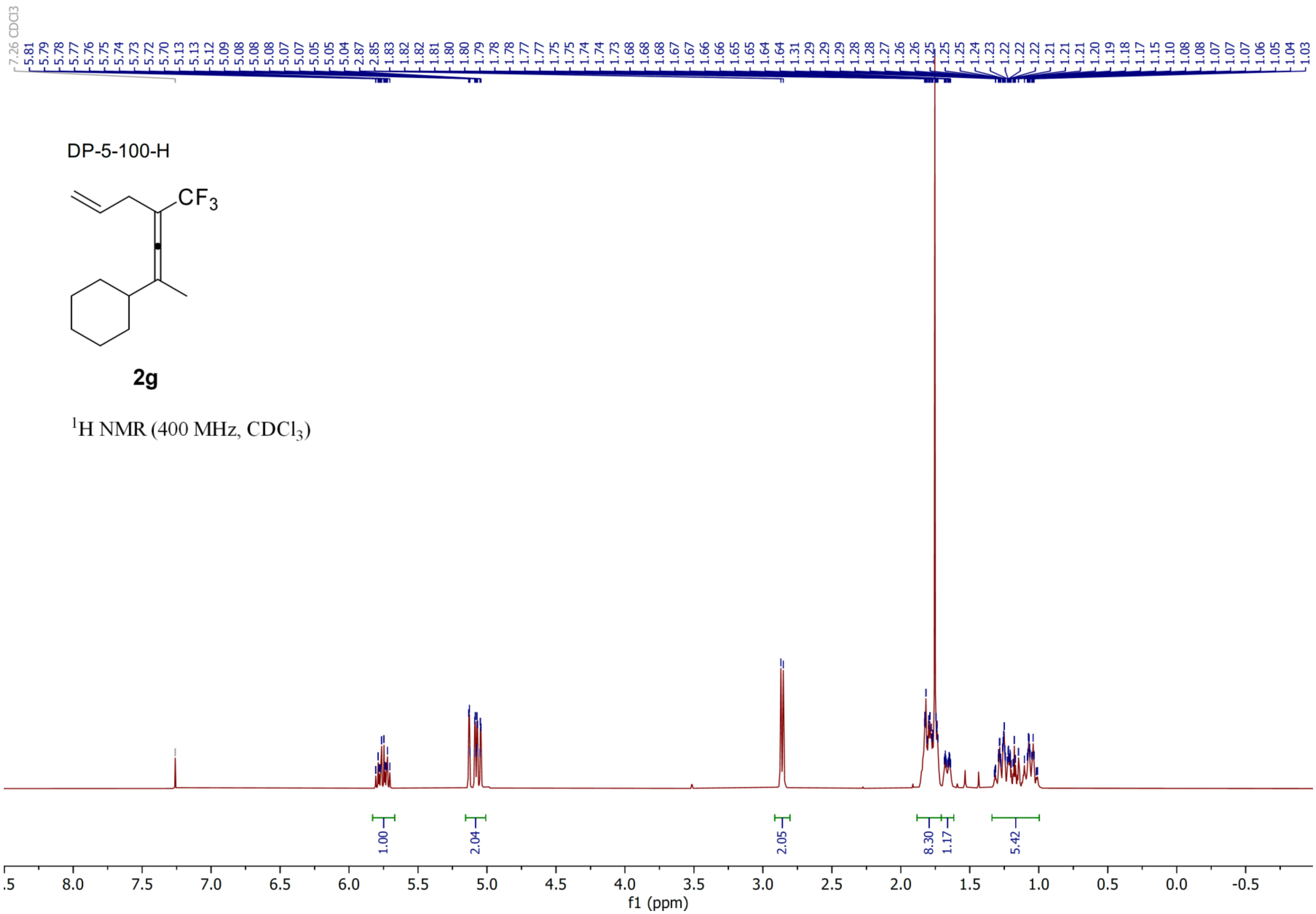
7.26 CDCl3
5.81 5.79 5.78 5.77 5.76 5.75 5.74 5.73 5.72 5.70 5.13 5.13 5.12 5.09 5.08 5.08 5.08 5.07 5.07 5.05 5.05 5.04 2.87 2.85 1.83 1.82 1.82 1.81 1.80 1.80 1.79 1.78 1.78 1.77 1.77 1.75 1.75 1.74 1.74 1.73 1.68 1.68 1.68 1.67 1.67 1.66 1.66 1.65 1.65 1.64 1.64 1.31 1.29 1.29 1.29 1.28 1.28 1.27 1.26 1.26 1.25 1.25 1.25 1.24 1.23 1.22 1.22 1.22 1.22 1.21 1.21 1.21 1.20 1.19 1.18 1.17 1.15 1.10 1.08 1.08 1.07 1.07 1.07 1.06 1.05 1.04 1.03
DP-5-100-H
CF3
2g
1H NMR (400 MHz, CDCl3)
1.00
2.04
2.05
8.30
1.17
5.42
.5 8.0 7.5 7.0 6.5 6.0 5.5 5.0 4.5 4.0 3.5 3.0 2.5 2.0 1.5 1.0 0.5 0.0 -0.5
f1 (ppm)

DP-5-100-C

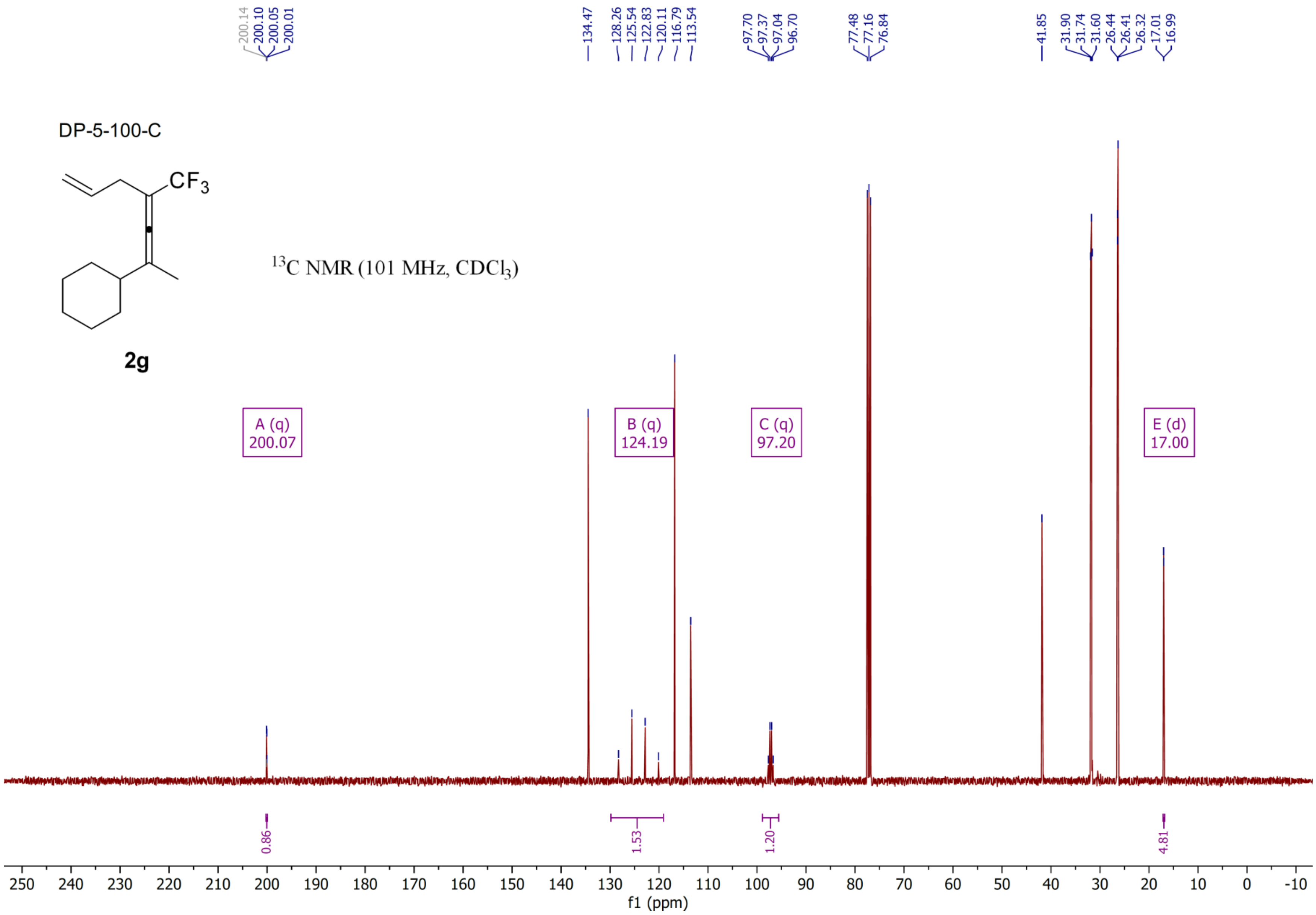

CF3
2g
13C NMR (101 MHz, CDCl3)
200.14
200.10
200.05
200.01
134.47
128.26
125.54
122.83
120.11
116.79
113.54
97.70
97.37
97.04
96.70
77.48
77.16
76.84
41.85
31.90
31.74
31.60
26.44
26.41
26.32
17.01
16.99
A (q)
200.07
B (q)
124.19
C (q)
97.20
E (d)
17.00
0.86
1.53
1.20
4.81
250
240
230
220
210
200
190
180
170
160
150
140
130
120
110
100
90
80
70
60
50
40
30
20
10
0
-10
f1 (ppm)

DP-5-100-F

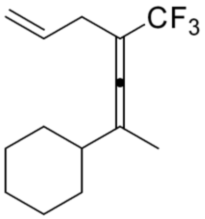

2g

^{19}F NMR (377 MHz, $CDCl_3$)

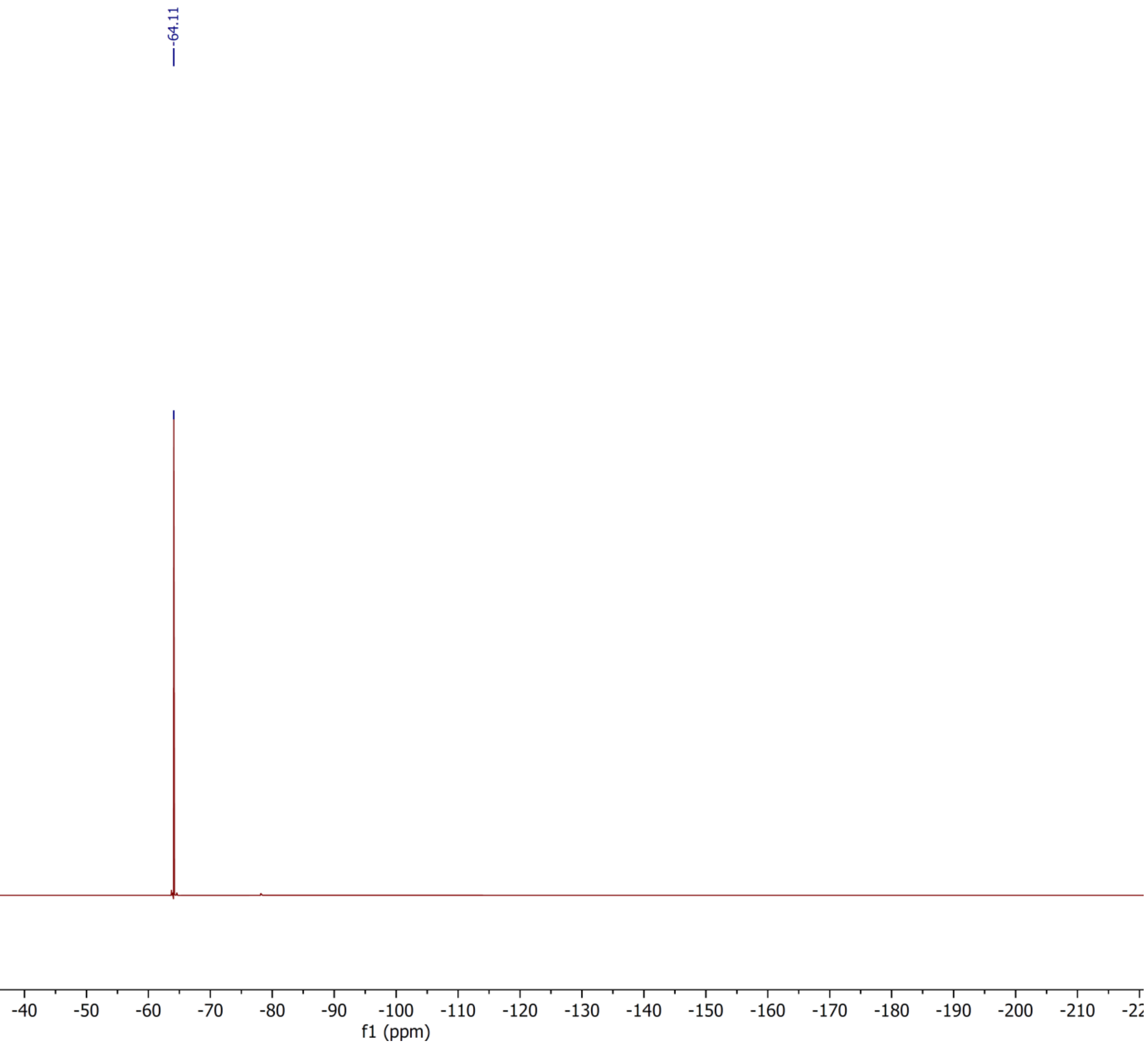

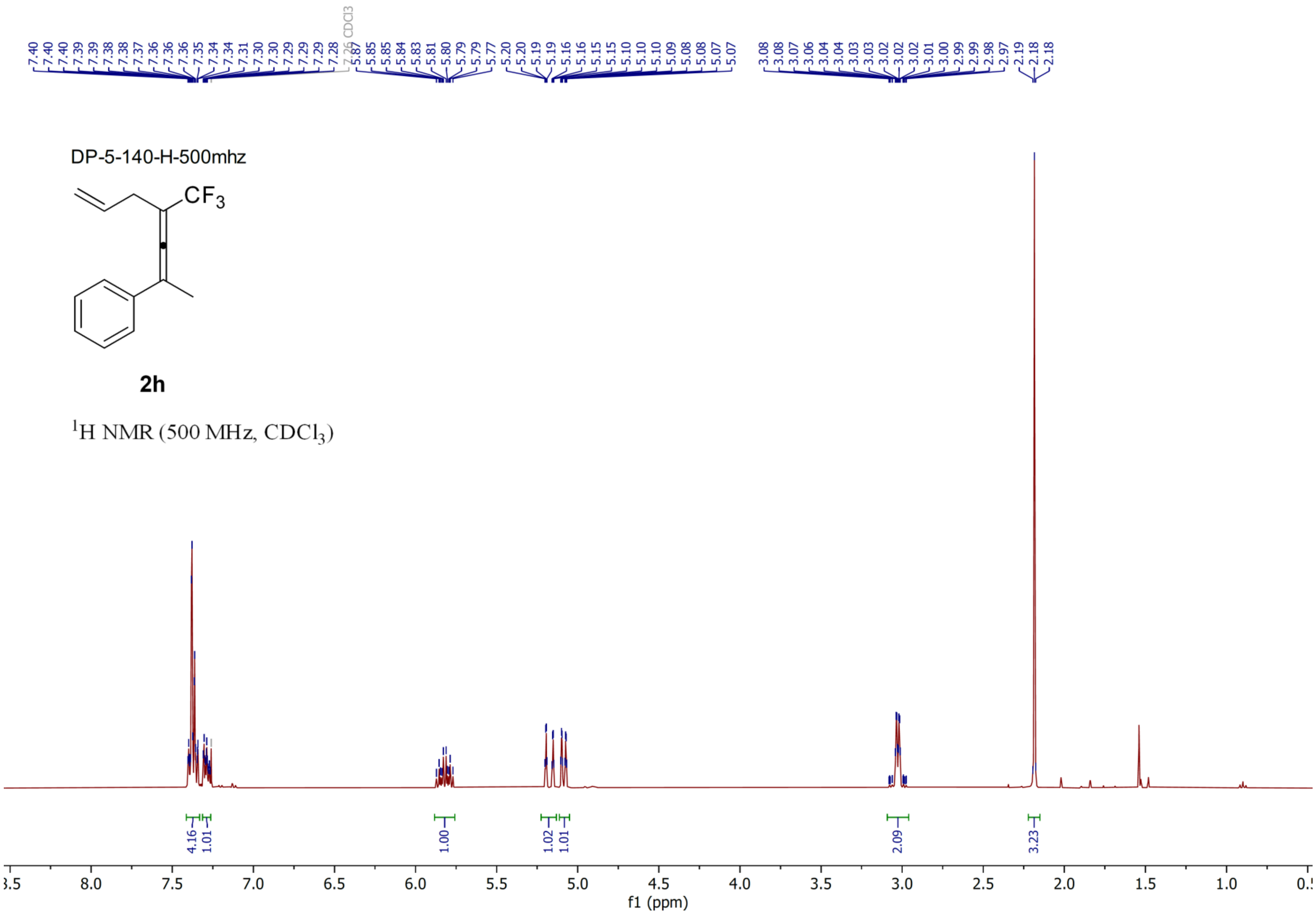
DP-5-140-H-500mhz
CF3
2h
1H NMR (500 MHz, CDCl3)
f1 (ppm)

DP-5-140-C-500mhz

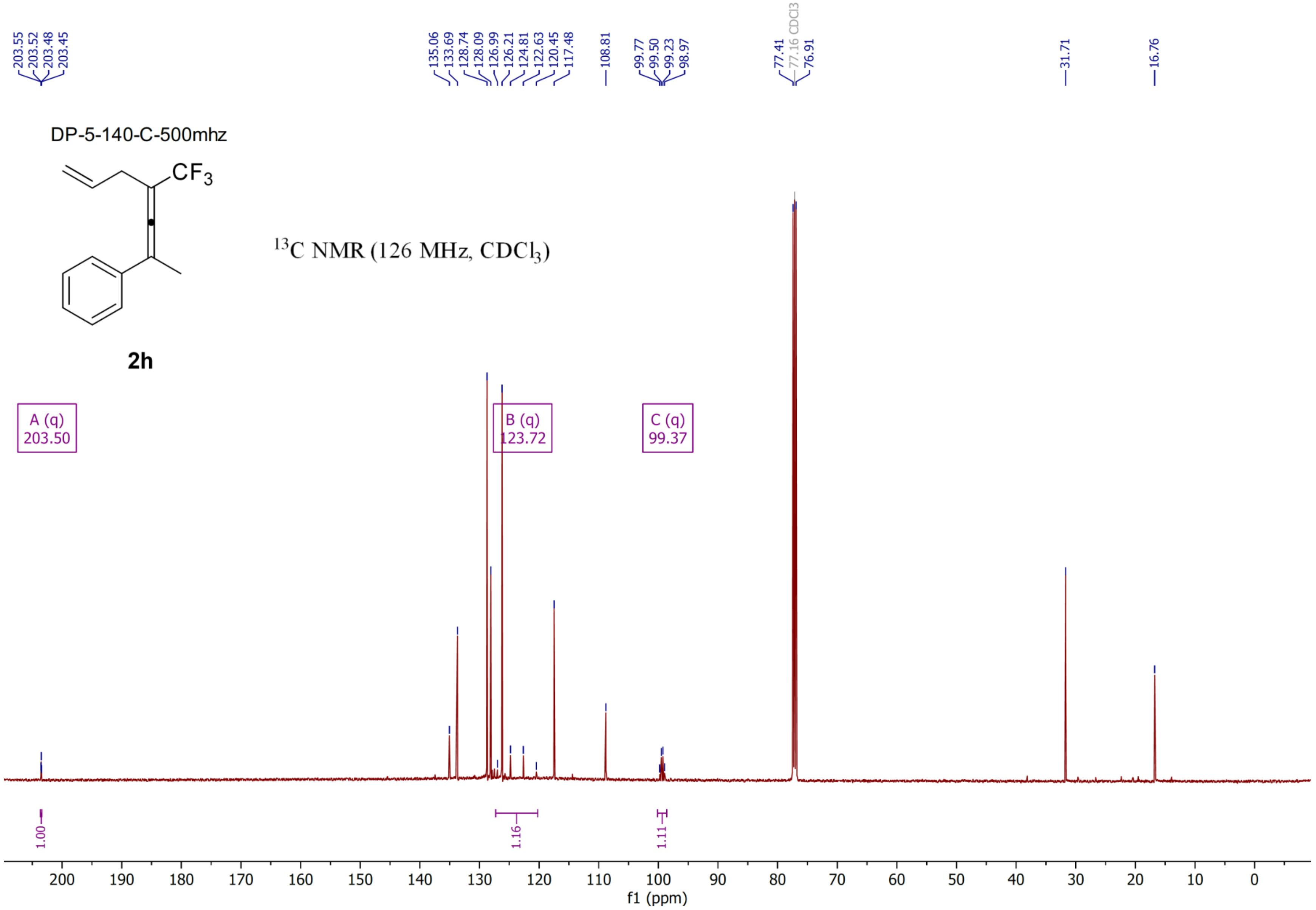

DP-5-140-F

2h

^{19}F NMR (377 MHz, $CDCl_3$)

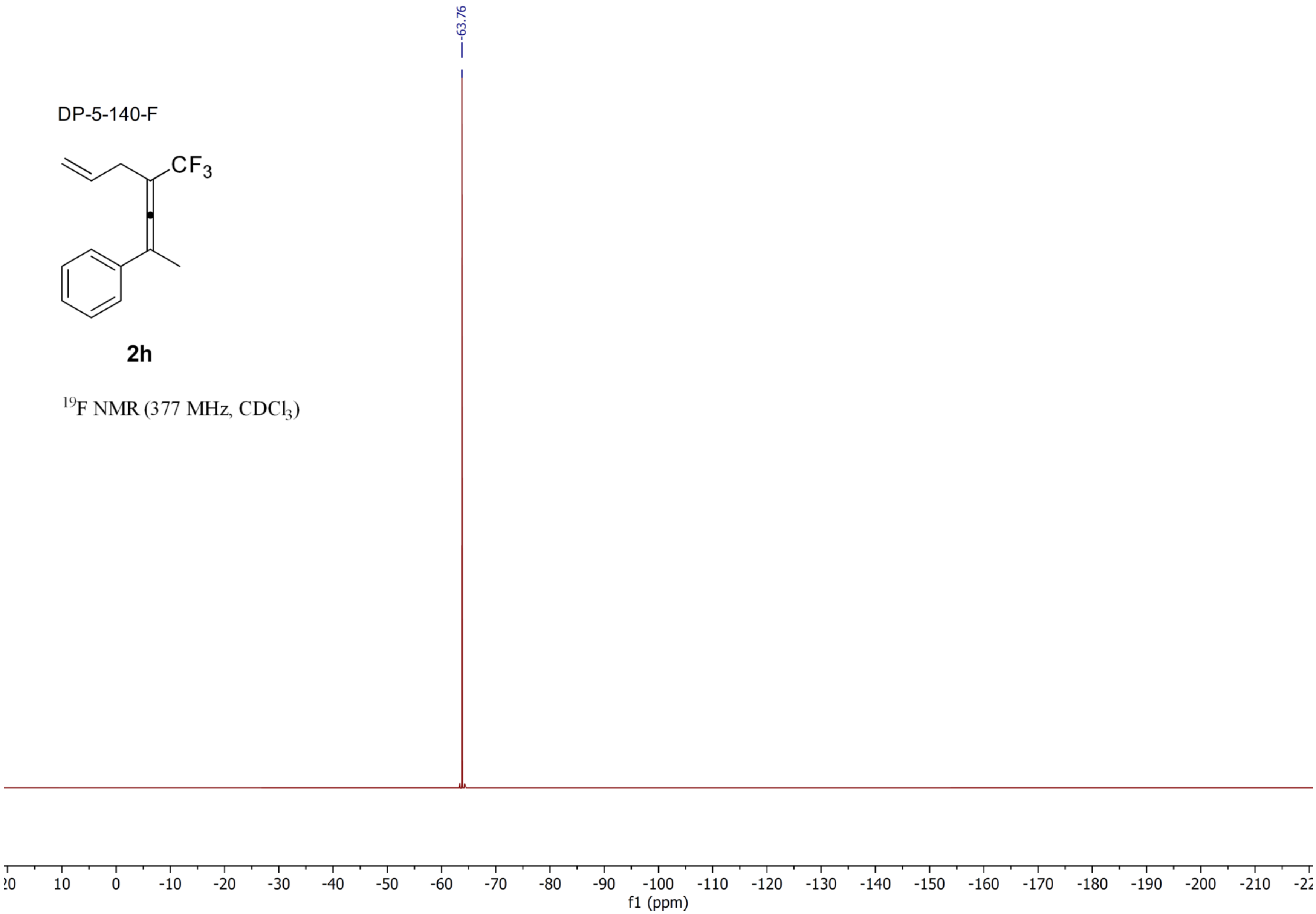

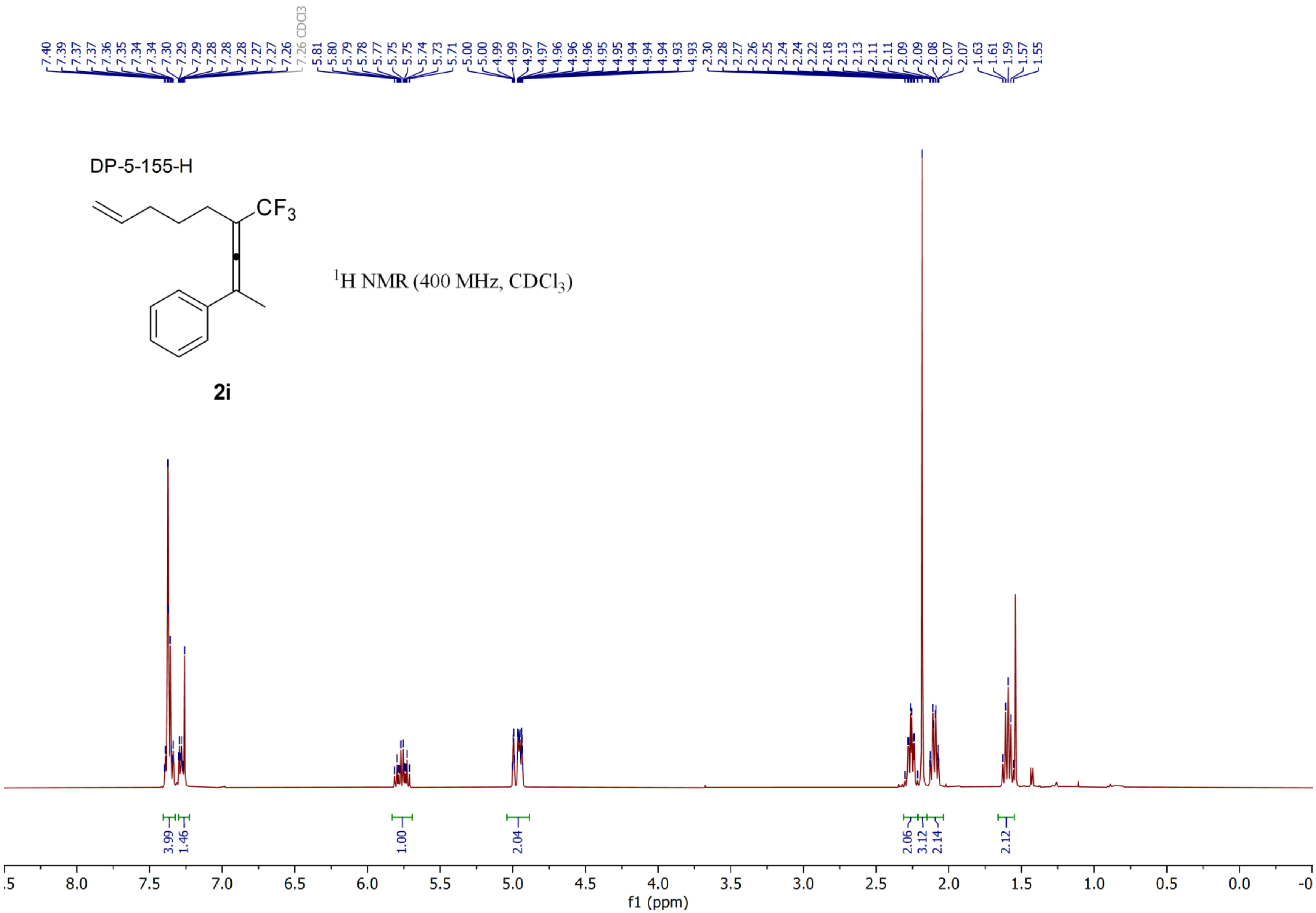
DP-5-155-H
CF3
^{1}H NMR (400 MHz, CDCl$_3$)
7.40 7.39 7.37 7.37 7.36 7.35 7.34 7.34 7.30 7.29 7.29 7.28 7.28 7.28 7.27 7.27 7.26 7.26 CDCl3 5.81 5.80 5.79 5.78 5.77 5.75 5.75 5.74 5.73 5.71 5.00 5.00 4.99 4.99 4.97 4.97 4.96 4.96 4.96 4.95 4.95 4.94 4.94 4.94 4.94 4.93 4.93 2.30 2.28 2.27 2.26 2.25 2.24 2.24 2.22 2.18 2.13 2.13 2.11 2.11 2.09 2.09 2.08 2.07 2.07 1.63 1.61 1.59 1.57 1.55
3.99 1.46 1.00 2.04 2.06 3.12 2.14 2.12
f1 (ppm)

2i

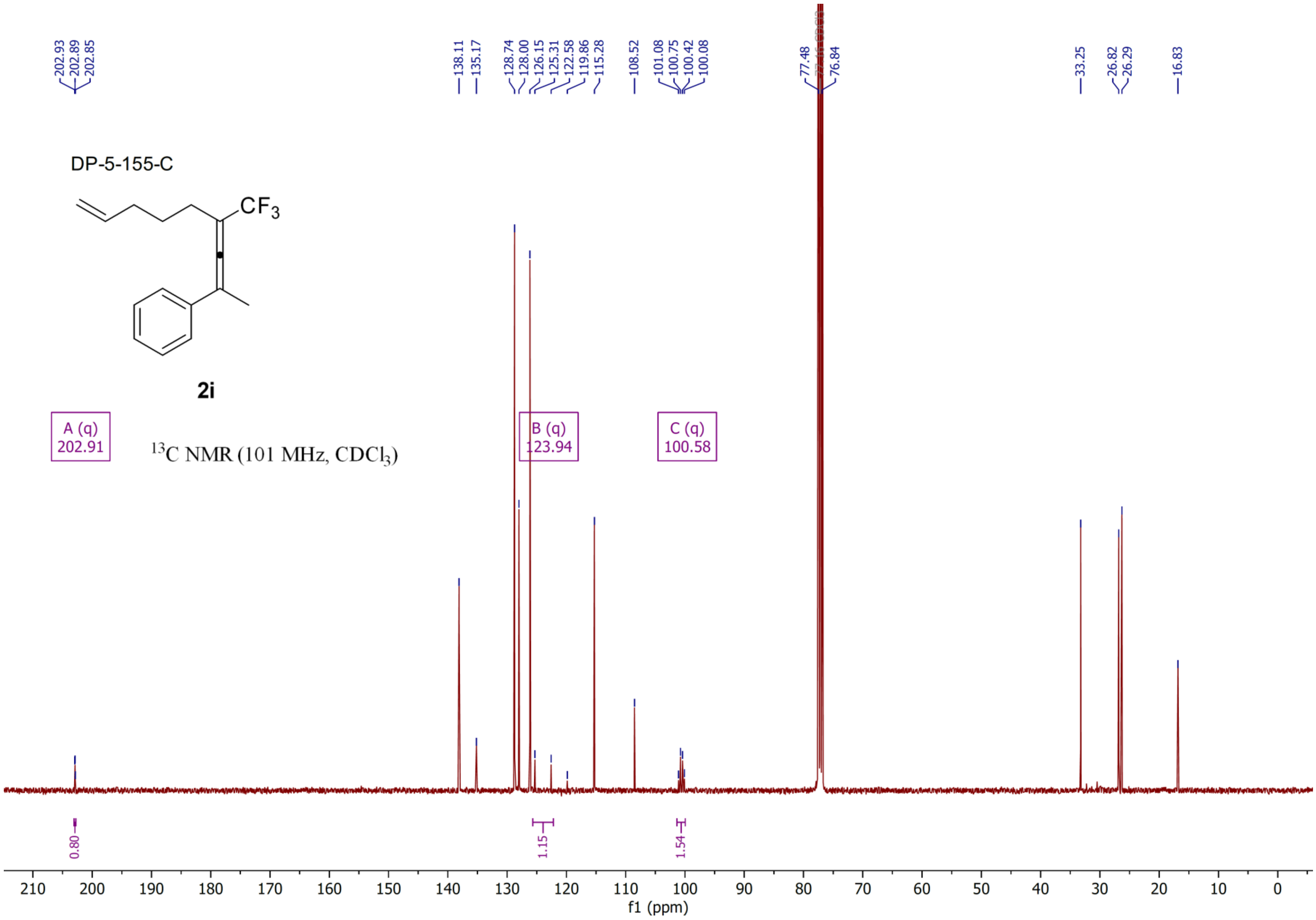
202.93
202.89
202.85
138.11
135.17
128.74
128.00
126.15
125.31
122.58
119.86
115.28
108.52
101.08
100.75
100.42
100.08
77.48
76.84
33.25
26.82
26.29
16.83
DP-5-155-C
CF3
2i
A (q)
202.91
B (q)
123.94
C (q)
100.58
13C NMR (101 MHz, CDCl3)
0.80
1.15
1.54
210 200 190 180 170 160 150 140 130 120 110 100 90 80 70 60 50 40 30 20 10 0
f1 (ppm)

DP-5-155-F

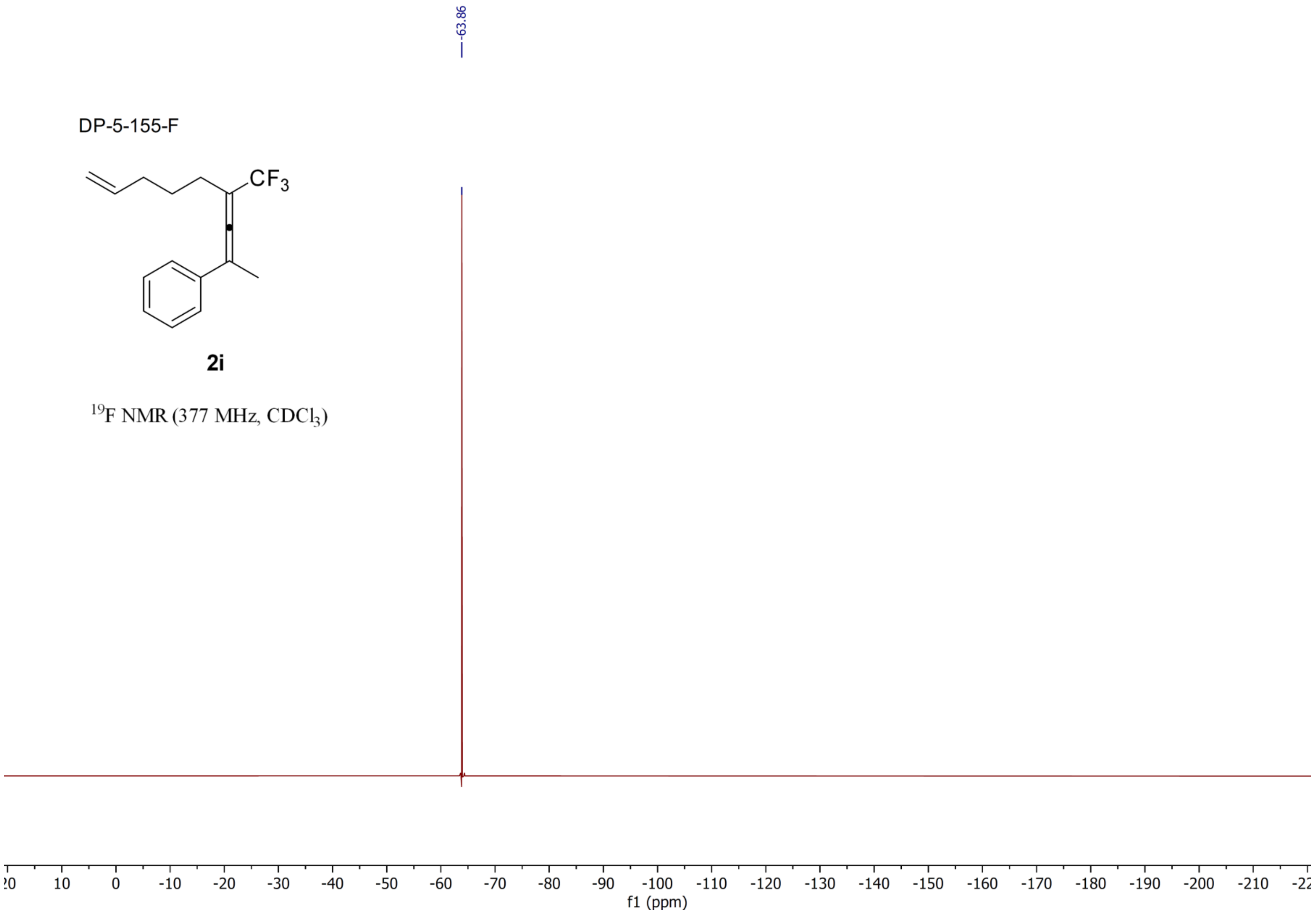

2i

^{19}F NMR (377 MHz, $CDCl_3$)

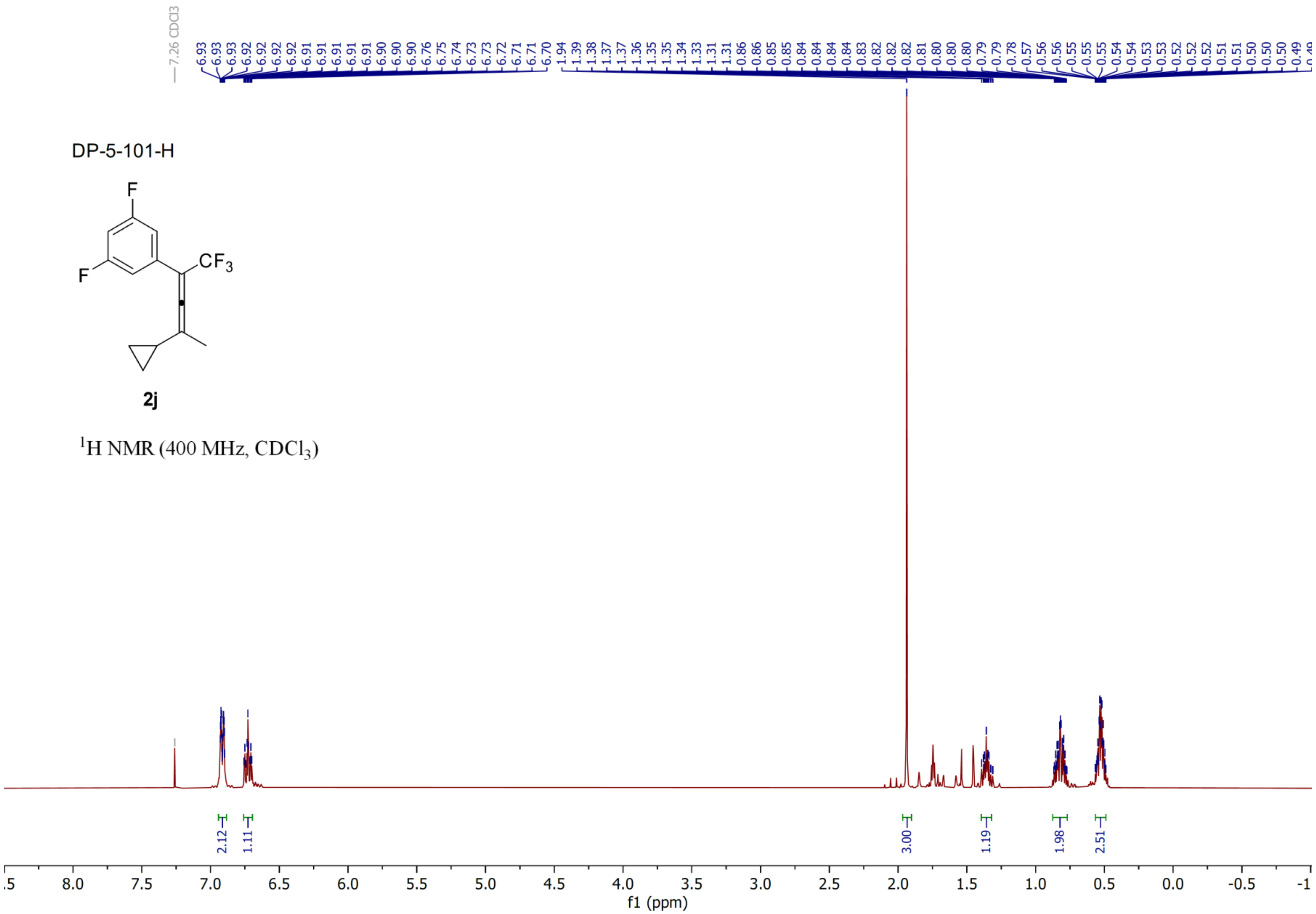
7.26 CDCl3
6.93 6.93 6.93 6.92 6.92 6.92 6.91 6.91 6.91 6.91 6.91 6.90 6.90 6.90 6.76 6.75 6.74 6.73 6.73 6.72 6.71 6.71 6.70
1.94 1.39 1.38 1.37 1.37 1.36 1.35 1.35 1.34 1.33 1.31 1.31 0.86 0.86 0.85 0.85 0.84 0.84 0.84 0.84 0.83 0.82 0.82 0.82 0.81 0.80 0.80 0.80 0.79 0.79 0.78 0.57 0.56 0.56 0.55 0.55 0.55 0.54 0.54 0.53 0.53 0.52 0.52 0.52 0.51 0.51 0.50 0.50 0.50 0.49
DP-5-101-H
F
F
CF3
2j
^{1}H NMR (400 MHz, CDCl$_3$)
2.12
1.11
3.00
1.19
1.98
2.51
.5 8.0 7.5 7.0 6.5 6.0 5.5 5.0 4.5 4.0 3.5 3.0 2.5 2.0 1.5 1.0 0.5 0.0 -0.5 -1
f1 (ppm)

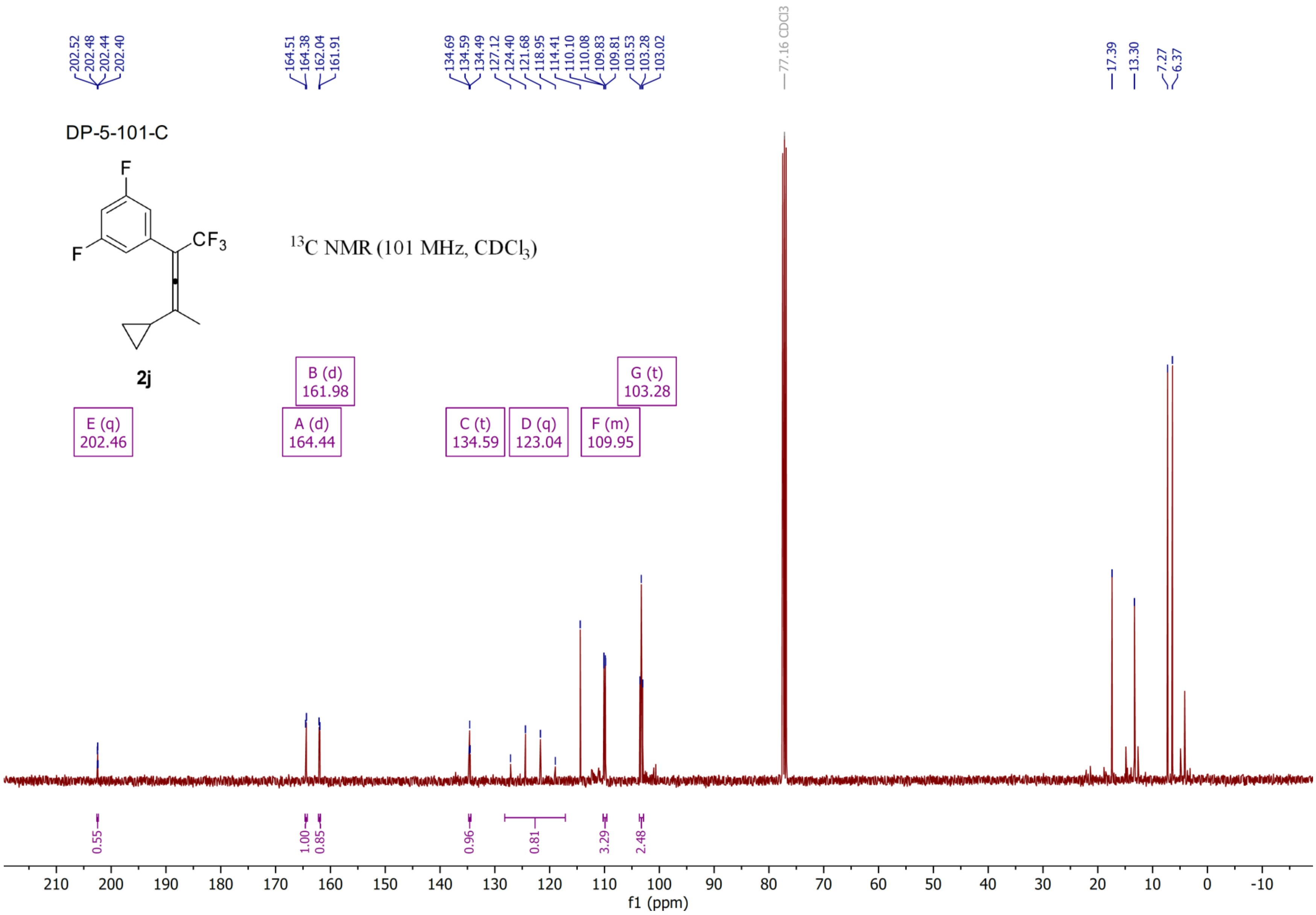

202.52
202.48
202.44
202.40
164.51
164.38
162.04
161.91
134.69
134.59
134.49
127.12
124.40
121.68
118.95
114.41
110.10
110.08
109.83
109.81
103.53
103.28
103.02
77.16 CDCl3
17.39
13.30
7.27
6.37
DP-5-101-C
F
F
CF3
2j
^{13}C NMR (101 MHz, CDCl$_3$)
B (d) 161.98
G (t) 103.28
E (q) 202.46
A (d) 164.44
C (t) 134.59
D (q) 123.04
F (m) 109.95
0.55
1.00
0.85
0.96
0.81
3.29
2.48
210 200 190 180 170 160 150 140 130 120 110 100 90 80 70 60 50 40 30 20 10 0 -10
f1 (ppm)

DP-5-101-F

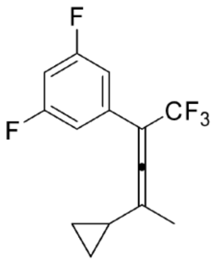

2j

^{19}F NMR (377 MHz, $CDCl_3$)

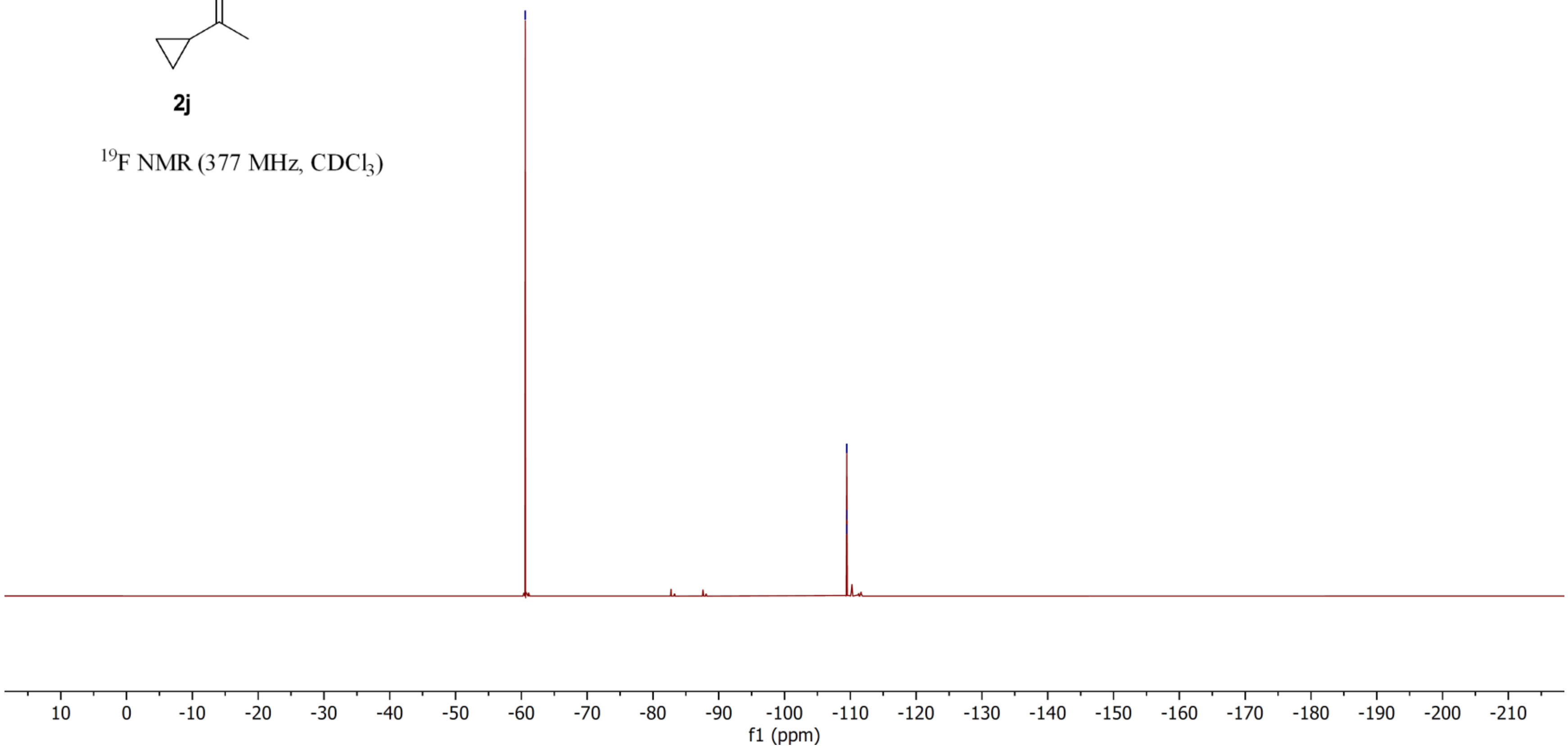

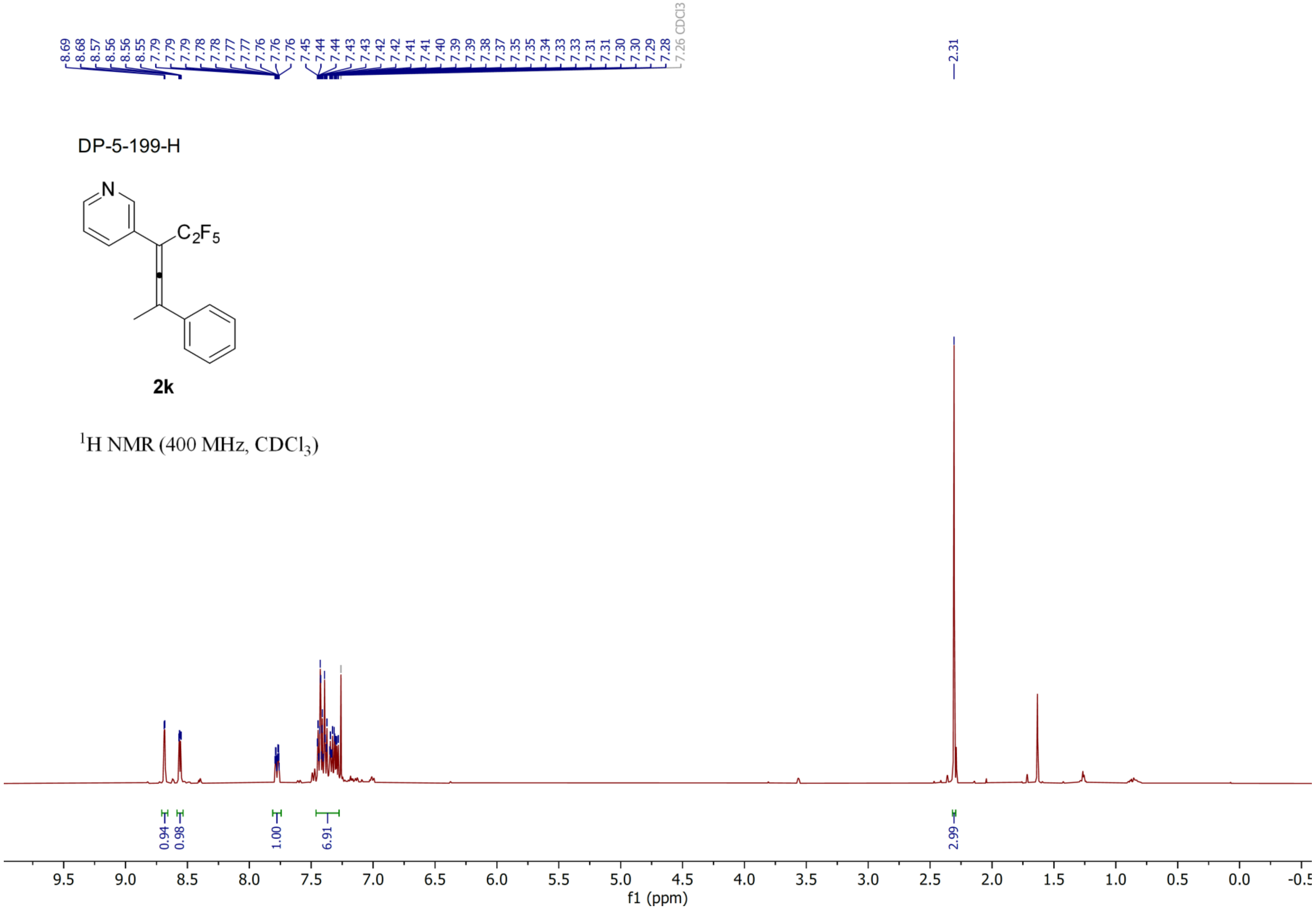
8.69 8.68 8.57 8.56 8.56 8.55 7.79 7.79 7.79 7.78 7.78 7.77 7.77 7.76 7.76 7.76 7.45 7.44 7.44 7.43 7.43 7.42 7.42 7.41 7.41 7.40 7.39 7.39 7.38 7.37 7.35 7.35 7.34 7.33 7.33 7.31 7.31 7.30 7.30 7.29 7.28 7.26 CDCl3
2.31
DP-5-199-H
N
C_2F_5
2k
^{1}H NMR (400 MHz, $CDCl_3$)
0.94 0.98 1.00 6.91 2.99
9.5 9.0 8.5 8.0 7.5 7.0 6.5 6.0 5.5 5.0 4.5 4.0 3.5 3.0 2.5 2.0 1.5 1.0 0.5 0.0
f1 (ppm)

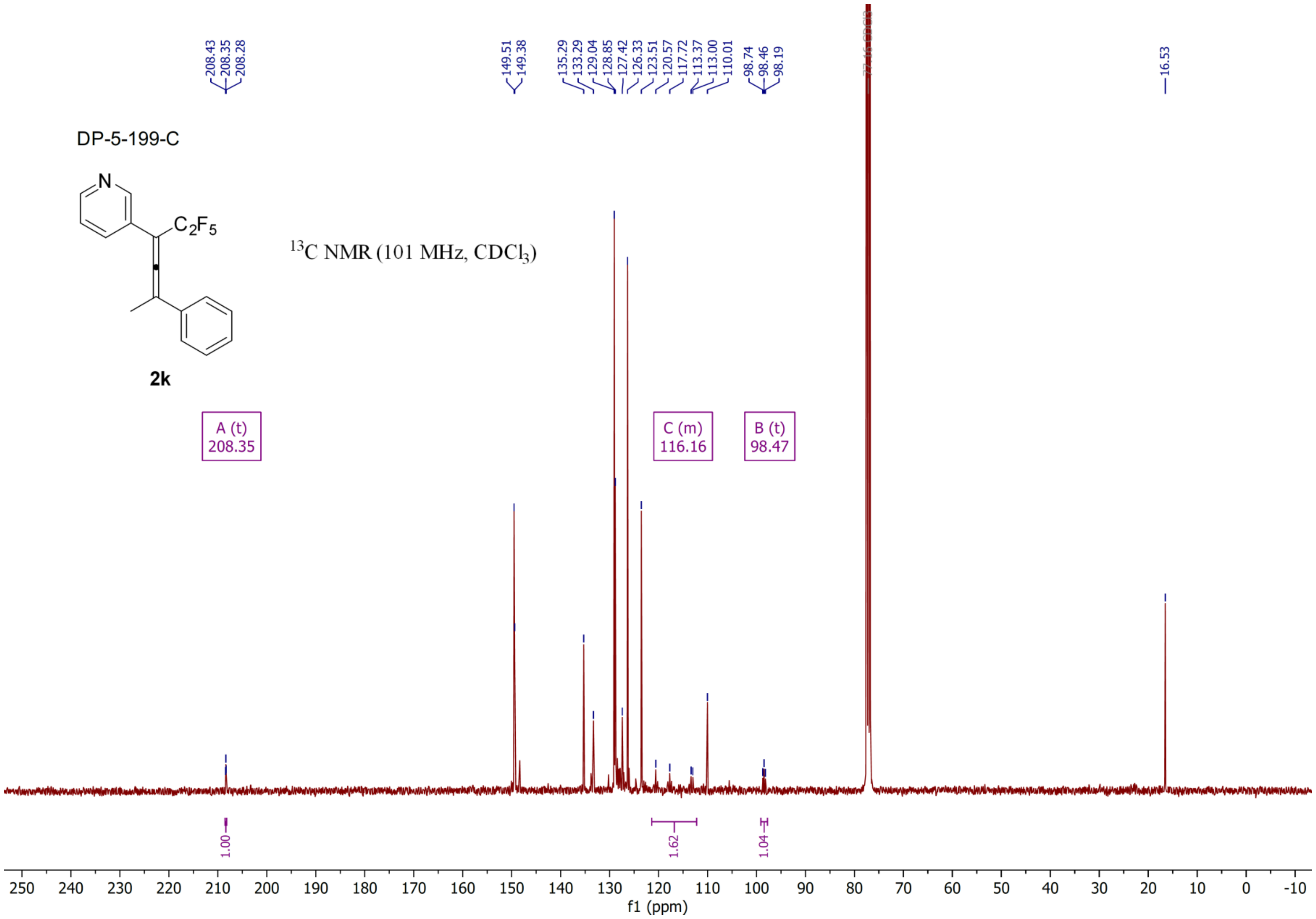

208.43
208.35
208.28
149.51
149.38
135.29
133.29
129.04
128.85
127.42
126.33
123.51
120.57
117.72
113.37
113.00
110.01
98.74
98.46
98.19
16.53
DP-5-199-C
N
C_2F_5
2k
^{13}C NMR (101 MHz, CDCl$_3$)
A (t)
208.35
C (m)
116.16
B (t)
98.47
1.00
1.62
1.04
f1 (ppm)

DP-5-199-F

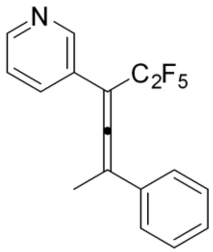

2k

^{19}F NMR (377 MHz, $CDCl_3$)

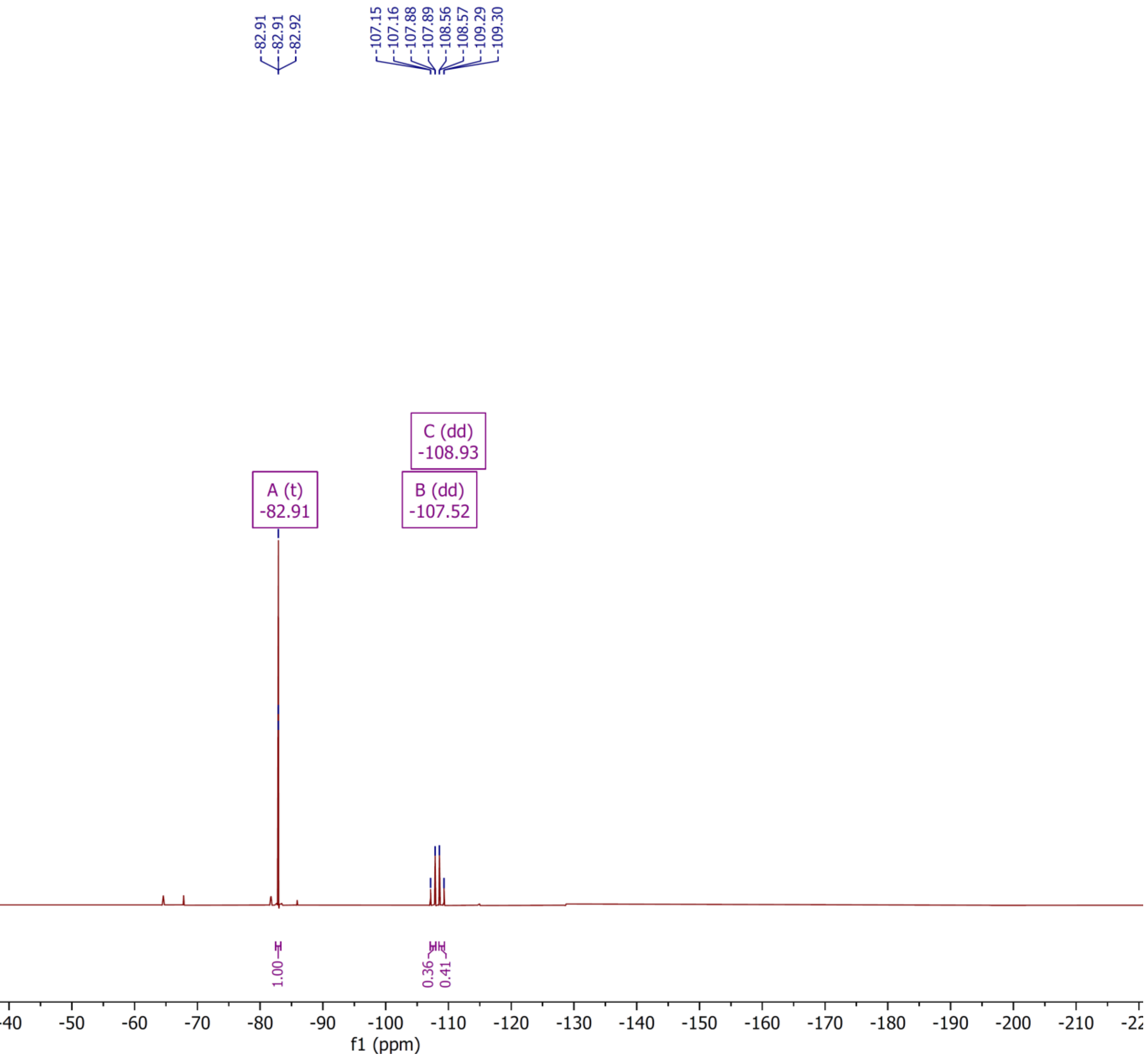

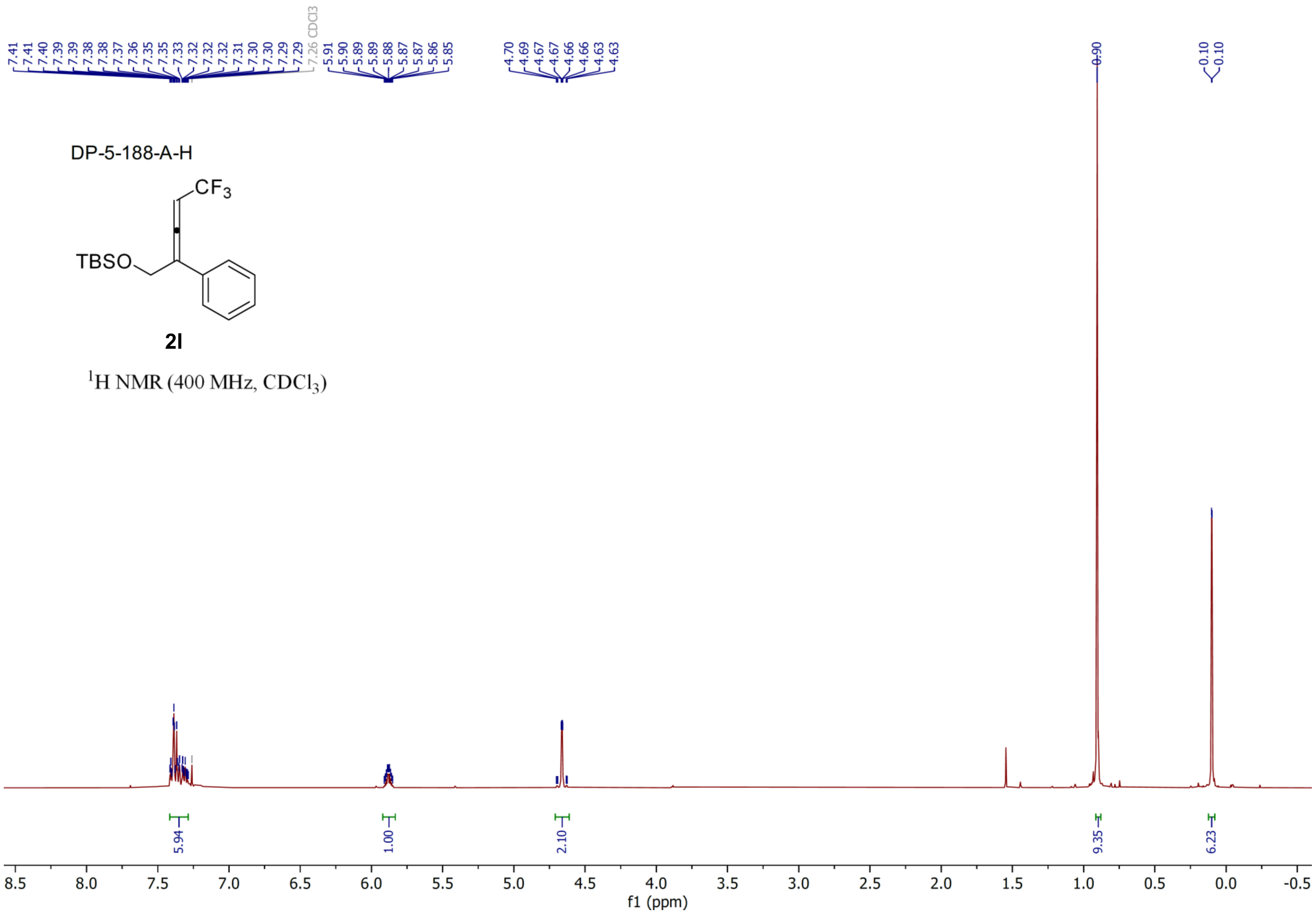
7.41
7.41
7.40
7.39
7.39
7.38
7.38
7.37
7.36
7.35
7.35
7.33
7.32
7.32
7.32
7.31
7.30
7.30
7.29
7.29
7.26 CDCl3
5.91
5.90
5.89
5.89
5.88
5.87
5.87
5.86
5.85
4.70
4.69
4.67
4.67
4.66
4.66
4.63
4.63
0.90
0.10
0.10
DP-5-188-A-H
CF3
TBSO
2l
^{1}H NMR (400 MHz, CDCl$_3$)
5.94
1.00
2.10
9.35
6.23
8.5
8.0
7.5
7.0
6.5
6.0
5.5
5.0
4.5
4.0
3.5
3.0
2.5
2.0
1.5
1.0
0.5
0.0
-0.5
f1 (ppm)

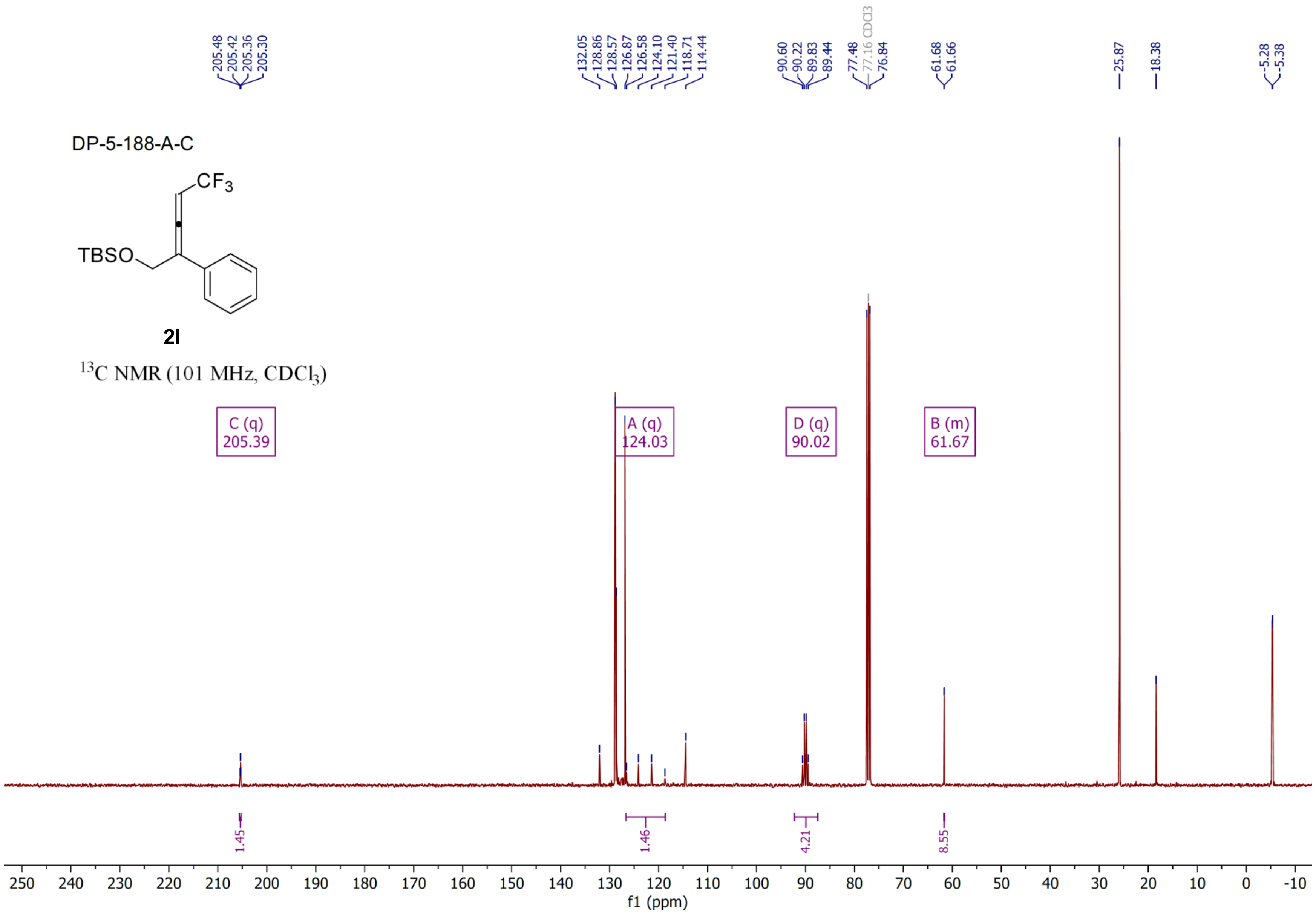

205.48
205.42
205.36
205.30
132.05
128.86
128.57
126.87
126.58
124.10
121.40
118.71
114.44
90.60
90.22
89.83
89.44
77.48
77.16 CDCl3
76.84
61.68
61.66
25.87
18.38
-5.28
-5.38
DP-5-188-A-C
CF3
TBSO
2l
13C NMR (101 MHz, CDCl3)
C (q)
205.39
A (q)
124.03
D (q)
90.02
B (m)
61.67
1.45
1.46
4.21
8.55
250 240 230 220 210 200 190 180 170 160 150 140 130 120 110 100 90 80 70 60 50 40 30 20 10 0 -10
f1 (ppm)

DP-5-188-A-F

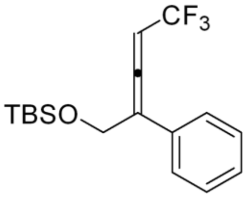

2l

^{19}F NMR (377 MHz, $CDCl_3$)

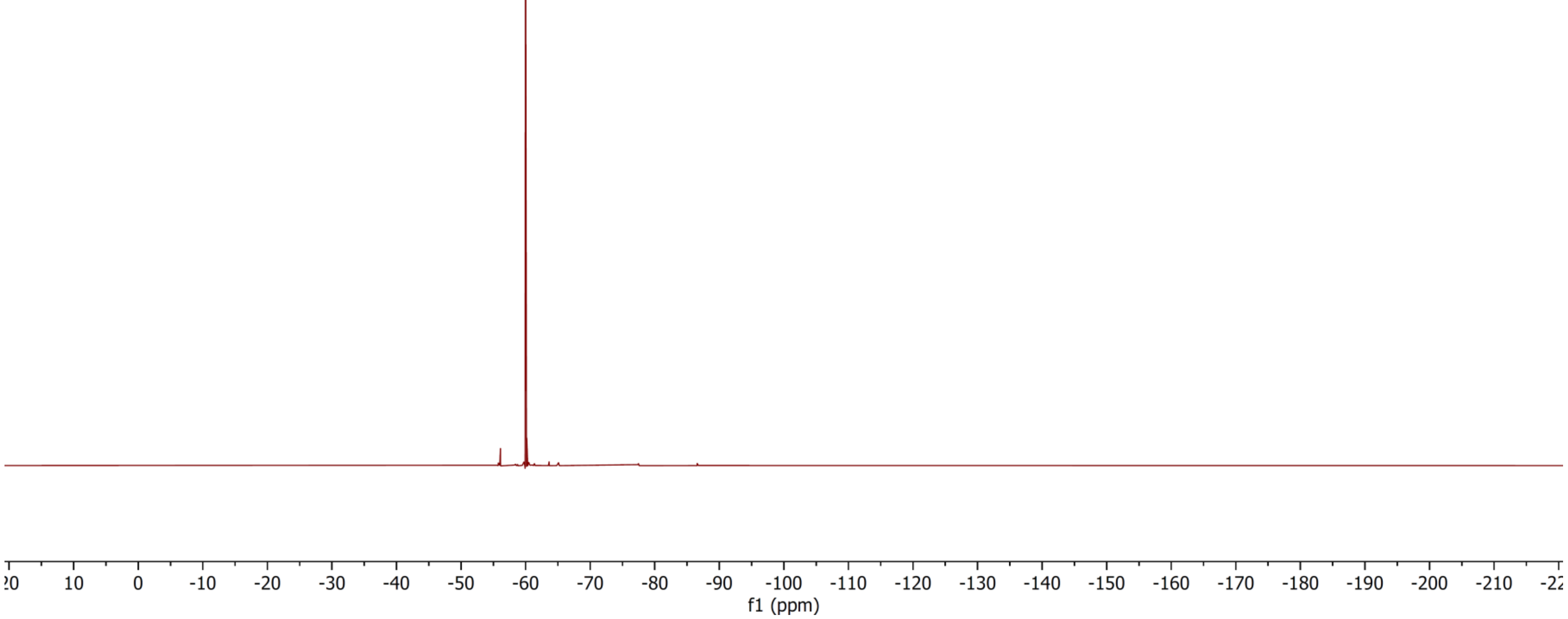

DP-5-149-H

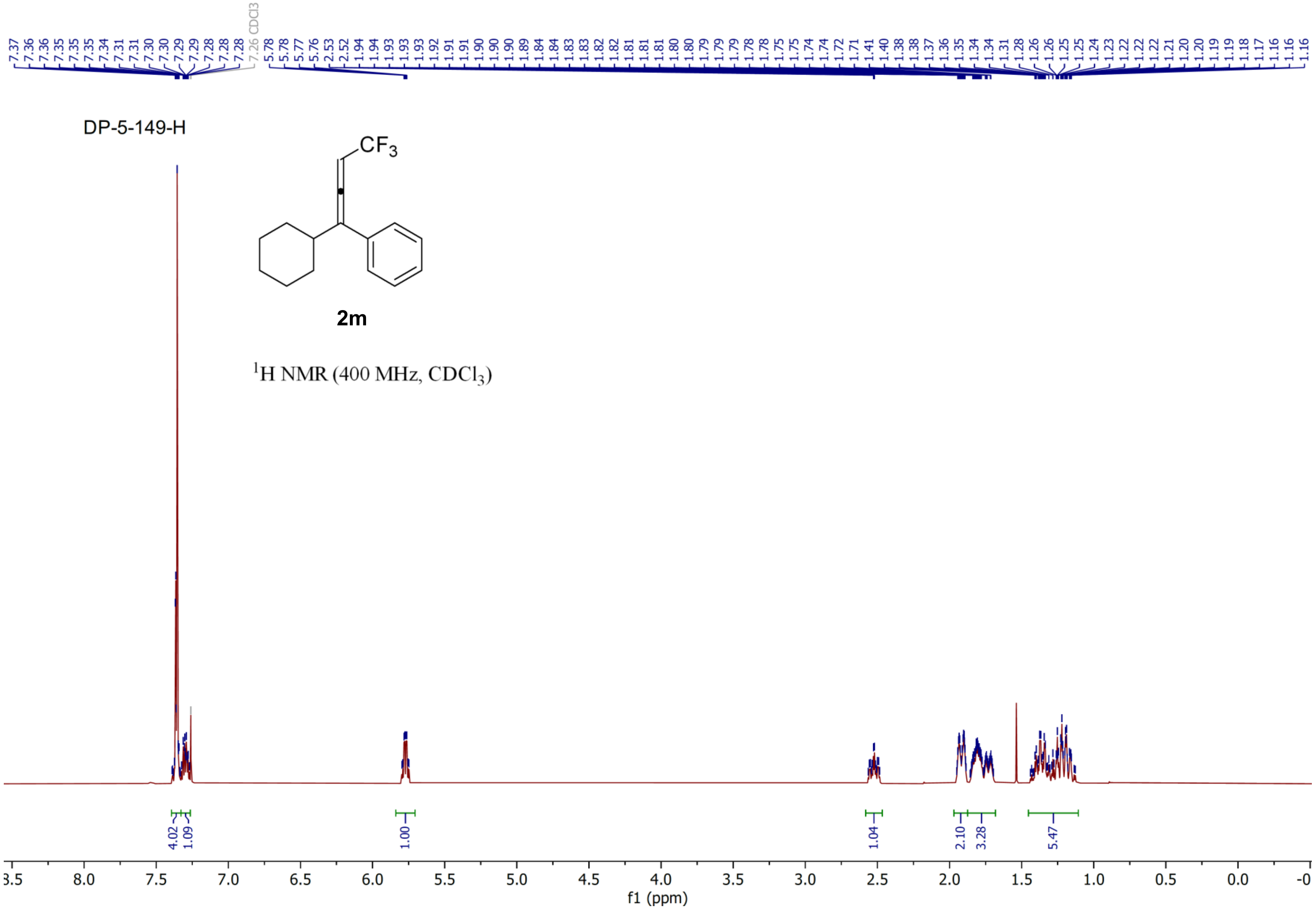

2m

^{1}H NMR (400 MHz, $CDCl_3$)

DP-5-149-C

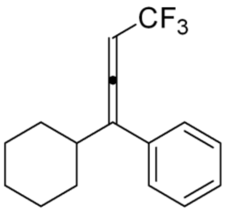

2m

^{13}C NMR (101 MHz, $CDCl_3$)

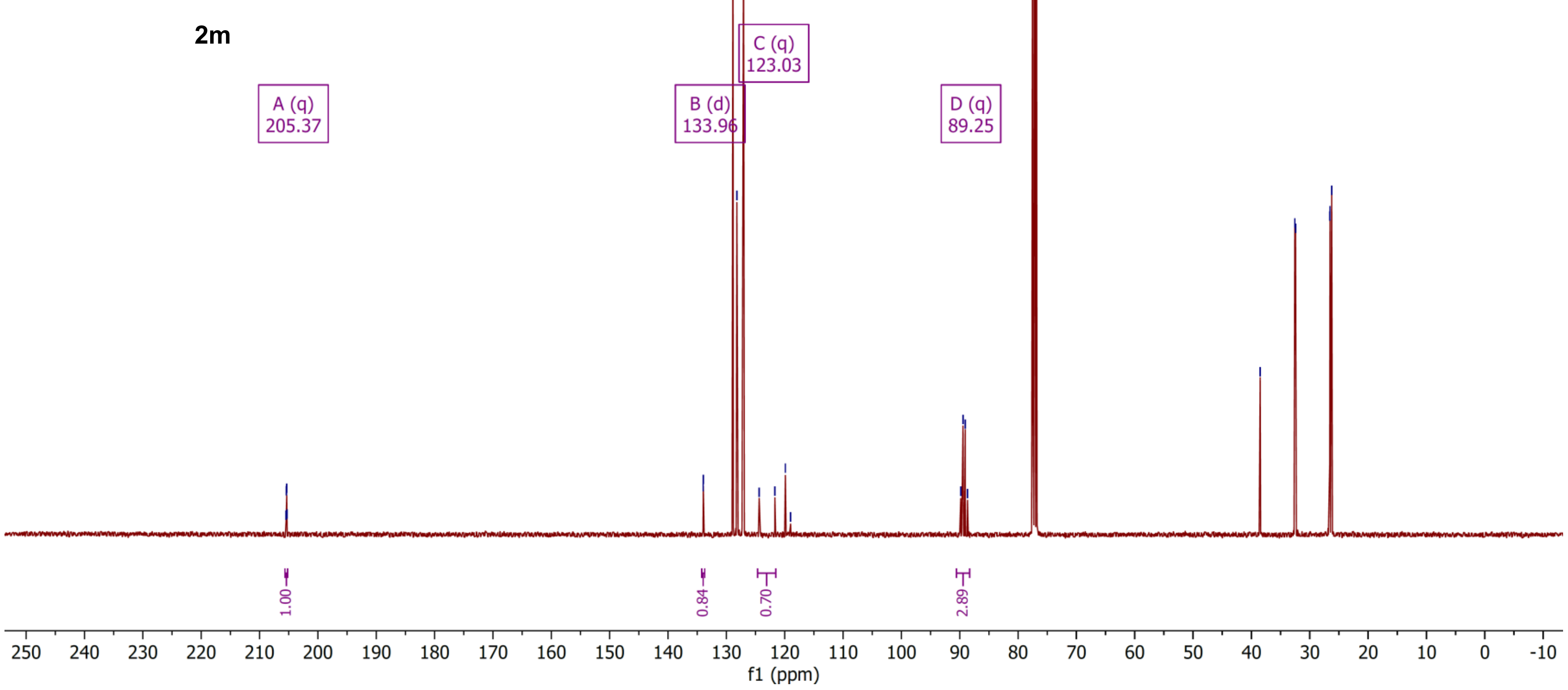

DP-5-149-F

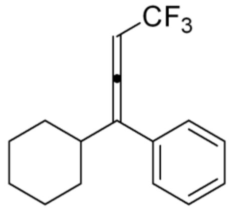

2m

^{19}F NMR (377 MHz, $CDCl_3$)

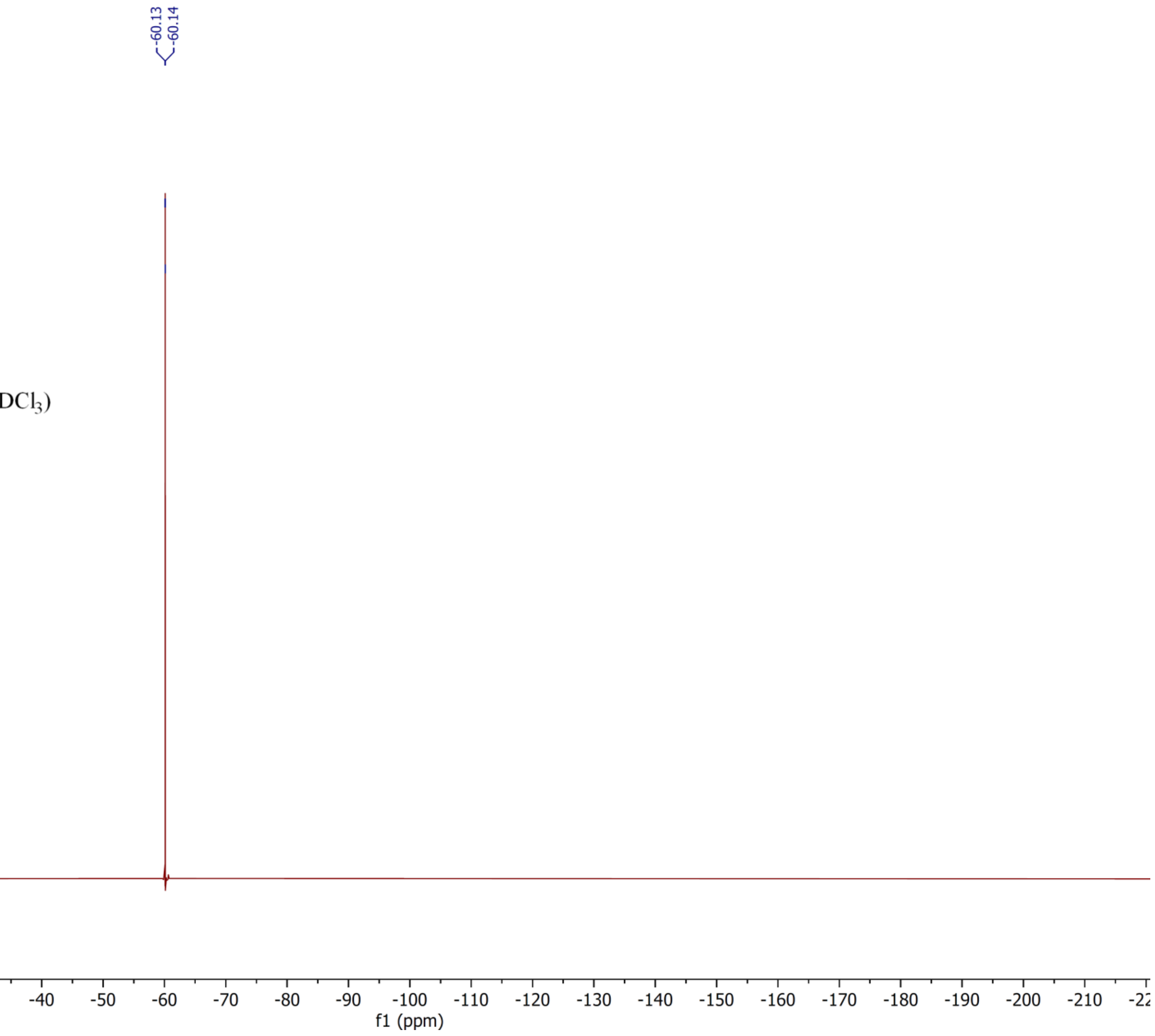

DP-5-179-H

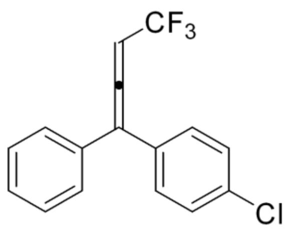

2n

^{1}H NMR (400 MHz, $CDCl_3$)

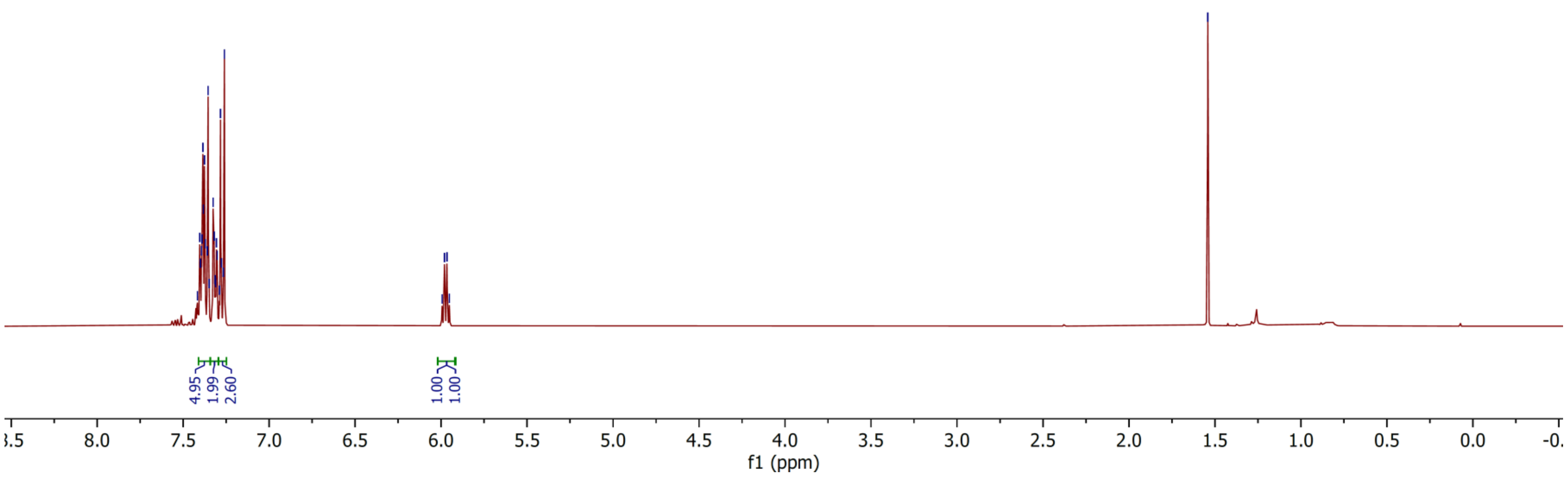

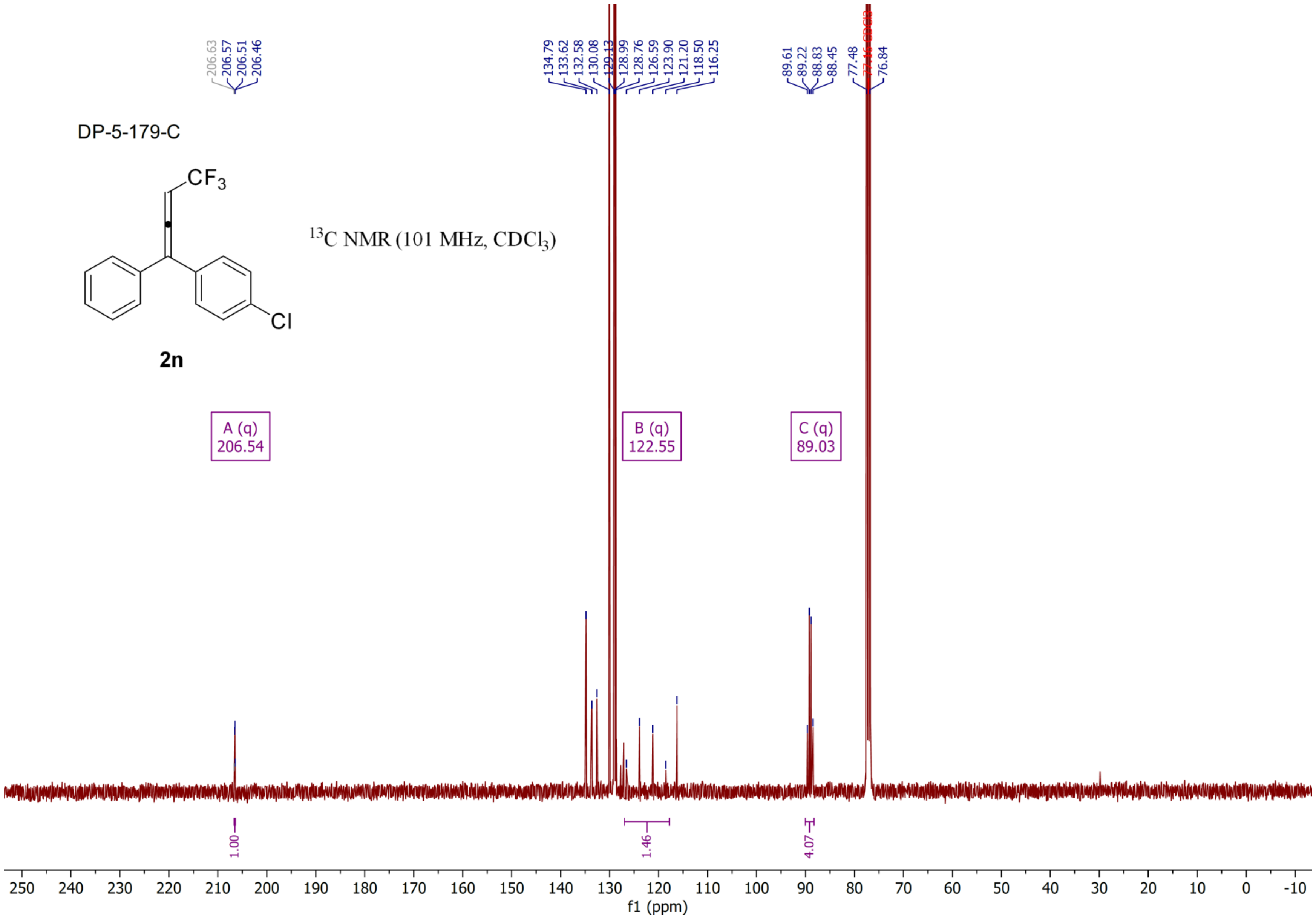
DP-5-179-C
CF3
Cl
2n
13C NMR (101 MHz, CDCl3)
206.63
206.57
206.51
206.46
134.79
133.62
132.58
130.08
129.13
128.99
128.76
126.59
123.90
121.20
118.50
116.25
89.61
89.22
88.83
88.45
77.48
77.16 CDCl3
76.84
A (q)
206.54
B (q)
122.55
C (q)
89.03
1.00
1.46
4.07
250
240
230
220
210
200
190
180
170
160
150
140
130
120
110
100
90
80
70
60
50
40
30
20
10
0
-10
f1 (ppm)

DP-5-179-F

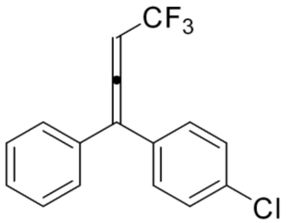

2n

^{19}F NMR (377 MHz, CDCl$_3$)

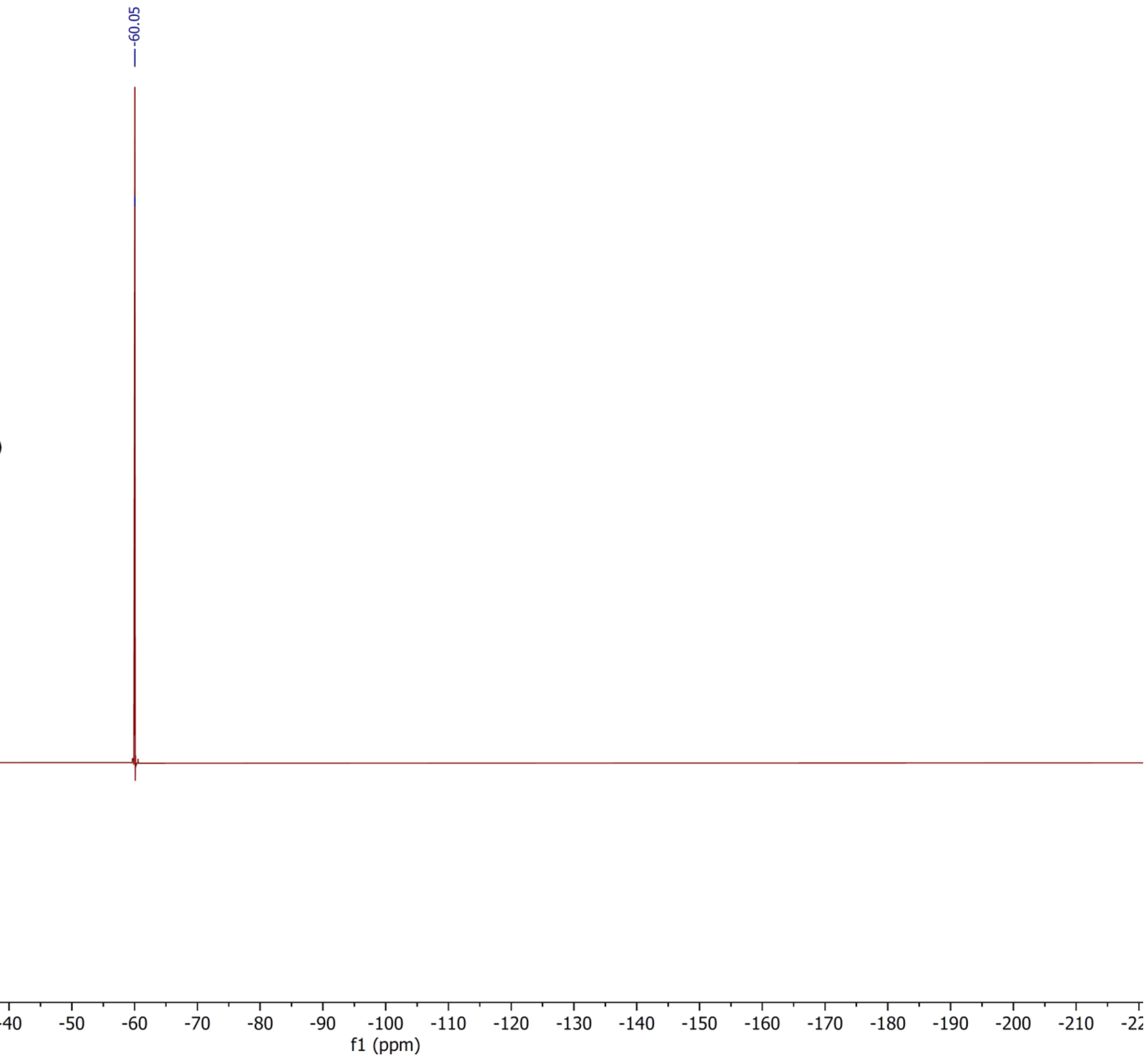

DP-6-47-H-500mhz

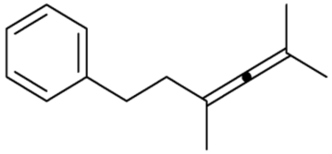

2oa

^{1}H NMR (500 MHz, $CDCl_3$)

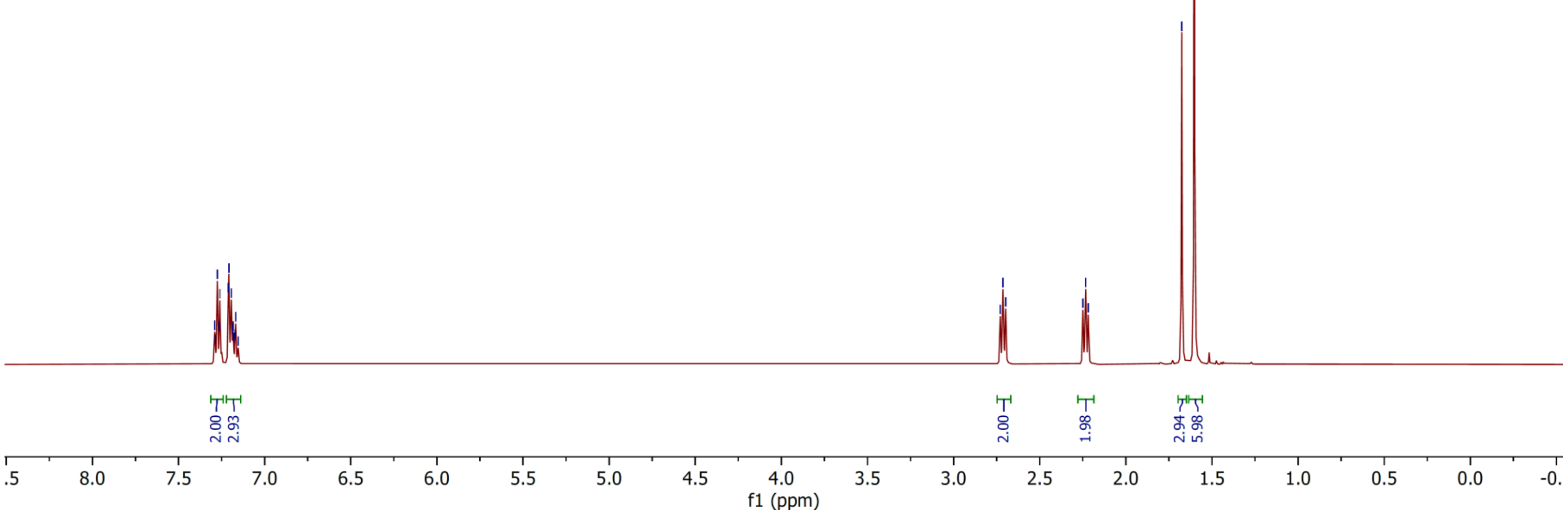

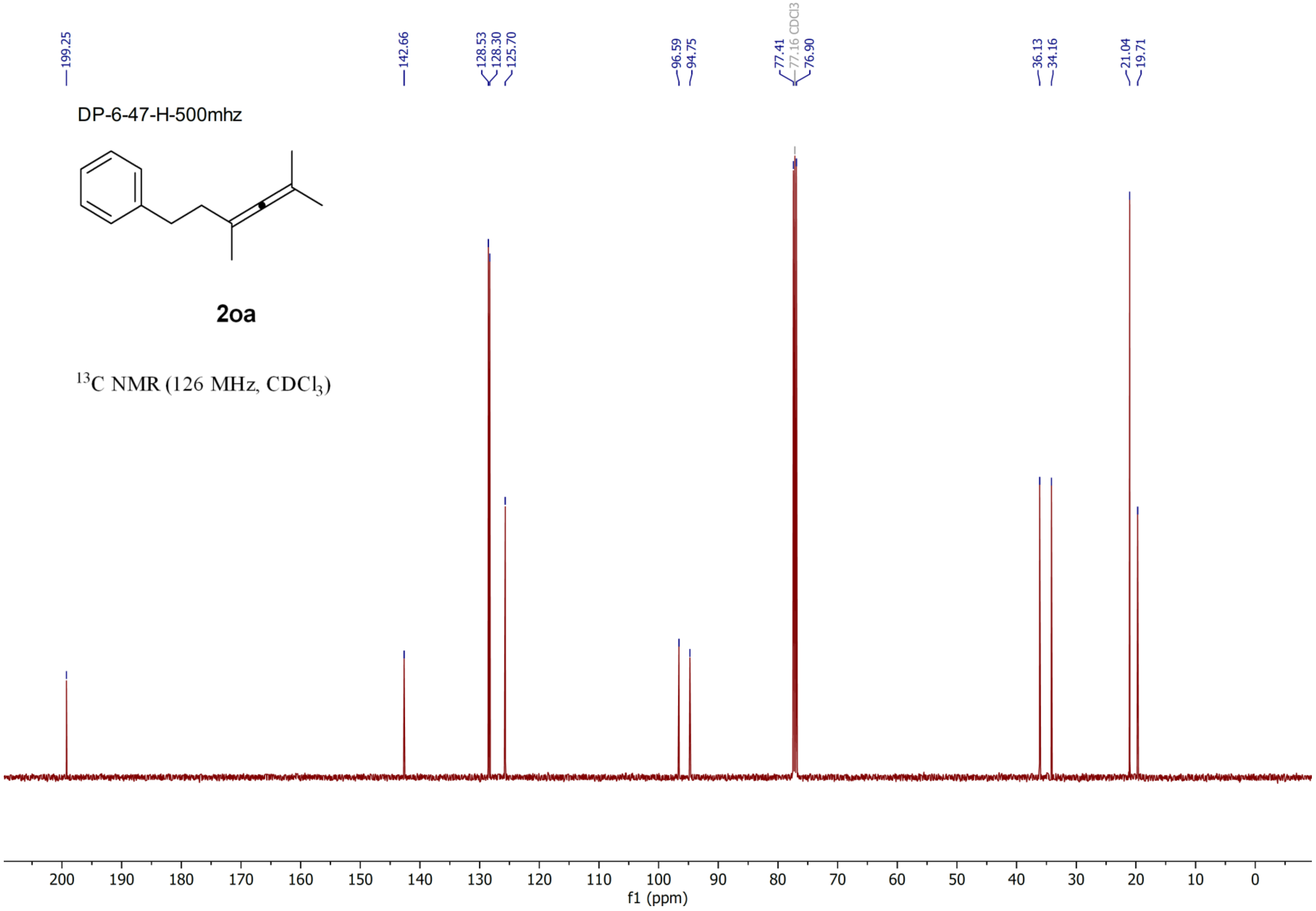
199.25
142.66
128.53
128.30
125.70
96.59
94.75
77.41
77.16 CDCl3
76.90
36.13
34.16
21.04
19.71
DP-6-47-H-500mhz
2oa
13C NMR (126 MHz, CDCl3)
200
190
180
170
160
150
140
130
120
110
100
90
80
70
60
50
40
30
20
10
0
f1 (ppm)

DP-6-69-H

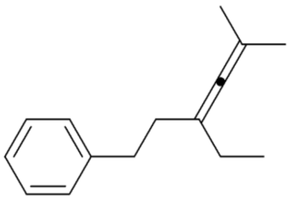

2ob

^{1}H NMR (400 MHz, $CDCl_3$)

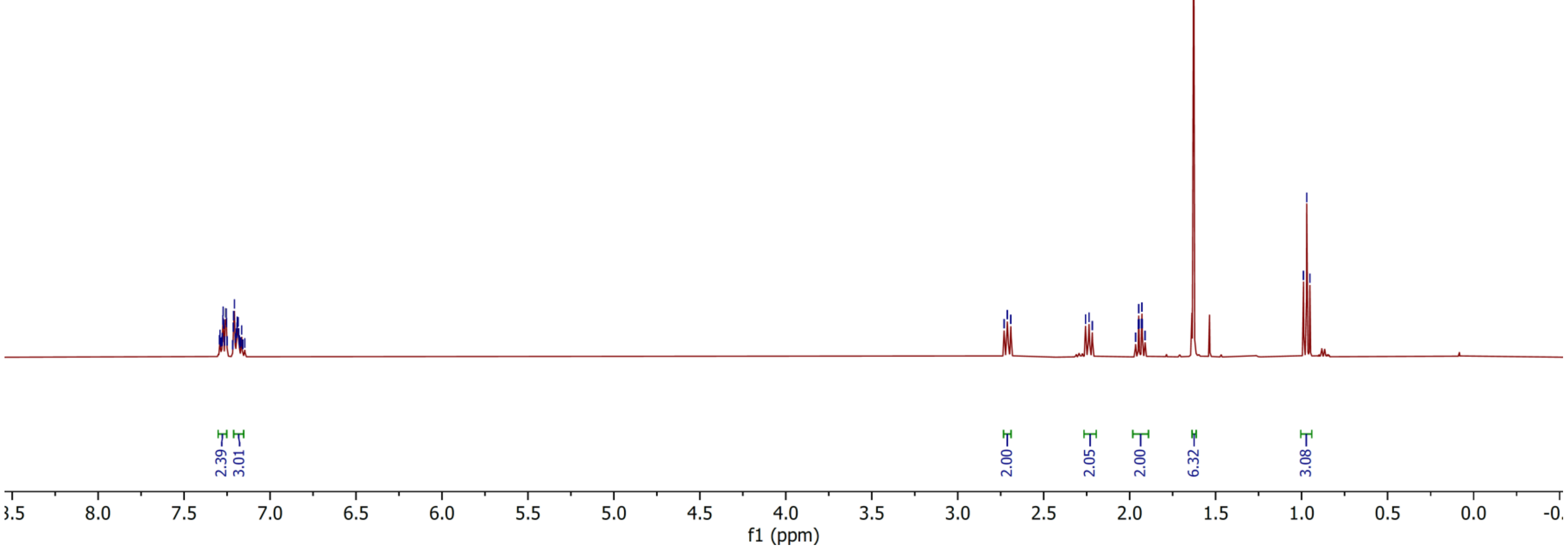

DP-6-69-C

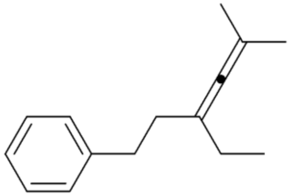

2ob

^{13}C NMR (101 MHz, $CDCl_3$)

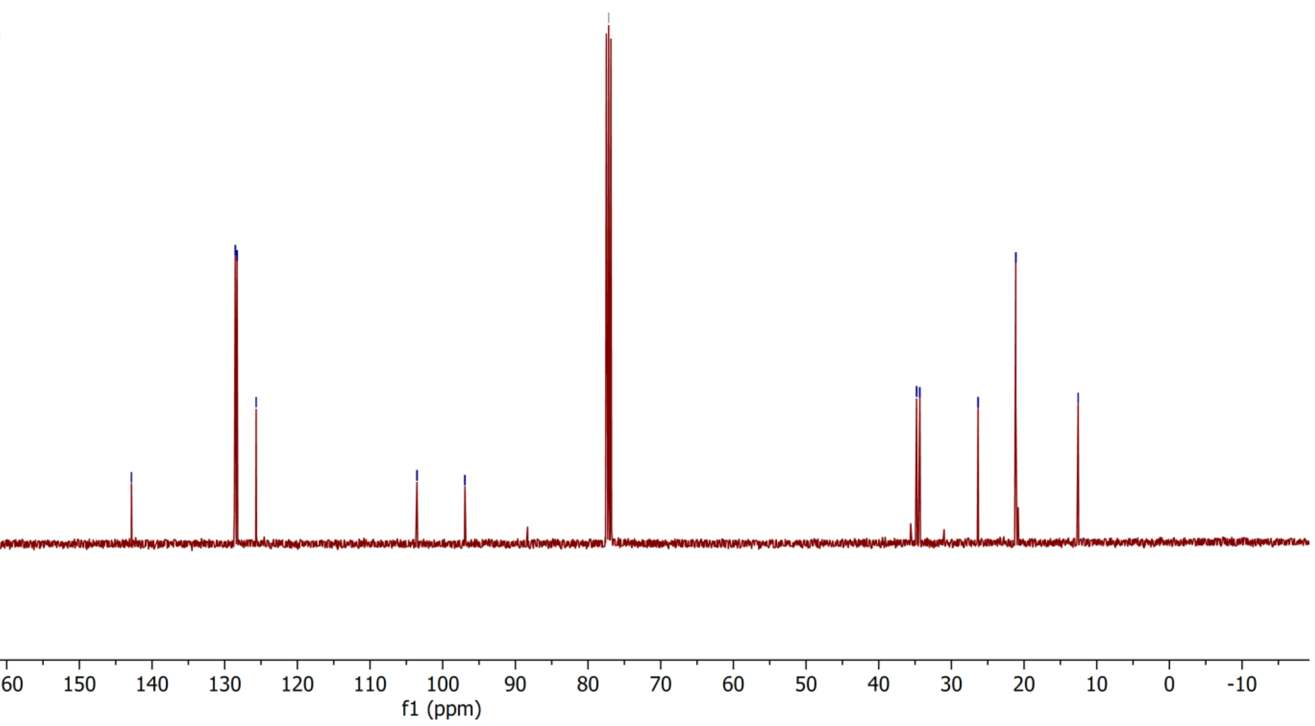

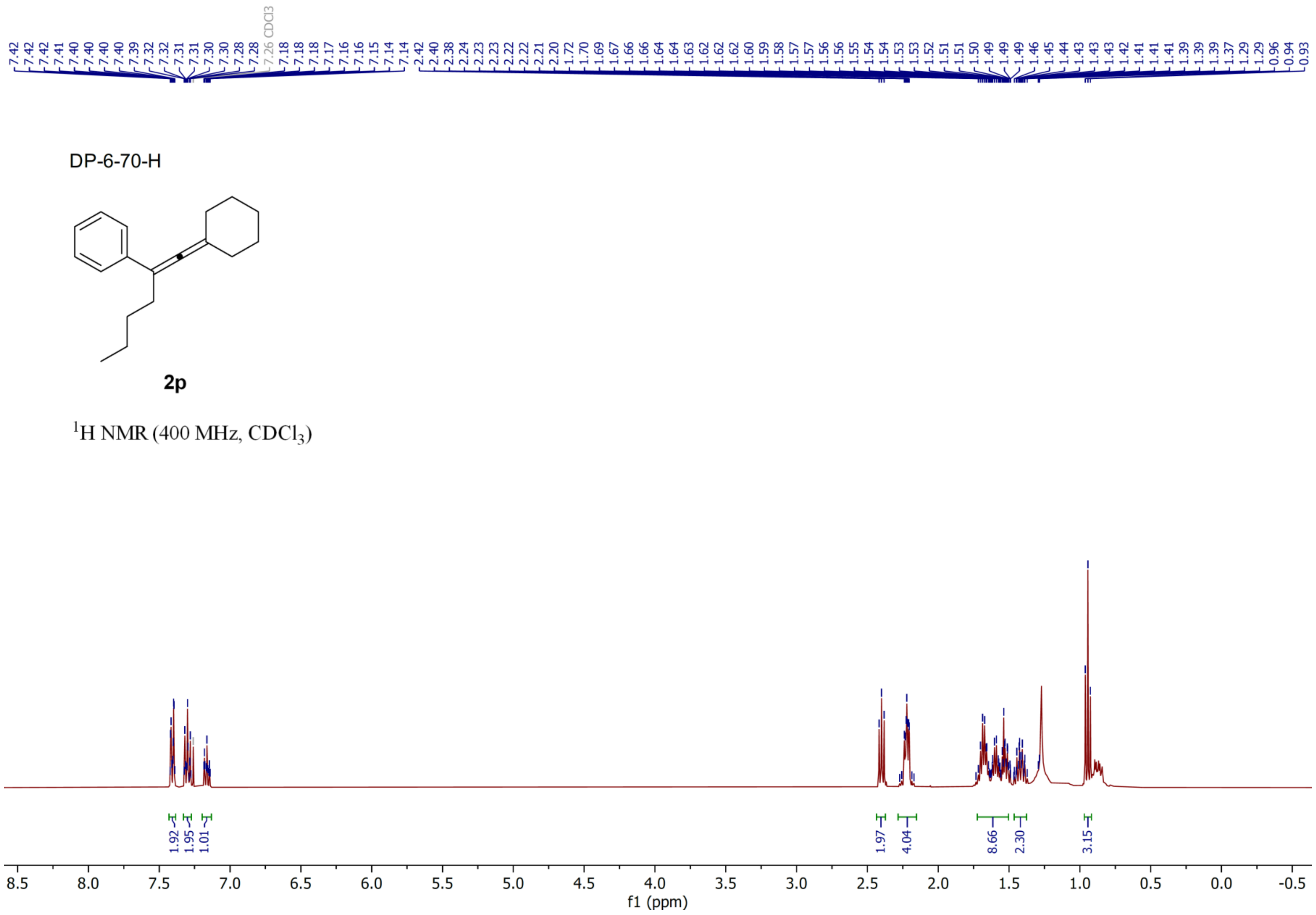
7.42 7.42 7.42 7.41 7.40 7.40 7.40 7.40 7.39 7.32 7.32 7.31 7.31 7.30 7.28 7.28 7.26 CDCl3 7.18 7.18 7.18 7.17 7.16 7.16 7.15 7.14 7.14
2.42 2.40 2.38 2.24 2.23 2.23 2.22 2.22 2.21 2.20 1.72 1.70 1.69 1.67 1.66 1.66 1.64 1.64 1.63 1.62 1.62 1.62 1.60 1.59 1.58 1.57 1.57 1.56 1.56 1.55 1.54 1.54 1.53 1.53 1.52 1.51 1.51 1.50 1.49 1.49 1.49 1.46 1.45 1.44 1.43 1.43 1.43 1.42 1.41 1.41 1.41 1.39 1.39 1.39 1.37 1.29 1.29 0.96 0.94 0.93
DP-6-70-H
2p
^{1}H NMR (400 MHz, $CDCl_3$)
1.92 1.95 1.01 1.97 4.04 8.66 2.30 3.15
8.5 8.0 7.5 7.0 6.5 6.0 5.5 5.0 4.5 4.0 3.5 3.0 2.5 2.0 1.5 1.0 0.5 0.0 -0.5
f1 (ppm)

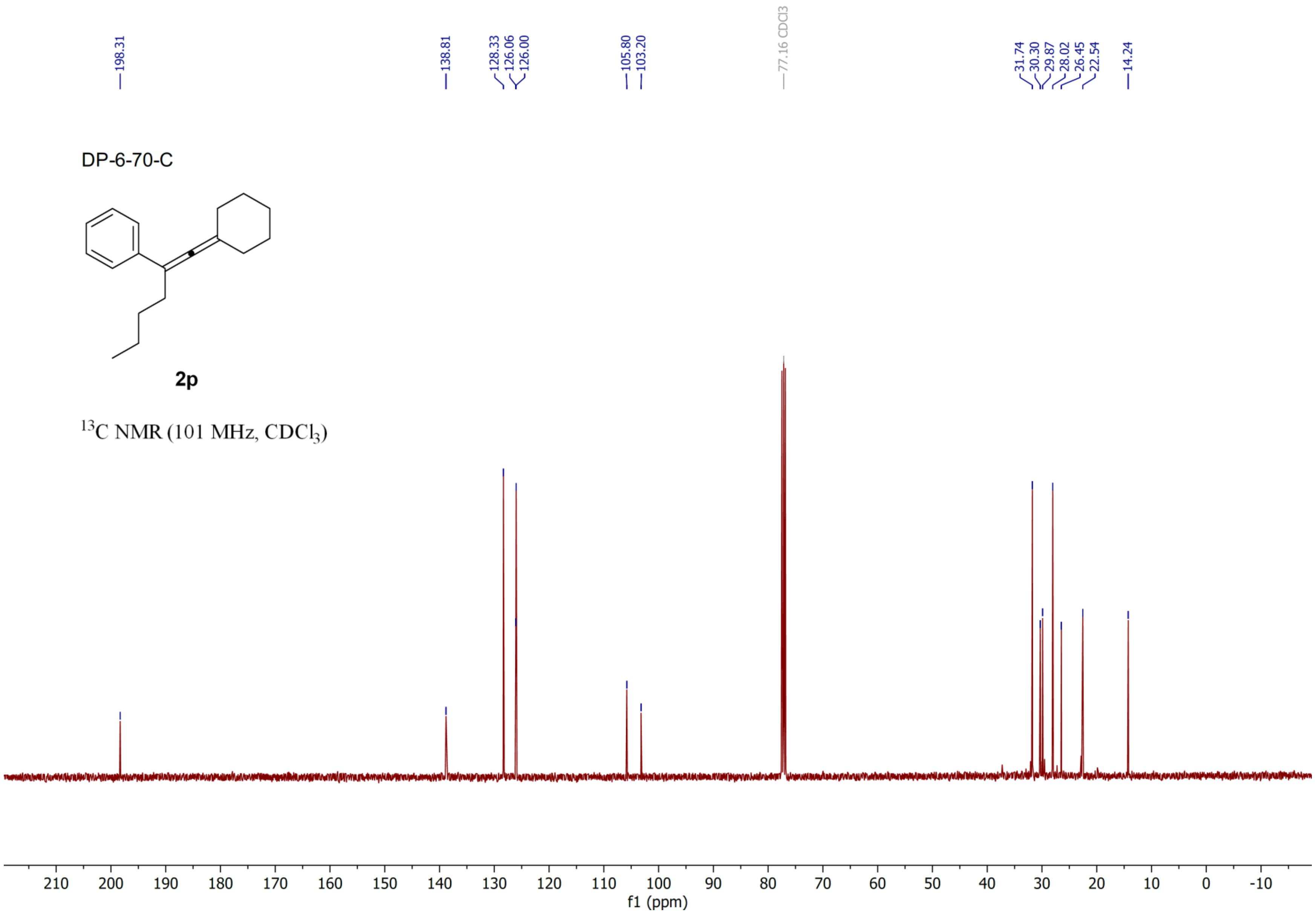
198.31
138.81
128.33
126.06
126.00
105.80
103.20
77.16 CDCl3
31.74
30.30
29.87
28.02
26.45
22.54
14.24
DP-6-70-C
2p
^{13}C NMR (101 MHz, $CDCl_3$)
210 200 190 180 170 160 150 140 130 120 110 100 90 80 70 60 50 40 30 20 10 0 -10
f1 (ppm)

DP-6-71-H-500mhz

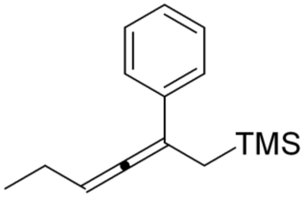

2q

^1H NMR (500 MHz, $CDCl_3$)

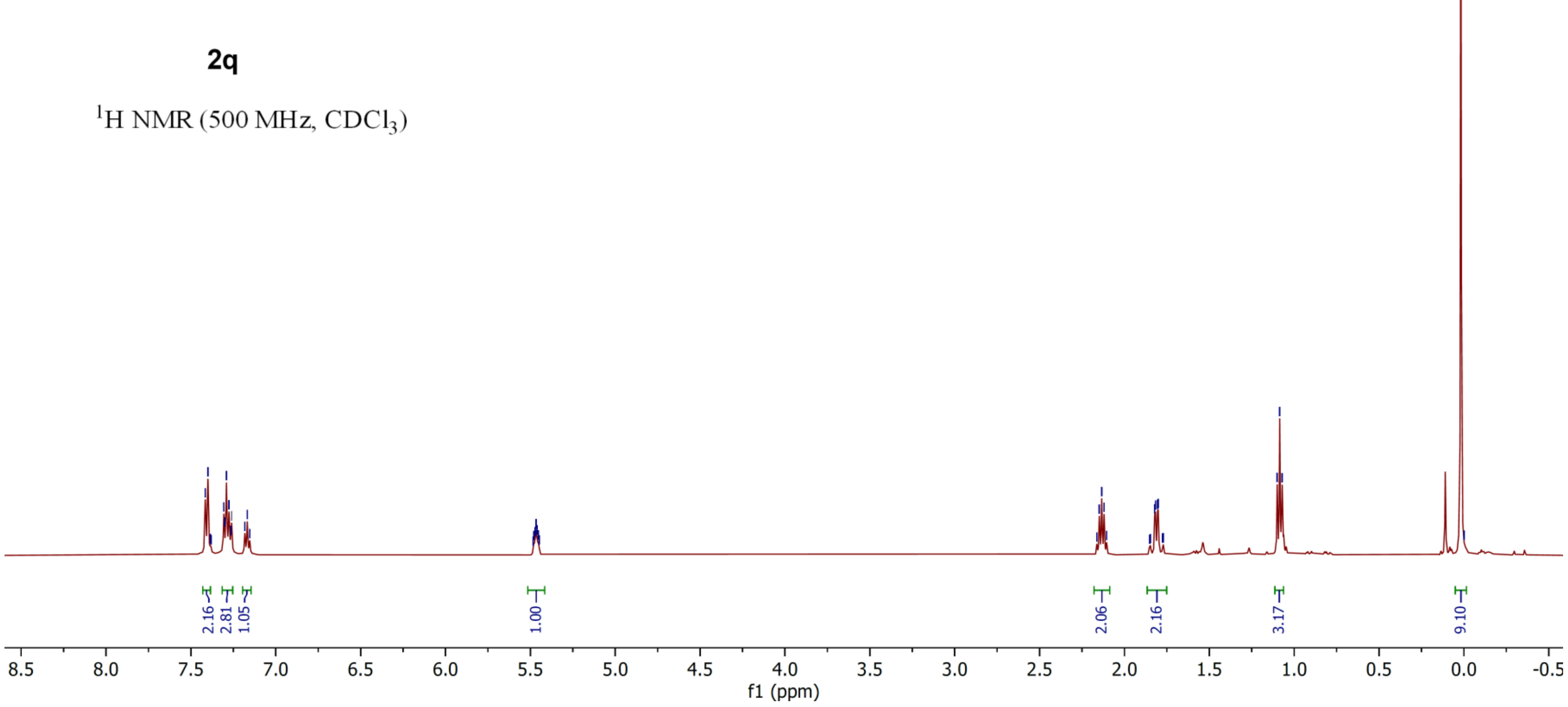

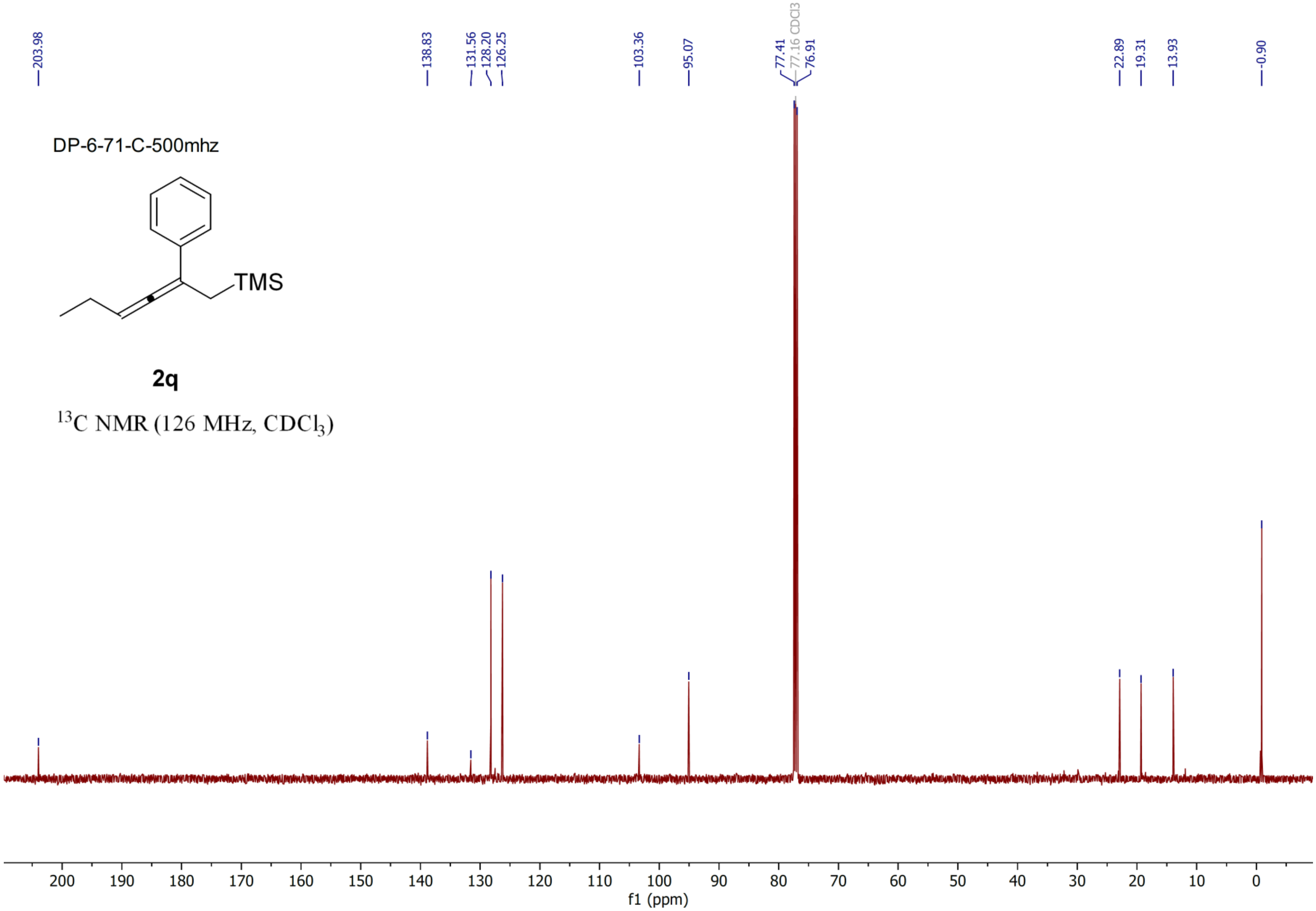

DP-6-71-C-500mhz
TMS
2q
13C NMR (126 MHz, CDCl3)
203.98
138.83
131.56
128.20
126.25
103.36
95.07
77.41
77.16 CDCl3
76.91
22.89
19.31
13.93
-0.90
200 190 180 170 160 150 140 130 120 110 100 90 80 70 60 50 40 30 20 10 0
f1 (ppm)

ABL-D-21-H

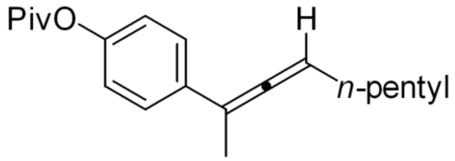

2r

^{1}H NMR (400 MHz, $CDCl_3$)

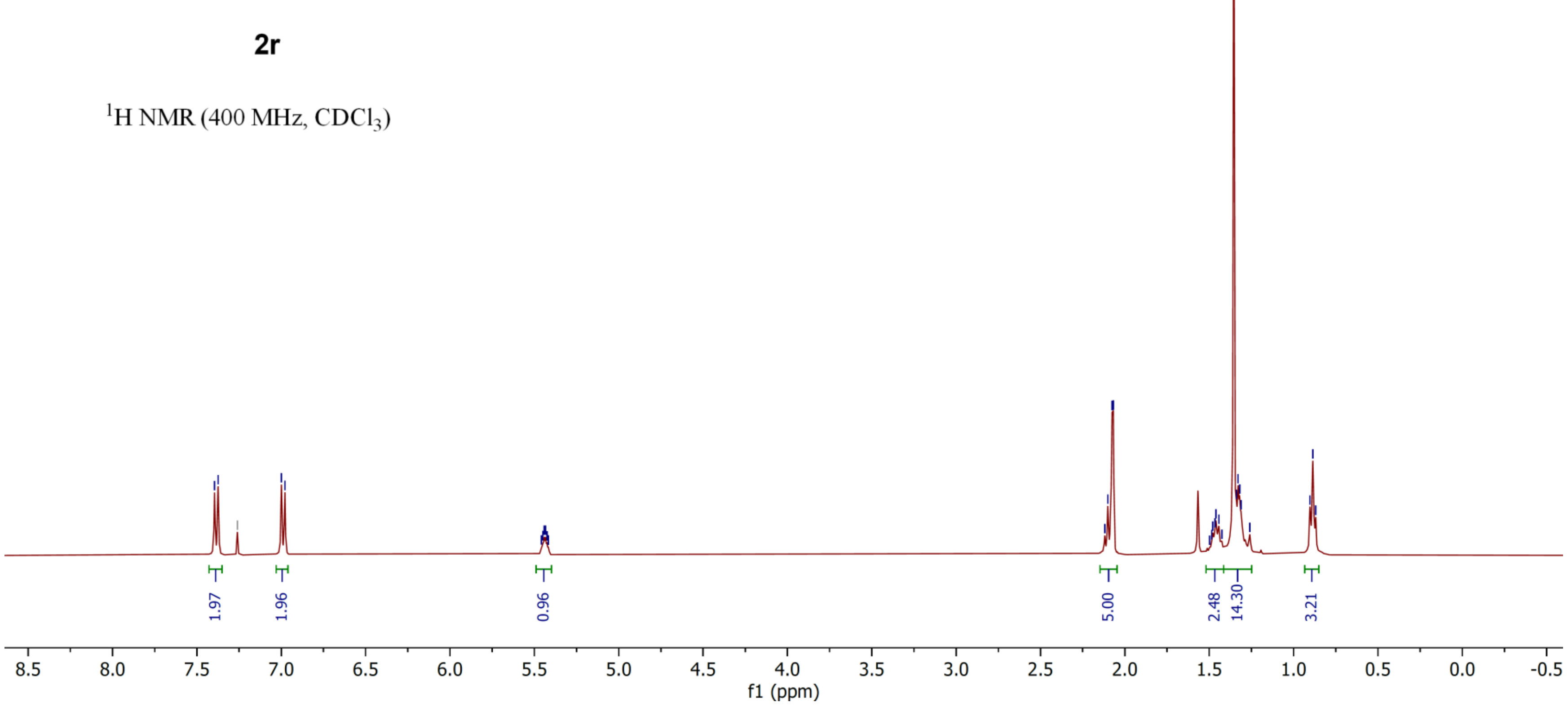

ABL-D-21-C

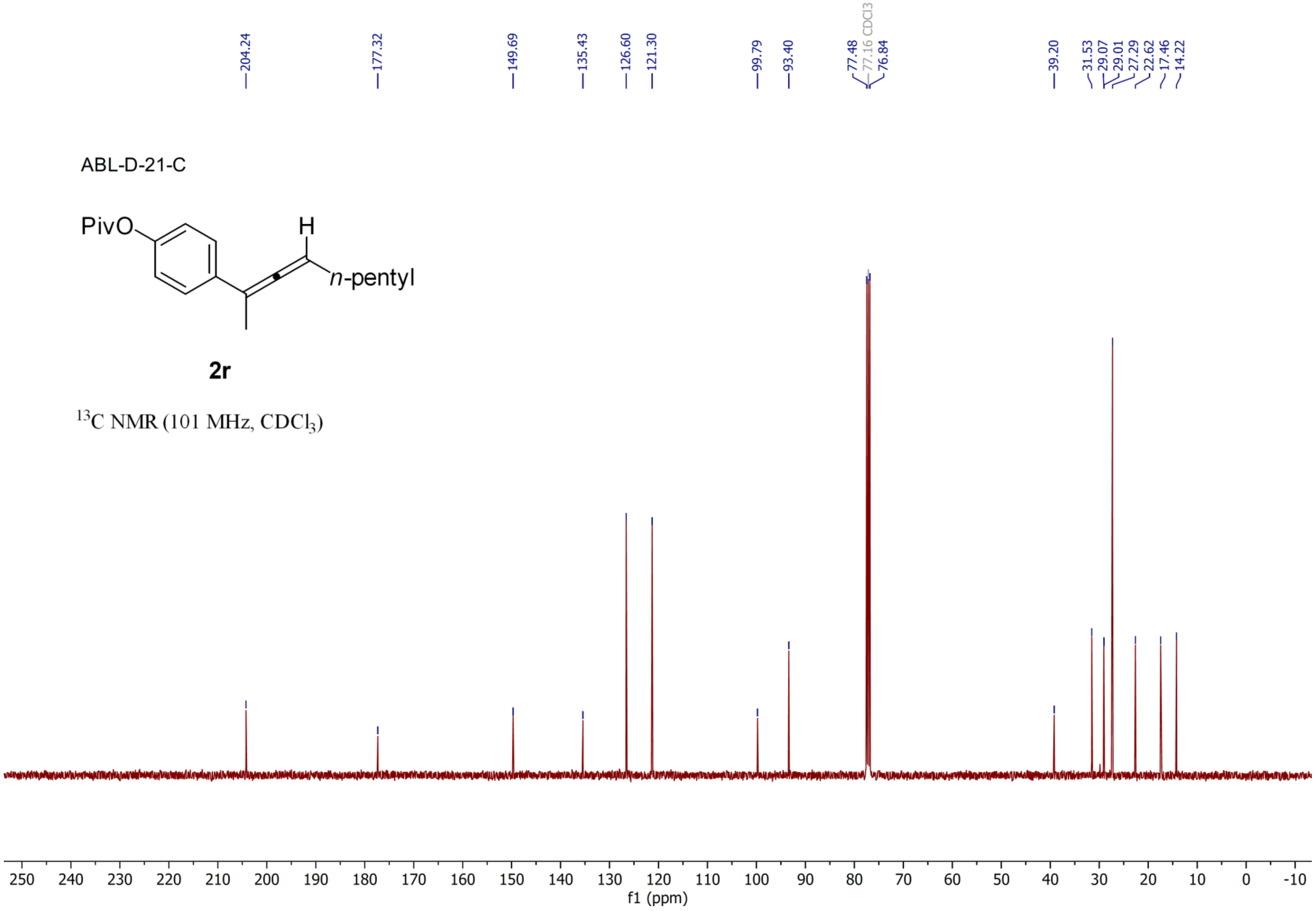

2r

^{13}C NMR (101 MHz, $CDCl_3$)

ABL-D-22-H

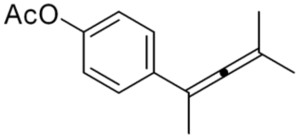

2s

^{1}H NMR (400 MHz, $CDCl_3$)

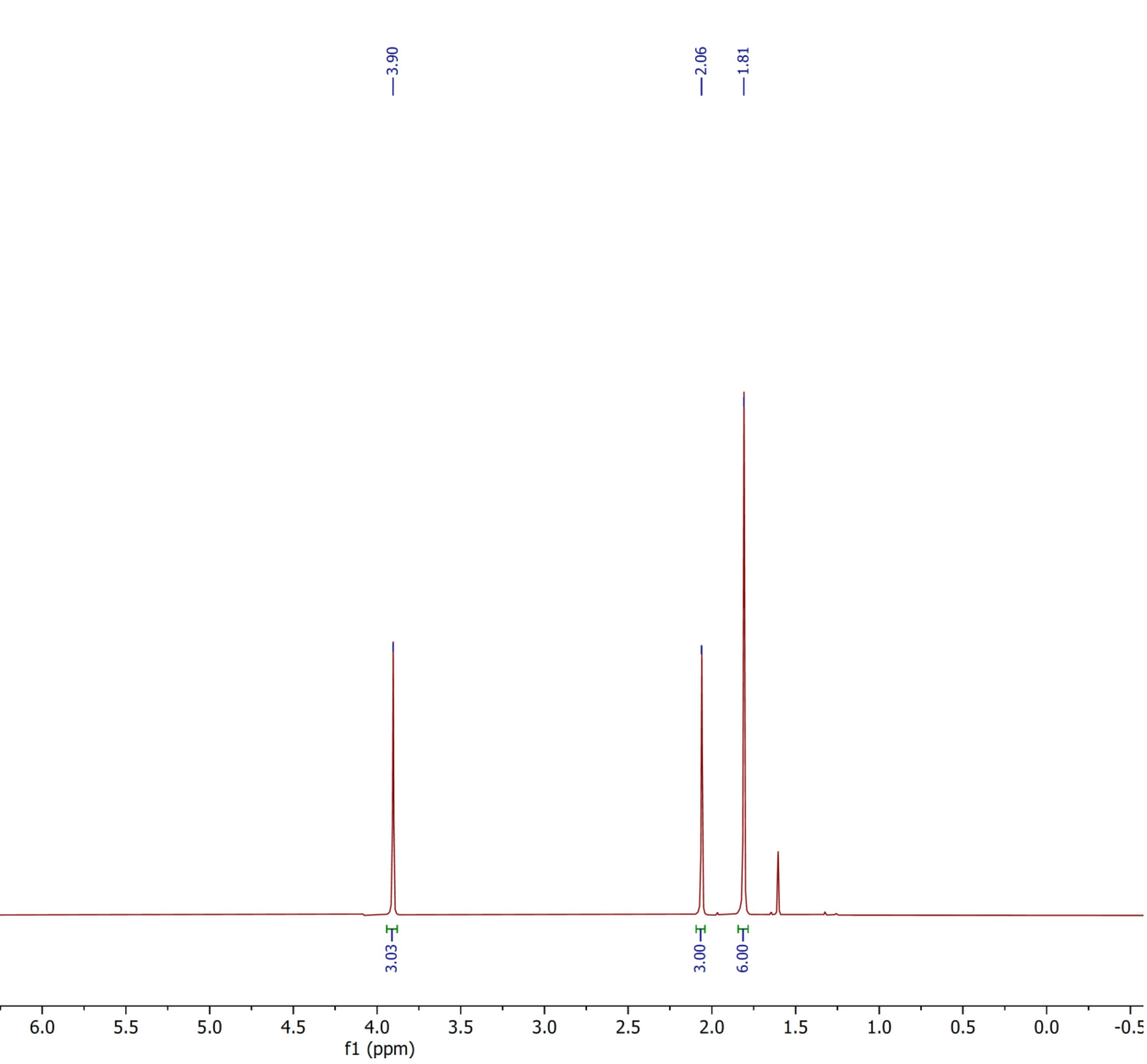

ABL-D-22-C

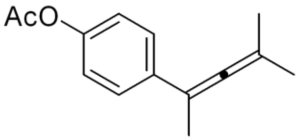

2s

^{13}C NMR (101 MHz, $CDCl_3$)

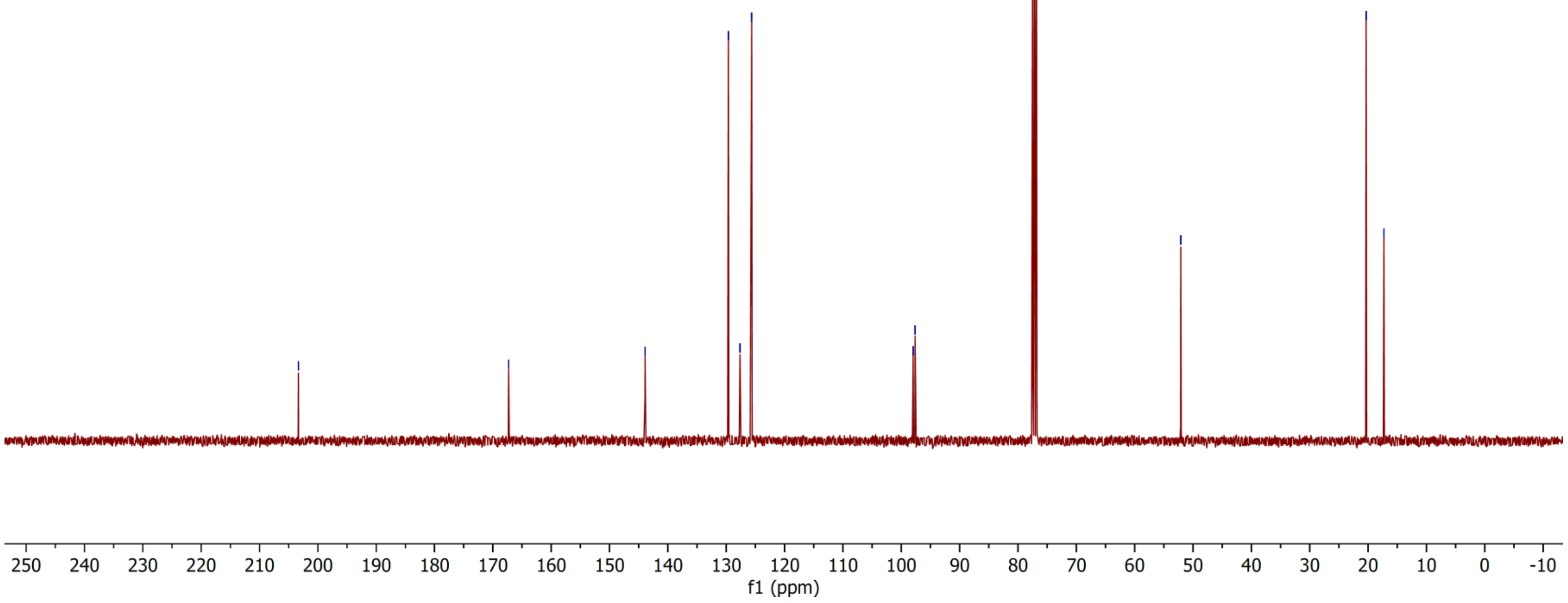

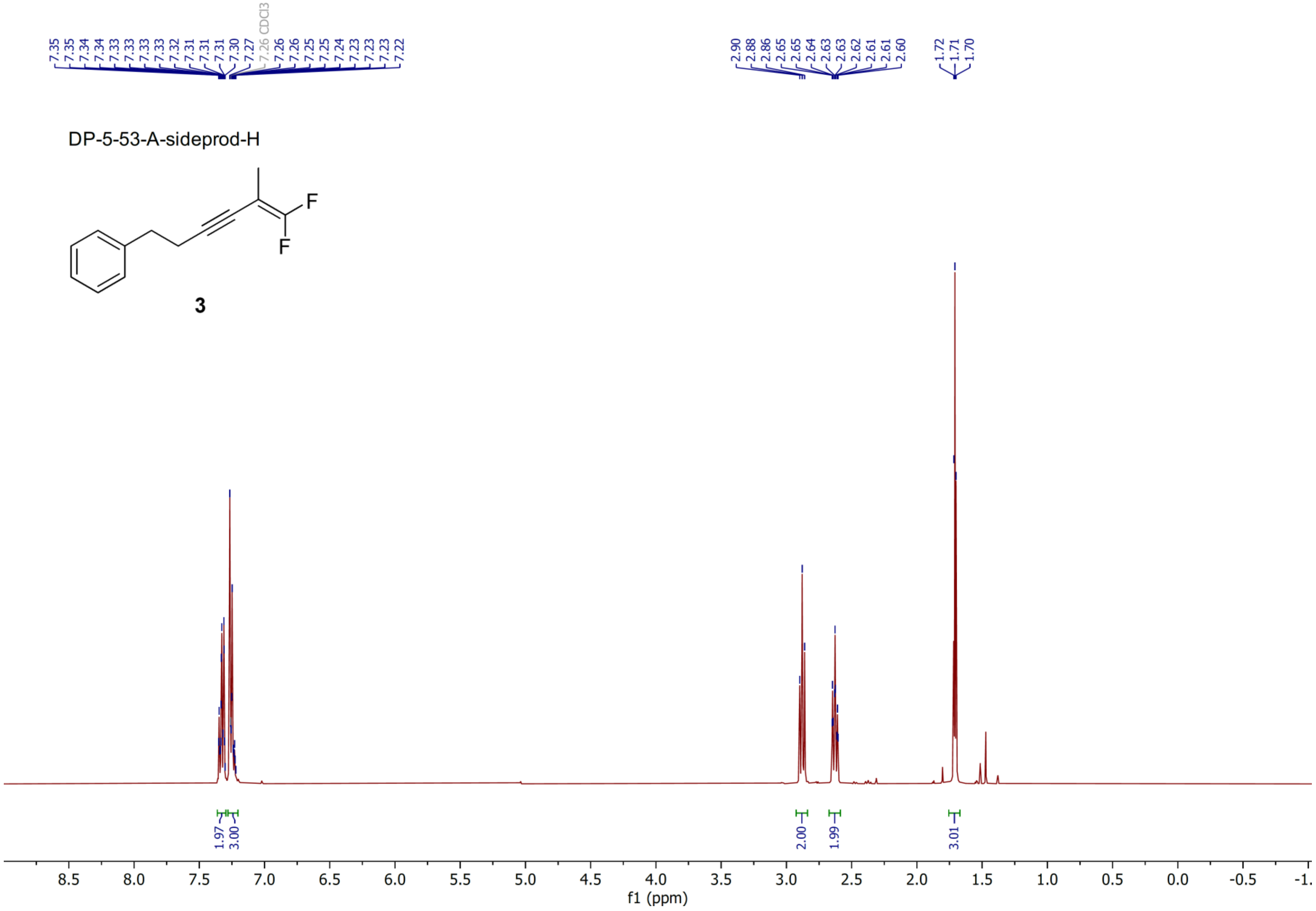

7.35 7.35 7.34 7.34 7.33 7.33 7.33 7.33 7.33 7.32 7.31 7.31 7.31 7.30 7.27 7.26 CDCl3 7.26 7.26 7.25 7.25 7.24 7.23 7.23 7.23 7.22
2.90 2.88 2.86 2.65 2.64 2.63 2.63 2.62 2.61 2.61 2.60
1.72 1.71 1.70
DP-5-53-A-sideprod-H
F
F
3
1.97
3.00
2.00
1.99
3.01
8.5 8.0 7.5 7.0 6.5 6.0 5.5 5.0 4.5 4.0 3.5 3.0 2.5 2.0 1.5 1.0 0.5 0.0 -0.5 -1.
f1 (ppm)

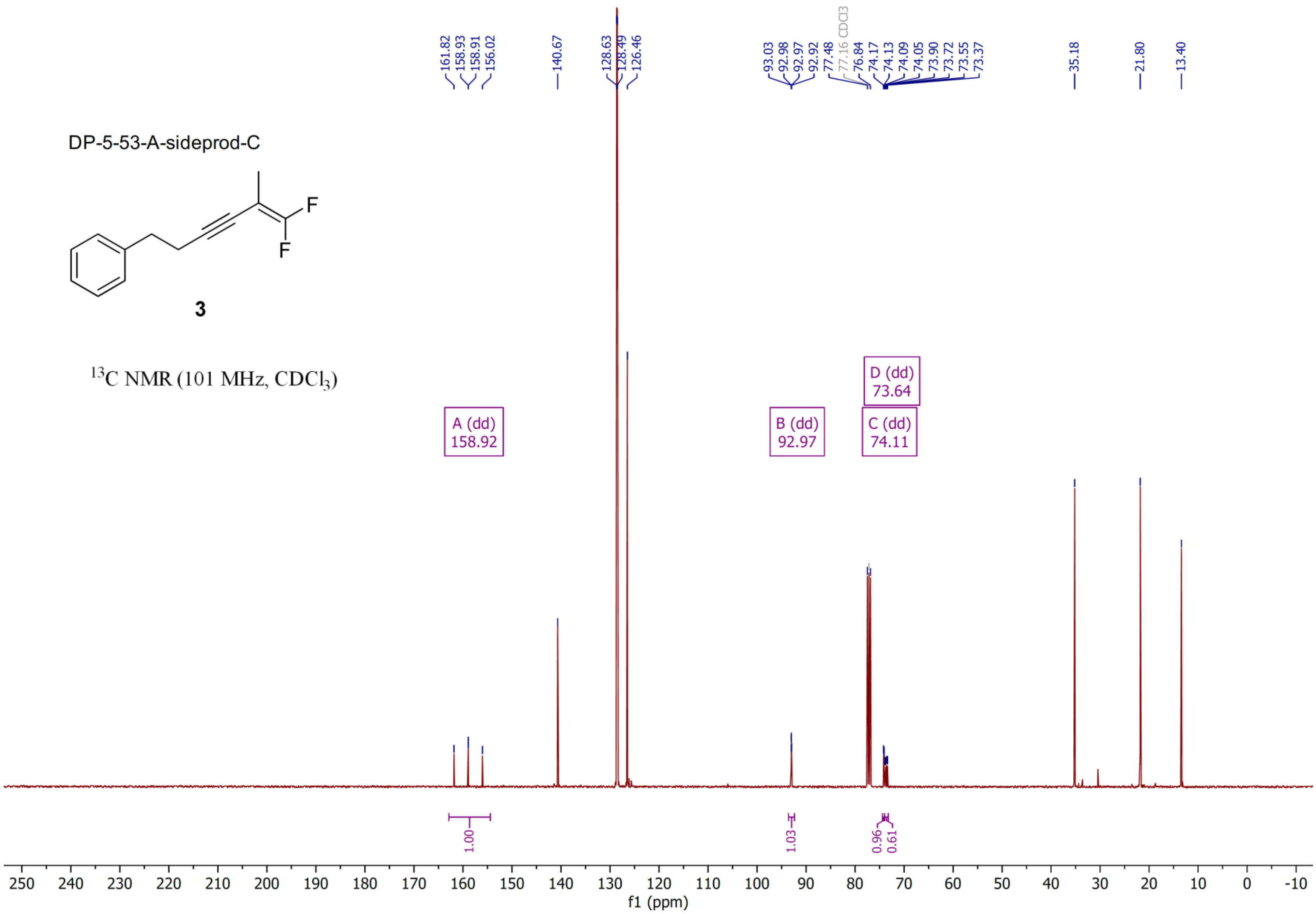
DP-5-53-A-sideprod-C
F
F
3
^{13}C NMR (101 MHz, CDCl$_3$)
161.82
158.93
158.91
156.02
140.67
128.63
128.49
126.46
93.03
92.98
92.97
92.92
77.48
77.16 CDCl3
76.84
74.17
74.13
74.09
74.05
73.90
73.72
73.55
73.37
35.18
21.80
13.40
A (dd)
158.92
B (dd)
92.97
C (dd)
74.11
D (dd)
73.64
1.00
1.03
0.96
0.61
f1 (ppm)

DP-5-53-A-sideprod-F

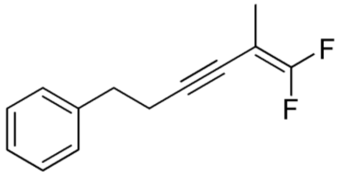

3

^{19}F NMR (377 MHz, $CDCl_3$)

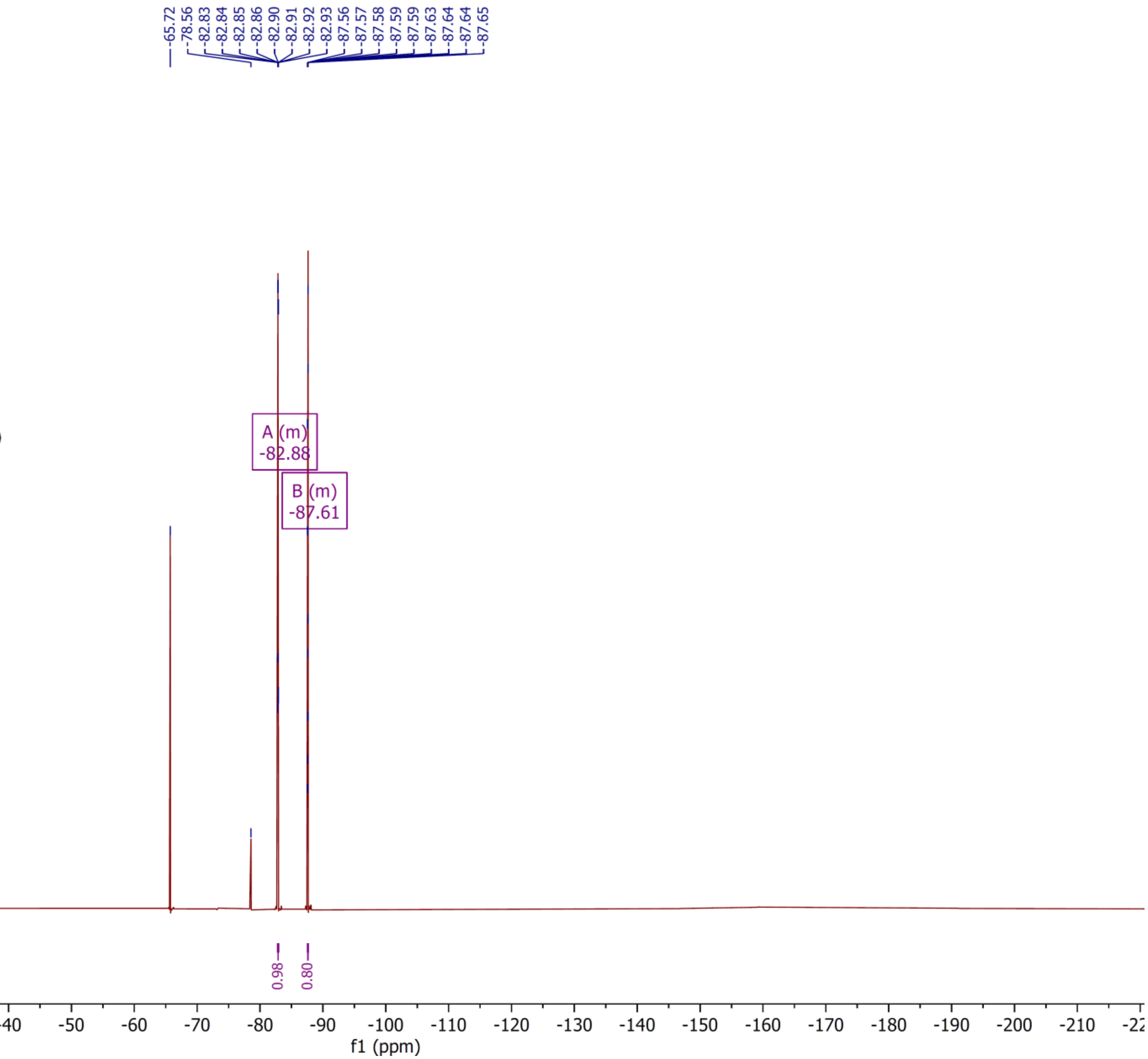

^{1}H and ^{13}C NMR spectra of compounds 5a – 5i:

DP-5-104-H

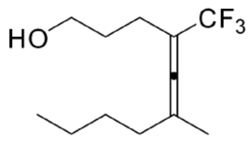

5a

^{1}H NMR (400 MHz, $CDCl_3$)

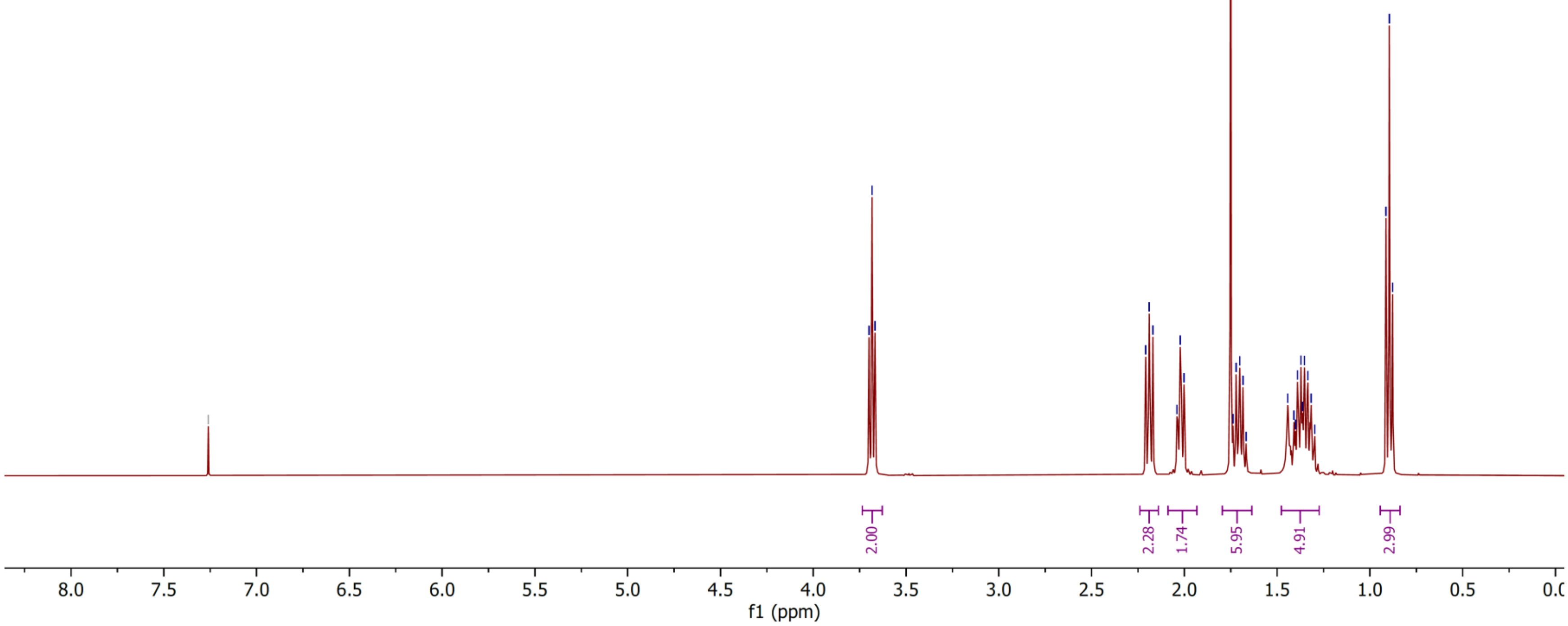

DP-5-104-C

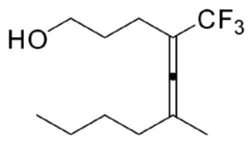

5a

^{13}C NMR (101 MHz, $CDCl_3$)

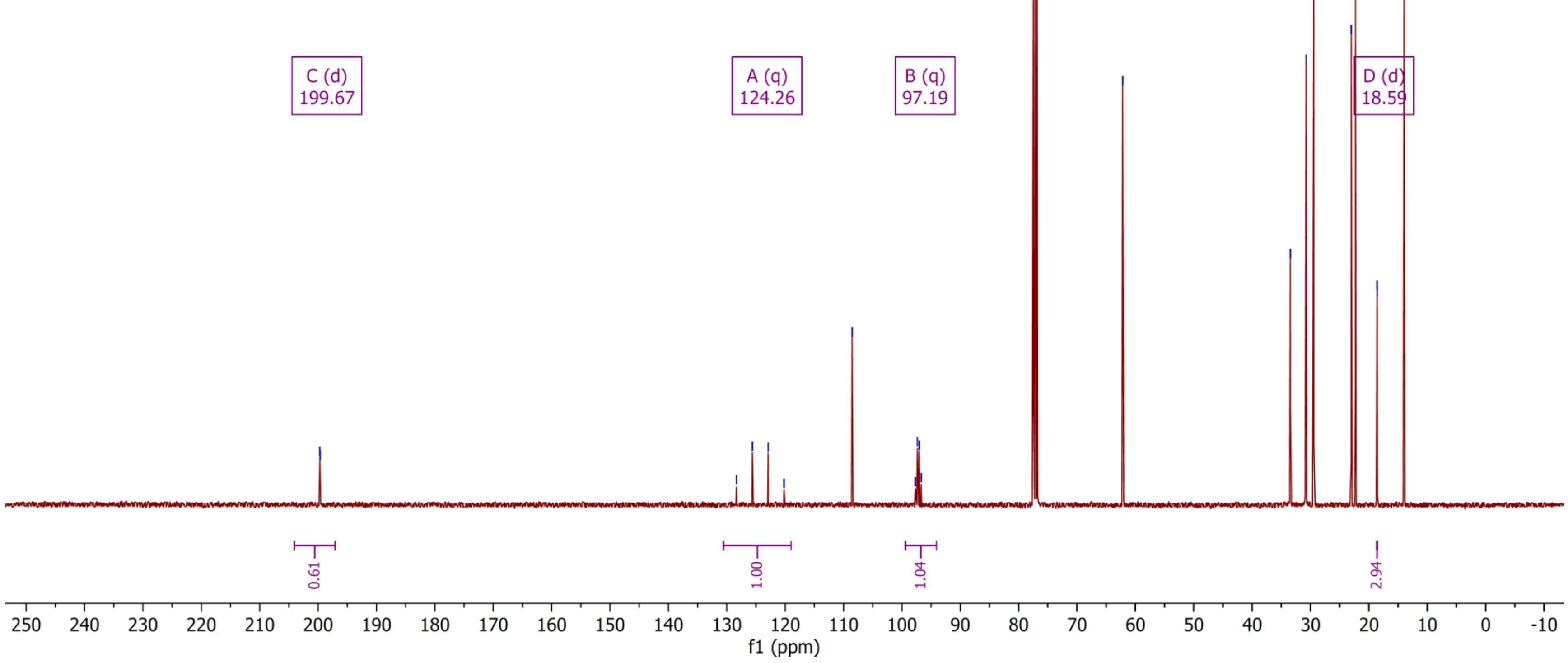

DP-5-104-F

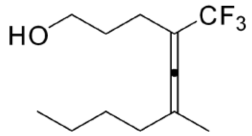

5a

^{19}F NMR (377 MHz, $CDCl_3$)

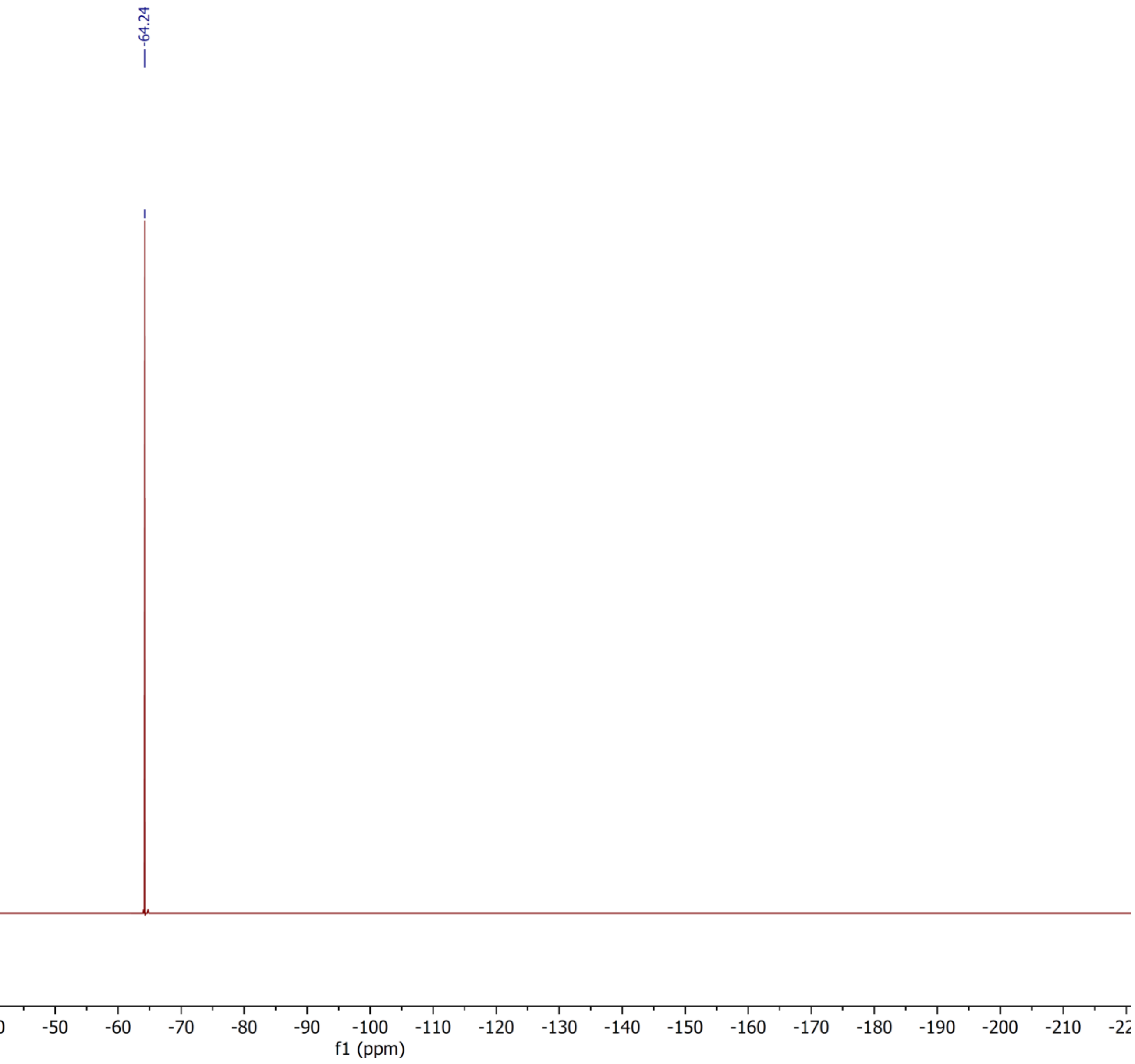

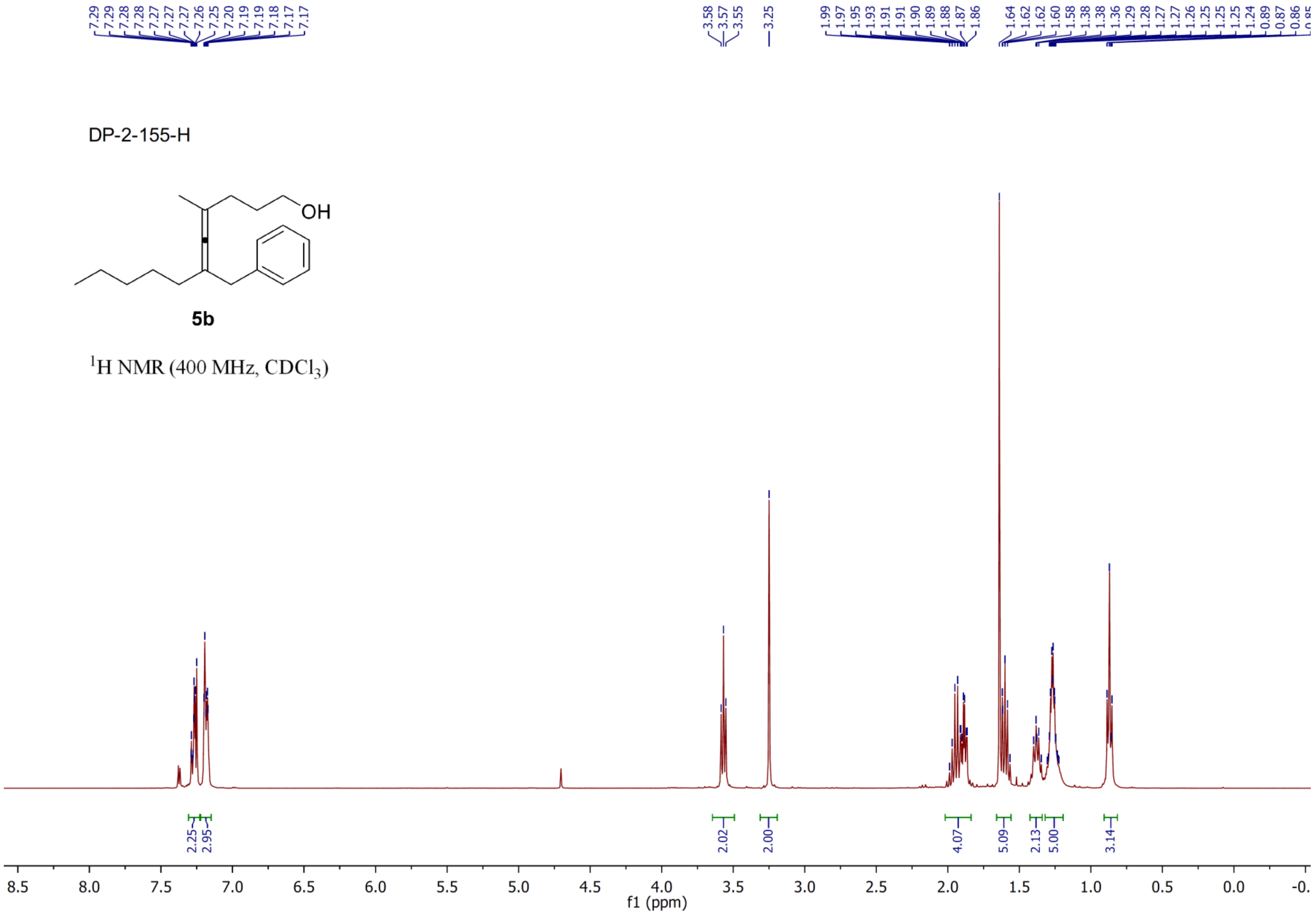
DP-2-155-H
OH
5b
¹H NMR (400 MHz, CDCl₃)
f1 (ppm)

DP-2-155-C

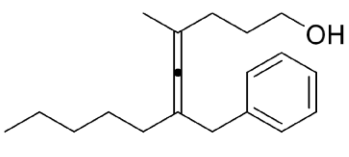

5b

^{13}C NMR (101 MHz, $CDCl_3$)

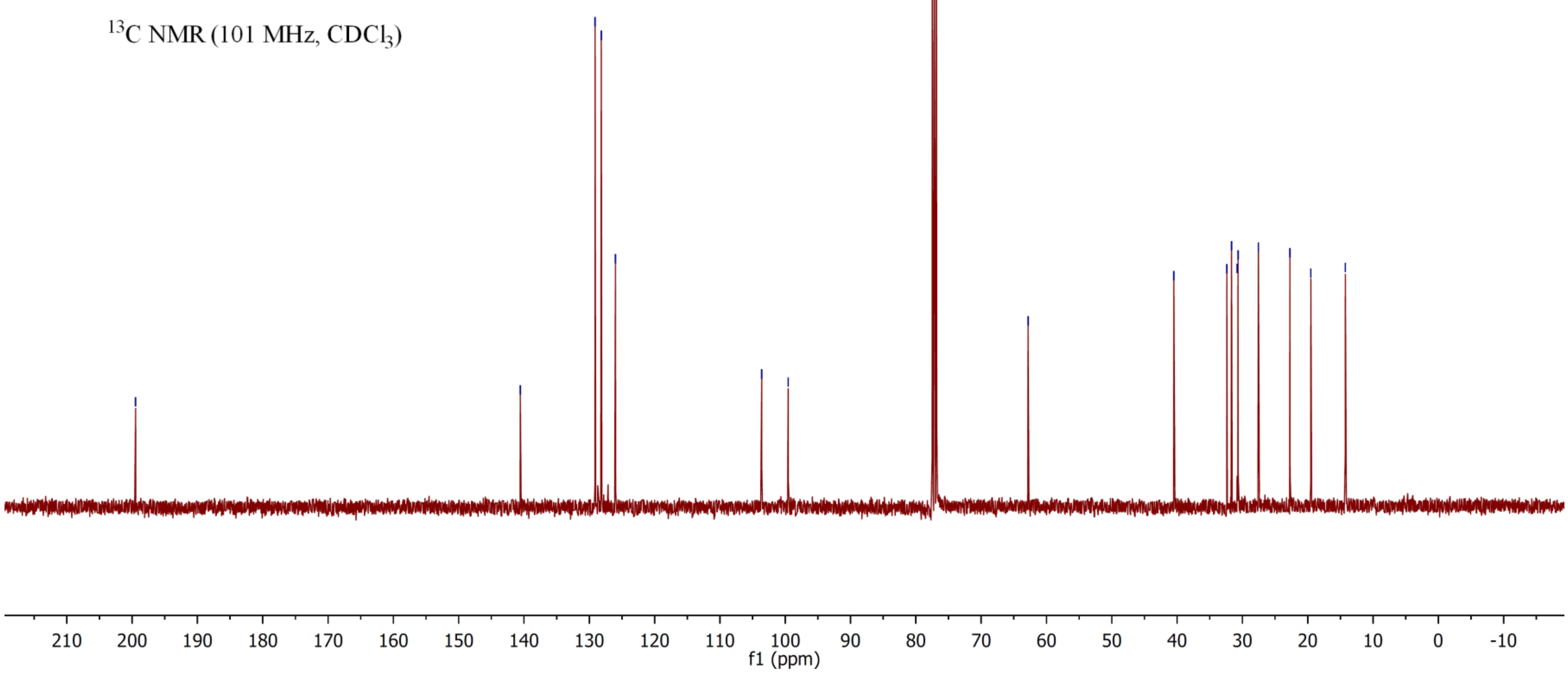

DP-5-158-H

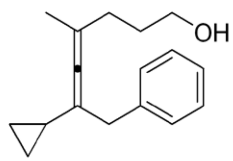

5c

^{1}H NMR (400 MHz, $CDCl_3$)

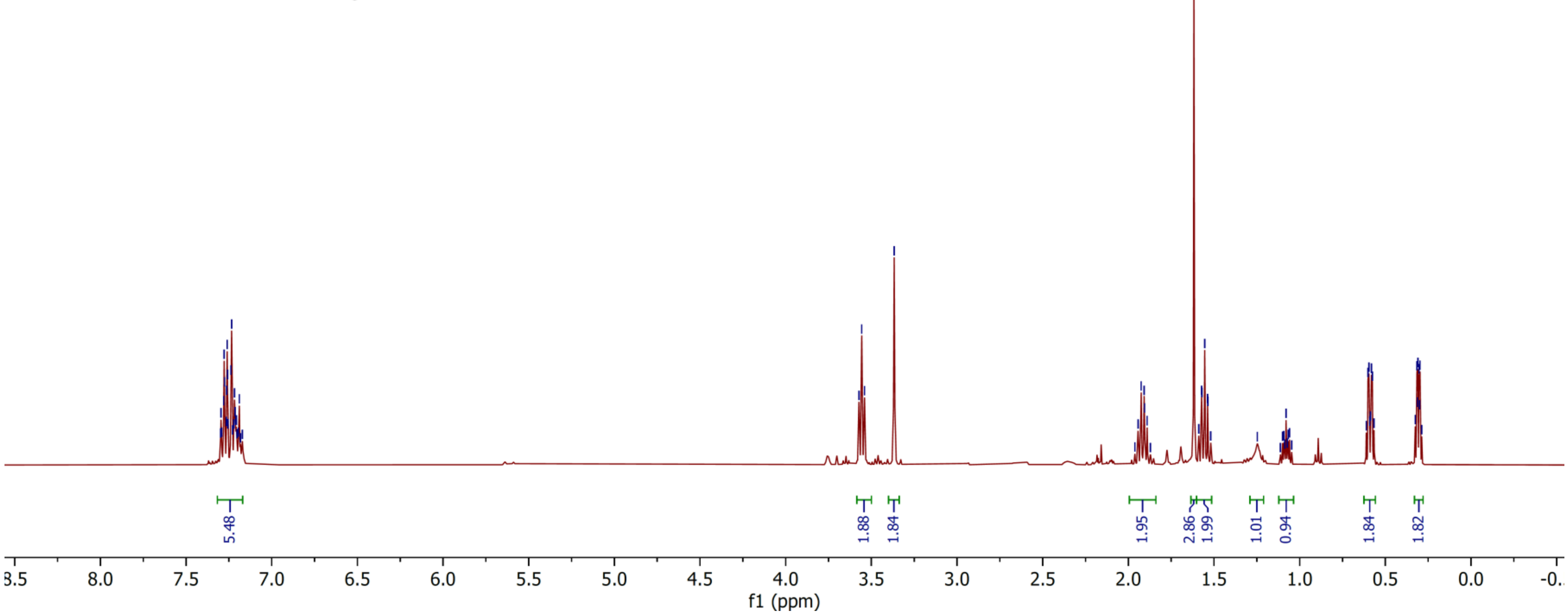

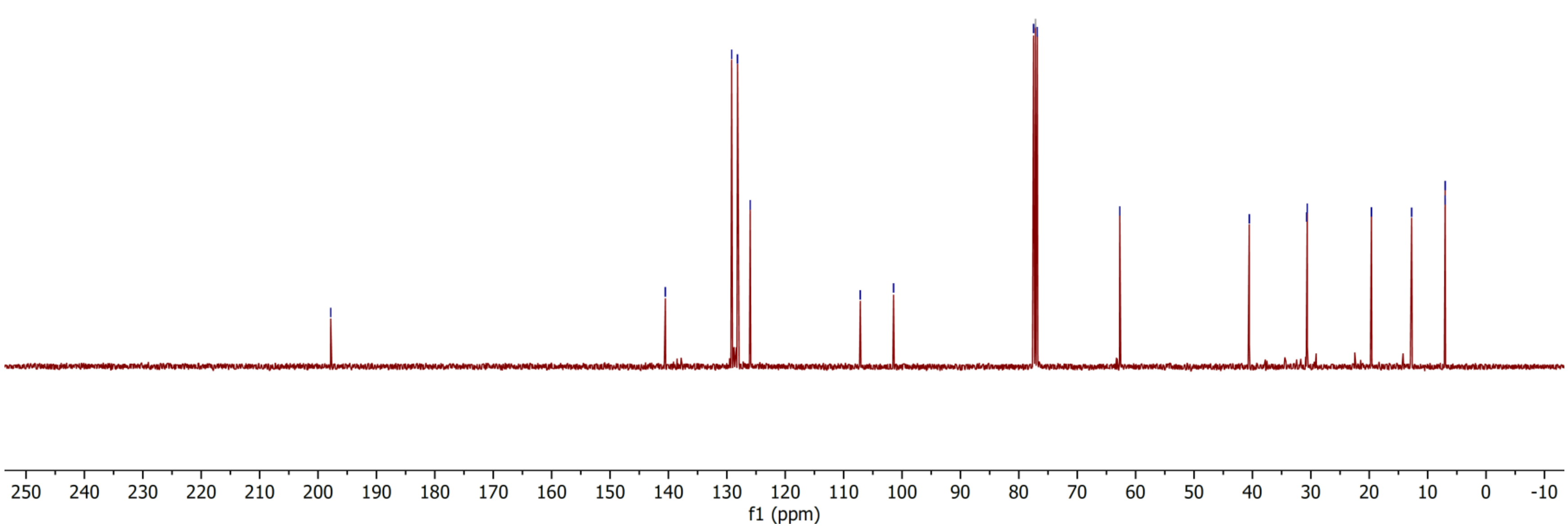

DP-5-158-C

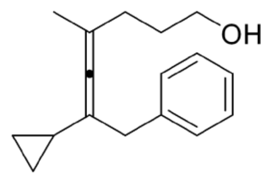

5c

^{13}C NMR (101 MHz, $CDCl_3$)

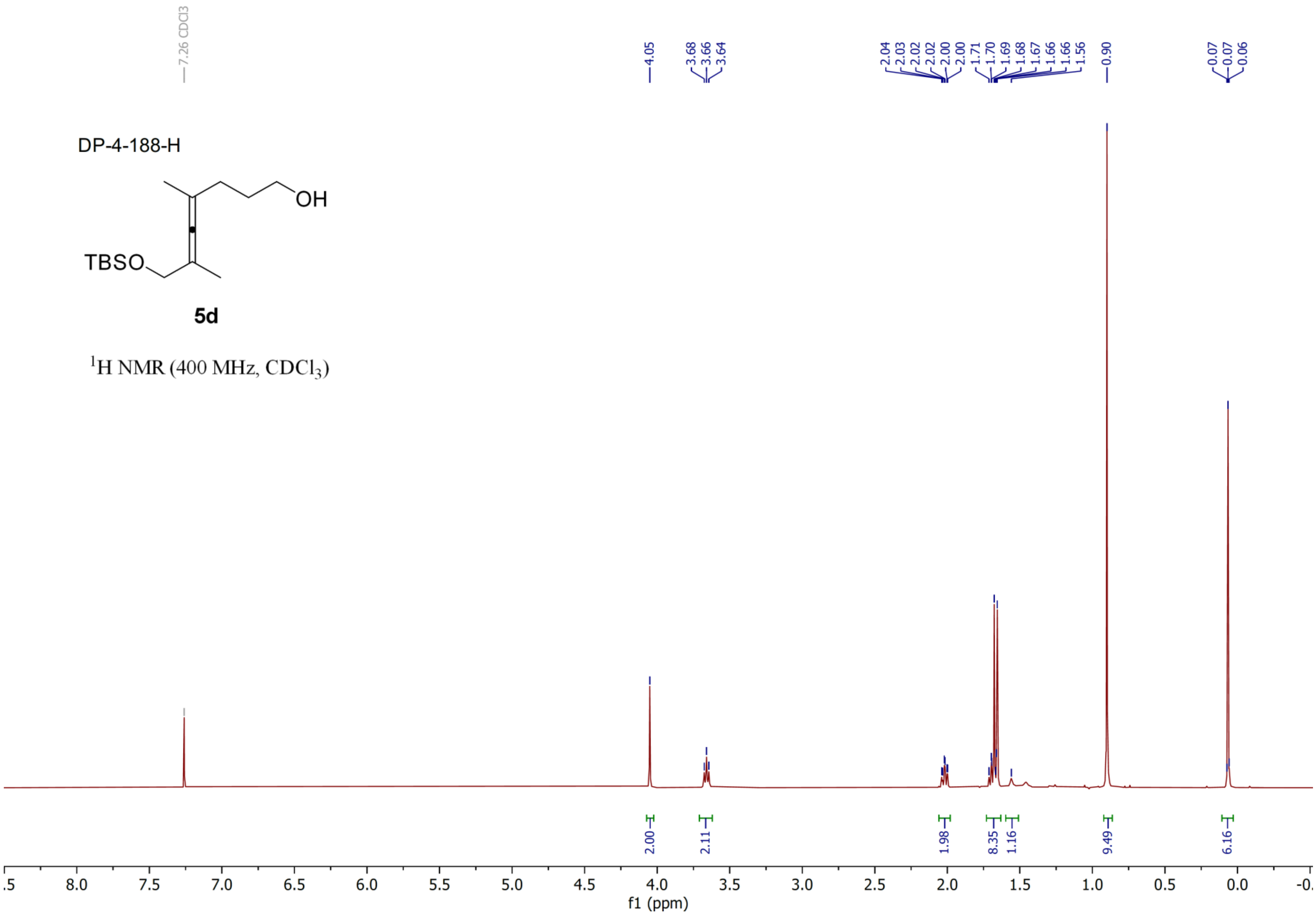
DP-4-188-H
OH
TBSO
5d
^{1}H NMR (400 MHz, CDCl$_3$)
7.26 CDCl3
4.05
3.68
3.66
3.64
2.04
2.03
2.02
2.02
2.00
2.00
1.71
1.70
1.69
1.68
1.67
1.66
1.66
1.56
0.90
0.07
0.07
0.06
2.00
2.11
1.98
8.35
1.16
9.49
6.16
f1 (ppm)

DP-4-188-C

5d

^{13}C NMR (101 MHz, $CDCl_3$)

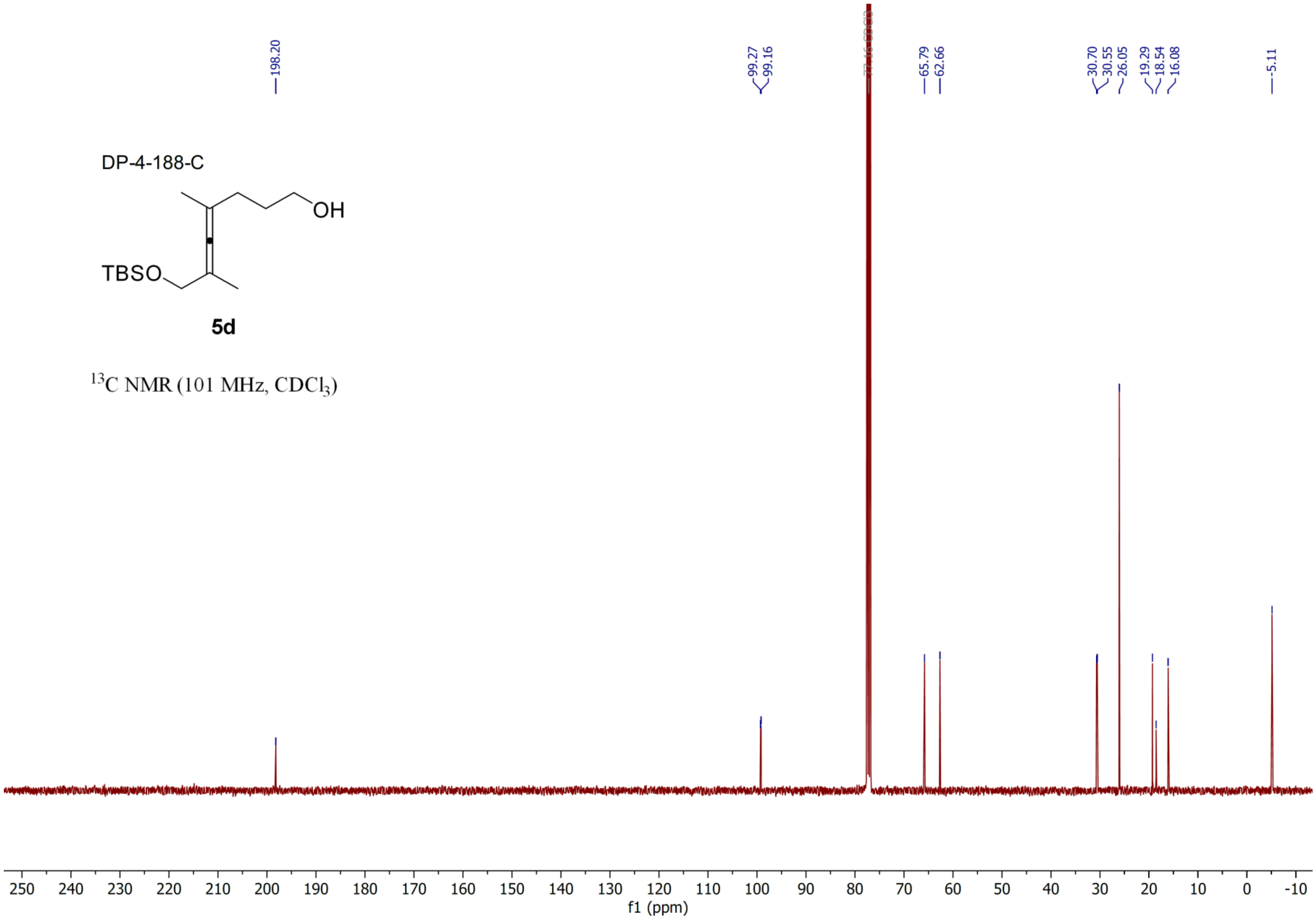

DP-4-191-H

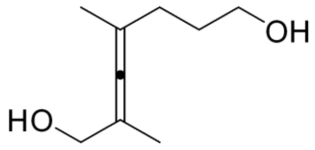

5e

^{1}H NMR (400 MHz, $CDCl_3$)

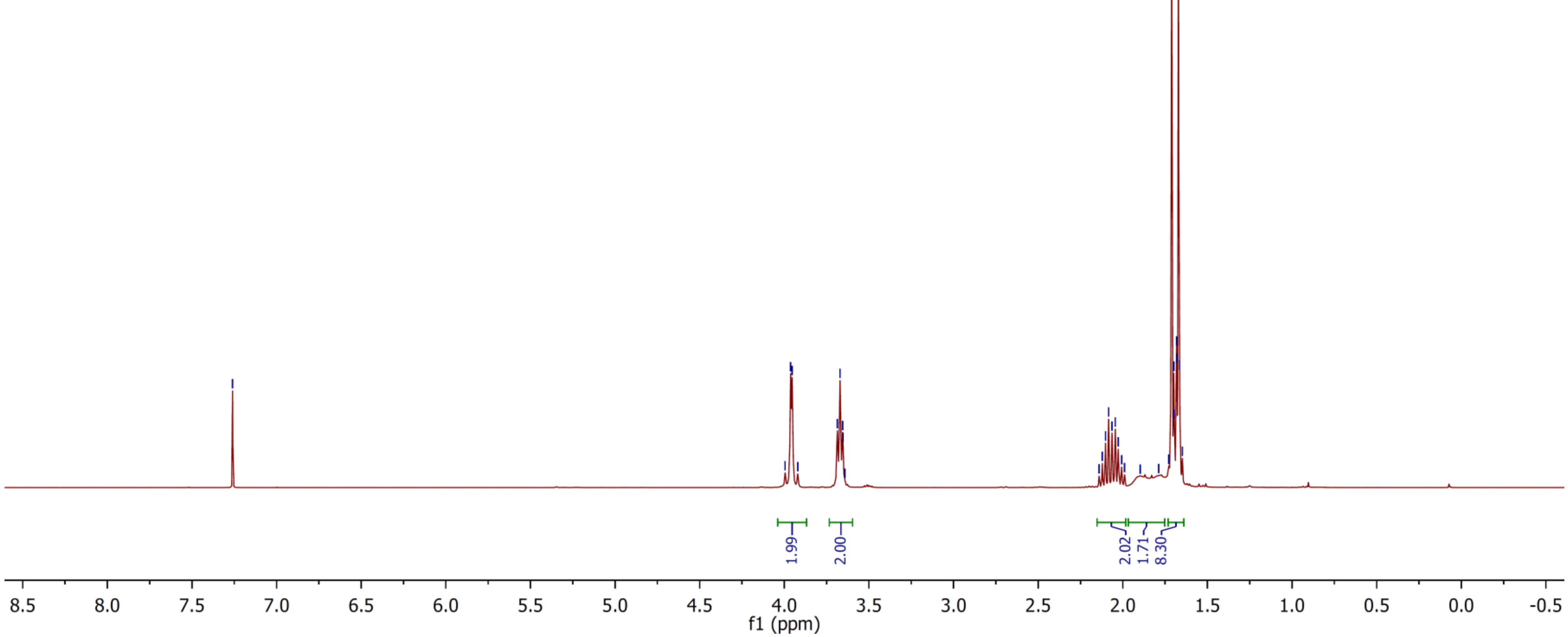

DP-4-191-C

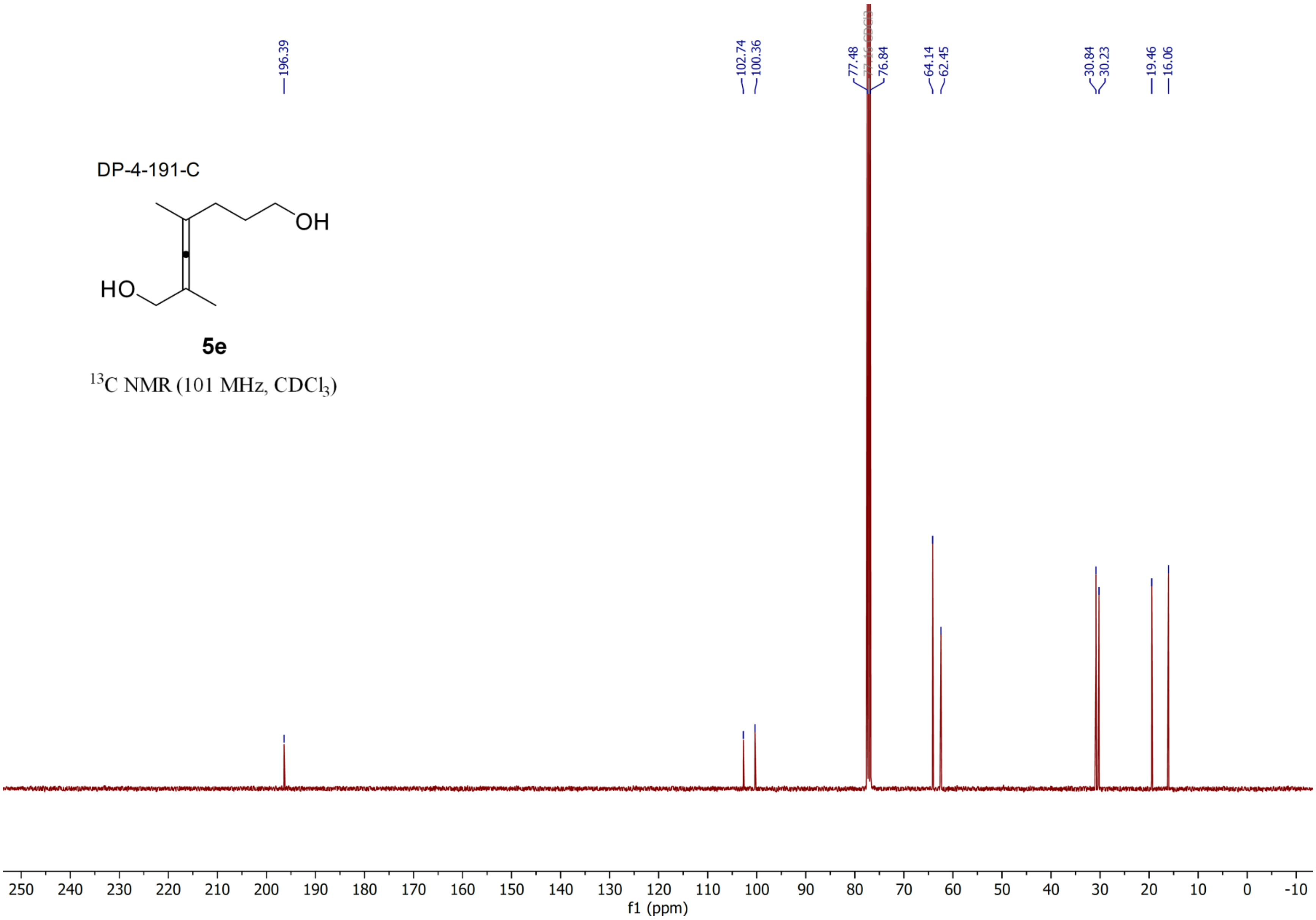

5e

^{13}C NMR (101 MHz, $CDCl_3$)

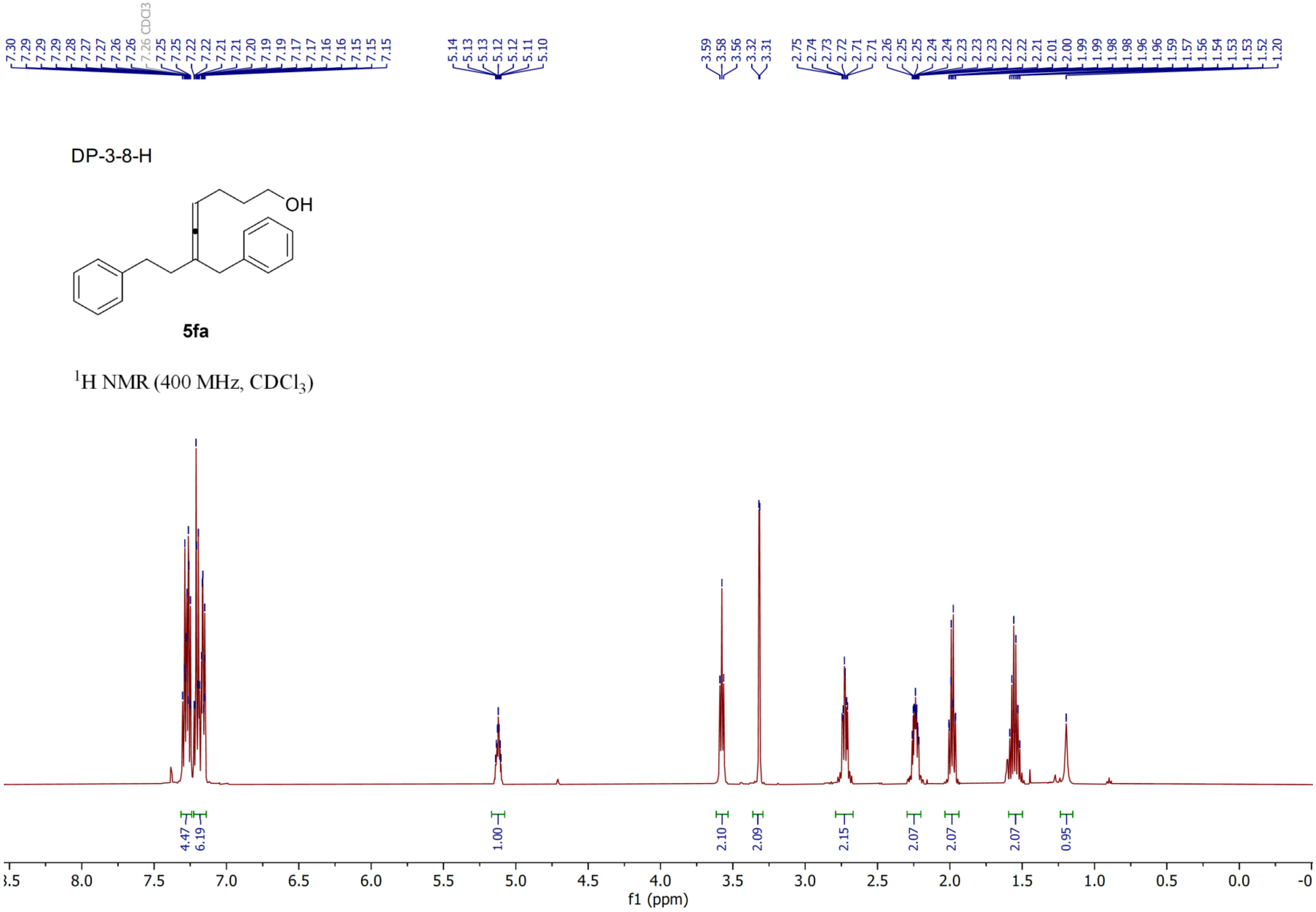
DP-3-8-H
OH
5fa
1H NMR (400 MHz, CDCl3)
f1 (ppm)

DP-3-8-C

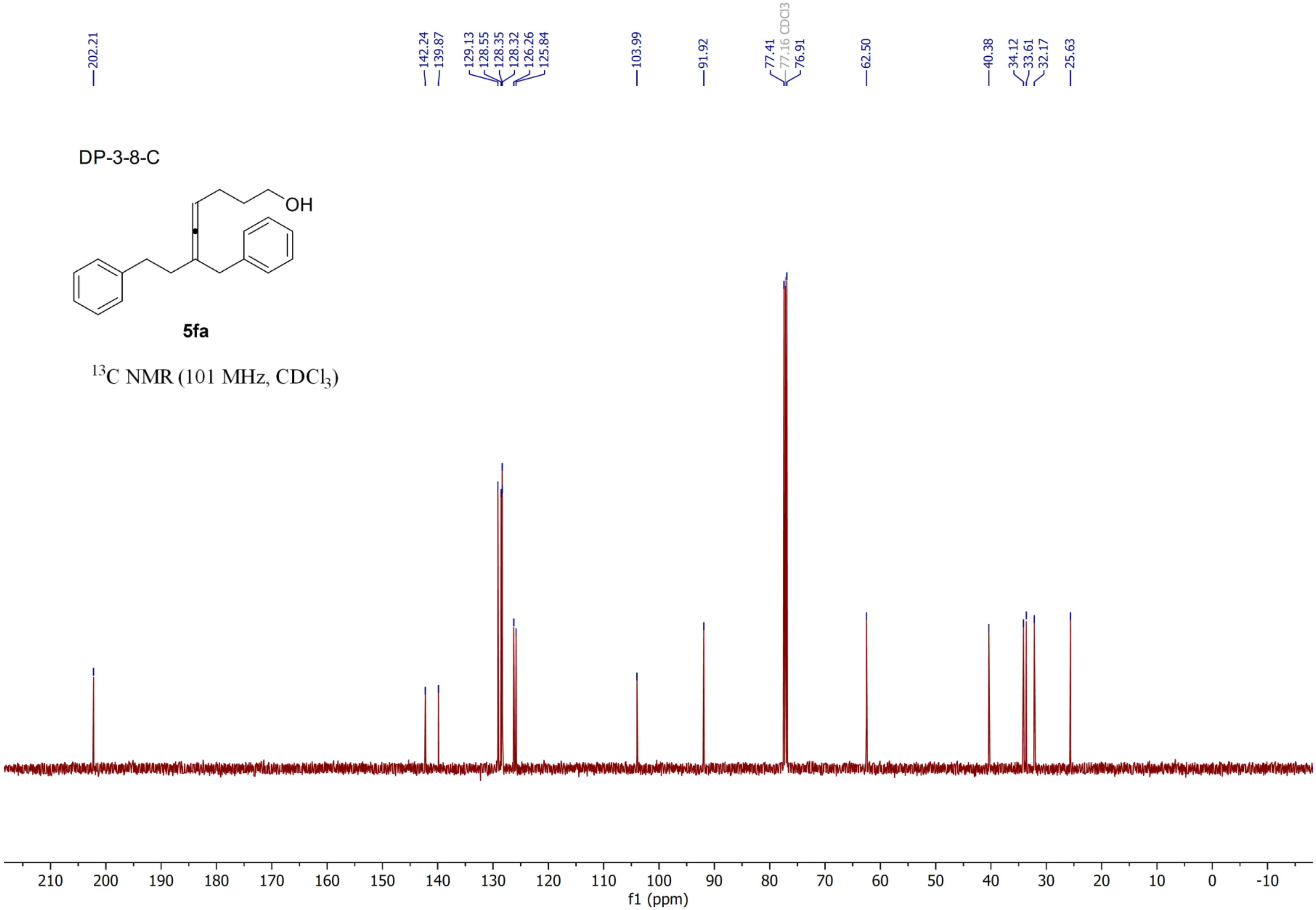

^{13}C NMR (101 MHz, $CDCl_3$)

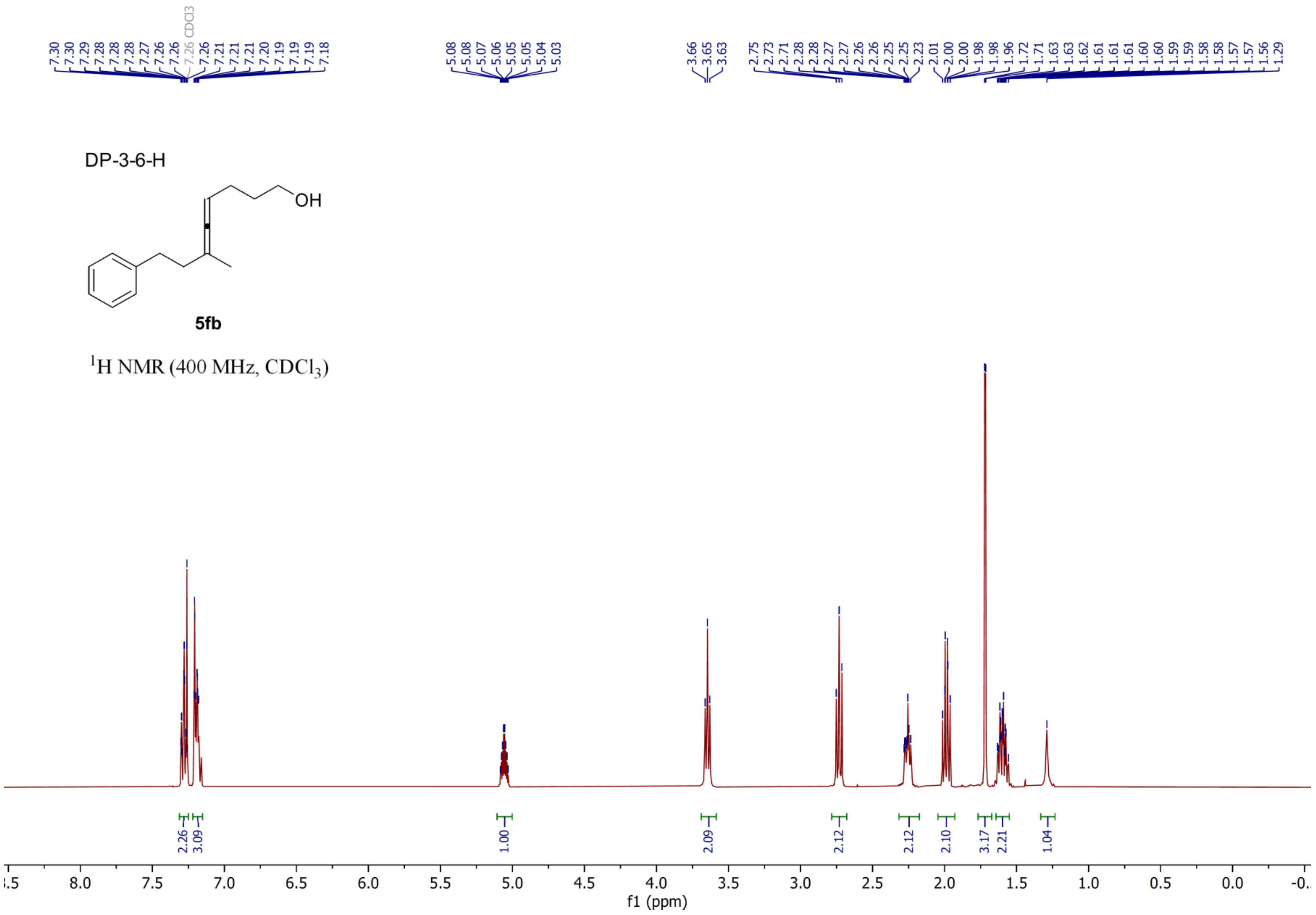
DP-3-6-H
OH
5fb
^{1}H NMR (400 MHz, $CDCl_3$)
7.30 7.30 7.29 7.28 7.28 7.28 7.27 7.26 7.26 7.26 CDCl3 7.26 7.21 7.21 7.21 7.20 7.19 7.19 7.19 7.18
5.08 5.08 5.07 5.06 5.05 5.05 5.04 5.03
3.66 3.65 3.63
2.75 2.73 2.71 2.28 2.28 2.27 2.27 2.26 2.26 2.25 2.25 2.23 2.01 2.00 2.00 1.98 1.98 1.96 1.72 1.71 1.63 1.63 1.62 1.61 1.61 1.61 1.60 1.60 1.59 1.59 1.58 1.58 1.57 1.57 1.56 1.29
2.26 3.09 1.00 2.09 2.12 2.12 2.10 3.17 2.21 1.04
f1 (ppm)

DP-3-6-C

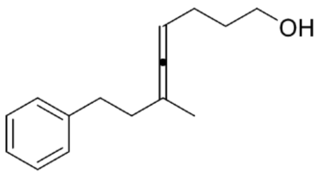

5fb

^{13}C NMR (101 MHz, $CDCl_3$)

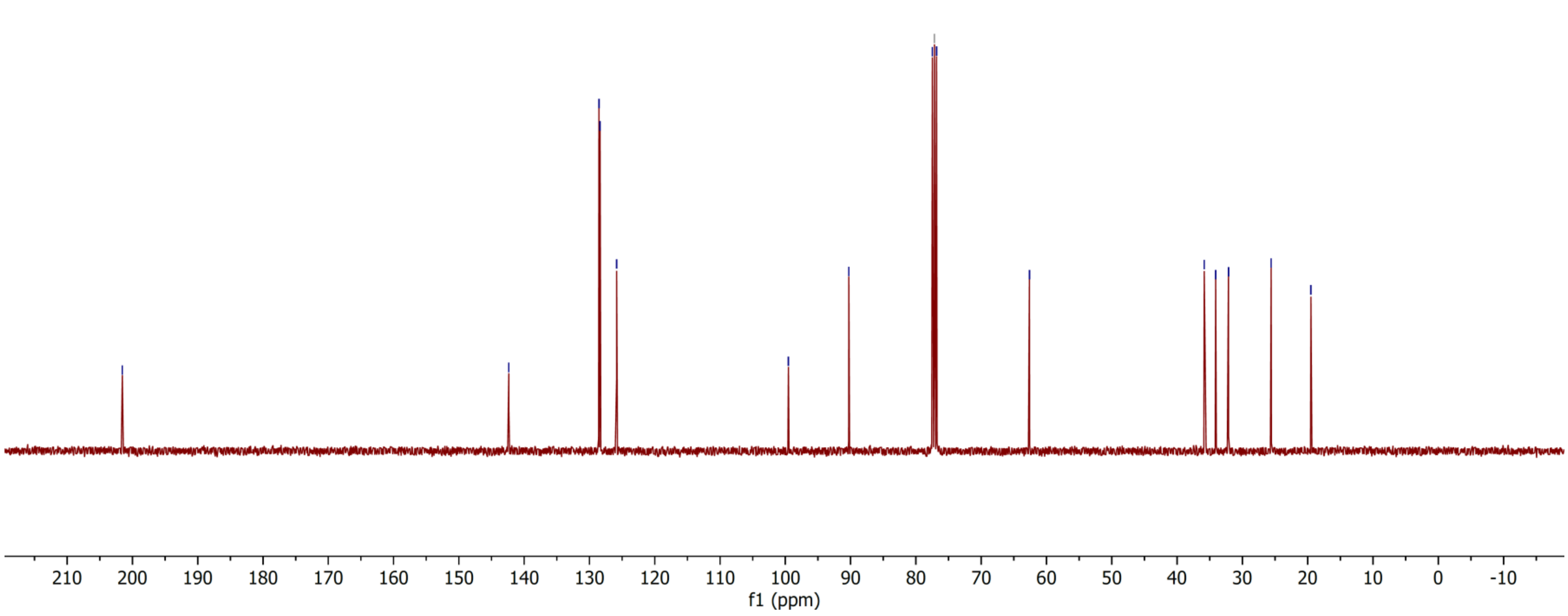

DP-5-143-H-500mhz

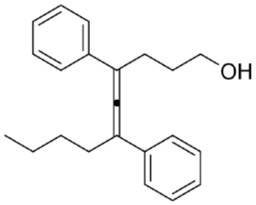

5g

^{1}H NMR (500 MHz, $CDCl_3$)

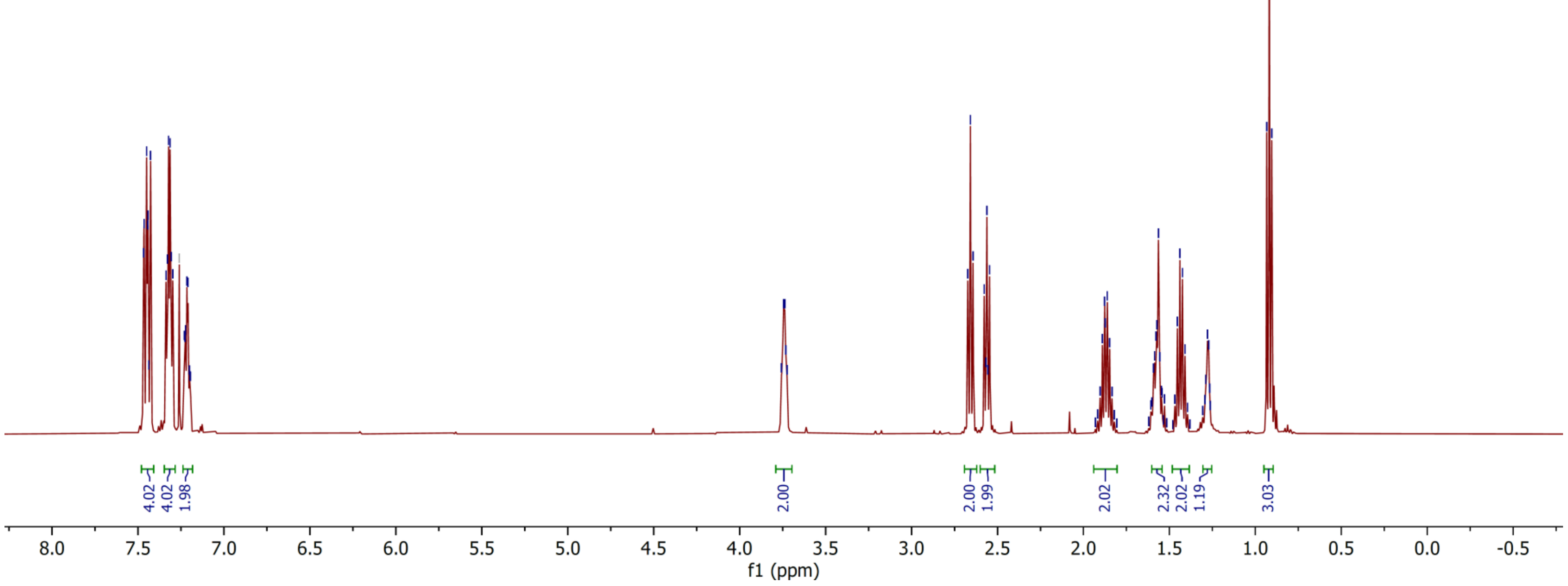

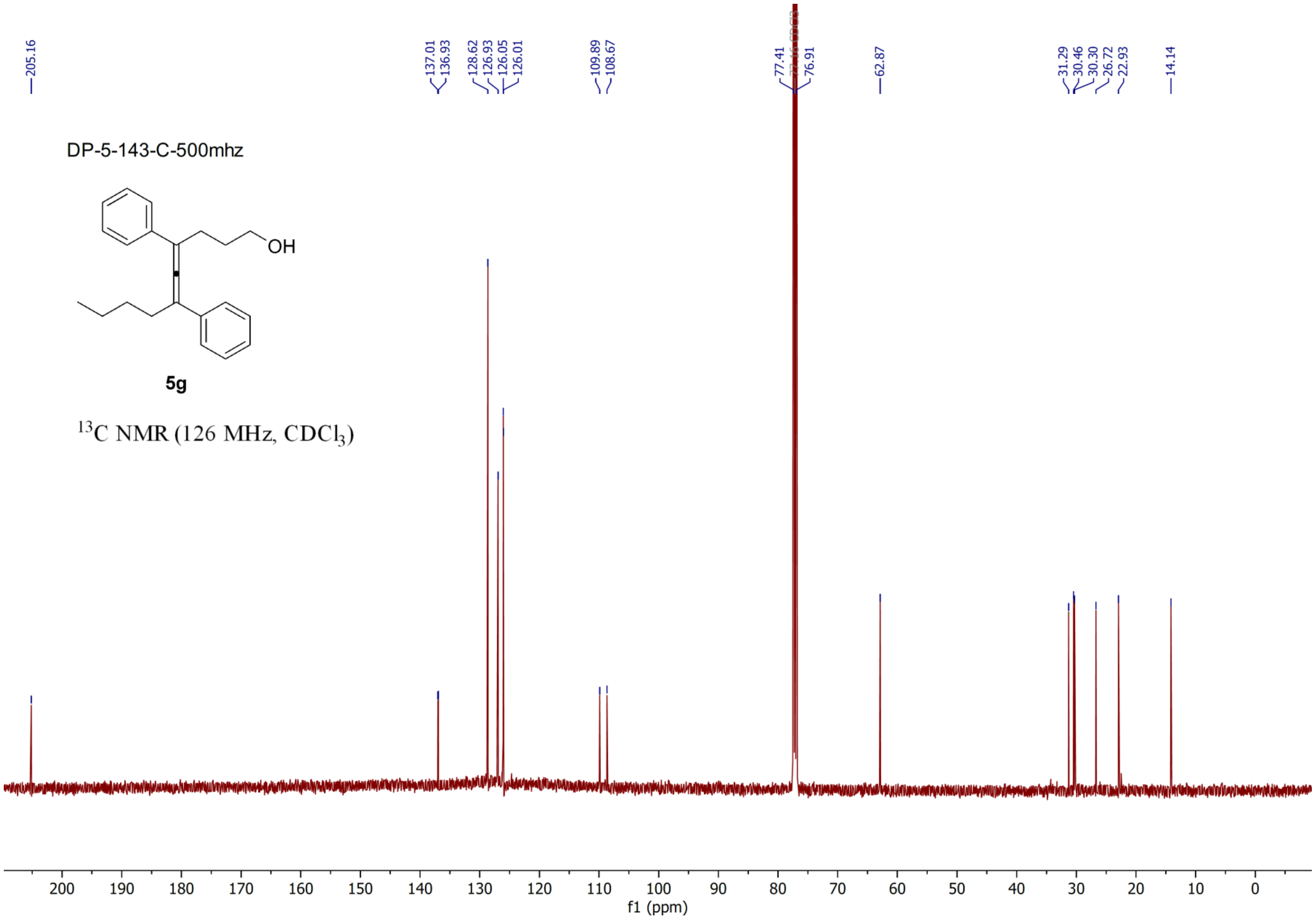

205.16
137.01
136.93
128.62
126.93
126.05
126.01
109.89
108.67
77.41
76.91
62.87
31.29
30.46
30.30
26.72
22.93
14.14
DP-5-143-C-500mhz
OH
5g
^{13}C NMR (126 MHz, $CDCl_3$)
200 190 180 170 160 150 140 130 120 110 100 90 80 70 60 50 40 30 20 10 0
f1 (ppm)

DP-5-105-H

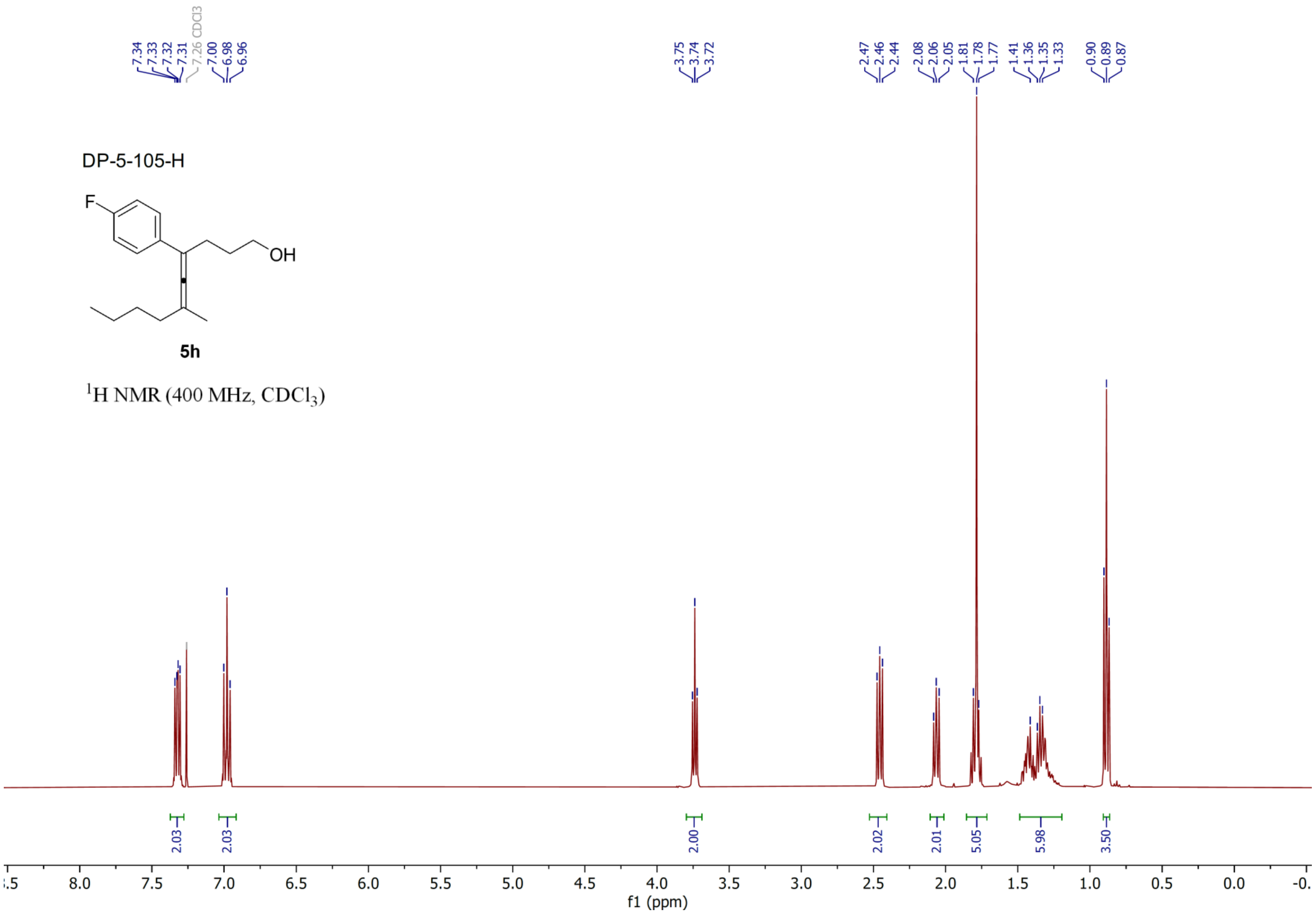

5h

^{1}H NMR (400 MHz, $CDCl_3$)

DP-5-105-C

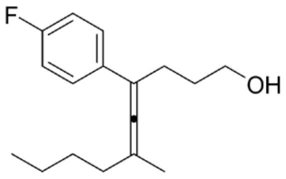

5h

^{13}C NMR (101 MHz, $CDCl_3$)

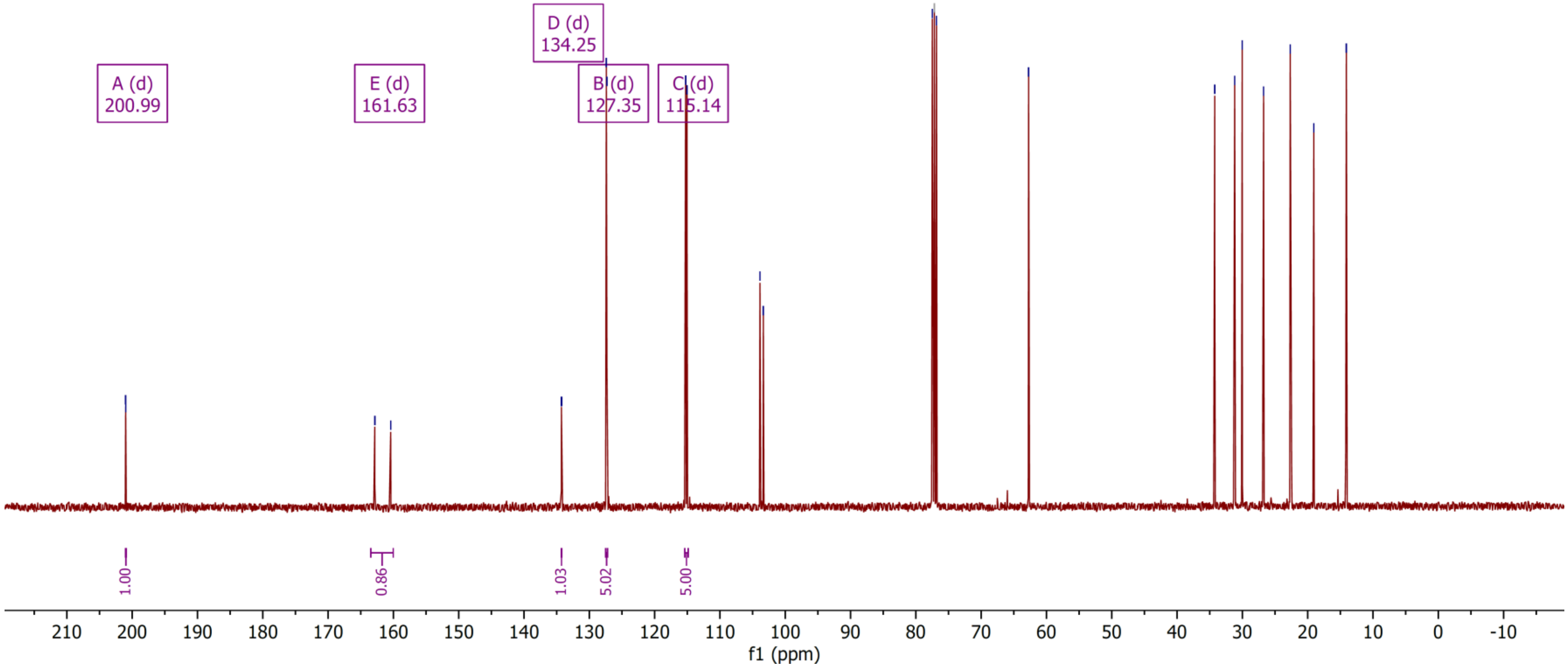

DP-5-105-F

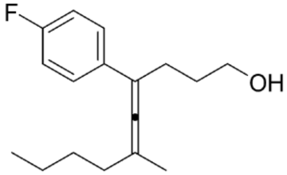

5h

^{19}F NMR (377 MHz, $CDCl_3$)

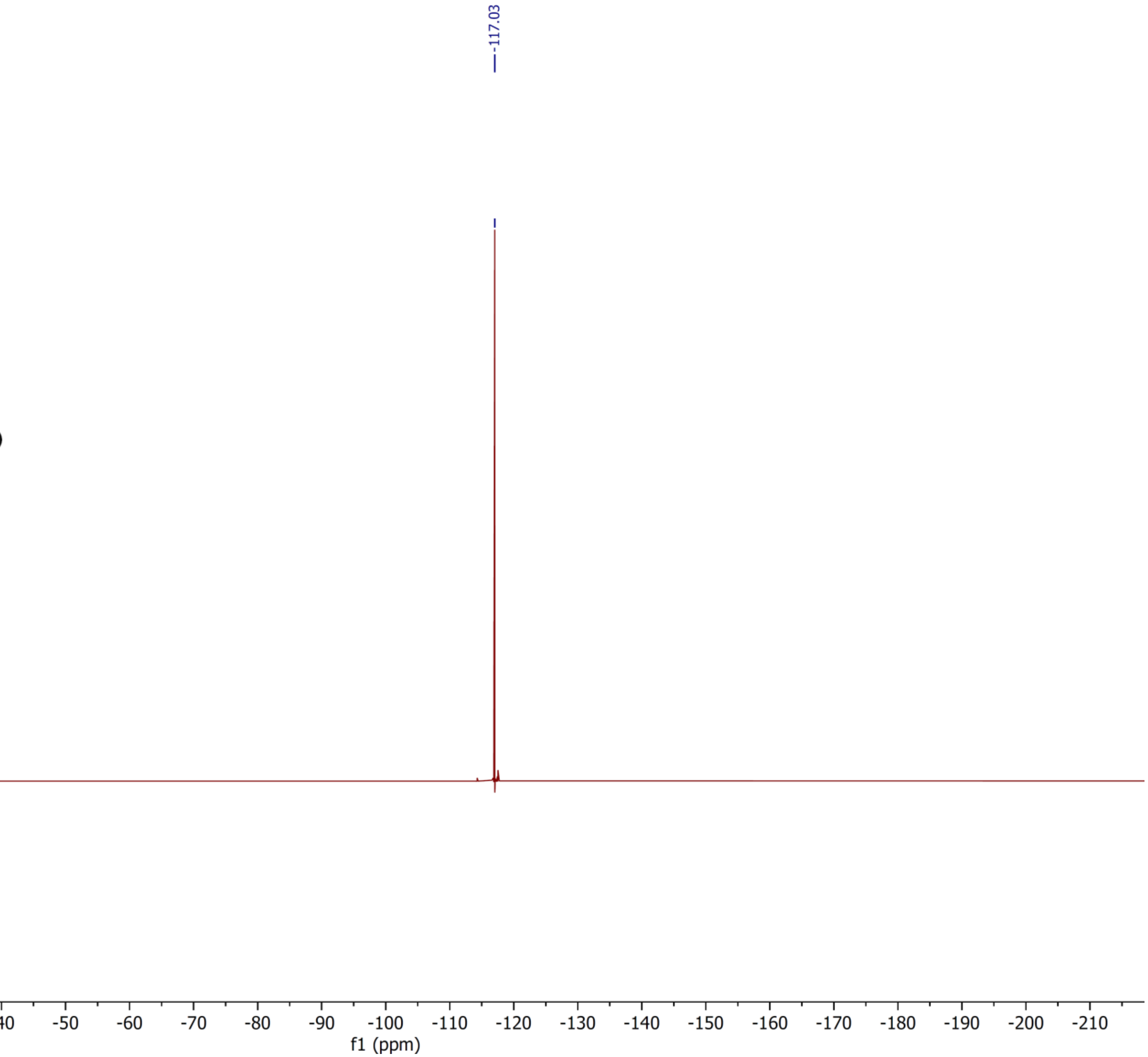

DP-5-107-H

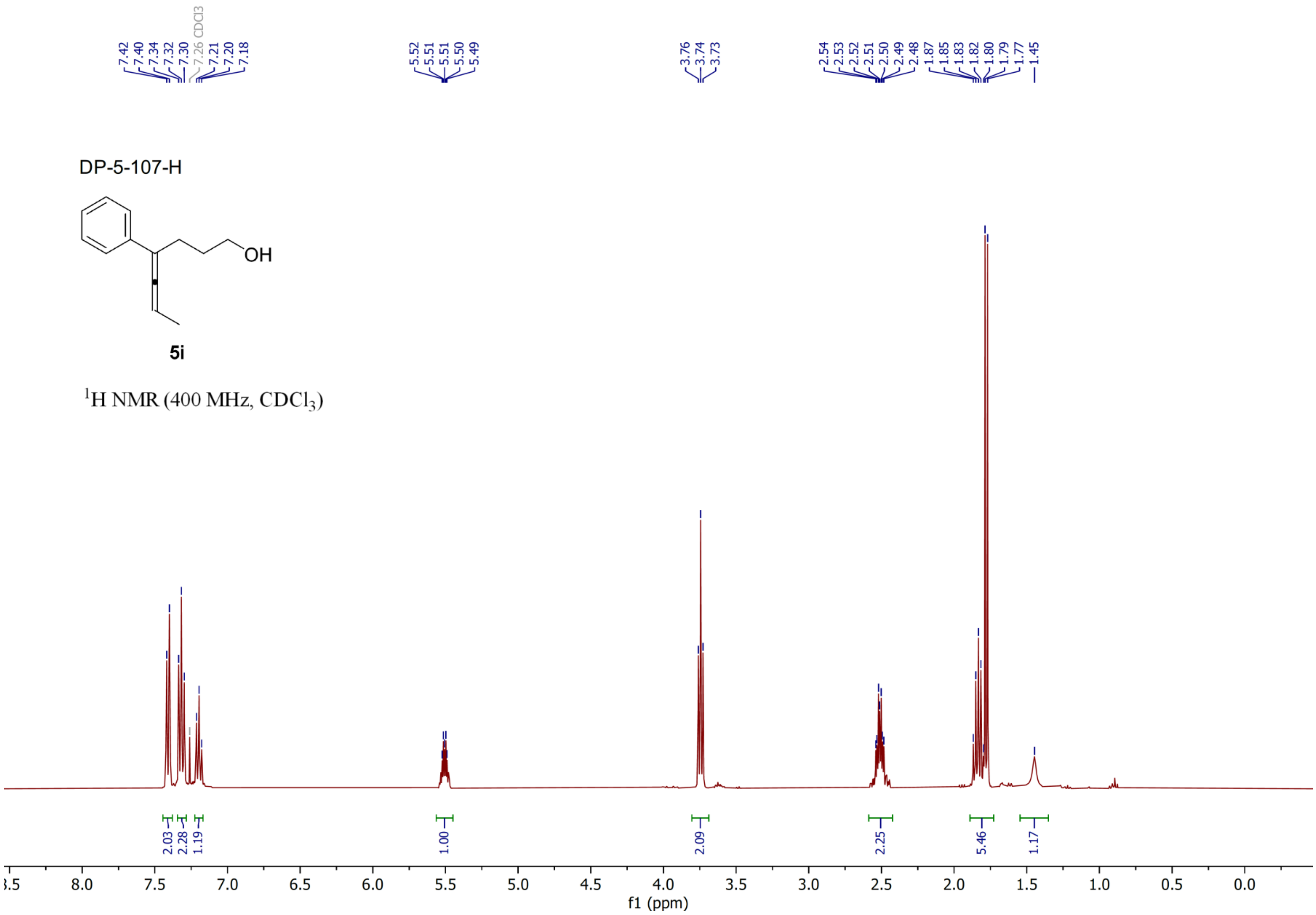

5i

^{1}H NMR (400 MHz, $CDCl_3$)

DP-5-107-C

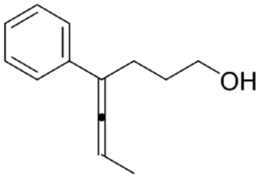

5i

^{13}C NMR (101 MHz, $CDCl_3$)

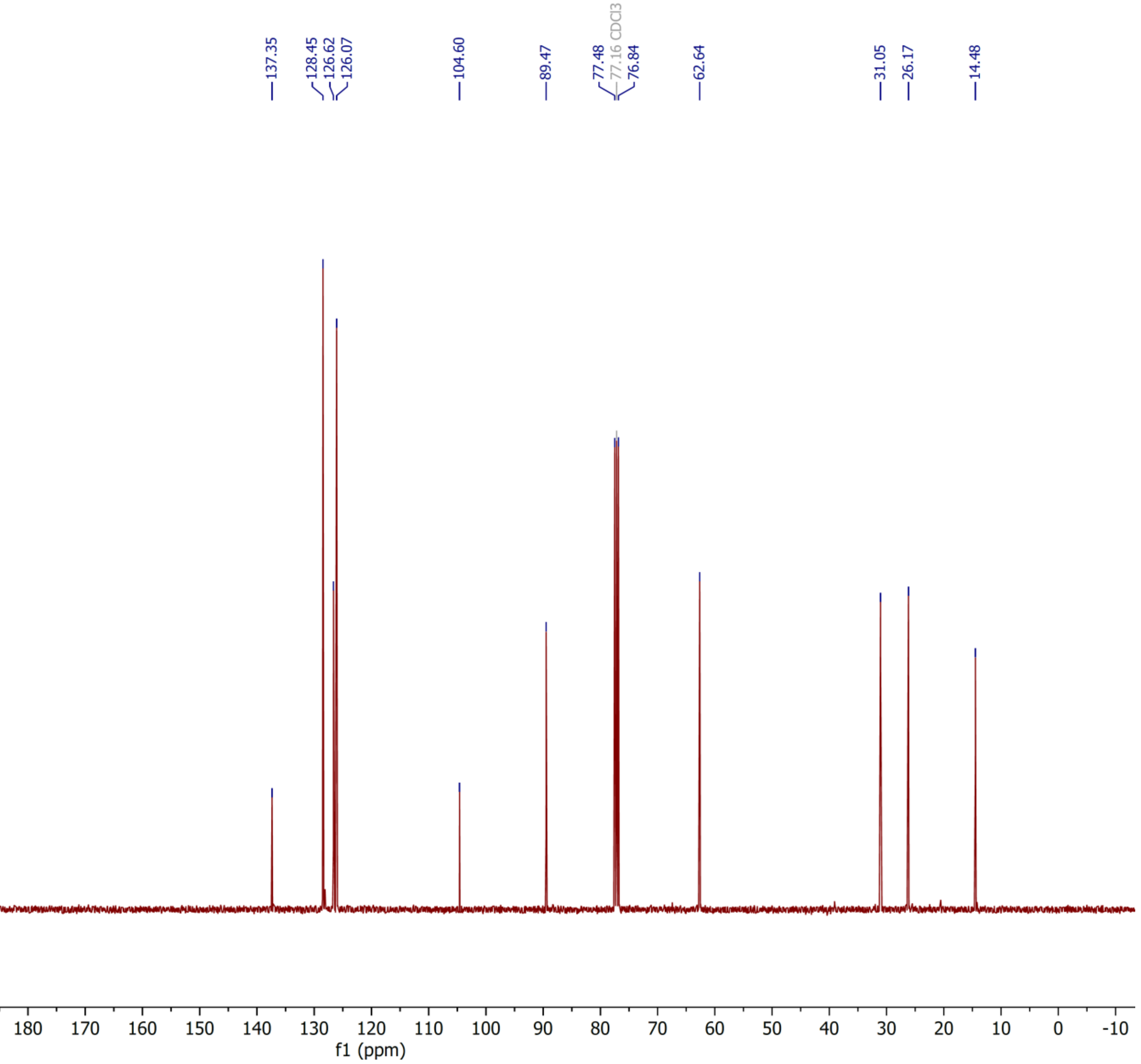

^{1}H and ^{13}C NMR spectra of compounds 7a – 7e, 8 and 9:

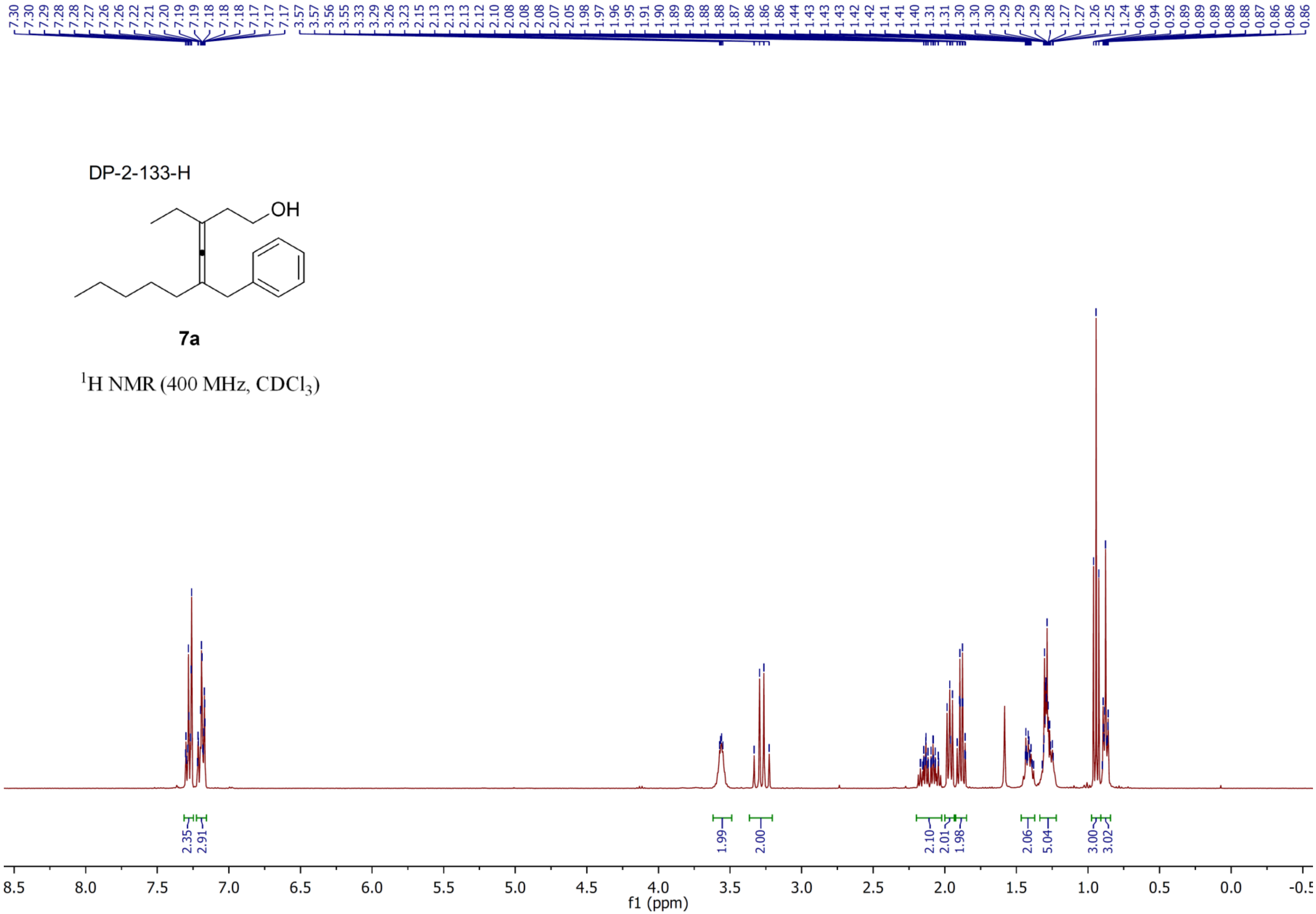

DP-2-133-C

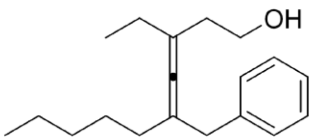

7a

^{13}C NMR (101 MHz, $CDCl_3$)

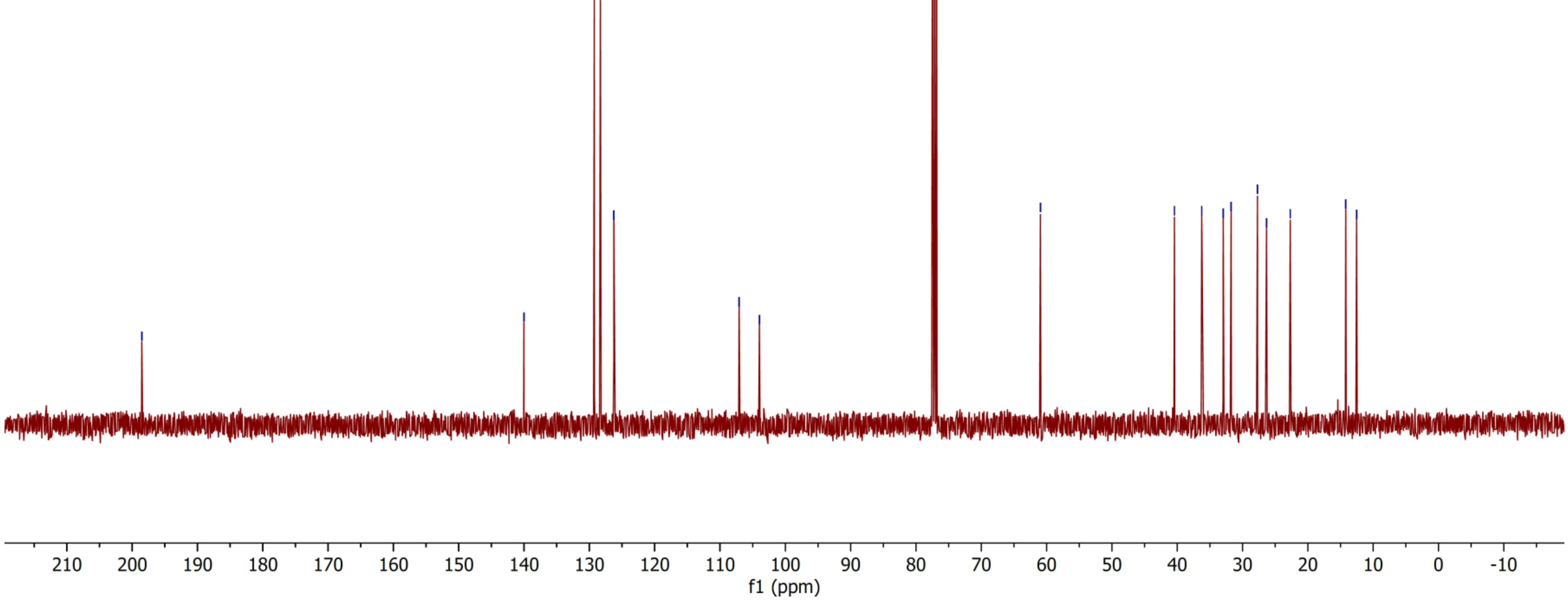

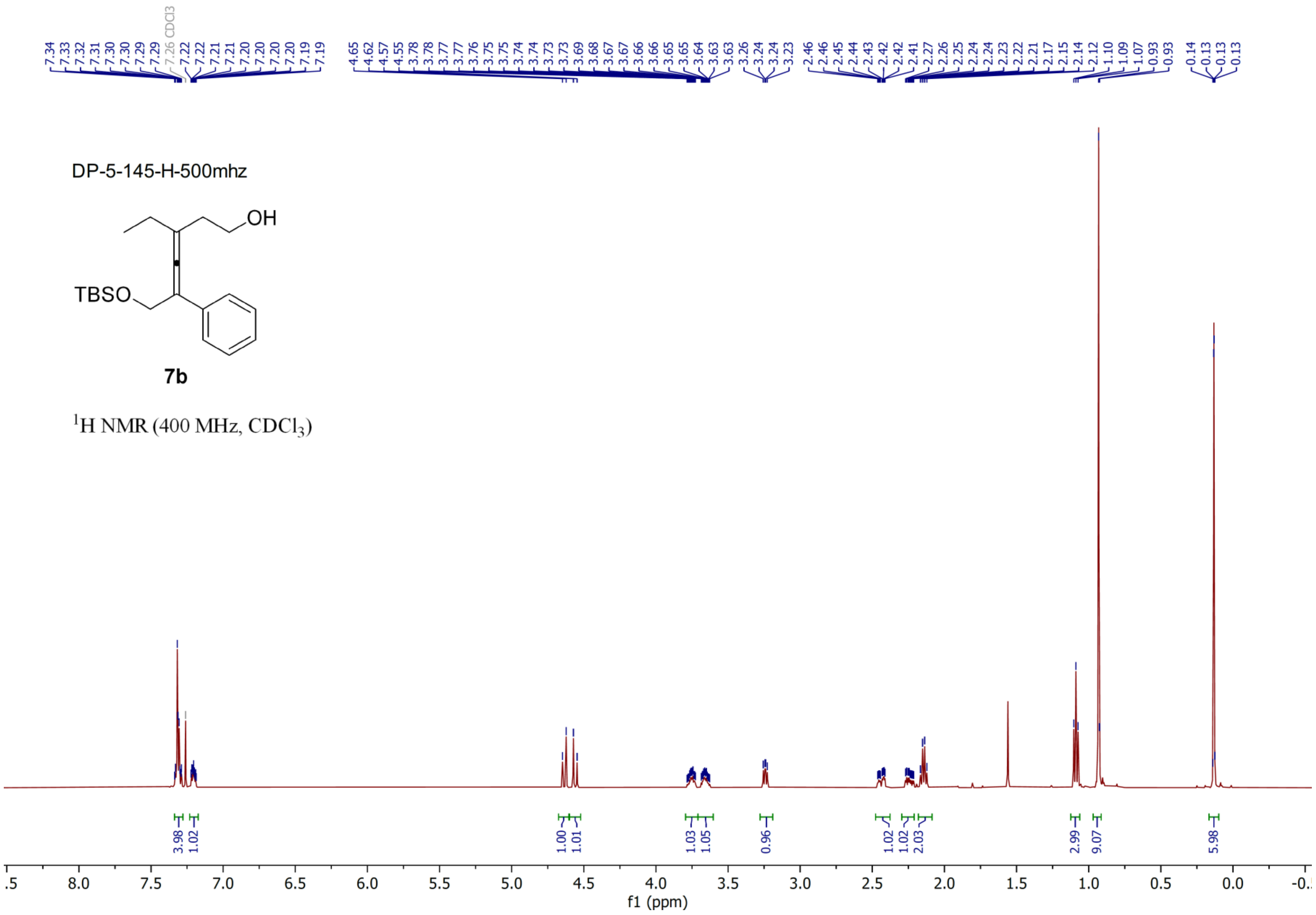
DP-5-145-H-500mhz
OH
TBSO
7b
^{1}H NMR (400 MHz, CDCl$_3$)
f1 (ppm)

DP-5-145-C-500mhz

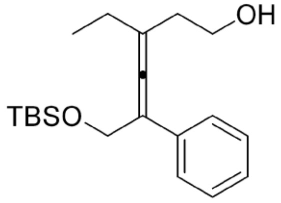

7b

^{13}C NMR (101 MHz, $CDCl_3$)

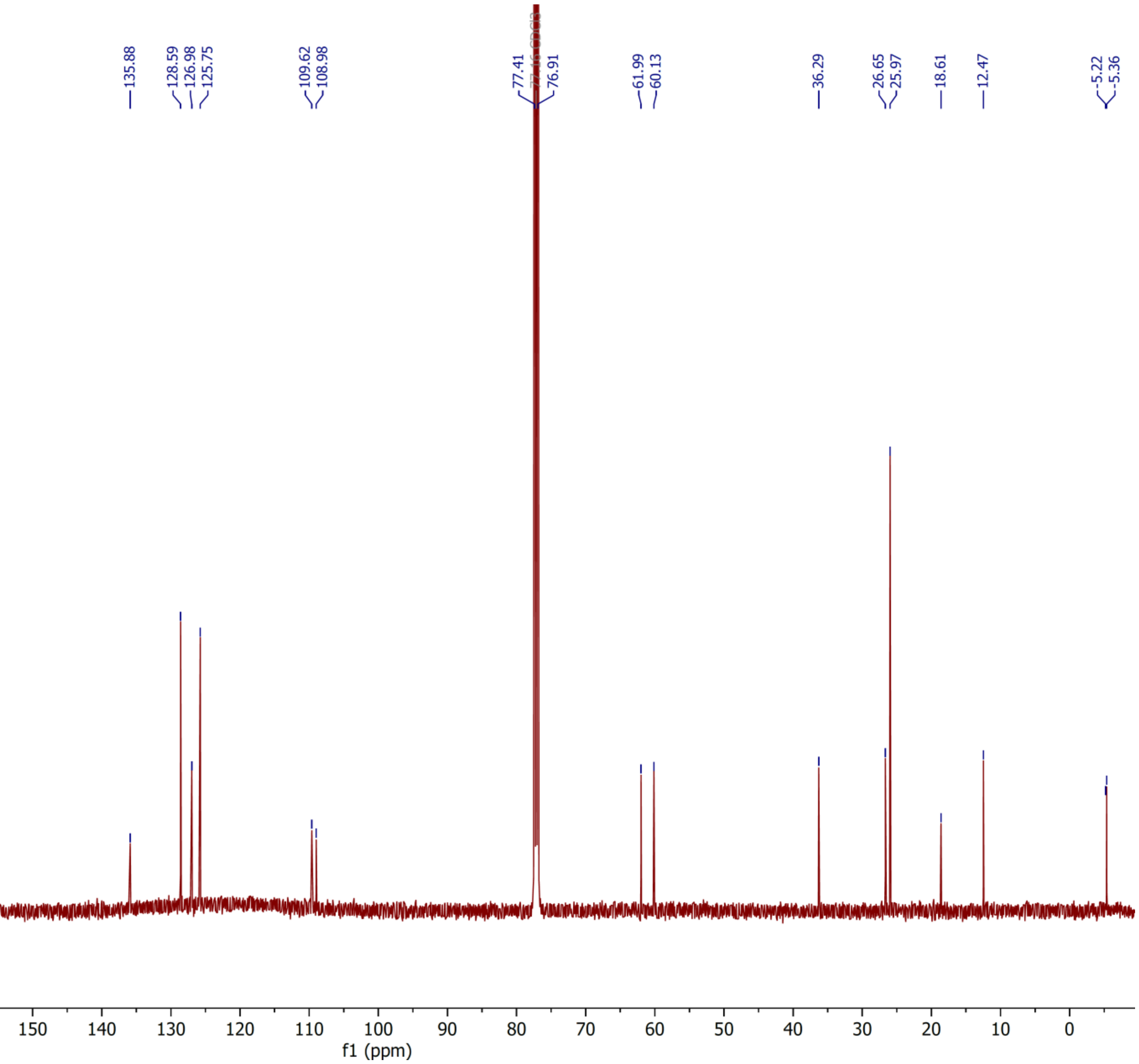

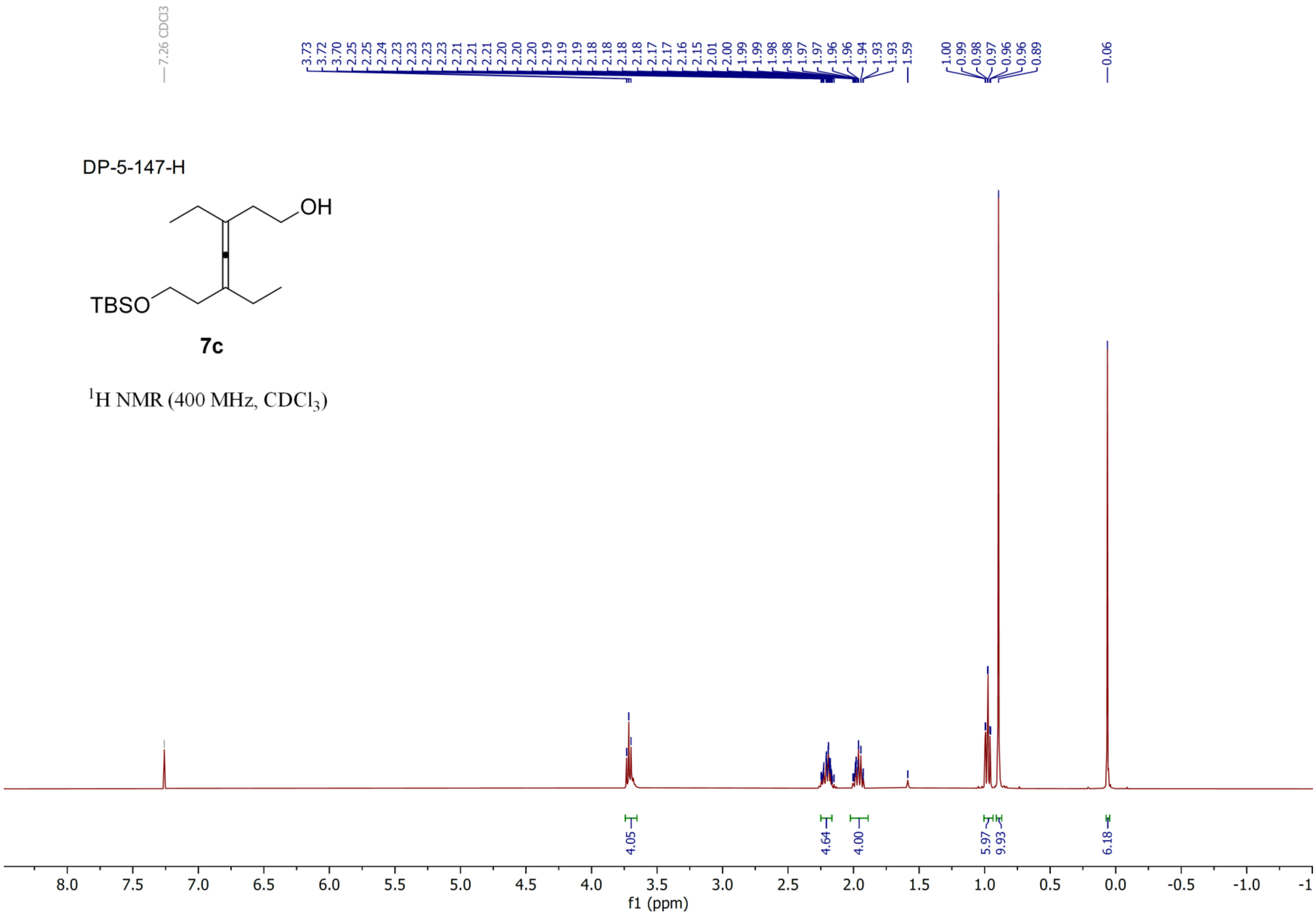
DP-5-147-H
OH
TBSO
7c
^{1}H NMR (400 MHz, $CDCl_3$)
7.26 CDCl3
3.73 3.72 3.70 2.25 2.25 2.24 2.23 2.23 2.23 2.23 2.21 2.21 2.21 2.20 2.20 2.19 2.19 2.19 2.18 2.18 2.18 2.18 2.17 2.17 2.16 2.15 2.01 2.00 1.99 1.99 1.98 1.98 1.97 1.97 1.96 1.96 1.94 1.93 1.93 1.59
1.00 0.99 0.98 0.97 0.96 0.96 0.89
0.06
4.05
4.64
4.00
5.97
9.93
6.18
8.0 7.5 7.0 6.5 6.0 5.5 5.0 4.5 4.0 3.5 3.0 2.5 2.0 1.5 1.0 0.5 0.0 -0.5 -1.0 -1
f1 (ppm)

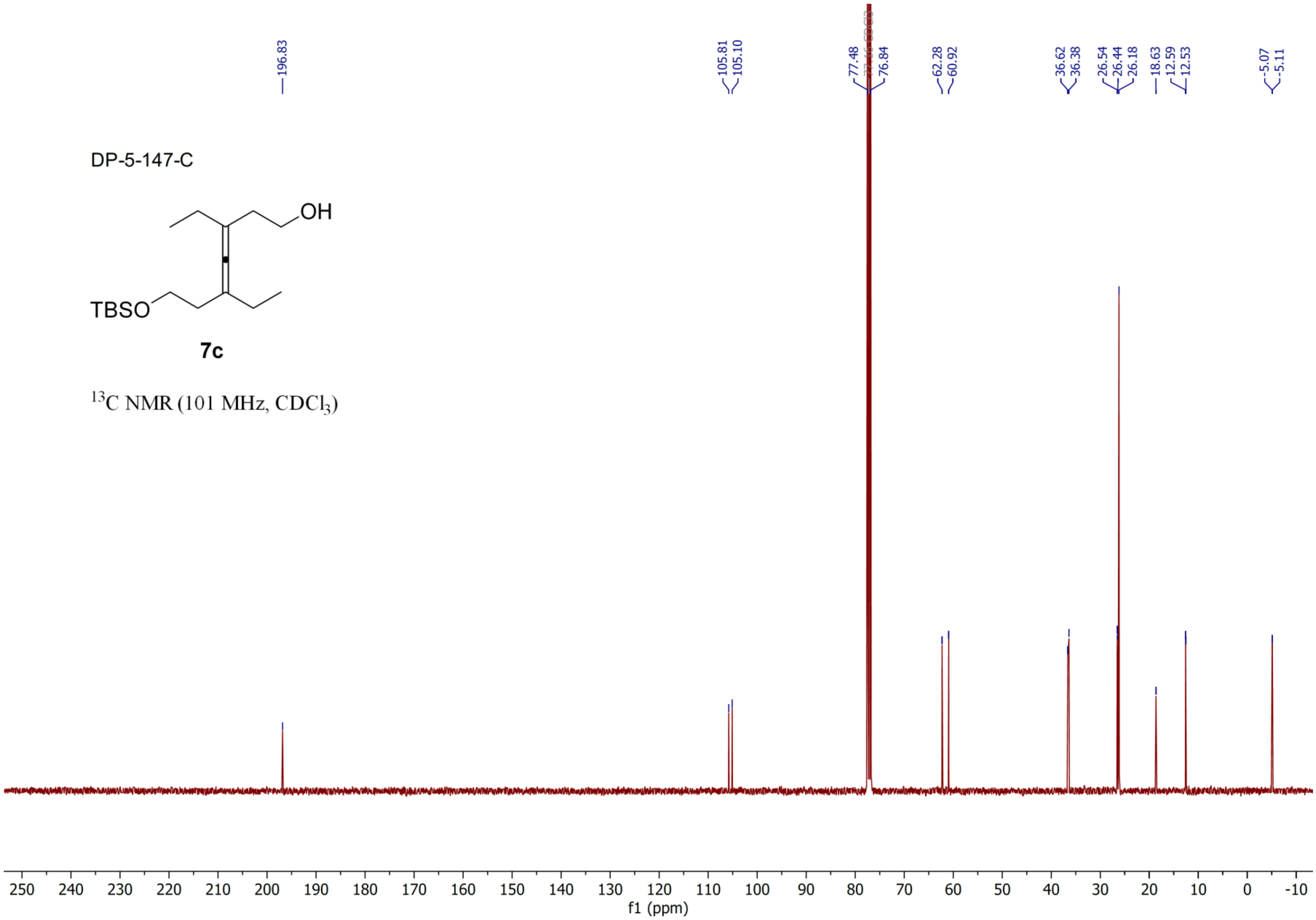

DP-5-147-C
OH
TBSO
7c
13C NMR (101 MHz, CDCl3)
196.83
105.81
105.10
77.48
77.16 CDCl3
76.84
62.28
60.92
36.62
36.38
26.54
26.44
26.18
18.63
12.59
12.53
-5.07
-5.11
250
240
230
220
210
200
190
180
170
160
150
140
130
120
110
100
90
80
70
60
50
40
30
20
10
0
-10
f1 (ppm)

DP-5-146-H

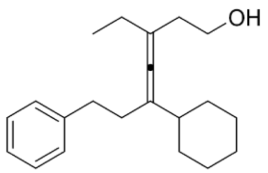

7d

^{1}H NMR (400 MHz, $CDCl_3$)

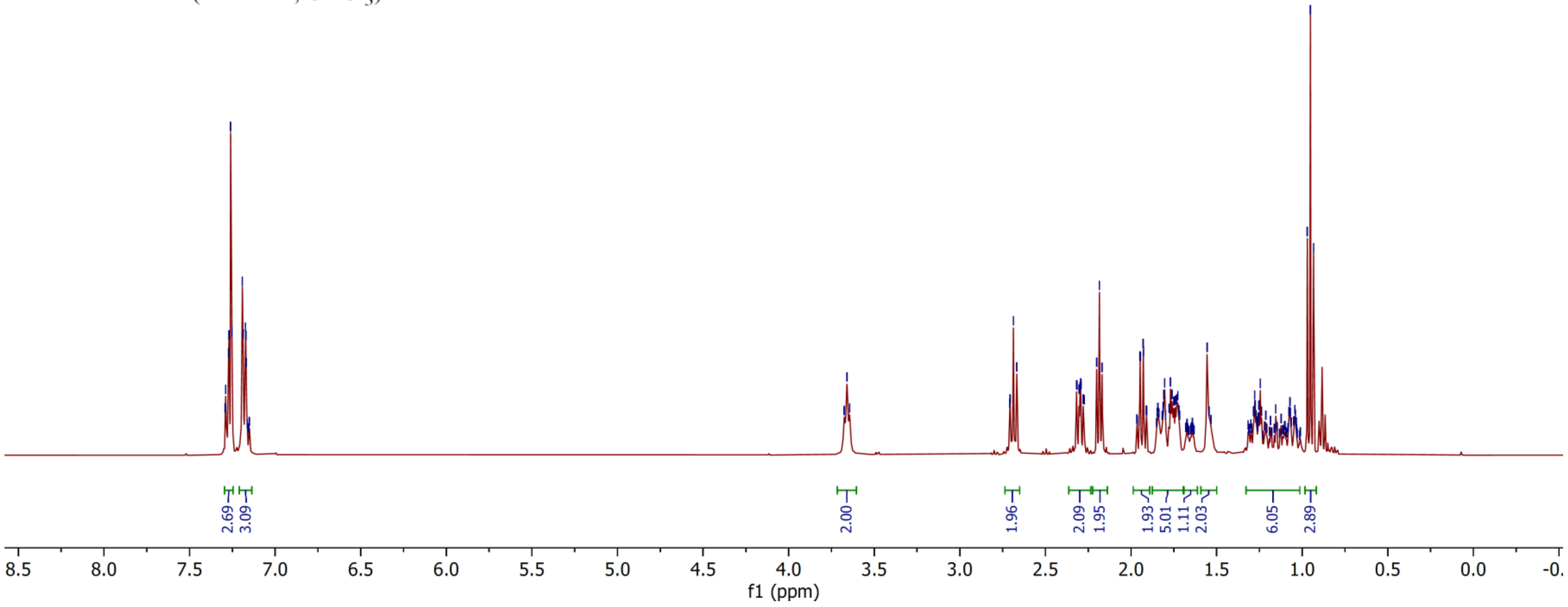

DP-5-146-C

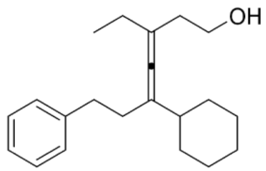

7d

^{13}C NMR (101 MHz, $CDCl_3$)

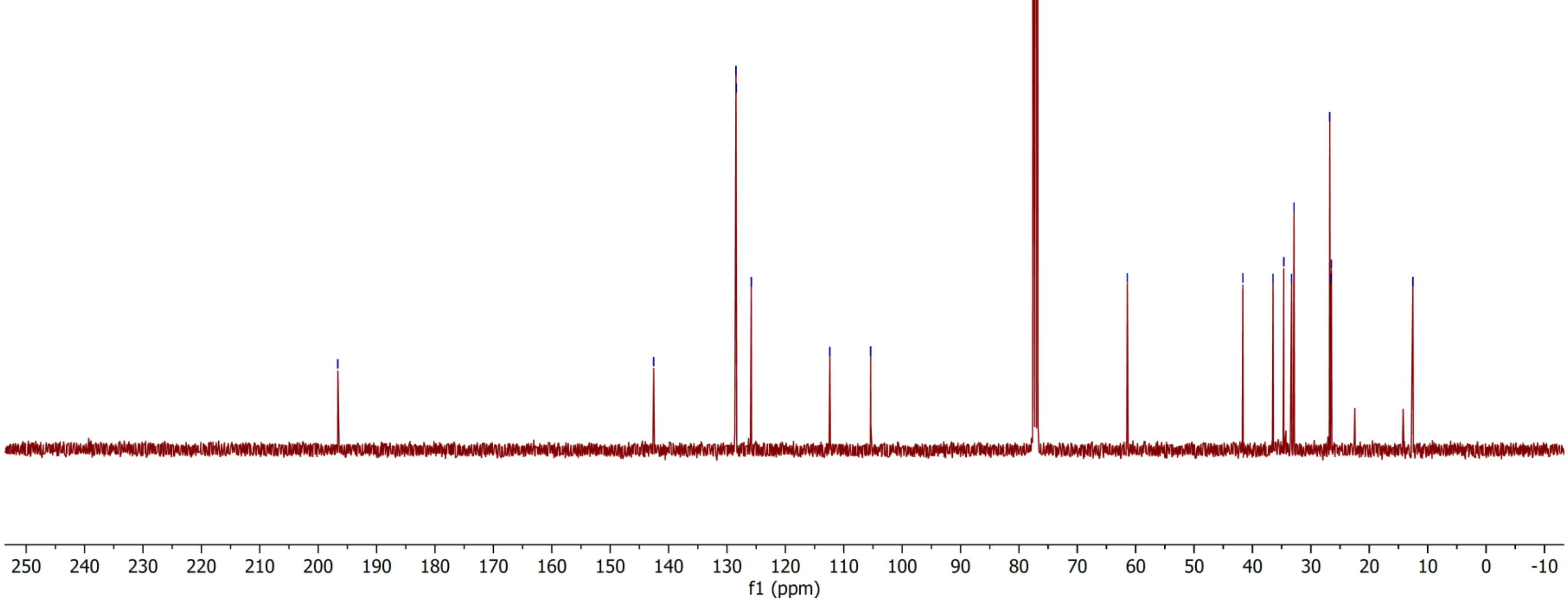

DP-5-144-H-500mhz

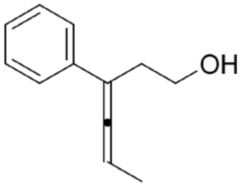

7e

^{1}H NMR (500 MHz, $CDCl_3$)

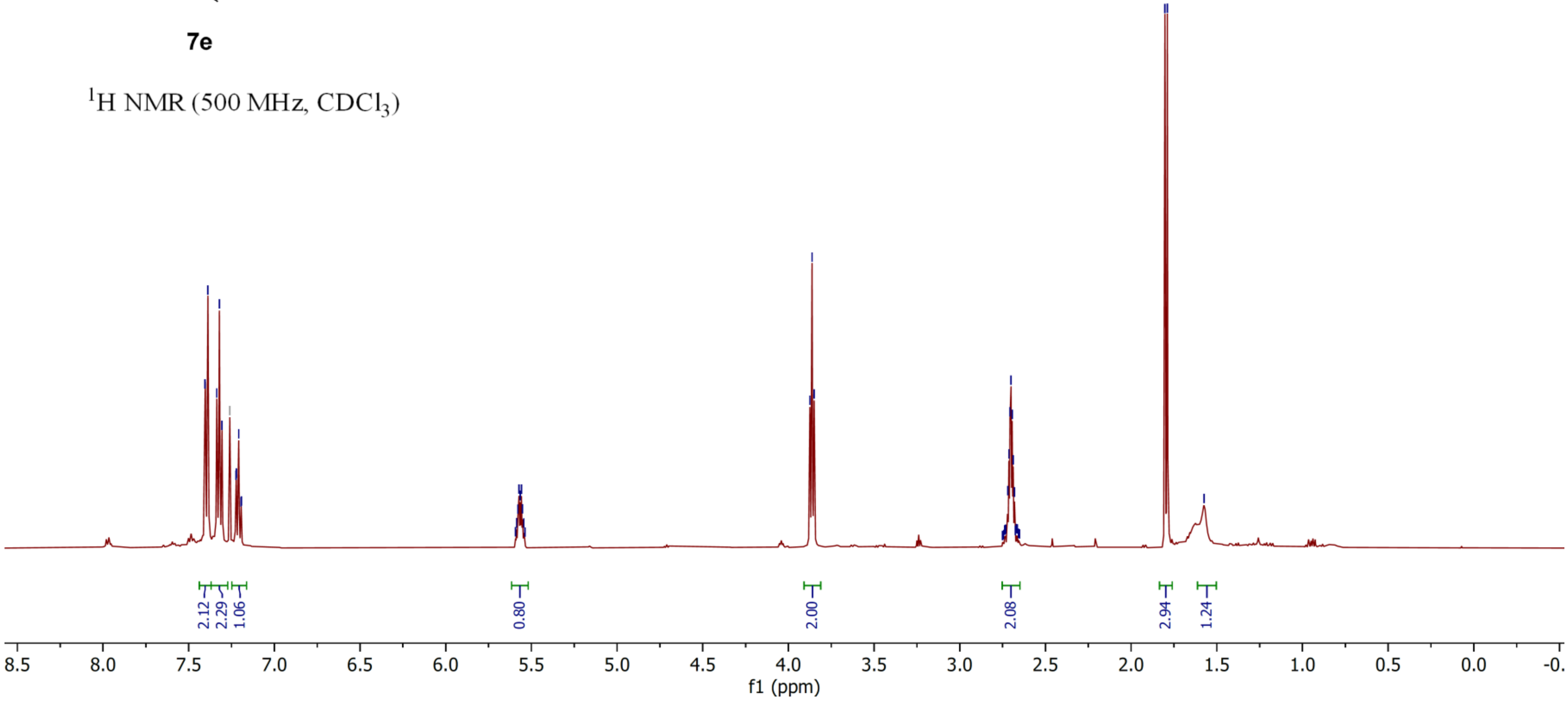

DP-5-144-C-500mhz

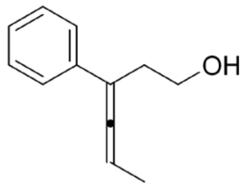

7e

^{13}C NMR (126 MHz, $CDCl_3$)

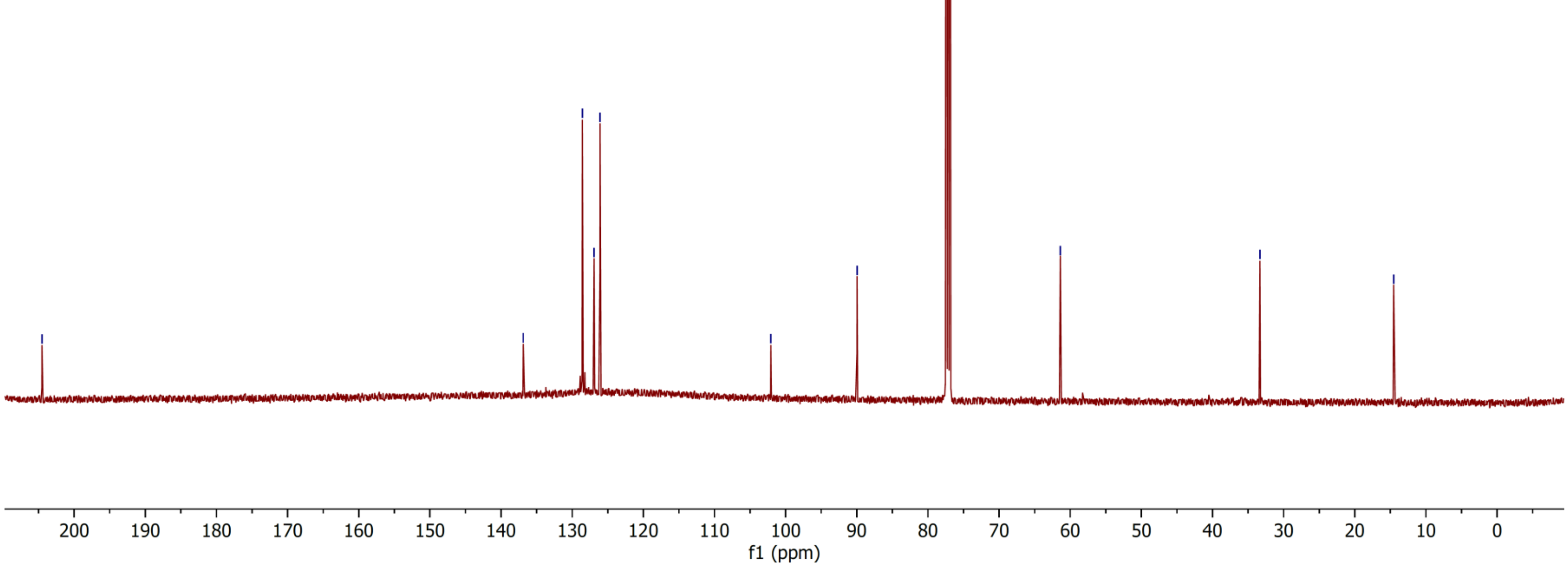

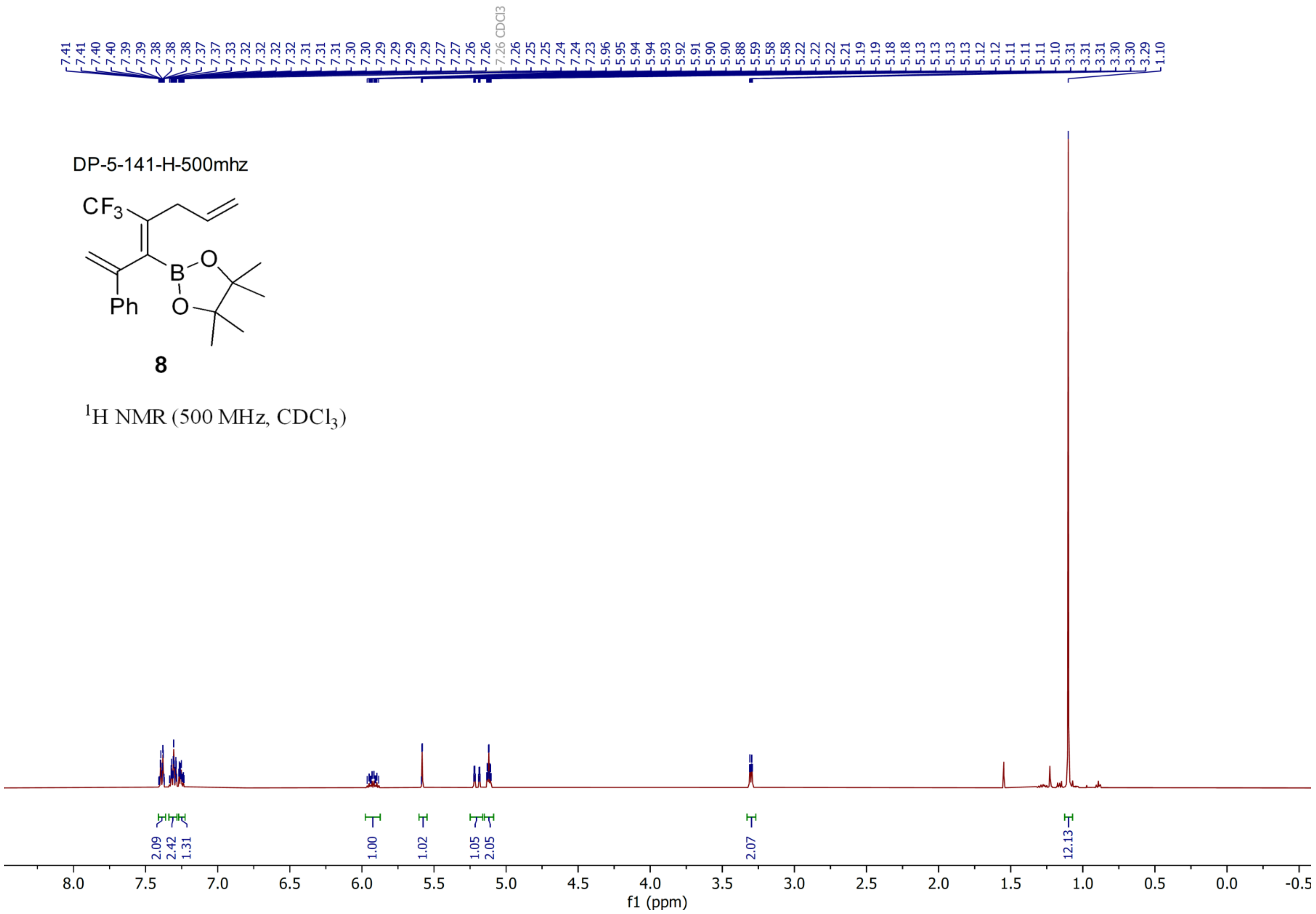

7.41
7.41
7.40
7.40
7.39
7.39
7.38
7.38
7.38
7.37
7.37
7.33
7.32
7.32
7.32
7.32
7.31
7.31
7.31
7.30
7.30
7.29
7.29
7.29
7.29
7.27
7.27
7.26
7.26
7.26 CDCl3
7.26
7.25
7.25
7.24
7.24
7.23
5.96
5.95
5.94
5.94
5.93
5.92
5.91
5.90
5.90
5.88
5.59
5.58
5.58
5.22
5.22
5.22
5.21
5.19
5.19
5.18
5.18
5.13
5.13
5.13
5.13
5.12
5.12
5.11
5.11
5.11
5.10
3.31
3.31
3.31
3.30
3.30
3.29
1.10
DP-5-141-H-500mhz
CF3
B
O
O
Ph
8
¹H NMR (500 MHz, CDCl3)
2.09
2.42
1.31
1.00
1.02
1.05
2.05
2.07
12.13
8.0
7.5
7.0
6.5
6.0
5.5
5.0
4.5
4.0
3.5
3.0
2.5
2.0
1.5
1.0
0.5
0.0
-0.5
f1 (ppm)

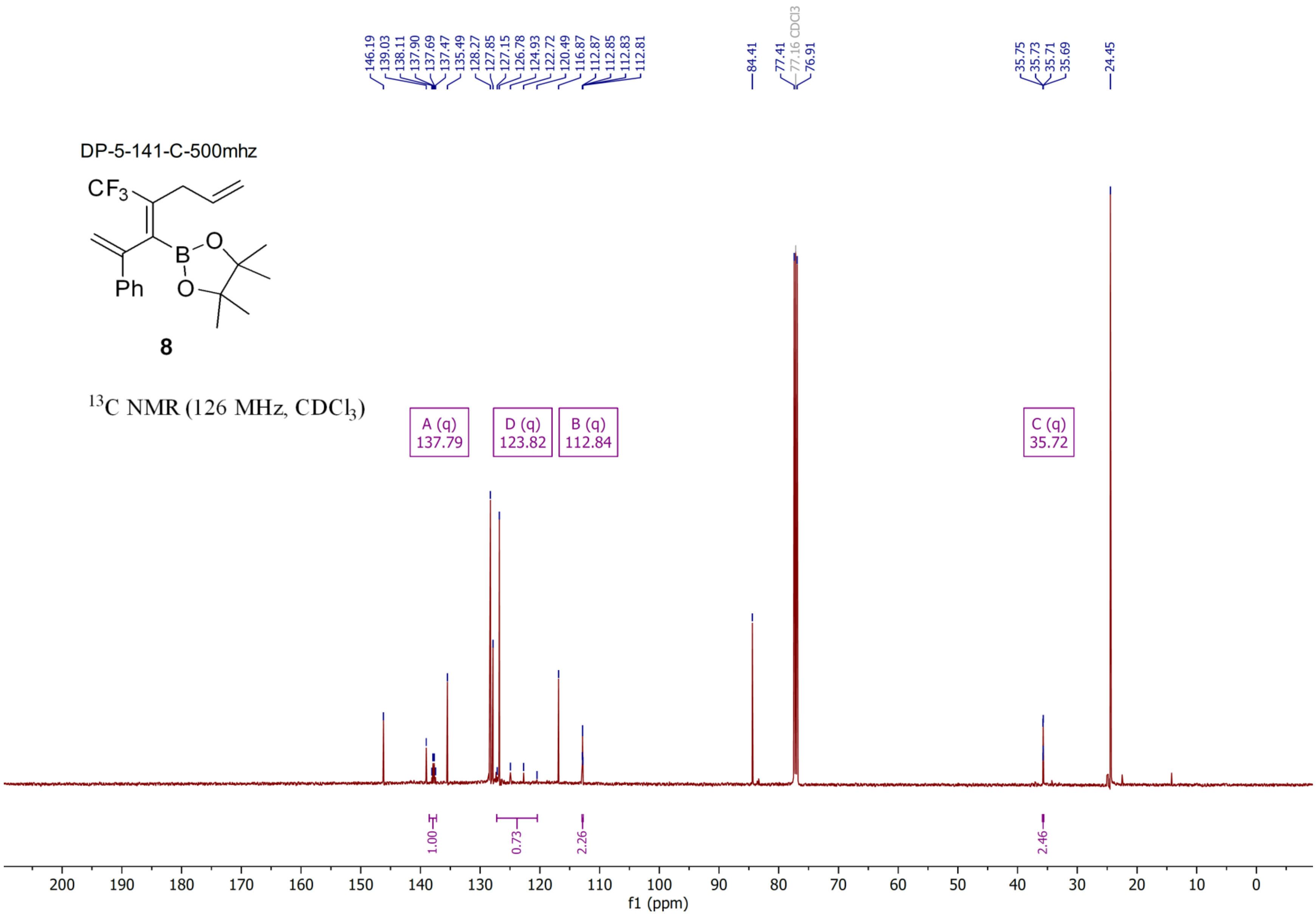

DP-5-141-C-500mhz
CF3
B
O
O
Ph
8
13C NMR (126 MHz, CDCl3)
146.19
139.03
138.11
137.90
137.69
137.47
135.49
128.27
127.85
127.15
126.78
124.93
122.72
120.49
116.87
112.87
112.85
112.83
112.81
84.41
77.41
77.16 CDCl3
76.91
35.75
35.73
35.71
35.69
24.45
A (q)
137.79
D (q)
123.82
B (q)
112.84
C (q)
35.72
1.00
0.73
2.26
2.46
200
190
180
170
160
150
140
130
120
110
100
90
80
70
60
50
40
30
20
10
0
f1 (ppm)

DP-5-141-F

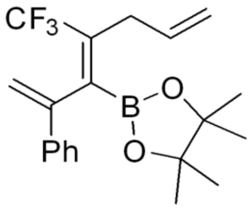

8

^{19}F NMR (377 MHz, $CDCl_3$)

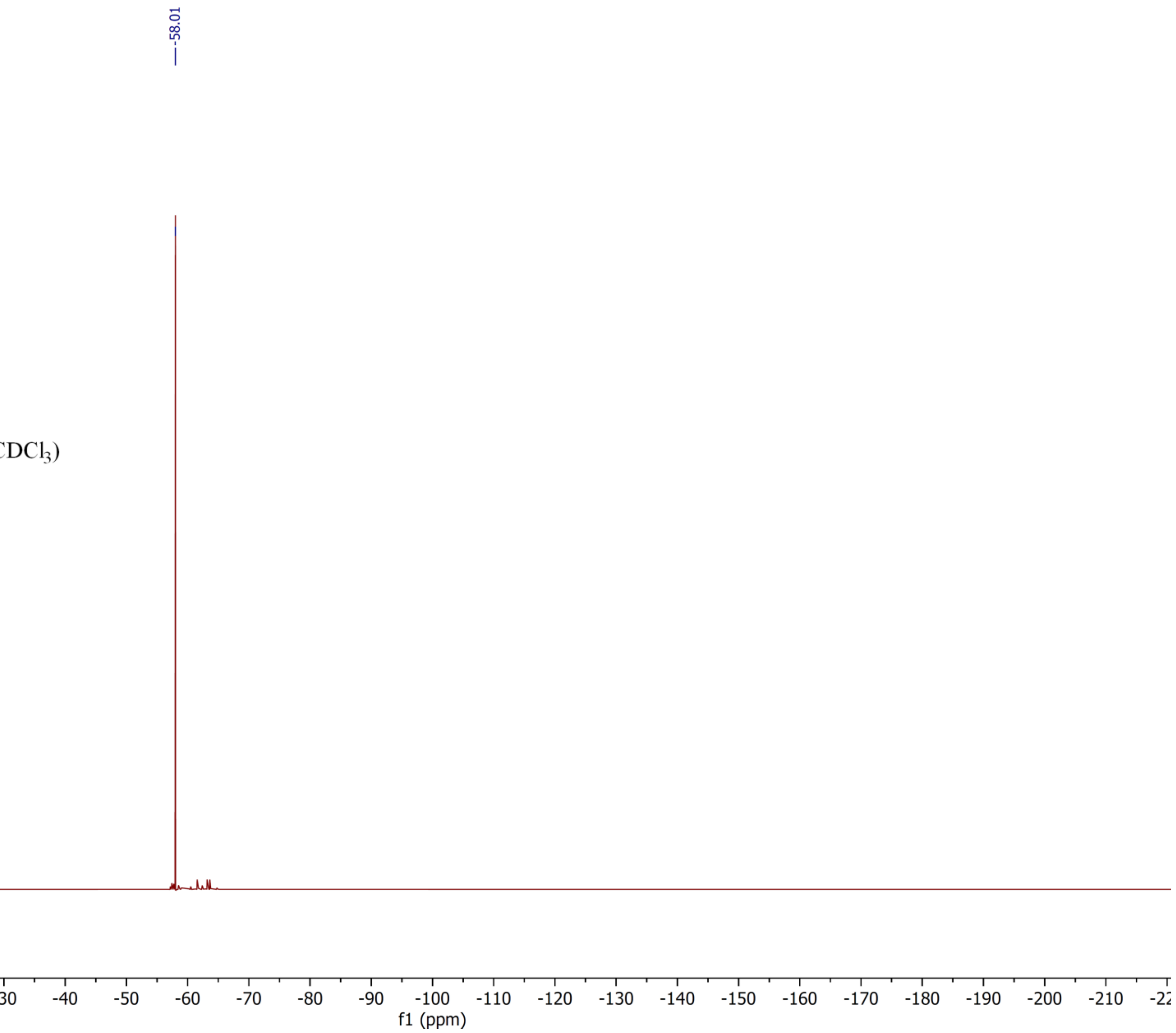

DP-5-141-NOESY-500mhz

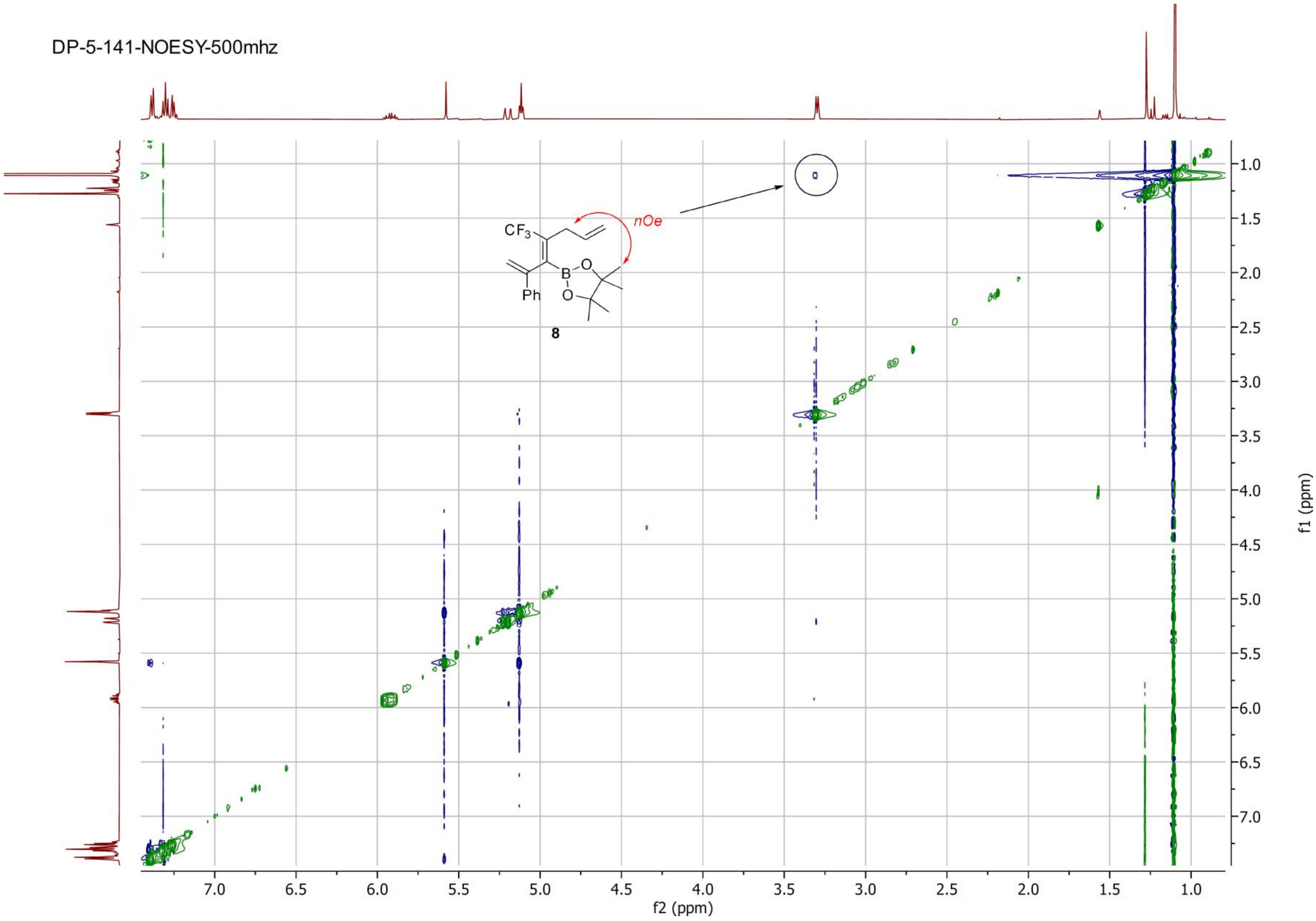

DP-5-193-ester-H

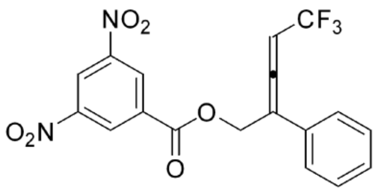

9

^{1}H NMR (400 MHz, $CDCl_3$)

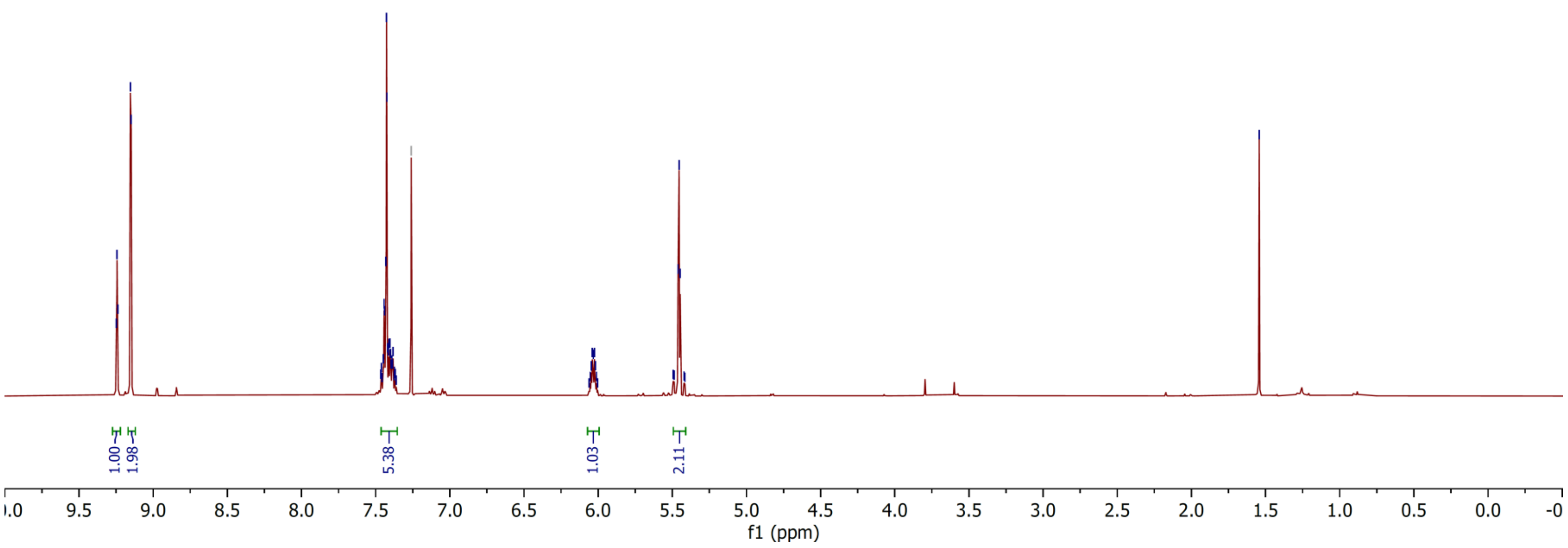

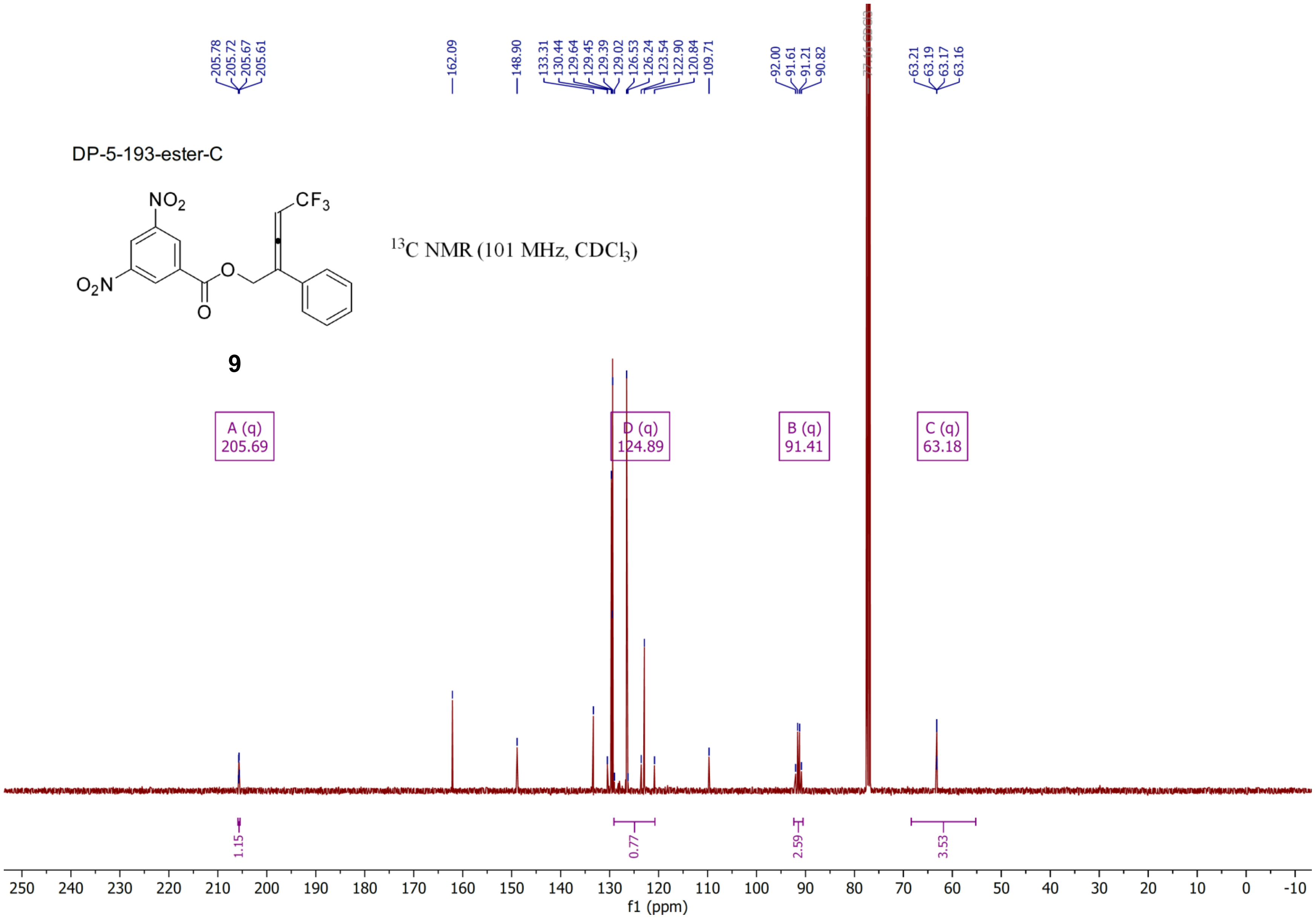
205.78
205.72
205.67
205.61
162.09
148.90
133.31
130.44
129.64
129.45
129.39
129.02
126.53
126.24
123.54
122.90
120.84
109.71
92.00
91.61
91.21
90.82
63.21
63.19
63.17
63.16
DP-5-193-ester-C
NO2
CF3
O2N
O
O
9
13C NMR (101 MHz, CDCl3)
A (q)
205.69
D (q)
124.89
B (q)
91.41
C (q)
63.18
1.15
0.77
2.59
3.53
250 240 230 220 210 200 190 180 170 160 150 140 130 120 110 100 90 80 70 60 50 40 30 20 10 0 -10
f1 (ppm)

DP-5-193-ester-F

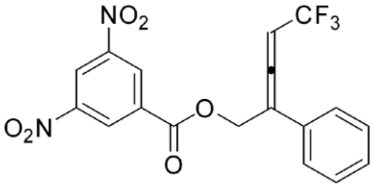

9

^{19}F NMR (377 MHz, $CDCl_3$)

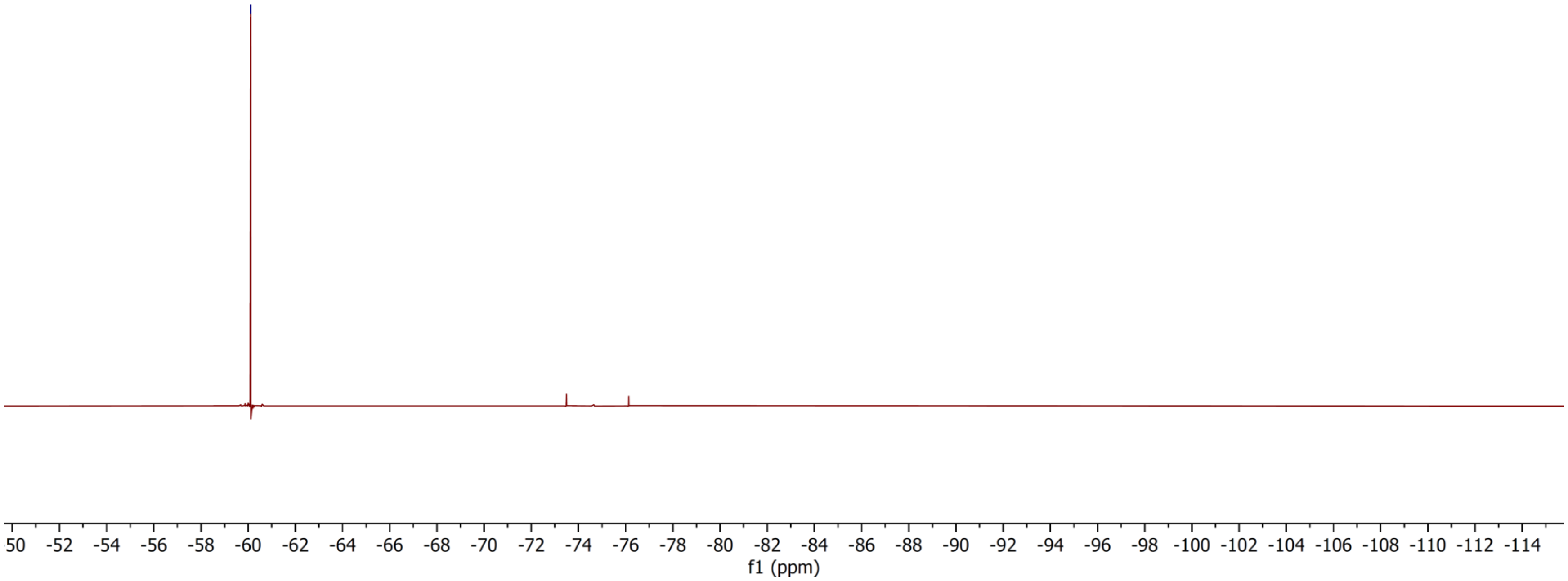

Supplement: Supplementary file 1 — Supporting Information [file ANIE-60-22178-s001.pdf]
